# Induction of ER and mitochondrial stress by the alkylphosphocholine erufosine in oral squamous cell carcinoma cells

**DOI:** 10.1038/s41419-018-0342-2

**Published:** 2018-02-20

**Authors:** Shariq S. Ansari, Ashwini K. Sharma, Himanshu Soni, Doaa M. Ali, Björn Tews, Rainer König, Hansjörg Eibl, Martin R. Berger

**Affiliations:** 1 Toxicology and Chemotherapy Unit, German Cancer Research Center, Heidelberg, Germany; 2 Institute for Pharmacy and Molecular Biotechnology (IPMB) and BioQuant, Heidelberg University, Heidelberg, Germany; 3 Molecular Mechanisms of Tumor Cell Invasion, German Cancer Research Center, Heidelberg, Germany; 4 Integrated Research and Treatment Center, Center for Sepsis Control and Care (CSCC), Jena University Hospital, Jena, Germany; 5 Network Modeling, Leibniz Institute for Natural Products Research and Infection Biology, Hans-Knöll-Institute, Jena, Germany; 6 Max Planck Institute of Biophysical Chemistry, Gottingen, Germany

## Abstract

Endoplasmic reticulum (ER) plays an essential role in cell function and survival. Accumulation of unfolded or misfolded proteins in the lumen of the ER activates the unfolded protein response (UPR), resulting in ER stress and subsequent apoptosis. The alkylphosphocholine erufosine is a known Akt-mTOR inhibitor in oral squamous cell carcinoma (OSCC). In the present study, we evaluate erufosine’s role to induce ER and mitochondrial stress leading to autophagy, apoptosis, and ROS induction. The cellular toxicity of erufosine was determined in two OSCC cell lines and gene expression and enrichment analyses were performed. A positive enrichment of ER stress upon erufosine exposure was observed, which was verified at protein levels for the ER stress sensors and their downstream mediators. Knockdown and pharmacological inhibition of the ER stress sensors PERK and XBP1 revealed their involvement into erufosine’s cellular effects, including proliferation, apoptosis, and autophagy induction. Autophagy was confirmed by increased acidic vacuoles and LC3-B levels. Upon erufosine exposure, calcium influx into the cytoplasm of the two OSCC cell lines was seen. Apoptosis was confirmed by nuclear staining, Annexin-V, and immunoblotting of caspases. The induction of mitochondrial stress upon erufosine exposure was predicted by gene set enrichment analysis (GSEA) and shown by erufosine’s effect on mitochondrial membrane potential, ATP, and ROS production in OSCC cells. These data show that ER and mitochondrial targeting by erufosine represents a new facet of its mechanism of action as well as a promising new framework in the treatment of head and neck cancers.

## Introduction

Head and neck squamous cell cancer (HNSCC) comprises a heterogeneous group of tumors^1^. Oral squamous cell carcinoma (OSCC) constitutes 90% of the total HNSCC cases and is the sixth most prevalent cancer worldwide^2^. HNSCC accounts for about 3% of all cancers in the United States^3^. The incidence of OSCC is higher in South East Asian countries than the Western world^4^. About one-third of patients are diagnosed with early stage disease, whereas the majority of cases are diagnosed with advanced stage cancer with lymph node metastasis^5^. About 60% of patients undergoing surgical removal show local recurrence and metastasis is seen in 15–20% of cases^6^. About 40–50% of patients with HNSCC survive for 5 years^2^. When detected at an early stage, the probability of survival is 90%.

Alcohol intake and tobacco use are the most prominent risk factors for HNSCC being responsible for at least 75% of its incidence^7^. People using both, tobacco and alcohol, are at greater risk than those who use either of the habits alone^7–9^.

Erufosine (erucylphospho-*N*,*N*,*N*-trimethylpropanolamine), a third-generation alkylphosphosphocholine^10^, inhibits proliferation by inducing apoptosis in cells originating from leukemia, breast cancer, colorectal cancer, prostate cancer, oral squamous cell carcinoma, human astrocytoma, and glioblastoma cell lines, both in vitro and in vivo^11–21^. For its long 22-carbon chain and ω-9 cis-double bond, erufosine lacks hemolytic toxicity and is therefore suitable for intravenous administration^22,23^. Erufosine has been shown to downregulate PI3K, c-Raf, and Akt proteins in breast cancer, both, in vitro and in vivo^13^. Also, downregulation of the Akt/m-TOR pathway^19^, inhibition of cell cycle processes, and increased expression of RhoB were shown in OSCC cells^20^.

The endoplasmic reticulum (ER) is a membrane-bound organelle, playing an important role in protein folding, processing, and trafficking, besides maintaining cell homeostasis^24^. It is the major organelle for Ca^2+^ regulation and synthesis of secretory proteins. Accumulation of unfolded or misfolded proteins activates the unfolded protein response (UPR), resulting in ER stress. UPR is sensed by three ER stress sensors, inositol requiring enzyme 1 (IRE-1), PKR‑like ER kinase (PERK), and activating transcription factor-6 (ATF-6), which are downstream components of ER chaperones^25^. Disturbance in Ca^2+^ homeostasis, accumulation of misfolded proteins, and inhibition of phosphatidylcholine disrupt the ER–Golgi network resulting in ER stress^26,27^. Excessive ER stress leads to apoptosis induction, mediated by the pro-apoptotic transcription factor CCAAT/enhancer-binding protein-homologous protein (CHOP) through the activation of PERK-eIF-2α axis, and JNK activation via the IRE-1α receptor. Besides induction of apoptosis, ER stress also induces autophagy for restoring cellular homeostasis. Further, accumulation of reactive oxygen species (ROS) due to chemotherapeutic agents is known to cause mitochondrial dysfunction and induce apoptosis^28^.

In the present study, we examine erufosine’s ability to induce ER stress, which results into induction of apoptosis and autophagy, and its effect on accumulation of ROS, which leads to mitochondrial dysfunction in OSCC cells.

## Results

### Cytotoxicity of OSCC cells

**Fig. 1:**
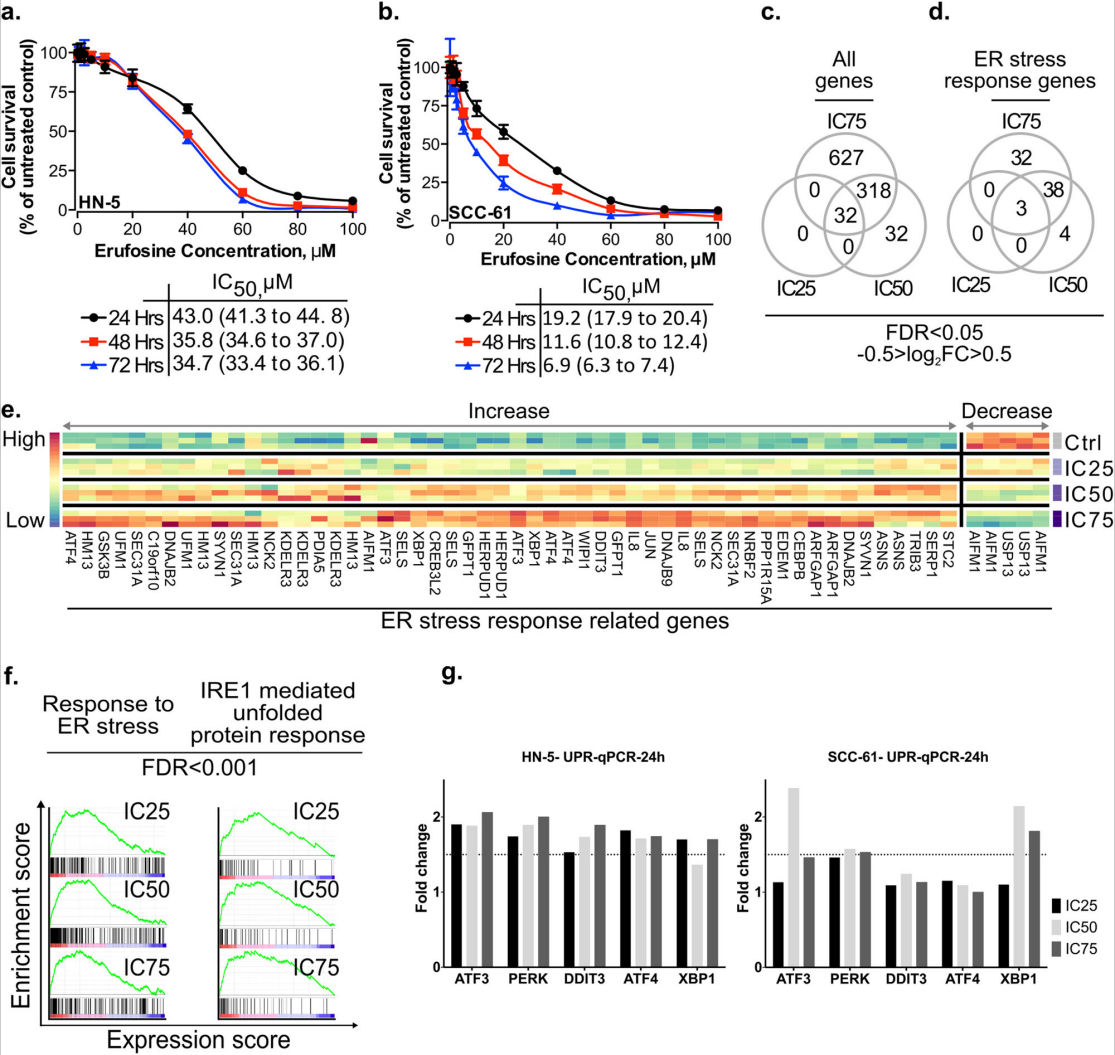
Cytotoxic concentrations of erufosine induce ER stress in HN5 and SCC61 cells. Cytotoxic effect of erufosine in OSCC cell lines HN-5 (**a**) and SCC-61 (**b**) as determined by MTT assay at 24, 48, and 72 h post treatment. The respective IC_50_ values are given below the graph. **c** Overlap among all genes (**c**) and ER stress-response genes (**d**), which are differentially expressed upon erufosine exposure at IC_25_, IC_50_, and IC_75_ concentrations. **e** Heat map of differentially expressed ER stress-response genes for at least two different concentrations of erufosine. (**f**) Positively correlated enrichment of ER stress-related pathways and transcription factors in erufosine-treated HN-5 cells (**g**) qRT-PCR verification of ER stress genes viz. PERK, ATF3, ATF4, DDIT3, and XPB1, which were upregulated from the microarray data in HN-5 cells post erufosine exposure. Fold changes are depicted as averages of triplicate experiments. The dotted line represents a 1.5-fold change in expression, beyond which a significant change in expression was assumed.

Erufosine exposure caused concentration- and time-dependent cell growth inhibition in HN-5 and SCC-61 cells. The latter were two–fivefold more sensitive to erufosine than the former as derived from their IC_50_ values (Fig.1a, b).

### Erufosine induces ER stress

Analysis of gene expression modulation caused by erufosine in HN-5 cells showed that 32 genes were differentially regulated in response to IC_25_, IC_50_, and IC_75_ concentrations (Fig.1c) (for all modulated genes, see Supplementary Tables S1a, b, c). Relation of these modulated genes to “Hallmarks of cancer” signaling chains revealed that 24, 34, and 35 signaling chains were either increased or decreased (Supplementary Tables S2a, b, c). Specifically, the ER stress-related genes were differentially expressed (Fig.1d, e and Supplementary Tables S3a, b, c). Furthermore, GSEA revealed that gene ontology terms based on processes, which relate to ER stress and stress induction via uncoupling of unfolded proteins were positively enriched (Fig. 1f, FDR <0.001; and Supplementary Tables S4a, b, c).

In order to support these findings, we carried out qRT-PCR analyses of UPR genes responsible for activating ER stress (Fig.1g). In HN-5 cells, the mRNA levels of PERK, ATF4, ATF3, DDIT3, and XBP1 showed more than 1.5-fold upregulation upon erufosine exposure, whereas in SCC-61 cells, upregulation was observed only for PERK, ATF3, and XBP1. Interestingly, the mRNA levels of XBP1 target genes EDEM1, HSPA5, and DNAJB9 were distinctly upregulated in SCC-61, but much less in HN-5 cells upon erufosine exposure (Supp. Fig S1a, b).

**Fig. 2:**
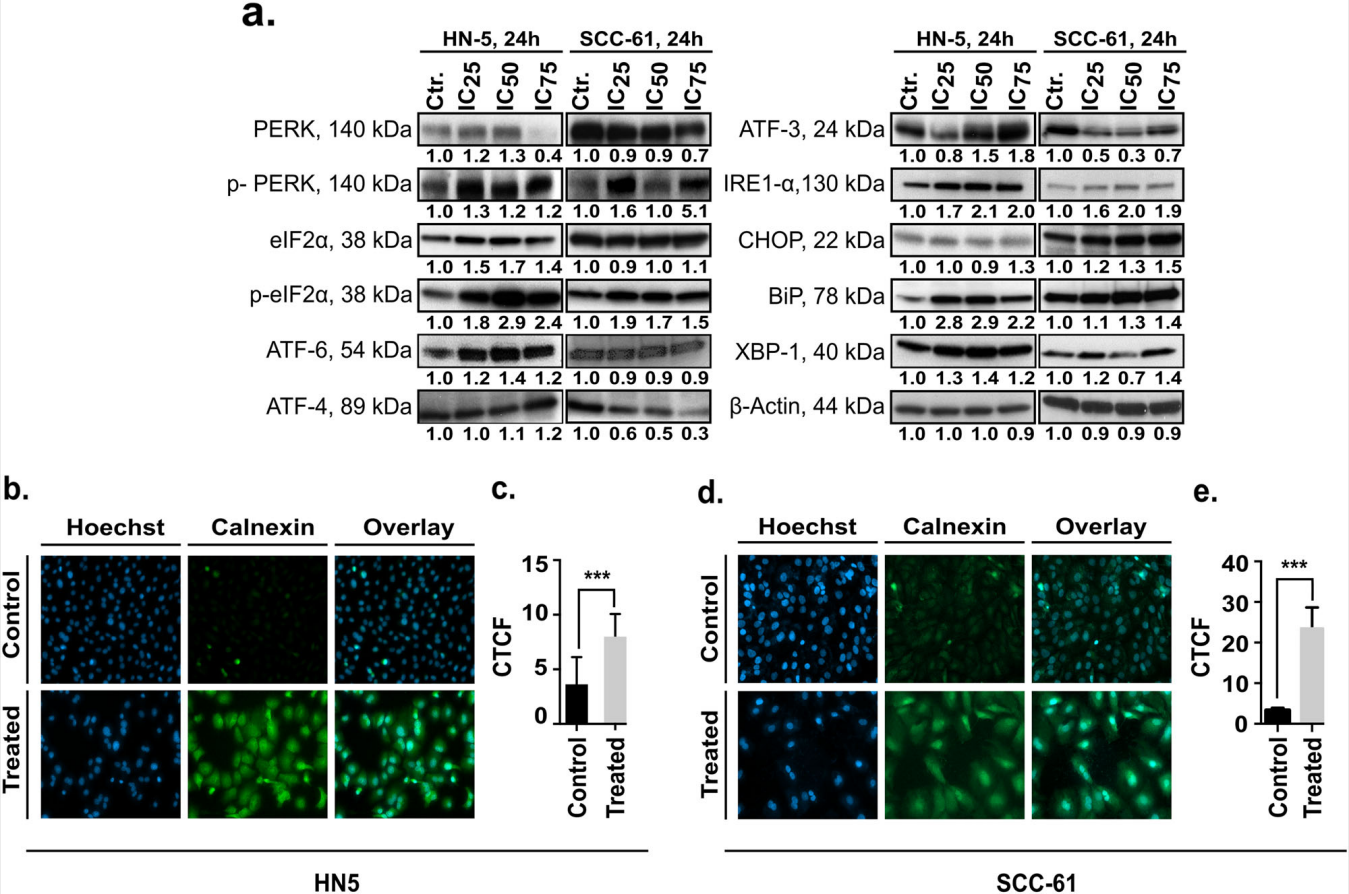
Upregulation of ER stress proteins and calnexin. **a** Expression of ER stress-related proteins in the two OSCC cell lines. β-Actin was used as loading standard. All protein bands were related to their respective control set to unity and corrected by the relative intensity of the loading control by using the densitometry function in ImageJ. **b** Immunofluorescence staining of calnexin in OSCC cell lines post 24 h of erufosine treatment. An increase in overall fluorescence intensity of calnexin in HN-5 and SCC-61 was seen (**b**, **d**) between control and treated cells. This was confirmed by CTCF (**c**, **e**), which was significantly increased in the two cell lines (*p* < 0.001). The CTCF was calculated as follows: (corrected total cell fluorescence) = integrated density − (area of selected cell × mean fluorescence of background readings). The experiment shows an average of three independent repeats.

We next analyzed the modulation of ER stress sensor proteins in the OSCC cells. Increases in the p-PERK, ATF-6, and IRE-1α levels were seen after erufosine exposure in HN-5 and SCC-61 cells (Fig. 2a). We also observed increased levels of the downstream proteins of the PERK and IRE-1α signaling viz. eIF2α, p-eIF2α, ATF4, CHOP, and XBP-1s proteins. Interestingly, a decrease in the ATF4 and ATF3 protein levels was observed in SCC-61 cells (Fig. 2a). Also, increases in the ER chaperone BiP/GPR78 were seen in HN-5 (2.9-fold) and SCC-61 cells (1.4-fold). These results show that erufosine-induced ER stress in both OSCC cell lines by upregulating one of the ER stress sensors.

As the protein calnexin is upregulated under ER stress, we analyzed its expression by immunofluorescence staining. Erufosine-exposed OSCC cells showed increased expression of calnexin (Fig. 2b, d). The “Corrected total cell fluorescence” (CTCF) in treated cells was increased 2.3-fold over control HN-5 cells (Fig. 2c) and by 11.5-fold in SCC-61 cells (Fig. 2e). These results reinforced our microarray findings of an increased enrichment of ER stress in both cell lines post erufosine exposure.

### Contribution of UPR sensors to erufosine’s antiproliferative activity

**Fig. 3:**
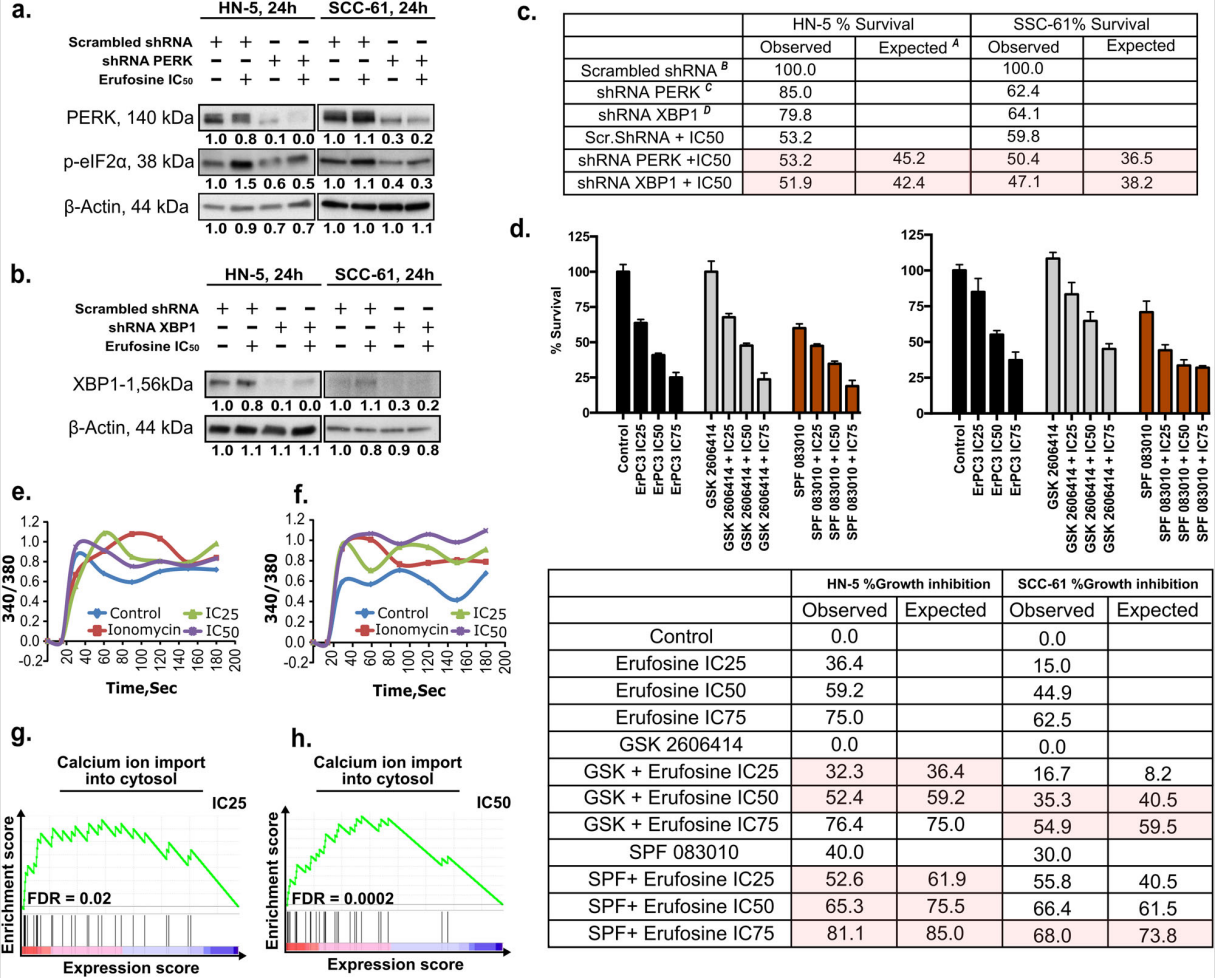
UPR sensors contribute to erufosine’s antiproliferative activity. **a** shRNA PERK knockdown in HN-5 and SCC-61 cells using lentivirus. Successful inhibition of PERK and its downstream mediator p-EIF2α are seen when compared to scrambled shRNA control. **b** Successful knockdown of XBP1 using lentivirus particle in HN-5 and SCC-61 cells. β-Actin was used as loading standard. **c** Percentage survival of scrambled, shPERK, and shXBP1 cells in response to erufosine in two cell lines. The expected survival is calculated by multiplying the individual survival percentages of each treatment arm (A = (B×C)/100 and A = (B×D)/100). **d** Percentage growth inhibition of wild-type cells in combination with erufosine and PERK inhibitor GSK-2606414 or IRE-1α inhibitor STF-083010. The expected growth inhibition is initially calculated as for survival under **c** and then by subtracting this value from 100. The highlighted columns show additive nature of combination at those doses. **e**, **f** Induction of Ca^2+^ release post erufosine treatment. Both cell lines showed an instantaneous increase in the intracellular Ca^2+^ levels as detected by Fura-2-AM dye post erufosine treatment. Ionomycin was used as positive control, and the signal was detected instantaneously post-drug exposure for 3 min. **g**, **h** Positively correlated enrichment of the term “calcium ion import in cytosol” upon erufosine exposure in HN-5 cells at IC_25_ and IC_50_ concentrations.

For analyzing how much activity of erufosine mediates through ER stress induction, HN-5 and SCC-61 cells were transduced with lentiviral particles containing shRNA^PERK^, shRNA^XBP1^, or shRNA^scrambled^. Western blots confirmed decreased levels of PERK, its downstream mediator p-eIF2α in shRNA^PERK^ (Fig. 3a) and XBP-1s in shRNA^XBP1^ (Fig. 3b) cells compared to shRNA^scrambled^.

For dissecting the contribution of ER stress to erufosine’s antiproliferative activity, erufosine at IC_50_ concentrations was administered to cells with permanently decreased PERK and XBP1. The proliferation of HN-5 cells transduced with shRNA^PERK^ and shRNA^XBP1^ was decreased by 15% and 20%, respectively, when compared to the shRNA^scrambled^ control cells. In SCC-61 cells, the survival of shRNA^PERK^ and shRNA^XBP1^ cells was 62% and 64%, respectively, in comparison to shRNA^scrambled^ control cells. When the knockdown cells were treated with erufosine, the observed cell survival was greater than that expected from an additive combination effect, thus showing resistance of the knockdown cells to erufosine and the UPR arm being required for erufosine’s full activity (Fig. 3c). Similar effects were observed for the combination of erufosine with pharmacological inhibitors of PERK (GSK-2606414) and IRE-Iα (STF-083010) in the two OSCC cell lines (Fig. 3d). GSK-2606414 at 500 nM caused no change in cell survival but when combined with erufosine, the observed growth inhibitions were lower than expected in both cell lines. On the other hand, STF-083010, the known IRE-1α inhibitor upstream of XBP-1, at 25 µM, showed 40% and 30% growth inhibition in HN-5 and SCC-61 cells, respectively. When STF-083010 and erufosine were combined, again, the observed growth inhibition was lower than expected in HN-5 cells at all concentrations, but only at the IC_75_ concentration of erufosine in SCC-61 cells. Taken together, the impairment of UPR sensors impedes the antineoplastic activity of erufosine.

### Erufosine causes release of calcium

Since ER has a critical role in controlling cellular Ca^2+^ levels, the effect of erufosine on calcium release was investigated. Ionomycin caused an increased 340/380 ratio over time, which served as positive control. Erufosine also caused an increases in the 340/380 ratio for both cell lines (Fig. 3e, f). The IC_25_ and IC_50_ levels caused peak increases in HN-5 cells followed by tapering of the signal over time. In SCC-61 cells, the 340/380 ratio was comparable to that of ionomycin with the IC_50_ concentration showing a sustained Ca^2+^ release. This hints at erufosine’s capacity to release Ca^2+^ from ER into cytoplasm under stress. In addition, our gene expression analysis indicated a significant enrichment of the gene ontology term “calcium import into cytosol” (Fig. 3g) in response to IC_25_ (FDR = 0.02) and IC_50_ (FDR = 0.0002) concentrations, hence validating our experimental findings.

### Erufosine causes induction of autophagy

**Fig. 4:**
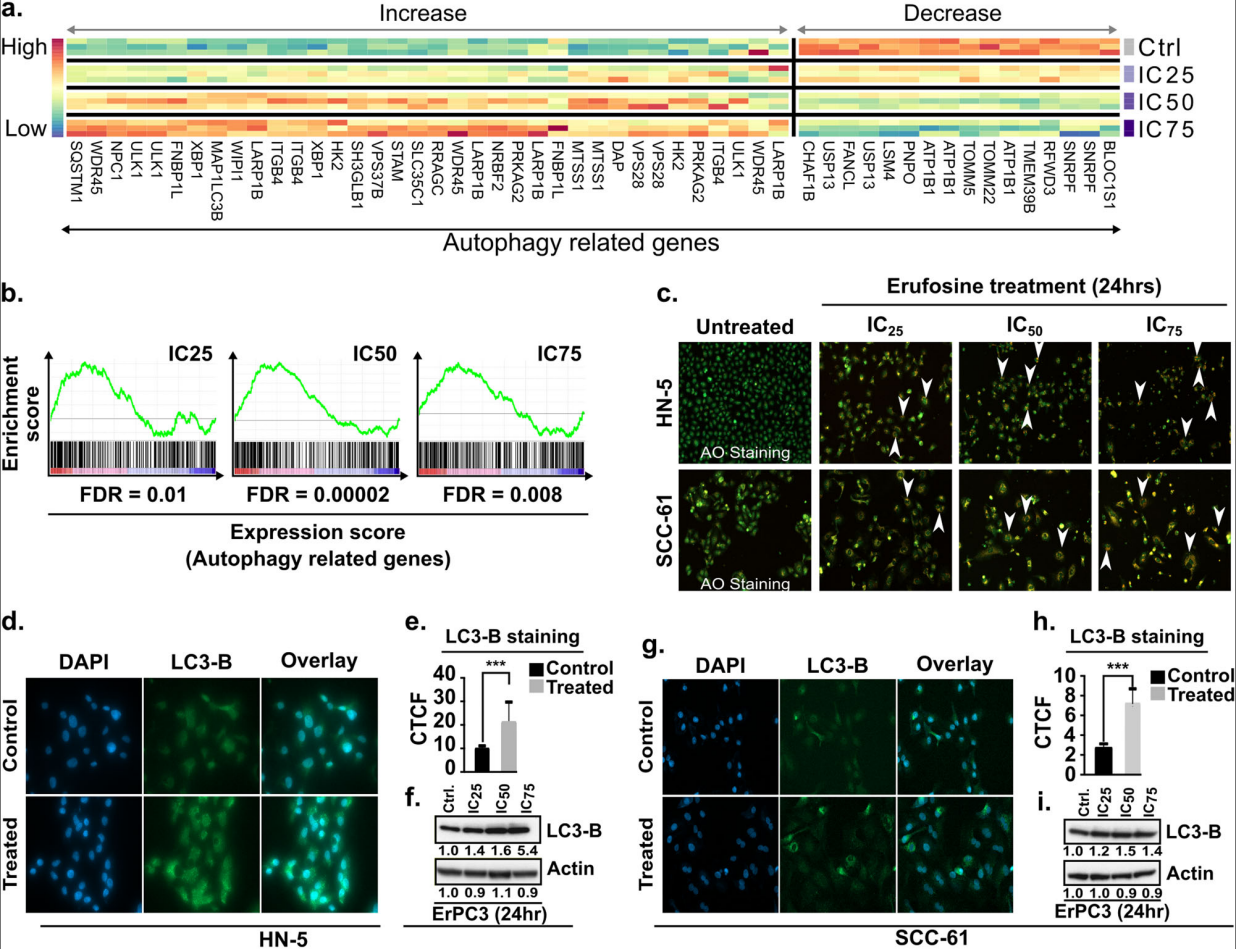
Induction of autophagy by erufosine exposure. **a** Heat map of the differentially expressed autophagy-related genes in response to erufosine at IC_25_, IC_50_, and IC_75_ concentrations in HN-5 cells. **b** The positive enrichment of autophagy-related genes upon erufosine exposure in HN-5 cells, with the FDR being a maximum of 0.01. **c** Acridine orange staining of HN-5 and SCC-61 cells exposed to increasing concentrations of erufosine shows accumulation of acidic vacuoles (AVOs) post 24 h as indicated by white arrow heads. No such AVOs can be seen in the untreated control cells. **d**, **g** Immunofluorescence staining of LC3B-II levels in HN-5 and SCC-61 cells showed increased intensity in erufosine-treated samples when compared to the control cells. A significant increase in the CTCF levels was seen (**e**, **h**) in the two cell lines, when treated with the respective IC_50_ concentration for each of the cell lines (*p* < 0.005). Western blot analysis of LC3B-II expression in HN-5 and SCC-61 cells (**f**, **i**) showed a dose-dependent increase of LC3-B-II protein levels post 24 h of exposure. β-Actin was used as loading standard. All protein bands were related to their respective control set to unity and corrected by the relative intensity of the loading control by using the densitometry function in Image J.

We hypothesized that ER stress induction in erufosine-treated cells causes autophagy, as many autophagy-related genes were differentially expressed between treated and control samples in our expression profiling assay (Fig. 4a and Supplementary Tables S5a, b). Additionally, the gene ontology term “autophagy” was significantly enriched in all treatment conditions (Fig. 4b). In order to validate this supposition, HN-5 and SCC-61 cells were stained by acridine orange for investigating the formation of acidic vesicular organelles (AVOs). Concentration dependently increased bright red to orange fluorescent vacuoles were seen in both cell lines exposed to erufosine (Fig. 4c).

This observation was further confirmed by immunofluorescence and blotting experiments. An increased LC3B-II fluorescence was seen in erufosine-treated samples when compared to control cells corresponding to accumulation of cleaved LC3B-II in both cell lines (Fig. 4d, g). The CTCF of the LC3B-II level was increased by 2.3-fold in HN-5 cells (Fig. 4e) and 3.3-fold in SCC-61 cells (Fig. 4h) when compared to untreated controls. Correspondingly, the blotting experiment also showed a concentration-dependent increase in LC3B-II protein levels in the two cell lines when compared to the untreated cells (Fig. 4f, i). We also looked into the LC3B-II levels of HN-5 and SCC-61 cells transduced with shRNA^PERK^ and shRNA^XBP1^, alone and in combination with erufosine at IC_50_ concentrations. In HN-5, LC3B-II protein levels showed no change upon shRNA^PERK^ knockdown, but were decreased by 30% in SCC-61 cells when compared to shRNA^scrambled^ control. Combination of erufosine with PERK knockdown showed LC3B-II levels, which were increased over that of untreated knockdown cells (Supp. Fig. S2a). On the other hand, shRNA^XBP1^ knockdown in HN-5 cells increased the LC3B-II level or was unchanged in SCC-61 cells when compared to scrambled controls. However, when shRNA^XBP1^ cells were exposed to erufosine, the LC3B-II levels were increased in both cell lines as compared to untreated shRNA^XBP1^ cells (Supp. Fig. S2b). These data indicate that PERK knockdown slightly decreased autophagy, but erufosine increased this process. On the other hand, knockdown of XBP1 caused increased autophagy, and exposure to erufosine further enhanced this process.

### Erufosine causes morphological changes and apoptosis in OSCC cells

**Fig. 5:**
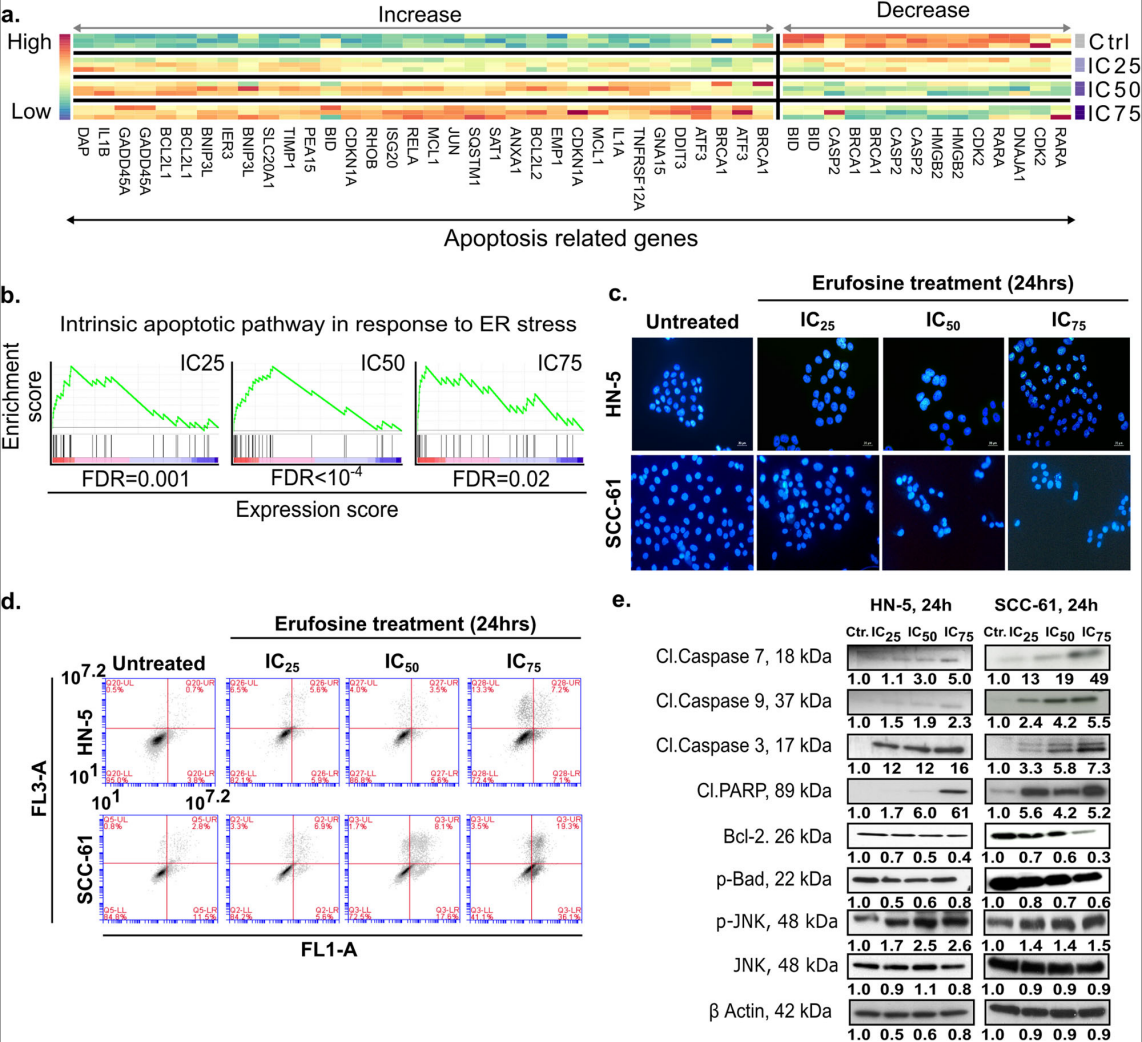
Induction of apoptosis by erufosine exposure. **a** Heat map of the differentially regulated apoptosis-related genes in response to erufosine at IC_25_, IC_50_, and IC_75_ concentrations in HN-5 cells. **b** The positive enrichment of intrinsic apoptotic pathway genes in response to ER stress upon erufosine treatment in HN-5 cells. **c** Staining of OSCC cells with Hoechst 33342 in response to erufosine exposure. Nuclear condensation and fragmentation, indicated by white arrows, were seen in the two OSCC cell lines post 24 h treatment. **d** Annexin-V-FITC staining of HN-5 and SCC-61 cells was carried out to examine the induction of apoptosis post 24 h treatment of erufosine exposure. Both OSCC cell lines showed increasing percentages of Annexin-V-bound cells as indicated by the corresponding increase in the percentage of cells in the lower right quadrant. **e** Immunoblotting of cleaved caspase and PARP in the two OSCC cell lines. Both cell lines were exposed to increasing concentration of erufosine and post 24 h an increase in the cleaved caspases 3, 7, and 9 and cleaved PARP was observed. Increased p-JNK levels were also observed in both cell lines. Further, decreased Bcl-2 and p-Bad levels were seen upon erufosine exposure. β-Actin was used as loading standard. The protein changes were derived by dividing the densitometry output for each band by that of the corresponding β-actin band.

In response to erufosine exposure for 24 h, HN-5 and SCC-61 cells showed morphological changes when compared to untreated controls. These changes included cell shrinkage, surface detachment, multi-nucleation, and blebbing (Supplementary Fig. S3). Based on these findings, we hypothesized that erufosine treatment induces apoptosis. This was corroborated by expression profiling results showing apoptosis-related genes to be differentially expressed in erufosine-treated cells (Fig. 5a and Supplementary Table S6a, b, c) and the gene ontology term “intrinsic apoptotic pathway response to ER stress” to be significantly enriched (Fig. 5b). To validate the activation of apoptosis in erufosine-treated OSCC cells, we performed Hoechst 33342 staining. Exposure to increasing concentrations, erufosine caused disruptive changes in the cells’ nuclei. Chromatin condensation was observed in treated cells with significant nuclear shrinkage and fragmentation when compared to control cells (Fig. 5c).

To further investigate the induction of apoptosis in HN-5 and SCC-61 cells, an Annexin-V-FITC-staining for inner membrane-bound phosphatidylserine (PS) was carried out. Both cell lines showed significantly increased Annexin-V-positive cells in response to rising concentrations of erufosine post 24 h exposure (Fig. 5d).

Next, the induction of apoptosis was confirmed by immunoblotting of proteins responsible for apoptosis (Fig. 5e). Induction of apoptosis was evident from increased levels of cleaved caspases 3, 7, and 9 in a dose-dependent manner. The highest increases in expression for caspases 3, 7, and 9 were 16-, 5.0-, and 2.3-fold in HN-5 cells and 7.0-, 49-, and 5.5-fold in SCC-61 cells. Increases in the cleaved fragment of PARP were also observed in a dose-dependent manner reaching a maximum induction of 6.1-fold in HN-5 cells and 5.2-fold in SCC-61 cells. Also, increased levels of p-JNK were observed in both cell lines, with 2.6-fold (HN-5 cells) and 1.5-fold (SCC-61 cells) surges. Activation of p-JNK leads to activation of apoptosis in cells and is also under the control of the ER stress sensor IRE-1α, hence suggesting the apoptosis, in part, is caused by ER stress induced via erufosine. Furthermore, decreased levels of the pro-survival proteins, p-Bad and Bcl-2 were observed. After exposure to erufosine for 24 h, Bcl-2 and p-Bad levels decreased to 40% and 80% were seen in HN-5 cells and to 30% and 60% in SCC-61 cells. These findings suggest that erufosine causes a stable increase in apoptosis induction and decrease in pro-survival proteins (Fig. 5e).

The effect of apoptosis in PERK and XBP1 knockdown cells was observed in HN-5 and SCC-61, either alone or in combination with erufosine, by investigating the cleaved PARP protein levels. In HN-5 and SCC-61 PERK knockdown cells, the cleaved PARP levels were increased 1.8- and 2.9-fold, respectively, when compared to scrambled controls. Following exposure to erufosine, the cleaved PARP levels further increased in knockdown cells to 2.2- and 5.8-fold with respect to HN-5 and SCC-61 scrambled control cells (Supp. Fig. S4a). However, the PARP level was lower than expected for an additive combination effect (Supp. Fig. S4a). On the other hand, XBP1 knockdown in HN-5 cells increased the cleaved PARP level by 2.7-fold and the additional exposure to erufosine was again less than additive by showing a 4.0-fold increase over scrambled control as opposed to a 4.6-fold increase following erufosine alone (Supp. Fig. S4b). In contrast, the cleaved PARP level was decreased by 70% in XBP1 knockdown SCC-61 cells as compared to scrambled control, and the exposure of these cells to erufosine caused a relative increase in their PARP level, which however, did not reach that of scrambled controls. This observation hints to a similar mechanism as for PERK, indicating that erufosine’s apoptotic activity depends upon the levels of the ER sensors PERK and XBP1.

### Erufosine exposure leads to loss of mitochondrial membrane potential and decreased ATP production

**Fig. 6:**
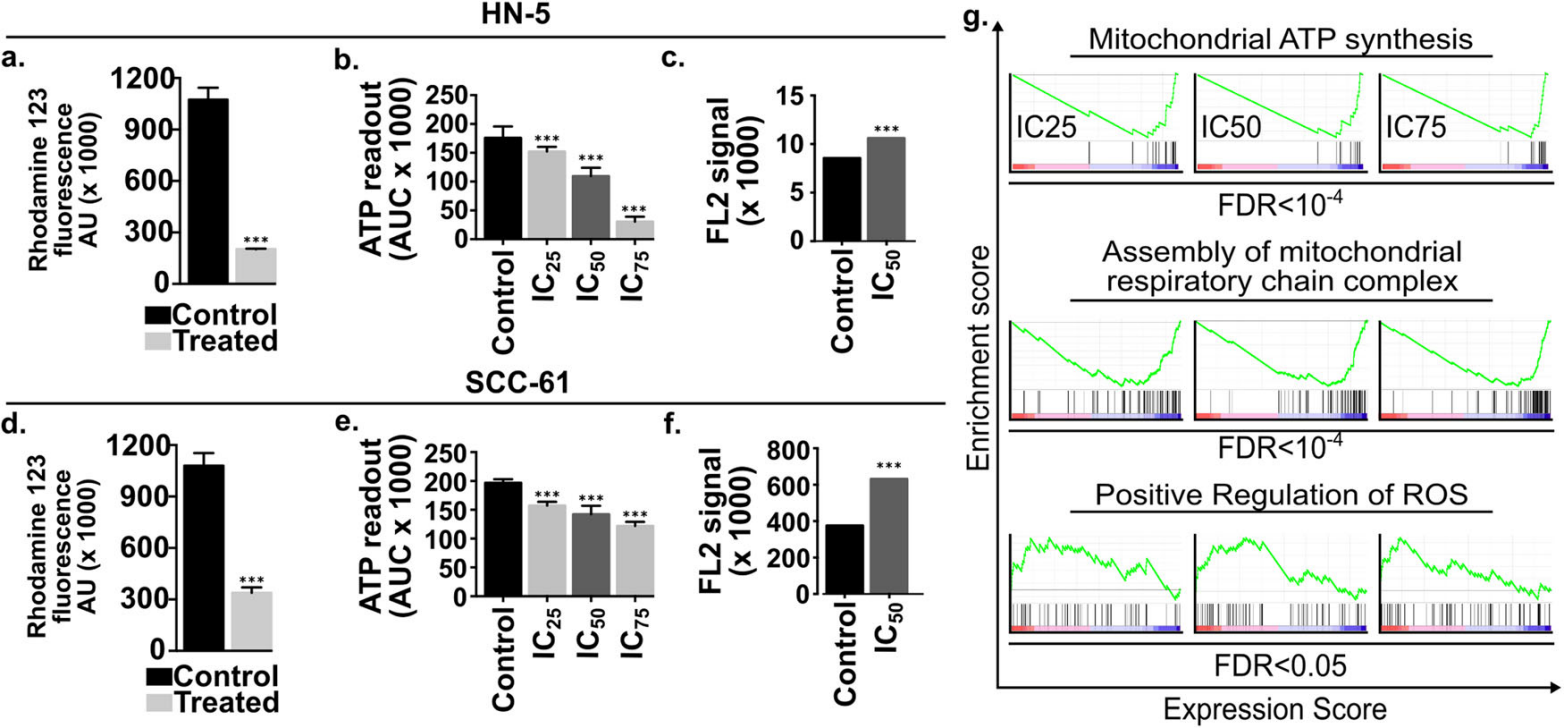
Dysregulation of mitochondria upon erufosine exposure. **a** Measurement of mitochondrial membrane potential (Δψm) with rhodamine in OSCC cells. The IC_50_ concentration of erufosine was used for both cell lines and fluorescence intensity was measured post 24 h. A significant decrease in the fluorescence intensity was seen in response to exposure (*p* < 0.001). **b** Measurement of ATP production in OSCC cell lines. The luminescence assay was used to measure the ATP levels post 24 h of erufosine treatment in HN-5 and SCC-6 cells. A significant decrease (**p < 0.05) (***p < 0.005) was seen in the two cell lines. **c** ROS measurement in HN-5 and SCC-61 cells post 24 h of erufosine treatment. An increase in the AUC of the DCF-DA fluorescence signal peak was seen in erufosine-treated cells when compared to the control cells. **g** Negative enrichment of the mitochondrial ATP synthesis and assembly of mitochondrial respiratory chain complex was seen at IC_25_, IC_50_, and IC_75_ concentration of erufosine, whereas a positive regulation of ROS was observed upon erufosine exposure at the three concentrations of erufosine in HN-5 cells.

The effect of erufosine on mitochondrial transmembrane potential (Δψm) was evaluated using rhodamin-123. Erufosine led to significant (70–80%) loss of Δψm compared to untreated control cells post 24 h of treatment (Fig. 6a, d). This loss of fluorescence intensity indicates the incapability of mitochondria to retain the dye and hints at mitochondrial depolarization due to erufosine exposure.

Then, the cellular ATP levels were measured in response to varying concentrations of erufosine. Dose dependently decreased ATP levels were observed in the two cell lines post 24 h of treatment (Fig. 6b, e). Furthermore, gene ontology terms like “Mitochondiral ATP synthesis” and “Assembly of mitochondrial respiratory chain complex” were strongly under-enriched in erufosine-treated HN-5 cells at all concentrations (Fig. 6g). These results show that erufosine causes mitochondrial depolarization in OSCC cells leading to decreased ATP production.

We also looked into the changes in Δψm occurring following PERK and XBP1 knockdown in HN-5 and SCC-61 cells, alone or in combination with erufosine. The Δψm of shRNA^PERK^ cells was higher than that of the scrambled control cells in both cell lines. However, the Δψm in shRNA^XBP1^ cells was higher than in control untreated HN-5 cells, but showed a decreased membrane potential in SCC-61 cells when compared to control. Nevertheless, exposure to erufosine in PERK and XBP1 knockdown cells decreased the membrane potential (Supp. Fig. S5a, b).

### Erufosine exposure leads to ROS generation

In order to investigate if exposure to erufosine leads to formation of ROS, a DCFH-DA-based flow cytometric analysis was performed. A strong increase in intracellular ROS levels was detected in both OSCC cell lines in response to erufosine exposure (Fig. 6c, f). Compared to the control group, the erufosine-exposed samples showed an increased area under the curve of the FL-2 peak. In addition, we observed a significant enrichment of the gene ontology term “positive regulation of ROS” upon erufosine treatment in our expression profiling assay (Fig. 6g). This shows that erufosine is capable of inducing ROS in OSCC cell lines, which may be due to the loss of mitochondrial membrane potential.

## Discussion

The ER is responsible for protein folding, translocation, and post-translation modification, which requires factors including adequate ATP and Ca^2+^ levels as well as an oxidizing environment to allow disulfide-bond formation^29^. ER stress can be induced by nutrient deprivation, altered glycosylation, calcium depletion, oxidative stress, or DNA damage and energy disturbance/fluctuation. These perturbations cause accumulation of unfolded or misfolded proteins in the ER, which responds by activating a signal transduction pathway, termed the unfolded protein response (UPR)^30^. However, when the stress is chronic or too severe, ER stress engages apoptosis^24,31^.

Erufosine caused toxicity in HN-5 and SCC-61 cells and upon microarray and pathway enrichment analysis, the GSEA category “Response to ER stress and IRE-1 mediated unfolded protein response” was positively and concentration dependently enriched. Moreover, UPR and apoptosis were also positively enriched thus indicating that these processes contribute toward the antineoplastic activity of erufosine.

The PERK, ATF-6, and IRE-1α pathways were validated in HN-5 and SCC-61 cells, and increased expression of the active form of PERK and its downstream mediator’s eIF2-α/ATF4 and CHOP were seen at protein level. Also, increased protein levels of ATF-6 and IRE-1α were observed in HN-5 cells and although SCC-61 cells showed no change in ATF-6 levels, an increased IRE-1α level was seen.

The PERK and XBP1 knockdown cells showed increased cell survival upon erufosine exposure compared to what was expected from an additive combination effect. A similar profile was observed when pharmacological inhibition of PERK and IRE-1α was combined with erufosine as the growth inhibition was lower than expected. These results show a connection between upregulation of ER stress sensors and erufosine exposure, as deficiency of the ER stress sensors contributed toward resistance to erufosine, and that a part of erufosine’s antineoplastic activity mediates through these sensors.

The ER sensor BiP/GPR78 targets misfolded proteins, regulates calcium homeostasis in cells as a response to ER stress, and has pro-apoptotic function^32,33^. We observed increased levels of BiP/GPR78 upon erufosine exposure, which may be due to the cells’ effort to counter the increased levels of unfolded proteins. This shows that erufosine induces UPR and attenuates protein translation by activating the PERK/eIF-2α/ATF4/CHOP, ATF-6, or IRE-1α in HN-5 cells and via the PERK and IRE-1α signaling axis in SCC-61 cells. Activated CHOP expression inhibits anti-apoptotic Bcl-2 protein^34^ and induces apoptotic cell death by activating Bim^35^. Also, triggering of IRE-1α led to activation of p-JNK in our cells, which is known to transmit the IRE-1α-induced apoptosis in cells^24^. ER stress in the OSCC cells was also confirmed by immunofluorescent staining of calnexin protein, which is a marker for ER-induced apoptosis^36^. Erufosine further caused increased cytosolic Ca^2+^ levels, and this increase may play a role in erufosine-mediated cell death. Similarly, GSEA indicated upregulation of calcium ion import into the cytoplasm. The alkyl-lysophospholipid analog edelfosine has also been shown to increase cytosolic Ca^2+^ levels in pancreatic cancer and induce apoptosis^37^.

Next, autophagy was highly enriched upon erufosine treatment and this was corroborated by increased acidic vacuole formation. Also, increases in total cell fluorescence and protein levels of LC3B-II were seen upon erufosine treatment. It is known that cells undergoing ER stress have activated macro-autophagy and membrane particles of the auto-phagosomal bodies originate from enlarged ER membranes^38^. The process of macro-autophagy can also be attributed to the activation of the PERK/eIF2-α pathway^39^. Erufosine is known to cause decreased levels of phosphatidylcholine (unpublished data), and the lack of this phospholipid may result in the disrupted ER–Golgi trafficking network leading to ER stress^40^.

Knockdown of PERK in OSCC cells decreased their LC3B-II levels, indicating a decreased autophagy process, as shown in osteosarcoma cells^41^. The exposure of shRNA^PERK^ knockdown cells to erufosine restored the LC3B-II levels, by upregulating the autophagic arm in these cells. On the other hand, shRNA^XBP-1^ knockdown increased the LC3B-II levels in HN-5 cells, but decreased them in SCC-61 cells when compared to controls. Although it has been reported that XBP-1 knockdown decreases autophagy in auditory cells^42^, similar to SCC-61 cells, this cannot be generalized, as derived from the observed increase in HN-5 cells. Similar to the effect in PERK knockdown cells, there was an increase in the LC3B-II protein levels when the XBP1 knockdown cells were treated with erufosine. Induction of autophagy following knockdown of ER stress sensors might be a protective mechanism against the cytotoxicity of erufosine.

We also analyzed the autophagy protein-5 (Atg-5) at mRNA and protein levels following erufosine exposure. Phosphorylated PERK activates ATF4, which regulates Atg-5 expression by binding directly to its promoter. Although an increase was seen at mRNA level of Atg-5 in both cell lines (Supp. Fig. S6a), this was not paralleled at the protein levels, where a slight decrease was observed in HN-5 cells and no change in SCC-61 cells (Supp. Fig. S6b). This indicates that erufosine induces macro-autophagy in OSCC cells via an Atg-5-independent mechanism^43^, which would be interesting to decipher in future studies.

Induction of apoptosis was confirmed in OSCC cells, which validates the positive gene enrichment that was seen in the GSEA analysis. Nuclear fragmentation post erufosine was confirmed by staining and induction of apoptosis was confirmed at protein level by increases in cleaved caspases and cleaved PARP intensities, and decreases in Bcl-2 and p-Bad levels. The induction of apoptosis could be attributed to ER stress, through upregulation of CHOP by PERK receptor and p-JNK activation via the IRE-1α receptor, that inhibits the Bcl-2 family, causing cell death^34^, or through the influx of Ca^2+^ into the cytoplasm, which is released from the ER lumen and transported to the mitochondria^44,45^.

Apoptosis induction was two–threefold increased in shRNA^PERK^ knockdown cells as compared to scrambled controls, as has been described in osteosarcoma cells^41^. Fragmented PARP levels increased further when shRNA^PERK^ cells were exposed to erufosine. However, this increase was less than additive, indicating that PERK is required to generate erufosine’s full apoptotic effect. A similar effect was observed in shRNA^XBP1^ HN-5 cells, showing increased levels of PARP, but a contrasting effect was observed in shRNA^XBP1^ SCC-61 cells, which showed cleaved PARP levels below that of scrambled controls. Again, the presence of XBP-1 proved to be essential for the apoptotic activity of erufosine.

Mitochondrial stress was also observed in erufosine-exposed OSCC cells as seen by decreased ATP levels, loss of mitochondrial membrane potential, and increased ROS production. Drugs, which induce apoptosis in cancer cells, are known to be associated with a rapid collapse of mitochondrial membrane potential^46^. Interestingly, we observed an unexpected modulation of Δψm in shRNA^PERK^ HN-5 and SCC-61 cells as well as in shRNA^XBP1^ HN-5 cells. This warrants further investigation; however, in any case, erufosine further decreased the mitochondrial membrane potential in knockdown cells.

Loss of membrane potential is known to precede DNA fragmentation, ROS production, and increased membrane permeability, which causes activation of caspases. The decrease in ATP levels and the loss of membrane potential can be explained by inhibition of the F_0_ subunit of the mitochondrial F_0_F_1_ ATP synthase and was shown by the same class of compounds in the glioblastoma U87MG and U118MG cell lines^47^. Also, the excess Ca^2+^ escaped from the ER might increase the production of ROS from mitochondria and lead to alteration of the mitochondrial permeability transition pore, resulting in cell death^48^.

**Fig. 7:**
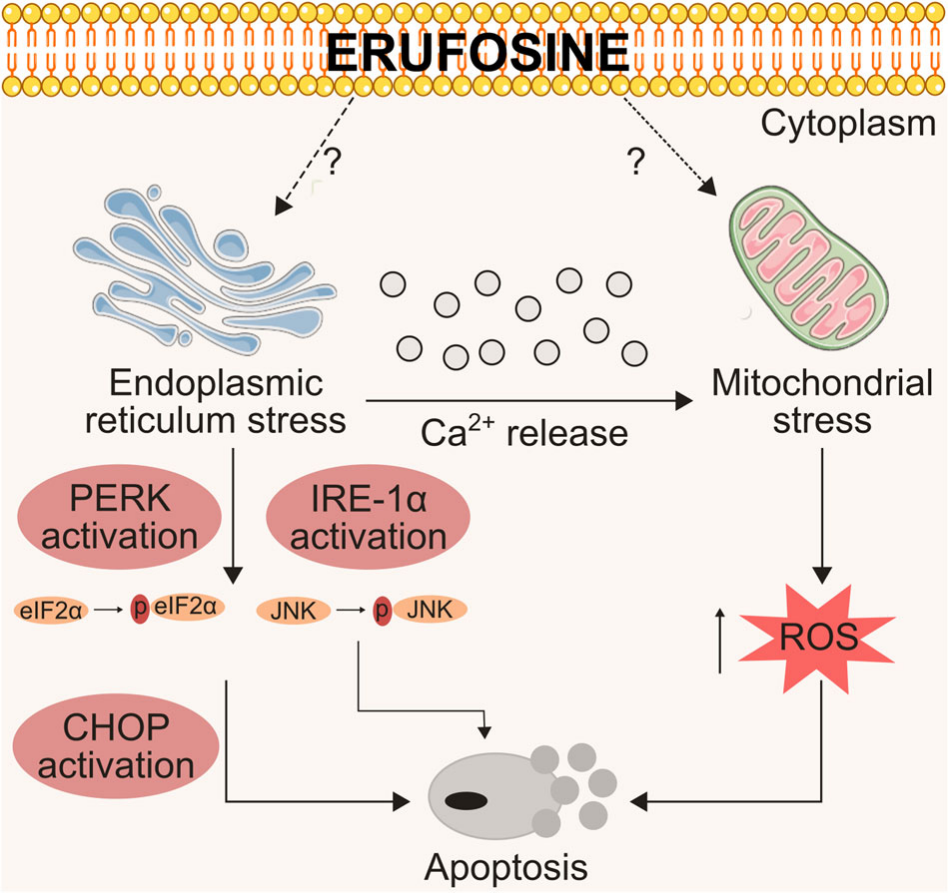
Graphical summary of erufosine’s effect on endoplasmic reticulum (ER) and mitochondria. Erufosine induces ER stress, which leads to activation of the ER stress sensors. One of the pathways activated is the PERK signaling, which causes phosphorylation of eIF2α and activation of CHOP via ATF4. Subsequently, apoptosis is induced. Also, activation of IRE-1α leads to activation of the JNK pathway. Likewise, erufosine causes release of Ca^2+^ into the cytoplasm, which acts on the mitochondria and may cause their depolarization, which is manifested by a loss of mitochondrial membrane potential and loss of ATP production. This eventually leads to ROS production, creating a pro-apoptotic environment within the cells. Also, erufosine causes activation of the intrinsic apoptotic pathway. Dotted lines indicate an indirect effect of erufosine on ER and mitochondrial responses. The question marks indicate unexplored mechanisms by which erufosine effects ER and mitochondrial membranes.

In summary, ER and mitochondrial targeting by erufosine represent a new facet of erufosine’s mechanism of action (Fig. 7).

## Materials and methods

### Cell culture and reagents

Human oral squamous cell carcinoma cell lines, HN-5 and SCC-61, were obtained as a kind gift from Prof. Myers’ lab, MD Anderson Cancer Center, USA. HN-5 cells were cultured in DMEM: F-12 medium (Lonza, Germany) and SCC-61 cells were maintained in Dulbecco’s modified Eagle’s medium (DMEM) (Invitrogen, USA) supplemented with 10% and 20% fetal bovine serum (FBS) respectively. Cell lines were maintained in a 37 °C, 5% CO_2_ humidified incubator. The cell lines had been authenticated by MD Anderson center using short tandem repeat analysis and were routinely tested for mycoplasma contamination.

Erufosine (erucylphospho-*N*,*N*,*N*-trimethylpropanolamine, ErPC3) was kindly provided by Prof. H. Eibl, Max Planck Institute of Biophysical Chemistry, Gottingen, Germany. It was dissolved in saline at a concentration of 20 mM and stored at 4 °C. The PERK inhibitor GSK-2606414 (Merck) and IRE-1α inhibitor STF-083010 (Biomol) were dissolved in DMSO to a stock concentration of 2 mM and 3 mM respectively, and stored in the dark at −20 °C. Compound stock solution was thawed at room temperature prior to dilution into aqueous media at appropriate concentrations for use in biological assays.

### MTT assay

The cytotoxic effect in response to erufosine treatment in HN-5 and SCC-61 oral squamous cancer cells was analyzed using the MTT [3-(4,5-dimethylthiazol-2-yl)-2,5-diphenyltetrazolium bromide] dye reduction assay as previously described^20^.

### Gene enrichment analysis

To analyze the global gene modulation taking place in response to erufosine treatment on HN-5 cells, gene expression profiling was performed by microarray. Briefly, HN-5 cells were treated with concentrations corresponding to IC_25_, IC_50_, and IC_75_ (27, 39, and 55 μM) of erufosine for 16, 24, and 48 h. mRNA was then extracted using the RNeasy Mini kit (Qiagen, Germany) following the manufacturer’s protocol and the extracted mRNA was subjected to gene expression analysis using Illumina Chip array.

The lumi^49^ and limma packages^50^ from Bioconductor were used to preprocess the raw expression data and perform differential gene expression analysis, respectively. Using lumi, we performed background correction, variance stabilization and robust spline normalization of the raw expression data. Then, differential gene expression analysis on the normalized data was achieved between erufosine-treated and untreated cells. After the differential gene analysis, we computed the differential gene expression score for each gene measured in the expression profiling assay by multiplying its log fold change (treated vs. control) with the negative log of its false discovery rate. We used this scoring scheme to rank all the ~20,000 genes such that a gene with the highest score would be the most differentially over-expressed gene in a treated vs. control comparison while the one with the lowest score would be the most under-expressed. This ranked list of genes was used to perform gene set enrichment analysis (GSEA; version 2-2.2.3)^51,52^ to identify significantly (FDR <0.05) up- and downregulated pathways. We used the gene set collections (H: Hallmarks and C5: GO biological processes) available at the Molecular Signatures Database v6.0 (MsigDB) (http://software.broadinstitute.org/gsea/msigdb/index.jsp) for our pathway enrichment analysis (Supplementary Materials 2 and 4) and for selecting genes involved in apoptosis, autophagy, and response to endoplasmic reticulum stress. All the above statistical analysis was carried out in the R language (https://cran.r-project.org/). The raw data for the gene expression analysis has been submitted to the Gene Expression Omnibus database (GEO ID: GSE96599).

### Quantitative real-time PCR (qRT-PCR) analysis

Total RNA was isolated from both cell lines post 24 h of treatment as described before. cDNA was synthesized using Mu-MLV reverse transcriptase (Thermo Scientific) from equal amounts of RNA (1 μg). qRT-PCR was carried out to investigate the microarray findings for UPR genes, XBP1s target genes and ATG5 using 2× LC480 master mix along with an appropriate probe from the Universal probe library (Roche). Experiments were performed in triplicates and the expression level of glyceraldehyde 3-phosphate dehydrogenase (GAPDH) was used for normalization. The fold changes were calculated by the 2^−∆∆CT^ method (Supplementary Material 7, Table S7).

### Immunoblotting

Immunoblotting was carried out to analyze changes in protein level in response to drug exposure. Cells from both cell lines were seeded at a density of 1 × 10^6^ in 75-cm^2^ culture flasks followed by drug exposure for 24 h at concentrations corresponding to IC_25_, IC_50_, and IC_75_ values. Cells were collected and lysed with RIPA buffer supplemented with complete protease inhibitor cocktail tablets. Protein concentrations were determined and protein lysates (30–50 µg) were mixed with (4×) LDS NuPAGE sample buffer and heated for 5 min at 99 °C and subjected to electrophoresis on 4–12% polyacrylamide gradient SDS gels (Serva, Heidelberg). Proteins were then transferred onto PVDF membranes and blotted for PERK, ATF-6, ATF4, ATF3, eIF2a, p-eIF2a, IRE-1α, Bip1, cleaved caspases 3, 7, 9, LC3-B, p-Bad (Cell Signaling), Bcl-2, p-PERK, CHOP, JNK, p-JNK, Bax, XBP-1, and β-actin (Santa Cruz). Immunoblots were developed using HRP-conjugated anti-rabbit IgG (Cell Signaling), anti-goat, anti-mouse, or anti-rabbit IgG (Santa Cruz, USA) and ECL-System (Amersham Pharmacia Biotech, Germany). Levels of β-actin were used to normalize the protein expression. Relative concentrations were assessed by densitometry analysis of digitized autographic images using the ImageJ software.

### shRNA

Lentiviral particles were produced using HEK293 cells according to The RNAi Consortium (TRC) recommendations. The shRNA^PERK^ oligo and the shRNA^XBP1^ oligo were cloned into the pLKO.1 plasmid (for sequences see Supplementary Material, Table S8). HEK293 cells (60–70% confluent) were transfected with pLKO.1 carrying the gene of interest using PEI (polyethyleneimine). For transfection, 8 µg of the construct carrying the gene of interest was diluted in Opti-MEM containing 4 µg of each of the supporting plasmids (pMDG.2 and pSPAX), thus making up a total volume of 250 µl. About 48 µg of PEI (1 mg/ml in water) was diluted in Opti-MEM separately in a total volume of 250 µl. Both the reaction mixtures were vortexed, and later mixed together (transfection mix) and vortexed again thoroughly. The transfection mix thus obtained was allowed to stand at room temperature for 20 min. Later, the 500 µl of the reaction mix was added drop-wise onto the HEK293 cells covering the whole surface. To harvest virus particles after 48 h of transfection, the supernatant from the transfected HEK293 cells was collected in a falcon tube and centrifuged at 1500 rpm for 5 min to remove floating cells. The collected supernatant was purified using a 0.45 µm filter into a new falcon tube to separate cell debris. This virus supernatant was then ultra-centrifuged using a SW41 swing-out rotor in a L8-M ultracentrifuge at 25,000 rpm for 90 min at 4 °C. The supernatant was aspirated and the virus pellet thus obtained was re-suspended in 100 µl of Opti-MEM solution. The virus dilution was aliquoted and frozen at −80 °C.

An aliquot of 10 µl of this virus was added to cells HN-5 and SCC-61 cells in a 5-well plate with 50% confluency. The cells were incubated with the virus for 48 h. Thereafter, media were changed, fresh media with 0.25 mg/ml of puromycin was added for selection. The selection was carried out for another 48 h before switching to normal media. The knockdown was confirmed by analyzing the respective proteins by western blots.

### Immunofluorescence staining

Immunofluorescence (IF) staining was performed to evaluate intracellular expression levels of LC3B-II and calnexin. Briefly, 1.25 × 10^5^ cells were grown as monolayers on a glass coverslip in 6-well plates. The following day, cells were treated with erufosine for 24 h. Post treatment, cells were washed with PBS, fixed in 4% paraformaldehyde for 15 min followed by another washing with PBS. Cells were permeabilized with 0.5% Triton X-100, blocked with 1% BSA and incubated with primary antibodies (1:100 dilutions in PBS) for 30 min. Subsequently, cells were washed and incubated in the fluorescently labeled secondary antibody for 30 min in dark at concentrations recommended by the manufacturer. Coverslips were then washed, counter stained with Hoechst dye, mounted on slides, and the fluorescence signal was analyzed using an Axio observer ZI microscope. The corrected total cell fluorescence (CTCF) was calculated as CTCF = integrated density − (area of selected cell × mean fluorescence of background readings) using the imageJ software.

### Fluorescent Ca^2+^ release

Changes in [Ca^2+^] levels were measured using the Ca^2+^ sensitive fluorescent ratio dye Fura-2AM (Molecular Probes; Invitrogen). Briefly, 4 × 10^3^ cells were seeded in a 96-well plate and incubated for 24 h. The following day, cells were loaded with 5 μM Fura-2AM in Hank’s buffered salt solution (HBSS) for 40 min at 37 °C in the dark. This was followed by washing with HBSS once and incubation for 20 min for de-esterification in PBS. Next, HBSS, ionomycin, IC_25_ or IC_50_ concentrations of erufosine were added and the plate was read using an Infinite M200 microplate reader (Tecan Trading, Switzerland). Fluorescence was evoked by 340- and 380-nm excitation wavelengths (F340 and F380, Fura-2) and collected at 510 nm. Data were collected every 15 s in the plate reader for 4 min. Data changes in 340/F380 ratio were calculated over time.

### Acridine orange (AO) staining

Cytoplasmic acidification was assessed by the AO staining procedure of the autophagic vacuoles. Briefly, 1.25 × 10^5^ cells per well were grown in a 6-well plate. Cells were then treated for 24 h with the drug. After the end of the treatment period, cells were incubated for 15 min at 37 °C in serum-free medium containing 1 μg/ml 3,6-bis(dimethylamine) acridine orange. Cells were then observed under the Axio Observer Z1 microscope and images were captured using the excitation filter (488 nm) and emission filters (505–530 nm and >650 nm).

### Hoechst 33342 staining

To analyze the nuclear staining post erufosine exposure, Hoechst 33342 dye was used. Briefly, 1.25 × 10^5^ cells/well were seeded in a 6-well plate on sterilized coverslips (sterilized with 70% ethanol) and allowed to attach and grow under standard incubation conditions. The following day, drug treatment was carried out for 24 h following which, cells were washed with PBS and fixed by 4% formaldehyde for 10 min. Cells were then permeabilized with 0.3% Triton X-100 in 1 ml PBS for 10 min and stained with 1.6 mM Hoechst 33342 solution for 10 min in the dark. The coverslips were mounted on glass slides using mounting solution and photographed with fluorescence Zeiss Axiophot microscope (350 nm excitation wavelength).

### Annexin-V assay

The Annexin-V-FITC assay kit (Affymetrix, eBioscience, (88-8007-74)) was used according to the manufacturer’s recommendation to analyze the apoptotic fraction post erufosine exposure. Briefly, 3.5 × 10^5^ cells were seeded in 25-cm^2^ cell culture flasks, followed by drug treatment for 24 h. Cells were then collected with EDTA-free trypsin and washed with PBS. The second washing step was carried out with 1× binding buffer (provided with kit). Total of 2 × 10^5^ of these washed cells were re-suspended in 100 µl of the 1× binding buffer and 5 µl of Annexin-V-FITC dye per sample was added. Following the incubation period for 15 min in dark at room temperature, the cells were washed again with 1× binding buffer in order to remove unbound Annexin-V-FITC. The cell pellets were re-suspended in 200 µl of the 1× binding buffer and 5 µl of propidium iodide/sample (provided with the kit) was added before flow cytometry analyses were performed using a BD Accuri C6.

### ROS measurement

Intracellular ROS generation was measured using 2′,7′-dichlorofluorescein diacetate (DCFH2-DA). Briefly, 3 × 10^5^ cells were seeded in a 25 cm^2^ flask and exposed to drug treatment for 24 h. DCFH2-DA was diluted in serum-free media to yield a 10 μM working solution. Cells were washed twice with PBS and then incubated with DCFH2-DA for half an hour in a dark environment (37 °C incubator). The cells were then washed with PBS, trypsinized and re-suspended in 1 ml of PBS. Negative control, positive control, and treated samples were subjected to flow cytometry for ROS detection using the 488 nm laser for excitation and detected at 535 nm using a BD Accuri C6.

### Mitochondrial membrane potential (ΔΨm)

The mitochondrial membrane potential (ΔΨm) was measured in control and erufosine-treated cells using rhodamine 123 dye. Briefly, 5 × 10^5^ cells were seeded in a 25-cm^2^ flask and treated with drug for 24 h. After the end of drug treatment, 5 µg/ml of rhodamine 123 was added to the flask and incubated for 30 min in the dark at 37 °C. The cells were then trypsinized, centrifuged, and the pellet was washed in PBS and finally re-suspended in 1 ml of PBS. Cells were immediately analyzed by flow cytometry using a BD C6 Accuri.

### Measurement of ATP levels

Total ATP was quantified between untreated and treated OSCC cells using a commercially available luciferin–luciferase assay kit (Promega). The cells were seeded in white sterile cell culture compatible 96-well plates and treated with varying concentration of drug for 24 h. Following the end of treatment, cell lysis, inhibition of endogenous ATPases, and detection of ATP were performed by adding the CellTiter-Glo^®^ Reagent to the culture wells in equal volumes. Lysing of the cells was ensured by incubating the plate for 10 min at RT and moderate shaking. The bioluminescence was then measured in a luminometer (Berthold Technologies, Germany).

### Statistical analysis

Student’s *t* test was used to determine statistical significance of differences between groups using the GraphPad Prism for all other experiments. ImageJ software was used for densitometry analysis of the western blots and for evaluating the corrected total cell fluorescence. The BD Accuri C6 software was used to evaluate the Annexin-V stainings. All the data were expressed as mean ± SD, with *p* values <0.05 considered as statistically significant. The combination effect on cell proliferation resulting from exposure to erufosine and the inhibitors GSK/STF, or the combination of gene knockdown with exposure to erufosine was determined by MTT assay. Expected (additive) combination effects were calculated from the individual treatments by multiplying the respective ratios in percent of control. Results showing a survival fraction that deviated by more than 30% from the expected combination effect were considered significantly synergistic or antagonistic, depending on the direction of deviation^53^.

## Electronic supplementary material

Supplementary Table 1a

Supplementary Table 1b

Supplementary Table 1c

Supplementary Table 2a

Supplementary Table 2b

Supplementary Table 2c

Supplementary Table 3a

Supplementary Table 3b

Supplementary Table 3c

Supplementary Table 4a

Supplementary Table 4b

Supplementary Table 4c

Supplementary Table 5a

Supplementary Table 5b

Supplementary Table 6a

Supplementary Table 6b

Supplementary Table 6c

Supplementary Table 7

Supplementary Table 8

Suppl. Figures S1-S3

Suppl. Figures S4-S6

Supplementary Figure Legends
