# Supplementary material for: Induction of ER and mitochondrial stress by the alkylphosphocholine erufosine in oral squamous cell carcinoma cells
Source: Cell Death Dis. 2018 Feb 20;9(3):296. doi: 10.1038/s41419-018-0342-2 (PMC5833417; doi:10.1038/s41419-018-0342-2)
Supplement: Supplementary file 1 — Supplementary Table 1a [file 41419_2018_342_MOESM1_ESM.docx]

**Table S1a: Differential regulation of genes upon IC25 exposure of erufosine in HN-5 cells**

| **Symbol** | **Definition** | **Log Fold Change** | **Average Expression** | **t-statistics** | **P.Value** | **adj.P.Val** |
| --- | --- | --- | --- | --- | --- | --- |
| ANGPTL4 | Homo sapiens angiopoietin-like 4 (ANGPTL4), transcript variant 1, mRNA. | 3,0034 | 9,836 | 7,28275 | 3,391E-05 | 0,02263 |
| HBEGF | Homo sapiens heparin-binding EGF-like growth factor (HBEGF), mRNA. | 2,8168 | 10,497 | 6,48970 | 8,609E-05 | 0,03387 |
| TM4SF19 | Homo sapiens transmembrane 4 L six family member 19 (TM4SF19), mRNA. | 2,6731 | 10,466 | 6,07241 | 1,450E-04 | 0,04276 |
| KLF6 | Homo sapiens Kruppel-like factor 6 (KLF6), transcript variant 2, mRNA. | 2,4646 | 11,1617 | 7,79743 | 1,925E-05 | 0,01920 |
| SERPINE1 | Homo sapiens serpin peptidase inhibitor, clade E (nexin, plasminogen activator inhibitor type 1), member 1 (SERPINE1), mRNA. | 2,4170 | 10,0778 | 8,09858 | 1,400E-05 | 0,01662 |
| CDKN1A | Homo sapiens cyclin-dependent kinase inhibitor 1A (p21, Cip1) (CDKN1A), transcript variant 1, mRNA. | 2,3019 | 11,5096 | 6,20365 | 1,228E-04 | 0,04011 |
| KLF6 | Homo sapiens Kruppel-like factor 6 (KLF6), transcript variant 1, mRNA. | 2,1922 | 10,3859 | 6,51605 | 8,336E-05 | 0,03387 |
| RHOB | Homo sapiens ras homolog gene family, member B (RHOB), mRNA. | 2,1676 | 10,1512 | 7,77621 | 1,969E-05 | 0,01920 |
| KLF2 | Homo sapiens Kruppel-like factor 2 (lung) (KLF2), mRNA. | 1,8393 | 9,6186 | 8,29697 | 1,140E-05 | 0,01662 |
| PHLDA1 | Homo sapiens pleckstrin homology-like domain, family A, member 1 (PHLDA1), mRNA. | 1,7319 | 12,2499 | 7,13147 | 4,027E-05 | 0,02438 |
| PLAU | Homo sapiens plasminogen activator, urokinase (PLAU), mRNA. | 1,6140 | 12,7476 | 8,82932 | 6,703E-06 | 0,01545 |
| FOSL1 | Homo sapiens FOS-like antigen 1 (FOSL1), mRNA. | 1,6027 | 10,3679 | 6,87592 | 5,418E-05 | 0,02862 |
| EMP1 | Homo sapiens epithelial membrane protein 1 (EMP1), mRNA. | 1,5630 | 10,7881 | 7,55856 | 2,494E-05 | 0,01979 |
| LAMB3 | Homo sapiens laminin, beta 3 (LAMB3), transcript variant 1, mRNA. | 1,4813 | 12,8511 | 6,35336 | 1,018E-04 | 0,03684 |
| BTG1 | Homo sapiens B-cell translocation gene 1, anti-proliferative (BTG1), mRNA. | 1,3921 | 10,9394 | 5,93559 | 1,729E-04 | 0,04574 |
| DUSP5 | Homo sapiens dual specificity phosphatase 5 (DUSP5), mRNA. | 1,3323 | 10,4625 | 5,84452 | 1,946E-04 | 0,04737 |
| UPP1 | Homo sapiens uridine phosphorylase 1 (UPP1), transcript variant 1, mRNA. | 1,2599 | 11,0534 | 7,39560 | 2,987E-05 | 0,02152 |
| SLC16A3 | Homo sapiens solute carrier family 16, member 3 (monocarboxylic acid transporter 4) (SLC16A3), transcript variant 2, mRNA. | 1,2246 | 8,7470 | 6,15272 | 1,309E-04 | 0,04016 |
| CITED4 | Homo sapiens Cbp/p300-interacting transactivator, with Glu/Asp-rich carboxy-terminal domain, 4 (CITED4), mRNA. | 1,1991 | 9,9969 | 6,19537 | 1,241E-04 | 0,04011 |
| SLC20A1 | Homo sapiens solute carrier family 20 (phosphate transporter), member 1 (SLC20A1), mRNA. | 1,1923 | 11,4086 | 8,17157 | 1,297E-05 | 0,01662 |
| FLNB | Homo sapiens filamin B, beta (actin binding protein 278) (FLNB), mRNA. | 1,1736 | 10,3910 | 8,52813 | 9,025E-06 | 0,01662 |
| PLEK2 | Homo sapiens pleckstrin 2 (PLEK2), mRNA. | 1,1684 | 10,0090 | 5,82968 | 1,985E-04 | 0,04737 |
| MIR1974 | Homo sapiens microRNA 1974 (MIR1974), microRNA. | 1,0965 | 11,8289 | 9,09525 | 5,189E-06 | 0,01462 |
| TUBB2A | Homo sapiens tubulin, beta 2A (TUBB2A), mRNA. | 1,0724 | 9,8472 | 6,01605 | 1,559E-04 | 0,04441 |
| ETV5 | Homo sapiens ets variant gene 5 (ets-related molecule) (ETV5), mRNA. | 1,0684 | 9,0801 | 5,94898 | 1,699E-04 | 0,04574 |
| MYADM | Homo sapiens myeloid-associated differentiation marker (MYADM), transcript variant 4, mRNA. | 1,0577 | 9,4204 | 7,09781 | 4,186E-05 | 0,02438 |
| LCP1 | Homo sapiens lymphocyte cytosolic protein 1 (L-plastin) (LCP1), mRNA. | 1,0214 | 9,9341 | 8,37920 | 1,049E-05 | 0,01662 |
| BCL2L1 | Homo sapiens BCL2-like 1 (BCL2L1), nuclear gene encoding mitochondrial protein, transcript variant 1, mRNA. | 1,0029 | 11,4377 | 6,14957 | 1,315E-04 | 0,04016 |
| TMEM154 | Homo sapiens transmembrane protein 154 (TMEM154), mRNA. | 0,9921 | 8,8333 | 6,03891 | 1,513E-04 | 0,04412 |
| SELS | Homo sapiens selenoprotein S (SELS), transcript variant 2, mRNA. | 0,9505 | 10,8747 | 6,30980 | 1,075E-04 | 0,03684 |
| RHOC | Homo sapiens ras homolog gene family, member C (RHOC), transcript variant 1, mRNA. | 0,9207 | 11,8562 | 5,80595 | 2,047E-04 | 0,04767 |
| PRNP | Homo sapiens prion protein (PRNP), transcript variant 3, mRNA. | 0,8527 | 12,6573 | 7,68332 | 2,177E-05 | 0,01971 |
| LOC729768 | PREDICTED: Homo sapiens misc_RNA (LOC729768), miscRNA. | 0,8407 | 11,6726 | 6,42923 | 9,271E-05 | 0,03432 |
| EHD1 | Homo sapiens EH-domain containing 1 (EHD1), mRNA. | 0,8384 | 9,6096 | 5,77850 | 2,122E-04 | 0,04849 |
| VIL2 | Homo sapiens villin 2 (ezrin) (VIL2), mRNA. | 0,8339 | 12,4829 | 8,26381 | 1,180E-05 | 0,01662 |
| GLIPR1 | Homo sapiens GLI pathogenesis-related 1 (GLIPR1), mRNA. | 0,8263 | 8,3514 | 6,51035 | 8,394E-05 | 0,03387 |
| LOC402221 | PREDICTED: Homo sapiens similar to actin alpha 1 skeletal muscle protein (LOC402221), mRNA. | 0,8235 | 10,1546 | 5,94431 | 1,710E-04 | 0,04574 |
| PGM3 | Homo sapiens phosphoglucomutase 3 (PGM3), mRNA. | 0,8012 | 9,0847 | 7,71480 | 2,104E-05 | 0,01971 |
| UAP1 | Homo sapiens UDP-N-acteylglucosamine pyrophosphorylase 1 (UAP1), mRNA. | 0,7754 | 9,1731 | 5,91484 | 1,776E-04 | 0,04611 |
| PGM3 | Homo sapiens phosphoglucomutase 3 (PGM3), mRNA. | 0,7330 | 9,6699 | 6,84270 | 5,634E-05 | 0,02876 |
| LOC654103 | PREDICTED: Homo sapiens similar to solute carrier family 25, member 37 (LOC654103), mRNA. | 0,6597 | 8,9399 | 5,95537 | 1,685E-04 | 0,04574 |
| GFPT1 | Homo sapiens glutamine-fructose-6-phosphate transaminase 1 (GFPT1), mRNA. | 0,6284 | 9,0827 | 6,48513 | 8,657E-05 | 0,03387 |
| FKSG30 | Homo sapiens actin-like protein (FKSG30), mRNA. | 0,6242 | 12,8546 | 6,71541 | 6,553E-05 | 0,03021 |
| EZR | Homo sapiens ezrin (EZR), transcript variant 1, mRNA. | 0,6179 | 12,9747 | 9,11623 | 5,087E-06 | 0,01462 |
| GPR172A | Homo sapiens G protein-coupled receptor 172A (GPR172A), mRNA. | 0,5712 | 11,0961 | 6,81908 | 5,793E-05 | 0,02881 |
| BRI3 | Homo sapiens brain protein I3 (BRI3), mRNA. | 0,5682 | 11,0048 | 6,90545 | 5,233E-05 | 0,02862 |
| ITGB1 | Homo sapiens integrin, beta 1 (fibronectin receptor, beta polypeptide, antigen CD29 includes MDF2, MSK12) (ITGB1), transcript variant 1C-2, mRNA. | 0,5260 | 11,5085 | 5,84032 | 1,957E-04 | 0,04737 |
| GFPT1 | Homo sapiens glutamine-fructose-6-phosphate transaminase 1 (GFPT1), mRNA. | 0,5154 | 9,3057 | 6,10964 | 1,383E-04 | 0,04175 |
| TPM4 | Homo sapiens tropomyosin 4 (TPM4), mRNA. | 0,5024 | 9,5569 | 6,42329 | 9,339E-05 | 0,03432 |
| RBM23 | Homo sapiens RNA binding motif protein 23 (RBM23), transcript variant 3, mRNA. | -0,5272 | 9,5000 | -6,95032 | 4,966E-05 | 0,02798 |
| TRIM6 | Homo sapiens tripartite motif-containing 6 (TRIM6), transcript variant 2, mRNA. | -0,5354 | 7,8005 | -7,34587 | 3,158E-05 | 0,02165 |
| TGIF2 | Homo sapiens TGFB-induced factor homeobox 2 (TGIF2), mRNA. | -0,5383 | 7,8910 | -7,55760 | 2,497E-05 | 0,01979 |
| HSPA2 | Homo sapiens heat shock 70kDa protein 2 (HSPA2), mRNA. | -0,5573 | 7,8547 | -5,93449 | 1,731E-04 | 0,04574 |
| PRICKLE1 | Homo sapiens prickle homolog 1 (Drosophila) (PRICKLE1), mRNA. | -0,5708 | 7,9931 | -7,59933 | 2,385E-05 | 0,01979 |
| ARHGEF19 | Homo sapiens Rho guanine nucleotide exchange factor (GEF) 19 (ARHGEF19), mRNA. | -0,5968 | 7,7448 | -6,83723 | 5,670E-05 | 0,02876 |
| NASP | Homo sapiens nuclear autoantigenic sperm protein (histone-binding) (NASP), transcript variant 2, mRNA. | -0,7089 | 7,8079 | -6,46052 | 8,922E-05 | 0,03428 |
| ARTN | Homo sapiens artemin (ARTN), transcript variant 2, mRNA. | -0,8039 | 8,9167 | -6,80213 | 5,911E-05 | 0,02882 |
| E2F2 | Homo sapiens E2F transcription factor 2 (E2F2), mRNA. | -0,9405 | 7,7153 | -11,13475 | 8,754E-07 | 0,00464 |
| LYPD1 | Homo sapiens LY6/PLAUR domain containing 1 (LYPD1), transcript variant 1, mRNA. | -1,0121 | 7,8731 | -6,16280 | 1,293E-04 | 0,04016 |
| SOX2 | Homo sapiens SRY (sex determining region Y)-box 2 (SOX2), mRNA. | -1,0622 | 7,7898 | -10,85659 | 1,097E-06 | 0,00464 |
| LOC100134073 | PREDICTED: Homo sapiens similar to LYPDC1 protein (LOC100134073), mRNA. | -1,1984 | 7,9358 | -5,78309 | 2,110E-04 | 0,04849 |
| SOX2 | Homo sapiens SRY (sex determining region Y)-box 2 (SOX2), mRNA. | -1,2035 | 7,9056 | -10,94866 | 1,017E-06 | 0,00464 |
