# Supplementary material for: Induction of ER and mitochondrial stress by the alkylphosphocholine erufosine in oral squamous cell carcinoma cells
Source: Cell Death Dis. 2018 Feb 20;9(3):296. doi: 10.1038/s41419-018-0342-2 (PMC5833417; doi:10.1038/s41419-018-0342-2)
Supplement: Supplementary file 2 — Supplementary Table 1b [file 41419_2018_342_MOESM2_ESM.docx]

**Table S1b: Differential regulation of genes upon IC50 exposure of erufosine in HN-5 cells**

| **Symbol** | **Definition** | **Log Fold Change** | **Average Expression** | **t-statistics** | **P.Value** | **adj.P.Val** |
| --- | --- | --- | --- | --- | --- | --- |
| HBEGF | Homo sapiens heparin-binding EGF-like growth factor (HBEGF), mRNA. | 3,93841 | 10,49675 | 9,07395 | 0,00001 | 0,00189 |
| KLF6 | Homo sapiens Kruppel-like factor 6 (KLF6), transcript variant 2, mRNA. | 3,66955 | 11,16168 | 11,60968 | 0,00000 | 0,00065 |
| CDKN1A | Homo sapiens cyclin-dependent kinase inhibitor 1A (p21, Cip1) (CDKN1A), transcript variant 1, mRNA. | 3,55917 | 11,50962 | 9,59188 | 0,00000 | 0,00167 |
| KLF6 | Homo sapiens Kruppel-like factor 6 (KLF6), transcript variant 1, mRNA. | 3,44717 | 10,38590 | 10,24644 | 0,00000 | 0,00122 |
| ANGPTL4 | Homo sapiens angiopoietin-like 4 (ANGPTL4), transcript variant 1, mRNA. | 3,39893 | 9,83629 | 8,24191 | 0,00001 | 0,00262 |
| TM4SF19 | Homo sapiens transmembrane 4 L six family member 19 (TM4SF19), mRNA. | 3,36515 | 10,46612 | 7,64444 | 0,00002 | 0,00334 |
| CSF2 | Homo sapiens colony stimulating factor 2 (granulocyte-macrophage) (CSF2), mRNA. | 3,33083 | 9,65573 | 7,91830 | 0,00002 | 0,00315 |
| FAM83A | Homo sapiens family with sequence similarity 83, member A (FAM83A), transcript variant 1, mRNA. | 3,29797 | 9,38552 | 4,12358 | 0,00228 | 0,02873 |
| FAM83A | Homo sapiens family with sequence similarity 83, member A (FAM83A), transcript variant 2, mRNA. | 3,29223 | 9,39537 | 3,84105 | 0,00354 | 0,03726 |
| RHOB | Homo sapiens ras homolog gene family, member B (RHOB), mRNA. | 3,20507 | 10,15124 | 11,49822 | 0,00000 | 0,00065 |
| IL1B | Homo sapiens interleukin 1, beta (IL1B), mRNA. | 3,11814 | 11,03957 | 4,13765 | 0,00223 | 0,02831 |
| MMP10 | Homo sapiens matrix metallopeptidase 10 (stromelysin 2) (MMP10), mRNA. | 3,07910 | 10,76432 | 6,09683 | 0,00014 | 0,00709 |
| TRIB1 | Homo sapiens tribbles homolog 1 (Drosophila) (TRIB1), mRNA. | 3,06340 | 10,40049 | 7,63961 | 0,00002 | 0,00334 |
| TM4SF19 | PREDICTED: Homo sapiens transmembrane 4 L six family member 19, transcript variant 2 (TM4SF19), mRNA. | 2,83416 | 9,57136 | 5,77513 | 0,00021 | 0,00853 |
| KLF2 | Homo sapiens Kruppel-like factor 2 (lung) (KLF2), mRNA. | 2,81284 | 9,61864 | 12,68859 | 0,00000 | 0,00056 |
| ODC1 | Homo sapiens ornithine decarboxylase 1 (ODC1), mRNA. | 2,79754 | 11,91395 | 8,27420 | 0,00001 | 0,00260 |
| LAMC2 | Homo sapiens laminin, gamma 2 (LAMC2), transcript variant 1, mRNA. | 2,77069 | 11,26640 | 6,56950 | 0,00008 | 0,00552 |
| LOC100132564 | PREDICTED: Homo sapiens hypothetical protein LOC100132564 (LOC100132564), mRNA. | 2,58602 | 11,23851 | 6,48953 | 0,00009 | 0,00582 |
| LAMC2 | Homo sapiens laminin, gamma 2 (LAMC2), transcript variant 2, mRNA. | 2,58111 | 9,59296 | 5,85624 | 0,00019 | 0,00807 |
| LAMC2 | Homo sapiens laminin, gamma 2 (LAMC2), transcript variant 1, mRNA. | 2,58102 | 11,91874 | 5,67150 | 0,00024 | 0,00911 |
| TRIB3 | Homo sapiens tribbles homolog 3 (Drosophila) (TRIB3), mRNA. | 2,56496 | 9,95780 | 6,86224 | 0,00006 | 0,00475 |
| LOC650517 | PREDICTED: Homo sapiens hypothetical LOC650517 (LOC650517), mRNA. | 2,54408 | 10,73639 | 4,93194 | 0,00068 | 0,01517 |
| LOC100008589 | Homo sapiens 28S ribosomal RNA (LOC100008589), non-coding RNA. | 2,49386 | 11,07999 | 5,52864 | 0,00030 | 0,00999 |
| DUSP5 | Homo sapiens dual specificity phosphatase 5 (DUSP5), mRNA. | 2,48053 | 10,46253 | 10,88149 | 0,00000 | 0,00088 |
| PHLDA1 | Homo sapiens pleckstrin homology-like domain, family A, member 1 (PHLDA1), mRNA. | 2,44494 | 12,24987 | 10,06777 | 0,00000 | 0,00126 |
| SERPINE1 | Homo sapiens serpin peptidase inhibitor, clade E (nexin, plasminogen activator inhibitor type 1), member 1 (SERPINE1), mRNA. | 2,41432 | 10,07778 | 8,08953 | 0,00001 | 0,00284 |
| PPP1R15A | Homo sapiens protein phosphatase 1, regulatory (inhibitor) subunit 15A (PPP1R15A), mRNA. | 2,35403 | 10,97953 | 7,73701 | 0,00002 | 0,00326 |
| PLAUR | Homo sapiens plasminogen activator, urokinase receptor (PLAUR), transcript variant 2, mRNA. | 2,35244 | 9,57540 | 7,12204 | 0,00004 | 0,00422 |
| SERPINB1 | Homo sapiens serpin peptidase inhibitor, clade B (ovalbumin), member 1 (SERPINB1), mRNA. | 2,34708 | 8,93339 | 4,01319 | 0,00270 | 0,03186 |
| NDRG1 | Homo sapiens N-myc downstream regulated gene 1 (NDRG1), mRNA. | 2,32984 | 12,37854 | 5,72412 | 0,00023 | 0,00886 |
| LOC441019 | PREDICTED: Homo sapiens hypothetical LOC441019 (LOC441019), mRNA. | 2,21497 | 11,19378 | 3,99218 | 0,00279 | 0,03244 |
| SPRY2 | Homo sapiens sprouty homolog 2 (Drosophila) (SPRY2), mRNA. | 2,16243 | 9,10997 | 4,20873 | 0,00199 | 0,02654 |
| PLAU | Homo sapiens plasminogen activator, urokinase (PLAU), mRNA. | 2,15250 | 12,74764 | 11,77527 | 0,00000 | 0,00065 |
| BTG1 | Homo sapiens B-cell translocation gene 1, anti-proliferative (BTG1), mRNA. | 2,14964 | 10,93937 | 9,16534 | 0,00000 | 0,00188 |
| GJB3 | Homo sapiens gap junction protein, beta 3, 31kDa (GJB3), transcript variant 2, mRNA. | 2,13741 | 10,12816 | 7,10251 | 0,00004 | 0,00422 |
| LAMB3 | Homo sapiens laminin, beta 3 (LAMB3), transcript variant 1, mRNA. | 2,12258 | 12,85113 | 9,10365 | 0,00001 | 0,00188 |
| ISG20 | Homo sapiens interferon stimulated exonuclease gene 20kDa (ISG20), mRNA. | 2,10418 | 9,91594 | 5,19799 | 0,00047 | 0,01245 |
| PLAUR | Homo sapiens plasminogen activator, urokinase receptor (PLAUR), transcript variant 1, mRNA. | 2,10239 | 9,19092 | 6,95559 | 0,00005 | 0,00449 |
| LOC100134364 | PREDICTED: Homo sapiens hypothetical protein LOC100134364 (LOC100134364), mRNA. | 2,07743 | 10,93696 | 5,52022 | 0,00030 | 0,01005 |
| LOC100132394 | PREDICTED: Homo sapiens hypothetical protein LOC100132394 (LOC100132394), mRNA. | 2,05773 | 11,37482 | 5,69896 | 0,00024 | 0,00902 |
| AKAP12 | Homo sapiens A kinase (PRKA) anchor protein (gravin) 12 (AKAP12), transcript variant 2, mRNA. | 2,04644 | 8,90755 | 7,36050 | 0,00003 | 0,00386 |
| LAMA3 | Homo sapiens laminin, alpha 3 (LAMA3), transcript variant 1, mRNA. | 2,04516 | 9,73462 | 4,58943 | 0,00112 | 0,01957 |
| NT5E | Homo sapiens 5'-nucleotidase, ecto (CD73) (NT5E), mRNA. | 2,04099 | 9,16091 | 4,17819 | 0,00209 | 0,02727 |
| C14orf78 | PREDICTED: Homo sapiens chromosome 14 open reading frame 78 (C14orf78), mRNA. | 2,03051 | 9,35838 | 6,52555 | 0,00008 | 0,00567 |
| AHNAK2 | Homo sapiens AHNAK nucleoprotein 2 (AHNAK2), mRNA. | 1,97306 | 9,11497 | 6,43172 | 0,00009 | 0,00606 |
| KRT17P3 | PREDICTED: Homo sapiens misc_RNA (KRT17P3), miscRNA. | 1,95605 | 11,88042 | 4,33096 | 0,00165 | 0,02419 |
| ITGA2 | Homo sapiens integrin, alpha 2 (CD49B, alpha 2 subunit of VLA-2 receptor) (ITGA2), mRNA. | 1,95565 | 10,84689 | 7,16915 | 0,00004 | 0,00418 |
| IER3 | Homo sapiens immediate early response 3 (IER3), mRNA. | 1,93910 | 12,79992 | 6,14264 | 0,00013 | 0,00704 |
| ERRFI1 | Homo sapiens ERBB receptor feedback inhibitor 1 (ERRFI1), mRNA. | 1,93034 | 10,53577 | 6,82349 | 0,00006 | 0,00479 |
| ARHGEF2 | Homo sapiens rho/rac guanine nucleotide exchange factor (GEF) 2 (ARHGEF2), mRNA. | 1,92340 | 10,19904 | 8,54524 | 0,00001 | 0,00236 |
| IL1A | Homo sapiens interleukin 1, alpha (IL1A), mRNA. | 1,91772 | 11,28831 | 7,74395 | 0,00002 | 0,00325 |
| EMP1 | Homo sapiens epithelial membrane protein 1 (EMP1), mRNA. | 1,91284 | 10,78806 | 9,25021 | 0,00000 | 0,00188 |
| UPP1 | Homo sapiens uridine phosphorylase 1 (UPP1), transcript variant 1, mRNA. | 1,90570 | 11,05342 | 11,18688 | 0,00000 | 0,00076 |
| TGFA | Homo sapiens transforming growth factor, alpha (TGFA), mRNA. | 1,89738 | 9,58148 | 6,23302 | 0,00012 | 0,00671 |
| LOC387841 | PREDICTED: Homo sapiens similar to ribosomal protein L13a, transcript variant 2 (LOC387841), mRNA. | 1,87855 | 9,80741 | 5,22623 | 0,00045 | 0,01212 |
| ULK1 | Homo sapiens unc-51-like kinase 1 (C. elegans) (ULK1), mRNA. | 1,87767 | 10,44580 | 5,18343 | 0,00048 | 0,01260 |
| MT2A | Homo sapiens metallothionein 2A (MT2A), mRNA. | 1,87579 | 12,66393 | 4,72578 | 0,00092 | 0,01755 |
| MALL | Homo sapiens mal, T-cell differentiation protein-like (MALL), mRNA. | 1,86062 | 10,35042 | 6,04256 | 0,00015 | 0,00728 |
| ECGF1 | Homo sapiens endothelial cell growth factor 1 (platelet-derived) (ECGF1), mRNA. | 1,85579 | 10,40934 | 9,88019 | 0,00000 | 0,00142 |
| CGB5 | Homo sapiens chorionic gonadotropin, beta polypeptide 5 (CGB5), mRNA. | 1,84189 | 8,90877 | 5,57815 | 0,00028 | 0,00970 |
| SLC16A3 | Homo sapiens solute carrier family 16, member 3 (monocarboxylic acid transporter 4) (SLC16A3), transcript variant 2, mRNA. | 1,83473 | 8,74702 | 9,21824 | 0,00000 | 0,00188 |
| FOSL1 | Homo sapiens FOS-like antigen 1 (FOSL1), mRNA. | 1,82904 | 10,36786 | 7,84686 | 0,00002 | 0,00322 |
| PIM1 | Homo sapiens pim-1 oncogene (PIM1), mRNA. | 1,82164 | 9,12018 | 8,42240 | 0,00001 | 0,00244 |
| LOC100008589 | Homo sapiens 28S ribosomal RNA (LOC100008589), non-coding RNA. | 1,81919 | 12,95099 | 8,25700 | 0,00001 | 0,00262 |
| ASNS | Homo sapiens asparagine synthetase (ASNS), transcript variant 1, mRNA. | 1,80628 | 11,28671 | 4,09918 | 0,00236 | 0,02942 |
| SAT1 | Homo sapiens spermidine/spermine N1-acetyltransferase 1 (SAT1), mRNA. | 1,80526 | 11,07082 | 6,78625 | 0,00006 | 0,00479 |
| LAMA3 | Homo sapiens laminin, alpha 3 (LAMA3), transcript variant 1, mRNA. | 1,80448 | 9,02605 | 3,89030 | 0,00328 | 0,03555 |
| ULK1 | Homo sapiens unc-51-like kinase 1 (C. elegans) (ULK1), mRNA. | 1,79931 | 10,15535 | 4,89848 | 0,00071 | 0,01563 |
| RAC2 | Homo sapiens ras-related C3 botulinum toxin substrate 2 (rho family, small GTP binding protein Rac2) (RAC2), mRNA. | 1,79402 | 11,65662 | 6,82136 | 0,00006 | 0,00479 |
| ANTXR2 | Homo sapiens anthrax toxin receptor 2 (ANTXR2), mRNA. | 1,77249 | 8,80744 | 6,15115 | 0,00013 | 0,00699 |
| TNFRSF25 | Homo sapiens tumor necrosis factor receptor superfamily, member 25 (TNFRSF25), transcript variant 10, mRNA. | 1,75058 | 9,35920 | 5,45042 | 0,00033 | 0,01050 |
| TMBIM1 | Homo sapiens transmembrane BAX inhibitor motif containing 1 (TMBIM1), mRNA. | 1,74867 | 9,55475 | 4,79646 | 0,00083 | 0,01665 |
| RAP1GAP | Homo sapiens RAP1 GTPase activating protein (RAP1GAP), mRNA. | 1,74611 | 9,36659 | 7,78293 | 0,00002 | 0,00325 |
| PLEC1 | Homo sapiens plectin 1, intermediate filament binding protein 500kDa (PLEC1), transcript variant 1, mRNA. | 1,73879 | 10,64046 | 6,12410 | 0,00014 | 0,00709 |
| FLNB | Homo sapiens filamin B, beta (actin binding protein 278) (FLNB), mRNA. | 1,73454 | 10,39104 | 12,60396 | 0,00000 | 0,00056 |
| GRB7 | Homo sapiens growth factor receptor-bound protein 7 (GRB7), transcript variant 2, mRNA. | 1,72887 | 9,21895 | 7,14033 | 0,00004 | 0,00422 |
| KRT16 | Homo sapiens keratin 16 (focal non-epidermolytic palmoplantar keratoderma) (KRT16), mRNA. | 1,72239 | 11,08467 | 3,61994 | 0,00505 | 0,04604 |
| MYADM | Homo sapiens myeloid-associated differentiation marker (MYADM), transcript variant 4, mRNA. | 1,72119 | 9,42038 | 11,55033 | 0,00000 | 0,00065 |
| CDCP1 | Homo sapiens CUB domain containing protein 1 (CDCP1), transcript variant 1, mRNA. | 1,71441 | 9,31416 | 6,95849 | 0,00005 | 0,00449 |
| TUBB2A | Homo sapiens tubulin, beta 2A (TUBB2A), mRNA. | 1,70886 | 9,84717 | 9,58641 | 0,00000 | 0,00167 |
| KCNK1 | Homo sapiens potassium channel, subfamily K, member 1 (KCNK1), mRNA. | 1,69978 | 9,74288 | 4,88063 | 0,00073 | 0,01579 |
| FERMT1 | Homo sapiens fermitin family homolog 1 (Drosophila) (FERMT1), mRNA. | 1,69032 | 10,08223 | 4,86675 | 0,00075 | 0,01587 |
| DUSP6 | Homo sapiens dual specificity phosphatase 6 (DUSP6), transcript variant 2, mRNA. | 1,66828 | 9,85705 | 5,04643 | 0,00058 | 0,01383 |
| TGFA | Homo sapiens transforming growth factor, alpha (TGFA), transcript variant 2, mRNA. | 1,66461 | 9,14896 | 5,99901 | 0,00016 | 0,00743 |
| ASNS | Homo sapiens asparagine synthetase (ASNS), transcript variant 1, mRNA. | 1,65656 | 9,47989 | 4,75254 | 0,00088 | 0,01721 |
| KRT17 | Homo sapiens keratin 17 (KRT17), mRNA. | 1,65382 | 12,23817 | 4,42242 | 0,00144 | 0,02246 |
| MTSS1 | Homo sapiens metastasis suppressor 1 (MTSS1), mRNA. | 1,63410 | 9,94104 | 7,42024 | 0,00003 | 0,00370 |
| KCNK1 | Homo sapiens potassium channel, subfamily K, member 1 (KCNK1), mRNA. | 1,63237 | 9,23906 | 4,18304 | 0,00208 | 0,02717 |
| SPRR1A | Homo sapiens small proline-rich protein 1A (SPRR1A), mRNA. | 1,62683 | 8,59386 | 5,29210 | 0,00041 | 0,01162 |
| CAPRIN2 | Homo sapiens caprin family member 2 (CAPRIN2), transcript variant 1, mRNA. | 1,62642 | 9,74203 | 6,11265 | 0,00014 | 0,00709 |
| MMP9 | Homo sapiens matrix metallopeptidase 9 (gelatinase B, 92kDa gelatinase, 92kDa type IV collagenase) (MMP9), mRNA. | 1,61888 | 8,84094 | 4,28524 | 0,00177 | 0,02510 |
| CITED4 | Homo sapiens Cbp/p300-interacting transactivator, with Glu/Asp-rich carboxy-terminal domain, 4 (CITED4), mRNA. | 1,61197 | 9,99686 | 8,32875 | 0,00001 | 0,00252 |
| MXD1 | Homo sapiens MAX dimerization protein 1 (MXD1), mRNA. | 1,59731 | 8,66462 | 7,52967 | 0,00003 | 0,00357 |
| SH3PXD2A | Homo sapiens SH3 and PX domains 2A (SH3PXD2A), mRNA. | 1,58287 | 10,30422 | 4,78161 | 0,00085 | 0,01689 |
| SELS | Homo sapiens selenoprotein S (SELS), transcript variant 2, mRNA. | 1,57801 | 10,87473 | 10,47515 | 0,00000 | 0,00112 |
| PHLDA1 | Homo sapiens pleckstrin homology-like domain, family A, member 1 (PHLDA1), mRNA. | 1,57111 | 8,77963 | 4,23956 | 0,00190 | 0,02607 |
| STX1A | Homo sapiens syntaxin 1A (brain) (STX1A), mRNA. | 1,56736 | 8,66318 | 6,42286 | 0,00009 | 0,00609 |
| S100A6 | Homo sapiens S100 calcium binding protein A6 (S100A6), mRNA. | 1,56671 | 11,31604 | 3,79482 | 0,00382 | 0,03886 |
| PLEK2 | Homo sapiens pleckstrin 2 (PLEK2), mRNA. | 1,56134 | 10,00903 | 7,79008 | 0,00002 | 0,00325 |
| DUSP1 | Homo sapiens dual specificity phosphatase 1 (DUSP1), mRNA. | 1,55990 | 9,04368 | 6,83545 | 0,00006 | 0,00479 |
| LOC653506 | PREDICTED: Homo sapiens similar to meteorin, glial cell differentiation regulator-like (LOC653506), mRNA. | 1,55824 | 8,70328 | 6,38063 | 0,00010 | 0,00614 |
| IRF9 | Homo sapiens interferon regulatory factor 9 (IRF9), mRNA. | 1,55510 | 8,95232 | 5,04667 | 0,00058 | 0,01383 |
| CD68 | Homo sapiens CD68 antigen (CD68), mRNA. | 1,54473 | 10,18266 | 3,63794 | 0,00491 | 0,04522 |
| BCAR3 | Homo sapiens breast cancer anti-estrogen resistance 3 (BCAR3), mRNA. | 1,54458 | 10,16351 | 6,04305 | 0,00015 | 0,00728 |
| CGB1 | Homo sapiens chorionic gonadotropin, beta polypeptide 1 (CGB1), mRNA. | 1,53792 | 8,68094 | 5,26040 | 0,00043 | 0,01185 |
| WIPI1 | Homo sapiens WD repeat domain, phosphoinositide interacting 1 (WIPI1), mRNA. | 1,53398 | 8,74354 | 8,90456 | 0,00001 | 0,00198 |
| BHLHB2 | Homo sapiens basic helix-loop-helix domain containing, class B, 2 (BHLHB2), mRNA. | 1,53107 | 11,00627 | 5,45991 | 0,00033 | 0,01048 |
| LOC100008588 | Homo sapiens 18S ribosomal RNA (LOC100008588), non-coding RNA. | 1,52624 | 11,30533 | 4,18663 | 0,00206 | 0,02709 |
| MIR1974 | Homo sapiens microRNA 1974 (MIR1974), microRNA. | 1,51871 | 11,82894 | 12,59746 | 0,00000 | 0,00056 |
| CEBPB | Homo sapiens CCAAT/enhancer binding protein (C/EBP), beta (CEBPB), mRNA. | 1,50109 | 12,10256 | 7,74330 | 0,00002 | 0,00325 |
| LARP6 | Homo sapiens La ribonucleoprotein domain family, member 6 (LARP6), transcript variant 1, mRNA. | 1,49972 | 9,08465 | 8,45806 | 0,00001 | 0,00238 |
| C5orf32 | Homo sapiens chromosome 5 open reading frame 32 (C5orf32), mRNA. | 1,49763 | 9,23920 | 5,67153 | 0,00024 | 0,00911 |
| OAF | Homo sapiens OAF homolog (Drosophila) (OAF), mRNA. | 1,48621 | 9,68202 | 4,42080 | 0,00144 | 0,02247 |
| PLAUR | Homo sapiens plasminogen activator, urokinase receptor (PLAUR), transcript variant 2, mRNA. | 1,48081 | 8,65266 | 4,51492 | 0,00125 | 0,02067 |
| CSNK1E | Homo sapiens casein kinase 1, epsilon (CSNK1E), transcript variant 1, mRNA. | 1,47303 | 10,62446 | 8,54857 | 0,00001 | 0,00236 |
| IL11 | Homo sapiens interleukin 11 (IL11), mRNA. | 1,46589 | 8,66764 | 6,10427 | 0,00014 | 0,00709 |
| CPXM1 | Homo sapiens carboxypeptidase X (M14 family), member 1 (CPXM1), mRNA. | 1,45678 | 8,89281 | 5,19892 | 0,00047 | 0,01245 |
| JUN | Homo sapiens jun oncogene (JUN), mRNA. | 1,45151 | 10,79761 | 7,10243 | 0,00004 | 0,00422 |
| OPLAH | Homo sapiens 5-oxoprolinase (ATP-hydrolysing) (OPLAH), mRNA. | 1,44850 | 8,92019 | 9,35599 | 0,00000 | 0,00184 |
| HPCAL1 | Homo sapiens hippocalcin-like 1 (HPCAL1), transcript variant 2, mRNA. | 1,44132 | 10,70770 | 6,87656 | 0,00005 | 0,00470 |
| ZBED2 | Homo sapiens zinc finger, BED-type containing 2 (ZBED2), mRNA. | 1,42826 | 8,83962 | 3,67022 | 0,00466 | 0,04385 |
| F3 | Homo sapiens coagulation factor III (thromboplastin, tissue factor) (F3), mRNA. | 1,42793 | 10,70559 | 4,85969 | 0,00075 | 0,01594 |
| IRF6 | Homo sapiens interferon regulatory factor 6 (IRF6), mRNA. | 1,42705 | 10,24959 | 6,75807 | 0,00006 | 0,00482 |
| AHNAK | Homo sapiens AHNAK nucleoprotein (AHNAK), transcript variant 1, mRNA. | 1,42697 | 10,46861 | 4,53568 | 0,00122 | 0,02030 |
| LRRC8A | Homo sapiens leucine rich repeat containing 8 family, member A (LRRC8A), mRNA. | 1,42257 | 9,38729 | 8,52684 | 0,00001 | 0,00236 |
| ANXA3 | Homo sapiens annexin A3 (ANXA3), mRNA. | 1,42206 | 9,51599 | 6,16516 | 0,00013 | 0,00695 |
| SPRY4 | Homo sapiens sprouty homolog 4 (Drosophila) (SPRY4), mRNA. | 1,41896 | 8,23377 | 5,34354 | 0,00038 | 0,01131 |
| ZFP36L2 | Homo sapiens zinc finger protein 36, C3H type-like 2 (ZFP36L2), mRNA. | 1,40904 | 8,88702 | 4,82052 | 0,00080 | 0,01634 |
| PCK2 | Homo sapiens phosphoenolpyruvate carboxykinase 2 (mitochondrial) (PCK2), nuclear gene encoding mitochondrial protein, transcript variant 1, mRNA. | 1,40774 | 8,85816 | 3,88827 | 0,00329 | 0,03559 |
| NET1 | Homo sapiens neuroepithelial cell transforming 1 (NET1), transcript variant 1, mRNA. | 1,40450 | 11,64353 | 6,06632 | 0,00015 | 0,00722 |
| LCP1 | Homo sapiens lymphocyte cytosolic protein 1 (L-plastin) (LCP1), mRNA. | 1,39946 | 9,93412 | 11,48098 | 0,00000 | 0,00065 |
| TSC22D1 | Homo sapiens TSC22 domain family, member 1 (TSC22D1), transcript variant 2, mRNA. | 1,39910 | 10,13533 | 4,36297 | 0,00158 | 0,02358 |
| ITPR3 | Homo sapiens inositol 1,4,5-triphosphate receptor, type 3 (ITPR3), mRNA. | 1,39214 | 10,61597 | 4,23163 | 0,00193 | 0,02621 |
| ATP9A | Homo sapiens ATPase, class II, type 9A (ATP9A), mRNA. | 1,39104 | 10,07937 | 7,84162 | 0,00002 | 0,00322 |
| BCL2L1 | Homo sapiens BCL2-like 1 (BCL2L1), nuclear gene encoding mitochondrial protein, transcript variant 1, mRNA. | 1,38610 | 11,43766 | 8,49890 | 0,00001 | 0,00236 |
| SPHK1 | Homo sapiens sphingosine kinase 1 (SPHK1), transcript variant 1, mRNA. | 1,38593 | 8,62963 | 5,74040 | 0,00022 | 0,00873 |
| S100A16 | Homo sapiens S100 calcium binding protein A16 (S100A16), mRNA. | 1,38341 | 11,23901 | 5,53133 | 0,00030 | 0,00999 |
| C1orf106 | Homo sapiens chromosome 1 open reading frame 106 (C1orf106), mRNA. | 1,38123 | 9,54317 | 5,26246 | 0,00043 | 0,01183 |
| NDEL1 | Homo sapiens nudE nuclear distribution gene E homolog (A. nidulans)-like 1 (NDEL1), transcript variant 2, mRNA. | 1,38014 | 10,31483 | 5,38905 | 0,00036 | 0,01102 |
| METRNL | PREDICTED: Homo sapiens meteorin, glial cell differentiation regulator-like (METRNL), mRNA. | 1,37808 | 8,50205 | 6,10580 | 0,00014 | 0,00709 |
| LIMA1 | Homo sapiens LIM domain and actin binding 1 (LIMA1), mRNA. | 1,37677 | 10,18710 | 5,94058 | 0,00017 | 0,00774 |
| SELS | Homo sapiens selenoprotein S (SELS), transcript variant 2, mRNA. | 1,37661 | 9,46883 | 9,26828 | 0,00000 | 0,00188 |
| RHOC | Homo sapiens ras homolog gene family, member C (RHOC), transcript variant 1, mRNA. | 1,37308 | 11,85616 | 8,65866 | 0,00001 | 0,00222 |
| KLC3 | Homo sapiens kinesin light chain 3 (KLC3), transcript variant 1, mRNA. | 1,36471 | 8,83947 | 5,48431 | 0,00031 | 0,01028 |
| HERPUD1 | Homo sapiens homocysteine-inducible, endoplasmic reticulum stress-inducible, ubiquitin-like domain member 1 (HERPUD1), transcript variant 3, mRNA. | 1,36312 | 9,74352 | 7,43787 | 0,00003 | 0,00368 |
| TSC22D1 | Homo sapiens TSC22 domain family, member 1 (TSC22D1), transcript variant 2, mRNA. | 1,35497 | 11,09906 | 4,77645 | 0,00085 | 0,01692 |
| SH3KBP1 | Homo sapiens SH3-domain kinase binding protein 1 (SH3KBP1), transcript variant 1, mRNA. | 1,35258 | 9,83658 | 6,15351 | 0,00013 | 0,00698 |
| CLIC4 | Homo sapiens chloride intracellular channel 4 (CLIC4), nuclear gene encoding mitochondrial protein, mRNA. | 1,34963 | 9,56375 | 7,68783 | 0,00002 | 0,00332 |
| ACSS2 | Homo sapiens acyl-CoA synthetase short-chain family member 2 (ACSS2), transcript variant 1, mRNA. | 1,34705 | 9,38072 | 5,63504 | 0,00026 | 0,00933 |
| ITGB4 | Homo sapiens integrin, beta 4 (ITGB4), transcript variant 2, mRNA. | 1,34705 | 11,22811 | 8,39012 | 0,00001 | 0,00246 |
| GNA15 | Homo sapiens guanine nucleotide binding protein (G protein), alpha 15 (Gq class) (GNA15), mRNA. | 1,34588 | 9,64492 | 6,97193 | 0,00005 | 0,00448 |
| ITGA5 | Homo sapiens integrin, alpha 5 (fibronectin receptor, alpha polypeptide) (ITGA5), mRNA. | 1,34474 | 8,86052 | 3,86772 | 0,00340 | 0,03631 |
| HERPUD1 | Homo sapiens homocysteine-inducible, endoplasmic reticulum stress-inducible, ubiquitin-like domain member 1 (HERPUD1), transcript variant 3, mRNA. | 1,34188 | 9,85245 | 7,60528 | 0,00002 | 0,00341 |
| ACSS2 | Homo sapiens acyl-CoA synthetase short-chain family member 2 (ACSS2), transcript variant 2, mRNA. | 1,34073 | 9,18352 | 5,48706 | 0,00031 | 0,01025 |
| CAPRIN2 | Homo sapiens caprin family member 2 (CAPRIN2), transcript variant 2, mRNA. | 1,33644 | 8,86824 | 5,08854 | 0,00054 | 0,01343 |
| RIOK3 | Homo sapiens RIO kinase 3 (yeast) (RIOK3), mRNA. | 1,33638 | 9,92218 | 6,46776 | 0,00009 | 0,00592 |
| NPC1 | Homo sapiens Niemann-Pick disease, type C1 (NPC1), mRNA. | 1,33397 | 9,25546 | 7,27672 | 0,00003 | 0,00401 |
| TMEM154 | Homo sapiens transmembrane protein 154 (TMEM154), mRNA. | 1,32713 | 8,83332 | 8,07798 | 0,00001 | 0,00286 |
| SH3BGRL3 | Homo sapiens SH3 domain binding glutamic acid-rich protein like 3 (SH3BGRL3), mRNA. | 1,32653 | 11,01116 | 4,47221 | 0,00134 | 0,02153 |
| FAM84B | Homo sapiens family with sequence similarity 84, member B (FAM84B), mRNA. | 1,31815 | 10,18896 | 5,91513 | 0,00018 | 0,00775 |
| ANXA2P1 | Homo sapiens annexin A2 pseudogene 1 (ANXA2P1) on chromosome 4. | 1,30980 | 11,72825 | 6,34676 | 0,00010 | 0,00624 |
| SOX9 | Homo sapiens SRY (sex determining region Y)-box 9 (campomelic dysplasia, autosomal sex-reversal) (SOX9), mRNA. | 1,29783 | 9,07857 | 3,95256 | 0,00297 | 0,03355 |
| CSNK1E | Homo sapiens casein kinase 1, epsilon (CSNK1E), transcript variant 1, mRNA. | 1,29439 | 9,56986 | 9,00206 | 0,00001 | 0,00194 |
| SLC20A1 | Homo sapiens solute carrier family 20 (phosphate transporter), member 1 (SLC20A1), mRNA. | 1,29372 | 11,40864 | 8,86694 | 0,00001 | 0,00198 |
| RNF19B | Homo sapiens ring finger protein 19B (RNF19B), mRNA. | 1,29331 | 9,32915 | 5,62644 | 0,00026 | 0,00933 |
| TSC22D3 | Homo sapiens TSC22 domain family, member 3 (TSC22D3), transcript variant 1, mRNA. | 1,29252 | 9,22442 | 5,85243 | 0,00019 | 0,00807 |
| EPAS1 | Homo sapiens endothelial PAS domain protein 1 (EPAS1), mRNA. | 1,28607 | 9,72411 | 5,44698 | 0,00033 | 0,01051 |
| ABCC3 | Homo sapiens ATP-binding cassette, sub-family C (CFTR/MRP), member 3 (ABCC3), mRNA. | 1,27986 | 9,18309 | 4,90930 | 0,00070 | 0,01546 |
| NP | Homo sapiens nucleoside phosphorylase (NP), mRNA. | 1,27747 | 9,63458 | 4,65469 | 0,00102 | 0,01844 |
| C12orf35 | Homo sapiens chromosome 12 open reading frame 35 (C12orf35), mRNA. | 1,27469 | 8,99749 | 3,86521 | 0,00341 | 0,03644 |
| PGM3 | Homo sapiens phosphoglucomutase 3 (PGM3), mRNA. | 1,27300 | 9,66988 | 11,88307 | 0,00000 | 0,00065 |
| LRRC8C | Homo sapiens leucine rich repeat containing 8 family, member C (LRRC8C), mRNA. | 1,27229 | 8,81986 | 4,47490 | 0,00133 | 0,02148 |
| VIM | Homo sapiens vimentin (VIM), mRNA. | 1,27200 | 11,38179 | 5,02277 | 0,00060 | 0,01414 |
| SMOX | Homo sapiens spermine oxidase (SMOX), transcript variant 2, mRNA. | 1,26927 | 8,51662 | 4,94164 | 0,00067 | 0,01508 |
| CD68 | Homo sapiens CD68 molecule (CD68), transcript variant 1, mRNA. | 1,26423 | 9,29240 | 4,36991 | 0,00156 | 0,02341 |
| IRAK2 | Homo sapiens interleukin-1 receptor-associated kinase 2 (IRAK2), mRNA. | 1,26260 | 8,55181 | 5,31922 | 0,00039 | 0,01140 |
| NET1 | Homo sapiens neuroepithelial cell transforming 1 (NET1), transcript variant 2, mRNA. | 1,26181 | 9,34156 | 5,28931 | 0,00041 | 0,01164 |
| ZFP36 | Homo sapiens zinc finger protein 36, C3H type, homolog (mouse) (ZFP36), mRNA. | 1,25984 | 9,95745 | 5,25259 | 0,00043 | 0,01192 |
| TIMP1 | Homo sapiens TIMP metallopeptidase inhibitor 1 (TIMP1), mRNA. | 1,25737 | 10,41281 | 5,58723 | 0,00027 | 0,00960 |
| RRAS | Homo sapiens related RAS viral (r-ras) oncogene homolog (RRAS), mRNA. | 1,24507 | 10,02297 | 5,57193 | 0,00028 | 0,00973 |
| ITGB4 | Homo sapiens integrin, beta 4 (ITGB4), transcript variant 3, mRNA. | 1,23483 | 10,26505 | 7,92601 | 0,00002 | 0,00315 |
| TMC6 | Homo sapiens transmembrane channel-like 6 (TMC6), mRNA. | 1,23106 | 8,47370 | 5,35106 | 0,00038 | 0,01129 |
| S100A2 | Homo sapiens S100 calcium binding protein A2 (S100A2), mRNA. | 1,22980 | 11,00546 | 4,48000 | 0,00132 | 0,02137 |
| KIAA1539 | Homo sapiens KIAA1539 (KIAA1539), mRNA. | 1,22614 | 8,36292 | 5,88514 | 0,00018 | 0,00789 |
| VIM | Homo sapiens vimentin (VIM), mRNA. | 1,22174 | 12,17271 | 4,72304 | 0,00092 | 0,01757 |
| ZFP36L1 | Homo sapiens zinc finger protein 36, C3H type-like 1 (ZFP36L1), mRNA. | 1,21932 | 9,63443 | 6,26049 | 0,00011 | 0,00663 |
| RHOC | Homo sapiens ras homolog gene family, member C (RHOC), transcript variant 2, mRNA. | 1,21597 | 10,12231 | 4,96874 | 0,00065 | 0,01475 |
| SPIRE1 | Homo sapiens spire homolog 1 (Drosophila) (SPIRE1), transcript variant 2, mRNA. | 1,21271 | 10,43094 | 6,79881 | 0,00006 | 0,00479 |
| PPP2R2C | Homo sapiens protein phosphatase 2 (formerly 2A), regulatory subunit B, gamma isoform (PPP2R2C), transcript variant 2, mRNA. | 1,21249 | 8,71807 | 4,89466 | 0,00072 | 0,01569 |
| IL8 | Homo sapiens interleukin 8 (IL8), mRNA. | 1,21233 | 8,67154 | 6,24053 | 0,00012 | 0,00668 |
| RIOK3 | Homo sapiens RIO kinase 3 (yeast) (RIOK3), transcript variant 1, mRNA. | 1,21102 | 9,80423 | 5,01332 | 0,00061 | 0,01424 |
| KIAA1949 | Homo sapiens KIAA1949 (KIAA1949), mRNA. | 1,20881 | 9,88498 | 5,66746 | 0,00025 | 0,00912 |
| HMGA1 | Homo sapiens high mobility group AT-hook 1 (HMGA1), transcript variant 1, mRNA. | 1,20621 | 12,00058 | 3,71988 | 0,00430 | 0,04181 |
| AMTN | Homo sapiens amelotin (AMTN), mRNA. | 1,20618 | 8,40875 | 5,33886 | 0,00038 | 0,01132 |
| ITGA3 | Homo sapiens integrin, alpha 3 (antigen CD49C, alpha 3 subunit of VLA-3 receptor) (ITGA3), transcript variant a, mRNA. | 1,20068 | 11,43943 | 7,24345 | 0,00004 | 0,00403 |
| PGM3 | Homo sapiens phosphoglucomutase 3 (PGM3), mRNA. | 1,19749 | 9,08472 | 11,53060 | 0,00000 | 0,00065 |
| ATP2B4 | Homo sapiens ATPase, Ca++ transporting, plasma membrane 4 (ATP2B4), transcript variant 2, mRNA. | 1,19440 | 8,75075 | 6,39902 | 0,00010 | 0,00613 |
| TMEM44 | Homo sapiens transmembrane protein 44 (TMEM44), transcript variant 1, mRNA. | 1,19407 | 8,66880 | 4,47946 | 0,00132 | 0,02138 |
| PTPN12 | Homo sapiens protein tyrosine phosphatase, non-receptor type 12 (PTPN12), mRNA. | 1,18245 | 9,65186 | 4,95825 | 0,00065 | 0,01484 |
| KLF11 | PREDICTED: Homo sapiens Kruppel-like factor 11 (KLF11), mRNA. | 1,17743 | 8,86635 | 4,75651 | 0,00088 | 0,01716 |
| SH3KBP1 | Homo sapiens SH3-domain kinase binding protein 1 (SH3KBP1), transcript variant 1, mRNA. | 1,17509 | 9,47840 | 6,05681 | 0,00015 | 0,00726 |
| FXYD5 | Homo sapiens FXYD domain containing ion transport regulator 5 (FXYD5), transcript variant 2, mRNA. | 1,17317 | 12,42879 | 3,87818 | 0,00334 | 0,03594 |
| P4HA2 | Homo sapiens prolyl 4-hydroxylase, alpha polypeptide II (P4HA2), transcript variant 2, mRNA. | 1,17093 | 10,80563 | 5,07144 | 0,00056 | 0,01358 |
| GFPT1 | Homo sapiens glutamine-fructose-6-phosphate transaminase 1 (GFPT1), mRNA. | 1,17045 | 9,08266 | 12,07828 | 0,00000 | 0,00059 |
| TNFAIP3 | Homo sapiens tumor necrosis factor, alpha-induced protein 3 (TNFAIP3), mRNA. | 1,16207 | 8,50215 | 5,72299 | 0,00023 | 0,00886 |
| ATP2B4 | Homo sapiens ATPase, Ca++ transporting, plasma membrane 4 (ATP2B4), transcript variant 1, mRNA. | 1,15949 | 8,78542 | 5,94011 | 0,00017 | 0,00774 |
| PYGB | Homo sapiens phosphorylase, glycogen; brain (PYGB), mRNA. | 1,15891 | 9,90389 | 5,30940 | 0,00040 | 0,01152 |
| C13orf15 | Homo sapiens chromosome 13 open reading frame 15 (C13orf15), mRNA. | 1,15781 | 8,56369 | 6,41039 | 0,00009 | 0,00609 |
| GSK3B | Homo sapiens glycogen synthase kinase 3 beta (GSK3B), mRNA. | 1,14957 | 9,07713 | 4,11273 | 0,00231 | 0,02903 |
| BPGM | Homo sapiens 2,3-bisphosphoglycerate mutase (BPGM), transcript variant 1, mRNA. | 1,14509 | 8,48050 | 3,85750 | 0,00345 | 0,03676 |
| IRS2 | Homo sapiens insulin receptor substrate 2 (IRS2), mRNA. | 1,14255 | 9,04924 | 4,22448 | 0,00195 | 0,02637 |
| EHD1 | Homo sapiens EH-domain containing 1 (EHD1), mRNA. | 1,14217 | 9,60964 | 7,87186 | 0,00002 | 0,00322 |
| HAS3 | Homo sapiens hyaluronan synthase 3 (HAS3), transcript variant 1, mRNA. | 1,13896 | 9,97100 | 5,83616 | 0,00020 | 0,00817 |
| SH2D5 | PREDICTED: Homo sapiens SH2 domain containing 5 (SH2D5), mRNA. | 1,13879 | 8,27340 | 5,52925 | 0,00030 | 0,00999 |
| MTSS1 | Homo sapiens metastasis suppressor 1 (MTSS1), mRNA. | 1,13553 | 8,39421 | 6,57087 | 0,00008 | 0,00552 |
| GFPT1 | Homo sapiens glutamine-fructose-6-phosphate transaminase 1 (GFPT1), mRNA. | 1,13260 | 9,30569 | 13,42574 | 0,00000 | 0,00056 |
| NIPA1 | Homo sapiens non imprinted in Prader-Willi/Angelman syndrome 1 (NIPA1), mRNA. | 1,13130 | 8,60224 | 4,56600 | 0,00116 | 0,01991 |
| NAGK | Homo sapiens N-acetylglucosamine kinase (NAGK), mRNA. | 1,12914 | 8,92582 | 7,07049 | 0,00004 | 0,00430 |
| GLIPR1 | Homo sapiens GLI pathogenesis-related 1 (GLIPR1), mRNA. | 1,12497 | 8,35138 | 8,86350 | 0,00001 | 0,00198 |
| FAM129B | Homo sapiens family with sequence similarity 129, member B (FAM129B), transcript variant 1, mRNA. | 1,12493 | 11,62823 | 4,55315 | 0,00118 | 0,02014 |
| TGFB1I1 | Homo sapiens transforming growth factor beta 1 induced transcript 1 (TGFB1I1), transcript variant 2, mRNA. | 1,11795 | 8,34601 | 7,58791 | 0,00002 | 0,00344 |
| AKAP12 | Homo sapiens A kinase (PRKA) anchor protein (gravin) 12 (AKAP12), transcript variant 1, mRNA. | 1,11062 | 8,03588 | 4,93919 | 0,00067 | 0,01509 |
| LOC729768 | PREDICTED: Homo sapiens misc_RNA (LOC729768), miscRNA. | 1,10855 | 11,67259 | 8,47716 | 0,00001 | 0,00236 |
| JUP | Homo sapiens junction plakoglobin (JUP), transcript variant 1, mRNA. | 1,10530 | 11,84809 | 6,21327 | 0,00012 | 0,00683 |
| DGKA | Homo sapiens diacylglycerol kinase, alpha 80kDa (DGKA), transcript variant 4, mRNA. | 1,10498 | 8,86574 | 3,88542 | 0,00330 | 0,03567 |
| MCL1 | Homo sapiens myeloid cell leukemia sequence 1 (BCL2-related) (MCL1), transcript variant 1, mRNA. | 1,10309 | 9,72149 | 5,97636 | 0,00016 | 0,00756 |
| DNAJB2 | Homo sapiens DnaJ (Hsp40) homolog, subfamily B, member 2 (DNAJB2), transcript variant 2, mRNA. | 1,09707 | 10,65620 | 4,38482 | 0,00152 | 0,02312 |
| CHIC2 | Homo sapiens cysteine-rich hydrophobic domain 2 (CHIC2), mRNA. | 1,09556 | 9,64007 | 5,45833 | 0,00033 | 0,01048 |
| P4HA2 | Homo sapiens prolyl 4-hydroxylase, alpha polypeptide II (P4HA2), transcript variant 3, mRNA. | 1,09496 | 9,74620 | 4,41420 | 0,00146 | 0,02249 |
| SMOX | Homo sapiens spermine oxidase (SMOX), transcript variant 4, mRNA. | 1,09283 | 8,40689 | 4,69779 | 0,00096 | 0,01781 |
| KIAA0363 | PREDICTED: Homo sapiens KIAA0363 protein (KIAA0363), mRNA. | 1,09103 | 8,03107 | 8,51246 | 0,00001 | 0,00236 |
| LOC143666 | PREDICTED: Homo sapiens hypothetical protein LOC143666 (LOC143666), mRNA. | 1,09055 | 8,24694 | 7,32112 | 0,00003 | 0,00394 |
| F3 | Homo sapiens coagulation factor III (thromboplastin, tissue factor) (F3), mRNA. | 1,08828 | 10,18018 | 4,09261 | 0,00239 | 0,02960 |
| DAB2 | Homo sapiens disabled homolog 2, mitogen-responsive phosphoprotein (Drosophila) (DAB2), mRNA. | 1,08526 | 8,30106 | 4,03833 | 0,00260 | 0,03120 |
| GADD45A | Homo sapiens growth arrest and DNA-damage-inducible, alpha (GADD45A), mRNA. | 1,08282 | 9,89642 | 5,13282 | 0,00051 | 0,01299 |
| GADD45A | Homo sapiens growth arrest and DNA-damage-inducible, alpha (GADD45A), mRNA. | 1,08245 | 9,61294 | 4,66166 | 0,00101 | 0,01835 |
| KCNK6 | Homo sapiens potassium channel, subfamily K, member 6 (KCNK6), mRNA. | 1,07913 | 8,86877 | 8,36471 | 0,00001 | 0,00248 |
| PTPN12 | Homo sapiens protein tyrosine phosphatase, non-receptor type 12 (PTPN12), mRNA. | 1,07909 | 9,53512 | 4,98745 | 0,00063 | 0,01451 |
| ETV5 | Homo sapiens ets variant gene 5 (ets-related molecule) (ETV5), mRNA. | 1,07808 | 9,08014 | 6,00291 | 0,00016 | 0,00743 |
| ANXA2P1 | Homo sapiens annexin A2 pseudogene 1 (ANXA2P1) on chromosome 4. | 1,07698 | 9,00415 | 4,11205 | 0,00232 | 0,02904 |
| KLF13 | Homo sapiens Kruppel-like factor 13 (KLF13), mRNA. | 1,07631 | 9,20460 | 4,62934 | 0,00106 | 0,01894 |
| ARFGAP3 | Homo sapiens ADP-ribosylation factor GTPase activating protein 3 (ARFGAP3), mRNA. | 1,07325 | 9,22238 | 8,18679 | 0,00001 | 0,00270 |
| FOXD1 | Homo sapiens forkhead box D1 (FOXD1), mRNA. | 1,07029 | 8,72556 | 4,35322 | 0,00160 | 0,02376 |
| VIL2 | Homo sapiens villin 2 (ezrin) (VIL2), mRNA. | 1,06717 | 12,48288 | 10,57548 | 0,00000 | 0,00106 |
| KDELR3 | Homo sapiens KDEL (Lys-Asp-Glu-Leu) endoplasmic reticulum protein retention receptor 3 (KDELR3), transcript variant 2, mRNA. | 1,06585 | 8,93893 | 5,07936 | 0,00055 | 0,01351 |
| KLF9 | Homo sapiens Kruppel-like factor 9 (KLF9), mRNA. | 1,06344 | 8,54401 | 3,56617 | 0,00551 | 0,04860 |
| C9orf21 | Homo sapiens chromosome 9 open reading frame 21 (C9orf21), mRNA. | 1,06316 | 8,81666 | 4,07602 | 0,00245 | 0,03005 |
| PTPRE | Homo sapiens protein tyrosine phosphatase, receptor type, E (PTPRE), transcript variant 2, mRNA. | 1,05582 | 8,47368 | 7,52978 | 0,00003 | 0,00357 |
| GPSM1 | PREDICTED: Homo sapiens G-protein signalling modulator 1 (AGS3-like, C. elegans) (GPSM1), mRNA. | 1,05398 | 8,09773 | 6,77527 | 0,00006 | 0,00481 |
| XBP1 | Homo sapiens X-box binding protein 1 (XBP1), transcript variant 1, mRNA. | 1,05119 | 10,85507 | 6,71209 | 0,00007 | 0,00494 |
| ETS1 | Homo sapiens v-ets erythroblastosis virus E26 oncogene homolog 1 (avian) (ETS1), mRNA. | 1,04956 | 8,99819 | 7,16115 | 0,00004 | 0,00418 |
| STC2 | Homo sapiens stanniocalcin 2 (STC2), mRNA. | 1,04724 | 8,97259 | 6,04444 | 0,00015 | 0,00728 |
| GARS | Homo sapiens glycyl-tRNA synthetase (GARS), mRNA. | 1,04475 | 12,35241 | 6,15599 | 0,00013 | 0,00698 |
| GJB5 | Homo sapiens gap junction protein, beta 5, 31.1kDa (GJB5), mRNA. | 1,03807 | 8,25667 | 5,62583 | 0,00026 | 0,00933 |
| PDLIM7 | Homo sapiens PDZ and LIM domain 7 (enigma) (PDLIM7), transcript variant 4, mRNA. | 1,03675 | 9,34340 | 3,60627 | 0,00516 | 0,04665 |
| CD55 | Homo sapiens CD55 molecule, decay accelerating factor for complement (Cromer blood group) (CD55), mRNA. | 1,03486 | 8,11689 | 5,90172 | 0,00018 | 0,00782 |
| TMEM17 | Homo sapiens transmembrane protein 17 (TMEM17), mRNA. | 1,03384 | 10,83892 | 3,60052 | 0,00521 | 0,04691 |
| LOC100130154 | PREDICTED: Homo sapiens similar to thymosin, beta 10 (LOC100130154), mRNA. | 1,03371 | 9,00969 | 5,17151 | 0,00048 | 0,01265 |
| FAM129B | Homo sapiens family with sequence similarity 129, member B (FAM129B), transcript variant 2, mRNA. | 1,03309 | 9,67471 | 5,12636 | 0,00052 | 0,01303 |
| MAP1LC3B | Homo sapiens microtubule-associated protein 1 light chain 3 beta (MAP1LC3B), mRNA. | 1,03284 | 9,02125 | 7,43500 | 0,00003 | 0,00368 |
| TRIM8 | Homo sapiens tripartite motif-containing 8 (TRIM8), mRNA. | 1,03079 | 10,10435 | 5,56929 | 0,00028 | 0,00974 |
| FOXO3 | Homo sapiens forkhead box O3 (FOXO3), transcript variant 2, mRNA. | 1,02917 | 10,22782 | 5,47029 | 0,00032 | 0,01038 |
| CDK5R1 | Homo sapiens cyclin-dependent kinase 5, regulatory subunit 1 (p35) (CDK5R1), mRNA. | 1,02735 | 8,79765 | 4,16308 | 0,00214 | 0,02764 |
| CLDN12 | Homo sapiens claudin 12 (CLDN12), mRNA. | 1,02550 | 8,99458 | 3,69857 | 0,00445 | 0,04269 |
| TMEM154 | Homo sapiens transmembrane protein 154 (TMEM154), mRNA. | 1,02474 | 8,46083 | 6,49056 | 0,00009 | 0,00582 |
| FAM20C | Homo sapiens family with sequence similarity 20, member C (FAM20C), mRNA. | 1,02421 | 8,58271 | 3,61583 | 0,00509 | 0,04624 |
| OXSR1 | Homo sapiens oxidative-stress responsive 1 (OXSR1), mRNA. | 1,02309 | 10,04420 | 5,08331 | 0,00055 | 0,01344 |
| S100A11 | Homo sapiens S100 calcium binding protein A11 (S100A11), mRNA. | 1,02166 | 11,27151 | 4,23927 | 0,00190 | 0,02607 |
| XBP1 | Homo sapiens X-box binding protein 1 (XBP1), transcript variant 2, mRNA. | 1,02050 | 10,79362 | 5,35889 | 0,00037 | 0,01123 |
| YPEL5 | Homo sapiens yippee-like 5 (Drosophila) (YPEL5), mRNA. | 1,02003 | 9,74576 | 5,26616 | 0,00042 | 0,01181 |
| LOC730278 | PREDICTED: Homo sapiens hypothetical LOC730278 (LOC730278), mRNA. | 1,01638 | 12,56324 | 4,59381 | 0,00111 | 0,01952 |
| LRP10 | Homo sapiens low density lipoprotein receptor-related protein 10 (LRP10), mRNA. | 1,01460 | 10,12007 | 5,05855 | 0,00057 | 0,01372 |
| CLCF1 | Homo sapiens cardiotrophin-like cytokine factor 1 (CLCF1), transcript variant 1, mRNA. | 1,01386 | 8,47030 | 4,40909 | 0,00147 | 0,02260 |
| SLC38A2 | Homo sapiens solute carrier family 38, member 2 (SLC38A2), mRNA. | 1,01271 | 10,02929 | 5,47104 | 0,00032 | 0,01038 |
| TMEM16A | Homo sapiens transmembrane protein 16A (TMEM16A), mRNA. | 1,01242 | 9,30291 | 3,91844 | 0,00314 | 0,03455 |
| LOC399748 | PREDICTED: Homo sapiens misc_RNA (LOC399748), miscRNA. | 1,01042 | 11,09494 | 6,57374 | 0,00008 | 0,00552 |
| KDELR3 | Homo sapiens KDEL (Lys-Asp-Glu-Leu) endoplasmic reticulum protein retention receptor 3 (KDELR3), transcript variant 1, mRNA. | 1,01033 | 8,69308 | 7,21903 | 0,00004 | 0,00409 |
| SOCS1 | Homo sapiens suppressor of cytokine signaling 1 (SOCS1), mRNA. | 1,00348 | 7,93738 | 3,63064 | 0,00496 | 0,04552 |
| ADRB2 | Homo sapiens adrenergic, beta-2-, receptor, surface (ADRB2), mRNA. | 1,00079 | 8,23217 | 4,43905 | 0,00140 | 0,02210 |
| FSTL3 | Homo sapiens follistatin-like 3 (secreted glycoprotein) (FSTL3), mRNA. | 0,99469 | 9,07586 | 5,19991 | 0,00047 | 0,01245 |
| FAM107B | Homo sapiens family with sequence similarity 107, member B (FAM107B), mRNA. | 0,99278 | 8,75740 | 6,83951 | 0,00006 | 0,00479 |
| ANG | Homo sapiens angiogenin, ribonuclease, RNase A family, 5 (ANG), transcript variant 2, mRNA. | 0,99268 | 8,48752 | 4,01894 | 0,00268 | 0,03171 |
| MFSD10 | Homo sapiens major facilitator superfamily domain containing 10 (MFSD10), mRNA. | 0,99181 | 11,00010 | 6,25598 | 0,00011 | 0,00663 |
| DEDD2 | Homo sapiens death effector domain containing 2 (DEDD2), mRNA. | 0,99066 | 9,85401 | 6,02301 | 0,00015 | 0,00734 |
| LOC729231 | PREDICTED: Homo sapiens misc_RNA (LOC729231), miscRNA. | 0,98953 | 8,25277 | 6,05509 | 0,00015 | 0,00726 |
| LARP1B | Homo sapiens La ribonucleoprotein domain family, member 1B (LARP1B), transcript variant 3, mRNA. | 0,98654 | 9,12820 | 7,70640 | 0,00002 | 0,00332 |
| PRIC285 | Homo sapiens peroxisomal proliferator-activated receptor A interacting complex 285 (PRIC285), transcript variant 2, mRNA. | 0,98642 | 8,44304 | 5,82654 | 0,00020 | 0,00823 |
| DHRS7 | Homo sapiens dehydrogenase/reductase (SDR family) member 7 (DHRS7), mRNA. | 0,98408 | 10,28857 | 4,21459 | 0,00198 | 0,02647 |
| SLC39A4 | Homo sapiens solute carrier family 39 (zinc transporter), member 4 (SLC39A4), transcript variant 1, mRNA. | 0,98406 | 9,12213 | 3,97231 | 0,00288 | 0,03308 |
| ANO1 | Homo sapiens anoctamin 1, calcium activated chloride channel (ANO1), transcript variant 1, mRNA. | 0,98392 | 9,41878 | 3,89954 | 0,00323 | 0,03526 |
| PPP2R2C | Homo sapiens protein phosphatase 2 (formerly 2A), regulatory subunit B, gamma isoform (PPP2R2C), transcript variant 2, mRNA. | 0,98052 | 8,45303 | 3,83807 | 0,00356 | 0,03734 |
| DNASE1L1 | Homo sapiens deoxyribonuclease I-like 1 (DNASE1L1), transcript variant 4, mRNA. | 0,97533 | 8,55227 | 6,02714 | 0,00015 | 0,00732 |
| LOC392437 | PREDICTED: Homo sapiens misc_RNA (LOC392437), miscRNA. | 0,97412 | 11,96988 | 3,56108 | 0,00556 | 0,04878 |
| SPAG9 | Homo sapiens sperm associated antigen 9 (SPAG9), mRNA. | 0,97384 | 9,42320 | 4,60998 | 0,00109 | 0,01926 |
| FNBP1 | Homo sapiens formin binding protein 1 (FNBP1), mRNA. | 0,97005 | 8,58271 | 5,27034 | 0,00042 | 0,01179 |
| PRKCD | Homo sapiens protein kinase C, delta (PRKCD), transcript variant 1, mRNA. | 0,96849 | 8,95583 | 5,70878 | 0,00023 | 0,00897 |
| SARS | Homo sapiens seryl-tRNA synthetase (SARS), mRNA. | 0,96275 | 9,52940 | 5,83555 | 0,00020 | 0,00817 |
| PRNP | Homo sapiens prion protein (PRNP), transcript variant 2, mRNA. | 0,96253 | 11,91295 | 6,08530 | 0,00014 | 0,00713 |
| MBOAT2 | PREDICTED: Homo sapiens membrane bound O-acyltransferase domain containing 2 (MBOAT2), mRNA. | 0,96239 | 8,52694 | 3,94411 | 0,00301 | 0,03377 |
| CIB1 | Homo sapiens calcium and integrin binding 1 (calmyrin) (CIB1), mRNA. | 0,95713 | 11,23817 | 4,77466 | 0,00085 | 0,01693 |
| LOC642469 | PREDICTED: Homo sapiens misc_RNA (LOC642469), miscRNA. | 0,94892 | 8,61634 | 5,07203 | 0,00056 | 0,01358 |
| GOLGA3 | Homo sapiens golgi autoantigen, golgin subfamily a, 3 (GOLGA3), mRNA. | 0,94625 | 10,63137 | 5,91498 | 0,00018 | 0,00775 |
| DDEF2 | Homo sapiens development and differentiation enhancing factor 2 (DDEF2), mRNA. | 0,94592 | 9,73259 | 6,56429 | 0,00008 | 0,00552 |
| KRT6A | Homo sapiens keratin 6A (KRT6A), mRNA. | 0,94538 | 12,73982 | 4,27089 | 0,00181 | 0,02538 |
| PRNP | Homo sapiens prion protein (PRNP), transcript variant 3, mRNA. | 0,94336 | 12,65728 | 8,49974 | 0,00001 | 0,00236 |
| TMEM16A | Homo sapiens transmembrane protein 16A (TMEM16A), mRNA. | 0,94181 | 9,45198 | 3,83058 | 0,00360 | 0,03758 |
| SPAG9 | Homo sapiens sperm associated antigen 9 (SPAG9), mRNA. | 0,93963 | 9,16801 | 3,66120 | 0,00473 | 0,04428 |
| DYNLT3 | Homo sapiens dynein, light chain, Tctex-type 3 (DYNLT3), mRNA. | 0,93826 | 8,98217 | 7,51672 | 0,00003 | 0,00358 |
| SDC4 | Homo sapiens syndecan 4 (SDC4), mRNA. | 0,93550 | 10,21324 | 5,28722 | 0,00041 | 0,01165 |
| UAP1 | Homo sapiens UDP-N-acteylglucosamine pyrophosphorylase 1 (UAP1), mRNA. | 0,93382 | 9,17310 | 7,12369 | 0,00004 | 0,00422 |
| TUBB3 | Homo sapiens tubulin, beta 3 (TUBB3), mRNA. | 0,93045 | 10,29154 | 5,69741 | 0,00024 | 0,00902 |
| NRBP2 | Homo sapiens nuclear receptor binding protein 2 (NRBP2), mRNA. | 0,93005 | 8,49722 | 4,00455 | 0,00274 | 0,03212 |
| SQLE | Homo sapiens squalene epoxidase (SQLE), mRNA. | 0,92923 | 11,70579 | 3,63057 | 0,00497 | 0,04552 |
| NUMB | Homo sapiens numb homolog (Drosophila) (NUMB), transcript variant 3, mRNA. | 0,92724 | 9,59421 | 4,87107 | 0,00074 | 0,01585 |
| BAIAP2L1 | Homo sapiens BAI1-associated protein 2-like 1 (BAIAP2L1), mRNA. | 0,92294 | 11,09884 | 9,03801 | 0,00001 | 0,00190 |
| IFNGR2 | Homo sapiens interferon gamma receptor 2 (interferon gamma transducer 1) (IFNGR2), mRNA. | 0,92168 | 10,48820 | 5,99955 | 0,00016 | 0,00743 |
| MAD1L1 | Homo sapiens MAD1 mitotic arrest deficient-like 1 (yeast) (MAD1L1), transcript variant 2, mRNA. | 0,92130 | 8,48970 | 3,55802 | 0,00558 | 0,04886 |
| NET1 | Homo sapiens neuroepithelial cell transforming 1 (NET1), transcript variant 2, mRNA. | 0,92011 | 8,47914 | 4,82717 | 0,00079 | 0,01627 |
| TPM4 | Homo sapiens tropomyosin 4 (TPM4), mRNA. | 0,91949 | 9,67153 | 8,14427 | 0,00001 | 0,00277 |
| SLC38A1 | Homo sapiens solute carrier family 38, member 1 (SLC38A1), transcript variant 1, mRNA. | 0,91831 | 9,04950 | 6,96223 | 0,00005 | 0,00448 |
| DCBLD2 | Homo sapiens discoidin, CUB and LCCL domain containing 2 (DCBLD2), mRNA. | 0,91221 | 11,20632 | 3,77001 | 0,00397 | 0,03985 |
| RRAGC | Homo sapiens Ras-related GTP binding C (RRAGC), mRNA. | 0,91023 | 8,97200 | 4,97056 | 0,00064 | 0,01473 |
| VPS28 | Homo sapiens vacuolar protein sorting 28 homolog (S. cerevisiae) (VPS28), transcript variant 1, mRNA. | 0,90940 | 9,76918 | 4,07809 | 0,00244 | 0,02997 |
| TSC22D3 | Homo sapiens TSC22 domain family, member 3 (TSC22D3), transcript variant 2, mRNA. | 0,90831 | 8,44057 | 5,52384 | 0,00030 | 0,01002 |
| FRMD6 | Homo sapiens FERM domain containing 6 (FRMD6), mRNA. | 0,90505 | 9,68694 | 5,93737 | 0,00017 | 0,00774 |
| GPR172A | Homo sapiens G protein-coupled receptor 172A (GPR172A), mRNA. | 0,90498 | 11,09615 | 10,80451 | 0,00000 | 0,00091 |
| RABAC1 | Homo sapiens Rab acceptor 1 (prenylated) (RABAC1), mRNA. | 0,90445 | 9,99887 | 4,18797 | 0,00206 | 0,02707 |
| GRB7 | Homo sapiens growth factor receptor-bound protein 7 (GRB7), transcript variant 1, mRNA. | 0,90264 | 8,13189 | 4,74115 | 0,00090 | 0,01736 |
| SH3GLB1 | Homo sapiens SH3-domain GRB2-like endophilin B1 (SH3GLB1), mRNA. | 0,90254 | 10,25645 | 8,17201 | 0,00001 | 0,00272 |
| OCIAD2 | Homo sapiens OCIA domain containing 2 (OCIAD2), transcript variant 1, mRNA. | 0,90211 | 11,87166 | 6,46251 | 0,00009 | 0,00593 |
| LRRFIP1 | Homo sapiens leucine rich repeat (in FLII) interacting protein 1 (LRRFIP1), mRNA. | 0,90118 | 10,18050 | 4,46954 | 0,00134 | 0,02154 |
| RFTN1 | Homo sapiens raftlin, lipid raft linker 1 (RFTN1), mRNA. | 0,89992 | 8,74529 | 4,30234 | 0,00173 | 0,02477 |
| SERPINB8 | Homo sapiens serpin peptidase inhibitor, clade B (ovalbumin), member 8 (SERPINB8), transcript variant 2, mRNA. | 0,89188 | 8,08784 | 5,35039 | 0,00038 | 0,01129 |
| SPRR1B | Homo sapiens small proline-rich protein 1B (cornifin) (SPRR1B), mRNA. | 0,89074 | 7,75099 | 3,97240 | 0,00288 | 0,03308 |
| PODXL | Homo sapiens podocalyxin-like (PODXL), transcript variant 1, mRNA. | 0,88808 | 7,99237 | 6,43959 | 0,00009 | 0,00603 |
| HK2 | Homo sapiens hexokinase 2 (HK2), mRNA. | 0,88618 | 8,71941 | 4,22716 | 0,00194 | 0,02631 |
| ELL2 | Homo sapiens elongation factor, RNA polymerase II, 2 (ELL2), mRNA. | 0,88534 | 9,35539 | 4,70444 | 0,00095 | 0,01771 |
| EFNB1 | Homo sapiens ephrin-B1 (EFNB1), mRNA. | 0,88315 | 8,93581 | 4,50717 | 0,00127 | 0,02076 |
| LOC402221 | PREDICTED: Homo sapiens similar to actin alpha 1 skeletal muscle protein (LOC402221), mRNA. | 0,88304 | 10,15457 | 6,37427 | 0,00010 | 0,00614 |
| LOC401076 | PREDICTED: Homo sapiens misc_RNA (LOC401076), miscRNA. | 0,88287 | 10,04583 | 5,29486 | 0,00041 | 0,01161 |
| DCBLD2 | Homo sapiens discoidin, CUB and LCCL domain containing 2 (DCBLD2), mRNA. | 0,87749 | 10,62384 | 3,76386 | 0,00401 | 0,04005 |
| CTSL1 | Homo sapiens cathepsin L1 (CTSL1), transcript variant 1, mRNA. | 0,87705 | 10,18520 | 3,60947 | 0,00514 | 0,04648 |
| CEBPG | Homo sapiens CCAAT/enhancer binding protein (C/EBP), gamma (CEBPG), mRNA. | 0,87696 | 9,82925 | 4,91877 | 0,00069 | 0,01538 |
| ASAP2 | Homo sapiens ArfGAP with SH3 domain, ankyrin repeat and PH domain 2 (ASAP2), transcript variant 1, mRNA. | 0,87644 | 9,88919 | 7,28819 | 0,00003 | 0,00401 |
| CTNS | Homo sapiens cystinosis, nephropathic (CTNS), transcript variant 2, mRNA. | 0,87613 | 8,50912 | 3,59573 | 0,00525 | 0,04715 |
| ITGA6 | Homo sapiens integrin, alpha 6 (ITGA6), transcript variant 2, mRNA. | 0,87343 | 8,69724 | 4,58410 | 0,00113 | 0,01965 |
| CHMP1B | Homo sapiens chromatin modifying protein 1B (CHMP1B), mRNA. | 0,87247 | 11,64123 | 3,75413 | 0,00407 | 0,04044 |
| DNAJB9 | Homo sapiens DnaJ (Hsp40) homolog, subfamily B, member 9 (DNAJB9), mRNA. | 0,87208 | 8,19112 | 19,06914 | 0,00000 | 0,00016 |
| DUSP22 | PREDICTED: Homo sapiens dual specificity phosphatase 22 (DUSP22), mRNA. | 0,87158 | 9,91432 | 3,83152 | 0,00360 | 0,03756 |
| ZNF598 | Homo sapiens zinc finger protein 598 (ZNF598), mRNA. | 0,87078 | 10,33487 | 5,30632 | 0,00040 | 0,01154 |
| RTKN | Homo sapiens rhotekin (RTKN), transcript variant 2, mRNA. | 0,87057 | 10,35616 | 5,69990 | 0,00024 | 0,00902 |
| SLMO1 | Homo sapiens slowmo homolog 1 (Drosophila) (SLMO1), mRNA. | 0,86975 | 9,63682 | 4,04409 | 0,00257 | 0,03102 |
| LTBP4 | Homo sapiens latent transforming growth factor beta binding protein 4 (LTBP4), transcript variant 3, mRNA. | 0,86811 | 9,20831 | 5,82536 | 0,00020 | 0,00823 |
| MYO10 | Homo sapiens myosin X (MYO10), mRNA. | 0,86567 | 9,08375 | 4,84233 | 0,00077 | 0,01612 |
| TMED5 | Homo sapiens transmembrane emp24 protein transport domain containing 5 (TMED5), mRNA. | 0,86208 | 9,37643 | 7,12709 | 0,00004 | 0,00422 |
| RTCD1 | Homo sapiens RNA terminal phosphate cyclase domain 1 (RTCD1), mRNA. | 0,86169 | 9,58749 | 4,05654 | 0,00253 | 0,03065 |
| C3orf52 | Homo sapiens chromosome 3 open reading frame 52 (C3orf52), mRNA. | 0,86152 | 8,46641 | 3,82963 | 0,00361 | 0,03760 |
| CDA | Homo sapiens cytidine deaminase (CDA), mRNA. | 0,86149 | 8,00395 | 4,79955 | 0,00082 | 0,01665 |
| JUND | Homo sapiens jun D proto-oncogene (JUND), mRNA. | 0,86107 | 12,26168 | 4,88351 | 0,00073 | 0,01577 |
| ERCC1 | Homo sapiens excision repair cross-complementing rodent repair deficiency, complementation group 1 (includes overlapping antisense sequence) (ERCC1), transcript variant 2, mRNA. | 0,86095 | 9,79393 | 4,58258 | 0,00113 | 0,01965 |
| PPP1R13L | Homo sapiens protein phosphatase 1, regulatory (inhibitor) subunit 13 like (PPP1R13L), mRNA. | 0,85033 | 9,93709 | 5,78290 | 0,00021 | 0,00849 |
| GFPT2 | Homo sapiens glutamine-fructose-6-phosphate transaminase 2 (GFPT2), mRNA. | 0,85021 | 7,93457 | 4,59889 | 0,00111 | 0,01942 |
| KDELR2 | Homo sapiens KDEL (Lys-Asp-Glu-Leu) endoplasmic reticulum protein retention receptor 2 (KDELR2), transcript variant 1, mRNA. | 0,84891 | 11,08316 | 7,00338 | 0,00005 | 0,00442 |
| IGF2BP2 | Homo sapiens insulin-like growth factor 2 mRNA binding protein 2 (IGF2BP2), transcript variant 1, mRNA. | 0,84687 | 11,47040 | 7,06897 | 0,00004 | 0,00430 |
| PTPRE | Homo sapiens protein tyrosine phosphatase, receptor type, E (PTPRE), transcript variant 2, mRNA. | 0,84600 | 8,15255 | 7,00667 | 0,00005 | 0,00442 |
| PIK3CD | Homo sapiens phosphoinositide-3-kinase, catalytic, delta polypeptide (PIK3CD), mRNA. | 0,84410 | 8,05797 | 5,71693 | 0,00023 | 0,00890 |
| FAM127A | Homo sapiens family with sequence similarity 127, member A (FAM127A), mRNA. | 0,84355 | 9,91468 | 4,86845 | 0,00075 | 0,01585 |
| EHD4 | Homo sapiens EH-domain containing 4 (EHD4), mRNA. | 0,84318 | 9,21865 | 6,21065 | 0,00012 | 0,00683 |
| ZNF165 | Homo sapiens zinc finger protein 165 (ZNF165), mRNA. | 0,84267 | 8,60883 | 4,50908 | 0,00126 | 0,02075 |
| DFNA5 | Homo sapiens deafness, autosomal dominant 5 (DFNA5), transcript variant 1, mRNA. | 0,84243 | 10,72279 | 6,71218 | 0,00007 | 0,00494 |
| ANXA1 | Homo sapiens annexin A1 (ANXA1), mRNA. | 0,84074 | 12,82326 | 6,55385 | 0,00008 | 0,00555 |
| TPBG | Homo sapiens trophoblast glycoprotein (TPBG), mRNA. | 0,83996 | 8,95279 | 4,94885 | 0,00066 | 0,01498 |
| C19orf10 | Homo sapiens chromosome 19 open reading frame 10 (C19orf10), mRNA. | 0,83972 | 9,96635 | 4,16856 | 0,00212 | 0,02751 |
| MICAL1 | Homo sapiens microtubule associated monoxygenase, calponin and LIM domain containing 1 (MICAL1), mRNA. | 0,83891 | 8,02500 | 6,03194 | 0,00015 | 0,00732 |
| DAP | Homo sapiens death-associated protein (DAP), mRNA. | 0,83836 | 9,19919 | 4,63599 | 0,00105 | 0,01881 |
| SEC31A | Homo sapiens SEC31 homolog A (S. cerevisiae) (SEC31A), transcript variant 1, mRNA. | 0,83777 | 9,70885 | 6,89538 | 0,00005 | 0,00463 |
| ACOT7 | Homo sapiens acyl-CoA thioesterase 7 (ACOT7), transcript variant hBACHa, mRNA. | 0,83655 | 10,41909 | 4,76386 | 0,00087 | 0,01712 |
| COPA | Homo sapiens coatomer protein complex, subunit alpha (COPA), transcript variant 2, mRNA. | 0,83636 | 10,63691 | 6,41914 | 0,00009 | 0,00609 |
| ETS1 | Homo sapiens v-ets erythroblastosis virus E26 oncogene homolog 1 (avian) (ETS1), mRNA. | 0,83267 | 10,06964 | 5,72391 | 0,00023 | 0,00886 |
| ITGB5 | Homo sapiens integrin, beta 5 (ITGB5), mRNA. XM_944688 XM_944693 | 0,83208 | 9,95261 | 4,21642 | 0,00197 | 0,02642 |
| LHB | Homo sapiens luteinizing hormone beta polypeptide (LHB), mRNA. | 0,83165 | 7,95887 | 3,73788 | 0,00418 | 0,04114 |
| LIPG | Homo sapiens lipase, endothelial (LIPG), mRNA. | 0,82982 | 8,12418 | 5,94508 | 0,00017 | 0,00774 |
| CENTA1 | Homo sapiens centaurin, alpha 1 (CENTA1), mRNA. | 0,82870 | 10,27379 | 5,77269 | 0,00021 | 0,00853 |
| ST3GAL1 | Homo sapiens ST3 beta-galactoside alpha-2,3-sialyltransferase 1 (ST3GAL1), transcript variant 1, mRNA. | 0,82858 | 8,49919 | 3,68426 | 0,00455 | 0,04329 |
| UBR4 | Homo sapiens ubiquitin protein ligase E3 component n-recognin 4 (UBR4), mRNA. | 0,82841 | 8,80416 | 4,74033 | 0,00090 | 0,01737 |
| TNFAIP1 | Homo sapiens tumor necrosis factor, alpha-induced protein 1 (endothelial) (TNFAIP1), mRNA. | 0,82825 | 9,56333 | 8,11362 | 0,00001 | 0,00281 |
| RALGDS | Homo sapiens ral guanine nucleotide dissociation stimulator (RALGDS), transcript variant 1, mRNA. | 0,82765 | 9,57017 | 4,13849 | 0,00222 | 0,02831 |
| KDM5B | Homo sapiens lysine (K)-specific demethylase 5B (KDM5B), mRNA. | 0,82728 | 9,42062 | 4,34328 | 0,00162 | 0,02395 |
| LOC389787 | PREDICTED: Homo sapiens similar to Translationally-controlled tumor protein (TCTP) (p23) (Histamine-releasing factor) (HRF) (Fortilin) (LOC389787), mRNA. | 0,82687 | 11,05985 | 3,82513 | 0,00364 | 0,03768 |
| STK17B | Homo sapiens serine/threonine kinase 17b (STK17B), mRNA. | 0,82630 | 8,33317 | 4,31578 | 0,00169 | 0,02445 |
| EZR | Homo sapiens ezrin (EZR), transcript variant 1, mRNA. | 0,82472 | 12,97466 | 12,16734 | 0,00000 | 0,00059 |
| PTPRF | Homo sapiens protein tyrosine phosphatase, receptor type, F (PTPRF), transcript variant 1, mRNA. | 0,82371 | 11,06712 | 5,31839 | 0,00039 | 0,01140 |
| SLC35D2 | Homo sapiens solute carrier family 35, member D2 (SLC35D2), mRNA. | 0,82362 | 8,22961 | 5,45902 | 0,00033 | 0,01048 |
| EDEM1 | Homo sapiens ER degradation enhancer, mannosidase alpha-like 1 (EDEM1), mRNA. | 0,82308 | 8,63175 | 6,02825 | 0,00015 | 0,00732 |
| C19orf22 | Homo sapiens chromosome 19 open reading frame 22 (C19orf22), mRNA. | 0,82049 | 10,46801 | 6,73057 | 0,00006 | 0,00490 |
| B4GALT5 | Homo sapiens UDP-Gal:betaGlcNAc beta 1,4- galactosyltransferase, polypeptide 5 (B4GALT5), mRNA. | 0,82027 | 10,40225 | 4,14924 | 0,00219 | 0,02799 |
| CARS | Homo sapiens cysteinyl-tRNA synthetase (CARS), transcript variant 4, mRNA. | 0,81685 | 8,49047 | 6,08533 | 0,00014 | 0,00713 |
| M6PRBP1 | Homo sapiens mannose-6-phosphate receptor binding protein 1 (M6PRBP1), mRNA. | 0,81662 | 11,10015 | 3,58782 | 0,00532 | 0,04761 |
| UBAP1 | Homo sapiens ubiquitin associated protein 1 (UBAP1), mRNA. | 0,81626 | 10,17798 | 5,73263 | 0,00023 | 0,00881 |
| GDI1 | Homo sapiens GDP dissociation inhibitor 1 (GDI1), mRNA. | 0,81269 | 9,45703 | 7,80392 | 0,00002 | 0,00325 |
| FOXO3 | Homo sapiens forkhead box O3 (FOXO3), transcript variant 2, mRNA. | 0,81127 | 9,20802 | 3,63227 | 0,00495 | 0,04547 |
| MIDN | Homo sapiens midnolin (MIDN), mRNA. | 0,80596 | 9,26242 | 4,36002 | 0,00158 | 0,02365 |
| PRSS22 | Homo sapiens protease, serine, 22 (PRSS22), mRNA. | 0,80496 | 8,18130 | 7,80378 | 0,00002 | 0,00325 |
| CCNDBP1 | Homo sapiens cyclin D-type binding-protein 1 (CCNDBP1), transcript variant 2, mRNA. | 0,80253 | 10,22896 | 3,91910 | 0,00313 | 0,03455 |
| FNTA | Homo sapiens farnesyltransferase, CAAX box, alpha (FNTA), transcript variant 3, mRNA. | 0,80109 | 9,88444 | 12,58780 | 0,00000 | 0,00056 |
| JUP | Homo sapiens junction plakoglobin (JUP), transcript variant 2, mRNA. | 0,80069 | 8,57150 | 4,45156 | 0,00138 | 0,02184 |
| VEGFA | Homo sapiens vascular endothelial growth factor A (VEGFA), transcript variant 2, mRNA. | 0,79996 | 8,05727 | 6,05044 | 0,00015 | 0,00727 |
| LPP | Homo sapiens LIM domain containing preferred translocation partner in lipoma (LPP), mRNA. | 0,79948 | 10,38098 | 6,66289 | 0,00007 | 0,00507 |
| SPCS3 | Homo sapiens signal peptidase complex subunit 3 homolog (S. cerevisiae) (SPCS3), mRNA. | 0,79871 | 9,20556 | 6,19209 | 0,00012 | 0,00686 |
| ATF3 | Homo sapiens activating transcription factor 3 (ATF3), transcript variant 4, mRNA. | 0,79794 | 8,58906 | 5,38991 | 0,00036 | 0,01102 |
| YIF1A | Homo sapiens Yip1 interacting factor homolog A (S. cerevisiae) (YIF1A), mRNA. | 0,79761 | 10,36793 | 4,61538 | 0,00108 | 0,01921 |
| ARF4 | Homo sapiens ADP-ribosylation factor 4 (ARF4), mRNA. | 0,79468 | 11,99482 | 3,73136 | 0,00422 | 0,04144 |
| TNFRSF12A | Homo sapiens tumor necrosis factor receptor superfamily, member 12A (TNFRSF12A), mRNA. | 0,79445 | 11,50558 | 8,55686 | 0,00001 | 0,00236 |
| IFFO1 | Homo sapiens intermediate filament family orphan 1 (IFFO1), transcript variant 2, mRNA. | 0,79436 | 8,00425 | 4,87369 | 0,00074 | 0,01585 |
| LDLR | Homo sapiens low density lipoprotein receptor (familial hypercholesterolemia) (LDLR), mRNA. | 0,79239 | 11,73328 | 4,70833 | 0,00094 | 0,01769 |
| MACF1 | Homo sapiens microtubule-actin crosslinking factor 1 (MACF1), transcript variant 1, mRNA. | 0,78814 | 8,29706 | 4,63979 | 0,00104 | 0,01875 |
| ZNF281 | Homo sapiens zinc finger protein 281 (ZNF281), mRNA. | 0,78646 | 8,76897 | 9,12565 | 0,00001 | 0,00188 |
| METRNL | PREDICTED: Homo sapiens meteorin, glial cell differentiation regulator-like (METRNL), mRNA. | 0,78597 | 7,97583 | 6,03254 | 0,00015 | 0,00732 |
| SEL1L3 | Homo sapiens sel-1 suppressor of lin-12-like 3 (C. elegans) (SEL1L3), mRNA. | 0,78592 | 8,84712 | 4,43580 | 0,00141 | 0,02218 |
| SLC24A6 | Homo sapiens solute carrier family 24 (sodium/potassium/calcium exchanger), member 6 (SLC24A6), mRNA. | 0,78463 | 8,37720 | 4,32500 | 0,00167 | 0,02429 |
| TMEM120A | Homo sapiens transmembrane protein 120A (TMEM120A), mRNA. | 0,78446 | 8,35911 | 3,62830 | 0,00498 | 0,04563 |
| ACOT7 | Homo sapiens acyl-CoA thioesterase 7 (ACOT7), transcript variant hBACHb, mRNA. | 0,78413 | 11,09948 | 3,96029 | 0,00294 | 0,03340 |
| BCL2L1 | Homo sapiens BCL2-like 1 (BCL2L1), nuclear gene encoding mitochondrial protein, transcript variant 1, mRNA. | 0,78411 | 8,31739 | 6,41167 | 0,00009 | 0,00609 |
| HK1 | Homo sapiens hexokinase 1 (HK1), nuclear gene encoding mitochondrial protein, transcript variant 5, mRNA. | 0,78310 | 11,54032 | 6,55534 | 0,00008 | 0,00555 |
| TUBB6 | PREDICTED: Homo sapiens tubulin, beta 6 (TUBB6), mRNA. | 0,78199 | 11,32630 | 3,55523 | 0,00561 | 0,04898 |
| ZFAND2A | Homo sapiens zinc finger, AN1-type domain 2A (ZFAND2A), mRNA. | 0,78169 | 9,25343 | 6,29021 | 0,00011 | 0,00653 |
| WSB1 | Homo sapiens WD repeat and SOCS box-containing 1 (WSB1), transcript variant 2, mRNA. | 0,77911 | 9,86702 | 3,85969 | 0,00344 | 0,03669 |
| VPS37B | Homo sapiens vacuolar protein sorting 37 homolog B (S. cerevisiae) (VPS37B), mRNA. | 0,77615 | 9,62231 | 3,79227 | 0,00383 | 0,03897 |
| CAB39 | Homo sapiens calcium binding protein 39 (CAB39), mRNA. | 0,77593 | 11,52787 | 5,03977 | 0,00058 | 0,01395 |
| MGC87042 | PREDICTED: Homo sapiens similar to Six transmembrane epithelial antigen of prostate (MGC87042), mRNA. | 0,77523 | 9,85234 | 3,89394 | 0,00326 | 0,03547 |
| LOC654103 | PREDICTED: Homo sapiens similar to solute carrier family 25, member 37 (LOC654103), mRNA. | 0,77509 | 8,93990 | 6,99669 | 0,00005 | 0,00442 |
| SPTLC1 | Homo sapiens serine palmitoyltransferase, long chain base subunit 1 (SPTLC1), transcript variant 2, mRNA. | 0,77229 | 8,14051 | 3,94339 | 0,00302 | 0,03377 |
| LOC644936 | Homo sapiens cytoplasmic beta-actin pseudogene (LOC644936), non-coding RNA. | 0,77187 | 12,42548 | 4,40195 | 0,00149 | 0,02275 |
| PPARD | Homo sapiens peroxisome proliferative activated receptor, delta (PPARD), transcript variant 1, mRNA. | 0,77067 | 8,09840 | 3,80341 | 0,00376 | 0,03857 |
| EIF1 | Homo sapiens eukaryotic translation initiation factor 1 (EIF1), mRNA. | 0,77012 | 12,14876 | 6,48998 | 0,00009 | 0,00582 |
| PI3 | Homo sapiens peptidase inhibitor 3, skin-derived (SKALP) (PI3), mRNA. | 0,76862 | 7,76866 | 4,59683 | 0,00111 | 0,01945 |
| ASAP2 | Homo sapiens ArfGAP with SH3 domain, ankyrin repeat and PH domain 2 (ASAP2), transcript variant 1, mRNA. | 0,76826 | 8,84804 | 5,49734 | 0,00031 | 0,01018 |
| LPAR5 | Homo sapiens lysophosphatidic acid receptor 5 (LPAR5), mRNA. | 0,76587 | 8,25625 | 4,43056 | 0,00142 | 0,02224 |
| SERPINB5 | Homo sapiens serpin peptidase inhibitor, clade B (ovalbumin), member 5 (SERPINB5), mRNA. | 0,76376 | 11,42710 | 3,90020 | 0,00323 | 0,03524 |
| DDIT3 | Homo sapiens DNA-damage-inducible transcript 3 (DDIT3), mRNA. | 0,76275 | 8,13181 | 5,45107 | 0,00033 | 0,01050 |
| FRMD6 | Homo sapiens FERM domain containing 6 (FRMD6), transcript variant 2, mRNA. | 0,76257 | 8,96383 | 4,31571 | 0,00169 | 0,02445 |
| MED8 | Homo sapiens mediator of RNA polymerase II transcription, subunit 8 homolog (S. cerevisiae) (MED8), transcript variant 5, mRNA. | 0,75912 | 8,88146 | 8,89052 | 0,00001 | 0,00198 |
| CSNK1D | Homo sapiens casein kinase 1, delta (CSNK1D), transcript variant 1, mRNA. | 0,75688 | 9,45771 | 4,94929 | 0,00066 | 0,01498 |
| LOC200030 | Homo sapiens neuroblastoma breakpoint family, member 11-like (LOC200030), mRNA. | 0,75499 | 9,27696 | 4,37185 | 0,00155 | 0,02336 |
| OSTC | Homo sapiens oligosaccharyltransferase complex subunit (OSTC), mRNA. | 0,75354 | 9,66256 | 5,34546 | 0,00038 | 0,01130 |
| DLGAP4 | Homo sapiens discs, large (Drosophila) homolog-associated protein 4 (DLGAP4), transcript variant 3, mRNA. | 0,75281 | 8,86462 | 4,01757 | 0,00268 | 0,03172 |
| FAM89B | Homo sapiens family with sequence similarity 89, member B (FAM89B), transcript variant 3, mRNA. | 0,75249 | 8,58505 | 5,44751 | 0,00033 | 0,01051 |
| AGPAT2 | Homo sapiens 1-acylglycerol-3-phosphate O-acyltransferase 2 (lysophosphatidic acid acyltransferase, beta) (AGPAT2), transcript variant 1, mRNA. | 0,75087 | 8,45523 | 5,79533 | 0,00021 | 0,00838 |
| AXIN1 | Homo sapiens axin 1 (AXIN1), transcript variant 2, mRNA. | 0,74970 | 9,26409 | 6,43167 | 0,00009 | 0,00606 |
| ITGB5 | Homo sapiens integrin, beta 5 (ITGB5), mRNA. XM_944688 XM_944693 | 0,74550 | 10,53567 | 3,95639 | 0,00295 | 0,03346 |
| C9orf30 | Homo sapiens chromosome 9 open reading frame 30 (C9orf30), mRNA. | 0,74390 | 10,39788 | 5,22834 | 0,00045 | 0,01212 |
| PQLC3 | Homo sapiens PQ loop repeat containing 3 (PQLC3), mRNA. | 0,74316 | 8,84001 | 4,58452 | 0,00113 | 0,01965 |
| FKSG30 | Homo sapiens actin-like protein (FKSG30), mRNA. | 0,74315 | 12,85456 | 7,99560 | 0,00002 | 0,00300 |
| MGEA5 | Homo sapiens meningioma expressed antigen 5 (hyaluronidase) (MGEA5), mRNA. | 0,74287 | 10,21906 | 4,72292 | 0,00092 | 0,01757 |
| STK10 | Homo sapiens serine/threonine kinase 10 (STK10), mRNA. | 0,74246 | 8,06624 | 4,66199 | 0,00101 | 0,01835 |
| ACBD3 | Homo sapiens acyl-Coenzyme A binding domain containing 3 (ACBD3), mRNA. | 0,74095 | 9,80791 | 5,19857 | 0,00047 | 0,01245 |
| ATF4 | Homo sapiens activating transcription factor 4 (tax-responsive enhancer element B67) (ATF4), transcript variant 2, mRNA. | 0,74003 | 9,15814 | 3,94350 | 0,00301 | 0,03377 |
| SEC31A | Homo sapiens SEC31 homolog A (S. cerevisiae) (SEC31A), transcript variant 1, mRNA. | 0,73945 | 8,86463 | 5,63317 | 0,00026 | 0,00933 |
| TPM4 | Homo sapiens tropomyosin 4 (TPM4), mRNA. | 0,73934 | 9,55688 | 9,45346 | 0,00000 | 0,00178 |
| C2orf24 | Homo sapiens chromosome 2 open reading frame 24 (C2orf24), mRNA. | 0,73902 | 8,53651 | 4,93296 | 0,00068 | 0,01517 |
| RTTN | Homo sapiens rotatin (RTTN), mRNA. | 0,73790 | 8,47050 | 4,03586 | 0,00261 | 0,03126 |
| ARHGAP21 | Homo sapiens Rho GTPase activating protein 21 (ARHGAP21), mRNA. | 0,73705 | 9,45676 | 6,16028 | 0,00013 | 0,00695 |
| ZDHHC9 | Homo sapiens zinc finger, DHHC-type containing 9 (ZDHHC9), transcript variant 1, mRNA. | 0,73682 | 9,70124 | 6,10809 | 0,00014 | 0,00709 |
| UGCG | Homo sapiens UDP-glucose ceramide glucosyltransferase (UGCG), mRNA. | 0,73664 | 8,79636 | 3,54882 | 0,00567 | 0,04935 |
| MFSD6 | Homo sapiens major facilitator superfamily domain containing 6 (MFSD6), mRNA. | 0,73446 | 8,48314 | 5,77218 | 0,00021 | 0,00853 |
| LOC100131336 | PREDICTED: Homo sapiens misc_RNA (LOC100131336), miscRNA. | 0,73304 | 10,04580 | 4,25568 | 0,00186 | 0,02572 |
| SPATA2L | Homo sapiens spermatogenesis associated 2-like (SPATA2L), mRNA. | 0,73256 | 8,31569 | 5,93607 | 0,00017 | 0,00774 |
| DUSP6 | Homo sapiens dual specificity phosphatase 6 (DUSP6), transcript variant 1, mRNA. | 0,73244 | 8,01367 | 3,74866 | 0,00411 | 0,04061 |
| DBN1 | Homo sapiens drebrin 1 (DBN1), transcript variant 1, mRNA. | 0,73078 | 8,95688 | 6,19923 | 0,00012 | 0,00686 |
| CYP27B1 | Homo sapiens cytochrome P450, family 27, subfamily B, polypeptide 1 (CYP27B1), nuclear gene encoding mitochondrial protein, mRNA. | 0,73003 | 7,89524 | 3,79776 | 0,00380 | 0,03875 |
| DOCK6 | Homo sapiens dedicator of cytokinesis 6 (DOCK6), mRNA. | 0,72993 | 8,10671 | 4,98471 | 0,00063 | 0,01453 |
| TFAP2C | Homo sapiens transcription factor AP-2 gamma (activating enhancer binding protein 2 gamma) (TFAP2C), mRNA. | 0,72723 | 9,59172 | 4,51836 | 0,00125 | 0,02061 |
| BNIP3L | Homo sapiens BCL2/adenovirus E1B 19kDa interacting protein 3-like (BNIP3L), mRNA. | 0,72694 | 8,99688 | 3,76953 | 0,00397 | 0,03985 |
| FERMT2 | Homo sapiens fermitin family homolog 2 (Drosophila) (FERMT2), mRNA. | 0,72665 | 8,62430 | 5,38183 | 0,00036 | 0,01105 |
| LYN | Homo sapiens v-yes-1 Yamaguchi sarcoma viral related oncogene homolog (LYN), mRNA. | 0,72582 | 8,86924 | 7,12660 | 0,00004 | 0,00422 |
| C14orf173 | Homo sapiens chromosome 14 open reading frame 173 (C14orf173), transcript variant 2, mRNA. | 0,72579 | 9,55233 | 5,12430 | 0,00052 | 0,01303 |
| METRNL | PREDICTED: Homo sapiens meteorin, glial cell differentiation regulator-like (METRNL), mRNA. | 0,72469 | 7,85326 | 4,98058 | 0,00063 | 0,01457 |
| ARID3A | Homo sapiens AT rich interactive domain 3A (BRIGHT-like) (ARID3A), mRNA. | 0,72454 | 8,28165 | 4,58350 | 0,00113 | 0,01965 |
| IQGAP1 | Homo sapiens IQ motif containing GTPase activating protein 1 (IQGAP1), mRNA. | 0,72391 | 8,94837 | 5,16466 | 0,00049 | 0,01269 |
| OSBPL10 | Homo sapiens oxysterol binding protein-like 10 (OSBPL10), mRNA. | 0,72338 | 9,22043 | 3,93367 | 0,00306 | 0,03408 |
| PTPRF | Homo sapiens protein tyrosine phosphatase, receptor type, F (PTPRF), transcript variant 2, mRNA. | 0,72329 | 10,05496 | 4,15295 | 0,00217 | 0,02789 |
| TRAM1 | Homo sapiens translocation associated membrane protein 1 (TRAM1), mRNA. | 0,72191 | 11,65434 | 14,76201 | 0,00000 | 0,00056 |
| BRI3P1 | PREDICTED: Homo sapiens misc_RNA (BRI3P1), miscRNA. | 0,72059 | 9,09476 | 4,33982 | 0,00163 | 0,02401 |
| SOCS2 | Homo sapiens suppressor of cytokine signaling 2 (SOCS2), mRNA. | 0,72047 | 10,14411 | 3,68002 | 0,00459 | 0,04345 |
| RASA1 | Homo sapiens RAS p21 protein activator (GTPase activating protein) 1 (RASA1), transcript variant 1, mRNA. | 0,71862 | 9,64058 | 5,33522 | 0,00039 | 0,01132 |
| FMNL2 | Homo sapiens formin-like 2 (FMNL2), mRNA. | 0,71826 | 8,46564 | 5,20751 | 0,00046 | 0,01238 |
| LZTR1 | Homo sapiens leucine-zipper-like transcription regulator 1 (LZTR1), mRNA. | 0,71670 | 9,91542 | 4,22104 | 0,00196 | 0,02637 |
| AGPAT2 | Homo sapiens 1-acylglycerol-3-phosphate O-acyltransferase 2 (lysophosphatidic acid acyltransferase, beta) (AGPAT2), transcript variant 1, mRNA. | 0,71645 | 8,54708 | 4,93195 | 0,00068 | 0,01517 |
| PDLIM7 | Homo sapiens PDZ and LIM domain 7 (enigma) (PDLIM7), transcript variant 1, mRNA. | 0,71347 | 8,59182 | 4,05251 | 0,00254 | 0,03074 |
| FAM177A1 | Homo sapiens family with sequence similarity 177, member A1 (FAM177A1), transcript variant 1, mRNA. | 0,71168 | 8,29032 | 4,32929 | 0,00166 | 0,02422 |
| LOC730820 | PREDICTED: Homo sapiens similar to nuclear receptor binding factor 2 (LOC730820), mRNA. | 0,71163 | 8,90271 | 3,57025 | 0,00547 | 0,04844 |
| JOSD1 | Homo sapiens Josephin domain containing 1 (JOSD1), mRNA. | 0,71141 | 8,76231 | 6,73752 | 0,00006 | 0,00489 |
| ATF4 | Homo sapiens activating transcription factor 4 (tax-responsive enhancer element B67) (ATF4), transcript variant 1, mRNA. | 0,71098 | 12,56939 | 4,40965 | 0,00147 | 0,02259 |
| PDIA5 | Homo sapiens protein disulfide isomerase family A, member 5 (PDIA5), mRNA. | 0,70864 | 8,85485 | 4,34547 | 0,00162 | 0,02394 |
| WBP5 | Homo sapiens WW domain binding protein 5 (WBP5), transcript variant 4, mRNA. | 0,70735 | 9,47438 | 4,19124 | 0,00205 | 0,02698 |
| SERP1 | Homo sapiens stress-associated endoplasmic reticulum protein 1 (SERP1), mRNA. | 0,70724 | 9,08245 | 4,65457 | 0,00102 | 0,01844 |
| OCIAD2 | Homo sapiens OCIA domain containing 2 (OCIAD2), transcript variant 2, mRNA. | 0,70703 | 10,02682 | 4,32509 | 0,00167 | 0,02429 |
| RTCD1 | Homo sapiens RNA terminal phosphate cyclase domain 1 (RTCD1), mRNA. | 0,70671 | 9,42631 | 4,40554 | 0,00148 | 0,02268 |
| BCAP29 | Homo sapiens B-cell receptor-associated protein 29 (BCAP29), transcript variant 1, mRNA. | 0,70622 | 8,57156 | 3,96766 | 0,00290 | 0,03314 |
| RTN2 | Homo sapiens reticulon 2 (RTN2), transcript variant 3, mRNA. | 0,70619 | 7,90694 | 5,68146 | 0,00024 | 0,00908 |
| RNF216L | Homo sapiens ring finger protein 216-like (RNF216L), transcript variant 2, non-coding RNA. | 0,70481 | 9,04631 | 5,38551 | 0,00036 | 0,01102 |
| OSBPL2 | Homo sapiens oxysterol binding protein-like 2 (OSBPL2), transcript variant 2, mRNA. | 0,70444 | 8,84749 | 4,71484 | 0,00093 | 0,01764 |
| ZYX | Homo sapiens zyxin (ZYX), transcript variant 1, mRNA. | 0,70056 | 9,80776 | 4,61133 | 0,00109 | 0,01926 |
| DSG3 | Homo sapiens desmoglein 3 (pemphigus vulgaris antigen) (DSG3), mRNA. | 0,70050 | 9,02135 | 4,01499 | 0,00269 | 0,03180 |
| RAB5B | Homo sapiens RAB5B, member RAS oncogene family (RAB5B), mRNA. | 0,70008 | 9,94662 | 4,86567 | 0,00075 | 0,01588 |
| IRF6 | Homo sapiens interferon regulatory factor 6 (IRF6), mRNA. | 0,69896 | 8,35277 | 4,20636 | 0,00200 | 0,02659 |
| SLC33A1 | Homo sapiens solute carrier family 33 (acetyl-CoA transporter), member 1 (SLC33A1), mRNA. | 0,69752 | 8,82007 | 7,18501 | 0,00004 | 0,00415 |
| YIPF4 | Homo sapiens Yip1 domain family, member 4 (YIPF4), mRNA. | 0,69642 | 9,30003 | 4,82429 | 0,00079 | 0,01631 |
| SQSTM1 | Homo sapiens sequestosome 1 (SQSTM1), mRNA. | 0,69621 | 12,84273 | 5,01068 | 0,00061 | 0,01424 |
| SERPINB8 | Homo sapiens serpin peptidase inhibitor, clade B (ovalbumin), member 8 (SERPINB8), transcript variant 1, mRNA. | 0,69610 | 8,03336 | 4,39289 | 0,00151 | 0,02295 |
| SLC25A37 | Homo sapiens solute carrier family 25, member 37 (SLC25A37), nuclear gene encoding mitochondrial protein, mRNA. | 0,69565 | 8,65724 | 7,18439 | 0,00004 | 0,00415 |
| EGFR | Homo sapiens epidermal growth factor receptor (erythroblastic leukemia viral (v-erb-b) oncogene homolog, avian) (EGFR), transcript variant 1, mRNA. | 0,69406 | 12,17579 | 5,36276 | 0,00037 | 0,01119 |
| CYLN2 | Homo sapiens cytoplasmic linker 2 (CYLN2), transcript variant 2, mRNA. | 0,69372 | 8,67364 | 4,09993 | 0,00236 | 0,02940 |
| MYO5A | Homo sapiens myosin VA (heavy chain 12, myoxin) (MYO5A), mRNA. | 0,69320 | 8,16091 | 3,69971 | 0,00444 | 0,04264 |
| PJA2 | Homo sapiens praja 2, RING-H2 motif containing (PJA2), mRNA. | 0,69266 | 9,91034 | 5,54747 | 0,00029 | 0,00992 |
| CLIP2 | Homo sapiens CAP-GLY domain containing linker protein 2 (CLIP2), transcript variant 2, mRNA. | 0,69204 | 8,58171 | 4,64975 | 0,00103 | 0,01853 |
| RNF149 | Homo sapiens ring finger protein 149 (RNF149), mRNA. | 0,68992 | 9,91925 | 5,20853 | 0,00046 | 0,01238 |
| POLD4 | Homo sapiens polymerase (DNA-directed), delta 4 (POLD4), mRNA. | 0,68968 | 8,32951 | 4,16814 | 0,00212 | 0,02751 |
| NUMB | Homo sapiens numb homolog (Drosophila) (NUMB), transcript variant 2, mRNA. | 0,68840 | 8,81290 | 4,65614 | 0,00102 | 0,01843 |
| CCDC50 | Homo sapiens coiled-coil domain containing 50 (CCDC50), transcript variant 1, mRNA. | 0,68717 | 10,75177 | 8,22818 | 0,00001 | 0,00262 |
| RHBDF1 | Homo sapiens rhomboid 5 homolog 1 (Drosophila) (RHBDF1), mRNA. | 0,68625 | 9,61895 | 3,56947 | 0,00548 | 0,04848 |
| LUZP1 | Homo sapiens leucine zipper protein 1 (LUZP1), mRNA. | 0,68572 | 10,11557 | 4,25512 | 0,00186 | 0,02573 |
| PPP4R1 | Homo sapiens protein phosphatase 4, regulatory subunit 1 (PPP4R1), transcript variant 2, mRNA. | 0,68441 | 11,08824 | 3,82013 | 0,00366 | 0,03789 |
| FGD6 | Homo sapiens FYVE, RhoGEF and PH domain containing 6 (FGD6), mRNA. | 0,68059 | 8,11964 | 5,63824 | 0,00026 | 0,00933 |
| WDR45 | Homo sapiens WD repeat domain 45 (WDR45), transcript variant 1, mRNA. | 0,68048 | 8,09291 | 5,30754 | 0,00040 | 0,01153 |
| SERINC1 | Homo sapiens serine incorporator 1 (SERINC1), mRNA. | 0,67793 | 8,72097 | 4,62842 | 0,00106 | 0,01894 |
| TAX1BP1 | Homo sapiens Tax1 (human T-cell leukemia virus type I) binding protein 1 (TAX1BP1), transcript variant 2, mRNA. | 0,67789 | 11,11378 | 4,69169 | 0,00096 | 0,01788 |
| FEZ2 | Homo sapiens fasciculation and elongation protein zeta 2 (zygin II) (FEZ2), transcript variant 1, mRNA. | 0,67675 | 10,77073 | 7,62212 | 0,00002 | 0,00337 |
| BCAP29 | Homo sapiens B-cell receptor-associated protein 29 (BCAP29), transcript variant 2, mRNA. | 0,67651 | 8,60147 | 3,96513 | 0,00291 | 0,03324 |
| NGEF | Homo sapiens neuronal guanine nucleotide exchange factor (NGEF), mRNA. | 0,67581 | 7,99059 | 4,92819 | 0,00068 | 0,01522 |
| GSTO1 | Homo sapiens glutathione S-transferase omega 1 (GSTO1), mRNA. | 0,67569 | 12,59097 | 5,10985 | 0,00053 | 0,01316 |
| ULBP2 | Homo sapiens UL16 binding protein 2 (ULBP2), mRNA. | 0,67501 | 8,89694 | 4,19904 | 0,00202 | 0,02678 |
| PIGA | Homo sapiens phosphatidylinositol glycan anchor biosynthesis, class A (PIGA), transcript variant 3, mRNA. | 0,67437 | 8,60222 | 5,05126 | 0,00057 | 0,01379 |
| MAPKAP1 | Homo sapiens mitogen-activated protein kinase associated protein 1 (MAPKAP1), transcript variant 6, mRNA. | 0,67195 | 8,33042 | 5,49775 | 0,00031 | 0,01018 |
| C6orf141 | Homo sapiens chromosome 6 open reading frame 141 (C6orf141), mRNA. | 0,67027 | 8,29133 | 5,49088 | 0,00031 | 0,01023 |
| EFTUD1 | Homo sapiens elongation factor Tu GTP binding domain containing 1 (EFTUD1), transcript variant 1, mRNA. | 0,66821 | 8,18476 | 4,84566 | 0,00077 | 0,01612 |
| GMPPA | Homo sapiens GDP-mannose pyrophosphorylase A (GMPPA), transcript variant 2, mRNA. | 0,66526 | 9,70775 | 6,77535 | 0,00006 | 0,00481 |
| ANXA2 | Homo sapiens annexin A2 (ANXA2), transcript variant 2, mRNA. | 0,66305 | 8,52158 | 4,20474 | 0,00201 | 0,02662 |
| SERINC3 | Homo sapiens serine incorporator 3 (SERINC3), transcript variant 1, mRNA. | 0,66297 | 9,49036 | 5,16945 | 0,00049 | 0,01265 |
| UGDH | Homo sapiens UDP-glucose dehydrogenase (UGDH), mRNA. | 0,66270 | 9,37240 | 7,85247 | 0,00002 | 0,00322 |
| IL32 | Homo sapiens interleukin 32 (IL32), transcript variant 4, mRNA. | 0,66183 | 7,81673 | 6,17573 | 0,00013 | 0,00689 |
| SBF1 | Homo sapiens SET binding factor 1 (SBF1), transcript variant 1, mRNA. | 0,66154 | 8,45595 | 4,19408 | 0,00204 | 0,02690 |
| STARD10 | Homo sapiens StAR-related lipid transfer (START) domain containing 10 (STARD10), mRNA. | 0,66149 | 9,04299 | 4,14987 | 0,00218 | 0,02798 |
| EDNRA | Homo sapiens endothelin receptor type A (EDNRA), mRNA. | 0,66128 | 7,78730 | 3,97199 | 0,00288 | 0,03308 |
| C2orf30 | Homo sapiens chromosome 2 open reading frame 30 (C2orf30), mRNA. | 0,65804 | 9,56061 | 10,35273 | 0,00000 | 0,00115 |
| RNF44 | Homo sapiens ring finger protein 44 (RNF44), mRNA. | 0,65660 | 8,57220 | 5,26388 | 0,00043 | 0,01182 |
| ZCCHC9 | Homo sapiens zinc finger, CCHC domain containing 9 (ZCCHC9), mRNA. | 0,65590 | 9,51001 | 5,05766 | 0,00057 | 0,01372 |
| ANXA2 | Homo sapiens annexin A2 (ANXA2), transcript variant 1, mRNA. | 0,65503 | 13,47723 | 7,10817 | 0,00004 | 0,00422 |
| CHMP5 | Homo sapiens chromatin modifying protein 5 (CHMP5), mRNA. | 0,65441 | 10,75023 | 6,97970 | 0,00005 | 0,00446 |
| SMAGP | Homo sapiens small cell adhesion glycoprotein (SMAGP), transcript variant 2, mRNA. | 0,65313 | 8,81313 | 4,59027 | 0,00112 | 0,01957 |
| PAOX | Homo sapiens polyamine oxidase (exo-N4-amino) (PAOX), transcript variant 5, mRNA. | 0,65018 | 7,98876 | 5,31386 | 0,00040 | 0,01146 |
| TPD52L2 | Homo sapiens tumor protein D52-like 2 (TPD52L2), transcript variant 5, mRNA. | 0,64977 | 10,67983 | 5,88045 | 0,00019 | 0,00789 |
| ILK | Homo sapiens integrin-linked kinase (ILK), transcript variant 1, mRNA. | 0,64932 | 10,98352 | 4,40726 | 0,00147 | 0,02265 |
| COPB1 | Homo sapiens coatomer protein complex, subunit beta 1 (COPB1), mRNA. | 0,64906 | 10,53665 | 5,26503 | 0,00042 | 0,01182 |
| FNBP1L | Homo sapiens formin binding protein 1-like (FNBP1L), transcript variant 1, mRNA. | 0,64832 | 8,46408 | 4,61514 | 0,00108 | 0,01921 |
| CCDC93 | Homo sapiens coiled-coil domain containing 93 (CCDC93), mRNA. | 0,64823 | 8,59956 | 4,72331 | 0,00092 | 0,01757 |
| OSBP | Homo sapiens oxysterol binding protein (OSBP), mRNA. | 0,64579 | 9,70857 | 4,98044 | 0,00063 | 0,01457 |
| PROCR | Homo sapiens protein C receptor, endothelial (EPCR) (PROCR), mRNA. | 0,64474 | 8,80025 | 3,67765 | 0,00460 | 0,04354 |
| ARL8B | Homo sapiens ADP-ribosylation factor-like 8B (ARL8B), mRNA. | 0,64410 | 9,64687 | 6,13419 | 0,00013 | 0,00704 |
| DAPP1 | Homo sapiens dual adaptor of phosphotyrosine and 3-phosphoinositides (DAPP1), mRNA. | 0,64395 | 10,26057 | 4,02832 | 0,00264 | 0,03146 |
| TUBB6 | Homo sapiens tubulin, beta 6 (TUBB6), mRNA. | 0,64383 | 10,57534 | 3,64275 | 0,00487 | 0,04500 |
| MSN | Homo sapiens moesin (MSN), mRNA. | 0,64239 | 11,15209 | 3,97121 | 0,00289 | 0,03308 |
| NUAK2 | Homo sapiens NUAK family, SNF1-like kinase, 2 (NUAK2), mRNA. | 0,63944 | 8,06841 | 3,54435 | 0,00571 | 0,04951 |
| ZNF259 | Homo sapiens zinc finger protein 259 (ZNF259), mRNA. | 0,63821 | 9,08652 | 4,72080 | 0,00092 | 0,01757 |
| EHBP1 | Homo sapiens EH domain binding protein 1 (EHBP1), mRNA. | 0,63787 | 9,14974 | 5,38679 | 0,00036 | 0,01102 |
| SLC15A4 | Homo sapiens solute carrier family 15, member 4 (SLC15A4), mRNA. | 0,63726 | 9,09432 | 8,66073 | 0,00001 | 0,00222 |
| CCDC130 | Homo sapiens coiled-coil domain containing 130 (CCDC130), mRNA. | 0,63724 | 9,35275 | 4,43972 | 0,00140 | 0,02210 |
| FHL2 | Homo sapiens four and a half LIM domains 2 (FHL2), transcript variant 2, mRNA. | 0,63718 | 11,61753 | 5,42455 | 0,00034 | 0,01068 |
| PJA1 | Homo sapiens praja ring finger 1 (PJA1), transcript variant 2, mRNA. | 0,63615 | 9,01508 | 5,09978 | 0,00054 | 0,01331 |
| TYMP | Homo sapiens thymidine phosphorylase (TYMP), transcript variant 3, mRNA. | 0,63595 | 7,85075 | 6,82005 | 0,00006 | 0,00479 |
| BCL2L2 | Homo sapiens BCL2-like 2 (BCL2L2), mRNA. | 0,63521 | 9,90133 | 3,85767 | 0,00345 | 0,03676 |
| RTN4 | Homo sapiens reticulon 4 (RTN4), transcript variant 1, mRNA. | 0,63461 | 10,32898 | 4,71156 | 0,00094 | 0,01768 |
| BSDC1 | Homo sapiens BSD domain containing 1 (BSDC1), mRNA. | 0,63455 | 9,61799 | 4,48464 | 0,00131 | 0,02125 |
| PEA15 | Homo sapiens phosphoprotein enriched in astrocytes 15 (PEA15), mRNA. | 0,63450 | 10,13164 | 4,78385 | 0,00084 | 0,01686 |
| ZYX | Homo sapiens zyxin (ZYX), transcript variant 1, mRNA. | 0,63375 | 9,18904 | 5,68546 | 0,00024 | 0,00906 |
| F2RL1 | Homo sapiens coagulation factor II (thrombin) receptor-like 1 (F2RL1), mRNA. | 0,63320 | 8,35478 | 8,83031 | 0,00001 | 0,00202 |
| BRI3 | Homo sapiens brain protein I3 (BRI3), mRNA. | 0,63218 | 11,00482 | 7,68305 | 0,00002 | 0,00332 |
| DGKQ | Homo sapiens diacylglycerol kinase, theta 110kDa (DGKQ), mRNA. | 0,63218 | 8,49309 | 5,62225 | 0,00026 | 0,00934 |
| EIF2C2 | Homo sapiens eukaryotic translation initiation factor 2C, 2 (EIF2C2), mRNA. | 0,63216 | 9,54816 | 3,53563 | 0,00579 | 0,05000 |
| DPH3 | Homo sapiens DPH3, KTI11 homolog (S. cerevisiae) (DPH3), transcript variant 1, mRNA. | 0,63056 | 8,42234 | 4,95516 | 0,00066 | 0,01489 |
| ECGF1 | Homo sapiens endothelial cell growth factor 1 (platelet-derived) (ECGF1), mRNA. | 0,63024 | 7,82078 | 10,19357 | 0,00000 | 0,00125 |
| PRKAG2 | Homo sapiens protein kinase, AMP-activated, gamma 2 non-catalytic subunit (PRKAG2), transcript variant c, mRNA. | 0,62974 | 8,51105 | 3,58494 | 0,00535 | 0,04775 |
| RTN4R | Homo sapiens reticulon 4 receptor (RTN4R), mRNA. | 0,62905 | 7,90195 | 7,91859 | 0,00002 | 0,00315 |
| RHBDF2 | Homo sapiens rhomboid 5 homolog 2 (Drosophila) (RHBDF2), transcript variant 2, mRNA. | 0,62898 | 9,08167 | 4,79244 | 0,00083 | 0,01673 |
| KIAA0355 | Homo sapiens KIAA0355 (KIAA0355), mRNA. | 0,62886 | 8,37764 | 6,37322 | 0,00010 | 0,00614 |
| BIRC2 | Homo sapiens baculoviral IAP repeat-containing 2 (BIRC2), mRNA. | 0,62777 | 9,57329 | 4,00101 | 0,00275 | 0,03220 |
| SEC24D | Homo sapiens SEC24 related gene family, member D (S. cerevisiae) (SEC24D), mRNA. | 0,62739 | 8,17860 | 5,77498 | 0,00021 | 0,00853 |
| FHL1 | Homo sapiens four and a half LIM domains 1 (FHL1), mRNA. | 0,62696 | 8,86881 | 3,74856 | 0,00411 | 0,04061 |
| YRDC | Homo sapiens yrdC domain containing (E. coli) (YRDC), nuclear gene encoding mitochondrial protein, mRNA. | 0,62645 | 10,09841 | 3,55927 | 0,00557 | 0,04881 |
| F2RL1 | Homo sapiens coagulation factor II (thrombin) receptor-like 1 (F2RL1), mRNA. | 0,62535 | 8,57284 | 8,35430 | 0,00001 | 0,00248 |
| CREB3L2 | Homo sapiens cAMP responsive element binding protein 3-like 2 (CREB3L2), mRNA. | 0,62430 | 9,08561 | 4,30812 | 0,00171 | 0,02462 |
| KIFC2 | Homo sapiens kinesin family member C2 (KIFC2), mRNA. | 0,62418 | 9,13388 | 4,15495 | 0,00217 | 0,02786 |
| FLNA | Homo sapiens filamin A, alpha (actin binding protein 280) (FLNA), mRNA. | 0,62370 | 8,66834 | 3,78910 | 0,00385 | 0,03909 |
| SLMO1 | Homo sapiens slowmo homolog 1 (Drosophila) (SLMO1), mRNA. | 0,62339 | 8,26123 | 5,60777 | 0,00027 | 0,00942 |
| JUP | Homo sapiens junction plakoglobin (JUP), transcript variant 1, mRNA. | 0,62259 | 8,72320 | 3,94945 | 0,00299 | 0,03365 |
| FEZ2 | Homo sapiens fasciculation and elongation protein zeta 2 (zygin II) (FEZ2), transcript variant 1, mRNA. | 0,62226 | 9,97863 | 4,40410 | 0,00148 | 0,02270 |
| CLDND1 | Homo sapiens claudin domain containing 1 (CLDND1), transcript variant 1, mRNA. | 0,62150 | 10,36384 | 4,45414 | 0,00137 | 0,02183 |
| ACOT9 | Homo sapiens acyl-CoA thioesterase 9 (ACOT9), transcript variant 2, mRNA. | 0,61974 | 9,40313 | 3,85641 | 0,00346 | 0,03677 |
| TMSL3 | Homo sapiens thymosin-like 3 (TMSL3), mRNA. | 0,61949 | 10,56580 | 4,25746 | 0,00185 | 0,02570 |
| TSC22D2 | Homo sapiens TSC22 domain family, member 2 (TSC22D2), mRNA. | 0,61708 | 8,51299 | 4,13373 | 0,00224 | 0,02844 |
| GSTO1 | Homo sapiens glutathione S-transferase omega 1 (GSTO1), mRNA. | 0,61452 | 12,05162 | 3,77732 | 0,00392 | 0,03958 |
| LOC728772 | PREDICTED: Homo sapiens similar to transmembrane protein 106A, transcript variant 1 (LOC728772), mRNA. | 0,61436 | 8,15860 | 5,54035 | 0,00029 | 0,00996 |
| VASP | Homo sapiens vasodilator-stimulated phosphoprotein (VASP), mRNA. | 0,61360 | 8,35273 | 3,99597 | 0,00278 | 0,03234 |
| OSTC | Homo sapiens oligosaccharyltransferase complex subunit (OSTC), mRNA. | 0,61349 | 11,34772 | 4,72789 | 0,00091 | 0,01754 |
| CRB3 | Homo sapiens crumbs homolog 3 (Drosophila) (CRB3), transcript variant 2, mRNA. | 0,61294 | 8,19238 | 6,08840 | 0,00014 | 0,00713 |
| KIAA0430 | Homo sapiens KIAA0430 (KIAA0430), mRNA. | 0,61291 | 8,55652 | 3,69406 | 0,00448 | 0,04285 |
| FHL2 | Homo sapiens four and a half LIM domains 2 (FHL2), transcript variant 4, mRNA. | 0,61225 | 11,02868 | 6,99571 | 0,00005 | 0,00442 |
| FAM114A1 | Homo sapiens family with sequence similarity 114, member A1 (FAM114A1), mRNA. | 0,61206 | 8,10768 | 6,92730 | 0,00005 | 0,00457 |
| LOC646567 | PREDICTED: Homo sapiens similar to HSPC307 (LOC646567), mRNA. | 0,61185 | 10,31347 | 5,76387 | 0,00022 | 0,00858 |
| PI4KAP2 | Homo sapiens phosphatidylinositol 4-kinase, catalytic, alpha polypeptide pseudogene 2 (PI4KAP2), mRNA. | 0,61112 | 9,21634 | 4,09816 | 0,00237 | 0,02944 |
| ELOVL1 | Homo sapiens elongation of very long chain fatty acids (FEN1/Elo2, SUR4/Elo3, yeast)-like 1 (ELOVL1), mRNA. | 0,61026 | 8,67273 | 4,22004 | 0,00196 | 0,02639 |
| IL4R | Homo sapiens interleukin 4 receptor (IL4R), transcript variant 1, mRNA. | 0,61001 | 8,67573 | 4,75140 | 0,00088 | 0,01721 |
| METRNL | Homo sapiens meteorin, glial cell differentiation regulator-like (METRNL), mRNA. | 0,60949 | 7,78319 | 5,29944 | 0,00041 | 0,01159 |
| CD99 | Homo sapiens CD99 molecule (CD99), transcript variant 1, mRNA. | 0,60890 | 8,68417 | 3,84317 | 0,00353 | 0,03723 |
| HLA-E | Homo sapiens major histocompatibility complex, class I, E (HLA-E), mRNA. | 0,60808 | 9,86013 | 4,34659 | 0,00162 | 0,02391 |
| MED8 | Homo sapiens mediator of RNA polymerase II transcription, subunit 8 homolog (S. cerevisiae) (MED8), transcript variant 3, mRNA. | 0,60736 | 8,62243 | 8,28029 | 0,00001 | 0,00260 |
| HEY1 | Homo sapiens hairy/enhancer-of-split related with YRPW motif 1 (HEY1), transcript variant 2, mRNA. | 0,60731 | 8,03862 | 8,03595 | 0,00001 | 0,00292 |
| PHLDA3 | Homo sapiens pleckstrin homology-like domain, family A, member 3 (PHLDA3), mRNA. | 0,60696 | 9,26566 | 3,54252 | 0,00573 | 0,04962 |
| GOSR2 | Homo sapiens golgi SNAP receptor complex member 2 (GOSR2), transcript variant A, mRNA. | 0,60634 | 8,90755 | 3,62362 | 0,00502 | 0,04586 |
| ENTPD6 | Homo sapiens ectonucleoside triphosphate diphosphohydrolase 6 (putative function) (ENTPD6), mRNA. | 0,60617 | 8,89232 | 4,54638 | 0,00120 | 0,02019 |
| MAFF | Homo sapiens v-maf musculoaponeurotic fibrosarcoma oncogene homolog F (avian) (MAFF), transcript variant 1, mRNA. | 0,60475 | 7,89546 | 4,85079 | 0,00076 | 0,01607 |
| MYH9 | Homo sapiens myosin, heavy chain 9, non-muscle (MYH9), mRNA. | 0,60457 | 12,19362 | 4,13415 | 0,00224 | 0,02844 |
| SMTN | Homo sapiens smoothelin (SMTN), transcript variant 3, mRNA. | 0,60433 | 8,10422 | 3,57877 | 0,00540 | 0,04804 |
| IL8 | Homo sapiens interleukin 8 (IL8), mRNA. | 0,60057 | 8,13344 | 4,76605 | 0,00086 | 0,01710 |
| SMG1 | Homo sapiens PI-3-kinase-related kinase SMG-1 (SMG1), mRNA. | 0,59952 | 8,32962 | 3,82621 | 0,00363 | 0,03768 |
| UFM1 | Homo sapiens ubiquitin-fold modifier 1 (UFM1), mRNA. | 0,59906 | 9,27631 | 3,91950 | 0,00313 | 0,03455 |
| MAPKAPK3 | Homo sapiens mitogen-activated protein kinase-activated protein kinase 3 (MAPKAPK3), mRNA. | 0,59880 | 9,43437 | 4,35372 | 0,00160 | 0,02376 |
| ACOT9 | Homo sapiens acyl-CoA thioesterase 9 (ACOT9), transcript variant 1, mRNA. | 0,59818 | 8,33084 | 3,77823 | 0,00392 | 0,03955 |
| ITPRIP | Homo sapiens inositol 1,4,5-triphosphate receptor interacting protein (ITPRIP), mRNA. | 0,59763 | 8,98284 | 4,99778 | 0,00062 | 0,01436 |
| KLHL28 | Homo sapiens kelch-like 28 (Drosophila) (KLHL28), mRNA. | 0,59763 | 8,15562 | 5,01653 | 0,00060 | 0,01422 |
| RAB5A | Homo sapiens RAB5A, member RAS oncogene family (RAB5A), mRNA. | 0,59739 | 9,17630 | 4,37539 | 0,00155 | 0,02330 |
| CSNK1D | Homo sapiens casein kinase 1, delta (CSNK1D), transcript variant 2, mRNA. | 0,59731 | 9,43863 | 4,20662 | 0,00200 | 0,02659 |
| ABR | Homo sapiens active BCR-related gene (ABR), transcript variant 2, mRNA. | 0,59650 | 9,23277 | 3,56360 | 0,00553 | 0,04867 |
| ASB1 | Homo sapiens ankyrin repeat and SOCS box-containing 1 (ASB1), mRNA. | 0,59496 | 8,08085 | 7,46282 | 0,00003 | 0,00368 |
| FLII | Homo sapiens flightless I homolog (Drosophila) (FLII), mRNA. | 0,59336 | 8,40159 | 4,61058 | 0,00109 | 0,01926 |
| RAB31 | Homo sapiens RAB31, member RAS oncogene family (RAB31), mRNA. | 0,59304 | 11,87036 | 5,17589 | 0,00048 | 0,01265 |
| GK | Homo sapiens glycerol kinase (GK), transcript variant 1, mRNA. | 0,59134 | 7,82821 | 3,97097 | 0,00289 | 0,03308 |
| RPRC1 | Homo sapiens arginine/proline rich coiled-coil 1 (RPRC1), mRNA. | 0,59116 | 11,77962 | 5,45086 | 0,00033 | 0,01050 |
| PLCH2 | Homo sapiens phospholipase C, eta 2 (PLCH2), mRNA. | 0,59059 | 7,63103 | 5,71430 | 0,00023 | 0,00892 |
| SYVN1 | Homo sapiens synovial apoptosis inhibitor 1, synoviolin (SYVN1), transcript variant 1, mRNA. | 0,59041 | 8,72269 | 4,32578 | 0,00167 | 0,02429 |
| MYH9 | Homo sapiens myosin, heavy chain 9, non-muscle (MYH9), mRNA. | 0,58882 | 11,63374 | 4,53711 | 0,00121 | 0,02030 |
| CITED2 | Homo sapiens Cbp/p300-interacting transactivator, with Glu/Asp-rich carboxy-terminal domain, 2 (CITED2), transcript variant 1, mRNA. | 0,58813 | 8,56028 | 4,06859 | 0,00248 | 0,03027 |
| IBTK | Homo sapiens inhibitor of Bruton agammaglobulinemia tyrosine kinase (IBTK), mRNA. | 0,58427 | 9,00647 | 6,35813 | 0,00010 | 0,00620 |
| PSCD2 | Homo sapiens pleckstrin homology, Sec7 and coiled-coil domains 2 (cytohesin-2) (PSCD2), transcript variant 2, mRNA. | 0,58409 | 8,05925 | 4,54321 | 0,00120 | 0,02022 |
| SYT7 | Homo sapiens synaptotagmin VII (SYT7), mRNA. | 0,58342 | 7,69338 | 3,86046 | 0,00344 | 0,03666 |
| S100A3 | Homo sapiens S100 calcium binding protein A3 (S100A3), mRNA. | 0,58132 | 8,02547 | 4,71932 | 0,00093 | 0,01757 |
| HS1BP3 | Homo sapiens HCLS1 binding protein 3 (HS1BP3), mRNA. | 0,58122 | 8,46626 | 5,54321 | 0,00029 | 0,00996 |
| ARPC5 | Homo sapiens actin related protein 2/3 complex, subunit 5, 16kDa (ARPC5), mRNA. | 0,58104 | 11,09619 | 7,25106 | 0,00004 | 0,00401 |
| CAP1 | Homo sapiens CAP, adenylate cyclase-associated protein 1 (yeast) (CAP1), mRNA. | 0,58102 | 12,72959 | 5,38373 | 0,00036 | 0,01103 |
| FBXO11 | Homo sapiens F-box protein 11 (FBXO11), transcript variant 1, mRNA. | 0,58081 | 9,42727 | 5,59763 | 0,00027 | 0,00952 |
| RNF14 | Homo sapiens ring finger protein 14 (RNF14), transcript variant 3, mRNA. | 0,57900 | 9,16000 | 6,21747 | 0,00012 | 0,00681 |
| LHFPL2 | Homo sapiens lipoma HMGIC fusion partner-like 2 (LHFPL2), mRNA. | 0,57853 | 8,15097 | 3,74352 | 0,00414 | 0,04091 |
| MAPK6 | Homo sapiens mitogen-activated protein kinase 6 (MAPK6), mRNA. | 0,57767 | 9,84532 | 3,60970 | 0,00514 | 0,04648 |
| TINF2 | Homo sapiens TERF1 (TRF1)-interacting nuclear factor 2 (TINF2), mRNA. | 0,57719 | 8,93365 | 4,30194 | 0,00173 | 0,02477 |
| DPH3 | Homo sapiens DPH3, KTI11 homolog (S. cerevisiae) (DPH3), transcript variant 2, mRNA. | 0,57691 | 8,44927 | 6,24300 | 0,00012 | 0,00667 |
| TPD52L2 | Homo sapiens tumor protein D52-like 2 (TPD52L2), transcript variant 2, mRNA. | 0,57421 | 9,00139 | 5,46314 | 0,00032 | 0,01046 |
| TSPO | Homo sapiens translocator protein (18kDa) (TSPO), transcript variant PBR, mRNA. | 0,57287 | 11,31828 | 4,28923 | 0,00176 | 0,02499 |
| GALE | Homo sapiens UDP-galactose-4-epimerase (GALE), transcript variant 1, mRNA. | 0,57276 | 8,77056 | 5,17494 | 0,00048 | 0,01265 |
| CBLC | Homo sapiens Cas-Br-M (murine) ecotropic retroviral transforming sequence c (CBLC), mRNA. | 0,57247 | 8,21452 | 5,37576 | 0,00036 | 0,01111 |
| RELA | Homo sapiens v-rel reticuloendotheliosis viral oncogene homolog A (avian) (RELA), mRNA. | 0,57246 | 8,52701 | 3,78326 | 0,00389 | 0,03934 |
| CLDND1 | Homo sapiens claudin domain containing 1 (CLDND1), transcript variant 1, mRNA. | 0,57199 | 10,57089 | 5,01493 | 0,00060 | 0,01423 |
| BTBD10 | Homo sapiens BTB (POZ) domain containing 10 (BTBD10), mRNA. | 0,57132 | 9,13239 | 7,77960 | 0,00002 | 0,00325 |
| HM13 | Homo sapiens histocompatibility (minor) 13 (HM13), transcript variant 2, mRNA. | 0,57037 | 8,61247 | 6,79660 | 0,00006 | 0,00479 |
| AIDA | Homo sapiens axin interactor, dorsalization associated (AIDA), mRNA. | 0,56842 | 10,77873 | 7,75312 | 0,00002 | 0,00325 |
| NUCB2 | Homo sapiens nucleobindin 2 (NUCB2), mRNA. | 0,56820 | 8,53095 | 5,01340 | 0,00061 | 0,01424 |
| DRAP1 | Homo sapiens DR1-associated protein 1 (negative cofactor 2 alpha) (DRAP1), mRNA. | 0,56691 | 12,26420 | 4,15300 | 0,00217 | 0,02789 |
| SEC23B | Homo sapiens Sec23 homolog B (S. cerevisiae) (SEC23B), transcript variant 2, mRNA. | 0,56658 | 9,78715 | 8,65260 | 0,00001 | 0,00222 |
| GOLGB1 | Homo sapiens golgin B1, golgi integral membrane protein (GOLGB1), mRNA. | 0,56644 | 8,51365 | 3,65447 | 0,00478 | 0,04459 |
| SLC25A43 | Homo sapiens solute carrier family 25, member 43 (SLC25A43), mRNA. | 0,56580 | 10,37708 | 5,07169 | 0,00056 | 0,01358 |
| BNIP2 | Homo sapiens BCL2/adenovirus E1B 19kDa interacting protein 2 (BNIP2), mRNA. | 0,56481 | 9,05959 | 4,56887 | 0,00116 | 0,01989 |
| UBE2Q2 | Homo sapiens ubiquitin-conjugating enzyme E2Q family member 2 (UBE2Q2), mRNA. | 0,56115 | 8,43986 | 6,59714 | 0,00008 | 0,00541 |
| RALB | Homo sapiens v-ral simian leukemia viral oncogene homolog B (ras related; GTP binding protein) (RALB), mRNA. | 0,56114 | 10,70904 | 4,91247 | 0,00070 | 0,01546 |
| APCDD1L | Homo sapiens adenomatosis polyposis coli down-regulated 1-like (APCDD1L), mRNA. | 0,56045 | 8,28454 | 3,59922 | 0,00522 | 0,04699 |
| KIFAP3 | Homo sapiens kinesin-associated protein 3 (KIFAP3), mRNA. | 0,56028 | 8,18126 | 4,36072 | 0,00158 | 0,02364 |
| MYL12A | Homo sapiens myosin, light chain 12A, regulatory, non-sarcomeric (MYL12A), mRNA. | 0,55995 | 13,28314 | 9,82845 | 0,00000 | 0,00146 |
| CCDC92 | Homo sapiens coiled-coil domain containing 92 (CCDC92), mRNA. | 0,55965 | 8,40324 | 3,61250 | 0,00511 | 0,04639 |
| LOC644590 | PREDICTED: Homo sapiens similar to EVIN1 (LOC644590), mRNA. | 0,55893 | 7,75944 | 4,70901 | 0,00094 | 0,01769 |
| EFTUD1 | Homo sapiens elongation factor Tu GTP binding domain containing 1 (EFTUD1), transcript variant 1, mRNA. | 0,55866 | 8,10608 | 4,17161 | 0,00211 | 0,02745 |
| TAX1BP1 | Homo sapiens Tax1 (human T-cell leukemia virus type I) binding protein 1 (TAX1BP1), transcript variant 2, mRNA. | 0,55833 | 11,35407 | 5,15889 | 0,00049 | 0,01270 |
| NCK2 | Homo sapiens NCK adaptor protein 2 (NCK2), transcript variant 2, mRNA. | 0,55709 | 9,61059 | 7,76273 | 0,00002 | 0,00325 |
| CRLF3 | Homo sapiens cytokine receptor-like factor 3 (CRLF3), mRNA. | 0,55679 | 8,62304 | 4,07986 | 0,00244 | 0,02995 |
| LOC653778 | PREDICTED: Homo sapiens similar to solute carrier family 25, member 37 (LOC653778), mRNA. | 0,55621 | 8,17421 | 7,03700 | 0,00004 | 0,00440 |
| SEC23B | Homo sapiens Sec23 homolog B (S. cerevisiae) (SEC23B), transcript variant 2, mRNA. | 0,55581 | 9,51537 | 7,70145 | 0,00002 | 0,00332 |
| MGST3 | Homo sapiens microsomal glutathione S-transferase 3 (MGST3), mRNA. | 0,55571 | 10,40011 | 4,21359 | 0,00198 | 0,02649 |
| CLIC4 | Homo sapiens chloride intracellular channel 4 (CLIC4), nuclear gene encoding mitochondrial protein, mRNA. | 0,55435 | 7,92538 | 4,52659 | 0,00123 | 0,02048 |
| ITPRIP | Homo sapiens inositol 1,4,5-triphosphate receptor interacting protein (ITPRIP), mRNA. | 0,55418 | 9,23825 | 3,55416 | 0,00562 | 0,04905 |
| C16orf72 | Homo sapiens chromosome 16 open reading frame 72 (C16orf72), mRNA. | 0,55351 | 8,97912 | 3,82400 | 0,00364 | 0,03773 |
| ANKRD11 | Homo sapiens ankyrin repeat domain 11 (ANKRD11), mRNA. | 0,55341 | 9,37963 | 3,71378 | 0,00434 | 0,04202 |
| MAP4K4 | Homo sapiens mitogen-activated protein kinase kinase kinase kinase 4 (MAP4K4), transcript variant 3, mRNA. | 0,55333 | 8,16106 | 5,13988 | 0,00051 | 0,01290 |
| SLC35C1 | Homo sapiens solute carrier family 35, member C1 (SLC35C1), mRNA. | 0,55321 | 8,58541 | 6,47749 | 0,00009 | 0,00588 |
| NOP10 | Homo sapiens NOP10 ribonucleoprotein homolog (yeast) (NOP10), mRNA. | 0,55279 | 11,62335 | 3,65516 | 0,00477 | 0,04458 |
| DSE | Homo sapiens dermatan sulfate epimerase (DSE), transcript variant 1, mRNA. | 0,55247 | 10,20767 | 4,06601 | 0,00249 | 0,03032 |
| PBX3 | Homo sapiens pre-B-cell leukemia homeobox 3 (PBX3), mRNA. | 0,55196 | 9,01158 | 5,74251 | 0,00022 | 0,00873 |
| CDK2AP2 | Homo sapiens cyclin-dependent kinase 2 associated protein 2 (CDK2AP2), mRNA. | 0,55188 | 9,07532 | 3,62154 | 0,00504 | 0,04598 |
| RSPRY1 | Homo sapiens ring finger and SPRY domain containing 1 (RSPRY1), mRNA. | 0,54986 | 9,25039 | 4,19883 | 0,00203 | 0,02678 |
| LPAR3 | Homo sapiens lysophosphatidic acid receptor 3 (LPAR3), mRNA. | 0,54940 | 8,18383 | 4,80529 | 0,00082 | 0,01661 |
| INO80C | Homo sapiens INO80 complex subunit C (INO80C), transcript variant 2, mRNA. | 0,54917 | 8,65877 | 4,14734 | 0,00219 | 0,02804 |
| SLC6A9 | Homo sapiens solute carrier family 6 (neurotransmitter transporter, glycine), member 9 (SLC6A9), transcript variant 3, mRNA. | 0,54706 | 7,97199 | 3,72386 | 0,00427 | 0,04167 |
| ATP6V1G1 | Homo sapiens ATPase, H+ transporting, lysosomal 13kDa, V1 subunit G1 (ATP6V1G1), mRNA. | 0,54679 | 11,84894 | 5,33450 | 0,00039 | 0,01132 |
| FOXN2 | Homo sapiens forkhead box N2 (FOXN2), mRNA. | 0,54679 | 8,57021 | 4,27761 | 0,00179 | 0,02520 |
| PICALM | Homo sapiens phosphatidylinositol binding clathrin assembly protein (PICALM), transcript variant 1, mRNA. | 0,54641 | 10,30004 | 4,34513 | 0,00162 | 0,02394 |
| WASL | Homo sapiens Wiskott-Aldrich syndrome-like (WASL), mRNA. | 0,54633 | 9,05829 | 4,20954 | 0,00199 | 0,02654 |
| LOC728532 | PREDICTED: Homo sapiens misc_RNA (LOC728532), miscRNA. | 0,54578 | 9,23972 | 4,41583 | 0,00145 | 0,02248 |
| PXN | Homo sapiens paxillin (PXN), mRNA. | 0,54422 | 8,36727 | 4,60025 | 0,00110 | 0,01941 |
| QSOX1 | Homo sapiens quiescin Q6 sulfhydryl oxidase 1 (QSOX1), transcript variant 2, mRNA. | 0,54372 | 8,69120 | 4,46064 | 0,00136 | 0,02168 |
| MYADM | Homo sapiens myeloid-associated differentiation marker (MYADM), transcript variant 2, mRNA. | 0,54370 | 7,71391 | 7,92671 | 0,00002 | 0,00315 |
| FAM100B | Homo sapiens family with sequence similarity 100, member B (FAM100B), mRNA. | 0,54316 | 8,07606 | 6,02872 | 0,00015 | 0,00732 |
| C6orf160 | PREDICTED: Homo sapiens chromosome 6 open reading frame 160, transcript variant 4 (C6orf160), mRNA. | 0,54251 | 11,80834 | 7,87749 | 0,00002 | 0,00322 |
| PSD4 | Homo sapiens pleckstrin and Sec7 domain containing 4 (PSD4), mRNA. | 0,54231 | 7,79636 | 6,10842 | 0,00014 | 0,00709 |
| PHF11 | Homo sapiens PHD finger protein 11 (PHF11), transcript variant 1, mRNA. | 0,54222 | 8,36653 | 4,11918 | 0,00229 | 0,02881 |
| SLK | Homo sapiens STE20-like kinase (yeast) (SLK), mRNA. | 0,54149 | 8,90908 | 3,67902 | 0,00459 | 0,04349 |
| RELB | Homo sapiens v-rel reticuloendotheliosis viral oncogene homolog B (RELB), mRNA. | 0,54020 | 8,06927 | 6,60671 | 0,00007 | 0,00537 |
| STAM | Homo sapiens signal transducing adaptor molecule (SH3 domain and ITAM motif) 1 (STAM), mRNA. | 0,53918 | 8,40637 | 4,65973 | 0,00101 | 0,01836 |
| TNFRSF10B | Homo sapiens tumor necrosis factor receptor superfamily, member 10b (TNFRSF10B), transcript variant 1, mRNA. | 0,53794 | 9,65035 | 3,80305 | 0,00377 | 0,03857 |
| DYSF | Homo sapiens dysferlin, limb girdle muscular dystrophy 2B (autosomal recessive) (DYSF), mRNA. | 0,53732 | 7,60858 | 5,16991 | 0,00049 | 0,01265 |
| GOLT1B | Homo sapiens golgi transport 1 homolog B (S. cerevisiae) (GOLT1B), mRNA. | 0,53729 | 8,72319 | 3,97384 | 0,00287 | 0,03307 |
| FTL | Homo sapiens ferritin, light polypeptide (FTL), mRNA. | 0,53620 | 13,57729 | 3,91481 | 0,00315 | 0,03472 |
| DMTF1 | Homo sapiens cyclin D binding myb-like transcription factor 1 (DMTF1), mRNA. | 0,53559 | 8,23382 | 3,65476 | 0,00478 | 0,04459 |
| MTMR11 | Homo sapiens myotubularin related protein 11 (MTMR11), mRNA. | 0,53480 | 8,38871 | 3,65745 | 0,00475 | 0,04446 |
| KCNN4 | Homo sapiens potassium intermediate/small conductance calcium-activated channel, subfamily N, member 4 (KCNN4), mRNA. | 0,53459 | 7,79545 | 3,85690 | 0,00346 | 0,03676 |
| MAPKAPK2 | Homo sapiens mitogen-activated protein kinase-activated protein kinase 2 (MAPKAPK2), transcript variant 2, mRNA. | 0,53455 | 9,38568 | 4,02953 | 0,00263 | 0,03145 |
| ARFGAP1 | Homo sapiens ADP-ribosylation factor GTPase activating protein 1 (ARFGAP1), transcript variant 1, mRNA. | 0,53430 | 9,16085 | 3,95789 | 0,00295 | 0,03345 |
| DVL1 | Homo sapiens dishevelled, dsh homolog 1 (Drosophila) (DVL1), transcript variant 2, mRNA. | 0,53170 | 7,88630 | 4,18691 | 0,00206 | 0,02709 |
| SLCO1B3 | Homo sapiens solute carrier organic anion transporter family, member 1B3 (SLCO1B3), mRNA. | 0,53158 | 8,21249 | 4,07074 | 0,00247 | 0,03024 |
| NRBF2 | Homo sapiens nuclear receptor binding factor 2 (NRBF2), mRNA. | 0,53105 | 8,17743 | 4,38682 | 0,00152 | 0,02308 |
| NAMPT | Homo sapiens nicotinamide phosphoribosyltransferase (NAMPT), mRNA. | 0,53057 | 8,30726 | 4,41985 | 0,00145 | 0,02247 |
| UBA6 | Homo sapiens ubiquitin-like modifier activating enzyme 6 (UBA6), mRNA. | 0,53044 | 8,90764 | 5,76797 | 0,00022 | 0,00855 |
| ITGB1 | Homo sapiens integrin, beta 1 (fibronectin receptor, beta polypeptide, antigen CD29 includes MDF2, MSK12) (ITGB1), transcript variant 1C-2, mRNA. | 0,53040 | 11,50849 | 5,88874 | 0,00018 | 0,00789 |
| SLC31A2 | Homo sapiens solute carrier family 31 (copper transporters), member 2 (SLC31A2), mRNA. | 0,53026 | 10,31743 | 5,85451 | 0,00019 | 0,00807 |
| CBL | Homo sapiens Cas-Br-M (murine) ecotropic retroviral transforming sequence (CBL), mRNA. | 0,52843 | 8,53561 | 4,80423 | 0,00082 | 0,01661 |
| SPRYD3 | Homo sapiens SPRY domain containing 3 (SPRYD3), mRNA. | 0,52834 | 8,29163 | 6,80332 | 0,00006 | 0,00479 |
| RSL24D1 | Homo sapiens ribosomal L24 domain containing 1 (RSL24D1), mRNA. | 0,52509 | 10,83382 | 5,91146 | 0,00018 | 0,00776 |
| ITGB1 | Homo sapiens integrin, beta 1 (fibronectin receptor, beta polypeptide, antigen CD29 includes MDF2, MSK12) (ITGB1), transcript variant 1A, mRNA. | 0,52494 | 12,47945 | 6,26089 | 0,00011 | 0,00663 |
| TMEM214 | Homo sapiens transmembrane protein 214 (TMEM214), transcript variant 1, mRNA. | 0,52348 | 9,20882 | 4,05828 | 0,00252 | 0,03062 |
| DEF8 | Homo sapiens differentially expressed in FDCP 8 homolog (mouse) (DEF8), transcript variant 1, mRNA. | 0,52004 | 8,68112 | 4,94365 | 0,00067 | 0,01506 |
| INSIG2 | Homo sapiens insulin induced gene 2 (INSIG2), mRNA. | 0,51936 | 8,58891 | 5,53085 | 0,00030 | 0,00999 |
| HABP4 | Homo sapiens hyaluronan binding protein 4 (HABP4), mRNA. | 0,51890 | 8,12914 | 6,29135 | 0,00011 | 0,00653 |
| OSTC | Homo sapiens oligosaccharyltransferase complex subunit (OSTC), mRNA. | 0,51849 | 11,74396 | 4,59721 | 0,00111 | 0,01945 |
| NBPF3 | Homo sapiens neuroblastoma breakpoint family, member 3 (NBPF3), mRNA. | 0,51764 | 8,13048 | 4,28291 | 0,00178 | 0,02512 |
| DUSP4 | Homo sapiens dual specificity phosphatase 4 (DUSP4), transcript variant 1, mRNA. | 0,51535 | 7,57875 | 4,41601 | 0,00145 | 0,02248 |
| TRPC4AP | Homo sapiens transient receptor potential cation channel, subfamily C, member 4 associated protein (TRPC4AP), transcript variant 1, mRNA. | 0,51145 | 9,27549 | 3,98695 | 0,00282 | 0,03262 |
| NMD3 | Homo sapiens NMD3 homolog (S. cerevisiae) (NMD3), mRNA. | 0,51075 | 9,40379 | 6,44933 | 0,00009 | 0,00599 |
| PLEKHM2 | Homo sapiens pleckstrin homology domain containing, family M (with RUN domain) member 2 (PLEKHM2), mRNA. | 0,50503 | 9,60758 | 3,70874 | 0,00438 | 0,04223 |
| DUSP11 | Homo sapiens dual specificity phosphatase 11 (RNA/RNP complex 1-interacting) (DUSP11), mRNA. | 0,50463 | 9,94095 | 3,54051 | 0,00574 | 0,04970 |
| PDGFB | Homo sapiens platelet-derived growth factor beta polypeptide (simian sarcoma viral (v-sis) oncogene homolog) (PDGFB), transcript variant 1, mRNA. | 0,50371 | 8,28328 | 3,82851 | 0,00362 | 0,03765 |
| RAI14 | Homo sapiens retinoic acid induced 14 (RAI14), mRNA. | 0,50339 | 9,97750 | 4,73551 | 0,00090 | 0,01742 |
| ZNF277 | Homo sapiens zinc finger protein 277 (ZNF277), mRNA. | 0,50336 | 8,45728 | 4,51770 | 0,00125 | 0,02061 |
| FBXO11 | Homo sapiens F-box protein 11 (FBXO11), transcript variant 1, mRNA. | 0,50258 | 8,82592 | 3,87966 | 0,00333 | 0,03589 |
| ITGB1 | Homo sapiens integrin, beta 1 (fibronectin receptor, beta polypeptide, antigen CD29 includes MDF2, MSK12) (ITGB1), transcript variant 1D, mRNA. | 0,50083 | 10,74427 | 4,55301 | 0,00118 | 0,02014 |
| SEPHS1 | Homo sapiens selenophosphate synthetase 1 (SEPHS1), mRNA. | -0,50026 | 8,08194 | -4,50516 | 0,00127 | 0,02079 |
| HNRPUL1 | Homo sapiens heterogeneous nuclear ribonucleoprotein U-like 1 (HNRPUL1), transcript variant 4, mRNA. | -0,50147 | 10,66336 | -6,05943 | 0,00015 | 0,00726 |
| LSM3 | Homo sapiens LSM3 homolog, U6 small nuclear RNA associated (S. cerevisiae) (LSM3), mRNA. | -0,50296 | 10,52897 | -3,69805 | 0,00445 | 0,04271 |
| FIGNL1 | Homo sapiens fidgetin-like 1 (FIGNL1), transcript variant 1, mRNA. | -0,50433 | 7,66064 | -7,12171 | 0,00004 | 0,00422 |
| ABCB9 | Homo sapiens ATP-binding cassette, sub-family B (MDR/TAP), member 9 (ABCB9), transcript variant 2, mRNA. | -0,50460 | 7,87417 | -4,68638 | 0,00097 | 0,01795 |
| DDX10 | Homo sapiens DEAD (Asp-Glu-Ala-Asp) box polypeptide 10 (DDX10), mRNA. | -0,50537 | 9,13248 | -3,56528 | 0,00552 | 0,04861 |
| MRPL35 | Homo sapiens mitochondrial ribosomal protein L35 (MRPL35), nuclear gene encoding mitochondrial protein, transcript variant 1, mRNA. | -0,50568 | 8,07817 | -6,51476 | 0,00008 | 0,00572 |
| ATP1A1 | Homo sapiens ATPase, Na+/K+ transporting, alpha 1 polypeptide (ATP1A1), transcript variant 1, mRNA. | -0,50591 | 9,45688 | -4,10223 | 0,00235 | 0,02935 |
| CXCL16 | Homo sapiens chemokine (C-X-C motif) ligand 16 (CXCL16), mRNA. | -0,50594 | 7,77802 | -4,12525 | 0,00227 | 0,02871 |
| BRI3BP | Homo sapiens BRI3 binding protein (BRI3BP), mRNA. | -0,50662 | 8,07871 | -4,43214 | 0,00142 | 0,02220 |
| RAB34 | Homo sapiens RAB34, member RAS oncogene family (RAB34), mRNA. | -0,50990 | 9,60165 | -5,66329 | 0,00025 | 0,00915 |
| UQCRFS1 | Homo sapiens ubiquinol-cytochrome c reductase, Rieske iron-sulfur polypeptide 1 (UQCRFS1), mRNA. | -0,51047 | 11,86264 | -4,11984 | 0,00229 | 0,02880 |
| GTSE1 | Homo sapiens G-2 and S-phase expressed 1 (GTSE1), mRNA. | -0,51064 | 7,75774 | -3,72433 | 0,00427 | 0,04167 |
| ALDH3A2 | Homo sapiens aldehyde dehydrogenase 3 family, member A2 (ALDH3A2), transcript variant 1, mRNA. | -0,51098 | 7,80892 | -7,88736 | 0,00002 | 0,00321 |
| TIMM8A | Homo sapiens translocase of inner mitochondrial membrane 8 homolog A (yeast) (TIMM8A), nuclear gene encoding mitochondrial protein, mRNA. | -0,51121 | 8,05180 | -3,85385 | 0,00347 | 0,03689 |
| TRAFD1 | Homo sapiens TRAF-type zinc finger domain containing 1 (TRAFD1), mRNA. | -0,51131 | 7,96649 | -5,78987 | 0,00021 | 0,00843 |
| TCP1 | Homo sapiens t-complex 1 (TCP1), transcript variant 1, mRNA. | -0,51152 | 9,58267 | -5,92996 | 0,00017 | 0,00774 |
| POLR2L | Homo sapiens polymerase (RNA) II (DNA directed) polypeptide L, 7.6kDa (POLR2L), mRNA. | -0,51167 | 7,65312 | -4,90150 | 0,00071 | 0,01559 |
| RFC2 | Homo sapiens replication factor C (activator 1) 2, 40kDa (RFC2), transcript variant 1, mRNA. | -0,51254 | 7,79332 | -5,32754 | 0,00039 | 0,01134 |
| PEG10 | Homo sapiens paternally expressed 10 (PEG10), transcript variant 1, mRNA. XM_940378 | -0,51373 | 7,63917 | -4,00355 | 0,00274 | 0,03212 |
| WDR12 | Homo sapiens WD repeat domain 12 (WDR12), mRNA. | -0,51391 | 8,88247 | -4,02238 | 0,00266 | 0,03165 |
| METTL1 | Homo sapiens methyltransferase like 1 (METTL1), transcript variant 1, mRNA. | -0,51430 | 8,21641 | -3,67652 | 0,00461 | 0,04357 |
| DNAJC8 | Homo sapiens DnaJ (Hsp40) homolog, subfamily C, member 8 (DNAJC8), mRNA. | -0,51468 | 10,94472 | -3,84070 | 0,00355 | 0,03726 |
| LOC644774 | PREDICTED: Homo sapiens similar to Phosphoglycerate kinase 1 (LOC644774), mRNA. | -0,51470 | 10,81722 | -4,33777 | 0,00164 | 0,02401 |
| SESN3 | Homo sapiens sestrin 3 (SESN3), mRNA. | -0,51498 | 7,49406 | -5,11371 | 0,00053 | 0,01314 |
| PSMB2 | Homo sapiens proteasome (prosome, macropain) subunit, beta type, 2 (PSMB2), mRNA. | -0,51509 | 11,59577 | -6,67878 | 0,00007 | 0,00503 |
| EXO1 | Homo sapiens exonuclease 1 (EXO1), transcript variant 1, mRNA. | -0,51562 | 7,56502 | -7,00653 | 0,00005 | 0,00442 |
| GLE1 | Homo sapiens GLE1 RNA export mediator homolog (yeast) (GLE1), transcript variant 1, mRNA. | -0,51593 | 9,66140 | -3,63533 | 0,00493 | 0,04531 |
| EARS2 | Homo sapiens glutamyl-tRNA synthetase 2, mitochondrial (putative) (EARS2), transcript variant 2, non-coding RNA. | -0,51595 | 7,97637 | -3,66832 | 0,00467 | 0,04394 |
| TIPIN | Homo sapiens TIMELESS interacting protein (TIPIN), mRNA. | -0,51628 | 7,80063 | -4,54894 | 0,00119 | 0,02018 |
| FANCI | Homo sapiens Fanconi anemia, complementation group I (FANCI), transcript variant 2, mRNA. | -0,51652 | 7,82606 | -3,57955 | 0,00539 | 0,04801 |
| LOC100130178 | PREDICTED: Homo sapiens misc_RNA (LOC100130178), miscRNA. | -0,51707 | 9,80103 | -4,03815 | 0,00260 | 0,03120 |
| EZH2 | Homo sapiens enhancer of zeste homolog 2 (Drosophila) (EZH2), transcript variant 2, mRNA. | -0,51713 | 7,75906 | -4,46781 | 0,00135 | 0,02155 |
| NUP62 | Homo sapiens nucleoporin 62kDa (NUP62), transcript variant 2, mRNA. | -0,51781 | 11,12623 | -3,73919 | 0,00417 | 0,04107 |
| PSMB4 | Homo sapiens proteasome (prosome, macropain) subunit, beta type, 4 (PSMB4), mRNA. | -0,51813 | 10,70239 | -3,68446 | 0,00455 | 0,04329 |
| DNM3 | Homo sapiens dynamin 3 (DNM3), mRNA. | -0,51820 | 7,80187 | -4,17300 | 0,00211 | 0,02742 |
| ZW10 | Homo sapiens ZW10, kinetochore associated, homolog (Drosophila) (ZW10), mRNA. | -0,51953 | 9,31588 | -6,26361 | 0,00011 | 0,00663 |
| RPL29 | Homo sapiens ribosomal protein L29 (RPL29), mRNA. | -0,51961 | 9,51296 | -3,54178 | 0,00573 | 0,04965 |
| MTP18 | Homo sapiens mitochondrial protein 18 kDa (MTP18), nuclear gene encoding mitochondrial protein, transcript variant 1, mRNA. | -0,52039 | 8,80443 | -5,50132 | 0,00031 | 0,01017 |
| TAF1C | Homo sapiens TATA box binding protein (TBP)-associated factor, RNA polymerase I, C, 110kDa (TAF1C), transcript variant 2, mRNA. | -0,52072 | 9,30857 | -4,55328 | 0,00118 | 0,02014 |
| GTF2IP1 | Homo sapiens general transcription factor II, i, pseudogene 1 (GTF2IP1) on chromosome 7. | -0,52097 | 7,97975 | -4,26590 | 0,00183 | 0,02550 |
| TUBB2C | Homo sapiens tubulin, beta 2C (TUBB2C), mRNA. | -0,52105 | 11,94077 | -7,35606 | 0,00003 | 0,00386 |
| ATP5J2 | Homo sapiens ATP synthase, H+ transporting, mitochondrial F0 complex, subunit F2 (ATP5J2), nuclear gene encoding mitochondrial protein, transcript variant 3, mRNA. | -0,52106 | 11,30074 | -4,50981 | 0,00126 | 0,02074 |
| ENAH | Homo sapiens enabled homolog (Drosophila) (ENAH), transcript variant 2, mRNA. | -0,52175 | 7,69326 | -5,95796 | 0,00017 | 0,00770 |
| PSMA3 | Homo sapiens proteasome (prosome, macropain) subunit, alpha type, 3 (PSMA3), transcript variant 2, mRNA. | -0,52223 | 10,39514 | -4,73004 | 0,00091 | 0,01751 |
| TMEM19 | Homo sapiens transmembrane protein 19 (TMEM19), mRNA. | -0,52342 | 7,93731 | -4,46200 | 0,00136 | 0,02167 |
| CKB | Homo sapiens creatine kinase, brain (CKB), mRNA. | -0,52364 | 8,29065 | -3,78535 | 0,00387 | 0,03924 |
| ATP5L | Homo sapiens ATP synthase, H+ transporting, mitochondrial F0 complex, subunit G (ATP5L), nuclear gene encoding mitochondrial protein, mRNA. | -0,52424 | 12,13360 | -6,67717 | 0,00007 | 0,00503 |
| MRPL18 | Homo sapiens mitochondrial ribosomal protein L18 (MRPL18), nuclear gene encoding mitochondrial protein, mRNA. | -0,52430 | 10,48050 | -4,69277 | 0,00096 | 0,01788 |
| MRPS7 | Homo sapiens mitochondrial ribosomal protein S7 (MRPS7), nuclear gene encoding mitochondrial protein, mRNA. | -0,52467 | 9,11448 | -3,96370 | 0,00292 | 0,03329 |
| PHB2 | Homo sapiens prohibitin 2 (PHB2), transcript variant 2, mRNA. | -0,52504 | 10,29162 | -4,57473 | 0,00115 | 0,01980 |
| CCT3 | Homo sapiens chaperonin containing TCP1, subunit 3 (gamma) (CCT3), transcript variant 3, mRNA. | -0,52562 | 9,56588 | -5,07175 | 0,00056 | 0,01358 |
| C3orf21 | Homo sapiens chromosome 3 open reading frame 21 (C3orf21), mRNA. | -0,52589 | 9,71765 | -4,52696 | 0,00123 | 0,02048 |
| SCO1 | Homo sapiens SCO cytochrome oxidase deficient homolog 1 (yeast) (SCO1), nuclear gene encoding mitochondrial protein, mRNA. | -0,52702 | 9,29943 | -5,16403 | 0,00049 | 0,01269 |
| LOC100132992 | PREDICTED: Homo sapiens misc_RNA (LOC100132992), miscRNA. | -0,52717 | 10,28213 | -5,26855 | 0,00042 | 0,01180 |
| RPL29 | Homo sapiens ribosomal protein L29 (RPL29), mRNA. | -0,52732 | 9,47564 | -4,08018 | 0,00243 | 0,02995 |
| LOC730740 | PREDICTED: Homo sapiens similar to H3 histone, family 3B (LOC730740), mRNA. | -0,52779 | 11,06363 | -5,12655 | 0,00052 | 0,01303 |
| PSRC1 | Homo sapiens proline/serine-rich coiled-coil 1 (PSRC1), transcript variant 3, mRNA. | -0,52796 | 7,83723 | -5,09848 | 0,00054 | 0,01333 |
| RUVBL2 | Homo sapiens RuvB-like 2 (E. coli) (RUVBL2), mRNA. | -0,52822 | 9,38623 | -4,04914 | 0,00255 | 0,03083 |
| SNRNP70 | Homo sapiens small nuclear ribonucleoprotein 70kDa (U1) (SNRNP70), mRNA. | -0,52868 | 9,67847 | -6,91063 | 0,00005 | 0,00458 |
| LOC100130932 | PREDICTED: Homo sapiens similar to Sm protein G (LOC100130932), mRNA. | -0,53002 | 8,79679 | -4,33588 | 0,00164 | 0,02404 |
| LOC642946 | PREDICTED: Homo sapiens hypothetical protein LOC642945, transcript variant 2 (LOC642946), mRNA. | -0,53032 | 7,87002 | -4,28089 | 0,00179 | 0,02513 |
| LOC440043 | PREDICTED: Homo sapiens misc_RNA (LOC440043), miscRNA. | -0,53135 | 12,37151 | -5,61437 | 0,00026 | 0,00937 |
| BCLAF1 | Homo sapiens BCL2-associated transcription factor 1 (BCLAF1), transcript variant 2, mRNA. | -0,53147 | 9,20611 | -3,77395 | 0,00394 | 0,03968 |
| ANKRD22 | Homo sapiens ankyrin repeat domain 22 (ANKRD22), mRNA. | -0,53230 | 7,83724 | -4,32172 | 0,00168 | 0,02433 |
| PGAM5 | PREDICTED: Homo sapiens phosphoglycerate mutase family member 5 (PGAM5), mRNA. | -0,53238 | 8,45832 | -4,87869 | 0,00073 | 0,01579 |
| CSTF2 | Homo sapiens cleavage stimulation factor, 3' pre-RNA, subunit 2, 64kDa (CSTF2), mRNA. | -0,53255 | 9,11072 | -4,94302 | 0,00067 | 0,01506 |
| SREBF1 | Homo sapiens sterol regulatory element binding transcription factor 1 (SREBF1), transcript variant 1, mRNA. | -0,53261 | 8,13554 | -4,29643 | 0,00174 | 0,02487 |
| RPS15A | Homo sapiens ribosomal protein S15a (RPS15A), transcript variant 2, mRNA. | -0,53266 | 10,14973 | -6,10981 | 0,00014 | 0,00709 |
| LOC375295 | PREDICTED: Homo sapiens hypothetical gene supported by BC013438 (LOC375295), mRNA. | -0,53267 | 8,40695 | -4,41912 | 0,00145 | 0,02247 |
| C19orf48 | Homo sapiens chromosome 19 open reading frame 48 (C19orf48), mRNA. | -0,53297 | 9,69982 | -4,26208 | 0,00184 | 0,02558 |
| BCCIP | Homo sapiens BRCA2 and CDKN1A interacting protein (BCCIP), transcript variant A, mRNA. | -0,53305 | 9,29349 | -4,83861 | 0,00078 | 0,01617 |
| TSR2 | Homo sapiens TSR2, 20S rRNA accumulation, homolog (S. cerevisiae) (TSR2), mRNA. | -0,53330 | 9,16247 | -5,37639 | 0,00036 | 0,01111 |
| USP13 | Homo sapiens ubiquitin specific peptidase 13 (isopeptidase T-3) (USP13), mRNA. | -0,53380 | 7,96105 | -4,63255 | 0,00105 | 0,01887 |
| DNCL1 | Homo sapiens dynein, cytoplasmic, light polypeptide 1 (DNCL1), mRNA. | -0,53478 | 12,46739 | -3,99788 | 0,00277 | 0,03230 |
| SNX27 | Homo sapiens sorting nexin family member 27 (SNX27), mRNA. | -0,53480 | 9,01417 | -5,01029 | 0,00061 | 0,01424 |
| EXOSC2 | Homo sapiens exosome component 2 (EXOSC2), mRNA. | -0,53489 | 8,01726 | -4,33657 | 0,00164 | 0,02403 |
| COPS7B | Homo sapiens COP9 constitutive photomorphogenic homolog subunit 7B (Arabidopsis) (COPS7B), mRNA. | -0,53500 | 8,47046 | -4,01937 | 0,00268 | 0,03171 |
| DDX18 | Homo sapiens DEAD (Asp-Glu-Ala-Asp) box polypeptide 18 (DDX18), mRNA. | -0,53563 | 10,13302 | -4,82185 | 0,00080 | 0,01634 |
| CCDC72 | Homo sapiens coiled-coil domain containing 72 (CCDC72), mRNA. | -0,53636 | 11,65020 | -4,68372 | 0,00098 | 0,01799 |
| SAP30 | Homo sapiens Sin3A-associated protein, 30kDa (SAP30), mRNA. | -0,53853 | 7,73559 | -7,46620 | 0,00003 | 0,00368 |
| ORC1L | Homo sapiens origin recognition complex, subunit 1-like (yeast) (ORC1L), mRNA. | -0,53874 | 7,61484 | -7,70739 | 0,00002 | 0,00332 |
| EXOSC5 | Homo sapiens exosome component 5 (EXOSC5), mRNA. | -0,53880 | 8,18919 | -3,77860 | 0,00392 | 0,03954 |
| DNAJC9 | Homo sapiens DnaJ (Hsp40) homolog, subfamily C, member 9 (DNAJC9), mRNA. | -0,53916 | 10,22098 | -3,77087 | 0,00396 | 0,03983 |
| ZNF618 | Homo sapiens zinc finger protein 618 (ZNF618), mRNA. | -0,53965 | 7,70870 | -8,89029 | 0,00001 | 0,00198 |
| ZC3HC1 | Homo sapiens zinc finger, C3HC-type containing 1 (ZC3HC1), mRNA. | -0,53973 | 8,54384 | -6,81063 | 0,00006 | 0,00479 |
| IRX2 | Homo sapiens iroquois homeobox 2 (IRX2), mRNA. | -0,53979 | 8,99798 | -4,99050 | 0,00063 | 0,01446 |
| UQCRB | Homo sapiens ubiquinol-cytochrome c reductase binding protein (UQCRB), mRNA. | -0,53998 | 9,47631 | -4,06898 | 0,00248 | 0,03027 |
| C1QTNF6 | Homo sapiens C1q and tumor necrosis factor related protein 6 (C1QTNF6), transcript variant 1, mRNA. | -0,54017 | 7,97552 | -4,11041 | 0,00232 | 0,02904 |
| LSM2 | Homo sapiens LSM2 homolog, U6 small nuclear RNA associated (S. cerevisiae) (LSM2), mRNA. | -0,54201 | 9,86245 | -4,34017 | 0,00163 | 0,02401 |
| SNRPA1 | Homo sapiens small nuclear ribonucleoprotein polypeptide A' (SNRPA1), mRNA. | -0,54256 | 10,86742 | -4,99910 | 0,00062 | 0,01436 |
| API5 | Homo sapiens apoptosis inhibitor 5 (API5), mRNA. | -0,54352 | 9,50344 | -3,65346 | 0,00479 | 0,04465 |
| ZNF462 | Homo sapiens zinc finger protein 462 (ZNF462), mRNA. | -0,54385 | 8,01841 | -4,23347 | 0,00192 | 0,02615 |
| TMEM177 | Homo sapiens transmembrane protein 177 (TMEM177), mRNA. | -0,54397 | 8,26651 | -4,41775 | 0,00145 | 0,02247 |
| LOC440957 | Homo sapiens similar to CG32736-PA (LOC440957), mRNA. | -0,54407 | 8,25223 | -4,75343 | 0,00088 | 0,01721 |
| ATP5J | Homo sapiens ATP synthase, H+ transporting, mitochondrial F0 complex, subunit F6 (ATP5J), nuclear gene encoding mitochondrial protein, transcript variant 1, mRNA. | -0,54562 | 8,56520 | -5,01698 | 0,00060 | 0,01422 |
| CHAF1B | Homo sapiens chromatin assembly factor 1, subunit B (p60) (CHAF1B), mRNA. | -0,54700 | 7,82196 | -6,21213 | 0,00012 | 0,00683 |
| NOP56 | Homo sapiens NOP56 ribonucleoprotein homolog (yeast) (NOP56), transcript variant 1, mRNA. | -0,54757 | 9,01353 | -3,71071 | 0,00436 | 0,04217 |
| CCDC5 | Homo sapiens coiled-coil domain containing 5 (spindle associated) (CCDC5), mRNA. | -0,54829 | 8,43252 | -4,40011 | 0,00149 | 0,02278 |
| EZH2 | Homo sapiens enhancer of zeste homolog 2 (Drosophila) (EZH2), transcript variant 2, mRNA. | -0,54927 | 7,80632 | -4,85202 | 0,00076 | 0,01607 |
| CEP78 | Homo sapiens centrosomal protein 78kDa (CEP78), transcript variant 2, mRNA. | -0,54977 | 7,85550 | -3,90533 | 0,00320 | 0,03513 |
| TUBG1 | Homo sapiens tubulin, gamma 1 (TUBG1), mRNA. | -0,55059 | 8,85517 | -3,66313 | 0,00471 | 0,04417 |
| BRCA1 | Homo sapiens breast cancer 1, early onset (BRCA1), transcript variant BRCA1-delta14-17, mRNA. | -0,55159 | 7,78842 | -5,33213 | 0,00039 | 0,01132 |
| CEBPA | Homo sapiens CCAAT/enhancer binding protein (C/EBP), alpha (CEBPA), mRNA. | -0,55202 | 7,85803 | -4,34999 | 0,00161 | 0,02383 |
| NDUFV2 | Homo sapiens NADH dehydrogenase (ubiquinone) flavoprotein 2, 24kDa (NDUFV2), mRNA. | -0,55229 | 11,87746 | -4,09205 | 0,00239 | 0,02961 |
| BID | Homo sapiens BH3 interacting domain death agonist (BID), transcript variant 1, mRNA. | -0,55263 | 8,16570 | -5,14296 | 0,00050 | 0,01289 |
| CHPT1 | Homo sapiens choline phosphotransferase 1 (CHPT1), mRNA. | -0,55275 | 8,62669 | -3,87700 | 0,00335 | 0,03597 |
| UPF3B | Homo sapiens UPF3 regulator of nonsense transcripts homolog B (yeast) (UPF3B), transcript variant 2, mRNA. | -0,55370 | 9,11009 | -6,04681 | 0,00015 | 0,00728 |
| CCNJL | Homo sapiens cyclin J-like (CCNJL), mRNA. | -0,55424 | 7,93508 | -7,59874 | 0,00002 | 0,00342 |
| ATP5D | Homo sapiens ATP synthase, H+ transporting, mitochondrial F1 complex, delta subunit (ATP5D), nuclear gene encoding mitochondrial protein, transcript variant 1, mRNA. | -0,55442 | 10,31021 | -5,42607 | 0,00034 | 0,01068 |
| U2AF2 | Homo sapiens U2 small nuclear RNA auxiliary factor 2 (U2AF2), transcript variant 1, mRNA. | -0,55524 | 10,71060 | -4,86064 | 0,00075 | 0,01593 |
| ATP5A1 | Homo sapiens ATP synthase, H+ transporting, mitochondrial F1 complex, alpha subunit 1, cardiac muscle (ATP5A1), nuclear gene encoding mitochondrial protein, transcript variant 2, mRNA. | -0,55656 | 12,30799 | -3,78269 | 0,00389 | 0,03936 |
| NIF3L1 | Homo sapiens NIF3 NGG1 interacting factor 3-like 1 (S. pombe) (NIF3L1), mRNA. | -0,55693 | 9,81067 | -3,79180 | 0,00383 | 0,03898 |
| RPL34 | Homo sapiens ribosomal protein L34 (RPL34), transcript variant 2, mRNA. | -0,55814 | 9,85897 | -3,71706 | 0,00432 | 0,04191 |
| LOC347376 | PREDICTED: Homo sapiens similar to H3 histone, family 3B (LOC347376), mRNA. | -0,55952 | 8,42944 | -6,39385 | 0,00010 | 0,00614 |
| EYA2 | Homo sapiens eyes absent homolog 2 (Drosophila) (EYA2), transcript variant 4, mRNA. | -0,55959 | 7,59254 | -6,19182 | 0,00012 | 0,00686 |
| MRPL13 | Homo sapiens mitochondrial ribosomal protein L13 (MRPL13), nuclear gene encoding mitochondrial protein, mRNA. | -0,56086 | 10,04978 | -3,59727 | 0,00524 | 0,04710 |
| KCTD3 | Homo sapiens potassium channel tetramerisation domain containing 3 (KCTD3), mRNA. | -0,56089 | 9,02513 | -6,24546 | 0,00012 | 0,00667 |
| KATNB1 | Homo sapiens katanin p80 (WD repeat containing) subunit B 1 (KATNB1), mRNA. | -0,56194 | 8,28304 | -4,23578 | 0,00191 | 0,02613 |
| HSPA4 | Homo sapiens heat shock 70kDa protein 4 (HSPA4), mRNA. | -0,56218 | 7,74297 | -4,63855 | 0,00104 | 0,01877 |
| FAT2 | Homo sapiens FAT tumor suppressor homolog 2 (Drosophila) (FAT2), mRNA. | -0,56268 | 7,62119 | -3,98909 | 0,00281 | 0,03254 |
| NQO1 | Homo sapiens NAD(P)H dehydrogenase, quinone 1 (NQO1), transcript variant 1, mRNA. | -0,56280 | 10,68582 | -4,84648 | 0,00077 | 0,01612 |
| CXorf57 | Homo sapiens chromosome X open reading frame 57 (CXorf57), mRNA. | -0,56290 | 7,99274 | -4,55999 | 0,00117 | 0,02000 |
| THOC7 | Homo sapiens THO complex 7 homolog (Drosophila) (THOC7), mRNA. | -0,56295 | 10,74189 | -3,55335 | 0,00563 | 0,04909 |
| BCL11A | Homo sapiens B-cell CLL/lymphoma 11A (zinc finger protein) (BCL11A), transcript variant 1, mRNA. | -0,56300 | 7,65287 | -5,33722 | 0,00038 | 0,01132 |
| NCLN | Homo sapiens nicalin homolog (zebrafish) (NCLN), mRNA. | -0,56462 | 8,98571 | -3,83722 | 0,00357 | 0,03734 |
| CSTF3 | Homo sapiens cleavage stimulation factor, 3' pre-RNA, subunit 3, 77kDa (CSTF3), transcript variant 1, mRNA. | -0,56480 | 8,62604 | -3,80103 | 0,00378 | 0,03866 |
| PSMA3 | Homo sapiens proteasome (prosome, macropain) subunit, alpha type, 3 (PSMA3), transcript variant 1, mRNA. | -0,56482 | 9,18066 | -4,78435 | 0,00084 | 0,01686 |
| RBM38 | Homo sapiens RNA binding motif protein 38 (RBM38), transcript variant 2, mRNA. | -0,56602 | 8,33731 | -4,52411 | 0,00124 | 0,02054 |
| MRPL39 | Homo sapiens mitochondrial ribosomal protein L39 (MRPL39), nuclear gene encoding mitochondrial protein, transcript variant 1, mRNA. | -0,56607 | 9,17804 | -4,46893 | 0,00134 | 0,02154 |
| LOC728969 | PREDICTED: Homo sapiens similar to heparan sulfate 6-O-sulfotransferase 1 (LOC728969), mRNA. | -0,56688 | 7,93088 | -7,44160 | 0,00003 | 0,00368 |
| ZNF323 | Homo sapiens zinc finger protein 323 (ZNF323), transcript variant 2, mRNA. | -0,56813 | 7,47286 | -8,99115 | 0,00001 | 0,00194 |
| ATP5J | Homo sapiens ATP synthase, H+ transporting, mitochondrial F0 complex, subunit F6 (ATP5J), nuclear gene encoding mitochondrial protein, transcript variant 5, mRNA. | -0,56870 | 11,13185 | -4,82192 | 0,00080 | 0,01634 |
| MRPL37 | Homo sapiens mitochondrial ribosomal protein L37 (MRPL37), nuclear gene encoding mitochondrial protein, mRNA. | -0,56884 | 10,88028 | -3,71460 | 0,00434 | 0,04202 |
| THOP1 | Homo sapiens thimet oligopeptidase 1 (THOP1), mRNA. | -0,56937 | 8,59027 | -5,29472 | 0,00041 | 0,01161 |
| LOC93622 | PREDICTED: Homo sapiens hypothetical protein BC006130 (LOC93622), misc RNA. | -0,56998 | 8,06547 | -6,84916 | 0,00006 | 0,00477 |
| PCNA | Homo sapiens proliferating cell nuclear antigen (PCNA), transcript variant 2, mRNA. | -0,57082 | 7,81082 | -4,84712 | 0,00077 | 0,01612 |
| MRPL35 | Homo sapiens mitochondrial ribosomal protein L35 (MRPL35), nuclear gene encoding mitochondrial protein, transcript variant 1, mRNA. | -0,57426 | 8,40257 | -4,30785 | 0,00171 | 0,02462 |
| FAM195A | Homo sapiens family with sequence similarity 195, member A (FAM195A), mRNA. | -0,57474 | 8,55202 | -5,25405 | 0,00043 | 0,01192 |
| OBFC2B | Homo sapiens oligonucleotide/oligosaccharide-binding fold containing 2B (OBFC2B), mRNA. | -0,57560 | 8,53994 | -5,76210 | 0,00022 | 0,00858 |
| MRPS34 | Homo sapiens mitochondrial ribosomal protein S34 (MRPS34), nuclear gene encoding mitochondrial protein, mRNA. | -0,57575 | 8,31676 | -4,76729 | 0,00086 | 0,01709 |
| WDR77 | Homo sapiens WD repeat domain 77 (WDR77), mRNA. | -0,57680 | 8,16191 | -4,47344 | 0,00133 | 0,02150 |
| BARD1 | Homo sapiens BRCA1 associated RING domain 1 (BARD1), mRNA. | -0,57685 | 7,95591 | -4,22141 | 0,00196 | 0,02637 |
| FAM96A | Homo sapiens family with sequence similarity 96, member A (FAM96A), transcript variant 2, mRNA. | -0,57685 | 8,67895 | -5,56088 | 0,00028 | 0,00982 |
| BCL11A | Homo sapiens B-cell CLL/lymphoma 11A (zinc finger protein) (BCL11A), transcript variant 1, mRNA. | -0,57753 | 7,58714 | -6,40218 | 0,00010 | 0,00612 |
| DCTN3 | Homo sapiens dynactin 3 (p22) (DCTN3), transcript variant 1, mRNA. | -0,57786 | 9,37465 | -3,93064 | 0,00308 | 0,03416 |
| GOT2 | Homo sapiens glutamic-oxaloacetic transaminase 2, mitochondrial (aspartate aminotransferase 2) (GOT2), nuclear gene encoding mitochondrial protein, mRNA. | -0,57848 | 11,23080 | -4,85011 | 0,00077 | 0,01608 |
| SUMO3 | Homo sapiens SMT3 suppressor of mif two 3 homolog 3 (S. cerevisiae) (SUMO3), mRNA. | -0,57855 | 11,30680 | -6,72244 | 0,00006 | 0,00492 |
| ATP5J2 | Homo sapiens ATP synthase, H+ transporting, mitochondrial F0 complex, subunit F2 (ATP5J2), nuclear gene encoding mitochondrial protein, transcript variant 1, mRNA. | -0,57981 | 11,35316 | -5,00349 | 0,00061 | 0,01435 |
| LSMD1 | Homo sapiens LSM domain containing 1 (LSMD1), mRNA. | -0,58051 | 8,40291 | -5,15502 | 0,00050 | 0,01272 |
| HAUS8 | Homo sapiens HAUS augmin-like complex, subunit 8 (HAUS8), transcript variant 1, mRNA. | -0,58102 | 7,80148 | -5,27063 | 0,00042 | 0,01179 |
| HMGB1L1 | Homo sapiens high-mobility group box 1-like 1 (HMGB1L1), mRNA. | -0,58264 | 8,69036 | -3,97021 | 0,00289 | 0,03309 |
| SMC4 | Homo sapiens structural maintenance of chromosomes 4 (SMC4), transcript variant 2, mRNA. | -0,58347 | 7,95734 | -4,72707 | 0,00092 | 0,01754 |
| OAT | Homo sapiens ornithine aminotransferase (gyrate atrophy) (OAT), nuclear gene encoding mitochondrial protein, mRNA. | -0,58348 | 10,85422 | -4,12041 | 0,00229 | 0,02879 |
| HELLS | Homo sapiens helicase, lymphoid-specific (HELLS), mRNA. | -0,58363 | 7,71852 | -5,83773 | 0,00020 | 0,00817 |
| FBL | Homo sapiens fibrillarin (FBL), mRNA. | -0,58369 | 10,45820 | -5,80648 | 0,00020 | 0,00829 |
| COBLL1 | Homo sapiens COBL-like 1 (COBLL1), mRNA. | -0,58393 | 7,97538 | -4,97369 | 0,00064 | 0,01468 |
| FN3KRP | Homo sapiens fructosamine-3-kinase-related protein (FN3KRP), mRNA. | -0,58438 | 9,30877 | -5,09180 | 0,00054 | 0,01341 |
| BCHE | Homo sapiens butyrylcholinesterase (BCHE), mRNA. | -0,58487 | 7,68885 | -4,56480 | 0,00116 | 0,01991 |
| TMEM39B | Homo sapiens transmembrane protein 39B (TMEM39B), mRNA. | -0,58494 | 8,55520 | -5,84403 | 0,00019 | 0,00812 |
| LOC442727 | PREDICTED: Homo sapiens misc_RNA (LOC442727), miscRNA. | -0,58495 | 8,02037 | -4,22928 | 0,00193 | 0,02626 |
| DPH5 | Homo sapiens DPH5 homolog (S. cerevisiae) (DPH5), transcript variant 1, mRNA. | -0,58560 | 8,72886 | -3,95283 | 0,00297 | 0,03355 |
| AKR7A2 | Homo sapiens aldo-keto reductase family 7, member A2 (aflatoxin aldehyde reductase) (AKR7A2), mRNA. | -0,58582 | 10,37901 | -5,15680 | 0,00049 | 0,01271 |
| CSTF3 | Homo sapiens cleavage stimulation factor, 3' pre-RNA, subunit 3, 77kDa (CSTF3), transcript variant 2, mRNA. | -0,58618 | 8,15268 | -6,63441 | 0,00007 | 0,00522 |
| HSPD1 | Homo sapiens heat shock 60kDa protein 1 (chaperonin) (HSPD1), nuclear gene encoding mitochondrial protein, transcript variant 1, mRNA. | -0,58707 | 7,92375 | -5,45474 | 0,00033 | 0,01050 |
| SLC2A8 | Homo sapiens solute carrier family 2 (facilitated glucose transporter), member 8 (SLC2A8), mRNA. | -0,58732 | 8,45418 | -5,12503 | 0,00052 | 0,01303 |
| SDHD | Homo sapiens succinate dehydrogenase complex, subunit D, integral membrane protein (SDHD), nuclear gene encoding mitochondrial protein, mRNA. | -0,58751 | 9,18758 | -3,55943 | 0,00557 | 0,04881 |
| TUBG1 | Homo sapiens tubulin, gamma 1 (TUBG1), mRNA. | -0,58774 | 9,97903 | -3,75950 | 0,00404 | 0,04019 |
| MRRF | Homo sapiens mitochondrial ribosome recycling factor (MRRF), nuclear gene encoding mitochondrial protein, transcript variant 2, mRNA. | -0,58806 | 8,61611 | -5,84990 | 0,00019 | 0,00809 |
| RNASEH1 | Homo sapiens ribonuclease H1 (RNASEH1), mRNA. | -0,58825 | 9,64500 | -4,56793 | 0,00116 | 0,01990 |
| MRPL23 | Homo sapiens mitochondrial ribosomal protein L23 (MRPL23), nuclear gene encoding mitochondrial protein, mRNA. | -0,58897 | 9,94335 | -6,33972 | 0,00010 | 0,00627 |
| PHF19 | Homo sapiens PHD finger protein 19 (PHF19), transcript variant 2, mRNA. | -0,58929 | 8,31296 | -5,63034 | 0,00026 | 0,00933 |
| BRCA1 | Homo sapiens breast cancer 1, early onset (BRCA1), transcript variant BRCA1-delta11b, mRNA. | -0,58930 | 7,63589 | -5,38922 | 0,00036 | 0,01102 |
| LOC647302 | PREDICTED: Homo sapiens misc_RNA (LOC647302), miscRNA. | -0,58994 | 10,27928 | -4,01745 | 0,00268 | 0,03172 |
| CCDC58 | Homo sapiens coiled-coil domain containing 58 (CCDC58), mRNA. | -0,59012 | 7,86655 | -4,22238 | 0,00195 | 0,02637 |
| LOC644877 | PREDICTED: Homo sapiens misc_RNA (LOC644877), miscRNA. | -0,59048 | 8,82363 | -6,08887 | 0,00014 | 0,00713 |
| TSEN34 | Homo sapiens tRNA splicing endonuclease 34 homolog (S. cerevisiae) (TSEN34), transcript variant 2, mRNA. | -0,59132 | 9,73790 | -5,01126 | 0,00061 | 0,01424 |
| FAM53B | Homo sapiens family with sequence similarity 53, member B (FAM53B), mRNA. | -0,59156 | 7,91330 | -5,16820 | 0,00049 | 0,01266 |
| C1orf112 | Homo sapiens chromosome 1 open reading frame 112 (C1orf112), mRNA. | -0,59256 | 7,86906 | -3,88077 | 0,00333 | 0,03586 |
| TMEM48 | Homo sapiens transmembrane protein 48 (TMEM48), mRNA. | -0,59286 | 8,34394 | -5,62546 | 0,00026 | 0,00933 |
| LOC100130003 | PREDICTED: Homo sapiens misc_RNA (LOC100130003), miscRNA. | -0,59469 | 11,89993 | -4,35931 | 0,00158 | 0,02365 |
| TYSND1 | Homo sapiens trypsin domain containing 1 (TYSND1), transcript variant 2, mRNA. | -0,59480 | 9,04122 | -4,08986 | 0,00240 | 0,02966 |
| THYN1 | Homo sapiens thymocyte nuclear protein 1 (THYN1), transcript variant 2, mRNA. | -0,59480 | 8,27865 | -4,27375 | 0,00181 | 0,02531 |
| HSPA2 | Homo sapiens heat shock 70kDa protein 2 (HSPA2), mRNA. | -0,59557 | 7,60949 | -10,37339 | 0,00000 | 0,00115 |
| MXRA5 | Homo sapiens matrix-remodelling associated 5 (MXRA5), mRNA. | -0,59633 | 7,62278 | -4,67083 | 0,00099 | 0,01817 |
| LOC653884 | PREDICTED: Homo sapiens similar to FUS interacting protein (serine-arginine rich) 1 (LOC653884), mRNA. | -0,59653 | 8,93231 | -4,79102 | 0,00083 | 0,01675 |
| MRTO4 | Homo sapiens mRNA turnover 4 homolog (S. cerevisiae) (MRTO4), mRNA. | -0,59688 | 8,50449 | -3,96900 | 0,00290 | 0,03311 |
| NUDCD2 | Homo sapiens NudC domain containing 2 (NUDCD2), mRNA. | -0,59714 | 9,11936 | -3,98915 | 0,00281 | 0,03254 |
| NDUFA6 | Homo sapiens NADH dehydrogenase (ubiquinone) 1 alpha subcomplex, 6, 14kDa (NDUFA6), nuclear gene encoding mitochondrial protein, mRNA. | -0,59888 | 8,55817 | -5,46503 | 0,00032 | 0,01044 |
| LYPD1 | Homo sapiens LY6/PLAUR domain containing 1 (LYPD1), transcript variant 2, mRNA. | -0,60008 | 7,57989 | -7,01038 | 0,00005 | 0,00442 |
| LOC100134304 | PREDICTED: Homo sapiens similar to hCG1983233 (LOC100134304), mRNA. | -0,60096 | 8,00773 | -3,61655 | 0,00508 | 0,04622 |
| LOC100132457 | PREDICTED: Homo sapiens similar to Sm protein G (LOC100132457), mRNA. | -0,60168 | 10,73169 | -4,69724 | 0,00096 | 0,01781 |
| RECQL4 | Homo sapiens RecQ protein-like 4 (RECQL4), mRNA. | -0,60268 | 7,99778 | -3,92893 | 0,00308 | 0,03420 |
| PFKM | Homo sapiens phosphofructokinase, muscle (PFKM), mRNA. | -0,60279 | 8,08899 | -3,76089 | 0,00403 | 0,04016 |
| MRPL20 | Homo sapiens mitochondrial ribosomal protein L20 (MRPL20), nuclear gene encoding mitochondrial protein, mRNA. | -0,60352 | 9,13700 | -3,72897 | 0,00424 | 0,04154 |
| NOL11 | Homo sapiens nucleolar protein 11 (NOL11), mRNA. | -0,60382 | 9,60223 | -4,82952 | 0,00079 | 0,01625 |
| NUP35 | Homo sapiens nucleoporin 35kDa (NUP35), mRNA. | -0,60454 | 8,36319 | -3,92706 | 0,00309 | 0,03427 |
| DNAJA1 | Homo sapiens DnaJ (Hsp40) homolog, subfamily A, member 1 (DNAJA1), mRNA. | -0,60625 | 11,63228 | -3,85680 | 0,00346 | 0,03676 |
| DCXR | Homo sapiens dicarbonyl/L-xylulose reductase (DCXR), mRNA. | -0,60872 | 8,46724 | -4,15830 | 0,00216 | 0,02776 |
| SNRPG | Homo sapiens small nuclear ribonucleoprotein polypeptide G (SNRPG), mRNA. | -0,60872 | 12,32217 | -4,22196 | 0,00195 | 0,02637 |
| AIFM1 | Homo sapiens apoptosis-inducing factor, mitochondrion-associated, 1 (AIFM1), nuclear gene encoding mitochondrial protein, transcript variant 3, mRNA. | -0,60911 | 8,98728 | -4,60107 | 0,00110 | 0,01941 |
| RAB3IP | Homo sapiens RAB3A interacting protein (rabin3) (RAB3IP), transcript variant A, mRNA. | -0,60970 | 9,31797 | -5,29640 | 0,00041 | 0,01161 |
| SUMO2 | Homo sapiens SMT3 suppressor of mif two 3 homolog 2 (S. cerevisiae) (SUMO2), transcript variant 2, mRNA. | -0,60976 | 11,04599 | -4,86803 | 0,00075 | 0,01585 |
| C15orf23 | Homo sapiens chromosome 15 open reading frame 23 (C15orf23), mRNA. | -0,60979 | 8,15180 | -3,54548 | 0,00570 | 0,04950 |
| H3F3A | Homo sapiens H3 histone, family 3A (H3F3A), mRNA. | -0,61299 | 12,31687 | -4,96461 | 0,00065 | 0,01476 |
| FAM173B | Homo sapiens family with sequence similarity 173, member B (FAM173B), mRNA. | -0,61308 | 7,93199 | -4,32784 | 0,00166 | 0,02424 |
| RAD54L | Homo sapiens RAD54-like (S. cerevisiae) (RAD54L), mRNA. | -0,61361 | 7,92633 | -3,73751 | 0,00418 | 0,04114 |
| PGAM4 | Homo sapiens phosphoglycerate mutase family member 4 (PGAM4), mRNA. | -0,61433 | 11,10824 | -7,65792 | 0,00002 | 0,00334 |
| WBP11 | Homo sapiens WW domain binding protein 11 (WBP11), mRNA. | -0,61470 | 10,45397 | -5,12754 | 0,00051 | 0,01303 |
| JAG2 | Homo sapiens jagged 2 (JAG2), transcript variant 2, mRNA. | -0,61695 | 7,98162 | -5,17018 | 0,00049 | 0,01265 |
| XPO1 | Homo sapiens exportin 1 (CRM1 homolog, yeast) (XPO1), mRNA. | -0,61720 | 9,92675 | -4,17526 | 0,00210 | 0,02734 |
| CDCA4 | Homo sapiens cell division cycle associated 4 (CDCA4), transcript variant 13, mRNA. | -0,61755 | 8,76304 | -4,18249 | 0,00208 | 0,02718 |
| ISOC1 | Homo sapiens isochorismatase domain containing 1 (ISOC1), mRNA. | -0,61805 | 8,51438 | -4,52096 | 0,00124 | 0,02059 |
| M6PR | Homo sapiens mannose-6-phosphate receptor (cation dependent) (M6PR), mRNA. | -0,61832 | 9,49905 | -5,79910 | 0,00021 | 0,00836 |
| NDUFB10 | Homo sapiens NADH dehydrogenase (ubiquinone) 1 beta subcomplex, 10, 22kDa (NDUFB10), mRNA. | -0,62022 | 10,18831 | -3,77398 | 0,00394 | 0,03968 |
| S1PR5 | Homo sapiens sphingosine-1-phosphate receptor 5 (S1PR5), mRNA. | -0,62183 | 7,56707 | -5,22748 | 0,00045 | 0,01212 |
| NUP107 | Homo sapiens nucleoporin 107kDa (NUP107), mRNA. | -0,62210 | 8,71600 | -3,61299 | 0,00511 | 0,04637 |
| RFC3 | Homo sapiens replication factor C (activator 1) 3, 38kDa (RFC3), transcript variant 1, mRNA. | -0,62285 | 7,84928 | -4,88782 | 0,00072 | 0,01572 |
| KCNMB4 | Homo sapiens potassium large conductance calcium-activated channel, subfamily M, beta member 4 (KCNMB4), mRNA. | -0,62290 | 7,63134 | -5,57252 | 0,00028 | 0,00973 |
| GPR177 | Homo sapiens G protein-coupled receptor 177 (GPR177), transcript variant 2, mRNA. | -0,62332 | 8,23444 | -6,38716 | 0,00010 | 0,00614 |
| ANXA8L2 | Homo sapiens annexin A8-like 2 (ANXA8L2), mRNA. | -0,62424 | 9,20347 | -4,27783 | 0,00179 | 0,02520 |
| PPP1CC | Homo sapiens protein phosphatase 1, catalytic subunit, gamma isoform (PPP1CC), mRNA. | -0,62501 | 11,13216 | -4,96204 | 0,00065 | 0,01479 |
| MRPS28 | Homo sapiens mitochondrial ribosomal protein S28 (MRPS28), nuclear gene encoding mitochondrial protein, mRNA. | -0,62599 | 9,09280 | -4,67328 | 0,00099 | 0,01816 |
| RTN4IP1 | Homo sapiens reticulon 4 interacting protein 1 (RTN4IP1), nuclear gene encoding mitochondrial protein, mRNA. | -0,62653 | 8,59961 | -5,47400 | 0,00032 | 0,01036 |
| LTV1 | Homo sapiens LTV1 homolog (S. cerevisiae) (LTV1), mRNA. | -0,62666 | 9,96951 | -5,09590 | 0,00054 | 0,01336 |
| LOC644877 | PREDICTED: Homo sapiens misc_RNA (LOC644877), miscRNA. | -0,62678 | 8,37810 | -6,72755 | 0,00006 | 0,00490 |
| TFAM | Homo sapiens transcription factor A, mitochondrial (TFAM), nuclear gene encoding mitochondrial protein, mRNA. | -0,62756 | 8,37837 | -5,45096 | 0,00033 | 0,01050 |
| CDCA2 | Homo sapiens cell division cycle associated 2 (CDCA2), mRNA. | -0,62825 | 7,76348 | -5,30494 | 0,00040 | 0,01155 |
| CHCHD4 | Homo sapiens coiled-coil-helix-coiled-coil-helix domain containing 4 (CHCHD4), nuclear gene encoding mitochondrial protein, transcript variant 2, mRNA. | -0,62903 | 8,81624 | -3,72605 | 0,00426 | 0,04165 |
| ATP5A1 | Homo sapiens ATP synthase, H+ transporting, mitochondrial F1 complex, alpha subunit 1, cardiac muscle (ATP5A1), nuclear gene encoding mitochondrial protein, transcript variant 2, mRNA. | -0,63007 | 11,95560 | -4,11354 | 0,00231 | 0,02901 |
| BEX1 | Homo sapiens brain expressed, X-linked 1 (BEX1), mRNA. | -0,63046 | 8,08197 | -5,63045 | 0,00026 | 0,00933 |
| PSMB8 | Homo sapiens proteasome (prosome, macropain) subunit, beta type, 8 (large multifunctional peptidase 7) (PSMB8), transcript variant 2, mRNA. | -0,63258 | 8,06829 | -5,89139 | 0,00018 | 0,00788 |
| HSBP1 | Homo sapiens heat shock factor binding protein 1 (HSBP1), mRNA. | -0,63280 | 9,52215 | -8,22583 | 0,00001 | 0,00262 |
| NUP85 | Homo sapiens nucleoporin 85kDa (NUP85), mRNA. | -0,63308 | 9,74109 | -4,62868 | 0,00106 | 0,01894 |
| C19orf48 | Homo sapiens chromosome 19 open reading frame 48 (C19orf48), mRNA. | -0,63366 | 8,87311 | -4,33807 | 0,00164 | 0,02401 |
| HAT1 | Homo sapiens histone acetyltransferase 1 (HAT1), transcript variant 1, mRNA. | -0,63403 | 9,13841 | -3,64480 | 0,00485 | 0,04491 |
| SLC25A3 | Homo sapiens solute carrier family 25 (mitochondrial carrier; phosphate carrier), member 3 (SLC25A3), nuclear gene encoding mitochondrial protein, transcript variant 2, mRNA. | -0,63471 | 11,63195 | -4,99563 | 0,00062 | 0,01438 |
| KHSRP | Homo sapiens KH-type splicing regulatory protein (KHSRP), mRNA. | -0,63530 | 8,97462 | -11,57879 | 0,00000 | 0,00065 |
| HNRNPC | Homo sapiens heterogeneous nuclear ribonucleoprotein C (C1/C2) (HNRNPC), transcript variant 3, mRNA. | -0,63602 | 8,17533 | -4,29525 | 0,00175 | 0,02488 |
| SNHG1 | Homo sapiens small nucleolar RNA host gene 1 (non-protein coding) (SNHG1), non-coding RNA. | -0,63638 | 9,38976 | -4,48755 | 0,00131 | 0,02121 |
| LOC402112 | PREDICTED: Homo sapiens misc_RNA (LOC402112), miscRNA. | -0,63648 | 10,72791 | -3,58818 | 0,00532 | 0,04761 |
| PSMB9 | Homo sapiens proteasome (prosome, macropain) subunit, beta type, 9 (large multifunctional peptidase 2) (PSMB9), transcript variant 1, mRNA. | -0,63742 | 7,77735 | -4,16875 | 0,00212 | 0,02751 |
| TMEM160 | Homo sapiens transmembrane protein 160 (TMEM160), mRNA. | -0,63914 | 9,44461 | -3,70856 | 0,00438 | 0,04223 |
| CENPM | Homo sapiens centromere protein M (CENPM), transcript variant 2, mRNA. | -0,63928 | 7,65125 | -5,99323 | 0,00016 | 0,00747 |
| HMBS | Homo sapiens hydroxymethylbilane synthase (HMBS), transcript variant 1, mRNA. | -0,63983 | 9,22737 | -3,75213 | 0,00408 | 0,04051 |
| ZNF362 | Homo sapiens zinc finger protein 362 (ZNF362), mRNA. | -0,64037 | 7,61957 | -7,37554 | 0,00003 | 0,00382 |
| C15orf23 | Homo sapiens chromosome 15 open reading frame 23 (C15orf23), mRNA. | -0,64065 | 8,06033 | -4,83587 | 0,00078 | 0,01622 |
| CNPY4 | Homo sapiens canopy 4 homolog (zebrafish) (CNPY4), mRNA. | -0,64134 | 8,01316 | -5,48051 | 0,00032 | 0,01030 |
| LOC653226 | PREDICTED: Homo sapiens similar to Signal recognition particle 9 kDa protein (SRP9) (LOC653226), mRNA. | -0,64301 | 10,41195 | -4,18470 | 0,00207 | 0,02714 |
| VRK1 | Homo sapiens vaccinia related kinase 1 (VRK1), mRNA. | -0,64351 | 8,22723 | -3,74297 | 0,00414 | 0,04093 |
| IFIT1 | Homo sapiens interferon-induced protein with tetratricopeptide repeats 1 (IFIT1), transcript variant 2, mRNA. | -0,64443 | 7,71118 | -5,93520 | 0,00017 | 0,00774 |
| CBX2 | Homo sapiens chromobox homolog 2 (Pc class homolog, Drosophila) (CBX2), transcript variant 1, mRNA. | -0,64612 | 7,92395 | -6,09609 | 0,00014 | 0,00709 |
| WDR57 | Homo sapiens WD repeat domain 57 (U5 snRNP specific) (WDR57), mRNA. | -0,64652 | 9,01090 | -8,70798 | 0,00001 | 0,00218 |
| LOC728188 | PREDICTED: Homo sapiens similar to phosphoglycerate mutase processed protein (LOC728188), mRNA. | -0,64780 | 9,41372 | -7,09715 | 0,00004 | 0,00422 |
| SUV39H1 | Homo sapiens suppressor of variegation 3-9 homolog 1 (Drosophila) (SUV39H1), mRNA. | -0,64781 | 8,32504 | -4,23944 | 0,00190 | 0,02607 |
| LOC729769 | PREDICTED: Homo sapiens similar to Ubiquinol-cytochrome c reductase hinge protein (LOC729769), mRNA. | -0,64821 | 10,81195 | -5,47841 | 0,00032 | 0,01032 |
| ATP5J | Homo sapiens ATP synthase, H+ transporting, mitochondrial F0 complex, subunit F6 (ATP5J), nuclear gene encoding mitochondrial protein, transcript variant 3, mRNA. | -0,64859 | 11,20076 | -4,70973 | 0,00094 | 0,01769 |
| PHLDB1 | Homo sapiens pleckstrin homology-like domain, family B, member 1 (PHLDB1), mRNA. | -0,64962 | 8,01790 | -6,33972 | 0,00010 | 0,00627 |
| BSCL2 | Homo sapiens Bernardinelli-Seip congenital lipodystrophy 2 (seipin) (BSCL2), mRNA. | -0,65144 | 8,09515 | -5,81886 | 0,00020 | 0,00826 |
| SCARNA13 | Homo sapiens small Cajal body-specific RNA 13 (SCARNA13), guide RNA. | -0,65212 | 7,73261 | -3,77468 | 0,00394 | 0,03966 |
| PDXP | Homo sapiens pyridoxal (pyridoxine, vitamin B6) phosphatase (PDXP), mRNA. | -0,65225 | 8,78340 | -3,89486 | 0,00326 | 0,03546 |
| SARS2 | Homo sapiens seryl-tRNA synthetase 2, mitochondrial (SARS2), nuclear gene encoding mitochondrial protein, mRNA. | -0,65267 | 8,76210 | -4,56135 | 0,00117 | 0,01997 |
| EXOSC9 | Homo sapiens exosome component 9 (EXOSC9), transcript variant 1, mRNA. | -0,65299 | 8,94284 | -3,71953 | 0,00430 | 0,04181 |
| XYLT2 | Homo sapiens xylosyltransferase II (XYLT2), mRNA. | -0,65307 | 9,44783 | -4,57379 | 0,00115 | 0,01982 |
| PKMYT1 | Homo sapiens protein kinase, membrane associated tyrosine/threonine 1 (PKMYT1), transcript variant 2, mRNA. | -0,65334 | 7,95133 | -4,16710 | 0,00213 | 0,02752 |
| ARS2 | Homo sapiens arsenate resistance protein 2 (ARS2), transcript variant 1, mRNA. | -0,65363 | 10,60172 | -7,76482 | 0,00002 | 0,00325 |
| H2AFY2 | Homo sapiens H2A histone family, member Y2 (H2AFY2), mRNA. | -0,65443 | 8,03845 | -4,78152 | 0,00085 | 0,01689 |
| RAD51C | Homo sapiens RAD51 homolog C (S. cerevisiae) (RAD51C), transcript variant 1, mRNA. | -0,65523 | 7,99265 | -3,54775 | 0,00568 | 0,04936 |
| POLR2F | Homo sapiens polymerase (RNA) II (DNA directed) polypeptide F (POLR2F), mRNA. | -0,65551 | 10,53075 | -5,03705 | 0,00059 | 0,01398 |
| DHX9 | Homo sapiens DEAH (Asp-Glu-Ala-His) box polypeptide 9 (DHX9), mRNA. | -0,65630 | 8,25394 | -4,52250 | 0,00124 | 0,02056 |
| IMPDH2 | Homo sapiens IMP (inosine monophosphate) dehydrogenase 2 (IMPDH2), mRNA. | -0,65672 | 11,21984 | -3,66648 | 0,00469 | 0,04398 |
| MRPS9 | Homo sapiens mitochondrial ribosomal protein S9 (MRPS9), nuclear gene encoding mitochondrial protein, mRNA. | -0,65700 | 9,37081 | -5,09093 | 0,00054 | 0,01341 |
| LYAR | Homo sapiens Ly1 antibody reactive homolog (mouse) (LYAR), mRNA. | -0,65710 | 9,49031 | -3,57810 | 0,00541 | 0,04808 |
| CISD1 | Homo sapiens CDGSH iron sulfur domain 1 (CISD1), mRNA. | -0,65777 | 10,40260 | -5,23948 | 0,00044 | 0,01201 |
| KLHDC3 | Homo sapiens kelch domain containing 3 (KLHDC3), mRNA. | -0,65844 | 9,54778 | -4,45017 | 0,00138 | 0,02184 |
| BLMH | Homo sapiens bleomycin hydrolase (BLMH), mRNA. | -0,65988 | 8,23076 | -5,91003 | 0,00018 | 0,00776 |
| C14orf106 | Homo sapiens chromosome 14 open reading frame 106 (C14orf106), mRNA. | -0,66265 | 8,16553 | -4,37755 | 0,00154 | 0,02325 |
| GCNT1 | Homo sapiens glucosaminyl (N-acetyl) transferase 1, core 2 (beta-1,6-N-acetylglucosaminyltransferase) (GCNT1), transcript variant 4, mRNA. | -0,66287 | 7,90523 | -5,62421 | 0,00026 | 0,00933 |
| ATP5F1 | Homo sapiens ATP synthase, H+ transporting, mitochondrial F0 complex, subunit B1 (ATP5F1), nuclear gene encoding mitochondrial protein, mRNA. | -0,66711 | 9,79598 | -3,68325 | 0,00456 | 0,04332 |
| CHEK1 | Homo sapiens CHK1 checkpoint homolog (S. pombe) (CHEK1), mRNA. | -0,66818 | 8,33820 | -4,04679 | 0,00256 | 0,03090 |
| RUVBL1 | Homo sapiens RuvB-like 1 (E. coli) (RUVBL1), mRNA. | -0,66895 | 8,66651 | -3,99317 | 0,00279 | 0,03242 |
| BLOC1S1 | Homo sapiens biogenesis of lysosome-related organelles complex-1, subunit 1 (BLOC1S1), mRNA. | -0,66958 | 8,33632 | -4,14718 | 0,00219 | 0,02804 |
| C16orf59 | Homo sapiens chromosome 16 open reading frame 59 (C16orf59), mRNA. | -0,66988 | 7,74145 | -7,63665 | 0,00002 | 0,00334 |
| NGFRAP1 | Homo sapiens nerve growth factor receptor (TNFRSF16) associated protein 1 (NGFRAP1), transcript variant 1, mRNA. | -0,67026 | 10,58827 | -3,65781 | 0,00475 | 0,04445 |
| NUDC | Homo sapiens nuclear distribution gene C homolog (A. nidulans) (NUDC), mRNA. | -0,67069 | 11,48325 | -4,67794 | 0,00098 | 0,01809 |
| COPS6 | Homo sapiens COP9 constitutive photomorphogenic homolog subunit 6 (Arabidopsis) (COPS6), mRNA. | -0,67219 | 9,21243 | -4,06624 | 0,00249 | 0,03032 |
| HNRNPM | Homo sapiens heterogeneous nuclear ribonucleoprotein M (HNRNPM), transcript variant 1, mRNA. | -0,67377 | 11,34988 | -4,37762 | 0,00154 | 0,02325 |
| HSPA14 | Homo sapiens heat shock 70kDa protein 14 (HSPA14), transcript variant 1, mRNA. | -0,67429 | 8,18667 | -5,42929 | 0,00034 | 0,01065 |
| TOMM5 | Homo sapiens translocase of outer mitochondrial membrane 5 homolog (yeast) (TOMM5), nuclear gene encoding mitochondrial protein, transcript variant 1, mRNA. | -0,67519 | 11,04395 | -4,45021 | 0,00138 | 0,02184 |
| C16orf53 | Homo sapiens chromosome 16 open reading frame 53 (C16orf53), mRNA. | -0,67738 | 9,08722 | -4,51416 | 0,00125 | 0,02067 |
| MND1 | Homo sapiens meiotic nuclear divisions 1 homolog (S. cerevisiae) (MND1), mRNA. | -0,67740 | 7,80523 | -3,61521 | 0,00509 | 0,04624 |
| SEPX1 | Homo sapiens selenoprotein X, 1 (SEPX1), mRNA. | -0,67776 | 9,23869 | -4,77737 | 0,00085 | 0,01692 |
| NT5DC2 | Homo sapiens 5'-nucleotidase domain containing 2 (NT5DC2), mRNA. | -0,67784 | 8,77449 | -6,10041 | 0,00014 | 0,00709 |
| MID1 | Homo sapiens midline 1 (Opitz/BBB syndrome) (MID1), transcript variant 3, mRNA. | -0,67857 | 8,29362 | -5,68414 | 0,00024 | 0,00906 |
| BASP1 | Homo sapiens brain abundant, membrane attached signal protein 1 (BASP1), mRNA. | -0,67899 | 12,81751 | -5,10174 | 0,00053 | 0,01329 |
| IRS1 | Homo sapiens insulin receptor substrate 1 (IRS1), mRNA. | -0,68052 | 8,50284 | -4,70471 | 0,00095 | 0,01771 |
| LOC389049 | PREDICTED: Homo sapiens misc_RNA (LOC389049), miscRNA. | -0,68123 | 8,29748 | -3,86901 | 0,00339 | 0,03629 |
| FOXM1 | Homo sapiens forkhead box M1 (FOXM1), transcript variant 3, mRNA. | -0,68125 | 7,92569 | -3,78838 | 0,00385 | 0,03910 |
| CDK2 | Homo sapiens cyclin-dependent kinase 2 (CDK2), transcript variant 1, mRNA. | -0,68233 | 9,11065 | -5,20990 | 0,00046 | 0,01238 |
| DLEU1 | Homo sapiens deleted in lymphocytic leukemia 1 (non-protein coding) (DLEU1), non-coding RNA. | -0,68247 | 8,02972 | -5,05908 | 0,00057 | 0,01372 |
| PAQR4 | Homo sapiens progestin and adipoQ receptor family member IV (PAQR4), mRNA. | -0,68360 | 9,10317 | -4,50658 | 0,00127 | 0,02076 |
| EXOSC9 | Homo sapiens exosome component 9 (EXOSC9), transcript variant 1, mRNA. | -0,68372 | 8,91526 | -4,34870 | 0,00161 | 0,02385 |
| CDC7 | Homo sapiens cell division cycle 7 homolog (S. cerevisiae) (CDC7), mRNA. | -0,68506 | 7,78428 | -8,96398 | 0,00001 | 0,00196 |
| SAC3D1 | Homo sapiens SAC3 domain containing 1 (SAC3D1), mRNA. | -0,68656 | 9,26428 | -5,68460 | 0,00024 | 0,00906 |
| TSEN34 | Homo sapiens tRNA splicing endonuclease 34 homolog (S. cerevisiae) (TSEN34), transcript variant 2, mRNA. | -0,68815 | 10,58403 | -7,02310 | 0,00005 | 0,00442 |
| CAMK2N2 | Homo sapiens calcium/calmodulin-dependent protein kinase II inhibitor 2 (CAMK2N2), mRNA. | -0,68879 | 8,38045 | -4,43975 | 0,00140 | 0,02210 |
| LPHN1 | Homo sapiens latrophilin 1 (LPHN1), transcript variant 2, mRNA. | -0,68884 | 8,04672 | -5,75515 | 0,00022 | 0,00865 |
| HNRNPAB | Homo sapiens heterogeneous nuclear ribonucleoprotein A/B (HNRNPAB), transcript variant 1, mRNA. | -0,69013 | 9,83216 | -4,20993 | 0,00199 | 0,02654 |
| C14orf156 | Homo sapiens chromosome 14 open reading frame 156 (C14orf156), mRNA. | -0,69097 | 12,23796 | -3,91157 | 0,00317 | 0,03485 |
| PDSS1 | Homo sapiens prenyl (decaprenyl) diphosphate synthase, subunit 1 (PDSS1), mRNA. | -0,69168 | 8,21890 | -4,53030 | 0,00122 | 0,02039 |
| LSM3 | Homo sapiens LSM3 homolog, U6 small nuclear RNA associated (S. cerevisiae) (LSM3), mRNA. | -0,69224 | 10,45687 | -6,25985 | 0,00011 | 0,00663 |
| NMU | Homo sapiens neuromedin U (NMU), mRNA. | -0,69307 | 7,73834 | -4,04936 | 0,00255 | 0,03083 |
| THYN1 | Homo sapiens thymocyte nuclear protein 1 (THYN1), transcript variant 3, mRNA. | -0,69367 | 8,19317 | -3,66280 | 0,00471 | 0,04418 |
| BMP4 | Homo sapiens bone morphogenetic protein 4 (BMP4), transcript variant 3, mRNA. | -0,69426 | 7,61788 | -7,18625 | 0,00004 | 0,00415 |
| APEX1 | Homo sapiens APEX nuclease (multifunctional DNA repair enzyme) 1 (APEX1), transcript variant 3, mRNA. | -0,69427 | 10,73115 | -5,33629 | 0,00039 | 0,01132 |
| KAT2A | Homo sapiens K(lysine) acetyltransferase 2A (KAT2A), mRNA. | -0,69467 | 8,53285 | -4,12255 | 0,00228 | 0,02875 |
| RBM23 | Homo sapiens RNA binding motif protein 23 (RBM23), transcript variant 2, mRNA. | -0,69587 | 9,55468 | -4,51744 | 0,00125 | 0,02061 |
| RANGAP1 | Homo sapiens Ran GTPase activating protein 1 (RANGAP1), mRNA. | -0,69676 | 10,21080 | -3,70232 | 0,00442 | 0,04252 |
| RBM23 | Homo sapiens RNA binding motif protein 23 (RBM23), transcript variant 3, mRNA. | -0,69703 | 9,49998 | -9,18957 | 0,00000 | 0,00188 |
| NDUFB6 | Homo sapiens NADH dehydrogenase (ubiquinone) 1 beta subcomplex, 6, 17kDa (NDUFB6), nuclear gene encoding mitochondrial protein, transcript variant 1, mRNA. | -0,69718 | 10,04125 | -3,96939 | 0,00289 | 0,03311 |
| FLJ12684 | Homo sapiens hypothetical protein FLJ12684 (FLJ12684), mRNA. XR_001254 | -0,69794 | 8,10219 | -4,70339 | 0,00095 | 0,01773 |
| CTDSPL | Homo sapiens CTD (carboxy-terminal domain, RNA polymerase II, polypeptide A) small phosphatase-like (CTDSPL), transcript variant 2, mRNA. | -0,69861 | 8,37191 | -3,65059 | 0,00481 | 0,04469 |
| C3orf37 | Homo sapiens chromosome 3 open reading frame 37 (C3orf37), transcript variant 2, mRNA. | -0,69966 | 8,71805 | -6,72826 | 0,00006 | 0,00490 |
| AKR1B1 | Homo sapiens aldo-keto reductase family 1, member B1 (aldose reductase) (AKR1B1), mRNA. | -0,70007 | 9,93531 | -4,54420 | 0,00120 | 0,02021 |
| NDUFS3 | Homo sapiens NADH dehydrogenase (ubiquinone) Fe-S protein 3, 30kDa (NADH-coenzyme Q reductase) (NDUFS3), mRNA. | -0,70031 | 10,64426 | -4,49338 | 0,00129 | 0,02105 |
| C5orf13 | Homo sapiens chromosome 5 open reading frame 13 (C5orf13), mRNA. | -0,70167 | 7,81816 | -3,98295 | 0,00283 | 0,03278 |
| CDCA4 | Homo sapiens cell division cycle associated 4 (CDCA4), transcript variant 14, mRNA. | -0,70578 | 8,97454 | -6,33367 | 0,00010 | 0,00630 |
| FANCL | Homo sapiens Fanconi anemia, complementation group L (FANCL), mRNA. | -0,70689 | 7,91916 | -5,33233 | 0,00039 | 0,01132 |
| SNRNP40 | Homo sapiens small nuclear ribonucleoprotein 40kDa (U5) (SNRNP40), mRNA. | -0,70832 | 9,70243 | -5,08455 | 0,00055 | 0,01344 |
| SPRY1 | Homo sapiens sprouty homolog 1, antagonist of FGF signaling (Drosophila) (SPRY1), transcript variant 2, mRNA. | -0,70872 | 7,70235 | -6,12113 | 0,00014 | 0,00709 |
| SERTAD4 | Homo sapiens SERTA domain containing 4 (SERTAD4), mRNA. | -0,70910 | 8,00076 | -5,00100 | 0,00062 | 0,01436 |
| ARHGEF19 | Homo sapiens Rho guanine nucleotide exchange factor (GEF) 19 (ARHGEF19), mRNA. | -0,70922 | 7,74481 | -8,12447 | 0,00001 | 0,00281 |
| RBBP8 | Homo sapiens retinoblastoma binding protein 8 (RBBP8), transcript variant 2, mRNA. | -0,71071 | 8,76037 | -3,96074 | 0,00293 | 0,03339 |
| CASP2 | Homo sapiens caspase 2, apoptosis-related cysteine peptidase (CASP2), transcript variant 1, mRNA. | -0,71169 | 9,39808 | -4,36274 | 0,00158 | 0,02358 |
| C7orf59 | Homo sapiens chromosome 7 open reading frame 59 (C7orf59), mRNA. | -0,71218 | 10,38013 | -6,32662 | 0,00011 | 0,00633 |
| WDR34 | Homo sapiens WD repeat domain 34 (WDR34), mRNA. | -0,71385 | 8,37051 | -4,64063 | 0,00104 | 0,01875 |
| CCDC115 | Homo sapiens coiled-coil domain containing 115 (CCDC115), mRNA. | -0,71431 | 8,36060 | -4,28139 | 0,00178 | 0,02512 |
| LOC641844 | PREDICTED: Homo sapiens misc_RNA (LOC641844), miscRNA. | -0,71534 | 9,62712 | -5,23278 | 0,00044 | 0,01208 |
| ENSA | Homo sapiens endosulfine alpha (ENSA), transcript variant 2, mRNA. | -0,71567 | 8,55148 | -3,58041 | 0,00538 | 0,04798 |
| POLD1 | Homo sapiens polymerase (DNA directed), delta 1, catalytic subunit 125kDa (POLD1), mRNA. | -0,71643 | 8,04652 | -4,02749 | 0,00264 | 0,03148 |
| APEX1 | Homo sapiens APEX nuclease (multifunctional DNA repair enzyme) 1 (APEX1), transcript variant 1, mRNA. | -0,71676 | 9,90740 | -4,72002 | 0,00092 | 0,01757 |
| THOC3 | Homo sapiens THO complex 3 (THOC3), mRNA. | -0,71756 | 9,11887 | -6,66931 | 0,00007 | 0,00506 |
| THOC4 | Homo sapiens THO complex 4 (THOC4), mRNA. | -0,71785 | 8,53672 | -5,18584 | 0,00047 | 0,01259 |
| CPSF4 | Homo sapiens cleavage and polyadenylation specific factor 4, 30kDa (CPSF4), transcript variant 2, mRNA. | -0,71985 | 10,31245 | -4,57312 | 0,00115 | 0,01982 |
| LOC100133372 | PREDICTED: Homo sapiens misc_RNA (LOC100133372), miscRNA. | -0,72120 | 11,19981 | -6,49319 | 0,00009 | 0,00582 |
| LRRCC1 | Homo sapiens leucine rich repeat and coiled-coil domain containing 1 (LRRCC1), transcript variant 1, mRNA. | -0,72160 | 8,09389 | -3,88037 | 0,00333 | 0,03587 |
| LMAN2L | Homo sapiens lectin, mannose-binding 2-like (LMAN2L), mRNA. | -0,72235 | 8,71455 | -5,06999 | 0,00056 | 0,01358 |
| HNRPM | Homo sapiens heterogeneous nuclear ribonucleoprotein M (HNRPM), transcript variant 1, mRNA. | -0,72311 | 11,73460 | -5,42932 | 0,00034 | 0,01065 |
| GEMIN6 | Homo sapiens gem (nuclear organelle) associated protein 6 (GEMIN6), mRNA. | -0,72349 | 8,77503 | -3,79607 | 0,00381 | 0,03881 |
| C7orf27 | Homo sapiens chromosome 7 open reading frame 27 (C7orf27), mRNA. | -0,72396 | 8,96102 | -4,21263 | 0,00198 | 0,02652 |
| PDCD2 | Homo sapiens programmed cell death 2 (PDCD2), transcript variant 2, mRNA. | -0,72517 | 9,01251 | -5,99135 | 0,00016 | 0,00747 |
| PGAM1 | Homo sapiens phosphoglycerate mutase 1 (brain) (PGAM1), mRNA. | -0,72520 | 10,29130 | -6,19768 | 0,00012 | 0,00686 |
| LOC732007 | PREDICTED: Homo sapiens similar to Phosphoglycerate mutase 1 (Phosphoglycerate mutase isozyme B) (PGAM-B) (BPG-dependent PGAM 1) (LOC732007), mRNA. | -0,72582 | 10,55396 | -7,55099 | 0,00003 | 0,00354 |
| POP5 | Homo sapiens processing of precursor 5, ribonuclease P/MRP subunit (S. cerevisiae) (POP5), transcript variant 3, mRNA. | -0,72635 | 8,62240 | -3,97293 | 0,00288 | 0,03308 |
| ISOC2 | Homo sapiens isochorismatase domain containing 2 (ISOC2), mRNA. | -0,72691 | 9,10267 | -4,54947 | 0,00119 | 0,02018 |
| H2AFZ | Homo sapiens H2A histone family, member Z (H2AFZ), mRNA. | -0,72743 | 12,67414 | -3,70370 | 0,00441 | 0,04248 |
| HMBS | Homo sapiens hydroxymethylbilane synthase (HMBS), transcript variant 1, mRNA. | -0,72747 | 8,82502 | -5,34306 | 0,00038 | 0,01131 |
| RMI1 | Homo sapiens RMI1, RecQ mediated genome instability 1, homolog (S. cerevisiae) (RMI1), mRNA. | -0,72759 | 8,09029 | -6,56455 | 0,00008 | 0,00552 |
| PSMB8 | Homo sapiens proteasome (prosome, macropain) subunit, beta type, 8 (large multifunctional peptidase 7) (PSMB8), transcript variant 1, mRNA. | -0,72949 | 8,53625 | -3,95232 | 0,00297 | 0,03355 |
| SFRS10 | Homo sapiens splicing factor, arginine/serine-rich 10 (transformer 2 homolog, Drosophila) (SFRS10), mRNA. | -0,73032 | 9,81596 | -3,97439 | 0,00287 | 0,03307 |
| HNRNPAB | Homo sapiens heterogeneous nuclear ribonucleoprotein A/B (HNRNPAB), transcript variant 2, mRNA. | -0,73257 | 10,89856 | -4,96517 | 0,00065 | 0,01476 |
| TOMM22 | Homo sapiens translocase of outer mitochondrial membrane 22 homolog (yeast) (TOMM22), nuclear gene encoding mitochondrial protein, mRNA. | -0,73670 | 9,32872 | -6,20691 | 0,00012 | 0,00684 |
| MRPL34 | Homo sapiens mitochondrial ribosomal protein L34 (MRPL34), nuclear gene encoding mitochondrial protein, mRNA. | -0,73703 | 9,31606 | -3,99227 | 0,00279 | 0,03244 |
| USP39 | Homo sapiens ubiquitin specific peptidase 39 (USP39), mRNA. | -0,73755 | 8,93280 | -5,45802 | 0,00033 | 0,01048 |
| ITGAE | Homo sapiens integrin, alpha E (antigen CD103, human mucosal lymphocyte antigen 1; alpha polypeptide) (ITGAE), mRNA. | -0,73817 | 8,88387 | -6,01562 | 0,00016 | 0,00738 |
| C12orf32 | Homo sapiens chromosome 12 open reading frame 32 (C12orf32), mRNA. | -0,73938 | 8,27841 | -4,76167 | 0,00087 | 0,01715 |
| SMC2 | Homo sapiens structural maintenance of chromosomes 2 (SMC2), transcript variant 1, mRNA. | -0,73942 | 8,26335 | -3,76558 | 0,00400 | 0,03999 |
| LSM10 | Homo sapiens LSM10, U7 small nuclear RNA associated (LSM10), mRNA. | -0,74162 | 8,31855 | -6,41320 | 0,00009 | 0,00609 |
| RMI1 | Homo sapiens RMI1, RecQ mediated genome instability 1, homolog (S. cerevisiae) (RMI1), mRNA. | -0,74237 | 8,29132 | -7,32782 | 0,00003 | 0,00393 |
| CISD1 | Homo sapiens CDGSH iron sulfur domain 1 (CISD1), mRNA. | -0,74382 | 10,67623 | -4,62722 | 0,00106 | 0,01895 |
| PRICKLE1 | Homo sapiens prickle homolog 1 (Drosophila) (PRICKLE1), mRNA. | -0,74385 | 7,99312 | -9,90300 | 0,00000 | 0,00142 |
| PSME3 | Homo sapiens proteasome (prosome, macropain) activator subunit 3 (PA28 gamma; Ki) (PSME3), transcript variant 1, mRNA. | -0,74474 | 8,82276 | -4,12046 | 0,00229 | 0,02879 |
| NCL | Homo sapiens nucleolin (NCL), mRNA. | -0,74573 | 9,16278 | -6,16347 | 0,00013 | 0,00695 |
| LOC387703 | PREDICTED: Homo sapiens misc_RNA (LOC387703), miscRNA. | -0,74708 | 8,88484 | -4,47022 | 0,00134 | 0,02154 |
| RAD51C | Homo sapiens RAD51 homolog C (S. cerevisiae) (RAD51C), transcript variant 1, mRNA. | -0,74797 | 8,05200 | -4,88985 | 0,00072 | 0,01571 |
| FANCG | Homo sapiens Fanconi anemia, complementation group G (FANCG), mRNA. | -0,74916 | 8,38409 | -3,57513 | 0,00543 | 0,04821 |
| ARID5B | Homo sapiens AT rich interactive domain 5B (MRF1-like) (ARID5B), mRNA. | -0,74928 | 8,02639 | -6,82014 | 0,00006 | 0,00479 |
| ACN9 | Homo sapiens ACN9 homolog (S. cerevisiae) (ACN9), mRNA. | -0,75019 | 8,97805 | -3,60981 | 0,00513 | 0,04648 |
| C9orf3 | Homo sapiens chromosome 9 open reading frame 3 (C9orf3), mRNA. | -0,75097 | 7,91422 | -3,82504 | 0,00364 | 0,03768 |
| THOC3 | Homo sapiens THO complex 3 (THOC3), mRNA. | -0,75200 | 9,65497 | -5,88437 | 0,00018 | 0,00789 |
| RFC3 | Homo sapiens replication factor C (activator 1) 3, 38kDa (RFC3), transcript variant 1, mRNA. | -0,75233 | 7,85067 | -6,11774 | 0,00014 | 0,00709 |
| MNS1 | Homo sapiens meiosis-specific nuclear structural 1 (MNS1), mRNA. | -0,75281 | 7,85534 | -3,88622 | 0,00330 | 0,03566 |
| STIP1 | Homo sapiens stress-induced-phosphoprotein 1 (Hsp70/Hsp90-organizing protein) (STIP1), mRNA. | -0,75313 | 10,13526 | -4,80766 | 0,00081 | 0,01659 |
| OIP5 | Homo sapiens Opa interacting protein 5 (OIP5), mRNA. | -0,75314 | 7,92292 | -3,95328 | 0,00297 | 0,03355 |
| PSMB10 | Homo sapiens proteasome (prosome, macropain) subunit, beta type, 10 (PSMB10), mRNA. | -0,75316 | 9,87772 | -5,25583 | 0,00043 | 0,01192 |
| PGAM4 | Homo sapiens phosphoglycerate mutase family member 4 (PGAM4), mRNA. | -0,75357 | 10,01458 | -7,69011 | 0,00002 | 0,00332 |
| B3GNT1 | Homo sapiens UDP-GlcNAc:betaGal beta-1,3-N-acetylglucosaminyltransferase 1 (B3GNT1), mRNA. | -0,75360 | 7,89411 | -6,36813 | 0,00010 | 0,00617 |
| ARV1 | Homo sapiens ARV1 homolog (S. cerevisiae) (ARV1), mRNA. | -0,75581 | 8,34157 | -3,98241 | 0,00284 | 0,03279 |
| JAG2 | Homo sapiens jagged 2 (JAG2), transcript variant 1, mRNA. | -0,75661 | 8,31179 | -4,75232 | 0,00088 | 0,01721 |
| MRPL20 | Homo sapiens mitochondrial ribosomal protein L20 (MRPL20), nuclear gene encoding mitochondrial protein, mRNA. | -0,75722 | 9,16785 | -5,67920 | 0,00024 | 0,00908 |
| GTF2IP1 | Homo sapiens general transcription factor II, i, pseudogene 1 (GTF2IP1) on chromosome 7. | -0,75749 | 8,90474 | -4,82981 | 0,00079 | 0,01625 |
| PGAM4 | Homo sapiens phosphoglycerate mutase family member 4 (PGAM4), mRNA. | -0,75754 | 9,45606 | -9,18890 | 0,00000 | 0,00188 |
| LPCAT3 | Homo sapiens lysophosphatidylcholine acyltransferase 3 (LPCAT3), mRNA. | -0,76063 | 8,52187 | -5,85329 | 0,00019 | 0,00807 |
| CCDC77 | Homo sapiens coiled-coil domain containing 77 (CCDC77), mRNA. | -0,76464 | 8,09838 | -5,24579 | 0,00044 | 0,01194 |
| TMEM14A | Homo sapiens transmembrane protein 14A (TMEM14A), mRNA. | -0,76543 | 10,50548 | -5,19400 | 0,00047 | 0,01247 |
| NDUFB7 | Homo sapiens NADH dehydrogenase (ubiquinone) 1 beta subcomplex, 7, 18kDa (NDUFB7), nuclear gene encoding mitochondrial protein, mRNA. | -0,76631 | 10,38513 | -4,41433 | 0,00146 | 0,02249 |
| NHP2L1 | Homo sapiens NHP2 non-histone chromosome protein 2-like 1 (S. cerevisiae) (NHP2L1), transcript variant 2, mRNA. | -0,76686 | 9,10002 | -3,93971 | 0,00303 | 0,03388 |
| HNRPA2B1 | Homo sapiens heterogeneous nuclear ribonucleoprotein A2/B1 (HNRPA2B1), transcript variant B1, mRNA. | -0,76898 | 12,30709 | -4,01790 | 0,00268 | 0,03172 |
| LOC653110 | PREDICTED: Homo sapiens similar to annexin A8, transcript variant 1 (LOC653110), mRNA. | -0,76906 | 9,26834 | -4,54019 | 0,00121 | 0,02027 |
| CNTNAP2 | Homo sapiens contactin associated protein-like 2 (CNTNAP2), mRNA. | -0,76921 | 7,56740 | -5,97211 | 0,00016 | 0,00758 |
| PGAM1 | Homo sapiens phosphoglycerate mutase 1 (brain) (PGAM1), mRNA. | -0,76992 | 11,16400 | -10,08942 | 0,00000 | 0,00126 |
| D2HGDH | Homo sapiens D-2-hydroxyglutarate dehydrogenase (D2HGDH), nuclear gene encoding mitochondrial protein, mRNA. | -0,77028 | 8,01373 | -4,75561 | 0,00088 | 0,01717 |
| ICMT | Homo sapiens isoprenylcysteine carboxyl methyltransferase (ICMT), mRNA. | -0,77199 | 9,07829 | -3,93270 | 0,00307 | 0,03411 |
| XRCC6BP1 | Homo sapiens XRCC6 binding protein 1 (XRCC6BP1), mRNA. | -0,77361 | 8,03618 | -5,82870 | 0,00020 | 0,00823 |
| CDCA7L | Homo sapiens cell division cycle associated 7-like (CDCA7L), mRNA. | -0,77602 | 8,53821 | -5,77048 | 0,00021 | 0,00854 |
| ILF3 | Homo sapiens interleukin enhancer binding factor 3, 90kDa (ILF3), transcript variant 2, mRNA. | -0,77639 | 8,80373 | -6,25824 | 0,00011 | 0,00663 |
| SNCA | Homo sapiens synuclein, alpha (non A4 component of amyloid precursor) (SNCA), transcript variant NACP140, mRNA. | -0,77658 | 7,91741 | -4,20246 | 0,00201 | 0,02670 |
| TGIF2 | Homo sapiens TGFB-induced factor homeobox 2 (TGIF2), mRNA. | -0,77802 | 7,89100 | -10,92422 | 0,00000 | 0,00088 |
| LOC647081 | PREDICTED: Homo sapiens misc_RNA (LOC647081), miscRNA. | -0,77823 | 9,03725 | -5,13611 | 0,00051 | 0,01295 |
| JMJD8 | Homo sapiens jumonji domain containing 8 (JMJD8), mRNA. | -0,77842 | 9,63326 | -5,17481 | 0,00048 | 0,01265 |
| SEPHS1 | Homo sapiens selenophosphate synthetase 1 (SEPHS1), mRNA. | -0,77973 | 8,73494 | -4,11126 | 0,00232 | 0,02904 |
| GCSH | Homo sapiens glycine cleavage system protein H (aminomethyl carrier) (GCSH), mRNA. | -0,78146 | 9,50360 | -3,89258 | 0,00327 | 0,03548 |
| C21orf45 | Homo sapiens chromosome 21 open reading frame 45 (C21orf45), mRNA. | -0,78159 | 8,29539 | -5,17785 | 0,00048 | 0,01265 |
| USP5 | Homo sapiens ubiquitin specific peptidase 5 (isopeptidase T) (USP5), transcript variant 2, mRNA. | -0,78231 | 10,16799 | -3,59197 | 0,00529 | 0,04734 |
| KLHL13 | Homo sapiens kelch-like 13 (Drosophila) (KLHL13), mRNA. | -0,78304 | 7,64858 | -7,18256 | 0,00004 | 0,00415 |
| XRCC3 | Homo sapiens X-ray repair complementing defective repair in Chinese hamster cells 3 (XRCC3), transcript variant 3, mRNA. | -0,78735 | 7,99660 | -4,85839 | 0,00076 | 0,01595 |
| KIF11 | Homo sapiens kinesin family member 11 (KIF11), mRNA. | -0,78793 | 7,90531 | -5,20323 | 0,00046 | 0,01244 |
| CCNE2 | Homo sapiens cyclin E2 (CCNE2), transcript variant 2, mRNA. | -0,78987 | 7,84053 | -6,57175 | 0,00008 | 0,00552 |
| GLRX5 | Homo sapiens glutaredoxin 5 (GLRX5), mRNA. | -0,79076 | 11,19833 | -4,13046 | 0,00225 | 0,02853 |
| FAM111A | Homo sapiens family with sequence similarity 111, member A (FAM111A), transcript variant 1, mRNA. | -0,79118 | 8,03610 | -5,29125 | 0,00041 | 0,01162 |
| GGCT | Homo sapiens gamma-glutamyl cyclotransferase (GGCT), mRNA. | -0,79136 | 9,00961 | -5,53206 | 0,00029 | 0,00999 |
| RFX5 | Homo sapiens regulatory factor X, 5 (influences HLA class II expression) (RFX5), transcript variant 2, mRNA. | -0,79197 | 8,50103 | -4,70534 | 0,00095 | 0,01771 |
| C20orf27 | Homo sapiens chromosome 20 open reading frame 27 (C20orf27), mRNA. | -0,79239 | 8,59066 | -6,17665 | 0,00013 | 0,00689 |
| XRCC6 | Homo sapiens X-ray repair complementing defective repair in Chinese hamster cells 6 (XRCC6), mRNA. | -0,79417 | 10,44842 | -4,06574 | 0,00249 | 0,03032 |
| POLR3K | Homo sapiens polymerase (RNA) III (DNA directed) polypeptide K, 12.3 kDa (POLR3K), mRNA. | -0,79422 | 8,03685 | -5,28155 | 0,00042 | 0,01169 |
| UBR7 | Homo sapiens ubiquitin protein ligase E3 component n-recognin 7 (putative) (UBR7), transcript variant 3, mRNA. | -0,79424 | 8,23263 | -4,88260 | 0,00073 | 0,01577 |
| CACYBP | Homo sapiens calcyclin binding protein (CACYBP), transcript variant 1, mRNA. | -0,79545 | 9,54333 | -6,70977 | 0,00007 | 0,00494 |
| JMJD8 | Homo sapiens jumonji domain containing 8 (JMJD8), mRNA. | -0,79569 | 10,32467 | -6,12092 | 0,00014 | 0,00709 |
| RPA2 | Homo sapiens replication protein A2, 32kDa (RPA2), mRNA. | -0,79648 | 9,57427 | -5,12585 | 0,00052 | 0,01303 |
| HSPE1 | Homo sapiens heat shock 10kDa protein 1 (chaperonin 10) (HSPE1), mRNA. | -0,79682 | 11,37236 | -3,91362 | 0,00316 | 0,03477 |
| LMNB1 | Homo sapiens lamin B1 (LMNB1), mRNA. | -0,79742 | 7,74173 | -6,81440 | 0,00006 | 0,00479 |
| PRMT6 | Homo sapiens protein arginine methyltransferase 6 (PRMT6), mRNA. | -0,79777 | 8,12768 | -5,32983 | 0,00039 | 0,01132 |
| CCDC14 | Homo sapiens coiled-coil domain containing 14 (CCDC14), mRNA. | -0,79793 | 8,69588 | -5,69554 | 0,00024 | 0,00902 |
| RPL8 | Homo sapiens ribosomal protein L8 (RPL8), transcript variant 2, mRNA. | -0,79898 | 9,47260 | -3,57284 | 0,00545 | 0,04830 |
| KIF20B | Homo sapiens kinesin family member 20B (KIF20B), mRNA. | -0,79993 | 8,37865 | -3,84131 | 0,00354 | 0,03726 |
| LOC648695 | PREDICTED: Homo sapiens similar to retinoblastoma binding protein 4, transcript variant 5 (LOC648695), mRNA. | -0,80030 | 9,47507 | -4,01880 | 0,00268 | 0,03171 |
| MARCKS | Homo sapiens myristoylated alanine-rich protein kinase C substrate (MARCKS), mRNA. | -0,80184 | 11,51027 | -4,80256 | 0,00082 | 0,01662 |
| NDUFA2 | Homo sapiens NADH dehydrogenase (ubiquinone) 1 alpha subcomplex, 2, 8kDa (NDUFA2), mRNA. | -0,80199 | 10,44908 | -4,47453 | 0,00133 | 0,02148 |
| NUP37 | Homo sapiens nucleoporin 37kDa (NUP37), mRNA. | -0,80289 | 10,47752 | -4,07802 | 0,00244 | 0,02997 |
| NFIB | Homo sapiens nuclear factor I/B (NFIB), mRNA. | -0,80378 | 9,05857 | -3,60094 | 0,00521 | 0,04691 |
| C1QBP | Homo sapiens complement component 1, q subcomponent binding protein (C1QBP), nuclear gene encoding mitochondrial protein, mRNA. | -0,80384 | 10,83225 | -3,67400 | 0,00463 | 0,04371 |
| GRWD1 | Homo sapiens glutamate-rich WD repeat containing 1 (GRWD1), mRNA. | -0,80405 | 9,25316 | -5,92205 | 0,00018 | 0,00775 |
| APTX | Homo sapiens aprataxin (APTX), transcript variant 2, mRNA. | -0,80528 | 8,87597 | -4,03050 | 0,00263 | 0,03142 |
| XRCC5 | Homo sapiens X-ray repair complementing defective repair in Chinese hamster cells 5 (double-strand-break rejoining; Ku autoantigen, 80kDa) (XRCC5), mRNA. | -0,80545 | 9,00032 | -5,35551 | 0,00037 | 0,01127 |
| TUBG1 | Homo sapiens tubulin, gamma 1 (TUBG1), mRNA. | -0,80549 | 9,53727 | -4,21811 | 0,00197 | 0,02639 |
| LQK1 | PREDICTED: Homo sapiens misc_RNA (LQK1), miscRNA. | -0,80710 | 8,21341 | -5,19467 | 0,00047 | 0,01247 |
| TMEM109 | Homo sapiens transmembrane protein 109 (TMEM109), mRNA. | -0,80792 | 8,36546 | -4,89323 | 0,00072 | 0,01571 |
| NSL1 | Homo sapiens NSL1, MIND kinetochore complex component, homolog (S. cerevisiae) (NSL1), transcript variant 2, mRNA. | -0,80829 | 8,82789 | -5,05245 | 0,00057 | 0,01378 |
| GEMIN4 | Homo sapiens gem (nuclear organelle) associated protein 4 (GEMIN4), mRNA. | -0,80936 | 9,22432 | -5,41234 | 0,00035 | 0,01080 |
| DTL | Homo sapiens denticleless homolog (Drosophila) (DTL), mRNA. | -0,81033 | 7,96118 | -6,52504 | 0,00008 | 0,00567 |
| PRPF31 | Homo sapiens PRP31 pre-mRNA processing factor 31 homolog (S. cerevisiae) (PRPF31), mRNA. | -0,81311 | 9,30743 | -7,00879 | 0,00005 | 0,00442 |
| STARD7 | Homo sapiens START domain containing 7 (STARD7), transcript variant 1, mRNA. | -0,81321 | 11,14452 | -4,56661 | 0,00116 | 0,01991 |
| BMP7 | Homo sapiens bone morphogenetic protein 7 (osteogenic protein 1) (BMP7), mRNA. | -0,81365 | 7,92287 | -5,99988 | 0,00016 | 0,00743 |
| ARTN | Homo sapiens artemin (ARTN), transcript variant 2, mRNA. | -0,81385 | 8,91674 | -6,88597 | 0,00005 | 0,00467 |
| STARD7 | Homo sapiens START domain containing 7 (STARD7), transcript variant 2, mRNA. | -0,81499 | 9,57096 | -5,64438 | 0,00025 | 0,00931 |
| ORC6L | Homo sapiens origin recognition complex, subunit 6 like (yeast) (ORC6L), mRNA. | -0,81798 | 8,68021 | -4,04954 | 0,00255 | 0,03083 |
| RFWD3 | Homo sapiens ring finger and WD repeat domain 3 (RFWD3), mRNA. | -0,81898 | 8,79470 | -6,01411 | 0,00016 | 0,00738 |
| SLBP | Homo sapiens stem-loop binding protein (SLBP), mRNA. | -0,82032 | 9,51069 | -7,74327 | 0,00002 | 0,00325 |
| PSME3 | Homo sapiens proteasome (prosome, macropain) activator subunit 3 (PA28 gamma; Ki) (PSME3), transcript variant 1, mRNA. | -0,82087 | 9,05361 | -5,34042 | 0,00038 | 0,01132 |
| MRPL12 | Homo sapiens mitochondrial ribosomal protein L12 (MRPL12), nuclear gene encoding mitochondrial protein, mRNA. | -0,82257 | 9,92978 | -4,11025 | 0,00232 | 0,02904 |
| GPR177 | Homo sapiens G protein-coupled receptor 177 (GPR177), transcript variant 1, mRNA. | -0,82288 | 9,61464 | -6,31032 | 0,00011 | 0,00641 |
| EFEMP1 | Homo sapiens EGF-containing fibulin-like extracellular matrix protein 1 (EFEMP1), transcript variant 1, mRNA. | -0,82300 | 7,81685 | -3,75259 | 0,00408 | 0,04051 |
| HNRNPA1 | Homo sapiens heterogeneous nuclear ribonucleoprotein A1 (HNRNPA1), transcript variant 2, mRNA. | -0,82338 | 8,51564 | -5,36198 | 0,00037 | 0,01119 |
| XRCC6 | Homo sapiens X-ray repair complementing defective repair in Chinese hamster cells 6 (XRCC6), mRNA. | -0,82356 | 9,68795 | -3,89429 | 0,00326 | 0,03547 |
| B3GNT6 | Homo sapiens UDP-GlcNAc:betaGal beta-1,3-N-acetylglucosaminyltransferase 6 (B3GNT6), mRNA. | -0,82548 | 7,93257 | -5,95472 | 0,00017 | 0,00772 |
| BCL2L12 | Homo sapiens BCL2-like 12 (proline rich) (BCL2L12), transcript variant 3, mRNA. | -0,82807 | 9,32352 | -7,11154 | 0,00004 | 0,00422 |
| FAM96A | Homo sapiens family with sequence similarity 96, member A (FAM96A), transcript variant 1, mRNA. | -0,83148 | 10,25528 | -6,36324 | 0,00010 | 0,00619 |
| LANCL2 | Homo sapiens LanC lantibiotic synthetase component C-like 2 (bacterial) (LANCL2), mRNA. | -0,83180 | 8,75417 | -5,03775 | 0,00058 | 0,01397 |
| POLE3 | Homo sapiens polymerase (DNA directed), epsilon 3 (p17 subunit) (POLE3), mRNA. | -0,83294 | 11,65568 | -5,69218 | 0,00024 | 0,00902 |
| DUT | Homo sapiens deoxyuridine triphosphatase (DUT), nuclear gene encoding mitochondrial protein, transcript variant 3, mRNA. | -0,83344 | 7,90515 | -4,46724 | 0,00135 | 0,02155 |
| BCL2L12 | Homo sapiens BCL2-like 12 (proline rich) (BCL2L12), transcript variant 3, mRNA. | -0,83572 | 9,48915 | -6,91923 | 0,00005 | 0,00457 |
| CALM3 | Homo sapiens calmodulin 3 (phosphorylase kinase, delta) (CALM3), mRNA. | -0,83831 | 11,28985 | -3,95634 | 0,00295 | 0,03346 |
| PRMT6 | Homo sapiens protein arginine methyltransferase 6 (PRMT6), mRNA. | -0,83847 | 8,22847 | -6,50268 | 0,00008 | 0,00579 |
| MYH10 | Homo sapiens myosin, heavy chain 10, non-muscle (MYH10), mRNA. | -0,83851 | 9,20954 | -3,56577 | 0,00551 | 0,04860 |
| PRPS1 | Homo sapiens phosphoribosyl pyrophosphate synthetase 1 (PRPS1), mRNA. | -0,83909 | 9,33557 | -3,64896 | 0,00482 | 0,04476 |
| FEN1 | Homo sapiens flap structure-specific endonuclease 1 (FEN1), mRNA. | -0,83949 | 8,22982 | -7,64218 | 0,00002 | 0,00334 |
| PKM2 | Homo sapiens pyruvate kinase, muscle (PKM2), transcript variant 1, mRNA. | -0,84203 | 9,62605 | -3,97772 | 0,00286 | 0,03294 |
| CCT3 | Homo sapiens chaperonin containing TCP1, subunit 3 (gamma) (CCT3), transcript variant 1, mRNA. | -0,84216 | 10,56645 | -5,17252 | 0,00048 | 0,01265 |
| CENPM | Homo sapiens centromere protein M (CENPM), transcript variant 1, mRNA. | -0,84579 | 7,81101 | -5,71990 | 0,00023 | 0,00888 |
| SLC2A5 | Homo sapiens solute carrier family 2 (facilitated glucose/fructose transporter), member 5 (SLC2A5), mRNA. | -0,84649 | 7,56329 | -5,57376 | 0,00028 | 0,00973 |
| LOC728554 | PREDICTED: Homo sapiens similar to THO complex 3 (LOC728554), mRNA. | -0,84798 | 11,00211 | -6,55329 | 0,00008 | 0,00555 |
| ATP1B1 | Homo sapiens ATPase, Na+/K+ transporting, beta 1 polypeptide (ATP1B1), transcript variant 1, mRNA. | -0,85388 | 9,50575 | -4,98139 | 0,00063 | 0,01457 |
| HSPA2 | Homo sapiens heat shock 70kDa protein 2 (HSPA2), mRNA. | -0,85489 | 7,85475 | -9,10394 | 0,00001 | 0,00188 |
| SF3A3 | Homo sapiens splicing factor 3a, subunit 3, 60kDa (SF3A3), mRNA. | -0,85776 | 9,31549 | -4,90376 | 0,00071 | 0,01555 |
| SLC44A2 | Homo sapiens solute carrier family 44, member 2 (SLC44A2), mRNA. | -0,85867 | 9,22150 | -4,08977 | 0,00240 | 0,02966 |
| RPL6 | Homo sapiens ribosomal protein L6 (RPL6), transcript variant 1, mRNA. | -0,85942 | 9,60469 | -5,02673 | 0,00059 | 0,01410 |
| HNRNPM | Homo sapiens heterogeneous nuclear ribonucleoprotein M (HNRNPM), transcript variant 2, mRNA. | -0,85960 | 10,81307 | -6,58043 | 0,00008 | 0,00551 |
| TFDP1 | Homo sapiens transcription factor Dp-1 (TFDP1), mRNA. | -0,85963 | 10,34403 | -4,19803 | 0,00203 | 0,02680 |
| STAT1 | Homo sapiens signal transducer and activator of transcription 1, 91kDa (STAT1), transcript variant alpha, mRNA. | -0,86154 | 9,09145 | -3,59441 | 0,00526 | 0,04719 |
| TUBB | Homo sapiens tubulin, beta (TUBB), mRNA. | -0,86910 | 11,43105 | -4,28140 | 0,00178 | 0,02512 |
| GPR177 | Homo sapiens G protein-coupled receptor 177 (GPR177), transcript variant 2, mRNA. | -0,87147 | 9,02428 | -7,83301 | 0,00002 | 0,00322 |
| SLBP | Homo sapiens stem-loop binding protein (SLBP), mRNA. | -0,87171 | 8,96627 | -7,02598 | 0,00005 | 0,00442 |
| CKAP2 | Homo sapiens cytoskeleton associated protein 2 (CKAP2), transcript variant 2, mRNA. | -0,87364 | 8,43840 | -3,82579 | 0,00363 | 0,03768 |
| VSNL1 | Homo sapiens visinin-like 1 (VSNL1), mRNA. | -0,87415 | 7,78486 | -4,73890 | 0,00090 | 0,01739 |
| TRIM28 | Homo sapiens tripartite motif-containing 28 (TRIM28), mRNA. | -0,87431 | 9,51903 | -6,96665 | 0,00005 | 0,00448 |
| RARA | Homo sapiens retinoic acid receptor, alpha (RARA), transcript variant 1, mRNA. | -0,87519 | 8,52297 | -3,89361 | 0,00326 | 0,03547 |
| CDT1 | Homo sapiens chromatin licensing and DNA replication factor 1 (CDT1), mRNA. | -0,87529 | 7,90244 | -6,70703 | 0,00007 | 0,00494 |
| PKM2 | Homo sapiens pyruvate kinase, muscle (PKM2), transcript variant 2, mRNA. | -0,87807 | 10,50614 | -3,61881 | 0,00506 | 0,04608 |
| C18orf55 | Homo sapiens chromosome 18 open reading frame 55 (C18orf55), mRNA. | -0,87997 | 8,70519 | -7,27027 | 0,00003 | 0,00401 |
| CDC25A | Homo sapiens cell division cycle 25 homolog A (S. pombe) (CDC25A), transcript variant 1, mRNA. | -0,88020 | 8,07148 | -5,76188 | 0,00022 | 0,00858 |
| LOC728026 | PREDICTED: Homo sapiens hypothetical LOC728026 (LOC728026), mRNA. | -0,88503 | 8,90135 | -4,29860 | 0,00174 | 0,02484 |
| HNRNPR | Homo sapiens heterogeneous nuclear ribonucleoprotein R (HNRNPR), transcript variant 2, mRNA. | -0,88600 | 11,12956 | -7,26114 | 0,00003 | 0,00401 |
| CDK4 | Homo sapiens cyclin-dependent kinase 4 (CDK4), mRNA. | -0,88601 | 10,10354 | -4,01771 | 0,00268 | 0,03172 |
| PPIH | Homo sapiens peptidylprolyl isomerase H (cyclophilin H) (PPIH), mRNA. | -0,88756 | 9,21786 | -3,95917 | 0,00294 | 0,03343 |
| PTMA | Homo sapiens prothymosin, alpha (PTMA), transcript variant 1, mRNA. | -0,88842 | 9,52129 | -3,75119 | 0,00409 | 0,04054 |
| TRIM6 | Homo sapiens tripartite motif-containing 6 (TRIM6), transcript variant 2, mRNA. | -0,88911 | 7,80046 | -12,19994 | 0,00000 | 0,00059 |
| SNRPF | Homo sapiens small nuclear ribonucleoprotein polypeptide F (SNRPF), mRNA. | -0,89040 | 11,45882 | -3,70242 | 0,00442 | 0,04252 |
| CBX6 | Homo sapiens chromobox homolog 6 (CBX6), mRNA. | -0,89217 | 9,60691 | -3,84728 | 0,00351 | 0,03708 |
| PSMA2 | Homo sapiens proteasome (prosome, macropain) subunit, alpha type, 2 (PSMA2), mRNA. | -0,89773 | 8,39015 | -4,79063 | 0,00083 | 0,01675 |
| ACN9 | Homo sapiens ACN9 homolog (S. cerevisiae) (ACN9), mRNA. | -0,90204 | 8,51288 | -5,14857 | 0,00050 | 0,01281 |
| SKP2 | Homo sapiens S-phase kinase-associated protein 2 (p45) (SKP2), transcript variant 1, mRNA. | -0,90287 | 7,82782 | -6,98289 | 0,00005 | 0,00446 |
| LOC100130561 | PREDICTED: Homo sapiens similar to high-mobility group (nonhistone chromosomal) protein 1-like 10, transcript variant 2 (LOC100130561), mRNA. | -0,90396 | 10,19856 | -4,16796 | 0,00212 | 0,02751 |
| HNRPR | Homo sapiens heterogeneous nuclear ribonucleoprotein R (HNRPR), mRNA. | -0,90667 | 10,28500 | -6,76838 | 0,00006 | 0,00481 |
| SHMT1 | Homo sapiens serine hydroxymethyltransferase 1 (soluble) (SHMT1), transcript variant 1, mRNA. | -0,90831 | 8,03540 | -5,52358 | 0,00030 | 0,01002 |
| LOC653874 | PREDICTED: Homo sapiens similar to Dihydrofolate reductase, transcript variant 1 (LOC653874), mRNA. | -0,90956 | 8,21209 | -4,02549 | 0,00265 | 0,03154 |
| TFDP1 | Homo sapiens transcription factor Dp-1 (TFDP1), mRNA. | -0,91393 | 10,33395 | -6,22734 | 0,00012 | 0,00675 |
| SPRY1 | Homo sapiens sprouty homolog 1, antagonist of FGF signaling (Drosophila) (SPRY1), transcript variant 1, mRNA. | -0,91462 | 7,90724 | -5,02864 | 0,00059 | 0,01408 |
| FZD2 | Homo sapiens frizzled homolog 2 (Drosophila) (FZD2), mRNA. | -0,91855 | 8,83499 | -7,67916 | 0,00002 | 0,00332 |
| RBM14 | Homo sapiens RNA binding motif protein 14 (RBM14), mRNA. | -0,91977 | 9,26021 | -4,80268 | 0,00082 | 0,01662 |
| ALDH3A2 | Homo sapiens aldehyde dehydrogenase 3 family, member A2 (ALDH3A2), transcript variant 2, mRNA. | -0,92173 | 8,73105 | -6,13320 | 0,00013 | 0,00704 |
| LIG1 | Homo sapiens ligase I, DNA, ATP-dependent (LIG1), mRNA. | -0,92304 | 8,14707 | -4,42215 | 0,00144 | 0,02246 |
| PPIL5 | Homo sapiens peptidylprolyl isomerase (cyclophilin)-like 5 (PPIL5), transcript variant 3, mRNA. | -0,92362 | 8,58769 | -5,08528 | 0,00055 | 0,01344 |
| LOC643287 | PREDICTED: Homo sapiens similar to prothymosin alpha, transcript variant 1 (LOC643287), mRNA. | -0,92545 | 8,69635 | -4,53690 | 0,00121 | 0,02030 |
| LOC727761 | PREDICTED: Homo sapiens similar to Deoxythymidylate kinase (thymidylate kinase), transcript variant 4 (LOC727761), mRNA. | -0,92614 | 9,66186 | -4,23689 | 0,00191 | 0,02611 |
| SIVA | Homo sapiens CD27-binding (Siva) protein (SIVA), transcript variant 1, mRNA. | -0,92912 | 9,35151 | -5,82592 | 0,00020 | 0,00823 |
| VIPR1 | Homo sapiens vasoactive intestinal peptide receptor 1 (VIPR1), mRNA. | -0,92986 | 7,94874 | -4,86953 | 0,00074 | 0,01585 |
| GALNTL4 | Homo sapiens UDP-N-acetyl-alpha-D-galactosamine:polypeptide N-acetylgalactosaminyltransferase-like 4 (GALNTL4), mRNA. | -0,93048 | 8,26050 | -5,33691 | 0,00038 | 0,01132 |
| SIVA1 | Homo sapiens SIVA1, apoptosis-inducing factor (SIVA1), transcript variant 1, mRNA. | -0,93339 | 9,38294 | -5,16948 | 0,00049 | 0,01265 |
| PHB | Homo sapiens prohibitin (PHB), mRNA. | -0,93387 | 9,38793 | -4,16386 | 0,00214 | 0,02764 |
| RNF26 | Homo sapiens ring finger protein 26 (RNF26), mRNA. | -0,93660 | 8,71161 | -7,32913 | 0,00003 | 0,00393 |
| HN1 | Homo sapiens hematological and neurological expressed 1 (HN1), transcript variant 1, mRNA. | -0,93664 | 8,82907 | -6,16264 | 0,00013 | 0,00695 |
| LSM4 | Homo sapiens LSM4 homolog, U6 small nuclear RNA associated (S. cerevisiae) (LSM4), mRNA. | -0,93970 | 10,59295 | -3,71244 | 0,00435 | 0,04210 |
| POLA2 | Homo sapiens polymerase (DNA directed), alpha 2 (70kD subunit) (POLA2), mRNA. | -0,94035 | 8,18748 | -5,56043 | 0,00028 | 0,00982 |
| SFRS2 | Homo sapiens splicing factor, arginine/serine-rich 2 (SFRS2), mRNA. | -0,94158 | 11,72250 | -12,49160 | 0,00000 | 0,00056 |
| ATP5G1 | Homo sapiens ATP synthase, H+ transporting, mitochondrial F0 complex, subunit C1 (subunit 9) (ATP5G1), nuclear gene encoding mitochondrial protein, transcript variant 2, mRNA. | -0,94167 | 9,84426 | -4,13773 | 0,00223 | 0,02831 |
| MCM7 | Homo sapiens minichromosome maintenance complex component 7 (MCM7), transcript variant 1, mRNA. | -0,94445 | 8,20788 | -5,11177 | 0,00053 | 0,01314 |
| GLA | Homo sapiens galactosidase, alpha (GLA), mRNA. | -0,94612 | 9,52893 | -7,04521 | 0,00004 | 0,00438 |
| SPR | Homo sapiens sepiapterin reductase (7,8-dihydrobiopterin:NADP+ oxidoreductase) (SPR), mRNA. | -0,95110 | 8,29861 | -5,54860 | 0,00029 | 0,00992 |
| GGCT | Homo sapiens gamma-glutamyl cyclotransferase (GGCT), mRNA. | -0,95189 | 10,53229 | -4,54741 | 0,00119 | 0,02018 |
| GINS3 | Homo sapiens GINS complex subunit 3 (Psf3 homolog) (GINS3), mRNA. | -0,95840 | 8,61978 | -6,69838 | 0,00007 | 0,00497 |
| KIF22 | Homo sapiens kinesin family member 22 (KIF22), mRNA. | -0,95920 | 8,65639 | -3,91111 | 0,00317 | 0,03486 |
| TMPO | Homo sapiens thymopoietin (TMPO), transcript variant 1, mRNA. | -0,95997 | 8,19740 | -4,61684 | 0,00108 | 0,01919 |
| DLK2 | Homo sapiens delta-like 2 homolog (Drosophila) (DLK2), transcript variant 2, mRNA. | -0,95998 | 8,47966 | -4,49388 | 0,00129 | 0,02105 |
| ATP1B1 | Homo sapiens ATPase, Na+/K+ transporting, beta 1 polypeptide (ATP1B1), transcript variant 2, mRNA. | -0,96706 | 10,42939 | -3,94376 | 0,00301 | 0,03377 |
| LOC100133328 | PREDICTED: Homo sapiens misc_RNA (LOC100133328), miscRNA. | -0,97732 | 9,13177 | -5,01234 | 0,00061 | 0,01424 |
| ATAD2 | Homo sapiens ATPase family, AAA domain containing 2 (ATAD2), mRNA. | -0,97804 | 8,82681 | -6,37453 | 0,00010 | 0,00614 |
| PSRC1 | Homo sapiens proline/serine-rich coiled-coil 1 (PSRC1), transcript variant 4, mRNA. | -0,97936 | 8,53818 | -4,27748 | 0,00180 | 0,02520 |
| C18orf55 | Homo sapiens chromosome 18 open reading frame 55 (C18orf55), mRNA. | -0,98040 | 9,47928 | -7,02912 | 0,00005 | 0,00442 |
| DCTPP1 | Homo sapiens dCTP pyrophosphatase 1 (DCTPP1), mRNA. | -0,98809 | 10,50167 | -3,72814 | 0,00424 | 0,04156 |
| RPS15 | Homo sapiens ribosomal protein S15 (RPS15), mRNA. | -0,98924 | 9,34624 | -4,31772 | 0,00169 | 0,02442 |
| GBP6 | Homo sapiens guanylate binding protein family, member 6 (GBP6), mRNA. | -0,98945 | 8,03019 | -5,50679 | 0,00031 | 0,01015 |
| KIAA0114 | Homo sapiens KIAA0114 (KIAA0114), non-coding RNA. | -0,99003 | 9,20632 | -6,78664 | 0,00006 | 0,00479 |
| CCND3 | Homo sapiens cyclin D3 (CCND3), mRNA. | -0,99288 | 8,65625 | -5,36751 | 0,00037 | 0,01117 |
| APITD1 | Homo sapiens apoptosis-inducing, TAF9-like domain 1 (APITD1), transcript variant B, mRNA. | -0,99726 | 8,30041 | -5,67623 | 0,00024 | 0,00909 |
| NASP | Homo sapiens nuclear autoantigenic sperm protein (histone-binding) (NASP), transcript variant 2, mRNA. | -1,00213 | 7,80785 | -9,13300 | 0,00001 | 0,00188 |
| CYBASC3 | Homo sapiens cytochrome b, ascorbate dependent 3 (CYBASC3), mRNA. | -1,00614 | 8,49795 | -4,53188 | 0,00122 | 0,02038 |
| LOC100132299 | PREDICTED: Homo sapiens similar to MSTP075 (LOC100132299), mRNA. | -1,00966 | 9,06374 | -5,50101 | 0,00031 | 0,01017 |
| DNMT1 | Homo sapiens DNA (cytosine-5-)-methyltransferase 1 (DNMT1), mRNA. | -1,01240 | 10,88732 | -3,79374 | 0,00382 | 0,03889 |
| POLE2 | Homo sapiens polymerase (DNA directed), epsilon 2 (p59 subunit) (POLE2), mRNA. | -1,01688 | 8,06387 | -9,09312 | 0,00001 | 0,00188 |
| PRSS23 | Homo sapiens protease, serine, 23 (PRSS23), mRNA. | -1,02079 | 10,16457 | -3,78586 | 0,00387 | 0,03923 |
| SUPT16H | Homo sapiens suppressor of Ty 16 homolog (S. cerevisiae) (SUPT16H), mRNA. | -1,02454 | 10,31597 | -4,72166 | 0,00092 | 0,01757 |
| FOXM1 | Homo sapiens forkhead box M1 (FOXM1), transcript variant 2, mRNA. | -1,02717 | 8,40459 | -3,57567 | 0,00543 | 0,04818 |
| PNPO | Homo sapiens pyridoxamine 5'-phosphate oxidase (PNPO), mRNA. | -1,03201 | 9,26012 | -3,72642 | 0,00426 | 0,04164 |
| DEK | Homo sapiens DEK oncogene (DNA binding) (DEK), mRNA. | -1,04122 | 9,83207 | -4,02832 | 0,00264 | 0,03146 |
| MSH6 | Homo sapiens mutS homolog 6 (E. coli) (MSH6), mRNA. | -1,04143 | 9,75204 | -5,49623 | 0,00031 | 0,01018 |
| THOC4 | PREDICTED: Homo sapiens THO complex 4 (THOC4), mRNA. | -1,04284 | 10,02125 | -5,87162 | 0,00019 | 0,00797 |
| KIF11 | Homo sapiens kinesin family member 11 (KIF11), mRNA. | -1,04416 | 8,21313 | -4,45840 | 0,00136 | 0,02174 |
| PTDSS1 | Homo sapiens phosphatidylserine synthase 1 (PTDSS1), mRNA. | -1,04488 | 9,87644 | -4,25586 | 0,00186 | 0,02572 |
| SIVA1 | Homo sapiens SIVA1, apoptosis-inducing factor (SIVA1), transcript variant 2, mRNA. | -1,05189 | 10,80826 | -4,87065 | 0,00074 | 0,01585 |
| MCM10 | Homo sapiens minichromosome maintenance complex component 10 (MCM10), transcript variant 2, mRNA. | -1,06090 | 8,08543 | -7,43508 | 0,00003 | 0,00368 |
| GMNN | Homo sapiens geminin, DNA replication inhibitor (GMNN), mRNA. | -1,06238 | 8,39691 | -9,03981 | 0,00001 | 0,00190 |
| ATP1B1 | Homo sapiens ATPase, Na+/K+ transporting, beta 1 polypeptide (ATP1B1), transcript variant 1, mRNA. | -1,06454 | 9,53913 | -4,68890 | 0,00097 | 0,01793 |
| RDH16 | Homo sapiens retinol dehydrogenase 16 (all-trans) (RDH16), mRNA. | -1,06506 | 7,66475 | -4,07813 | 0,00244 | 0,02997 |
| RFC4 | Homo sapiens replication factor C (activator 1) 4, 37kDa (RFC4), transcript variant 1, mRNA. | -1,06739 | 10,59721 | -3,56523 | 0,00552 | 0,04861 |
| PYCARD | Homo sapiens PYD and CARD domain containing (PYCARD), transcript variant 1, mRNA. | -1,07048 | 9,80884 | -4,29478 | 0,00175 | 0,02488 |
| EXO1 | Homo sapiens exonuclease 1 (EXO1), transcript variant 1, mRNA. | -1,07479 | 8,21625 | -4,67184 | 0,00099 | 0,01817 |
| COQ3 | Homo sapiens coenzyme Q3 homolog, methyltransferase (S. cerevisiae) (COQ3), mRNA. | -1,07598 | 8,69849 | -4,67480 | 0,00099 | 0,01814 |
| RPA1 | Homo sapiens replication protein A1, 70kDa (RPA1), mRNA. | -1,07735 | 10,41761 | -4,45365 | 0,00137 | 0,02183 |
| STRA13 | Homo sapiens stimulated by retinoic acid 13 homolog (mouse) (STRA13), mRNA. | -1,08263 | 10,04281 | -7,52251 | 0,00003 | 0,00358 |
| SKA2 | Homo sapiens spindle and kinetochore associated complex subunit 2 (SKA2), transcript variant 1, mRNA. | -1,08486 | 9,35107 | -5,36613 | 0,00037 | 0,01117 |
| TNS3 | Homo sapiens tensin 3 (TNS3), mRNA. | -1,08887 | 9,17421 | -4,81165 | 0,00081 | 0,01652 |
| CPA4 | Homo sapiens carboxypeptidase A4 (CPA4), mRNA. | -1,08985 | 7,77709 | -6,41972 | 0,00009 | 0,00609 |
| ZWINT | Homo sapiens ZW10 interactor (ZWINT), transcript variant 3, mRNA. | -1,09113 | 8,09727 | -7,25396 | 0,00004 | 0,00401 |
| TIMELESS | Homo sapiens timeless homolog (Drosophila) (TIMELESS), mRNA. | -1,09407 | 8,74267 | -4,06630 | 0,00249 | 0,03032 |
| MGC40489 | PREDICTED: Homo sapiens hypothetical protein MGC40489 (MGC40489), misc RNA. | -1,09593 | 9,53273 | -4,18843 | 0,00206 | 0,02707 |
| RAD51C | Homo sapiens RAD51 homolog C (S. cerevisiae) (RAD51C), transcript variant 2, mRNA. | -1,10787 | 8,61349 | -4,80056 | 0,00082 | 0,01664 |
| NUSAP1 | Homo sapiens nucleolar and spindle associated protein 1 (NUSAP1), transcript variant 2, mRNA. | -1,10973 | 8,35637 | -3,82617 | 0,00363 | 0,03768 |
| PTPLB | Homo sapiens protein tyrosine phosphatase-like (proline instead of catalytic arginine), member b (PTPLB), mRNA. | -1,12115 | 10,12105 | -3,63878 | 0,00490 | 0,04520 |
| ARHGAP23 | PREDICTED: Homo sapiens Rho GTPase activating protein 23, transcript variant 1 (ARHGAP23), mRNA. | -1,12181 | 8,35844 | -5,67177 | 0,00024 | 0,00911 |
| KPNA2 | Homo sapiens karyopherin alpha 2 (RAG cohort 1, importin alpha 1) (KPNA2), mRNA. XM_001133262 XM_001133265 XM_001133267 XM_001133271 | -1,12201 | 9,45680 | -3,88967 | 0,00328 | 0,03557 |
| MYLIP | Homo sapiens myosin regulatory light chain interacting protein (MYLIP), mRNA. | -1,12715 | 7,93245 | -6,74877 | 0,00006 | 0,00485 |
| RRM2 | Homo sapiens ribonucleotide reductase M2 polypeptide (RRM2), mRNA. | -1,13014 | 8,53689 | -4,13151 | 0,00225 | 0,02850 |
| CCDC34 | Homo sapiens coiled-coil domain containing 34 (CCDC34), transcript variant 1, mRNA. | -1,13235 | 8,35604 | -3,62630 | 0,00500 | 0,04571 |
| PTPLB | Homo sapiens protein tyrosine phosphatase-like (proline instead of catalytic arginine), member b (PTPLB), mRNA. | -1,13454 | 9,79850 | -4,21020 | 0,00199 | 0,02654 |
| RPA1 | Homo sapiens replication protein A1, 70kDa (RPA1), mRNA. | -1,13577 | 9,22249 | -6,36109 | 0,00010 | 0,00619 |
| SNHG3-RCC1 | Homo sapiens SNHG3-RCC1 readthrough transcript (SNHG3-RCC1), transcript variant 1, mRNA. | -1,13675 | 9,18757 | -7,22635 | 0,00004 | 0,00407 |
| STMN1 | Homo sapiens stathmin 1 (STMN1), transcript variant 1, mRNA. | -1,13814 | 9,34094 | -3,66777 | 0,00468 | 0,04394 |
| DUT | Homo sapiens deoxyuridine triphosphatase (DUT), nuclear gene encoding mitochondrial protein, transcript variant 1, mRNA. | -1,14686 | 8,16686 | -4,32174 | 0,00168 | 0,02433 |
| RAD51AP1 | Homo sapiens RAD51 associated protein 1 (RAD51AP1), mRNA. | -1,15636 | 8,44565 | -4,01179 | 0,00271 | 0,03188 |
| PBK | Homo sapiens PDZ binding kinase (PBK), mRNA. | -1,16150 | 8,48569 | -3,54436 | 0,00571 | 0,04951 |
| MCM5 | Homo sapiens minichromosome maintenance complex component 5 (MCM5), mRNA. | -1,16905 | 8,88544 | -4,60648 | 0,00109 | 0,01934 |
| C16orf33 | Homo sapiens chromosome 16 open reading frame 33 (C16orf33), mRNA. | -1,17714 | 9,17529 | -5,81992 | 0,00020 | 0,00826 |
| E2F2 | Homo sapiens E2F transcription factor 2 (E2F2), mRNA. | -1,18034 | 7,71533 | -13,97392 | 0,00000 | 0,00056 |
| HADH | Homo sapiens hydroxyacyl-Coenzyme A dehydrogenase (HADH), nuclear gene encoding mitochondrial protein, mRNA. | -1,18382 | 8,55257 | -3,91985 | 0,00313 | 0,03455 |
| FAM111A | Homo sapiens family with sequence similarity 111, member A (FAM111A), transcript variant 1, mRNA. | -1,19203 | 9,01049 | -5,40120 | 0,00035 | 0,01091 |
| CCNF | Homo sapiens cyclin F (CCNF), mRNA. | -1,19576 | 8,79198 | -3,81031 | 0,00372 | 0,03830 |
| LOC728873 | PREDICTED: Homo sapiens misc_RNA (LOC728873), miscRNA. | -1,19945 | 11,08466 | -3,78822 | 0,00386 | 0,03910 |
| DPYSL3 | Homo sapiens dihydropyrimidinase-like 3 (DPYSL3), mRNA. | -1,20019 | 9,28791 | -3,75895 | 0,00404 | 0,04021 |
| HMGB2 | Homo sapiens high-mobility group box 2 (HMGB2), mRNA. | -1,20989 | 8,72458 | -3,88535 | 0,00330 | 0,03567 |
| MCM4 | Homo sapiens minichromosome maintenance complex component 4 (MCM4), transcript variant 2, mRNA. | -1,21351 | 8,69621 | -7,31638 | 0,00003 | 0,00394 |
| WNT10A | Homo sapiens wingless-type MMTV integration site family, member 10A (WNT10A), mRNA. | -1,21446 | 7,85990 | -5,05932 | 0,00057 | 0,01372 |
| ARHGDIB | Homo sapiens Rho GDP dissociation inhibitor (GDI) beta (ARHGDIB), mRNA. | -1,22748 | 8,23121 | -5,36998 | 0,00037 | 0,01116 |
| LYPD1 | Homo sapiens LY6/PLAUR domain containing 1 (LYPD1), transcript variant 1, mRNA. | -1,23220 | 7,87309 | -7,50312 | 0,00003 | 0,00360 |
| RHOBTB3 | Homo sapiens Rho-related BTB domain containing 3 (RHOBTB3), mRNA. | -1,24602 | 8,47771 | -4,68837 | 0,00097 | 0,01793 |
| MCM7 | Homo sapiens minichromosome maintenance complex component 7 (MCM7), transcript variant 2, mRNA. | -1,26061 | 11,81087 | -4,18056 | 0,00208 | 0,02720 |
| SNCA | Homo sapiens synuclein, alpha (non A4 component of amyloid precursor) (SNCA), transcript variant NACP112, mRNA. | -1,28026 | 9,07198 | -4,29036 | 0,00176 | 0,02496 |
| DLGAP5 | Homo sapiens discs, large (Drosophila) homolog-associated protein 5 (DLGAP5), mRNA. | -1,30731 | 8,53512 | -3,70742 | 0,00439 | 0,04228 |
| MCM4 | Homo sapiens minichromosome maintenance complex component 4 (MCM4), transcript variant 1, mRNA. | -1,33590 | 10,24411 | -5,29914 | 0,00041 | 0,01159 |
| FEN1 | Homo sapiens flap structure-specific endonuclease 1 (FEN1), mRNA. | -1,34379 | 9,97679 | -7,43500 | 0,00003 | 0,00368 |
| CXXC5 | Homo sapiens CXXC finger 5 (CXXC5), mRNA. | -1,34459 | 8,94696 | -3,87116 | 0,00338 | 0,03622 |
| ID3 | Homo sapiens inhibitor of DNA binding 3, dominant negative helix-loop-helix protein (ID3), mRNA. | -1,34630 | 7,88223 | -5,43046 | 0,00034 | 0,01065 |
| SOX2 | Homo sapiens SRY (sex determining region Y)-box 2 (SOX2), mRNA. | -1,34748 | 7,78983 | -13,77277 | 0,00000 | 0,00056 |
| UBE2T | Homo sapiens ubiquitin-conjugating enzyme E2T (putative) (UBE2T), mRNA. | -1,38162 | 9,20955 | -4,21729 | 0,00197 | 0,02641 |
| C18orf56 | Homo sapiens chromosome 18 open reading frame 56 (C18orf56), mRNA. | -1,38816 | 8,68268 | -4,36646 | 0,00157 | 0,02352 |
| LOC100134073 | PREDICTED: Homo sapiens similar to LYPDC1 protein (LOC100134073), mRNA. | -1,39755 | 7,93575 | -6,74406 | 0,00006 | 0,00487 |
| FBXO5 | Homo sapiens F-box protein 5 (FBXO5), mRNA. | -1,40094 | 8,62934 | -6,45806 | 0,00009 | 0,00594 |
| PRIM1 | Homo sapiens primase, DNA, polypeptide 1 (49kDa) (PRIM1), mRNA. | -1,40157 | 8,59439 | -5,85367 | 0,00019 | 0,00807 |
| CENPM | Homo sapiens centromere protein M (CENPM), transcript variant 2, mRNA. | -1,40177 | 8,36364 | -5,43843 | 0,00033 | 0,01058 |
| PRIM1 | Homo sapiens primase, DNA, polypeptide 1 (49kDa) (PRIM1), mRNA. | -1,40594 | 8,58022 | -5,81390 | 0,00020 | 0,00827 |
| DLL1 | Homo sapiens delta-like 1 (Drosophila) (DLL1), mRNA. | -1,42178 | 9,63964 | -5,91373 | 0,00018 | 0,00775 |
| SPC24 | Homo sapiens SPC24, NDC80 kinetochore complex component, homolog (S. cerevisiae) (SPC24), mRNA. | -1,45597 | 8,60090 | -5,48724 | 0,00031 | 0,01025 |
| PCNA | Homo sapiens proliferating cell nuclear antigen (PCNA), transcript variant 2, mRNA. | -1,46379 | 9,93221 | -5,25385 | 0,00043 | 0,01192 |
| UHRF1 | Homo sapiens ubiquitin-like with PHD and ring finger domains 1 (UHRF1), transcript variant 1, mRNA. | -1,46879 | 9,28847 | -5,98083 | 0,00016 | 0,00755 |
| RFC5 | Homo sapiens replication factor C (activator 1) 5, 36.5kDa (RFC5), transcript variant 1, mRNA. | -1,47004 | 9,21453 | -4,07021 | 0,00247 | 0,03025 |
| NUSAP1 | Homo sapiens nucleolar and spindle associated protein 1 (NUSAP1), transcript variant 2, mRNA. | -1,47035 | 8,86427 | -3,53996 | 0,00575 | 0,04973 |
| SOX2 | Homo sapiens SRY (sex determining region Y)-box 2 (SOX2), mRNA. | -1,50535 | 7,90564 | -13,69498 | 0,00000 | 0,00056 |
| OIP5 | Homo sapiens Opa interacting protein 5 (OIP5), mRNA. | -1,50799 | 8,89579 | -3,81369 | 0,00370 | 0,03820 |
| HSPE1 | Homo sapiens heat shock 10kDa protein 1 (chaperonin 10) (HSPE1), mRNA. | -1,51478 | 9,31408 | -4,00346 | 0,00274 | 0,03212 |
| ASF1B | Homo sapiens ASF1 anti-silencing function 1 homolog B (S. cerevisiae) (ASF1B), mRNA. | -1,56328 | 8,54884 | -5,53653 | 0,00029 | 0,00999 |
| MCM6 | Homo sapiens minichromosome maintenance complex component 6 (MCM6), mRNA. | -1,56408 | 10,05468 | -4,79684 | 0,00083 | 0,01665 |
| CXXC5 | Homo sapiens CXXC finger 5 (CXXC5), mRNA. | -1,57060 | 9,23865 | -3,83573 | 0,00358 | 0,03740 |
| VSNL1 | Homo sapiens visinin-like 1 (VSNL1), mRNA. | -1,59147 | 8,49826 | -4,80194 | 0,00082 | 0,01662 |
| MCM2 | Homo sapiens minichromosome maintenance complex component 2 (MCM2), mRNA. | -1,61621 | 8,83378 | -6,18275 | 0,00013 | 0,00687 |
| CDCA7 | Homo sapiens cell division cycle associated 7 (CDCA7), transcript variant 1, mRNA. | -1,61895 | 8,87263 | -4,56461 | 0,00116 | 0,01991 |
| BIRC5 | Homo sapiens baculoviral IAP repeat-containing 5 (BIRC5), transcript variant 1, mRNA. | -1,62838 | 8,92160 | -3,87145 | 0,00338 | 0,03622 |
| MCM3 | Homo sapiens minichromosome maintenance complex component 3 (MCM3), mRNA. | -1,62980 | 11,67124 | -4,77656 | 0,00085 | 0,01692 |
| SKP2 | Homo sapiens S-phase kinase-associated protein 2 (p45) (SKP2), transcript variant 2, mRNA. | -1,63226 | 9,34212 | -5,32095 | 0,00039 | 0,01138 |
| LOC731314 | PREDICTED: Homo sapiens similar to H2A histone family, member X (LOC731314), mRNA. | -1,67563 | 9,78743 | -5,81289 | 0,00020 | 0,00827 |
| H2AFX | Homo sapiens H2A histone family, member X (H2AFX), mRNA. | -1,71358 | 8,95182 | -7,45458 | 0,00003 | 0,00368 |
| UNG | Homo sapiens uracil-DNA glycosylase (UNG), nuclear gene encoding mitochondrial protein, transcript variant 1, mRNA. | -1,72087 | 9,96237 | -3,85203 | 0,00348 | 0,03698 |
| KIAA0101 | Homo sapiens KIAA0101 (KIAA0101), transcript variant 1, mRNA. | -1,77381 | 9,43264 | -3,67671 | 0,00461 | 0,04357 |
| KIF20A | Homo sapiens kinesin family member 20A (KIF20A), mRNA. | -1,80499 | 8,72110 | -4,03337 | 0,00262 | 0,03135 |
| LFNG | Homo sapiens LFNG O-fucosylpeptide 3-beta-N-acetylglucosaminyltransferase (LFNG), transcript variant 1, mRNA. | -1,80804 | 8,31307 | -3,56076 | 0,00556 | 0,04878 |
| GINS2 | Homo sapiens GINS complex subunit 2 (Psf2 homolog) (GINS2), mRNA. | -1,81542 | 9,50542 | -4,61404 | 0,00108 | 0,01923 |
| FAM83D | Homo sapiens family with sequence similarity 83, member D (FAM83D), mRNA. | -1,85426 | 9,11980 | -4,12330 | 0,00228 | 0,02873 |
| MCM3 | Homo sapiens minichromosome maintenance complex component 3 (MCM3), mRNA. | -1,86503 | 9,83676 | -6,31528 | 0,00011 | 0,00638 |
| TYMS | Homo sapiens thymidylate synthetase (TYMS), mRNA. | -1,93363 | 10,23492 | -3,93639 | 0,00305 | 0,03398 |
