# Supplementary material for: Induction of ER and mitochondrial stress by the alkylphosphocholine erufosine in oral squamous cell carcinoma cells
Source: Cell Death Dis. 2018 Feb 20;9(3):296. doi: 10.1038/s41419-018-0342-2 (PMC5833417; doi:10.1038/s41419-018-0342-2)
Supplement: Supplementary file 3 — Supplementary Table 1c [file 41419_2018_342_MOESM3_ESM.docx]

**Table S1c: Differential regulation of genes upon IC75 exposure of erufosine in HN-5 cells**

| **Symbol** | **Definition** | **Log Fold Change** | | **Average Expression** | | **t-statistics** | | **P.Value** | **adj.P.Val** | |
| --- | --- | --- | --- | --- | --- | --- | --- | --- | --- | --- |
| RN5S9 | Homo sapiens RNA, 5S ribosomal 9 (RN5S9), ribosomal RNA. | 4,3894 | 9,3159 | | 5,6672 | | 0,000246 | | | 0,00365 |
| CSF2 | Homo sapiens colony stimulating factor 2 (granulocyte-macrophage) (CSF2), mRNA. | 4,1699 | 9,6557 | | 9,9129 | | 0,000002 | | | 0,00046 |
| HBEGF | Homo sapiens heparin-binding EGF-like growth factor (HBEGF), mRNA. | 4,1647 | 10,4967 | | 9,5953 | | 0,000003 | | | 0,00050 |
| KLF6 | Homo sapiens Kruppel-like factor 6 (KLF6), transcript variant 2, mRNA. | 4,1327 | 11,1617 | | 13,0751 | | 0,000000 | | | 0,00017 |
| KLF6 | Homo sapiens Kruppel-like factor 6 (KLF6), transcript variant 1, mRNA. | 4,1203 | 10,3859 | | 12,2473 | | 0,000000 | | | 0,00024 |
| LOC100008589 | Homo sapiens 28S ribosomal RNA (LOC100008589), non-coding RNA. | 4,0272 | 11,0800 | | 8,9278 | | 0,000006 | | | 0,00064 |
| RN7SK | Homo sapiens RNA, 7SK small nuclear (RN7SK), non-coding RNA. | 3,9823 | 9,0849 | | 6,4862 | | 0,000086 | | | 0,00208 |
| CDKN1A | Homo sapiens cyclin-dependent kinase inhibitor 1A (p21, Cip1) (CDKN1A), transcript variant 1, mRNA. | 3,8522 | 11,5096 | | 10,3817 | | 0,000002 | | | 0,00041 |
| LOC100132564 | PREDICTED: Homo sapiens hypothetical protein LOC100132564 (LOC100132564), mRNA. | 3,7945 | 11,2385 | | 9,5223 | | 0,000003 | | | 0,00051 |
| LOC100134364 | PREDICTED: Homo sapiens hypothetical protein LOC100134364 (LOC100134364), mRNA. | 3,7553 | 10,9370 | | 9,9786 | | 0,000002 | | | 0,00045 |
| SNORD3D | Homo sapiens small nucleolar RNA, C/D box 3D (SNORD3D), small nucleolar RNA. | 3,6214 | 8,9375 | | 5,9920 | | 0,000161 | | | 0,00290 |
| RN7SK | Homo sapiens RNA, 7SK small nuclear (RN7SK), non-coding RNA. | 3,4830 | 8,4852 | | 5,7944 | | 0,000208 | | | 0,00333 |
| LOC100132394 | PREDICTED: Homo sapiens hypothetical protein LOC100132394 (LOC100132394), mRNA. | 3,4603 | 11,3748 | | 9,5833 | | 0,000003 | | | 0,00051 |
| LOC650517 | PREDICTED: Homo sapiens hypothetical LOC650517 (LOC650517), mRNA. | 3,4371 | 10,7364 | | 6,6631 | | 0,000070 | | | 0,00187 |
| MMP10 | Homo sapiens matrix metallopeptidase 10 (stromelysin 2) (MMP10), mRNA. | 3,3995 | 10,7643 | | 6,7313 | | 0,000064 | | | 0,00181 |
| TRIB1 | Homo sapiens tribbles homolog 1 (Drosophila) (TRIB1), mRNA. | 3,3515 | 10,4005 | | 8,3580 | | 0,000011 | | | 0,00083 |
| ANGPTL4 | Homo sapiens angiopoietin-like 4 (ANGPTL4), transcript variant 1, mRNA. | 3,3283 | 9,8363 | | 8,0706 | | 0,000014 | | | 0,00095 |
| RHOB | Homo sapiens ras homolog gene family, member B (RHOB), mRNA. | 3,2696 | 10,1512 | | 11,7296 | | 0,000001 | | | 0,00028 |
| LOC100008588 | Homo sapiens 18S ribosomal RNA (LOC100008588), non-coding RNA. | 3,2277 | 11,3053 | | 8,8540 | | 0,000007 | | | 0,00066 |
| KLF2 | Homo sapiens Kruppel-like factor 2 (lung) (KLF2), mRNA. | 3,2090 | 9,6186 | | 14,4757 | | 0,000000 | | | 0,00012 |
| ODC1 | Homo sapiens ornithine decarboxylase 1 (ODC1), mRNA. | 3,2069 | 11,9140 | | 9,4851 | | 0,000004 | | | 0,00052 |
| DUSP1 | Homo sapiens dual specificity phosphatase 1 (DUSP1), mRNA. | 3,0925 | 9,0437 | | 13,5513 | | 0,000000 | | | 0,00014 |
| PLAUR | Homo sapiens plasminogen activator, urokinase receptor (PLAUR), transcript variant 2, mRNA. | 3,0765 | 9,5754 | | 9,3141 | | 0,000004 | | | 0,00054 |
| MMP1 | Homo sapiens matrix metallopeptidase 1 (interstitial collagenase) (MMP1), mRNA. | 3,0626 | 9,2878 | | 3,3516 | | 0,007822 | | | 0,03355 |
| PPP1R15A | Homo sapiens protein phosphatase 1, regulatory (inhibitor) subunit 15A (PPP1R15A), mRNA. | 3,0322 | 10,9795 | | 9,9658 | | 0,000002 | | | 0,00045 |
| DUSP5 | Homo sapiens dual specificity phosphatase 5 (DUSP5), mRNA. | 3,0301 | 10,4625 | | 13,2921 | | 0,000000 | | | 0,00015 |
| IL8 | Homo sapiens interleukin 8 (IL8), mRNA. | 3,0027 | 8,6715 | | 15,4566 | | 0,000000 | | | 0,00012 |
| RNU1A3 | Homo sapiens RNA, U1A3 small nuclear (RNU1A3), small nuclear RNA. | 2,9500 | 9,1661 | | 7,7158 | | 0,000021 | | | 0,00110 |
| SNORD3A | Homo sapiens small nucleolar RNA, C/D box 3A (SNORD3A), small nucleolar RNA. | 2,7911 | 8,5051 | | 6,0494 | | 0,000149 | | | 0,00277 |
| IL1B | Homo sapiens interleukin 1, beta (IL1B), mRNA. | 2,7883 | 11,0396 | | 3,7000 | | 0,004441 | | | 0,02220 |
| AKR1C4 | Homo sapiens aldo-keto reductase family 1, member C4 (chlordecone reductase; 3-alpha hydroxysteroid dehydrogenase, type I; dihydrodiol dehydrogenase 4) (AKR1C4), mRNA. | 2,7686 | 9,3728 | | 3,9797 | | 0,002848 | | | 0,01634 |
| ATF3 | Homo sapiens activating transcription factor 3 (ATF3), transcript variant 4, mRNA. | 2,7429 | 8,5891 | | 18,5281 | | 0,000000 | | | 0,00007 |
| TM4SF19 | Homo sapiens transmembrane 4 L six family member 19 (TM4SF19), mRNA. | 2,7275 | 10,4661 | | 6,1959 | | 0,000124 | | | 0,00248 |
| PLAUR | Homo sapiens plasminogen activator, urokinase receptor (PLAUR), transcript variant 1, mRNA. | 2,7097 | 9,1909 | | 8,9647 | | 0,000006 | | | 0,00064 |
| GRB7 | Homo sapiens growth factor receptor-bound protein 7 (GRB7), transcript variant 2, mRNA. | 2,6805 | 9,2190 | | 11,0707 | | 0,000001 | | | 0,00031 |
| PHLDA1 | Homo sapiens pleckstrin homology-like domain, family A, member 1 (PHLDA1), mRNA. | 2,6691 | 12,2499 | | 10,9909 | | 0,000001 | | | 0,00031 |
| RNU1F1 | Homo sapiens RNA, U1F1 small nuclear (RNU1F1), small nuclear RNA. | 2,6688 | 8,5430 | | 6,8514 | | 0,000056 | | | 0,00170 |
| ZFP36 | Homo sapiens zinc finger protein 36, C3H type, homolog (mouse) (ZFP36), mRNA. | 2,6254 | 9,9574 | | 10,9458 | | 0,000001 | | | 0,00031 |
| RNU1-5 | Homo sapiens RNA, U1 small nuclear 5 (RNU1-5), small nuclear RNA. | 2,6193 | 8,6685 | | 7,4713 | | 0,000027 | | | 0,00122 |
| LAMC2 | Homo sapiens laminin, gamma 2 (LAMC2), transcript variant 1, mRNA. | 2,6044 | 11,2664 | | 6,1752 | | 0,000127 | | | 0,00252 |
| RNU1-3 | Homo sapiens RNA, U1 small nuclear 3 (RNU1-3), small nuclear RNA. | 2,5821 | 8,5414 | | 8,0609 | | 0,000015 | | | 0,00095 |
| BTG1 | Homo sapiens B-cell translocation gene 1, anti-proliferative (BTG1), mRNA. | 2,5793 | 10,9394 | | 10,9973 | | 0,000001 | | | 0,00031 |
| IL11 | Homo sapiens interleukin 11 (IL11), mRNA. | 2,5387 | 8,6676 | | 10,5718 | | 0,000001 | | | 0,00037 |
| KRT17P3 | PREDICTED: Homo sapiens misc_RNA (KRT17P3), miscRNA. | 2,4805 | 11,8804 | | 5,4923 | | 0,000311 | | | 0,00416 |
| SNORD3C | Homo sapiens small nucleolar RNA, C/D box 3C (SNORD3C), small nucleolar RNA. | 2,4605 | 8,1487 | | 4,4074 | | 0,001473 | | | 0,01053 |
| RNU1G2 | Homo sapiens RNA, U1G2 small nuclear (RNU1G2), small nuclear RNA. | 2,4450 | 8,4628 | | 7,6002 | | 0,000024 | | | 0,00116 |
| KRT16 | Homo sapiens keratin 16 (focal non-epidermolytic palmoplantar keratoderma) (KRT16), mRNA. | 2,4315 | 11,0847 | | 5,1102 | | 0,000528 | | | 0,00559 |
| GJB3 | Homo sapiens gap junction protein, beta 3, 31kDa (GJB3), transcript variant 2, mRNA. | 2,4280 | 10,1282 | | 8,0681 | | 0,000014 | | | 0,00095 |
| KCNK1 | Homo sapiens potassium channel, subfamily K, member 1 (KCNK1), mRNA. | 2,3996 | 9,7429 | | 6,8900 | | 0,000053 | | | 0,00167 |
| TRIB3 | Homo sapiens tribbles homolog 3 (Drosophila) (TRIB3), mRNA. | 2,3729 | 9,9578 | | 6,3485 | | 0,000102 | | | 0,00226 |
| PIM1 | Homo sapiens pim-1 oncogene (PIM1), mRNA. | 2,3648 | 9,1202 | | 10,9335 | | 0,000001 | | | 0,00031 |
| SPRY2 | Homo sapiens sprouty homolog 2 (Drosophila) (SPRY2), mRNA. | 2,3598 | 9,1100 | | 4,5929 | | 0,001116 | | | 0,00880 |
| LAMC2 | Homo sapiens laminin, gamma 2 (LAMC2), transcript variant 1, mRNA. | 2,3490 | 11,9187 | | 5,1617 | | 0,000491 | | | 0,00537 |
| KCNK1 | Homo sapiens potassium channel, subfamily K, member 1 (KCNK1), mRNA. | 2,3429 | 9,2391 | | 6,0039 | | 0,000158 | | | 0,00287 |
| AKAP12 | Homo sapiens A kinase (PRKA) anchor protein (gravin) 12 (AKAP12), transcript variant 2, mRNA. | 2,3311 | 8,9075 | | 8,3845 | | 0,000010 | | | 0,00082 |
| ISG20 | Homo sapiens interferon stimulated exonuclease gene 20kDa (ISG20), mRNA. | 2,3268 | 9,9159 | | 5,7479 | | 0,000221 | | | 0,00345 |
| C12orf35 | Homo sapiens chromosome 12 open reading frame 35 (C12orf35), mRNA. | 2,3151 | 8,9975 | | 7,0199 | | 0,000046 | | | 0,00153 |
| SAT1 | Homo sapiens spermidine/spermine N1-acetyltransferase 1 (SAT1), mRNA. | 2,3029 | 11,0708 | | 8,6570 | | 0,000008 | | | 0,00073 |
| AKR1C2 | Homo sapiens aldo-keto reductase family 1, member C2 (dihydrodiol dehydrogenase 2; bile acid binding protein; 3-alpha hydroxysteroid dehydrogenase, type III) (AKR1C2), transcript variant 1, mRNA. XM_943424 XM_943425 XM_943427 | 2,2986 | 9,8093 | | 4,2468 | | 0,001881 | | | 0,01234 |
| ULK1 | Homo sapiens unc-51-like kinase 1 (C. elegans) (ULK1), mRNA. | 2,2728 | 10,1553 | | 6,1874 | | 0,000125 | | | 0,00250 |
| NDRG1 | Homo sapiens N-myc downstream regulated gene 1 (NDRG1), mRNA. | 2,2528 | 12,3785 | | 5,5348 | | 0,000294 | | | 0,00403 |
| ULK1 | Homo sapiens unc-51-like kinase 1 (C. elegans) (ULK1), mRNA. | 2,2485 | 10,4458 | | 6,2071 | | 0,000122 | | | 0,00247 |
| JUN | Homo sapiens jun oncogene (JUN), mRNA. | 2,2431 | 10,7976 | | 10,9756 | | 0,000001 | | | 0,00031 |
| MMP9 | Homo sapiens matrix metallopeptidase 9 (gelatinase B, 92kDa gelatinase, 92kDa type IV collagenase) (MMP9), mRNA. | 2,2418 | 8,8409 | | 5,9341 | | 0,000173 | | | 0,00302 |
| LAMC2 | Homo sapiens laminin, gamma 2 (LAMC2), transcript variant 2, mRNA. | 2,2363 | 9,5930 | | 5,0739 | | 0,000555 | | | 0,00575 |
| ERRFI1 | Homo sapiens ERBB receptor feedback inhibitor 1 (ERRFI1), mRNA. | 2,2335 | 10,5358 | | 7,8950 | | 0,000017 | | | 0,00100 |
| ITGA2 | Homo sapiens integrin, alpha 2 (CD49B, alpha 2 subunit of VLA-2 receptor) (ITGA2), mRNA. | 2,2219 | 10,8469 | | 8,1451 | | 0,000013 | | | 0,00091 |
| TMBIM1 | Homo sapiens transmembrane BAX inhibitor motif containing 1 (TMBIM1), mRNA. | 2,1793 | 9,5548 | | 5,9776 | | 0,000164 | | | 0,00292 |
| TM4SF19 | PREDICTED: Homo sapiens transmembrane 4 L six family member 19, transcript variant 2 (TM4SF19), mRNA. | 2,1605 | 9,5714 | | 4,4025 | | 0,001484 | | | 0,01058 |
| RAP1GAP | Homo sapiens RAP1 GTPase activating protein (RAP1GAP), mRNA. | 2,1595 | 9,3666 | | 9,6255 | | 0,000003 | | | 0,00050 |
| LAMB3 | Homo sapiens laminin, beta 3 (LAMB3), transcript variant 1, mRNA. | 2,1589 | 12,8511 | | 9,2594 | | 0,000004 | | | 0,00055 |
| TNFRSF25 | Homo sapiens tumor necrosis factor receptor superfamily, member 25 (TNFRSF25), transcript variant 10, mRNA. | 2,1517 | 9,3592 | | 6,6994 | | 0,000067 | | | 0,00183 |
| PLAU | Homo sapiens plasminogen activator, urokinase (PLAU), mRNA. | 2,1492 | 12,7476 | | 11,7572 | | 0,000001 | | | 0,00028 |
| EMP1 | Homo sapiens epithelial membrane protein 1 (EMP1), mRNA. | 2,1270 | 10,7881 | | 10,2858 | | 0,000002 | | | 0,00041 |
| MYADM | Homo sapiens myeloid-associated differentiation marker (MYADM), transcript variant 4, mRNA. | 2,1187 | 9,4204 | | 14,2177 | | 0,000000 | | | 0,00012 |
| KRT17 | Homo sapiens keratin 17 (KRT17), mRNA. | 2,1127 | 12,2382 | | 5,6496 | | 0,000252 | | | 0,00370 |
| C1orf106 | Homo sapiens chromosome 1 open reading frame 106 (C1orf106), mRNA. | 2,1041 | 9,5432 | | 8,0168 | | 0,000015 | | | 0,00095 |
| PRDM1 | Homo sapiens PR domain containing 1, with ZNF domain (PRDM1), transcript variant 1, mRNA. | 2,1018 | 8,2578 | | 3,9400 | | 0,003031 | | | 0,01699 |
| SERPINE1 | Homo sapiens serpin peptidase inhibitor, clade E (nexin, plasminogen activator inhibitor type 1), member 1 (SERPINE1), mRNA. | 2,0935 | 10,0778 | | 7,0146 | | 0,000046 | | | 0,00153 |
| RNU4-2 | Homo sapiens RNA, U4 small nuclear 2 (RNU4-2), small nuclear RNA. | 2,0730 | 7,9985 | | 5,0948 | | 0,000539 | | | 0,00565 |
| RNF19B | Homo sapiens ring finger protein 19B (RNF19B), mRNA. | 2,0398 | 9,3291 | | 8,8742 | | 0,000006 | | | 0,00065 |
| MIR1974 | Homo sapiens microRNA 1974 (MIR1974), microRNA. | 2,0349 | 11,8289 | | 16,8790 | | 0,000000 | | | 0,00008 |
| C14orf78 | PREDICTED: Homo sapiens chromosome 14 open reading frame 78 (C14orf78), mRNA. | 2,0312 | 9,3584 | | 6,5277 | | 0,000082 | | | 0,00202 |
| ARHGEF2 | Homo sapiens rho/rac guanine nucleotide exchange factor (GEF) 2 (ARHGEF2), mRNA. | 2,0231 | 10,1990 | | 8,9882 | | 0,000006 | | | 0,00064 |
| MXD1 | Homo sapiens MAX dimerization protein 1 (MXD1), mRNA. | 1,9935 | 8,6646 | | 9,3974 | | 0,000004 | | | 0,00052 |
| IER3 | Homo sapiens immediate early response 3 (IER3), mRNA. | 1,9812 | 12,7999 | | 6,2761 | | 0,000112 | | | 0,00237 |
| TSC22D1 | Homo sapiens TSC22 domain family, member 1 (TSC22D1), transcript variant 2, mRNA. | 1,9588 | 10,1353 | | 6,1083 | | 0,000139 | | | 0,00267 |
| SERPINB1 | Homo sapiens serpin peptidase inhibitor, clade B (ovalbumin), member 1 (SERPINB1), mRNA. | 1,9587 | 8,9334 | | 3,3492 | | 0,007853 | | | 0,03362 |
| IL8 | Homo sapiens interleukin 8 (IL8), mRNA. | 1,9489 | 8,1334 | | 15,4658 | | 0,000000 | | | 0,00012 |
| ARID3B | Homo sapiens AT rich interactive domain 3B (BRIGHT-like) (ARID3B), mRNA. | 1,9420 | 8,4517 | | 6,2338 | | 0,000118 | | | 0,00243 |
| LOC441763 | PREDICTED: Homo sapiens hypothetical LOC441763 (LOC441763), mRNA. | 1,9325 | 9,4187 | | 5,6384 | | 0,000256 | | | 0,00373 |
| LOC100008589 | Homo sapiens 28S ribosomal RNA (LOC100008589), non-coding RNA. | 1,9277 | 12,9510 | | 8,7497 | | 0,000007 | | | 0,00069 |
| PLAUR | Homo sapiens plasminogen activator, urokinase receptor (PLAUR), transcript variant 2, mRNA. | 1,9238 | 8,6527 | | 5,8657 | | 0,000189 | | | 0,00317 |
| LOC100133565 | PREDICTED: Homo sapiens similar to hCG23738 (LOC100133565), mRNA. | 1,9220 | 9,6164 | | 5,6007 | | 0,000269 | | | 0,00385 |
| PMAIP1 | Homo sapiens phorbol-12-myristate-13-acetate-induced protein 1 (PMAIP1), mRNA. | 1,9087 | 8,5844 | | 5,5527 | | 0,000287 | | | 0,00398 |
| PHLDA1 | Homo sapiens pleckstrin homology-like domain, family A, member 1 (PHLDA1), mRNA. | 1,9050 | 8,7796 | | 5,1406 | | 0,000506 | | | 0,00544 |
| AHNAK2 | Homo sapiens AHNAK nucleoprotein 2 (AHNAK2), mRNA. | 1,9030 | 9,1150 | | 6,2033 | | 0,000123 | | | 0,00247 |
| STX1A | Homo sapiens syntaxin 1A (brain) (STX1A), mRNA. | 1,9021 | 8,6632 | | 7,7946 | | 0,000019 | | | 0,00105 |
| IRF6 | Homo sapiens interferon regulatory factor 6 (IRF6), mRNA. | 1,8924 | 10,2496 | | 8,9620 | | 0,000006 | | | 0,00064 |
| S100A6 | Homo sapiens S100 calcium binding protein A6 (S100A6), mRNA. | 1,8798 | 11,3160 | | 4,5531 | | 0,001184 | | | 0,00911 |
| HPCAL1 | Homo sapiens hippocalcin-like 1 (HPCAL1), transcript variant 2, mRNA. | 1,8489 | 10,7077 | | 8,8212 | | 0,000007 | | | 0,00066 |
| HIST2H2AA3 | Homo sapiens histone cluster 2, H2aa3 (HIST2H2AA3), mRNA. | 1,8483 | 8,3087 | | 4,6066 | | 0,001093 | | | 0,00870 |
| CGB5 | Homo sapiens chorionic gonadotropin, beta polypeptide 5 (CGB5), mRNA. | 1,8475 | 8,9088 | | 5,5952 | | 0,000271 | | | 0,00386 |
| SMOX | Homo sapiens spermine oxidase (SMOX), transcript variant 2, mRNA. | 1,8443 | 8,5166 | | 7,1803 | | 0,000038 | | | 0,00141 |
| CSNK1E | Homo sapiens casein kinase 1, epsilon (CSNK1E), transcript variant 1, mRNA. | 1,8420 | 10,6245 | | 10,6896 | | 0,000001 | | | 0,00035 |
| IRAK2 | Homo sapiens interleukin-1 receptor-associated kinase 2 (IRAK2), mRNA. | 1,8395 | 8,5518 | | 7,7496 | | 0,000020 | | | 0,00108 |
| CEBPB | Homo sapiens CCAAT/enhancer binding protein (C/EBP), beta (CEBPB), mRNA. | 1,8367 | 12,1026 | | 9,4744 | | 0,000004 | | | 0,00052 |
| TGFA | Homo sapiens transforming growth factor, alpha (TGFA), mRNA. | 1,8345 | 9,5815 | | 6,0264 | | 0,000154 | | | 0,00282 |
| KLF9 | Homo sapiens Kruppel-like factor 9 (KLF9), mRNA. | 1,8288 | 8,5440 | | 6,1326 | | 0,000134 | | | 0,00262 |
| ECGF1 | Homo sapiens endothelial cell growth factor 1 (platelet-derived) (ECGF1), mRNA. | 1,8262 | 10,4093 | | 9,7228 | | 0,000003 | | | 0,00049 |
| TMEM158 | Homo sapiens transmembrane protein 158 (TMEM158), mRNA. | 1,8223 | 9,5246 | | 3,4304 | | 0,006874 | | | 0,03054 |
| RNU6-1 | Homo sapiens RNA, U6 small nuclear 1 (RNU6-1), small nuclear RNA. | 1,8211 | 10,2830 | | 6,0150 | | 0,000156 | | | 0,00284 |
| KLF11 | PREDICTED: Homo sapiens Kruppel-like factor 11 (KLF11), mRNA. | 1,8081 | 8,8663 | | 7,3044 | | 0,000033 | | | 0,00131 |
| BCAR3 | Homo sapiens breast cancer anti-estrogen resistance 3 (BCAR3), mRNA. | 1,8002 | 10,1635 | | 7,0432 | | 0,000045 | | | 0,00152 |
| GABARAPL1 | Homo sapiens GABA(A) receptor-associated protein like 1 (GABARAPL1), mRNA. | 1,7788 | 9,3636 | | 6,4344 | | 0,000092 | | | 0,00215 |
| FOSL1 | Homo sapiens FOS-like antigen 1 (FOSL1), mRNA. | 1,7735 | 10,3679 | | 7,6087 | | 0,000024 | | | 0,00115 |
| SPSB3 | Homo sapiens splA/ryanodine receptor domain and SOCS box containing 3 (SPSB3), mRNA. | 1,7683 | 8,6988 | | 7,1678 | | 0,000039 | | | 0,00141 |
| RIOK3 | Homo sapiens RIO kinase 3 (yeast) (RIOK3), transcript variant 1, mRNA. | 1,7678 | 9,8042 | | 7,3183 | | 0,000033 | | | 0,00130 |
| CAPRIN2 | Homo sapiens caprin family member 2 (CAPRIN2), transcript variant 1, mRNA. | 1,7577 | 9,7420 | | 6,6061 | | 0,000075 | | | 0,00193 |
| TUBB2A | Homo sapiens tubulin, beta 2A (TUBB2A), mRNA. | 1,7556 | 9,8472 | | 9,8485 | | 0,000003 | | | 0,00047 |
| HERPUD1 | Homo sapiens homocysteine-inducible, endoplasmic reticulum stress-inducible, ubiquitin-like domain member 1 (HERPUD1), transcript variant 3, mRNA. | 1,7471 | 9,7435 | | 9,5332 | | 0,000003 | | | 0,00051 |
| UPP1 | Homo sapiens uridine phosphorylase 1 (UPP1), transcript variant 1, mRNA. | 1,7458 | 11,0534 | | 10,2484 | | 0,000002 | | | 0,00041 |
| OAF | Homo sapiens OAF homolog (Drosophila) (OAF), mRNA. | 1,7335 | 9,6820 | | 5,1565 | | 0,000494 | | | 0,00538 |
| WIPI1 | Homo sapiens WD repeat domain, phosphoinositide interacting 1 (WIPI1), mRNA. | 1,7305 | 8,7435 | | 10,0455 | | 0,000002 | | | 0,00044 |
| NPC1 | Homo sapiens Niemann-Pick disease, type C1 (NPC1), mRNA. | 1,7288 | 9,2555 | | 9,4308 | | 0,000004 | | | 0,00052 |
| LOC441019 | PREDICTED: Homo sapiens hypothetical LOC441019 (LOC441019), mRNA. | 1,7286 | 11,1938 | | 3,1156 | | 0,011557 | | | 0,04425 |
| TNFAIP3 | Homo sapiens tumor necrosis factor, alpha-induced protein 3 (TNFAIP3), mRNA. | 1,7256 | 8,5022 | | 8,4980 | | 0,000009 | | | 0,00078 |
| RNU6-15 | Homo sapiens RNA, U6 small nuclear 15 (RNU6-15), small nuclear RNA. | 1,7131 | 10,3545 | | 5,9383 | | 0,000172 | | | 0,00301 |
| TSC22D1 | Homo sapiens TSC22 domain family, member 1 (TSC22D1), transcript variant 2, mRNA. | 1,7104 | 11,0991 | | 6,0294 | | 0,000153 | | | 0,00281 |
| HERPUD1 | Homo sapiens homocysteine-inducible, endoplasmic reticulum stress-inducible, ubiquitin-like domain member 1 (HERPUD1), transcript variant 3, mRNA. | 1,7091 | 9,8525 | | 9,6867 | | 0,000003 | | | 0,00049 |
| C12orf35 | Homo sapiens chromosome 12 open reading frame 35 (C12orf35), mRNA. | 1,7081 | 8,2899 | | 5,1971 | | 0,000467 | | | 0,00522 |
| LOC387841 | PREDICTED: Homo sapiens similar to ribosomal protein L13a, transcript variant 2 (LOC387841), mRNA. | 1,7054 | 9,8074 | | 4,7444 | | 0,000892 | | | 0,00769 |
| TGFA | Homo sapiens transforming growth factor, alpha (TGFA), transcript variant 2, mRNA. | 1,7050 | 9,1490 | | 6,1444 | | 0,000132 | | | 0,00259 |
| ITGA5 | Homo sapiens integrin, alpha 5 (fibronectin receptor, alpha polypeptide) (ITGA5), mRNA. | 1,7008 | 8,8605 | | 4,8917 | | 0,000721 | | | 0,00666 |
| SLC38A2 | Homo sapiens solute carrier family 38, member 2 (SLC38A2), mRNA. | 1,6993 | 10,0293 | | 9,1801 | | 0,000005 | | | 0,00058 |
| LARP6 | Homo sapiens La ribonucleoprotein domain family, member 6 (LARP6), transcript variant 1, mRNA. | 1,6957 | 9,0847 | | 9,5631 | | 0,000003 | | | 0,00051 |
| LRRC8A | Homo sapiens leucine rich repeat containing 8 family, member A (LRRC8A), mRNA. | 1,6935 | 9,3873 | | 10,1506 | | 0,000002 | | | 0,00042 |
| KLC3 | Homo sapiens kinesin light chain 3 (KLC3), transcript variant 1, mRNA. | 1,6846 | 8,8395 | | 6,7699 | | 0,000061 | | | 0,00178 |
| IL1A | Homo sapiens interleukin 1, alpha (IL1A), mRNA. | 1,6657 | 11,2883 | | 6,7262 | | 0,000065 | | | 0,00182 |
| FLNB | Homo sapiens filamin B, beta (actin binding protein 278) (FLNB), mRNA. | 1,6628 | 10,3910 | | 12,0824 | | 0,000000 | | | 0,00025 |
| CPXM1 | Homo sapiens carboxypeptidase X (M14 family), member 1 (CPXM1), mRNA. | 1,6597 | 8,8928 | | 5,9233 | | 0,000176 | | | 0,00305 |
| MAGT1 | Homo sapiens magnesium transporter 1 (MAGT1), mRNA. | 1,6539 | 9,3329 | | 8,4307 | | 0,000010 | | | 0,00080 |
| CD55 | Homo sapiens CD55 molecule, decay accelerating factor for complement (Cromer blood group) (CD55), mRNA. | 1,6528 | 8,1169 | | 9,4260 | | 0,000004 | | | 0,00052 |
| PLEK2 | Homo sapiens pleckstrin 2 (PLEK2), mRNA. | 1,6516 | 10,0090 | | 8,2405 | | 0,000012 | | | 0,00088 |
| LOC653506 | PREDICTED: Homo sapiens similar to meteorin, glial cell differentiation regulator-like (LOC653506), mRNA. | 1,6472 | 8,7033 | | 6,7450 | | 0,000063 | | | 0,00180 |
| NT5E | Homo sapiens 5'-nucleotidase, ecto (CD73) (NT5E), mRNA. | 1,6459 | 9,1609 | | 3,3693 | | 0,007598 | | | 0,03283 |
| NFKBIA | Homo sapiens nuclear factor of kappa light polypeptide gene enhancer in B-cells inhibitor, alpha (NFKBIA), mRNA. | 1,6445 | 10,8135 | | 9,1651 | | 0,000005 | | | 0,00059 |
| C5orf32 | Homo sapiens chromosome 5 open reading frame 32 (C5orf32), mRNA. | 1,6424 | 9,2392 | | 6,2197 | | 0,000120 | | | 0,00245 |
| ANTXR2 | Homo sapiens anthrax toxin receptor 2 (ANTXR2), mRNA. | 1,6361 | 8,8074 | | 5,6777 | | 0,000243 | | | 0,00362 |
| SELS | Homo sapiens selenoprotein S (SELS), transcript variant 2, mRNA. | 1,6313 | 9,4688 | | 10,9827 | | 0,000001 | | | 0,00031 |
| C17orf91 | Homo sapiens chromosome 17 open reading frame 91 (C17orf91), transcript variant 2, mRNA. | 1,6189 | 8,4174 | | 3,9325 | | 0,003067 | | | 0,01712 |
| NDEL1 | Homo sapiens nudE nuclear distribution gene E homolog (A. nidulans)-like 1 (NDEL1), transcript variant 2, mRNA. | 1,6170 | 10,3148 | | 6,3139 | | 0,000107 | | | 0,00230 |
| FOXO3 | Homo sapiens forkhead box O3 (FOXO3), transcript variant 2, mRNA. | 1,6155 | 10,2278 | | 8,5868 | | 0,000009 | | | 0,00074 |
| SMOX | Homo sapiens spermine oxidase (SMOX), transcript variant 4, mRNA. | 1,6137 | 8,4069 | | 6,9367 | | 0,000050 | | | 0,00162 |
| LRRFIP1 | Homo sapiens leucine rich repeat (in FLII) interacting protein 1 (LRRFIP1), mRNA. | 1,6047 | 10,1805 | | 7,9588 | | 0,000016 | | | 0,00097 |
| IRF9 | Homo sapiens interferon regulatory factor 9 (IRF9), mRNA. | 1,6032 | 8,9523 | | 5,2029 | | 0,000463 | | | 0,00520 |
| RAC2 | Homo sapiens ras-related C3 botulinum toxin substrate 2 (rho family, small GTP binding protein Rac2) (RAC2), mRNA. | 1,5940 | 11,6566 | | 6,0608 | | 0,000147 | | | 0,00276 |
| ITPR3 | Homo sapiens inositol 1,4,5-triphosphate receptor, type 3 (ITPR3), mRNA. | 1,5886 | 10,6160 | | 4,8287 | | 0,000789 | | | 0,00709 |
| ABCC3 | Homo sapiens ATP-binding cassette, sub-family C (CFTR/MRP), member 3 (ABCC3), mRNA. | 1,5873 | 9,1831 | | 6,0884 | | 0,000142 | | | 0,00271 |
| MCART1 | Homo sapiens mitochondrial carrier triple repeat 1 (MCART1), mRNA. | 1,5866 | 10,3956 | | 5,6979 | | 0,000236 | | | 0,00357 |
| DEDD2 | Homo sapiens death effector domain containing 2 (DEDD2), mRNA. | 1,5837 | 9,8540 | | 9,6288 | | 0,000003 | | | 0,00050 |
| FAM84B | Homo sapiens family with sequence similarity 84, member B (FAM84B), mRNA. | 1,5813 | 10,1890 | | 7,0961 | | 0,000042 | | | 0,00148 |
| SELS | Homo sapiens selenoprotein S (SELS), transcript variant 2, mRNA. | 1,5796 | 10,8747 | | 10,4855 | | 0,000001 | | | 0,00038 |
| SERTAD1 | Homo sapiens SERTA domain containing 1 (SERTAD1), mRNA. | 1,5680 | 9,5506 | | 5,7854 | | 0,000210 | | | 0,00335 |
| BHLHB2 | Homo sapiens basic helix-loop-helix domain containing, class B, 2 (BHLHB2), mRNA. | 1,5663 | 11,0063 | | 5,5855 | | 0,000274 | | | 0,00387 |
| GRB7 | Homo sapiens growth factor receptor-bound protein 7 (GRB7), transcript variant 1, mRNA. | 1,5562 | 8,1319 | | 8,1738 | | 0,000013 | | | 0,00090 |
| CGB1 | Homo sapiens chorionic gonadotropin, beta polypeptide 1 (CGB1), mRNA. | 1,5467 | 8,6809 | | 5,2903 | | 0,000410 | | | 0,00484 |
| CAPRIN2 | Homo sapiens caprin family member 2 (CAPRIN2), transcript variant 2, mRNA. | 1,5417 | 8,8682 | | 5,8699 | | 0,000188 | | | 0,00315 |
| TICAM1 | Homo sapiens toll-like receptor adaptor molecule 1 (TICAM1), transcript variant 2, mRNA. | 1,5414 | 8,7920 | | 6,2176 | | 0,000121 | | | 0,00245 |
| PLEC1 | Homo sapiens plectin 1, intermediate filament binding protein 500kDa (PLEC1), transcript variant 1, mRNA. | 1,5378 | 10,6405 | | 5,4163 | | 0,000345 | | | 0,00436 |
| LOC100130992 | PREDICTED: Homo sapiens similar to hCG2017625 (LOC100130992), mRNA. | 1,5371 | 7,8422 | | 5,5499 | | 0,000288 | | | 0,00398 |
| ISG15 | Homo sapiens ISG15 ubiquitin-like modifier (ISG15), mRNA. | 1,5370 | 9,0611 | | 4,6346 | | 0,001049 | | | 0,00847 |
| IRS2 | Homo sapiens insulin receptor substrate 2 (IRS2), mRNA. | 1,5254 | 9,0492 | | 5,6399 | | 0,000255 | | | 0,00373 |
| VGF | Homo sapiens VGF nerve growth factor inducible (VGF), mRNA. | 1,5190 | 8,5254 | | 6,8368 | | 0,000057 | | | 0,00171 |
| MT2A | Homo sapiens metallothionein 2A (MT2A), mRNA. | 1,5183 | 12,6639 | | 3,8252 | | 0,003635 | | | 0,01925 |
| TSC22D3 | Homo sapiens TSC22 domain family, member 3 (TSC22D3), transcript variant 1, mRNA. | 1,5182 | 9,2244 | | 6,8743 | | 0,000054 | | | 0,00168 |
| TMEM17 | Homo sapiens transmembrane protein 17 (TMEM17), mRNA. | 1,5172 | 10,8389 | | 5,2840 | | 0,000414 | | | 0,00487 |
| CSNK1E | Homo sapiens casein kinase 1, epsilon (CSNK1E), transcript variant 1, mRNA. | 1,5172 | 9,5699 | | 10,5517 | | 0,000001 | | | 0,00037 |
| PTAFR | Homo sapiens platelet-activating factor receptor (PTAFR), mRNA. | 1,5167 | 8,3379 | | 3,1478 | | 0,010955 | | | 0,04248 |
| GNA15 | Homo sapiens guanine nucleotide binding protein (G protein), alpha 15 (Gq class) (GNA15), mRNA. | 1,5096 | 9,6449 | | 7,8201 | | 0,000019 | | | 0,00104 |
| PDLIM7 | Homo sapiens PDZ and LIM domain 7 (enigma) (PDLIM7), transcript variant 4, mRNA. | 1,5074 | 9,3434 | | 5,2434 | | 0,000438 | | | 0,00500 |
| KLF4 | Homo sapiens Kruppel-like factor 4 (gut) (KLF4), mRNA. | 1,5074 | 8,5237 | | 6,5138 | | 0,000084 | | | 0,00204 |
| HMOX1 | Homo sapiens heme oxygenase (decycling) 1 (HMOX1), mRNA. | 1,5058 | 7,8583 | | 4,7181 | | 0,000928 | | | 0,00790 |
| CDCP1 | Homo sapiens CUB domain containing protein 1 (CDCP1), transcript variant 1, mRNA. | 1,5047 | 9,3142 | | 6,1073 | | 0,000139 | | | 0,00267 |
| IL1RN | Homo sapiens interleukin 1 receptor antagonist (IL1RN), transcript variant 4, mRNA. | 1,5025 | 8,0502 | | 3,0208 | | 0,013533 | | | 0,04990 |
| EPAS1 | Homo sapiens endothelial PAS domain protein 1 (EPAS1), mRNA. | 1,4907 | 9,7241 | | 6,3137 | | 0,000107 | | | 0,00230 |
| FKBP14 | Homo sapiens FK506 binding protein 14, 22 kDa (FKBP14), mRNA. | 1,4867 | 9,4597 | | 5,2543 | | 0,000431 | | | 0,00497 |
| SPIRE1 | Homo sapiens spire homolog 1 (Drosophila) (SPIRE1), transcript variant 2, mRNA. | 1,4860 | 10,4309 | | 8,3311 | | 0,000011 | | | 0,00084 |
| AHNAK | Homo sapiens AHNAK nucleoprotein (AHNAK), transcript variant 1, mRNA. | 1,4736 | 10,4686 | | 4,6839 | | 0,000975 | | | 0,00813 |
| OXSR1 | Homo sapiens oxidative-stress responsive 1 (OXSR1), mRNA. | 1,4722 | 10,0442 | | 7,3147 | | 0,000033 | | | 0,00130 |
| SLC20A1 | Homo sapiens solute carrier family 20 (phosphate transporter), member 1 (SLC20A1), mRNA. | 1,4679 | 11,4086 | | 10,0610 | | 0,000002 | | | 0,00043 |
| SPAG9 | Homo sapiens sperm associated antigen 9 (SPAG9), mRNA. | 1,4677 | 9,4232 | | 6,9478 | | 0,000050 | | | 0,00161 |
| YPEL5 | Homo sapiens yippee-like 5 (Drosophila) (YPEL5), mRNA. | 1,4650 | 9,7458 | | 7,5634 | | 0,000025 | | | 0,00118 |
| LOC143666 | PREDICTED: Homo sapiens hypothetical protein LOC143666 (LOC143666), mRNA. | 1,4636 | 8,2469 | | 9,8257 | | 0,000003 | | | 0,00048 |
| GSK3B | Homo sapiens glycogen synthase kinase 3 beta (GSK3B), mRNA. | 1,4620 | 9,0771 | | 5,2305 | | 0,000446 | | | 0,00507 |
| SNORD12C | Homo sapiens small nucleolar RNA, C/D box 12C (SNORD12C), small nucleolar RNA. | 1,4613 | 8,1338 | | 4,9807 | | 0,000634 | | | 0,00617 |
| LRRC8C | Homo sapiens leucine rich repeat containing 8 family, member C (LRRC8C), mRNA. | 1,4608 | 8,8199 | | 5,1378 | | 0,000508 | | | 0,00545 |
| DMC1 | Homo sapiens DMC1 dosage suppressor of mck1 homolog, meiosis-specific homologous recombination (yeast) (DMC1), mRNA. | 1,4554 | 9,9228 | | 5,6433 | | 0,000254 | | | 0,00372 |
| ASNS | Homo sapiens asparagine synthetase (ASNS), transcript variant 1, mRNA. | 1,4528 | 11,2867 | | 3,2970 | | 0,008557 | | | 0,03563 |
| ZFP36L1 | Homo sapiens zinc finger protein 36, C3H type-like 1 (ZFP36L1), mRNA. | 1,4515 | 9,6344 | | 7,4526 | | 0,000028 | | | 0,00123 |
| LIMA1 | Homo sapiens LIM domain and actin binding 1 (LIMA1), mRNA. | 1,4508 | 10,1871 | | 6,2601 | | 0,000114 | | | 0,00239 |
| KIAA1666 | PREDICTED: Homo sapiens KIAA1666 protein (KIAA1666), mRNA. | 1,4483 | 7,6982 | | 4,3619 | | 0,001578 | | | 0,01099 |
| ZFP36L2 | Homo sapiens zinc finger protein 36, C3H type-like 2 (ZFP36L2), mRNA. | 1,4468 | 8,8870 | | 4,9499 | | 0,000663 | | | 0,00633 |
| METRNL | PREDICTED: Homo sapiens meteorin, glial cell differentiation regulator-like (METRNL), mRNA. | 1,4466 | 8,5021 | | 6,4093 | | 0,000095 | | | 0,00217 |
| CLIC4 | Homo sapiens chloride intracellular channel 4 (CLIC4), nuclear gene encoding mitochondrial protein, mRNA. | 1,4462 | 9,5637 | | 8,2381 | | 0,000012 | | | 0,00088 |
| KIAA1949 | Homo sapiens KIAA1949 (KIAA1949), mRNA. | 1,4427 | 9,8850 | | 6,7641 | | 0,000062 | | | 0,00178 |
| LOC399900 | Homo sapiens hypothetical gene supported by AK093779 (LOC399900), mRNA. | 1,4330 | 11,4280 | | 7,3972 | | 0,000030 | | | 0,00126 |
| OASL | Homo sapiens 2'-5'-oligoadenylate synthetase-like (OASL), transcript variant 2, mRNA. | 1,4329 | 8,0484 | | 3,2793 | | 0,008811 | | | 0,03635 |
| C20orf111 | Homo sapiens chromosome 20 open reading frame 111 (C20orf111), mRNA. | 1,4302 | 10,5259 | | 7,6105 | | 0,000024 | | | 0,00115 |
| C10orf116 | Homo sapiens chromosome 10 open reading frame 116 (C10orf116), mRNA. | 1,4271 | 9,6789 | | 3,3423 | | 0,007942 | | | 0,03383 |
| C8orf45 | Homo sapiens chromosome 8 open reading frame 45 (C8orf45), mRNA. | 1,4183 | 10,0917 | | 6,1620 | | 0,000129 | | | 0,00255 |
| PNPT1 | Homo sapiens polyribonucleotide nucleotidyltransferase 1 (PNPT1), mRNA. | 1,4177 | 10,8640 | | 5,9416 | | 0,000172 | | | 0,00301 |
| PTPN12 | Homo sapiens protein tyrosine phosphatase, non-receptor type 12 (PTPN12), mRNA. | 1,4173 | 9,6519 | | 5,9431 | | 0,000171 | | | 0,00301 |
| PANX2 | Homo sapiens pannexin 2 (PANX2), mRNA. | 1,4158 | 8,8349 | | 4,0964 | | 0,002373 | | | 0,01447 |
| LOC728809 | PREDICTED: Homo sapiens hypothetical LOC728809 (LOC728809), mRNA. | 1,4126 | 10,5498 | | 5,0815 | | 0,000550 | | | 0,00572 |
| CHIC2 | Homo sapiens cysteine-rich hydrophobic domain 2 (CHIC2), mRNA. | 1,4100 | 9,6401 | | 7,0248 | | 0,000046 | | | 0,00153 |
| ATP9A | Homo sapiens ATPase, class II, type 9A (ATP9A), mRNA. | 1,4082 | 10,0794 | | 7,9381 | | 0,000017 | | | 0,00098 |
| CLK1 | Homo sapiens CDC-like kinase 1 (CLK1), mRNA. | 1,4051 | 9,2010 | | 4,9557 | | 0,000657 | | | 0,00630 |
| SH3PXD2A | Homo sapiens SH3 and PX domains 2A (SH3PXD2A), mRNA. | 1,3958 | 10,3042 | | 4,2165 | | 0,001971 | | | 0,01275 |
| NLRP8 | Homo sapiens NLR family, pyrin domain containing 8 (NLRP8), mRNA. | 1,3912 | 8,8883 | | 5,3978 | | 0,000354 | | | 0,00441 |
| LOC100133516 | PREDICTED: Homo sapiens hypothetical protein LOC100133516 (LOC100133516), mRNA. | 1,3893 | 8,6611 | | 7,4202 | | 0,000029 | | | 0,00125 |
| TMC6 | Homo sapiens transmembrane channel-like 6 (TMC6), mRNA. | 1,3888 | 8,4737 | | 6,0367 | | 0,000152 | | | 0,00280 |
| LOC255167 | Homo sapiens hypothetical LOC255167 (LOC255167), non-coding RNA. | 1,3879 | 9,3624 | | 4,9992 | | 0,000618 | | | 0,00611 |
| SPAG9 | Homo sapiens sperm associated antigen 9 (SPAG9), mRNA. | 1,3873 | 9,1680 | | 5,4053 | | 0,000350 | | | 0,00439 |
| FLJ40504 | Homo sapiens hypothetical protein FLJ40504 (FLJ40504), mRNA. | 1,3806 | 9,5329 | | 4,6532 | | 0,001021 | | | 0,00834 |
| CD68 | Homo sapiens CD68 antigen (CD68), mRNA. | 1,3804 | 10,1827 | | 3,2510 | | 0,009232 | | | 0,03750 |
| CDCP1 | Homo sapiens CUB domain containing protein 1 (CDCP1), transcript variant 2, mRNA. | 1,3804 | 8,2879 | | 4,5542 | | 0,001182 | | | 0,00911 |
| ARL16 | Homo sapiens ADP-ribosylation factor-like 16 (ARL16), mRNA. | 1,3739 | 10,3355 | | 5,8333 | | 0,000198 | | | 0,00324 |
| AMTN | Homo sapiens amelotin (AMTN), mRNA. | 1,3737 | 8,4087 | | 6,0801 | | 0,000144 | | | 0,00272 |
| KLF13 | Homo sapiens Kruppel-like factor 13 (KLF13), mRNA. | 1,3708 | 9,2046 | | 5,8958 | | 0,000182 | | | 0,00309 |
| LOC100130168 | PREDICTED: Homo sapiens hypothetical protein LOC100130168 (LOC100130168), mRNA. | 1,3685 | 10,7291 | | 5,7165 | | 0,000230 | | | 0,00352 |
| SH3KBP1 | Homo sapiens SH3-domain kinase binding protein 1 (SH3KBP1), transcript variant 1, mRNA. | 1,3682 | 9,8366 | | 6,2244 | | 0,000120 | | | 0,00245 |
| IL1RL1 | Homo sapiens interleukin 1 receptor-like 1 (IL1RL1), transcript variant 2, mRNA. | 1,3673 | 7,7433 | | 3,7714 | | 0,003961 | | | 0,02044 |
| BIRC3 | Homo sapiens baculoviral IAP repeat-containing 3 (BIRC3), transcript variant 2, mRNA. | 1,3608 | 8,1433 | | 5,4245 | | 0,000341 | | | 0,00434 |
| ZBTB43 | Homo sapiens zinc finger and BTB domain containing 43 (ZBTB43), mRNA. | 1,3584 | 8,2221 | | 4,9049 | | 0,000707 | | | 0,00660 |
| ZNF682 | Homo sapiens zinc finger protein 682 (ZNF682), transcript variant 1, mRNA. | 1,3562 | 9,4694 | | 4,7388 | | 0,000900 | | | 0,00772 |
| GFPT1 | Homo sapiens glutamine-fructose-6-phosphate transaminase 1 (GFPT1), mRNA. | 1,3557 | 9,0827 | | 13,9897 | | 0,000000 | | | 0,00012 |
| S100A16 | Homo sapiens S100 calcium binding protein A16 (S100A16), mRNA. | 1,3551 | 11,2390 | | 5,4183 | | 0,000344 | | | 0,00435 |
| ANG | Homo sapiens angiogenin, ribonuclease, RNase A family, 5 (ANG), transcript variant 2, mRNA. | 1,3539 | 8,4875 | | 5,4815 | | 0,000316 | | | 0,00420 |
| TSC22D1 | Homo sapiens TSC22 domain family, member 1 (TSC22D1), transcript variant 1, mRNA. | 1,3501 | 8,4585 | | 4,4620 | | 0,001357 | | | 0,00997 |
| ASS1 | Homo sapiens argininosuccinate synthetase 1 (ASS1), transcript variant 1, mRNA. | 1,3481 | 8,6999 | | 5,0539 | | 0,000571 | | | 0,00584 |
| MMP3 | Homo sapiens matrix metallopeptidase 3 (stromelysin 1, progelatinase) (MMP3), mRNA. | 1,3475 | 7,6619 | | 3,7813 | | 0,003899 | | | 0,02021 |
| RNU4-1 | Homo sapiens RNA, U4 small nuclear 1 (RNU4-1), small nuclear RNA. | 1,3473 | 7,6418 | | 3,6600 | | 0,004736 | | | 0,02319 |
| FAM129B | Homo sapiens family with sequence similarity 129, member B (FAM129B), transcript variant 1, mRNA. | 1,3466 | 11,6282 | | 5,4502 | | 0,000329 | | | 0,00429 |
| FOXO3 | Homo sapiens forkhead box O3 (FOXO3), transcript variant 2, mRNA. | 1,3463 | 9,2080 | | 6,0276 | | 0,000154 | | | 0,00281 |
| CLCF1 | Homo sapiens cardiotrophin-like cytokine factor 1 (CLCF1), transcript variant 1, mRNA. | 1,3458 | 8,4703 | | 5,8526 | | 0,000193 | | | 0,00320 |
| SNORD46 | Homo sapiens small nucleolar RNA, C/D box 46 (SNORD46), small nucleolar RNA. | 1,3452 | 7,7333 | | 4,6891 | | 0,000968 | | | 0,00809 |
| NP | Homo sapiens nucleoside phosphorylase (NP), mRNA. | 1,3443 | 9,6346 | | 4,8981 | | 0,000714 | | | 0,00663 |
| NIPA1 | Homo sapiens non imprinted in Prader-Willi/Angelman syndrome 1 (NIPA1), mRNA. | 1,3403 | 8,6022 | | 5,4095 | | 0,000348 | | | 0,00437 |
| HNRPDL | Homo sapiens heterogeneous nuclear ribonucleoprotein D-like (HNRPDL), transcript variant 3, transcribed RNA. | 1,3402 | 9,1472 | | 8,0492 | | 0,000015 | | | 0,00095 |
| SLC16A3 | Homo sapiens solute carrier family 16, member 3 (monocarboxylic acid transporter 4) (SLC16A3), transcript variant 2, mRNA. | 1,3400 | 8,7470 | | 6,7328 | | 0,000064 | | | 0,00181 |
| DUSP6 | Homo sapiens dual specificity phosphatase 6 (DUSP6), transcript variant 2, mRNA. | 1,3390 | 9,8570 | | 4,0504 | | 0,002550 | | | 0,01524 |
| UGCG | Homo sapiens UDP-glucose ceramide glucosyltransferase (UGCG), mRNA. | 1,3380 | 8,7964 | | 6,4458 | | 0,000091 | | | 0,00214 |
| RNU4ATAC | Homo sapiens RNA, U4atac small nuclear (U12-dependent splicing) (RNU4ATAC), small nuclear RNA. | 1,3374 | 7,7656 | | 5,5188 | | 0,000300 | | | 0,00408 |
| ATF4 | Homo sapiens activating transcription factor 4 (tax-responsive enhancer element B67) (ATF4), transcript variant 2, mRNA. | 1,3355 | 9,1581 | | 7,1167 | | 0,000041 | | | 0,00146 |
| RIOK3 | Homo sapiens RIO kinase 3 (yeast) (RIOK3), mRNA. | 1,3329 | 9,9222 | | 6,4510 | | 0,000090 | | | 0,00214 |
| LOC90586 | Homo sapiens AOC3 pseudogene (LOC90586), non-coding RNA. | 1,3309 | 9,2142 | | 4,4799 | | 0,001321 | | | 0,00980 |
| MAP1LC3B | Homo sapiens microtubule-associated protein 1 light chain 3 beta (MAP1LC3B), mRNA. | 1,3294 | 9,0212 | | 9,5696 | | 0,000003 | | | 0,00051 |
| PPP2R2C | Homo sapiens protein phosphatase 2 (formerly 2A), regulatory subunit B, gamma isoform (PPP2R2C), transcript variant 2, mRNA. | 1,3279 | 8,7181 | | 5,3606 | | 0,000372 | | | 0,00454 |
| ZFAND2A | Homo sapiens zinc finger, AN1-type domain 2A (ZFAND2A), mRNA. | 1,3260 | 9,2534 | | 10,6698 | | 0,000001 | | | 0,00035 |
| POFUT1 | Homo sapiens protein O-fucosyltransferase 1 (POFUT1), transcript variant 1, mRNA. | 1,3237 | 10,9551 | | 5,9625 | | 0,000167 | | | 0,00295 |
| LOC730313 | PREDICTED: Homo sapiens hypothetical LOC730313 (LOC730313), mRNA. | 1,3236 | 10,9684 | | 5,4203 | | 0,000343 | | | 0,00435 |
| MCL1 | Homo sapiens myeloid cell leukemia sequence 1 (BCL2-related) (MCL1), transcript variant 1, mRNA. | 1,3230 | 9,7215 | | 7,1680 | | 0,000039 | | | 0,00141 |
| DNAJB2 | Homo sapiens DnaJ (Hsp40) homolog, subfamily B, member 2 (DNAJB2), transcript variant 2, mRNA. | 1,3217 | 10,6562 | | 5,2826 | | 0,000415 | | | 0,00487 |
| PRIC285 | Homo sapiens peroxisomal proliferator-activated receptor A interacting complex 285 (PRIC285), transcript variant 2, mRNA. | 1,3209 | 8,4430 | | 7,8024 | | 0,000019 | | | 0,00105 |
| SPRR1A | Homo sapiens small proline-rich protein 1A (SPRR1A), mRNA. | 1,3208 | 8,5939 | | 4,2965 | | 0,001744 | | | 0,01176 |
| FOXQ1 | Homo sapiens forkhead box Q1 (FOXQ1), mRNA. | 1,3207 | 9,6821 | | 3,5832 | | 0,005360 | | | 0,02536 |
| KRT16 | Homo sapiens keratin 16 (focal non-epidermolytic palmoplantar keratoderma) (KRT16), mRNA. | 1,3205 | 8,9764 | | 3,4362 | | 0,006809 | | | 0,03033 |
| LARP1B | Homo sapiens La ribonucleoprotein domain family, member 1B (LARP1B), transcript variant 3, mRNA. | 1,3198 | 9,1282 | | 10,3098 | | 0,000002 | | | 0,00041 |
| MALL | Homo sapiens mal, T-cell differentiation protein-like (MALL), mRNA. | 1,3197 | 10,3504 | | 4,2860 | | 0,001772 | | | 0,01186 |
| XRCC2 | Homo sapiens X-ray repair complementing defective repair in Chinese hamster cells 2 (XRCC2), mRNA. | 1,3173 | 9,0707 | | 5,6329 | | 0,000257 | | | 0,00375 |
| SPHK1 | Homo sapiens sphingosine kinase 1 (SPHK1), transcript variant 1, mRNA. | 1,3164 | 8,6296 | | 5,4525 | | 0,000328 | | | 0,00428 |
| ZNF486 | PREDICTED: Homo sapiens zinc finger protein 486 (ZNF486), mRNA. | 1,3155 | 10,1461 | | 5,0585 | | 0,000568 | | | 0,00582 |
| OPLAH | Homo sapiens 5-oxoprolinase (ATP-hydrolysing) (OPLAH), mRNA. | 1,3144 | 8,9202 | | 8,4897 | | 0,000009 | | | 0,00078 |
| GADD45A | Homo sapiens growth arrest and DNA-damage-inducible, alpha (GADD45A), mRNA. | 1,3130 | 9,6129 | | 5,6544 | | 0,000250 | | | 0,00369 |
| GADD45A | Homo sapiens growth arrest and DNA-damage-inducible, alpha (GADD45A), mRNA. | 1,3115 | 9,8964 | | 6,2168 | | 0,000121 | | | 0,00245 |
| KIAA1751 | Homo sapiens KIAA1751 (KIAA1751), mRNA. | 1,3075 | 9,0478 | | 5,6191 | | 0,000262 | | | 0,00380 |
| SDHALP1 | Homo sapiens succinate dehydrogenase complex, subunit A, flavoprotein pseudogene 1 (SDHALP1) on chromosome 3. | 1,3070 | 8,4646 | | 5,0505 | | 0,000574 | | | 0,00585 |
| C8orf37 | Homo sapiens chromosome 8 open reading frame 37 (C8orf37), mRNA. | 1,3068 | 10,5108 | | 6,5425 | | 0,000081 | | | 0,00201 |
| TGFB1I1 | Homo sapiens transforming growth factor beta 1 induced transcript 1 (TGFB1I1), transcript variant 2, mRNA. | 1,3025 | 8,3460 | | 8,8406 | | 0,000007 | | | 0,00066 |
| SHROOM4 | Homo sapiens shroom family member 4 (SHROOM4), mRNA. | 1,3021 | 8,9623 | | 6,5170 | | 0,000083 | | | 0,00203 |
| RHOC | Homo sapiens ras homolog gene family, member C (RHOC), transcript variant 2, mRNA. | 1,3018 | 10,1223 | | 5,3195 | | 0,000394 | | | 0,00472 |
| KIAA0913 | Homo sapiens KIAA0913 (KIAA0913), mRNA. | 1,3003 | 8,7502 | | 5,0303 | | 0,000591 | | | 0,00595 |
| SNORA80 | Homo sapiens small nucleolar RNA, H/ACA box 80 (SNORA80), small nucleolar RNA. | 1,2988 | 7,8292 | | 3,8710 | | 0,003380 | | | 0,01831 |
| PDE4C | Homo sapiens phosphodiesterase 4C, cAMP-specific (phosphodiesterase E1 dunce homolog, Drosophila) (PDE4C), mRNA. | 1,2969 | 11,2812 | | 4,3436 | | 0,001623 | | | 0,01119 |
| DUSP10 | Homo sapiens dual specificity phosphatase 10 (DUSP10), transcript variant 3, mRNA. | 1,2951 | 8,3157 | | 7,0742 | | 0,000043 | | | 0,00149 |
| C9orf80 | Homo sapiens chromosome 9 open reading frame 80 (C9orf80), mRNA. | 1,2883 | 10,1557 | | 5,3703 | | 0,000367 | | | 0,00450 |
| PTGS2 | Homo sapiens prostaglandin-endoperoxide synthase 2 (prostaglandin G/H synthase and cyclooxygenase) (PTGS2), mRNA. | 1,2875 | 7,9718 | | 3,2785 | | 0,008822 | | | 0,03638 |
| ZNF165 | Homo sapiens zinc finger protein 165 (ZNF165), mRNA. | 1,2871 | 8,6088 | | 6,8870 | | 0,000053 | | | 0,00168 |
| ZNF483 | Homo sapiens zinc finger protein 483 (ZNF483), transcript variant 2, mRNA. | 1,2870 | 8,5456 | | 5,1267 | | 0,000516 | | | 0,00550 |
| TIPARP | Homo sapiens TCDD-inducible poly(ADP-ribose) polymerase (TIPARP), mRNA. | 1,2868 | 9,9777 | | 4,0583 | | 0,002518 | | | 0,01510 |
| LOC401098 | PREDICTED: Homo sapiens misc_RNA (LOC401098), miscRNA. | 1,2860 | 8,4091 | | 5,5504 | | 0,000288 | | | 0,00398 |
| HIST2H2AA4 | Homo sapiens histone cluster 2, H2aa4 (HIST2H2AA4), mRNA. | 1,2782 | 7,9577 | | 3,8107 | | 0,003720 | | | 0,01956 |
| LOC645452 | PREDICTED: Homo sapiens similar to hCG1782414 (LOC645452), mRNA. | 1,2782 | 10,4999 | | 4,8091 | | 0,000812 | | | 0,00723 |
| KIAA1539 | Homo sapiens KIAA1539 (KIAA1539), mRNA. | 1,2771 | 8,3629 | | 6,1296 | | 0,000135 | | | 0,00262 |
| ID2 | Homo sapiens inhibitor of DNA binding 2, dominant negative helix-loop-helix protein (ID2), mRNA. | 1,2763 | 7,6885 | | 5,4670 | | 0,000322 | | | 0,00423 |
| UBAP1 | Homo sapiens ubiquitin associated protein 1 (UBAP1), mRNA. | 1,2690 | 10,1780 | | 8,9122 | | 0,000006 | | | 0,00064 |
| ARFGAP3 | Homo sapiens ADP-ribosylation factor GTPase activating protein 3 (ARFGAP3), mRNA. | 1,2678 | 9,2224 | | 9,6710 | | 0,000003 | | | 0,00049 |
| LOC645452 | PREDICTED: Homo sapiens similar to hCG1782414 (LOC645452), mRNA. | 1,2676 | 9,3406 | | 4,8777 | | 0,000735 | | | 0,00675 |
| PPA2 | Homo sapiens pyrophosphatase (inorganic) 2 (PPA2), nuclear gene encoding mitochondrial protein, transcript variant 2, mRNA. | 1,2673 | 9,5880 | | 4,8285 | | 0,000790 | | | 0,00709 |
| EIF1 | Homo sapiens eukaryotic translation initiation factor 1 (EIF1), mRNA. | 1,2658 | 12,1488 | | 10,6673 | | 0,000001 | | | 0,00035 |
| C3orf52 | Homo sapiens chromosome 3 open reading frame 52 (C3orf52), mRNA. | 1,2655 | 8,4664 | | 5,6255 | | 0,000260 | | | 0,00378 |
| KIAA0363 | PREDICTED: Homo sapiens KIAA0363 protein (KIAA0363), mRNA. | 1,2649 | 8,0311 | | 9,8693 | | 0,000003 | | | 0,00047 |
| LOC644132 | PREDICTED: Homo sapiens misc_RNA (LOC644132), miscRNA. | 1,2630 | 8,8323 | | 6,5156 | | 0,000083 | | | 0,00204 |
| MOBKL2C | Homo sapiens MOB1, Mps One Binder kinase activator-like 2C (yeast) (MOBKL2C), transcript variant 1, mRNA. | 1,2622 | 8,8043 | | 4,7278 | | 0,000914 | | | 0,00782 |
| YOD1 | Homo sapiens YOD1 OTU deubiquinating enzyme 1 homolog (S. cerevisiae) (YOD1), mRNA. | 1,2586 | 8,8545 | | 4,2439 | | 0,001890 | | | 0,01237 |
| ASNS | Homo sapiens asparagine synthetase (ASNS), transcript variant 1, mRNA. | 1,2578 | 9,4799 | | 3,6085 | | 0,005145 | | | 0,02461 |
| RHOC | Homo sapiens ras homolog gene family, member C (RHOC), transcript variant 1, mRNA. | 1,2576 | 11,8562 | | 7,9305 | | 0,000017 | | | 0,00098 |
| C13orf15 | Homo sapiens chromosome 13 open reading frame 15 (C13orf15), mRNA. | 1,2570 | 8,5637 | | 6,9597 | | 0,000049 | | | 0,00159 |
| DUXAP3 | Homo sapiens double homeobox A pseudogene 3 (DUXAP3) on chromosome 10. | 1,2542 | 10,1690 | | 4,7542 | | 0,000880 | | | 0,00762 |
| PTPN12 | Homo sapiens protein tyrosine phosphatase, non-receptor type 12 (PTPN12), mRNA. | 1,2537 | 9,5351 | | 5,7944 | | 0,000208 | | | 0,00333 |
| PRKCD | Homo sapiens protein kinase C, delta (PRKCD), transcript variant 1, mRNA. | 1,2529 | 8,9558 | | 7,3853 | | 0,000030 | | | 0,00127 |
| KCNK6 | Homo sapiens potassium channel, subfamily K, member 6 (KCNK6), mRNA. | 1,2514 | 8,8688 | | 9,7000 | | 0,000003 | | | 0,00049 |
| ALPP | Homo sapiens alkaline phosphatase, placental (Regan isozyme) (ALPP), mRNA. | 1,2509 | 9,5113 | | 5,1046 | | 0,000532 | | | 0,00561 |
| ZNF430 | Homo sapiens zinc finger protein 430 (ZNF430), mRNA. | 1,2500 | 11,2019 | | 5,6227 | | 0,000261 | | | 0,00379 |
| KRT18P13 | PREDICTED: Homo sapiens keratin 18 pseudogene 13 (KRT18P13), mRNA. | 1,2480 | 8,9101 | | 5,6707 | | 0,000245 | | | 0,00364 |
| AKAP12 | Homo sapiens A kinase (PRKA) anchor protein (gravin) 12 (AKAP12), transcript variant 1, mRNA. | 1,2480 | 8,0359 | | 5,5500 | | 0,000288 | | | 0,00398 |
| ATP2B4 | Homo sapiens ATPase, Ca++ transporting, plasma membrane 4 (ATP2B4), transcript variant 2, mRNA. | 1,2442 | 8,7507 | | 6,6656 | | 0,000070 | | | 0,00187 |
| ZNF14 | Homo sapiens zinc finger protein 14 (ZNF14), mRNA. | 1,2437 | 10,3899 | | 5,7992 | | 0,000207 | | | 0,00333 |
| LOC100132391 | PREDICTED: Homo sapiens hypothetical protein LOC100132391 (LOC100132391), mRNA. | 1,2432 | 10,0058 | | 5,9914 | | 0,000161 | | | 0,00290 |
| LOC389517 | Homo sapiens Williams Beuren syndrome chromosome region 19 pseudogene (LOC389517) on chromosome 7. | 1,2421 | 11,1293 | | 4,5878 | | 0,001124 | | | 0,00883 |
| ZNF549 | Homo sapiens zinc finger protein 549 (ZNF549), mRNA. | 1,2417 | 11,0362 | | 5,8378 | | 0,000196 | | | 0,00322 |
| JUND | Homo sapiens jun D proto-oncogene (JUND), mRNA. | 1,2413 | 12,2617 | | 7,0398 | | 0,000045 | | | 0,00152 |
| LOC646463 | PREDICTED: Homo sapiens similar to Ubiquitin-conjugating enzyme E2 H (Ubiquitin-protein ligase H) (Ubiquitin carrier protein H) (UBCH2) (E2-20K) (LOC646463), mRNA. | 1,2395 | 8,5655 | | 7,0450 | | 0,000044 | | | 0,00152 |
| CITED4 | Homo sapiens Cbp/p300-interacting transactivator, with Glu/Asp-rich carboxy-terminal domain, 4 (CITED4), mRNA. | 1,2395 | 9,9969 | | 6,4042 | | 0,000096 | | | 0,00217 |
| LRRC37B2 | Homo sapiens leucine rich repeat containing 37, member B2 (LRRC37B2), non-coding RNA. | 1,2387 | 9,4241 | | 5,1763 | | 0,000481 | | | 0,00531 |
| ZNF69 | Homo sapiens zinc finger protein 69 (ZNF69), mRNA. | 1,2380 | 9,8423 | | 6,2107 | | 0,000122 | | | 0,00246 |
| CD68 | Homo sapiens CD68 molecule (CD68), transcript variant 1, mRNA. | 1,2357 | 9,2924 | | 4,2711 | | 0,001812 | | | 0,01203 |
| XBP1 | Homo sapiens X-box binding protein 1 (XBP1), transcript variant 1, mRNA. | 1,2355 | 10,8551 | | 7,8887 | | 0,000017 | | | 0,00101 |
| C14orf153 | Homo sapiens chromosome 14 open reading frame 153 (C14orf153), mRNA. | 1,2322 | 9,7921 | | 6,5364 | | 0,000081 | | | 0,00201 |
| CLDN12 | Homo sapiens claudin 12 (CLDN12), mRNA. | 1,2316 | 8,9946 | | 4,4420 | | 0,001398 | | | 0,01016 |
| LOC100128288 | Homo sapiens hypothetical protein LOC100128288 (LOC100128288), non-coding RNA. | 1,2310 | 9,8773 | | 5,1710 | | 0,000484 | | | 0,00533 |
| DENND2C | Homo sapiens DENN/MADD domain containing 2C (DENND2C), mRNA. | 1,2310 | 8,3480 | | 4,4202 | | 0,001445 | | | 0,01039 |
| ZNF394 | Homo sapiens zinc finger protein 394 (ZNF394), mRNA. | 1,2288 | 10,3577 | | 6,5024 | | 0,000085 | | | 0,00205 |
| LMOD3 | Homo sapiens leiomodin 3 (fetal) (LMOD3), mRNA. | 1,2281 | 8,5828 | | 5,6785 | | 0,000242 | | | 0,00362 |
| TSC22D3 | Homo sapiens TSC22 domain family, member 3 (TSC22D3), transcript variant 2, mRNA. | 1,2273 | 8,4406 | | 7,4641 | | 0,000028 | | | 0,00122 |
| TNIP1 | Homo sapiens TNFAIP3 interacting protein 1 (TNIP1), mRNA. | 1,2269 | 9,2692 | | 5,0139 | | 0,000605 | | | 0,00604 |
| EFNA1 | Homo sapiens ephrin-A1 (EFNA1), transcript variant 1, mRNA. | 1,2247 | 9,4784 | | 5,2999 | | 0,000405 | | | 0,00480 |
| SPRY4 | Homo sapiens sprouty homolog 4 (Drosophila) (SPRY4), mRNA. | 1,2238 | 8,2338 | | 4,6085 | | 0,001090 | | | 0,00869 |
| RALGDS | Homo sapiens ral guanine nucleotide dissociation stimulator (RALGDS), transcript variant 1, mRNA. | 1,2231 | 9,5702 | | 6,1160 | | 0,000137 | | | 0,00266 |
| GPSM1 | PREDICTED: Homo sapiens G-protein signalling modulator 1 (AGS3-like, C. elegans) (GPSM1), mRNA. | 1,2227 | 8,0977 | | 7,8602 | | 0,000018 | | | 0,00102 |
| DAPP1 | Homo sapiens dual adaptor of phosphotyrosine and 3-phosphoinositides (DAPP1), mRNA. | 1,2226 | 10,2606 | | 7,6479 | | 0,000023 | | | 0,00113 |
| MFSD10 | Homo sapiens major facilitator superfamily domain containing 10 (MFSD10), mRNA. | 1,2208 | 11,0001 | | 7,7005 | | 0,000021 | | | 0,00111 |
| PI4KAP2 | Homo sapiens phosphatidylinositol 4-kinase, catalytic, alpha polypeptide pseudogene 2 (PI4KAP2), mRNA. | 1,2206 | 9,2163 | | 8,1851 | | 0,000013 | | | 0,00089 |
| LOC100132585 | PREDICTED: Homo sapiens hypothetical protein LOC100132585 (LOC100132585), mRNA. | 1,2201 | 8,5004 | | 4,7220 | | 0,000922 | | | 0,00787 |
| LOC646723 | PREDICTED: Homo sapiens similar to Keratin, type I cytoskeletal 18 (Cytokeratin-18) (CK-18) (Keratin-18) (K18) (LOC646723), mRNA. | 1,2189 | 11,4257 | | 4,0800 | | 0,002435 | | | 0,01473 |
| CATSPER2 | Homo sapiens cation channel, sperm associated 2 (CATSPER2), transcript variant 4, mRNA. | 1,2179 | 9,4351 | | 4,8423 | | 0,000774 | | | 0,00700 |
| BAMBI | Homo sapiens BMP and activin membrane-bound inhibitor homolog (Xenopus laevis) (BAMBI), mRNA. | 1,2178 | 8,4989 | | 6,8084 | | 0,000059 | | | 0,00174 |
| ITGB4 | Homo sapiens integrin, beta 4 (ITGB4), transcript variant 2, mRNA. | 1,2172 | 11,2281 | | 7,5811 | | 0,000024 | | | 0,00117 |
| RRAS | Homo sapiens related RAS viral (r-ras) oncogene homolog (RRAS), mRNA. | 1,2154 | 10,0230 | | 5,4390 | | 0,000334 | | | 0,00431 |
| LOC100128505 | PREDICTED: Homo sapiens similar to hCG2021201 (LOC100128505), mRNA. | 1,2139 | 11,9827 | | 5,7653 | | 0,000216 | | | 0,00340 |
| ATP2B4 | Homo sapiens ATPase, Ca++ transporting, plasma membrane 4 (ATP2B4), transcript variant 1, mRNA. | 1,2138 | 8,7854 | | 6,2182 | | 0,000121 | | | 0,00245 |
| LCP1 | Homo sapiens lymphocyte cytosolic protein 1 (L-plastin) (LCP1), mRNA. | 1,2136 | 9,9341 | | 9,9564 | | 0,000002 | | | 0,00045 |
| AIRE | Homo sapiens autoimmune regulator (autoimmune polyendocrinopathy candidiasis ectodermal dystrophy) (AIRE), transcript variant AIRE-1, mRNA. | 1,2118 | 10,3426 | | 5,4646 | | 0,000323 | | | 0,00424 |
| ITGB4 | Homo sapiens integrin, beta 4 (ITGB4), transcript variant 3, mRNA. | 1,2115 | 10,2650 | | 7,7766 | | 0,000020 | | | 0,00107 |
| LOC728903 | PREDICTED: Homo sapiens hypothetical LOC728903, transcript variant 1 (LOC728903), mRNA. | 1,2112 | 9,1541 | | 5,3541 | | 0,000376 | | | 0,00457 |
| BLZF1 | Homo sapiens basic leucine zipper nuclear factor 1 (BLZF1), mRNA. | 1,2109 | 9,3263 | | 5,0348 | | 0,000587 | | | 0,00593 |
| FAM175A | Homo sapiens family with sequence similarity 175, member A (FAM175A), mRNA. | 1,2104 | 9,1543 | | 4,9347 | | 0,000677 | | | 0,00640 |
| STK17B | Homo sapiens serine/threonine kinase 17b (STK17B), mRNA. | 1,2101 | 8,3332 | | 6,3203 | | 0,000106 | | | 0,00229 |
| LOC100190938 | Homo sapiens hypothetical LOC100190938 (LOC100190938), transcript variant 2, non-coding RNA. | 1,2075 | 11,3488 | | 4,4357 | | 0,001412 | | | 0,01022 |
| NUMB | Homo sapiens numb homolog (Drosophila) (NUMB), transcript variant 3, mRNA. | 1,2074 | 9,5942 | | 6,3426 | | 0,000103 | | | 0,00227 |
| GFPT1 | Homo sapiens glutamine-fructose-6-phosphate transaminase 1 (GFPT1), mRNA. | 1,2071 | 9,3057 | | 14,3090 | | 0,000000 | | | 0,00012 |
| WSB1 | Homo sapiens WD repeat and SOCS box-containing 1 (WSB1), transcript variant 2, mRNA. | 1,2042 | 9,8670 | | 5,9658 | | 0,000166 | | | 0,00295 |
| RNF44 | Homo sapiens ring finger protein 44 (RNF44), mRNA. | 1,2039 | 8,5722 | | 9,6513 | | 0,000003 | | | 0,00049 |
| ACSS2 | Homo sapiens acyl-CoA synthetase short-chain family member 2 (ACSS2), transcript variant 1, mRNA. | 1,2038 | 9,3807 | | 5,0358 | | 0,000586 | | | 0,00593 |
| SQSTM1 | Homo sapiens sequestosome 1 (SQSTM1), mRNA. | 1,2036 | 12,8427 | | 8,6622 | | 0,000008 | | | 0,00073 |
| LOC643031 | PREDICTED: Homo sapiens similar to NADH dehydrogenase subunit 5 (LOC643031), mRNA. | 1,2029 | 11,4645 | | 5,1444 | | 0,000503 | | | 0,00542 |
| USP36 | Homo sapiens ubiquitin specific peptidase 36 (USP36), mRNA. | 1,1981 | 8,1353 | | 5,0019 | | 0,000615 | | | 0,00610 |
| PIGA | Homo sapiens phosphatidylinositol glycan anchor biosynthesis, class A (PIGA), transcript variant 3, mRNA. | 1,1975 | 8,6022 | | 8,9697 | | 0,000006 | | | 0,00064 |
| ANXA3 | Homo sapiens annexin A3 (ANXA3), mRNA. | 1,1972 | 9,5160 | | 5,1903 | | 0,000472 | | | 0,00525 |
| ASAM | Homo sapiens adipocyte-specific adhesion molecule (ASAM), mRNA. | 1,1972 | 8,1155 | | 3,4026 | | 0,007194 | | | 0,03156 |
| MTSS1 | Homo sapiens metastasis suppressor 1 (MTSS1), mRNA. | 1,1963 | 9,9410 | | 5,4321 | | 0,000338 | | | 0,00433 |
| PGM3 | Homo sapiens phosphoglucomutase 3 (PGM3), mRNA. | 1,1957 | 9,6699 | | 11,1614 | | 0,000001 | | | 0,00031 |
| EHD1 | Homo sapiens EH-domain containing 1 (EHD1), mRNA. | 1,1953 | 9,6096 | | 8,2384 | | 0,000012 | | | 0,00088 |
| HIATL2 | Homo sapiens hippocampus abundant transcript-like 2 (HIATL2), non-coding RNA. | 1,1952 | 9,8096 | | 5,1853 | | 0,000475 | | | 0,00528 |
| KLHL21 | Homo sapiens kelch-like 21 (Drosophila) (KLHL21), mRNA. | 1,1951 | 8,4318 | | 4,3329 | | 0,001649 | | | 0,01131 |
| NET1 | Homo sapiens neuroepithelial cell transforming 1 (NET1), transcript variant 2, mRNA. | 1,1946 | 9,3416 | | 5,0076 | | 0,000610 | | | 0,00607 |
| WDR45 | Homo sapiens WD repeat domain 45 (WDR45), transcript variant 1, mRNA. | 1,1942 | 8,0929 | | 9,3146 | | 0,000004 | | | 0,00054 |
| SPATA2L | Homo sapiens spermatogenesis associated 2-like (SPATA2L), mRNA. | 1,1931 | 8,3157 | | 9,6678 | | 0,000003 | | | 0,00049 |
| KCNH6 | Homo sapiens potassium voltage-gated channel, subfamily H (eag-related), member 6 (KCNH6), transcript variant 1, mRNA. | 1,1928 | 11,3106 | | 5,5634 | | 0,000283 | | | 0,00394 |
| FLJ36131 | PREDICTED: Homo sapiens hypothetical protein FLJ36131, transcript variant 2 (FLJ36131), mRNA. | 1,1920 | 8,9058 | | 5,8434 | | 0,000195 | | | 0,00322 |
| DAB2 | Homo sapiens disabled homolog 2, mitogen-responsive phosphoprotein (Drosophila) (DAB2), mRNA. | 1,1911 | 8,3011 | | 4,4323 | | 0,001419 | | | 0,01024 |
| ITGA3 | Homo sapiens integrin, alpha 3 (antigen CD49C, alpha 3 subunit of VLA-3 receptor) (ITGA3), transcript variant a, mRNA. | 1,1907 | 11,4394 | | 7,1834 | | 0,000038 | | | 0,00141 |
| PGM3 | Homo sapiens phosphoglucomutase 3 (PGM3), mRNA. | 1,1906 | 9,0847 | | 11,4646 | | 0,000001 | | | 0,00029 |
| PSME4 | Homo sapiens proteasome (prosome, macropain) activator subunit 4 (PSME4), mRNA. | 1,1890 | 10,1388 | | 3,9546 | | 0,002962 | | | 0,01676 |
| HNRNPU | Homo sapiens heterogeneous nuclear ribonucleoprotein U (scaffold attachment factor A) (HNRNPU), transcript variant 2, mRNA. | 1,1888 | 8,4858 | | 5,2217 | | 0,000451 | | | 0,00511 |
| LOC100128084 | PREDICTED: Homo sapiens hypothetical protein LOC100128084 (LOC100128084), mRNA. | 1,1881 | 10,8177 | | 5,7744 | | 0,000213 | | | 0,00338 |
| TXNRD1 | Homo sapiens thioredoxin reductase 1 (TXNRD1), transcript variant 5, mRNA. | 1,1880 | 9,7931 | | 4,4327 | | 0,001418 | | | 0,01024 |
| MXD4 | Homo sapiens MAX dimerization protein 4 (MXD4), mRNA. | 1,1880 | 8,3719 | | 3,1281 | | 0,011319 | | | 0,04358 |
| UBR4 | Homo sapiens ubiquitin protein ligase E3 component n-recognin 4 (UBR4), mRNA. | 1,1876 | 8,8042 | | 6,7956 | | 0,000060 | | | 0,00175 |
| GRIPAP1 | Homo sapiens GRIP1 associated protein 1 (GRIPAP1), transcript variant 2, mRNA. | 1,1875 | 9,4190 | | 5,7375 | | 0,000224 | | | 0,00348 |
| ACSS2 | Homo sapiens acyl-CoA synthetase short-chain family member 2 (ACSS2), transcript variant 2, mRNA. | 1,1849 | 9,1835 | | 4,8495 | | 0,000766 | | | 0,00695 |
| HGS | Homo sapiens hepatocyte growth factor-regulated tyrosine kinase substrate (HGS), mRNA. | 1,1845 | 10,5794 | | 5,4409 | | 0,000334 | | | 0,00431 |
| CRCP | Homo sapiens CGRP receptor component (CRCP), transcript variant 1, mRNA. | 1,1839 | 9,1526 | | 4,0203 | | 0,002672 | | | 0,01568 |
| PPP2R2C | Homo sapiens protein phosphatase 2 (formerly 2A), regulatory subunit B, gamma isoform (PPP2R2C), transcript variant 2, mRNA. | 1,1836 | 8,4530 | | 4,6330 | | 0,001051 | | | 0,00847 |
| HCG2P7 | Homo sapiens HLA complex group 2 pseudogene 7 (HCG2P7), non-coding RNA. | 1,1826 | 11,0631 | | 5,5854 | | 0,000274 | | | 0,00387 |
| NET1 | Homo sapiens neuroepithelial cell transforming 1 (NET1), transcript variant 1, mRNA. | 1,1810 | 11,6435 | | 5,1011 | | 0,000534 | | | 0,00562 |
| MCM8 | Homo sapiens minichromosome maintenance complex component 8 (MCM8), transcript variant 1, mRNA. | 1,1800 | 10,7166 | | 4,3740 | | 0,001550 | | | 0,01086 |
| LOC644250 | PREDICTED: Homo sapiens hypothetical protein LOC644250 (LOC644250), mRNA. | 1,1782 | 8,4273 | | 7,6102 | | 0,000024 | | | 0,00115 |
| BMS1P5 | Homo sapiens BMS1 pseudogene 5 (BMS1P5), non-coding RNA. | 1,1756 | 10,4374 | | 5,0662 | | 0,000562 | | | 0,00579 |
| METRNL | PREDICTED: Homo sapiens meteorin, glial cell differentiation regulator-like (METRNL), mRNA. | 1,1738 | 7,9758 | | 9,0089 | | 0,000006 | | | 0,00063 |
| DEM1 | Homo sapiens defects in morphology 1 homolog (S. cerevisiae) (DEM1), mRNA. | 1,1736 | 8,7471 | | 5,6359 | | 0,000256 | | | 0,00374 |
| AXUD1 | Homo sapiens AXIN1 up-regulated 1 (AXUD1), mRNA. | 1,1735 | 8,4309 | | 4,0761 | | 0,002449 | | | 0,01479 |
| S100A2 | Homo sapiens S100 calcium binding protein A2 (S100A2), mRNA. | 1,1732 | 11,0055 | | 4,2739 | | 0,001805 | | | 0,01201 |
| TRIM8 | Homo sapiens tripartite motif-containing 8 (TRIM8), mRNA. | 1,1732 | 10,1044 | | 6,3388 | | 0,000104 | | | 0,00227 |
| CDKN2AIPNL | Homo sapiens CDKN2A interacting protein N-terminal like (CDKN2AIPNL), mRNA. | 1,1721 | 11,3817 | | 4,8506 | | 0,000765 | | | 0,00695 |
| LOC100129211 | PREDICTED: Homo sapiens hypothetical protein LOC100129211 (LOC100129211), mRNA. | 1,1706 | 8,7809 | | 6,6923 | | 0,000067 | | | 0,00184 |
| TIMP1 | Homo sapiens TIMP metallopeptidase inhibitor 1 (TIMP1), mRNA. | 1,1695 | 10,4128 | | 5,1968 | | 0,000467 | | | 0,00522 |
| LOC653086 | PREDICTED: Homo sapiens similar to RAN-binding protein 2-like 1 isoform 2, transcript variant 10 (LOC653086), mRNA. | 1,1693 | 9,1476 | | 5,3177 | | 0,000395 | | | 0,00473 |
| SH3KBP1 | Homo sapiens SH3-domain kinase binding protein 1 (SH3KBP1), transcript variant 1, mRNA. | 1,1688 | 9,4784 | | 6,0243 | | 0,000154 | | | 0,00282 |
| DDIT3 | Homo sapiens DNA-damage-inducible transcript 3 (DDIT3), mRNA. | 1,1680 | 8,1318 | | 8,3472 | | 0,000011 | | | 0,00083 |
| ZNF738 | PREDICTED: Homo sapiens misc_RNA (ZNF738), partial miscRNA. | 1,1679 | 9,2224 | | 5,2457 | | 0,000437 | | | 0,00500 |
| HSD17B7 | Homo sapiens hydroxysteroid (17-beta) dehydrogenase 7 (HSD17B7), mRNA. | 1,1671 | 9,5320 | | 6,4377 | | 0,000092 | | | 0,00215 |
| MAD1L1 | Homo sapiens MAD1 mitotic arrest deficient-like 1 (yeast) (MAD1L1), transcript variant 2, mRNA. | 1,1669 | 8,4897 | | 4,5063 | | 0,001270 | | | 0,00951 |
| DNAJB9 | Homo sapiens DnaJ (Hsp40) homolog, subfamily B, member 9 (DNAJB9), mRNA. | 1,1644 | 8,1911 | | 25,4606 | | 0,000000 | | | 0,00001 |
| FCAR | Homo sapiens Fc fragment of IgA, receptor for (FCAR), transcript variant 9, mRNA. | 1,1627 | 8,4070 | | 7,7597 | | 0,000020 | | | 0,00107 |
| C21orf24 | Homo sapiens chromosome 21 open reading frame 24 (C21orf24), mRNA. | 1,1613 | 10,1688 | | 4,5445 | | 0,001199 | | | 0,00919 |
| JUP | Homo sapiens junction plakoglobin (JUP), transcript variant 1, mRNA. | 1,1611 | 11,8481 | | 6,5272 | | 0,000082 | | | 0,00202 |
| C14orf85 | Homo sapiens chromosome 14 open reading frame 85 (C14orf85), non-coding RNA. | 1,1574 | 11,1715 | | 6,5987 | | 0,000075 | | | 0,00194 |
| LOC441087 | Homo sapiens hypothetical gene supported by AK125735 (LOC441087), mRNA. | 1,1573 | 11,5472 | | 7,3555 | | 0,000031 | | | 0,00129 |
| JUNB | Homo sapiens jun B proto-oncogene (JUNB), mRNA. | 1,1544 | 9,3846 | | 3,6302 | | 0,004968 | | | 0,02404 |
| ASS1 | Homo sapiens argininosuccinate synthetase 1 (ASS1), transcript variant 2, mRNA. | 1,1527 | 9,1281 | | 5,4131 | | 0,000347 | | | 0,00437 |
| CCNL1 | Homo sapiens cyclin L1 (CCNL1), mRNA. | 1,1521 | 9,0734 | | 6,2101 | | 0,000122 | | | 0,00246 |
| PNPT1 | Homo sapiens polyribonucleotide nucleotidyltransferase 1 (PNPT1), mRNA. | 1,1518 | 9,0507 | | 5,8022 | | 0,000206 | | | 0,00332 |
| LOC100130445 | PREDICTED: Homo sapiens similar to AML-associated zinc finger protein (LOC100130445), mRNA. | 1,1516 | 10,8971 | | 5,5035 | | 0,000306 | | | 0,00412 |
| P4HA2 | Homo sapiens prolyl 4-hydroxylase, alpha polypeptide II (P4HA2), transcript variant 2, mRNA. | 1,1516 | 10,8056 | | 4,9876 | | 0,000628 | | | 0,00615 |
| GLIPR1 | Homo sapiens GLI pathogenesis-related 1 (GLIPR1), mRNA. | 1,1512 | 8,3514 | | 9,0700 | | 0,000005 | | | 0,00061 |
| DUSP19 | Homo sapiens dual specificity phosphatase 19 (DUSP19), mRNA. | 1,1503 | 10,3220 | | 6,3896 | | 0,000097 | | | 0,00220 |
| CSNK1D | Homo sapiens casein kinase 1, delta (CSNK1D), transcript variant 1, mRNA. | 1,1465 | 9,4577 | | 7,4971 | | 0,000027 | | | 0,00121 |
| STX3 | Homo sapiens syntaxin 3 (STX3), mRNA. | 1,1463 | 8,5542 | | 3,9326 | | 0,003067 | | | 0,01712 |
| H1F0 | Homo sapiens H1 histone family, member 0 (H1F0), mRNA. | 1,1445 | 10,6175 | | 3,7816 | | 0,003897 | | | 0,02021 |
| C15orf63 | Homo sapiens chromosome 15 open reading frame 63 (C15orf63), mRNA. | 1,1441 | 9,5787 | | 5,5244 | | 0,000298 | | | 0,00406 |
| C5orf41 | Homo sapiens chromosome 5 open reading frame 41 (C5orf41), mRNA. | 1,1426 | 7,7793 | | 6,7486 | | 0,000063 | | | 0,00180 |
| C5orf41 | Homo sapiens chromosome 5 open reading frame 41 (C5orf41), mRNA. | 1,1410 | 7,7368 | | 8,3717 | | 0,000011 | | | 0,00083 |
| ARHGEF4 | Homo sapiens Rho guanine nucleotide exchange factor (GEF) 4 (ARHGEF4), transcript variant 1, mRNA. | 1,1408 | 8,4266 | | 3,2850 | | 0,008728 | | | 0,03610 |
| RHBDF1 | Homo sapiens rhomboid 5 homolog 1 (Drosophila) (RHBDF1), mRNA. | 1,1400 | 9,6190 | | 5,9298 | | 0,000174 | | | 0,00303 |
| LOC392437 | PREDICTED: Homo sapiens misc_RNA (LOC392437), miscRNA. | 1,1397 | 11,9699 | | 4,1664 | | 0,002129 | | | 0,01341 |
| GJC1 | Homo sapiens gap junction protein, gamma 1, 45kDa (GJC1), transcript variant 1, mRNA. | 1,1393 | 11,9663 | | 5,5564 | | 0,000285 | | | 0,00397 |
| ZYX | Homo sapiens zyxin (ZYX), transcript variant 1, mRNA. | 1,1393 | 9,8078 | | 7,4990 | | 0,000027 | | | 0,00121 |
| N4BP2 | Homo sapiens Nedd4 binding protein 2 (N4BP2), mRNA. | 1,1357 | 8,3012 | | 6,2259 | | 0,000119 | | | 0,00245 |
| C4orf34 | Homo sapiens chromosome 4 open reading frame 34 (C4orf34), mRNA. | 1,1348 | 10,1970 | | 4,0030 | | 0,002746 | | | 0,01598 |
| EID2B | Homo sapiens EP300 interacting inhibitor of differentiation 2B (EID2B), mRNA. | 1,1338 | 10,6052 | | 5,8257 | | 0,000199 | | | 0,00326 |
| YRDC | Homo sapiens yrdC domain containing (E. coli) (YRDC), nuclear gene encoding mitochondrial protein, mRNA. | 1,1337 | 9,9856 | | 5,0649 | | 0,000563 | | | 0,00579 |
| HSPC268 | Homo sapiens hypothetical protein HSPC268 (HSPC268), mRNA. | 1,1333 | 8,9024 | | 5,6424 | | 0,000254 | | | 0,00373 |
| MAFF | Homo sapiens v-maf musculoaponeurotic fibrosarcoma oncogene homolog F (avian) (MAFF), transcript variant 1, mRNA. | 1,1329 | 7,8955 | | 9,0876 | | 0,000005 | | | 0,00061 |
| IL10 | Homo sapiens interleukin 10 (IL10), mRNA. | 1,1320 | 9,5740 | | 5,4092 | | 0,000348 | | | 0,00437 |
| ZNF598 | Homo sapiens zinc finger protein 598 (ZNF598), mRNA. | 1,1320 | 10,3349 | | 6,8979 | | 0,000053 | | | 0,00167 |
| BAIAP2L1 | Homo sapiens BAI1-associated protein 2-like 1 (BAIAP2L1), mRNA. | 1,1312 | 11,0988 | | 11,0774 | | 0,000001 | | | 0,00031 |
| FLJ46309 | Homo sapiens hypothetical protein LOC649598 (FLJ46309), mRNA. | 1,1309 | 10,5625 | | 4,6015 | | 0,001102 | | | 0,00873 |
| FNBP4 | Homo sapiens formin binding protein 4 (FNBP4), mRNA. | 1,1292 | 9,5501 | | 5,3032 | | 0,000403 | | | 0,00479 |
| KLF4 | Homo sapiens Kruppel-like factor 4 (gut) (KLF4), mRNA. | 1,1283 | 8,0928 | | 7,5731 | | 0,000025 | | | 0,00117 |
| XBP1 | Homo sapiens X-box binding protein 1 (XBP1), transcript variant 2, mRNA. | 1,1268 | 10,7936 | | 5,9169 | | 0,000177 | | | 0,00306 |
| DDX51 | Homo sapiens DEAD (Asp-Glu-Ala-Asp) box polypeptide 51 (DDX51), mRNA. | 1,1268 | 9,5110 | | 5,0547 | | 0,000571 | | | 0,00584 |
| CREB1 | Homo sapiens cAMP responsive element binding protein 1 (CREB1), transcript variant A, mRNA. | 1,1264 | 11,4866 | | 4,6876 | | 0,000970 | | | 0,00810 |
| CHMP1B | Homo sapiens chromatin modifying protein 1B (CHMP1B), mRNA. | 1,1264 | 11,6412 | | 4,8467 | | 0,000769 | | | 0,00697 |
| KIFC2 | Homo sapiens kinesin family member C2 (KIFC2), mRNA. | 1,1245 | 9,1339 | | 7,4857 | | 0,000027 | | | 0,00121 |
| LOC100128062 | PREDICTED: Homo sapiens misc_RNA (LOC100128062), miscRNA. | 1,1216 | 9,4712 | | 6,4044 | | 0,000096 | | | 0,00217 |
| CDCP1 | Homo sapiens CUB domain containing protein 1 (CDCP1), transcript variant 2, mRNA. | 1,1176 | 8,0474 | | 3,9038 | | 0,003210 | | | 0,01766 |
| CCBE1 | Homo sapiens collagen and calcium binding EGF domains 1 (CCBE1), mRNA. | 1,1171 | 8,4755 | | 5,2744 | | 0,000419 | | | 0,00491 |
| LOC285741 | PREDICTED: Homo sapiens misc_RNA (LOC285741), miscRNA. | 1,1132 | 9,8814 | | 4,1746 | | 0,002103 | | | 0,01330 |
| SLC44A4 | Homo sapiens solute carrier family 44, member 4 (SLC44A4), transcript variant 2, mRNA. | 1,1125 | 11,2532 | | 5,6845 | | 0,000240 | | | 0,00361 |
| COL7A1 | Homo sapiens collagen, type VII, alpha 1 (epidermolysis bullosa, dystrophic, dominant and recessive) (COL7A1), mRNA. | 1,1123 | 11,5347 | | 4,7828 | | 0,000844 | | | 0,00742 |
| CLIP2 | Homo sapiens CAP-GLY domain containing linker protein 2 (CLIP2), transcript variant 2, mRNA. | 1,1123 | 8,5817 | | 7,4735 | | 0,000027 | | | 0,00121 |
| P4HA2 | Homo sapiens prolyl 4-hydroxylase, alpha polypeptide II (P4HA2), transcript variant 3, mRNA. | 1,1079 | 9,7462 | | 4,4664 | | 0,001348 | | | 0,00993 |
| SCG5 | Homo sapiens secretogranin V (7B2 protein) (SCG5), mRNA. | 1,1068 | 7,8346 | | 3,3852 | | 0,007402 | | | 0,03221 |
| LYPD3 | Homo sapiens LY6/PLAUR domain containing 3 (LYPD3), mRNA. | 1,1048 | 7,9941 | | 3,8733 | | 0,003368 | | | 0,01827 |
| FAM107B | Homo sapiens family with sequence similarity 107, member B (FAM107B), mRNA. | 1,1045 | 8,7574 | | 7,6089 | | 0,000024 | | | 0,00115 |
| LOC653489 | PREDICTED: Homo sapiens similar to Ran-binding protein 2 (RanBP2) (Nuclear pore complex protein Nup358) (Nucleoporin Nup358) (358 kDa nucleoporin) (P270), transcript variant 7 (LOC653489), mRNA. | 1,1042 | 8,7395 | | 8,4511 | | 0,000010 | | | 0,00080 |
| VIL2 | Homo sapiens villin 2 (ezrin) (VIL2), mRNA. | 1,1019 | 12,4829 | | 10,9193 | | 0,000001 | | | 0,00031 |
| SEMA3E | Homo sapiens sema domain, immunoglobulin domain (Ig), short basic domain, secreted, (semaphorin) 3E (SEMA3E), mRNA. | 1,1006 | 9,1633 | | 9,3016 | | 0,000004 | | | 0,00054 |
| SH2D5 | PREDICTED: Homo sapiens SH2 domain containing 5 (SH2D5), mRNA. | 1,1003 | 8,2734 | | 5,3425 | | 0,000382 | | | 0,00461 |
| PIM3 | PREDICTED: Homo sapiens pim-3 oncogene (PIM3), mRNA. | 1,0988 | 8,5226 | | 8,6303 | | 0,000008 | | | 0,00073 |
| LOC389787 | PREDICTED: Homo sapiens similar to Translationally-controlled tumor protein (TCTP) (p23) (Histamine-releasing factor) (HRF) (Fortilin) (LOC389787), mRNA. | 1,0982 | 11,0598 | | 5,0804 | | 0,000550 | | | 0,00572 |
| LOC100128098 | PREDICTED: Homo sapiens hypothetical protein LOC100128098 (LOC100128098), mRNA. | 1,0965 | 8,4594 | | 4,1628 | | 0,002141 | | | 0,01346 |
| FTHL8 | Homo sapiens ferritin, heavy polypeptide-like 8 (FTHL8) on chromosome X. | 1,0923 | 11,0560 | | 3,2641 | | 0,009035 | | | 0,03699 |
| RAB11FIP5 | Homo sapiens RAB11 family interacting protein 5 (class I) (RAB11FIP5), mRNA. | 1,0912 | 8,3200 | | 5,4113 | | 0,000347 | | | 0,00437 |
| IL24 | Homo sapiens interleukin 24 (IL24), transcript variant 1, mRNA. | 1,0898 | 7,6631 | | 3,6263 | | 0,005000 | | | 0,02416 |
| OCIAD1 | Homo sapiens OCIA domain containing 1 (OCIAD1), transcript variant 5, mRNA. | 1,0867 | 9,9542 | | 5,2555 | | 0,000431 | | | 0,00497 |
| FKTN | Homo sapiens fukutin (FKTN), transcript variant 2, mRNA. | 1,0862 | 11,3730 | | 5,5151 | | 0,000302 | | | 0,00409 |
| SFRS17A | Homo sapiens splicing factor, arginine/serine-rich 17A (SFRS17A), transcript variant 1, mRNA. | 1,0838 | 8,7562 | | 7,3904 | | 0,000030 | | | 0,00127 |
| LOC100134159 | PREDICTED: Homo sapiens similar to Coiled-coil domain containing 144B (LOC100134159), mRNA. | 1,0834 | 9,4288 | | 4,6779 | | 0,000984 | | | 0,00817 |
| LTBP4 | Homo sapiens latent transforming growth factor beta binding protein 4 (LTBP4), transcript variant 3, mRNA. | 1,0829 | 9,2083 | | 7,2665 | | 0,000035 | | | 0,00134 |
| KLHL24 | Homo sapiens kelch-like 24 (Drosophila) (KLHL24), mRNA. | 1,0826 | 8,3663 | | 3,5782 | | 0,005404 | | | 0,02552 |
| GFPT2 | Homo sapiens glutamine-fructose-6-phosphate transaminase 2 (GFPT2), mRNA. | 1,0819 | 7,9346 | | 5,8519 | | 0,000193 | | | 0,00320 |
| SLMO1 | Homo sapiens slowmo homolog 1 (Drosophila) (SLMO1), mRNA. | 1,0796 | 9,6368 | | 5,0199 | | 0,000600 | | | 0,00600 |
| LOC729978 | PREDICTED: Homo sapiens similar to LOC339047 protein, transcript variant 2 (LOC729978), mRNA. | 1,0784 | 9,9115 | | 6,3775 | | 0,000099 | | | 0,00222 |
| RNY4 | Homo sapiens RNA, Ro-associated Y4 (RNY4), small cytoplasmic RNA. | 1,0782 | 8,7646 | | 4,9025 | | 0,000709 | | | 0,00661 |
| LOC729252 | PREDICTED: Homo sapiens similar to Keratin, type I cytoskeletal 14 (Cytokeratin-14) (CK-14) (Keratin-14) (K14) (LOC729252), mRNA. | 1,0780 | 8,2359 | | 4,5212 | | 0,001242 | | | 0,00938 |
| IFFO1 | Homo sapiens intermediate filament family orphan 1 (IFFO1), transcript variant 2, mRNA. | 1,0767 | 8,0043 | | 6,6060 | | 0,000075 | | | 0,00193 |
| NAGK | Homo sapiens N-acetylglucosamine kinase (NAGK), mRNA. | 1,0761 | 8,9258 | | 6,7383 | | 0,000064 | | | 0,00181 |
| EIF1B | Homo sapiens eukaryotic translation initiation factor 1B (EIF1B), mRNA. | 1,0748 | 9,5944 | | 6,9446 | | 0,000050 | | | 0,00161 |
| FAM119A | Homo sapiens family with sequence similarity 119, member A (FAM119A), mRNA. | 1,0735 | 10,5218 | | 5,8586 | | 0,000191 | | | 0,00319 |
| KLF11 | PREDICTED: Homo sapiens Kruppel-like factor 11 (KLF11), mRNA. | 1,0733 | 8,0135 | | 5,4275 | | 0,000340 | | | 0,00434 |
| LOC392437 | PREDICTED: Homo sapiens misc_RNA (LOC392437), miscRNA. | 1,0712 | 12,7817 | | 3,9817 | | 0,002839 | | | 0,01631 |
| FLJ35390 | Homo sapiens hypothetical LOC255031 (FLJ35390), transcript variant 1, non-coding RNA. | 1,0691 | 8,6963 | | 4,9752 | | 0,000639 | | | 0,00620 |
| CHRNA5 | Homo sapiens cholinergic receptor, nicotinic, alpha 5 (CHRNA5), mRNA. | 1,0676 | 9,0867 | | 4,9839 | | 0,000631 | | | 0,00617 |
| SH3BGRL3 | Homo sapiens SH3 domain binding glutamic acid-rich protein like 3 (SH3BGRL3), mRNA. | 1,0661 | 11,0112 | | 3,5942 | | 0,005266 | | | 0,02504 |
| CITED2 | Homo sapiens Cbp/p300-interacting transactivator, with Glu/Asp-rich carboxy-terminal domain, 2 (CITED2), transcript variant 1, mRNA. | 1,0652 | 8,5603 | | 7,3690 | | 0,000031 | | | 0,00128 |
| C16orf72 | Homo sapiens chromosome 16 open reading frame 72 (C16orf72), mRNA. | 1,0649 | 8,9791 | | 7,3570 | | 0,000031 | | | 0,00129 |
| SOX9 | Homo sapiens SRY (sex determining region Y)-box 9 (campomelic dysplasia, autosomal sex-reversal) (SOX9), mRNA. | 1,0647 | 9,0786 | | 3,2427 | | 0,009360 | | | 0,03788 |
| ABTB1 | Homo sapiens ankyrin repeat and BTB (POZ) domain containing 1 (ABTB1), transcript variant 1, mRNA. | 1,0643 | 7,7656 | | 5,7209 | | 0,000229 | | | 0,00352 |
| CCDC130 | Homo sapiens coiled-coil domain containing 130 (CCDC130), mRNA. | 1,0627 | 9,3527 | | 7,4043 | | 0,000030 | | | 0,00126 |
| VIM | Homo sapiens vimentin (VIM), mRNA. | 1,0574 | 11,3818 | | 4,1752 | | 0,002100 | | | 0,01330 |
| LOC729231 | PREDICTED: Homo sapiens misc_RNA (LOC729231), miscRNA. | 1,0566 | 8,2528 | | 6,4655 | | 0,000089 | | | 0,00211 |
| AXIN1 | Homo sapiens axin 1 (AXIN1), transcript variant 2, mRNA. | 1,0558 | 9,2641 | | 9,0574 | | 0,000005 | | | 0,00061 |
| C20orf199 | Homo sapiens chromosome 20 open reading frame 199 (C20orf199), transcript variant 3, non-coding RNA. | 1,0532 | 11,8466 | | 5,9791 | | 0,000163 | | | 0,00292 |
| UGDH | Homo sapiens UDP-glucose dehydrogenase (UGDH), mRNA. | 1,0526 | 9,3724 | | 12,4728 | | 0,000000 | | | 0,00022 |
| MGLL | Homo sapiens monoglyceride lipase (MGLL), transcript variant 1, mRNA. | 1,0519 | 8,0030 | | 3,1244 | | 0,011387 | | | 0,04376 |
| HES4 | Homo sapiens hairy and enhancer of split 4 (Drosophila) (HES4), mRNA. | 1,0514 | 9,3166 | | 4,6813 | | 0,000979 | | | 0,00815 |
| FGD6 | Homo sapiens FYVE, RhoGEF and PH domain containing 6 (FGD6), mRNA. | 1,0510 | 8,1196 | | 8,7072 | | 0,000008 | | | 0,00071 |
| LOC642469 | PREDICTED: Homo sapiens misc_RNA (LOC642469), miscRNA. | 1,0507 | 8,6163 | | 5,6162 | | 0,000263 | | | 0,00381 |
| ETS1 | Homo sapiens v-ets erythroblastosis virus E26 oncogene homolog 1 (avian) (ETS1), mRNA. | 1,0503 | 8,9982 | | 7,1661 | | 0,000039 | | | 0,00141 |
| DSC2 | Homo sapiens desmocollin 2 (DSC2), transcript variant Dsc2b, mRNA. | 1,0496 | 9,1217 | | 3,7072 | | 0,004389 | | | 0,02199 |
| CASZ1 | Homo sapiens castor zinc finger 1 (CASZ1), transcript variant 2, mRNA. | 1,0463 | 8,0897 | | 4,7453 | | 0,000891 | | | 0,00769 |
| LOC728620 | PREDICTED: Homo sapiens misc_RNA (LOC728620), miscRNA. | 1,0454 | 11,7250 | | 5,9859 | | 0,000162 | | | 0,00291 |
| IRF6 | Homo sapiens interferon regulatory factor 6 (IRF6), mRNA. | 1,0442 | 8,3528 | | 6,2840 | | 0,000111 | | | 0,00235 |
| WASL | Homo sapiens Wiskott-Aldrich syndrome-like (WASL), mRNA. | 1,0438 | 9,0583 | | 8,0423 | | 0,000015 | | | 0,00095 |
| AVPI1 | Homo sapiens arginine vasopressin-induced 1 (AVPI1), mRNA. | 1,0420 | 9,5843 | | 3,7221 | | 0,004285 | | | 0,02163 |
| FAM43A | Homo sapiens family with sequence similarity 43, member A (FAM43A), mRNA. | 1,0418 | 8,3563 | | 3,2327 | | 0,009516 | | | 0,03833 |
| LRP10 | Homo sapiens low density lipoprotein receptor-related protein 10 (LRP10), mRNA. | 1,0418 | 10,1201 | | 5,1941 | | 0,000469 | | | 0,00523 |
| VPS37B | Homo sapiens vacuolar protein sorting 37 homolog B (S. cerevisiae) (VPS37B), mRNA. | 1,0407 | 9,6223 | | 5,0851 | | 0,000547 | | | 0,00569 |
| FAM63A | Homo sapiens family with sequence similarity 63, member A (FAM63A), transcript variant 1, mRNA. | 1,0399 | 8,3579 | | 6,6457 | | 0,000071 | | | 0,00190 |
| QRFPR | Homo sapiens pyroglutamylated RFamide peptide receptor (QRFPR), mRNA. | 1,0394 | 9,8402 | | 5,3359 | | 0,000385 | | | 0,00464 |
| NFIL3 | Homo sapiens nuclear factor, interleukin 3 regulated (NFIL3), mRNA. | 1,0370 | 8,6505 | | 7,3381 | | 0,000032 | | | 0,00129 |
| FRMD6 | Homo sapiens FERM domain containing 6 (FRMD6), mRNA. | 1,0367 | 9,6869 | | 6,8011 | | 0,000059 | | | 0,00174 |
| SERPINB8 | Homo sapiens serpin peptidase inhibitor, clade B (ovalbumin), member 8 (SERPINB8), transcript variant 2, mRNA. | 1,0364 | 8,0878 | | 6,2174 | | 0,000121 | | | 0,00245 |
| PINK1 | Homo sapiens PTEN induced putative kinase 1 (PINK1), nuclear gene encoding mitochondrial protein, mRNA. | 1,0358 | 8,9697 | | 3,6922 | | 0,004497 | | | 0,02241 |
| PIP5K2B | Homo sapiens phosphatidylinositol-4-phosphate 5-kinase, type II, beta (PIP5K2B), transcript variant 2, mRNA. | 1,0345 | 8,6566 | | 4,4148 | | 0,001457 | | | 0,01044 |
| FLJ44124 | Homo sapiens hypothetical protein LOC641737 (FLJ44124), mRNA. | 1,0335 | 9,7527 | | 4,8943 | | 0,000718 | | | 0,00665 |
| GABPB2 | Homo sapiens GA binding protein transcription factor, beta subunit 2 (GABPB2), mRNA. | 1,0334 | 12,0704 | | 5,4193 | | 0,000344 | | | 0,00435 |
| FOXD1 | Homo sapiens forkhead box D1 (FOXD1), mRNA. | 1,0332 | 8,7256 | | 4,2024 | | 0,002014 | | | 0,01292 |
| NBPF20 | Homo sapiens neuroblastoma breakpoint family, member 20 (NBPF20), mRNA. | 1,0327 | 8,7513 | | 4,0366 | | 0,002605 | | | 0,01544 |
| EIF2C2 | Homo sapiens eukaryotic translation initiation factor 2C, 2 (EIF2C2), mRNA. | 1,0312 | 9,5482 | | 5,7676 | | 0,000215 | | | 0,00340 |
| ABTB1 | Homo sapiens ankyrin repeat and BTB (POZ) domain containing 1 (ABTB1), transcript variant 3, mRNA. | 1,0310 | 7,8227 | | 5,5714 | | 0,000280 | | | 0,00392 |
| BCL2L1 | Homo sapiens BCL2-like 1 (BCL2L1), nuclear gene encoding mitochondrial protein, transcript variant 1, mRNA. | 1,0305 | 11,4377 | | 6,3187 | | 0,000106 | | | 0,00229 |
| ADRB2 | Homo sapiens adrenergic, beta-2-, receptor, surface (ADRB2), mRNA. | 1,0298 | 8,2322 | | 4,5679 | | 0,001158 | | | 0,00901 |
| RRAGC | Homo sapiens Ras-related GTP binding C (RRAGC), mRNA. | 1,0286 | 8,9720 | | 5,6168 | | 0,000263 | | | 0,00381 |
| HAS3 | Homo sapiens hyaluronan synthase 3 (HAS3), transcript variant 1, mRNA. | 1,0284 | 9,9710 | | 5,2699 | | 0,000422 | | | 0,00493 |
| PIK4CA | Homo sapiens phosphatidylinositol 4-kinase, catalytic, alpha polypeptide (PIK4CA), transcript variant 1, mRNA. | 1,0281 | 9,3963 | | 9,0652 | | 0,000005 | | | 0,00061 |
| ANXA2P1 | Homo sapiens annexin A2 pseudogene 1 (ANXA2P1) on chromosome 4. | 1,0279 | 11,7282 | | 4,9806 | | 0,000634 | | | 0,00617 |
| LOC390345 | PREDICTED: Homo sapiens misc_RNA (LOC390345), miscRNA. | 1,0267 | 12,1469 | | 4,7324 | | 0,000908 | | | 0,00779 |
| BPGM | Homo sapiens 2,3-bisphosphoglycerate mutase (BPGM), transcript variant 1, mRNA. | 1,0258 | 8,4805 | | 3,4557 | | 0,006596 | | | 0,02961 |
| WARS | Homo sapiens tryptophanyl-tRNA synthetase (WARS), transcript variant 1, mRNA. | 1,0257 | 9,4948 | | 3,0234 | | 0,013477 | | | 0,04976 |
| SDC4 | Homo sapiens syndecan 4 (SDC4), mRNA. | 1,0256 | 10,2132 | | 5,7962 | | 0,000207 | | | 0,00333 |
| CDAN1 | Homo sapiens congenital dyserythropoietic anemia, type I (CDAN1), mRNA. | 1,0224 | 11,0856 | | 5,1734 | | 0,000483 | | | 0,00532 |
| EDEM1 | Homo sapiens ER degradation enhancer, mannosidase alpha-like 1 (EDEM1), mRNA. | 1,0223 | 8,6317 | | 7,4874 | | 0,000027 | | | 0,00121 |
| RAB11FIP1 | Homo sapiens RAB11 family interacting protein 1 (class I) (RAB11FIP1), transcript variant 3, mRNA. | 1,0209 | 8,3951 | | 5,1651 | | 0,000488 | | | 0,00536 |
| LOC729090 | PREDICTED: Homo sapiens similar to Eukaryotic translation elongation factor 1 alpha 1 (LOC729090), mRNA. | 1,0194 | 12,1271 | | 5,2868 | | 0,000412 | | | 0,00485 |
| TMEM44 | Homo sapiens transmembrane protein 44 (TMEM44), transcript variant 1, mRNA. | 1,0172 | 8,6688 | | 3,8158 | | 0,003690 | | | 0,01945 |
| DGKA | Homo sapiens diacylglycerol kinase, alpha 80kDa (DGKA), transcript variant 4, mRNA. | 1,0170 | 8,8657 | | 3,5760 | | 0,005424 | | | 0,02558 |
| SDHALP1 | Homo sapiens succinate dehydrogenase complex, subunit A, flavoprotein pseudogene 1 (SDHALP1) on chromosome 3. | 1,0156 | 9,3986 | | 4,4669 | | 0,001347 | | | 0,00993 |
| CSF2RA | Homo sapiens colony stimulating factor 2 receptor, alpha, low-affinity (granulocyte-macrophage) (CSF2RA), transcript variant 6, mRNA. | 1,0155 | 9,5753 | | 5,9085 | | 0,000179 | | | 0,00307 |
| HMGA1 | Homo sapiens high mobility group AT-hook 1 (HMGA1), transcript variant 1, mRNA. | 1,0155 | 12,0006 | | 3,1318 | | 0,011250 | | | 0,04335 |
| HBP1 | Homo sapiens HMG-box transcription factor 1 (HBP1), mRNA. | 1,0143 | 8,0972 | | 5,1854 | | 0,000475 | | | 0,00528 |
| AKR1D1 | Homo sapiens aldo-keto reductase family 1, member D1 (delta 4-3-ketosteroid-5-beta-reductase) (AKR1D1), mRNA. | 1,0128 | 11,7309 | | 6,0540 | | 0,000148 | | | 0,00277 |
| CDK5R1 | Homo sapiens cyclin-dependent kinase 5, regulatory subunit 1 (p35) (CDK5R1), mRNA. | 1,0126 | 8,7977 | | 4,1035 | | 0,002347 | | | 0,01434 |
| LOC100133177 | PREDICTED: Homo sapiens misc_RNA (LOC100133177), miscRNA. | 1,0121 | 11,5547 | | 4,9469 | | 0,000666 | | | 0,00633 |
| RPPH1 | Homo sapiens ribonuclease P RNA component H1 (RPPH1), RNase P RNA. | 1,0116 | 7,6531 | | 5,9553 | | 0,000169 | | | 0,00298 |
| LOC100129269 | PREDICTED: Homo sapiens hypothetical protein LOC100129269 (LOC100129269), mRNA. | 1,0114 | 8,6003 | | 4,1915 | | 0,002048 | | | 0,01307 |
| ETV5 | Homo sapiens ets variant gene 5 (ets-related molecule) (ETV5), mRNA. | 1,0111 | 9,0801 | | 5,6300 | | 0,000258 | | | 0,00376 |
| PTPRE | Homo sapiens protein tyrosine phosphatase, receptor type, E (PTPRE), transcript variant 2, mRNA. | 1,0105 | 8,4737 | | 7,2068 | | 0,000037 | | | 0,00139 |
| LYN | Homo sapiens v-yes-1 Yamaguchi sarcoma viral related oncogene homolog (LYN), mRNA. | 1,0090 | 8,8692 | | 9,9075 | | 0,000002 | | | 0,00046 |
| DHRS7 | Homo sapiens dehydrogenase/reductase (SDR family) member 7 (DHRS7), mRNA. | 1,0083 | 10,2886 | | 4,3182 | | 0,001687 | | | 0,01149 |
| CYLN2 | Homo sapiens cytoplasmic linker 2 (CYLN2), transcript variant 2, mRNA. | 1,0070 | 8,6736 | | 5,9516 | | 0,000169 | | | 0,00299 |
| SHCBP1 | Homo sapiens SHC SH2-domain binding protein 1 (SHCBP1), mRNA. | 1,0068 | 11,3937 | | 6,8067 | | 0,000059 | | | 0,00174 |
| ZNF652 | Homo sapiens zinc finger protein 652 (ZNF652), mRNA. | 1,0060 | 9,4980 | | 5,8963 | | 0,000182 | | | 0,00309 |
| SEC16A | Homo sapiens SEC16 homolog A (S. cerevisiae) (SEC16A), mRNA. | 1,0044 | 9,3518 | | 4,7100 | | 0,000939 | | | 0,00797 |
| MANBA | Homo sapiens mannosidase, beta A, lysosomal (MANBA), mRNA. | 1,0036 | 8,7352 | | 4,0870 | | 0,002408 | | | 0,01461 |
| PODXL | Homo sapiens podocalyxin-like (PODXL), transcript variant 1, mRNA. | 1,0033 | 7,9924 | | 7,2749 | | 0,000034 | | | 0,00134 |
| LOC729603 | Homo sapiens calcium binding protein P22 pseudogene (LOC729603), non-coding RNA. | 1,0026 | 11,3689 | | 3,8334 | | 0,003588 | | | 0,01911 |
| SLC38A1 | Homo sapiens solute carrier family 38, member 1 (SLC38A1), transcript variant 1, mRNA. | 1,0017 | 9,0495 | | 7,5945 | | 0,000024 | | | 0,00116 |
| B4GALT5 | Homo sapiens UDP-Gal:betaGlcNAc beta 1,4- galactosyltransferase, polypeptide 5 (B4GALT5), mRNA. | 1,0015 | 10,4023 | | 5,0661 | | 0,000562 | | | 0,00579 |
| LOC100133233 | PREDICTED: Homo sapiens hypothetical protein LOC100133233 (LOC100133233), mRNA. | 1,0002 | 11,5820 | | 5,1850 | | 0,000475 | | | 0,00528 |
| LILRB1 | Homo sapiens leukocyte immunoglobulin-like receptor, subfamily B (with TM and ITIM domains), member 1 (LILRB1), transcript variant 2, mRNA. | 1,0000 | 10,7264 | | 4,5158 | | 0,001252 | | | 0,00943 |
| PELI1 | Homo sapiens pellino homolog 1 (Drosophila) (PELI1), mRNA. | 0,9998 | 7,9896 | | 5,8768 | | 0,000187 | | | 0,00313 |
| KIAA0355 | Homo sapiens KIAA0355 (KIAA0355), mRNA. | 0,9996 | 8,3776 | | 10,1305 | | 0,000002 | | | 0,00042 |
| KDM5B | Homo sapiens lysine (K)-specific demethylase 5B (KDM5B), mRNA. | 0,9991 | 9,4206 | | 5,2454 | | 0,000437 | | | 0,00500 |
| SLC25A37 | Homo sapiens solute carrier family 25, member 37 (SLC25A37), nuclear gene encoding mitochondrial protein, mRNA. | 0,9973 | 8,6572 | | 10,3001 | | 0,000002 | | | 0,00041 |
| FSTL3 | Homo sapiens follistatin-like 3 (secreted glycoprotein) (FSTL3), mRNA. | 0,9966 | 9,0759 | | 5,2101 | | 0,000459 | | | 0,00517 |
| C14orf173 | Homo sapiens chromosome 14 open reading frame 173 (C14orf173), transcript variant 2, mRNA. | 0,9964 | 9,5523 | | 7,0349 | | 0,000045 | | | 0,00152 |
| IL18 | Homo sapiens interleukin 18 (interferon-gamma-inducing factor) (IL18), mRNA. | 0,9943 | 11,7893 | | 8,9636 | | 0,000006 | | | 0,00064 |
| SGK1 | Homo sapiens serum/glucocorticoid regulated kinase 1 (SGK1), transcript variant 1, mRNA. | 0,9943 | 9,1971 | | 3,6906 | | 0,004508 | | | 0,02244 |
| FAM131A | Homo sapiens family with sequence similarity 131, member A (FAM131A), mRNA. | 0,9942 | 8,9759 | | 3,3367 | | 0,008015 | | | 0,03401 |
| LOC100130154 | PREDICTED: Homo sapiens similar to thymosin, beta 10 (LOC100130154), mRNA. | 0,9929 | 9,0097 | | 4,9674 | | 0,000646 | | | 0,00624 |
| MGC26356 | PREDICTED: Homo sapiens misc_RNA (MGC26356), miscRNA. | 0,9924 | 11,8135 | | 6,2164 | | 0,000121 | | | 0,00245 |
| MAFF | Homo sapiens v-maf musculoaponeurotic fibrosarcoma oncogene homolog F (avian) (MAFF), transcript variant 1, mRNA. | 0,9917 | 7,8443 | | 9,8775 | | 0,000003 | | | 0,00047 |
| LOC730820 | PREDICTED: Homo sapiens similar to nuclear receptor binding factor 2 (LOC730820), mRNA. | 0,9915 | 8,9027 | | 4,9744 | | 0,000640 | | | 0,00620 |
| CEBPD | Homo sapiens CCAAT/enhancer binding protein (C/EBP), delta (CEBPD), mRNA. | 0,9901 | 9,4080 | | 3,8925 | | 0,003267 | | | 0,01789 |
| ARHGAP21 | Homo sapiens Rho GTPase activating protein 21 (ARHGAP21), mRNA. | 0,9889 | 9,4568 | | 8,2653 | | 0,000012 | | | 0,00087 |
| IFNGR2 | Homo sapiens interferon gamma receptor 2 (interferon gamma transducer 1) (IFNGR2), mRNA. | 0,9888 | 10,4882 | | 6,4363 | | 0,000092 | | | 0,00215 |
| JARID2 | Homo sapiens jumonji, AT rich interactive domain 2 (JARID2), mRNA. | 0,9867 | 9,2078 | | 3,4066 | | 0,007147 | | | 0,03143 |
| LOC731542 | PREDICTED: Homo sapiens misc_RNA (LOC731542), miscRNA. | 0,9861 | 12,2540 | | 5,4203 | | 0,000343 | | | 0,00435 |
| IL17RD | Homo sapiens interleukin 17 receptor D (IL17RD), transcript variant 1, mRNA. | 0,9838 | 8,5059 | | 4,5997 | | 0,001105 | | | 0,00875 |
| IGFBP6 | Homo sapiens insulin-like growth factor binding protein 6 (IGFBP6), mRNA. | 0,9834 | 10,2095 | | 3,7064 | | 0,004395 | | | 0,02201 |
| LOC613037 | Homo sapiens nuclear pore complex interacting protein pseudogene (LOC613037), non-coding RNA. | 0,9828 | 9,7682 | | 6,7207 | | 0,000065 | | | 0,00182 |
| TOM1 | Homo sapiens target of myb1 (chicken) (TOM1), mRNA. | 0,9826 | 8,3868 | | 3,5867 | | 0,005331 | | | 0,02526 |
| TSC22D2 | Homo sapiens TSC22 domain family, member 2 (TSC22D2), mRNA. | 0,9818 | 8,5130 | | 6,5772 | | 0,000077 | | | 0,00198 |
| METRNL | Homo sapiens meteorin, glial cell differentiation regulator-like (METRNL), mRNA. | 0,9817 | 7,7832 | | 8,5360 | | 0,000009 | | | 0,00077 |
| TRIM13 | Homo sapiens tripartite motif-containing 13 (TRIM13), transcript variant 4, mRNA. | 0,9813 | 8,3244 | | 6,1261 | | 0,000135 | | | 0,00263 |
| C21orf55 | Homo sapiens chromosome 21 open reading frame 55 (C21orf55), mRNA. | 0,9811 | 11,3281 | | 3,7967 | | 0,003804 | | | 0,01987 |
| LOC23117 | PREDICTED: Homo sapiens KIAA0220-like protein, transcript variant 16 (LOC23117), mRNA. | 0,9809 | 9,6677 | | 6,0492 | | 0,000149 | | | 0,00277 |
| SLC16A12 | Homo sapiens solute carrier family 16, member 12 (monocarboxylic acid transporter 12) (SLC16A12), mRNA. | 0,9801 | 11,3442 | | 4,5883 | | 0,001123 | | | 0,00883 |
| SWAP70 | Homo sapiens SWAP switching B-cell complex 70kDa subunit (SWAP70), mRNA. | 0,9796 | 9,0592 | | 3,2916 | | 0,008634 | | | 0,03585 |
| FNBP1 | Homo sapiens formin binding protein 1 (FNBP1), mRNA. | 0,9795 | 8,5827 | | 5,3217 | | 0,000393 | | | 0,00471 |
| RNU2-1 | Homo sapiens RNA, U2 small nuclear 1 (RNU2-1), non-coding RNA. | 0,9791 | 7,5352 | | 3,8125 | | 0,003709 | | | 0,01952 |
| KLHL28 | Homo sapiens kelch-like 28 (Drosophila) (KLHL28), mRNA. | 0,9776 | 8,1556 | | 8,2058 | | 0,000013 | | | 0,00089 |
| CYP26B1 | Homo sapiens cytochrome P450, family 26, subfamily B, polypeptide 1 (CYP26B1), mRNA. | 0,9771 | 8,8874 | | 3,4495 | | 0,006663 | | | 0,02983 |
| TMEM154 | Homo sapiens transmembrane protein 154 (TMEM154), mRNA. | 0,9770 | 8,8333 | | 5,9470 | | 0,000170 | | | 0,00300 |
| MAFB | Homo sapiens v-maf musculoaponeurotic fibrosarcoma oncogene homolog B (avian) (MAFB), mRNA. | 0,9757 | 7,7458 | | 3,5727 | | 0,005453 | | | 0,02568 |
| METRNL | PREDICTED: Homo sapiens meteorin, glial cell differentiation regulator-like (METRNL), mRNA. | 0,9750 | 7,8533 | | 6,7011 | | 0,000067 | | | 0,00183 |
| GPR172A | Homo sapiens G protein-coupled receptor 172A (GPR172A), mRNA. | 0,9743 | 11,0961 | | 11,6326 | | 0,000001 | | | 0,00029 |
| BLZF1 | Homo sapiens basic leucine zipper nuclear factor 1 (BLZF1), mRNA. | 0,9734 | 8,1560 | | 7,0498 | | 0,000044 | | | 0,00151 |
| CYTH2 | Homo sapiens cytohesin 2 (CYTH2), transcript variant 1, mRNA. | 0,9731 | 8,5841 | | 5,3262 | | 0,000390 | | | 0,00469 |
| IER5 | Homo sapiens immediate early response 5 (IER5), mRNA. | 0,9728 | 8,8814 | | 3,9859 | | 0,002820 | | | 0,01625 |
| LOC85389 | Homo sapiens RNA, small nucleolar (LOC85389), non-coding RNA. | 0,9721 | 7,8336 | | 9,7555 | | 0,000003 | | | 0,00049 |
| SERINC1 | Homo sapiens serine incorporator 1 (SERINC1), mRNA. | 0,9719 | 8,7210 | | 6,6352 | | 0,000072 | | | 0,00190 |
| LILRB3 | Homo sapiens leukocyte immunoglobulin-like receptor, subfamily B (with TM and ITIM domains), member 3 (LILRB3), transcript variant 2, mRNA. | 0,9702 | 11,9583 | | 4,9192 | | 0,000693 | | | 0,00650 |
| ATF4 | Homo sapiens activating transcription factor 4 (tax-responsive enhancer element B67) (ATF4), transcript variant 1, mRNA. | 0,9686 | 12,5694 | | 6,0075 | | 0,000158 | | | 0,00286 |
| JUP | Homo sapiens junction plakoglobin (JUP), transcript variant 2, mRNA. | 0,9674 | 8,5715 | | 5,3786 | | 0,000363 | | | 0,00447 |
| CCL5 | Homo sapiens chemokine (C-C motif) ligand 5 (CCL5), mRNA. | 0,9665 | 7,7822 | | 7,5638 | | 0,000025 | | | 0,00118 |
| LOC202781 | PREDICTED: Homo sapiens hypothetical protein LOC202781 (LOC202781), mRNA. | 0,9658 | 9,0317 | | 4,3492 | | 0,001609 | | | 0,01112 |
| SEC31A | Homo sapiens SEC31 homolog A (S. cerevisiae) (SEC31A), transcript variant 1, mRNA. | 0,9652 | 9,7088 | | 7,9441 | | 0,000016 | | | 0,00098 |
| SARS | Homo sapiens seryl-tRNA synthetase (SARS), mRNA. | 0,9650 | 9,5294 | | 5,8495 | | 0,000193 | | | 0,00320 |
| TXNRD1 | Homo sapiens thioredoxin reductase 1 (TXNRD1), transcript variant 4, mRNA. | 0,9635 | 9,3709 | | 4,0227 | | 0,002662 | | | 0,01564 |
| CD63 | Homo sapiens CD63 molecule (CD63), transcript variant 2, mRNA. | 0,9627 | 10,0733 | | 3,2852 | | 0,008725 | | | 0,03609 |
| TBC1D15 | Homo sapiens TBC1 domain family, member 15 (TBC1D15), mRNA. | 0,9624 | 8,4848 | | 4,4853 | | 0,001310 | | | 0,00974 |
| SGK | Homo sapiens serum/glucocorticoid regulated kinase (SGK), mRNA. | 0,9606 | 9,4888 | | 3,5141 | | 0,005996 | | | 0,02763 |
| LOC653171 | PREDICTED: Homo sapiens similar to MAPK-interacting and spindle-stabilizing protein (LOC653171), mRNA. | 0,9606 | 8,7625 | | 3,1742 | | 0,010484 | | | 0,04114 |
| TMED5 | Homo sapiens transmembrane emp24 protein transport domain containing 5 (TMED5), mRNA. | 0,9600 | 9,3764 | | 7,9363 | | 0,000017 | | | 0,00098 |
| DSG3 | Homo sapiens desmoglein 3 (pemphigus vulgaris antigen) (DSG3), mRNA. | 0,9585 | 9,0213 | | 5,4939 | | 0,000310 | | | 0,00416 |
| JOSD1 | Homo sapiens Josephin domain containing 1 (JOSD1), mRNA. | 0,9570 | 8,7623 | | 9,0632 | | 0,000005 | | | 0,00061 |
| LOC642947 | Homo sapiens hypothetical protein LOC642947 (LOC642947), mRNA. | 0,9560 | 11,5904 | | 5,2495 | | 0,000434 | | | 0,00498 |
| EZR | Homo sapiens ezrin (EZR), transcript variant 1, mRNA. | 0,9541 | 12,9747 | | 14,0763 | | 0,000000 | | | 0,00012 |
| NBPF10 | Homo sapiens neuroblastoma breakpoint family, member 10 (NBPF10), mRNA. XM_930727 XM_930739 XM_930751 XM_930759 XM_930766 XM_930776 XM_930785 XM_930797 XM_930808 XM_930830 XM_930841 XM_930850 XM_930862 XM_930872 XM_930880 XM_930889 XM_930897 XM_930903 XM_930910 XM_930917 XM_930926 XM_930936 XM_930943 XM_930951 XM_930954 XM_930961 XM_930967 XM_930975 XM_930985 XM_930993 XM_931003 XM_931009 XM_931015 XM_931021 XM_931027 XM_931033 XM_931038 XM_931044 XM_931049 XM_931055 XM_931060 XM_931066 XM_931069 XM_931072 XM_931076 XM_931080 XM_931084 XM_931090 XM_931096 XM_931102 XM_931110 XM_931119 XM_931125 XM_931131 XM_931137 XM_931138 XM_931145 XM_931149 XM_931157 XM_931161 XM_931164 XM_931169 XM_931174 XM_931178 XM_931183 XM_931188 XM_931191 XM_931196 XM_931202 XM_931208 XM_931213 XM_931221 XM_931229 XM_931234 XM_931240 XM_931245 XM_931251 XM_931255 XM_931259 XM_931264 XM_931269 XM_931277 XM_931282 XM_931291 XM_931299 XM_931308 XM_931317 XM_931322 XM_931328 XM_931335 | 0,9525 | 9,0478 | | 4,1470 | | 0,002194 | | | 0,01370 |
| LOC100129362 | PREDICTED: Homo sapiens hypothetical protein LOC100129362 (LOC100129362), mRNA. | 0,9521 | 11,1134 | | 5,0101 | | 0,000608 | | | 0,00606 |
| RNF149 | Homo sapiens ring finger protein 149 (RNF149), mRNA. | 0,9514 | 9,9192 | | 7,1824 | | 0,000038 | | | 0,00141 |
| C21orf58 | Homo sapiens chromosome 21 open reading frame 58 (C21orf58), mRNA. | 0,9488 | 8,8620 | | 4,3928 | | 0,001506 | | | 0,01068 |
| C16orf52 | Homo sapiens chromosome 16 open reading frame 52 (C16orf52), mRNA. | 0,9485 | 8,1913 | | 4,5067 | | 0,001269 | | | 0,00951 |
| STX5 | Homo sapiens syntaxin 5 (STX5), mRNA. | 0,9484 | 9,4947 | | 6,0333 | | 0,000152 | | | 0,00280 |
| LOC100128510 | PREDICTED: Homo sapiens hypothetical protein LOC100128510 (LOC100128510), mRNA. | 0,9478 | 8,2293 | | 4,9536 | | 0,000659 | | | 0,00631 |
| FAM100B | Homo sapiens family with sequence similarity 100, member B (FAM100B), mRNA. | 0,9472 | 8,0761 | | 10,5138 | | 0,000001 | | | 0,00038 |
| FTHL3 | Homo sapiens ferritin, heavy polypeptide-like 3 (FTHL3), non-coding RNA. | 0,9465 | 9,1062 | | 3,7269 | | 0,004253 | | | 0,02152 |
| LRRFIP2 | Homo sapiens leucine rich repeat (in FLII) interacting protein 2 (LRRFIP2), transcript variant 2, mRNA. | 0,9452 | 8,4555 | | 3,7384 | | 0,004176 | | | 0,02128 |
| DTWD2 | Homo sapiens DTW domain containing 2 (DTWD2), mRNA. | 0,9440 | 8,3485 | | 5,1708 | | 0,000485 | | | 0,00533 |
| SOX4 | Homo sapiens SRY (sex determining region Y)-box 4 (SOX4), mRNA. | 0,9440 | 8,6032 | | 3,8714 | | 0,003378 | | | 0,01830 |
| COQ10B | Homo sapiens coenzyme Q10 homolog B (S. cerevisiae) (COQ10B), mRNA. | 0,9433 | 9,8994 | | 4,1736 | | 0,002106 | | | 0,01331 |
| PRKAG2 | Homo sapiens protein kinase, AMP-activated, gamma 2 non-catalytic subunit (PRKAG2), transcript variant c, mRNA. | 0,9422 | 8,5111 | | 5,3634 | | 0,000371 | | | 0,00453 |
| FNTA | Homo sapiens farnesyltransferase, CAAX box, alpha (FNTA), transcript variant 3, mRNA. | 0,9402 | 9,8844 | | 14,7738 | | 0,000000 | | | 0,00012 |
| TOB1 | Homo sapiens transducer of ERBB2, 1 (TOB1), mRNA. | 0,9394 | 8,2780 | | 6,2826 | | 0,000111 | | | 0,00235 |
| FAM73A | Homo sapiens family with sequence similarity 73, member A (FAM73A), mRNA. | 0,9356 | 8,2728 | | 4,9339 | | 0,000678 | | | 0,00641 |
| ITPRIP | Homo sapiens inositol 1,4,5-triphosphate receptor interacting protein (ITPRIP), mRNA. | 0,9338 | 9,2382 | | 5,9889 | | 0,000161 | | | 0,00290 |
| ANXA1 | Homo sapiens annexin A1 (ANXA1), mRNA. | 0,9336 | 12,8233 | | 7,2779 | | 0,000034 | | | 0,00134 |
| TFAP2A | Homo sapiens transcription factor AP-2 alpha (activating enhancer binding protein 2 alpha) (TFAP2A), transcript variant 3, mRNA. | 0,9336 | 9,5751 | | 3,3667 | | 0,007631 | | | 0,03292 |
| CCDC125 | Homo sapiens coiled-coil domain containing 125 (CCDC125), mRNA. | 0,9332 | 8,4488 | | 5,4745 | | 0,000319 | | | 0,00422 |
| C9orf21 | Homo sapiens chromosome 9 open reading frame 21 (C9orf21), mRNA. | 0,9330 | 8,8167 | | 3,5771 | | 0,005414 | | | 0,02556 |
| CSF2RA | PREDICTED: Homo sapiens colony stimulating factor 2 receptor, alpha, low-affinity (granulocyte-macrophage) (CSF2RA), mRNA. | 0,9326 | 8,0587 | | 5,0947 | | 0,000539 | | | 0,00565 |
| SH3GLB1 | Homo sapiens SH3-domain GRB2-like endophilin B1 (SH3GLB1), mRNA. | 0,9308 | 10,2564 | | 8,4278 | | 0,000010 | | | 0,00080 |
| GJB5 | Homo sapiens gap junction protein, beta 5, 31.1kDa (GJB5), mRNA. | 0,9304 | 8,2567 | | 5,0424 | | 0,000581 | | | 0,00590 |
| LOC729768 | PREDICTED: Homo sapiens misc_RNA (LOC729768), miscRNA. | 0,9289 | 11,6726 | | 7,1035 | | 0,000042 | | | 0,00147 |
| RNASE4 | Homo sapiens ribonuclease, RNase A family, 4 (RNASE4), transcript variant 3, mRNA. | 0,9277 | 8,0874 | | 4,0396 | | 0,002593 | | | 0,01540 |
| DUSP10 | Homo sapiens dual specificity phosphatase 10 (DUSP10), transcript variant 3, mRNA. | 0,9274 | 8,1307 | | 4,8497 | | 0,000766 | | | 0,00695 |
| FAM115A | Homo sapiens family with sequence similarity 115, member A (FAM115A), mRNA. | 0,9253 | 12,5245 | | 6,8309 | | 0,000057 | | | 0,00172 |
| SLC39A4 | Homo sapiens solute carrier family 39 (zinc transporter), member 4 (SLC39A4), transcript variant 1, mRNA. | 0,9242 | 9,1221 | | 3,7307 | | 0,004227 | | | 0,02144 |
| PLEKHM1 | PREDICTED: Homo sapiens pleckstrin homology domain containing, family M (with RUN domain) member 1 (PLEKHM1), mRNA. | 0,9241 | 8,0584 | | 3,4660 | | 0,006486 | | | 0,02924 |
| HEBP2 | Homo sapiens heme binding protein 2 (HEBP2), mRNA. | 0,9240 | 10,1787 | | 3,1289 | | 0,011303 | | | 0,04353 |
| C5orf28 | Homo sapiens chromosome 5 open reading frame 28 (C5orf28), mRNA. | 0,9237 | 8,7650 | | 3,1142 | | 0,011583 | | | 0,04432 |
| C3orf34 | Homo sapiens chromosome 3 open reading frame 34 (C3orf34), mRNA. | 0,9231 | 10,8239 | | 7,9900 | | 0,000016 | | | 0,00096 |
| SPAG9 | Homo sapiens sperm associated antigen 9 (SPAG9), mRNA. | 0,9230 | 8,1728 | | 4,9961 | | 0,000620 | | | 0,00612 |
| RABAC1 | Homo sapiens Rab acceptor 1 (prenylated) (RABAC1), mRNA. | 0,9220 | 9,9989 | | 4,2695 | | 0,001817 | | | 0,01205 |
| DYNLT3 | Homo sapiens dynein, light chain, Tctex-type 3 (DYNLT3), mRNA. | 0,9219 | 8,9822 | | 7,3857 | | 0,000030 | | | 0,00127 |
| SYVN1 | Homo sapiens synovial apoptosis inhibitor 1, synoviolin (SYVN1), transcript variant 1, mRNA. | 0,9218 | 8,7227 | | 6,7541 | | 0,000063 | | | 0,00180 |
| LOC100132727 | PREDICTED: Homo sapiens hypothetical protein LOC100132727 (LOC100132727), mRNA. | 0,9217 | 9,2236 | | 5,1066 | | 0,000530 | | | 0,00560 |
| RASSF1 | Homo sapiens Ras association (RalGDS/AF-6) domain family 1 (RASSF1), transcript variant B, mRNA. | 0,9195 | 8,5026 | | 8,1057 | | 0,000014 | | | 0,00093 |
| FTHL11 | Homo sapiens ferritin, heavy polypeptide-like 11 (FTHL11) on chromosome 8. | 0,9192 | 10,2924 | | 3,8845 | | 0,003309 | | | 0,01806 |
| ANXA2P1 | Homo sapiens annexin A2 pseudogene 1 (ANXA2P1) on chromosome 4. | 0,9173 | 9,0042 | | 3,5022 | | 0,006114 | | | 0,02803 |
| S100A13 | Homo sapiens S100 calcium binding protein A13 (S100A13), transcript variant 3, mRNA. | 0,9169 | 10,0381 | | 3,8341 | | 0,003584 | | | 0,01909 |
| PPP4R4 | Homo sapiens protein phosphatase 4, regulatory subunit 4 (PPP4R4), transcript variant 2, mRNA. | 0,9148 | 7,7713 | | 5,3399 | | 0,000383 | | | 0,00462 |
| VEGFA | Homo sapiens vascular endothelial growth factor A (VEGFA), transcript variant 2, mRNA. | 0,9138 | 8,0573 | | 6,9111 | | 0,000052 | | | 0,00165 |
| S100A11 | Homo sapiens S100 calcium binding protein A11 (S100A11), mRNA. | 0,9125 | 11,2715 | | 3,7864 | | 0,003867 | | | 0,02010 |
| STC2 | Homo sapiens stanniocalcin 2 (STC2), mRNA. | 0,9111 | 8,9726 | | 5,2588 | | 0,000429 | | | 0,00496 |
| NET1 | Homo sapiens neuroepithelial cell transforming 1 (NET1), transcript variant 2, mRNA. | 0,9106 | 8,4791 | | 4,7773 | | 0,000851 | | | 0,00744 |
| S100A13 | Homo sapiens S100 calcium binding protein A13 (S100A13), transcript variant 3, mRNA. | 0,9091 | 9,5282 | | 4,2567 | | 0,001853 | | | 0,01220 |
| SLC24A6 | Homo sapiens solute carrier family 24 (sodium/potassium/calcium exchanger), member 6 (SLC24A6), mRNA. | 0,9080 | 8,3772 | | 5,0048 | | 0,000613 | | | 0,00608 |
| CLDND1 | Homo sapiens claudin domain containing 1 (CLDND1), transcript variant 1, mRNA. | 0,9075 | 10,5709 | | 7,9569 | | 0,000016 | | | 0,00097 |
| ELL2 | Homo sapiens elongation factor, RNA polymerase II, 2 (ELL2), mRNA. | 0,9071 | 9,3554 | | 4,8203 | | 0,000799 | | | 0,00714 |
| NFKBIZ | Homo sapiens nuclear factor of kappa light polypeptide gene enhancer in B-cells inhibitor, zeta (NFKBIZ), transcript variant 2, mRNA. | 0,9071 | 7,7761 | | 10,6949 | | 0,000001 | | | 0,00035 |
| BRD2 | Homo sapiens bromodomain containing 2 (BRD2), mRNA. | 0,9042 | 10,2003 | | 4,5055 | | 0,001271 | | | 0,00952 |
| FOSB | Homo sapiens FBJ murine osteosarcoma viral oncogene homolog B (FOSB), mRNA. | 0,9042 | 8,2428 | | 3,9386 | | 0,003038 | | | 0,01701 |
| FTHL11 | Homo sapiens ferritin, heavy polypeptide-like 11 (FTHL11) on chromosome 8. | 0,9036 | 8,7892 | | 3,9351 | | 0,003055 | | | 0,01706 |
| FAM110C | Homo sapiens family with sequence similarity 110, member C (FAM110C), mRNA. | 0,9036 | 7,7938 | | 3,7690 | | 0,003976 | | | 0,02049 |
| C6orf141 | Homo sapiens chromosome 6 open reading frame 141 (C6orf141), mRNA. | 0,9032 | 8,2913 | | 7,3992 | | 0,000030 | | | 0,00126 |
| FAM83B | Homo sapiens family with sequence similarity 83, member B (FAM83B), mRNA. | 0,9030 | 7,8740 | | 4,9507 | | 0,000662 | | | 0,00633 |
| SOCS1 | Homo sapiens suppressor of cytokine signaling 1 (SOCS1), mRNA. | 0,9027 | 7,9374 | | 3,2661 | | 0,009005 | | | 0,03692 |
| NEDD9 | Homo sapiens neural precursor cell expressed, developmentally down-regulated 9 (NEDD9), transcript variant 2, mRNA. | 0,9015 | 7,5414 | | 12,5362 | | 0,000000 | | | 0,00022 |
| CLDND1 | Homo sapiens claudin domain containing 1 (CLDND1), transcript variant 1, mRNA. | 0,9007 | 10,3638 | | 6,4550 | | 0,000090 | | | 0,00213 |
| LRAP | Homo sapiens leukocyte-derived arginine aminopeptidase (LRAP), mRNA. | 0,9002 | 11,8654 | | 5,4193 | | 0,000344 | | | 0,00435 |
| OSBPL2 | Homo sapiens oxysterol binding protein-like 2 (OSBPL2), transcript variant 2, mRNA. | 0,8992 | 8,8475 | | 6,0182 | | 0,000155 | | | 0,00284 |
| ERN1 | Homo sapiens endoplasmic reticulum to nucleus signalling 1 (ERN1), transcript variant 2, mRNA. | 0,8988 | 7,9418 | | 4,8358 | | 0,000781 | | | 0,00705 |
| ITPRIP | Homo sapiens inositol 1,4,5-triphosphate receptor interacting protein (ITPRIP), mRNA. | 0,8979 | 8,9828 | | 7,5088 | | 0,000026 | | | 0,00121 |
| ABL2 | Homo sapiens v-abl Abelson murine leukemia viral oncogene homolog 2 (arg, Abelson-related gene) (ABL2), transcript variant a, mRNA. | 0,8976 | 7,5868 | | 4,7785 | | 0,000849 | | | 0,00744 |
| SERPINB8 | Homo sapiens serpin peptidase inhibitor, clade B (ovalbumin), member 8 (SERPINB8), transcript variant 1, mRNA. | 0,8966 | 8,0334 | | 5,6582 | | 0,000249 | | | 0,00368 |
| LOC727808 | PREDICTED: Homo sapiens hypothetical protein LOC727808 (LOC727808), miscRNA. | 0,8949 | 12,6824 | | 4,1269 | | 0,002263 | | | 0,01397 |
| TNFAIP1 | Homo sapiens tumor necrosis factor, alpha-induced protein 1 (endothelial) (TNFAIP1), mRNA. | 0,8939 | 9,5633 | | 8,7566 | | 0,000007 | | | 0,00069 |
| HK2 | Homo sapiens hexokinase 2 (HK2), mRNA. | 0,8937 | 8,7194 | | 4,2632 | | 0,001835 | | | 0,01211 |
| C4orf34 | Homo sapiens chromosome 4 open reading frame 34 (C4orf34), mRNA. | 0,8931 | 8,3541 | | 4,0027 | | 0,002747 | | | 0,01598 |
| AKIRIN2 | Homo sapiens akirin 2 (AKIRIN2), mRNA. | 0,8911 | 10,2606 | | 4,8077 | | 0,000814 | | | 0,00724 |
| FAM129B | Homo sapiens family with sequence similarity 129, member B (FAM129B), transcript variant 2, mRNA. | 0,8904 | 9,6747 | | 4,4184 | | 0,001449 | | | 0,01041 |
| DSP | Homo sapiens desmoplakin (DSP), transcript variant 2, mRNA. | 0,8900 | 8,9195 | | 3,7727 | | 0,003953 | | | 0,02040 |
| REPS2 | Homo sapiens RALBP1 associated Eps domain containing 2 (REPS2), transcript variant 1, mRNA. | 0,8889 | 7,8923 | | 7,8365 | | 0,000018 | | | 0,00103 |
| UBE2H | Homo sapiens ubiquitin-conjugating enzyme E2H (UBC8 homolog, yeast) (UBE2H), transcript variant 1, mRNA. | 0,8879 | 8,1088 | | 5,4862 | | 0,000314 | | | 0,00418 |
| S100A14 | Homo sapiens S100 calcium binding protein A14 (S100A14), mRNA. | 0,8876 | 8,1345 | | 3,9688 | | 0,002897 | | | 0,01655 |
| LOC400721 | PREDICTED: Homo sapiens similar to Zinc finger protein 418 (LOC400721), mRNA. | 0,8876 | 11,8067 | | 4,5452 | | 0,001198 | | | 0,00919 |
| RBMS1 | Homo sapiens RNA binding motif, single stranded interacting protein 1 (RBMS1), transcript variant 3, mRNA. | 0,8874 | 9,3664 | | 4,3220 | | 0,001677 | | | 0,01143 |
| SNORD12 | Homo sapiens small nucleolar RNA, C/D box 12 (SNORD12), small nucleolar RNA. | 0,8865 | 7,6441 | | 3,5939 | | 0,005268 | | | 0,02505 |
| GOLGB1 | Homo sapiens golgin B1, golgi integral membrane protein (GOLGB1), mRNA. | 0,8864 | 8,5136 | | 5,7187 | | 0,000230 | | | 0,00352 |
| CTNS | Homo sapiens cystinosis, nephropathic (CTNS), transcript variant 2, mRNA. | 0,8864 | 8,5091 | | 3,6378 | | 0,004908 | | | 0,02381 |
| JMJD1C | Homo sapiens jumonji domain containing 1C (JMJD1C), transcript variant 1, mRNA. | 0,8862 | 7,9880 | | 5,0662 | | 0,000562 | | | 0,00579 |
| HBP1 | Homo sapiens HMG-box transcription factor 1 (HBP1), mRNA. | 0,8856 | 8,0124 | | 6,4742 | | 0,000088 | | | 0,00210 |
| SGK1 | Homo sapiens serum/glucocorticoid regulated kinase 1 (SGK1), transcript variant 1, mRNA. | 0,8852 | 9,3002 | | 3,4975 | | 0,006161 | | | 0,02817 |
| CCL3L3 | Homo sapiens chemokine (C-C motif) ligand 3-like 3 (CCL3L3), mRNA. | 0,8850 | 7,5236 | | 3,9543 | | 0,002964 | | | 0,01677 |
| SCYL2 | Homo sapiens SCY1-like 2 (S. cerevisiae) (SCYL2), mRNA. | 0,8845 | 9,0028 | | 4,3499 | | 0,001607 | | | 0,01111 |
| RNF103 | Homo sapiens ring finger protein 103 (RNF103), mRNA. | 0,8833 | 8,7494 | | 3,6635 | | 0,004709 | | | 0,02311 |
| C2orf69 | Homo sapiens chromosome 2 open reading frame 69 (C2orf69), mRNA. | 0,8831 | 9,7921 | | 3,5249 | | 0,005892 | | | 0,02729 |
| SOCS2 | Homo sapiens suppressor of cytokine signaling 2 (SOCS2), mRNA. | 0,8827 | 10,1441 | | 4,5084 | | 0,001266 | | | 0,00950 |
| LOC100131718 | PREDICTED: Homo sapiens misc_RNA (LOC100131718), miscRNA. | 0,8825 | 8,1128 | | 5,5914 | | 0,000272 | | | 0,00386 |
| MTMR3 | Homo sapiens myotubularin related protein 3 (MTMR3), transcript variant 3, mRNA. | 0,8823 | 8,3518 | | 6,2645 | | 0,000114 | | | 0,00239 |
| TESK1 | Homo sapiens testis-specific kinase 1 (TESK1), mRNA. | 0,8822 | 8,4331 | | 8,2752 | | 0,000012 | | | 0,00086 |
| SBF1 | Homo sapiens SET binding factor 1 (SBF1), transcript variant 1, mRNA. | 0,8819 | 8,4560 | | 5,5909 | | 0,000272 | | | 0,00386 |
| LOC400578 | PREDICTED: Homo sapiens similar to Keratin, type I cytoskeletal 14 (Cytokeratin-14) (CK-14) (Keratin-14) (K14) (LOC400578), mRNA. | 0,8817 | 8,2074 | | 3,5950 | | 0,005259 | | | 0,02503 |
| VIM | Homo sapiens vimentin (VIM), mRNA. | 0,8794 | 12,1727 | | 3,3996 | | 0,007230 | | | 0,03164 |
| ZC3H11B | PREDICTED: Homo sapiens misc_RNA (ZC3H11B), miscRNA. | 0,8793 | 9,5201 | | 4,5315 | | 0,001223 | | | 0,00928 |
| SDCBP | Homo sapiens syndecan binding protein (syntenin) (SDCBP), transcript variant 2, mRNA. | 0,8791 | 9,8273 | | 5,5088 | | 0,000304 | | | 0,00410 |
| LHB | Homo sapiens luteinizing hormone beta polypeptide (LHB), mRNA. | 0,8778 | 7,9589 | | 3,9453 | | 0,003006 | | | 0,01691 |
| TPBG | Homo sapiens trophoblast glycoprotein (TPBG), mRNA. | 0,8774 | 8,9528 | | 5,1692 | | 0,000486 | | | 0,00533 |
| LMTK3 | PREDICTED: Homo sapiens lemur tyrosine kinase 3 (LMTK3), mRNA. | 0,8762 | 9,0152 | | 3,8099 | | 0,003725 | | | 0,01957 |
| NRAS | Homo sapiens neuroblastoma RAS viral (v-ras) oncogene homolog (NRAS), mRNA. | 0,8762 | 8,7700 | | 4,2871 | | 0,001769 | | | 0,01186 |
| FOXN2 | Homo sapiens forkhead box N2 (FOXN2), mRNA. | 0,8755 | 8,5702 | | 6,8490 | | 0,000056 | | | 0,00170 |
| LOC653184 | PREDICTED: Homo sapiens similar to hypothetical protein LOC375127, transcript variant 1 (LOC653184), mRNA. | 0,8750 | 8,3916 | | 4,3417 | | 0,001628 | | | 0,01120 |
| ZNF281 | Homo sapiens zinc finger protein 281 (ZNF281), mRNA. | 0,8735 | 8,7690 | | 10,1359 | | 0,000002 | | | 0,00042 |
| RND3 | Homo sapiens Rho family GTPase 3 (RND3), mRNA. | 0,8732 | 9,9013 | | 7,4370 | | 0,000029 | | | 0,00124 |
| FAM20C | Homo sapiens family with sequence similarity 20, member C (FAM20C), mRNA. | 0,8725 | 8,5827 | | 3,0802 | | 0,012257 | | | 0,04631 |
| LOC100133649 | PREDICTED: Homo sapiens hypothetical protein LOC100133649 (LOC100133649), mRNA. | 0,8725 | 12,1571 | | 4,3026 | | 0,001727 | | | 0,01168 |
| CCNDBP1 | Homo sapiens cyclin D-type binding-protein 1 (CCNDBP1), transcript variant 2, mRNA. | 0,8725 | 10,2290 | | 4,2606 | | 0,001842 | | | 0,01215 |
| RIT1 | Homo sapiens Ras-like without CAAX 1 (RIT1), mRNA. | 0,8723 | 8,3528 | | 3,5300 | | 0,005844 | | | 0,02712 |
| LOC100133772 | PREDICTED: Homo sapiens similar to MCT (LOC100133772), mRNA. | 0,8720 | 9,6969 | | 4,6962 | | 0,000958 | | | 0,00805 |
| SNORA8 | Homo sapiens small nucleolar RNA, H/ACA box 8 (SNORA8), small nucleolar RNA. | 0,8718 | 8,0360 | | 6,3467 | | 0,000103 | | | 0,00226 |
| IRF2BP2 | Homo sapiens interferon regulatory factor 2 binding protein 2 (IRF2BP2), transcript variant 1, mRNA. | 0,8718 | 8,4809 | | 6,7540 | | 0,000063 | | | 0,00180 |
| M6PRBP1 | Homo sapiens mannose-6-phosphate receptor binding protein 1 (M6PRBP1), mRNA. | 0,8710 | 11,1001 | | 3,8269 | | 0,003626 | | | 0,01921 |
| PLEKHM2 | Homo sapiens pleckstrin homology domain containing, family M (with RUN domain) member 2 (PLEKHM2), mRNA. | 0,8710 | 9,6076 | | 6,3960 | | 0,000097 | | | 0,00219 |
| GOLGA7B | Homo sapiens golgi autoantigen, golgin subfamily a, 7B (GOLGA7B), mRNA. | 0,8706 | 8,0870 | | 4,4865 | | 0,001308 | | | 0,00973 |
| KIAA0430 | Homo sapiens KIAA0430 (KIAA0430), mRNA. | 0,8691 | 8,5565 | | 5,2380 | | 0,000441 | | | 0,00503 |
| ETNK1 | Homo sapiens ethanolamine kinase 1 (ETNK1), transcript variant 2, mRNA. | 0,8678 | 8,0974 | | 3,1097 | | 0,011670 | | | 0,04454 |
| IL32 | Homo sapiens interleukin 32 (IL32), transcript variant 4, mRNA. | 0,8672 | 7,8167 | | 8,0924 | | 0,000014 | | | 0,00093 |
| TMEM120A | Homo sapiens transmembrane protein 120A (TMEM120A), mRNA. | 0,8671 | 8,3591 | | 4,0106 | | 0,002713 | | | 0,01586 |
| C15orf39 | Homo sapiens chromosome 15 open reading frame 39 (C15orf39), mRNA. | 0,8669 | 8,0558 | | 4,6725 | | 0,000992 | | | 0,00821 |
| STARD10 | Homo sapiens StAR-related lipid transfer (START) domain containing 10 (STARD10), mRNA. | 0,8646 | 9,0430 | | 5,4239 | | 0,000341 | | | 0,00434 |
| ZAK | Homo sapiens sterile alpha motif and leucine zipper containing kinase AZK (ZAK), transcript variant 2, mRNA. | 0,8635 | 9,7103 | | 4,1474 | | 0,002193 | | | 0,01370 |
| SPCS3 | Homo sapiens signal peptidase complex subunit 3 homolog (S. cerevisiae) (SPCS3), mRNA. | 0,8635 | 9,2056 | | 6,6945 | | 0,000067 | | | 0,00184 |
| SEC31A | Homo sapiens SEC31 homolog A (S. cerevisiae) (SEC31A), transcript variant 1, mRNA. | 0,8632 | 8,8646 | | 6,5757 | | 0,000078 | | | 0,00198 |
| KHNYN | Homo sapiens KH and NYN domain containing (KHNYN), mRNA. | 0,8631 | 8,2136 | | 4,4697 | | 0,001341 | | | 0,00989 |
| CDA | Homo sapiens cytidine deaminase (CDA), mRNA. | 0,8625 | 8,0039 | | 4,8053 | | 0,000817 | | | 0,00724 |
| FTHL2 | Homo sapiens ferritin, heavy polypeptide-like 2 (FTHL2) on chromosome 1. | 0,8619 | 10,4836 | | 3,3139 | | 0,008322 | | | 0,03495 |
| CDCP1 | Homo sapiens CUB domain containing protein 1 (CDCP1), transcript variant 2, mRNA. | 0,8611 | 7,6608 | | 3,2029 | | 0,009997 | | | 0,03978 |
| FTHL12 | Homo sapiens ferritin, heavy polypeptide-like 12 (FTHL12) on chromosome 9. | 0,8606 | 11,0862 | | 3,1861 | | 0,010279 | | | 0,04054 |
| CEBPG | Homo sapiens CCAAT/enhancer binding protein (C/EBP), gamma (CEBPG), mRNA. | 0,8605 | 9,8292 | | 4,8266 | | 0,000792 | | | 0,00710 |
| EFTUD1 | Homo sapiens elongation factor Tu GTP binding domain containing 1 (EFTUD1), transcript variant 1, mRNA. | 0,8600 | 8,1848 | | 6,2364 | | 0,000118 | | | 0,00243 |
| LOC201175 | Homo sapiens hypothetical protein LOC201175 (LOC201175), mRNA. | 0,8595 | 7,7940 | | 4,6417 | | 0,001038 | | | 0,00841 |
| LOC650803 | PREDICTED: Homo sapiens hypothetical protein LOC650803 (LOC650803), mRNA. | 0,8591 | 8,4303 | | 6,5484 | | 0,000080 | | | 0,00200 |
| UBE2D3 | Homo sapiens ubiquitin-conjugating enzyme E2D 3 (UBC4/5 homolog, yeast) (UBE2D3), transcript variant 6, mRNA. | 0,8580 | 11,7157 | | 3,3416 | | 0,007952 | | | 0,03384 |
| GOLGA8B | Homo sapiens golgi autoantigen, golgin subfamily a, 8B (GOLGA8B), mRNA. | 0,8569 | 8,7072 | | 3,4610 | | 0,006539 | | | 0,02944 |
| RNY1 | Homo sapiens RNA, Ro-associated Y1 (RNY1), small cytoplasmic RNA. | 0,8566 | 9,4267 | | 4,0966 | | 0,002372 | | | 0,01447 |
| CORO1B | Homo sapiens coronin, actin binding protein, 1B (CORO1B), transcript variant 2, mRNA. | 0,8556 | 9,6289 | | 3,6854 | | 0,004546 | | | 0,02257 |
| CRB3 | Homo sapiens crumbs homolog 3 (Drosophila) (CRB3), transcript variant 2, mRNA. | 0,8549 | 8,1924 | | 8,4916 | | 0,000009 | | | 0,00078 |
| ITGA6 | Homo sapiens integrin, alpha 6 (ITGA6), transcript variant 2, mRNA. | 0,8545 | 8,6972 | | 4,4850 | | 0,001311 | | | 0,00974 |
| ZNF581 | Homo sapiens zinc finger protein 581 (ZNF581), mRNA. | 0,8537 | 9,2455 | | 7,1438 | | 0,000040 | | | 0,00144 |
| CBL | Homo sapiens Cas-Br-M (murine) ecotropic retroviral transforming sequence (CBL), mRNA. | 0,8537 | 8,5356 | | 7,7609 | | 0,000020 | | | 0,00107 |
| MFSD6 | Homo sapiens major facilitator superfamily domain containing 6 (MFSD6), mRNA. | 0,8536 | 8,4831 | | 6,7088 | | 0,000066 | | | 0,00183 |
| IL23A | Homo sapiens interleukin 23, alpha subunit p19 (IL23A), mRNA. | 0,8521 | 7,6895 | | 3,9666 | | 0,002907 | | | 0,01659 |
| TPM4 | Homo sapiens tropomyosin 4 (TPM4), mRNA. | 0,8521 | 9,6715 | | 7,5476 | | 0,000025 | | | 0,00119 |
| LIPG | Homo sapiens lipase, endothelial (LIPG), mRNA. | 0,8520 | 8,1242 | | 6,1039 | | 0,000139 | | | 0,00268 |
| NCF2 | Homo sapiens neutrophil cytosolic factor 2 (65kDa, chronic granulomatous disease, autosomal 2) (NCF2), mRNA. | 0,8516 | 7,6704 | | 4,7181 | | 0,000928 | | | 0,00790 |
| ST3GAL1 | Homo sapiens ST3 beta-galactoside alpha-2,3-sialyltransferase 1 (ST3GAL1), transcript variant 1, mRNA. | 0,8510 | 8,4992 | | 3,7837 | | 0,003884 | | | 0,02016 |
| EGFR | Homo sapiens epidermal growth factor receptor (erythroblastic leukemia viral (v-erb-b) oncogene homolog, avian) (EGFR), transcript variant 4, mRNA. | 0,8503 | 8,0419 | | 4,1162 | | 0,002301 | | | 0,01412 |
| CBX4 | Homo sapiens chromobox homolog 4 (Pc class homolog, Drosophila) (CBX4), mRNA. | 0,8488 | 8,6721 | | 5,3555 | | 0,000375 | | | 0,00456 |
| SULT1A1 | Homo sapiens sulfotransferase family, cytosolic, 1A, phenol-preferring, member 1 (SULT1A1), transcript variant 3, mRNA. | 0,8488 | 8,9781 | | 4,2910 | | 0,001758 | | | 0,01181 |
| MICAL1 | Homo sapiens microtubule associated monoxygenase, calponin and LIM domain containing 1 (MICAL1), mRNA. | 0,8481 | 8,0250 | | 6,0981 | | 0,000140 | | | 0,00269 |
| BSDC1 | Homo sapiens BSD domain containing 1 (BSDC1), mRNA. | 0,8480 | 9,6180 | | 5,9934 | | 0,000160 | | | 0,00290 |
| DDEF2 | Homo sapiens development and differentiation enhancing factor 2 (DDEF2), mRNA. | 0,8480 | 9,7326 | | 5,8845 | | 0,000185 | | | 0,00312 |
| SYF2 | Homo sapiens SYF2 homolog, RNA splicing factor (S. cerevisiae) (SYF2), transcript variant 1, mRNA. | 0,8465 | 9,8811 | | 3,2525 | | 0,009210 | | | 0,03745 |
| WSB1 | Homo sapiens WD repeat and SOCS box-containing 1 (WSB1), transcript variant 2, mRNA. | 0,8463 | 8,2522 | | 5,6617 | | 0,000248 | | | 0,00367 |
| ANGPTL4 | Homo sapiens angiopoietin-like 4 (ANGPTL4), transcript variant 3, mRNA. | 0,8455 | 8,0926 | | 3,0843 | | 0,012175 | | | 0,04608 |
| PI4KAP1 | Homo sapiens phosphatidylinositol 4-kinase, catalytic, alpha pseudogene 1 (PI4KAP1), non-coding RNA. | 0,8453 | 8,4642 | | 8,7542 | | 0,000007 | | | 0,00069 |
| RELB | Homo sapiens v-rel reticuloendotheliosis viral oncogene homolog B (RELB), mRNA. | 0,8446 | 8,0693 | | 10,3302 | | 0,000002 | | | 0,00041 |
| FERMT2 | Homo sapiens fermitin family homolog 2 (Drosophila) (FERMT2), mRNA. | 0,8443 | 8,6243 | | 6,2529 | | 0,000115 | | | 0,00241 |
| VPS28 | Homo sapiens vacuolar protein sorting 28 homolog (S. cerevisiae) (VPS28), transcript variant 1, mRNA. | 0,8431 | 9,7692 | | 3,7807 | | 0,003903 | | | 0,02022 |
| MAPK6 | Homo sapiens mitogen-activated protein kinase 6 (MAPK6), mRNA. | 0,8429 | 9,8453 | | 5,2669 | | 0,000424 | | | 0,00494 |
| AHNAK | Homo sapiens AHNAK nucleoprotein (AHNAK), transcript variant 2, mRNA. | 0,8426 | 8,8238 | | 3,4247 | | 0,006938 | | | 0,03074 |
| LOC401076 | PREDICTED: Homo sapiens misc_RNA (LOC401076), miscRNA. | 0,8426 | 10,0458 | | 5,0530 | | 0,000572 | | | 0,00584 |
| C19orf10 | Homo sapiens chromosome 19 open reading frame 10 (C19orf10), mRNA. | 0,8423 | 9,9664 | | 4,1812 | | 0,002081 | | | 0,01321 |
| AGPAT9 | Homo sapiens 1-acylglycerol-3-phosphate O-acyltransferase 9 (AGPAT9), mRNA. | 0,8410 | 7,9778 | | 3,2607 | | 0,009085 | | | 0,03711 |
| SPTAN1 | Homo sapiens spectrin, alpha, non-erythrocytic 1 (alpha-fodrin) (SPTAN1), mRNA. | 0,8402 | 8,6166 | | 5,1697 | | 0,000485 | | | 0,00533 |
| LAMB3 | Homo sapiens laminin, beta 3 (LAMB3), transcript variant 1, mRNA. | 0,8402 | 7,6665 | | 3,9531 | | 0,002969 | | | 0,01678 |
| RGS12 | Homo sapiens regulator of G-protein signaling 12 (RGS12), transcript variant 2, mRNA. | 0,8401 | 9,0535 | | 3,5941 | | 0,005267 | | | 0,02504 |
| BCL3 | Homo sapiens B-cell CLL/lymphoma 3 (BCL3), mRNA. | 0,8387 | 8,2677 | | 4,9441 | | 0,000668 | | | 0,00634 |
| ACBD3 | Homo sapiens acyl-Coenzyme A binding domain containing 3 (ACBD3), mRNA. | 0,8386 | 9,8079 | | 5,8837 | | 0,000185 | | | 0,00312 |
| C19orf22 | Homo sapiens chromosome 19 open reading frame 22 (C19orf22), mRNA. | 0,8376 | 10,4680 | | 6,8712 | | 0,000054 | | | 0,00169 |
| GDI1 | Homo sapiens GDP dissociation inhibitor 1 (GDI1), mRNA. | 0,8356 | 9,4570 | | 8,0244 | | 0,000015 | | | 0,00095 |
| SLC35D2 | Homo sapiens solute carrier family 35, member D2 (SLC35D2), mRNA. | 0,8345 | 8,2296 | | 5,5311 | | 0,000295 | | | 0,00404 |
| PPARD | Homo sapiens peroxisome proliferative activated receptor, delta (PPARD), transcript variant 1, mRNA. | 0,8341 | 8,0984 | | 4,1162 | | 0,002301 | | | 0,01412 |
| LOC729324 | PREDICTED: Homo sapiens misc_RNA (LOC729324), miscRNA. | 0,8339 | 12,7493 | | 4,0427 | | 0,002580 | | | 0,01535 |
| CTSL1 | Homo sapiens cathepsin L1 (CTSL1), transcript variant 1, mRNA. | 0,8324 | 10,1852 | | 3,4259 | | 0,006925 | | | 0,03071 |
| RIPK2 | Homo sapiens receptor-interacting serine-threonine kinase 2 (RIPK2), mRNA. | 0,8324 | 9,0560 | | 5,0826 | | 0,000549 | | | 0,00571 |
| RAPGEF5 | Homo sapiens Rap guanine nucleotide exchange factor (GEF) 5 (RAPGEF5), mRNA. | 0,8320 | 8,4533 | | 4,0373 | | 0,002602 | | | 0,01543 |
| FRMD6 | Homo sapiens FERM domain containing 6 (FRMD6), transcript variant 2, mRNA. | 0,8299 | 8,9638 | | 4,6967 | | 0,000957 | | | 0,00804 |
| YIPF4 | Homo sapiens Yip1 domain family, member 4 (YIPF4), mRNA. | 0,8294 | 9,3000 | | 5,7458 | | 0,000222 | | | 0,00345 |
| ZSWIM4 | Homo sapiens zinc finger, SWIM-type containing 4 (ZSWIM4), mRNA. | 0,8290 | 8,8678 | | 4,9774 | | 0,000637 | | | 0,00620 |
| LOC100130009 | PREDICTED: Homo sapiens similar to high mobility group protein (LOC100130009), mRNA. | 0,8279 | 8,2139 | | 3,4015 | | 0,007207 | | | 0,03158 |
| CDK2AP2 | Homo sapiens cyclin-dependent kinase 2 associated protein 2 (CDK2AP2), mRNA. | 0,8272 | 9,0753 | | 5,4284 | | 0,000339 | | | 0,00434 |
| ACADVL | Homo sapiens acyl-Coenzyme A dehydrogenase, very long chain (ACADVL), nuclear gene encoding mitochondrial protein, transcript variant 2, mRNA. | 0,8260 | 11,0546 | | 5,4521 | | 0,000329 | | | 0,00428 |
| ATG2A | Homo sapiens ATG2 autophagy related 2 homolog A (S. cerevisiae) (ATG2A), mRNA. | 0,8256 | 7,8575 | | 5,2726 | | 0,000421 | | | 0,00492 |
| LPP | Homo sapiens LIM domain containing preferred translocation partner in lipoma (LPP), mRNA. | 0,8235 | 10,3810 | | 6,8633 | | 0,000055 | | | 0,00169 |
| CCL3 | Homo sapiens chemokine (C-C motif) ligand 3 (CCL3), mRNA. | 0,8222 | 7,5283 | | 3,3759 | | 0,007516 | | | 0,03254 |
| FARSLB | Homo sapiens phenylalanine-tRNA synthetase-like, beta subunit (FARSLB), mRNA. | 0,8200 | 12,7186 | | 4,2636 | | 0,001834 | | | 0,01211 |
| CYP27B1 | Homo sapiens cytochrome P450, family 27, subfamily B, polypeptide 1 (CYP27B1), nuclear gene encoding mitochondrial protein, mRNA. | 0,8198 | 7,8952 | | 4,2647 | | 0,001831 | | | 0,01209 |
| ITGB5 | Homo sapiens integrin, beta 5 (ITGB5), mRNA. XM_944688 XM_944693 | 0,8192 | 9,9526 | | 4,1510 | | 0,002180 | | | 0,01365 |
| CCDC50 | Homo sapiens coiled-coil domain containing 50 (CCDC50), transcript variant 1, mRNA. | 0,8178 | 10,7518 | | 9,7924 | | 0,000003 | | | 0,00048 |
| NRBF2 | Homo sapiens nuclear receptor binding factor 2 (NRBF2), mRNA. | 0,8173 | 8,1774 | | 6,7517 | | 0,000063 | | | 0,00180 |
| NUMB | Homo sapiens numb homolog (Drosophila) (NUMB), transcript variant 2, mRNA. | 0,8163 | 8,8129 | | 5,5215 | | 0,000299 | | | 0,00407 |
| OSBP | Homo sapiens oxysterol binding protein (OSBP), mRNA. | 0,8162 | 9,7086 | | 6,2951 | | 0,000109 | | | 0,00233 |
| SFRS17A | Homo sapiens splicing factor, arginine/serine-rich 17A (SFRS17A), transcript variant 1, mRNA. | 0,8151 | 8,0983 | | 7,5506 | | 0,000025 | | | 0,00119 |
| TMEM154 | Homo sapiens transmembrane protein 154 (TMEM154), mRNA. | 0,8150 | 8,4608 | | 5,1624 | | 0,000490 | | | 0,00537 |
| PECR | Homo sapiens peroxisomal trans-2-enoyl-CoA reductase (PECR), mRNA. | 0,8148 | 8,1923 | | 4,2433 | | 0,001892 | | | 0,01237 |
| RAB32 | Homo sapiens RAB32, member RAS oncogene family (RAB32), mRNA. | 0,8146 | 10,2845 | | 3,7092 | | 0,004376 | | | 0,02195 |
| RELA | Homo sapiens v-rel reticuloendotheliosis viral oncogene homolog A (avian) (RELA), mRNA. | 0,8141 | 8,5270 | | 5,3801 | | 0,000363 | | | 0,00447 |
| ARFGAP1 | Homo sapiens ADP-ribosylation factor GTPase activating protein 1 (ARFGAP1), transcript variant 1, mRNA. | 0,8139 | 9,1608 | | 6,0290 | | 0,000153 | | | 0,00281 |
| SLC31A2 | Homo sapiens solute carrier family 31 (copper transporters), member 2 (SLC31A2), mRNA. | 0,8135 | 10,3174 | | 8,9821 | | 0,000006 | | | 0,00064 |
| RILPL1 | Homo sapiens Rab interacting lysosomal protein-like 1 (RILPL1), mRNA. | 0,8127 | 9,4099 | | 3,6599 | | 0,004736 | | | 0,02319 |
| RALGAPB | Homo sapiens Ral GTPase activating protein, beta subunit (non-catalytic) (RALGAPB), mRNA. | 0,8124 | 8,2150 | | 4,3951 | | 0,001501 | | | 0,01065 |
| IFIT2 | Homo sapiens interferon-induced protein with tetratricopeptide repeats 2 (IFIT2), mRNA. | 0,8119 | 7,8386 | | 3,5253 | | 0,005889 | | | 0,02728 |
| SPTLC1 | Homo sapiens serine palmitoyltransferase, long chain base subunit 1 (SPTLC1), transcript variant 2, mRNA. | 0,8117 | 9,8460 | | 4,4528 | | 0,001376 | | | 0,01006 |
| NRBP2 | Homo sapiens nuclear receptor binding protein 2 (NRBP2), mRNA. | 0,8117 | 8,4972 | | 3,4948 | | 0,006189 | | | 0,02826 |
| DBN1 | Homo sapiens drebrin 1 (DBN1), transcript variant 1, mRNA. | 0,8111 | 8,9569 | | 6,8808 | | 0,000054 | | | 0,00168 |
| MCL1 | Homo sapiens myeloid cell leukemia sequence 1 (BCL2-related) (MCL1), transcript variant 1, mRNA. | 0,8109 | 8,5527 | | 3,3181 | | 0,008265 | | | 0,03478 |
| CCDC93 | Homo sapiens coiled-coil domain containing 93 (CCDC93), mRNA. | 0,8098 | 8,5996 | | 5,9008 | | 0,000181 | | | 0,00308 |
| GARS | Homo sapiens glycyl-tRNA synthetase (GARS), mRNA. | 0,8086 | 12,3524 | | 4,7648 | | 0,000866 | | | 0,00756 |
| PAWR | Homo sapiens PRKC, apoptosis, WT1, regulator (PAWR), mRNA. | 0,8085 | 8,7702 | | 6,6948 | | 0,000067 | | | 0,00184 |
| MIDN | Homo sapiens midnolin (MIDN), mRNA. | 0,8085 | 9,2624 | | 4,3735 | | 0,001551 | | | 0,01086 |
| LOC643509 | PREDICTED: Homo sapiens similar to Dihydrofolate reductase, transcript variant 1 (LOC643509), mRNA. | 0,8083 | 12,4723 | | 4,7498 | | 0,000886 | | | 0,00766 |
| UAP1 | Homo sapiens UDP-N-acteylglucosamine pyrophosphorylase 1 (UAP1), mRNA. | 0,8076 | 9,1731 | | 6,1607 | | 0,000130 | | | 0,00255 |
| ZAK | Homo sapiens sterile alpha motif and leucine zipper containing kinase AZK (ZAK), transcript variant 1, mRNA. | 0,8074 | 8,1145 | | 3,9668 | | 0,002906 | | | 0,01658 |
| MYO10 | Homo sapiens myosin X (MYO10), mRNA. | 0,8066 | 9,0837 | | 4,5117 | | 0,001259 | | | 0,00947 |
| TNFAIP8 | Homo sapiens tumor necrosis factor, alpha-induced protein 8 (TNFAIP8), transcript variant 2, mRNA. | 0,8060 | 8,1387 | | 4,3218 | | 0,001677 | | | 0,01143 |
| CGGBP1 | Homo sapiens CGG triplet repeat binding protein 1 (CGGBP1), transcript variant 2, mRNA. | 0,8050 | 8,7349 | | 3,4487 | | 0,006671 | | | 0,02985 |
| TMF1 | Homo sapiens TATA element modulatory factor 1 (TMF1), mRNA. | 0,8045 | 7,9659 | | 4,4776 | | 0,001325 | | | 0,00982 |
| H1FX | Homo sapiens H1 histone family, member X (H1FX), mRNA. | 0,8043 | 9,4611 | | 3,1029 | | 0,011803 | | | 0,04499 |
| DOCK6 | Homo sapiens dedicator of cytokinesis 6 (DOCK6), mRNA. | 0,8043 | 8,1067 | | 5,4927 | | 0,000311 | | | 0,00416 |
| BNIP3L | Homo sapiens BCL2/adenovirus E1B 19kDa interacting protein 3-like (BNIP3L), mRNA. | 0,8042 | 8,9969 | | 4,1702 | | 0,002117 | | | 0,01337 |
| GPR1 | Homo sapiens G protein-coupled receptor 1 (GPR1), mRNA. | 0,8030 | 8,2030 | | 4,3435 | | 0,001623 | | | 0,01119 |
| LARP6 | Homo sapiens La ribonucleoprotein domain family, member 6 (LARP6), transcript variant 2, mRNA. | 0,8027 | 8,6131 | | 4,2214 | | 0,001956 | | | 0,01268 |
| F11R | Homo sapiens F11 receptor (F11R), transcript variant 5, mRNA. | 0,8018 | 9,2281 | | 7,4754 | | 0,000027 | | | 0,00121 |
| C2orf24 | Homo sapiens chromosome 2 open reading frame 24 (C2orf24), mRNA. | 0,8016 | 8,5365 | | 5,3509 | | 0,000377 | | | 0,00457 |
| TAX1BP1 | Homo sapiens Tax1 (human T-cell leukemia virus type I) binding protein 1 (TAX1BP1), transcript variant 2, mRNA. | 0,8011 | 11,1138 | | 5,5444 | | 0,000290 | | | 0,00400 |
| NRF1 | Homo sapiens nuclear respiratory factor 1 (NRF1), transcript variant 2, mRNA. | 0,8004 | 7,5652 | | 4,5752 | | 0,001146 | | | 0,00896 |
| FMNL2 | Homo sapiens formin-like 2 (FMNL2), mRNA. | 0,7998 | 8,4656 | | 5,7985 | | 0,000207 | | | 0,00333 |
| CYP4F11 | Homo sapiens cytochrome P450, family 4, subfamily F, polypeptide 11 (CYP4F11), mRNA. | 0,7997 | 7,8663 | | 4,0540 | | 0,002535 | | | 0,01518 |
| IQGAP1 | Homo sapiens IQ motif containing GTPase activating protein 1 (IQGAP1), mRNA. | 0,7996 | 8,9484 | | 5,7046 | | 0,000234 | | | 0,00356 |
| TGIF1 | Homo sapiens TGFB-induced factor homeobox 1 (TGIF1), transcript variant 1, mRNA. | 0,7995 | 9,6314 | | 4,3110 | | 0,001705 | | | 0,01156 |
| CTSL1 | Homo sapiens cathepsin L1 (CTSL1), transcript variant 2, mRNA. | 0,7994 | 10,4633 | | 3,0986 | | 0,011887 | | | 0,04519 |
| BCL2L2 | Homo sapiens BCL2-like 2 (BCL2L2), mRNA. | 0,7982 | 9,9013 | | 4,8478 | | 0,000768 | | | 0,00696 |
| LHFPL2 | Homo sapiens lipoma HMGIC fusion partner-like 2 (LHFPL2), mRNA. | 0,7981 | 8,1510 | | 5,1641 | | 0,000489 | | | 0,00537 |
| TPD52L2 | Homo sapiens tumor protein D52-like 2 (TPD52L2), transcript variant 5, mRNA. | 0,7980 | 10,6798 | | 7,2224 | | 0,000036 | | | 0,00138 |
| AFAP1L1 | Homo sapiens actin filament associated protein 1-like 1 (AFAP1L1), mRNA. | 0,7977 | 7,8319 | | 3,6943 | | 0,004481 | | | 0,02235 |
| BCAR1 | Homo sapiens breast cancer anti-estrogen resistance 1 (BCAR1), mRNA. | 0,7977 | 8,7408 | | 4,0004 | | 0,002757 | | | 0,01601 |
| MTSS1 | Homo sapiens metastasis suppressor 1 (MTSS1), mRNA. | 0,7962 | 8,3942 | | 4,6072 | | 0,001092 | | | 0,00870 |
| MGEA5 | Homo sapiens meningioma expressed antigen 5 (hyaluronidase) (MGEA5), mRNA. | 0,7938 | 10,2191 | | 5,0464 | | 0,000578 | | | 0,00587 |
| LOC654103 | PREDICTED: Homo sapiens similar to solute carrier family 25, member 37 (LOC654103), mRNA. | 0,7931 | 8,9399 | | 7,1593 | | 0,000039 | | | 0,00142 |
| CREB1 | Homo sapiens cAMP responsive element binding protein 1 (CREB1), transcript variant A, mRNA. | 0,7925 | 10,2054 | | 13,0048 | | 0,000000 | | | 0,00018 |
| PTPRE | Homo sapiens protein tyrosine phosphatase, receptor type, E (PTPRE), transcript variant 2, mRNA. | 0,7924 | 8,1525 | | 6,5628 | | 0,000079 | | | 0,00199 |
| SMG1 | Homo sapiens PI-3-kinase-related kinase SMG-1 (SMG1), mRNA. | 0,7924 | 8,3296 | | 5,0572 | | 0,000569 | | | 0,00583 |
| FLJ25363 | PREDICTED: Homo sapiens similar to hypothetical protein FLJ25976 (FLJ25363), mRNA. | 0,7916 | 7,8832 | | 5,2784 | | 0,000417 | | | 0,00489 |
| CDR2 | Homo sapiens cerebellar degeneration-related protein 2, 62kDa (CDR2), mRNA. | 0,7915 | 8,8809 | | 18,3883 | | 0,000000 | | | 0,00007 |
| FOXA2 | Homo sapiens forkhead box A2 (FOXA2), transcript variant 2, mRNA. | 0,7915 | 8,1282 | | 6,2156 | | 0,000121 | | | 0,00245 |
| BRPF3 | Homo sapiens bromodomain and PHD finger containing, 3 (BRPF3), mRNA. | 0,7914 | 8,0889 | | 3,7574 | | 0,004050 | | | 0,02080 |
| SULT2B1 | Homo sapiens sulfotransferase family, cytosolic, 2B, member 1 (SULT2B1), transcript variant 1, mRNA. | 0,7899 | 7,5824 | | 3,2295 | | 0,009567 | | | 0,03847 |
| HK1 | Homo sapiens hexokinase 1 (HK1), nuclear gene encoding mitochondrial protein, transcript variant 5, mRNA. | 0,7898 | 11,5403 | | 6,6115 | | 0,000074 | | | 0,00192 |
| RPS29 | Homo sapiens ribosomal protein S29 (RPS29), transcript variant 2, mRNA. | 0,7896 | 7,9282 | | 7,8112 | | 0,000019 | | | 0,00104 |
| DGKQ | Homo sapiens diacylglycerol kinase, theta 110kDa (DGKQ), mRNA. | 0,7893 | 8,4931 | | 7,0193 | | 0,000046 | | | 0,00153 |
| CAB39 | Homo sapiens calcium binding protein 39 (CAB39), mRNA. | 0,7891 | 11,5279 | | 5,1256 | | 0,000516 | | | 0,00551 |
| ZNF598 | Homo sapiens zinc finger protein 598 (ZNF598), mRNA. | 0,7883 | 8,4397 | | 3,7981 | | 0,003796 | | | 0,01983 |
| FAM91A1 | Homo sapiens family with sequence similarity 91, member A1 (FAM91A1), mRNA. | 0,7856 | 8,3050 | | 3,3387 | | 0,007990 | | | 0,03394 |
| MED10 | Homo sapiens mediator complex subunit 10 (MED10), mRNA. | 0,7854 | 10,2374 | | 4,1753 | | 0,002100 | | | 0,01330 |
| ANKRD30B | Homo sapiens ankyrin repeat domain 30B (ANKRD30B), mRNA. | 0,7852 | 9,7838 | | 4,9257 | | 0,000686 | | | 0,00647 |
| FAM177A1 | Homo sapiens family with sequence similarity 177, member A1 (FAM177A1), transcript variant 1, mRNA. | 0,7848 | 8,2903 | | 4,7739 | | 0,000855 | | | 0,00748 |
| UFM1 | Homo sapiens ubiquitin-fold modifier 1 (UFM1), mRNA. | 0,7845 | 9,2763 | | 5,1329 | | 0,000511 | | | 0,00548 |
| SLMO1 | Homo sapiens slowmo homolog 1 (Drosophila) (SLMO1), mRNA. | 0,7837 | 8,2612 | | 7,0496 | | 0,000044 | | | 0,00151 |
| GK | Homo sapiens glycerol kinase (GK), transcript variant 1, mRNA. | 0,7837 | 7,8282 | | 5,2624 | | 0,000426 | | | 0,00495 |
| PHLDB2 | Homo sapiens pleckstrin homology-like domain, family B, member 2 (PHLDB2), mRNA. | 0,7833 | 8,5770 | | 3,6703 | | 0,004658 | | | 0,02293 |
| RB1CC1 | Homo sapiens RB1-inducible coiled-coil 1 (RB1CC1), transcript variant 2, mRNA. | 0,7824 | 8,8484 | | 4,8977 | | 0,000714 | | | 0,00663 |
| TMEM97 | Homo sapiens transmembrane protein 97 (TMEM97), mRNA. | 0,7824 | 8,7099 | | 5,0233 | | 0,000597 | | | 0,00598 |
| C6orf160 | PREDICTED: Homo sapiens chromosome 6 open reading frame 160, transcript variant 4 (C6orf160), mRNA. | 0,7814 | 11,8083 | | 11,3465 | | 0,000001 | | | 0,00029 |
| GAB2 | Homo sapiens GRB2-associated binding protein 2 (GAB2), transcript variant 1, mRNA. | 0,7806 | 7,5501 | | 3,9460 | | 0,003003 | | | 0,01690 |
| TP53BP2 | Homo sapiens tumor protein p53 binding protein, 2 (TP53BP2), transcript variant 1, mRNA. | 0,7802 | 8,4783 | | 4,8806 | | 0,000732 | | | 0,00674 |
| SERINC3 | Homo sapiens serine incorporator 3 (SERINC3), transcript variant 1, mRNA. | 0,7798 | 9,4904 | | 6,0803 | | 0,000144 | | | 0,00272 |
| TMEM44 | Homo sapiens transmembrane protein 44 (TMEM44), transcript variant 1, mRNA. | 0,7797 | 8,1288 | | 3,1619 | | 0,010700 | | | 0,04175 |
| MBOAT2 | PREDICTED: Homo sapiens membrane bound O-acyltransferase domain containing 2 (MBOAT2), mRNA. | 0,7794 | 8,5269 | | 3,1941 | | 0,010145 | | | 0,04019 |
| GJB3 | Homo sapiens gap junction protein, beta 3, 31kDa (GJB3), transcript variant 1, mRNA. | 0,7792 | 7,7488 | | 4,0186 | | 0,002679 | | | 0,01571 |
| CLK3 | Homo sapiens CDC-like kinase 3 (CLK3), transcript variant phclk3, mRNA. | 0,7784 | 8,3010 | | 6,3989 | | 0,000096 | | | 0,00218 |
| LOC440595 | PREDICTED: Homo sapiens misc_RNA (LOC440595), miscRNA. | 0,7777 | 9,0698 | | 5,4777 | | 0,000317 | | | 0,00421 |
| LOC644739 | PREDICTED: Homo sapiens similar to Wiskott-Aldrich syndrome protein family member 4 (WASP-family protein member 4), transcript variant 2 (LOC644739), mRNA. | 0,7763 | 8,8516 | | 4,1438 | | 0,002205 | | | 0,01374 |
| EPHA2 | Homo sapiens EPH receptor A2 (EPHA2), mRNA. | 0,7759 | 8,9385 | | 3,1376 | | 0,011140 | | | 0,04305 |
| ARL8B | Homo sapiens ADP-ribosylation factor-like 8B (ARL8B), mRNA. | 0,7756 | 9,6469 | | 7,3870 | | 0,000030 | | | 0,00127 |
| PYGB | Homo sapiens phosphorylase, glycogen; brain (PYGB), mRNA. | 0,7752 | 9,9039 | | 3,5514 | | 0,005644 | | | 0,02637 |
| RNU6ATAC | Homo sapiens RNA, U6atac small nuclear (U12-dependent splicing) (RNU6ATAC), small nuclear RNA. | 0,7751 | 7,5598 | | 4,4918 | | 0,001298 | | | 0,00966 |
| MACF1 | Homo sapiens microtubule-actin crosslinking factor 1 (MACF1), transcript variant 1, mRNA. | 0,7748 | 8,2971 | | 4,5610 | | 0,001170 | | | 0,00907 |
| CSNK1D | Homo sapiens casein kinase 1, delta (CSNK1D), transcript variant 2, mRNA. | 0,7747 | 9,4386 | | 5,4562 | | 0,000327 | | | 0,00427 |
| TAF1D | Homo sapiens TATA box binding protein (TBP)-associated factor, RNA polymerase I, D, 41kDa (TAF1D), mRNA. | 0,7746 | 7,9748 | | 6,1931 | | 0,000124 | | | 0,00249 |
| MGC102966 | PREDICTED: Homo sapiens similar to Keratin, type I cytoskeletal 16 (Cytokeratin-16) (CK-16) (Keratin-16) (K16) (MGC102966), misc RNA. | 0,7739 | 8,1132 | | 3,5102 | | 0,006034 | | | 0,02776 |
| LOC730278 | PREDICTED: Homo sapiens hypothetical LOC730278 (LOC730278), mRNA. | 0,7728 | 12,5632 | | 3,4927 | | 0,006209 | | | 0,02832 |
| NAPG | Homo sapiens N-ethylmaleimide-sensitive factor attachment protein, gamma (NAPG), mRNA. | 0,7703 | 8,3660 | | 3,3567 | | 0,007757 | | | 0,03334 |
| ZNF296 | Homo sapiens zinc finger protein 296 (ZNF296), mRNA. | 0,7698 | 8,2784 | | 5,0535 | | 0,000572 | | | 0,00584 |
| LOC100131989 | PREDICTED: Homo sapiens hypothetical protein LOC100131989 (LOC100131989), mRNA. | 0,7698 | 7,8913 | | 4,9176 | | 0,000694 | | | 0,00651 |
| MKNK2 | Homo sapiens MAP kinase interacting serine/threonine kinase 2 (MKNK2), transcript variant 1, mRNA. | 0,7696 | 9,4602 | | 3,1944 | | 0,010140 | | | 0,04018 |
| PMAIP1 | Homo sapiens phorbol-12-myristate-13-acetate-induced protein 1 (PMAIP1), mRNA. | 0,7690 | 7,6813 | | 3,8337 | | 0,003587 | | | 0,01910 |
| RPRC1 | Homo sapiens arginine/proline rich coiled-coil 1 (RPRC1), mRNA. | 0,7682 | 11,7796 | | 7,0837 | | 0,000043 | | | 0,00148 |
| HIST2H2AC | Homo sapiens histone cluster 2, H2ac (HIST2H2AC), mRNA. | 0,7681 | 7,8522 | | 3,4493 | | 0,006664 | | | 0,02983 |
| NCRNA00219 | Homo sapiens non-protein coding RNA 219 (NCRNA00219), non-coding RNA. | 0,7679 | 9,3042 | | 11,5276 | | 0,000001 | | | 0,00029 |
| USP49 | Homo sapiens ubiquitin specific peptidase 49 (USP49), mRNA. | 0,7676 | 8,5896 | | 4,6829 | | 0,000977 | | | 0,00814 |
| MLL4 | Homo sapiens myeloid/lymphoid or mixed-lineage leukemia 4 (MLL4), mRNA. | 0,7667 | 8,2205 | | 6,4126 | | 0,000095 | | | 0,00216 |
| C18orf8 | Homo sapiens chromosome 18 open reading frame 8 (C18orf8), mRNA. | 0,7664 | 8,8138 | | 8,4606 | | 0,000010 | | | 0,00080 |
| C9orf30 | Homo sapiens chromosome 9 open reading frame 30 (C9orf30), mRNA. | 0,7663 | 10,3979 | | 5,3861 | | 0,000360 | | | 0,00445 |
| SPTLC1 | Homo sapiens serine palmitoyltransferase, long chain base subunit 1 (SPTLC1), transcript variant 2, mRNA. | 0,7660 | 8,1405 | | 3,9115 | | 0,003171 | | | 0,01751 |
| COPA | Homo sapiens coatomer protein complex, subunit alpha (COPA), transcript variant 2, mRNA. | 0,7642 | 10,6369 | | 5,8657 | | 0,000189 | | | 0,00317 |
| BMP2 | Homo sapiens bone morphogenetic protein 2 (BMP2), mRNA. | 0,7638 | 8,1419 | | 5,5985 | | 0,000270 | | | 0,00386 |
| LOC647673 | PREDICTED: Homo sapiens similar to Translationally-controlled tumor protein (TCTP) (p23) (Histamine-releasing factor) (HRF) (Fortilin) (LOC647673), mRNA. | 0,7630 | 8,6564 | | 4,4646 | | 0,001351 | | | 0,00994 |
| AMY1A | Homo sapiens amylase, alpha 1A (salivary) (AMY1A), transcript variant 1, mRNA. | 0,7626 | 9,7425 | | 3,4902 | | 0,006234 | | | 0,02841 |
| SNORA28 | Homo sapiens small nucleolar RNA, H/ACA box 28 (SNORA28), small nucleolar RNA. | 0,7624 | 7,5578 | | 7,5598 | | 0,000025 | | | 0,00118 |
| STK10 | Homo sapiens serine/threonine kinase 10 (STK10), mRNA. | 0,7619 | 8,0662 | | 4,7843 | | 0,000842 | | | 0,00741 |
| MSX1 | Homo sapiens msh homeobox 1 (MSX1), mRNA. | 0,7611 | 8,4014 | | 4,0912 | | 0,002392 | | | 0,01455 |
| FBXO11 | Homo sapiens F-box protein 11 (FBXO11), transcript variant 1, mRNA. | 0,7610 | 9,4273 | | 7,3342 | | 0,000032 | | | 0,00129 |
| RSRC2 | Homo sapiens arginine/serine-rich coiled-coil 2 (RSRC2), transcript variant 1, mRNA. | 0,7606 | 9,4395 | | 9,4411 | | 0,000004 | | | 0,00052 |
| LOC392145 | PREDICTED: Homo sapiens similar to Mtr3 (mRNA transport regulator 3)-homolog (LOC392145), mRNA. | 0,7603 | 7,5489 | | 4,5741 | | 0,001147 | | | 0,00897 |
| PSAP | Homo sapiens prosaposin (PSAP), transcript variant 1, mRNA. | 0,7600 | 11,3927 | | 6,0628 | | 0,000147 | | | 0,00276 |
| OSBPL10 | Homo sapiens oxysterol binding protein-like 10 (OSBPL10), mRNA. | 0,7595 | 9,2204 | | 4,1303 | | 0,002252 | | | 0,01393 |
| IRX4 | Homo sapiens iroquois homeobox 4 (IRX4), mRNA. | 0,7589 | 7,6707 | | 3,9951 | | 0,002780 | | | 0,01607 |
| LOC344887 | PREDICTED: Homo sapiens misc_RNA (LOC344887), miscRNA. | 0,7585 | 7,8336 | | 4,9025 | | 0,000709 | | | 0,00661 |
| TGIF1 | Homo sapiens TGFB-induced factor homeobox 1 (TGIF1), transcript variant 1, mRNA. | 0,7542 | 10,3149 | | 3,5412 | | 0,005738 | | | 0,02672 |
| LOC645895 | PREDICTED: Homo sapiens hypothetical protein LOC645895 (LOC645895), mRNA. | 0,7527 | 12,7164 | | 5,4552 | | 0,000327 | | | 0,00427 |
| SEC14L1 | Homo sapiens SEC14-like 1 (S. cerevisiae) (SEC14L1), transcript variant 1, mRNA. | 0,7527 | 7,9645 | | 5,9660 | | 0,000166 | | | 0,00295 |
| STAT2 | Homo sapiens signal transducer and activator of transcription 2, 113kDa (STAT2), mRNA. | 0,7514 | 8,9184 | | 4,1078 | | 0,002331 | | | 0,01427 |
| HLA-E | Homo sapiens major histocompatibility complex, class I, E (HLA-E), mRNA. | 0,7501 | 9,8601 | | 5,3616 | | 0,000372 | | | 0,00454 |
| AGPAT2 | Homo sapiens 1-acylglycerol-3-phosphate O-acyltransferase 2 (lysophosphatidic acid acyltransferase, beta) (AGPAT2), transcript variant 1, mRNA. | 0,7497 | 8,5471 | | 5,1611 | | 0,000491 | | | 0,00537 |
| RSPRY1 | Homo sapiens ring finger and SPRY domain containing 1 (RSPRY1), mRNA. | 0,7494 | 9,2504 | | 5,7224 | | 0,000229 | | | 0,00352 |
| DLGAP4 | Homo sapiens discs, large (Drosophila) homolog-associated protein 4 (DLGAP4), transcript variant 3, mRNA. | 0,7491 | 8,8646 | | 3,9976 | | 0,002769 | | | 0,01604 |
| TSC22D2 | Homo sapiens TSC22 domain family, member 2 (TSC22D2), mRNA. | 0,7476 | 8,2515 | | 4,7095 | | 0,000939 | | | 0,00797 |
| CRLF3 | Homo sapiens cytokine receptor-like factor 3 (CRLF3), mRNA. | 0,7476 | 8,6230 | | 5,4777 | | 0,000317 | | | 0,00421 |
| ROCK2 | Homo sapiens Rho-associated, coiled-coil containing protein kinase 2 (ROCK2), mRNA. | 0,7474 | 12,9111 | | 3,8034 | | 0,003764 | | | 0,01971 |
| CAMSAP1 | Homo sapiens calmodulin regulated spectrin-associated protein 1 (CAMSAP1), mRNA. | 0,7474 | 8,1153 | | 4,6448 | | 0,001033 | | | 0,00840 |
| EHD4 | Homo sapiens EH-domain containing 4 (EHD4), mRNA. | 0,7472 | 9,2186 | | 5,5039 | | 0,000306 | | | 0,00412 |
| SMURF1 | Homo sapiens SMAD specific E3 ubiquitin protein ligase 1 (SMURF1), transcript variant 2, mRNA. | 0,7471 | 7,9097 | | 3,5115 | | 0,006023 | | | 0,02772 |
| ARF4 | Homo sapiens ADP-ribosylation factor 4 (ARF4), mRNA. | 0,7464 | 11,9948 | | 3,5049 | | 0,006088 | | | 0,02796 |
| LOC729279 | PREDICTED: Homo sapiens misc_RNA (LOC729279), miscRNA. | 0,7453 | 10,6442 | | 6,5489 | | 0,000080 | | | 0,00200 |
| NAMPT | Homo sapiens nicotinamide phosphoribosyltransferase (NAMPT), mRNA. | 0,7444 | 8,3073 | | 6,2011 | | 0,000123 | | | 0,00247 |
| SPRR1B | Homo sapiens small proline-rich protein 1B (cornifin) (SPRR1B), mRNA. | 0,7435 | 7,7510 | | 3,3157 | | 0,008297 | | | 0,03487 |
| TNK2 | Homo sapiens tyrosine kinase, non-receptor, 2 (TNK2), transcript variant 1, mRNA. | 0,7433 | 9,0794 | | 5,1321 | | 0,000512 | | | 0,00548 |
| PI3 | Homo sapiens peptidase inhibitor 3, skin-derived (SKALP) (PI3), mRNA. | 0,7431 | 7,7687 | | 4,4441 | | 0,001394 | | | 0,01015 |
| RAB5B | Homo sapiens RAB5B, member RAS oncogene family (RAB5B), mRNA. | 0,7425 | 9,9466 | | 5,1603 | | 0,000492 | | | 0,00537 |
| FHL2 | Homo sapiens four and a half LIM domains 2 (FHL2), transcript variant 4, mRNA. | 0,7418 | 11,0287 | | 8,4761 | | 0,000010 | | | 0,00079 |
| AGPAT2 | Homo sapiens 1-acylglycerol-3-phosphate O-acyltransferase 2 (lysophosphatidic acid acyltransferase, beta) (AGPAT2), transcript variant 1, mRNA. | 0,7418 | 8,4552 | | 5,7253 | | 0,000228 | | | 0,00351 |
| UBA6 | Homo sapiens ubiquitin-like modifier activating enzyme 6 (UBA6), mRNA. | 0,7418 | 8,9076 | | 8,0658 | | 0,000014 | | | 0,00095 |
| INPP1 | Homo sapiens inositol polyphosphate-1-phosphatase (INPP1), mRNA. | 0,7409 | 8,5884 | | 4,9479 | | 0,000665 | | | 0,00633 |
| FHL2 | Homo sapiens four and a half LIM domains 2 (FHL2), transcript variant 2, mRNA. | 0,7406 | 11,6175 | | 6,3050 | | 0,000108 | | | 0,00231 |
| ZNF259 | Homo sapiens zinc finger protein 259 (ZNF259), mRNA. | 0,7398 | 9,0865 | | 5,4725 | | 0,000320 | | | 0,00422 |
| KLF5 | Homo sapiens Kruppel-like factor 5 (intestinal) (KLF5), mRNA. | 0,7388 | 7,7419 | | 5,4648 | | 0,000323 | | | 0,00424 |
| RAB5A | Homo sapiens RAB5A, member RAS oncogene family (RAB5A), mRNA. | 0,7388 | 9,1763 | | 5,4110 | | 0,000348 | | | 0,00437 |
| MBD4 | Homo sapiens methyl-CpG binding domain protein 4 (MBD4), mRNA. | 0,7388 | 8,7122 | | 3,2573 | | 0,009136 | | | 0,03724 |
| C12orf44 | Homo sapiens chromosome 12 open reading frame 44 (C12orf44), transcript variant 2, mRNA. | 0,7387 | 9,4256 | | 4,7947 | | 0,000829 | | | 0,00732 |
| ZNF277 | Homo sapiens zinc finger protein 277 (ZNF277), mRNA. | 0,7380 | 8,4573 | | 6,6232 | | 0,000073 | | | 0,00191 |
| DGKA | Homo sapiens diacylglycerol kinase, alpha 80kDa (DGKA), transcript variant 4, mRNA. | 0,7373 | 8,2031 | | 3,4254 | | 0,006930 | | | 0,03072 |
| ARID3A | Homo sapiens AT rich interactive domain 3A (BRIGHT-like) (ARID3A), mRNA. | 0,7372 | 8,2816 | | 4,6634 | | 0,001005 | | | 0,00827 |
| FAM100A | Homo sapiens family with sequence similarity 100, member A (FAM100A), mRNA. | 0,7368 | 8,5389 | | 5,5522 | | 0,000287 | | | 0,00398 |
| BRI3P1 | PREDICTED: Homo sapiens misc_RNA (BRI3P1), miscRNA. | 0,7367 | 9,0948 | | 4,4371 | | 0,001409 | | | 0,01022 |
| TNFRSF12A | Homo sapiens tumor necrosis factor receptor superfamily, member 12A (TNFRSF12A), mRNA. | 0,7358 | 11,5056 | | 7,9248 | | 0,000017 | | | 0,00098 |
| TUBB2B | Homo sapiens tubulin, beta 2B (TUBB2B), mRNA. | 0,7348 | 7,6010 | | 6,5879 | | 0,000076 | | | 0,00196 |
| ASAP2 | Homo sapiens ArfGAP with SH3 domain, ankyrin repeat and PH domain 2 (ASAP2), transcript variant 1, mRNA. | 0,7344 | 9,8892 | | 6,1073 | | 0,000139 | | | 0,00267 |
| PRNP | Homo sapiens prion protein (PRNP), transcript variant 2, mRNA. | 0,7344 | 11,9129 | | 4,6428 | | 0,001036 | | | 0,00841 |
| PPP1R13L | Homo sapiens protein phosphatase 1, regulatory (inhibitor) subunit 13 like (PPP1R13L), mRNA. | 0,7333 | 9,9371 | | 4,9870 | | 0,000629 | | | 0,00615 |
| JMJD1C | Homo sapiens jumonji domain containing 1C (JMJD1C), transcript variant 1, mRNA. | 0,7330 | 8,1137 | | 4,6422 | | 0,001037 | | | 0,00841 |
| ZMAT3 | Homo sapiens zinc finger, matrin type 3 (ZMAT3), transcript variant 2, mRNA. | 0,7327 | 11,8265 | | 5,1196 | | 0,000521 | | | 0,00553 |
| PIK3CD | Homo sapiens phosphoinositide-3-kinase, catalytic, delta polypeptide (PIK3CD), mRNA. | 0,7326 | 8,0580 | | 4,9617 | | 0,000652 | | | 0,00628 |
| LOC399748 | PREDICTED: Homo sapiens misc_RNA (LOC399748), miscRNA. | 0,7319 | 11,0949 | | 4,7618 | | 0,000870 | | | 0,00757 |
| WBP5 | Homo sapiens WW domain binding protein 5 (WBP5), transcript variant 4, mRNA. | 0,7313 | 9,4744 | | 4,3332 | | 0,001649 | | | 0,01131 |
| SELK | Homo sapiens selenoprotein K (SELK), mRNA. | 0,7311 | 8,8183 | | 7,9975 | | 0,000016 | | | 0,00096 |
| PRSS22 | Homo sapiens protease, serine, 22 (PRSS22), mRNA. | 0,7307 | 8,1813 | | 7,0836 | | 0,000043 | | | 0,00148 |
| PCGF1 | Homo sapiens polycomb group ring finger 1 (PCGF1), mRNA. | 0,7301 | 8,8247 | | 6,5167 | | 0,000083 | | | 0,00203 |
| DMWD | Homo sapiens dystrophia myotonica, WD repeat containing (DMWD), mRNA. | 0,7295 | 8,6380 | | 4,3179 | | 0,001688 | | | 0,01149 |
| ILK | Homo sapiens integrin-linked kinase (ILK), transcript variant 1, mRNA. | 0,7292 | 10,9835 | | 4,9491 | | 0,000664 | | | 0,00633 |
| PSAP | Homo sapiens prosaposin (PSAP), transcript variant 2, mRNA. | 0,7273 | 11,4096 | | 4,9972 | | 0,000619 | | | 0,00611 |
| C1orf63 | Homo sapiens chromosome 1 open reading frame 63 (C1orf63), mRNA. | 0,7271 | 7,9754 | | 5,7172 | | 0,000230 | | | 0,00352 |
| TRIP12 | Homo sapiens thyroid hormone receptor interactor 12 (TRIP12), mRNA. | 0,7256 | 9,2518 | | 3,7723 | | 0,003955 | | | 0,02041 |
| ARIH1 | Homo sapiens ariadne homolog, ubiquitin-conjugating enzyme E2 binding protein, 1 (Drosophila) (ARIH1), mRNA. | 0,7254 | 7,9935 | | 6,3746 | | 0,000099 | | | 0,00222 |
| PLDN | Homo sapiens pallidin homolog (mouse) (PLDN), mRNA. | 0,7252 | 9,1031 | | 5,7615 | | 0,000217 | | | 0,00342 |
| RTN2 | Homo sapiens reticulon 2 (RTN2), transcript variant 3, mRNA. | 0,7250 | 7,9069 | | 5,8330 | | 0,000198 | | | 0,00324 |
| FNBP1L | Homo sapiens formin binding protein 1-like (FNBP1L), transcript variant 1, mRNA. | 0,7245 | 8,4641 | | 5,1578 | | 0,000494 | | | 0,00538 |
| TRAM1 | Homo sapiens translocation associated membrane protein 1 (TRAM1), mRNA. | 0,7243 | 11,6543 | | 14,8116 | | 0,000000 | | | 0,00012 |
| DBNDD2 | Homo sapiens dysbindin (dystrobrevin binding protein 1) domain containing 2 (DBNDD2), transcript variant 3, mRNA. | 0,7235 | 9,4778 | | 3,1329 | | 0,011228 | | | 0,04330 |
| ARHGEF18 | Homo sapiens rho/rac guanine nucleotide exchange factor (GEF) 18 (ARHGEF18), mRNA. | 0,7231 | 10,1014 | | 3,5860 | | 0,005336 | | | 0,02527 |
| ICA1 | Homo sapiens islet cell autoantigen 1, 69kDa (ICA1), transcript variant 3, mRNA. | 0,7220 | 8,0068 | | 5,3110 | | 0,000399 | | | 0,00476 |
| CLDN15 | Homo sapiens claudin 15 (CLDN15), mRNA. | 0,7220 | 7,8851 | | 4,5411 | | 0,001205 | | | 0,00922 |
| PSCD2 | Homo sapiens pleckstrin homology, Sec7 and coiled-coil domains 2 (cytohesin-2) (PSCD2), transcript variant 2, mRNA. | 0,7210 | 8,0592 | | 5,6078 | | 0,000266 | | | 0,00383 |
| NFKB2 | Homo sapiens nuclear factor of kappa light polypeptide gene enhancer in B-cells 2 (p49/p100) (NFKB2), transcript variant 3, mRNA. | 0,7210 | 8,2853 | | 5,3113 | | 0,000399 | | | 0,00476 |
| STK40 | Homo sapiens serine/threonine kinase 40 (STK40), mRNA. | 0,7206 | 8,9267 | | 5,0678 | | 0,000560 | | | 0,00579 |
| SLC4A5 | Homo sapiens solute carrier family 4, sodium bicarbonate cotransporter, member 5 (SLC4A5), transcript variant c, mRNA. | 0,7204 | 7,9846 | | 4,5333 | | 0,001219 | | | 0,00927 |
| ANKRD11 | Homo sapiens ankyrin repeat domain 11 (ANKRD11), mRNA. | 0,7198 | 9,3796 | | 4,8301 | | 0,000788 | | | 0,00708 |
| DAB2 | Homo sapiens disabled homolog 2, mitogen-responsive phosphoprotein (Drosophila) (DAB2), mRNA. | 0,7190 | 7,7984 | | 3,6577 | | 0,004753 | | | 0,02325 |
| CREB3L2 | Homo sapiens cAMP responsive element binding protein 3-like 2 (CREB3L2), mRNA. | 0,7188 | 9,0856 | | 4,9604 | | 0,000653 | | | 0,00629 |
| PITPNM1 | Homo sapiens phosphatidylinositol transfer protein, membrane-associated 1 (PITPNM1), mRNA. | 0,7187 | 9,6454 | | 4,4646 | | 0,001352 | | | 0,00994 |
| MYADM | Homo sapiens myeloid-associated differentiation marker (MYADM), transcript variant 2, mRNA. | 0,7180 | 7,7139 | | 10,4682 | | 0,000002 | | | 0,00038 |
| MBOAT2 | Homo sapiens membrane bound O-acyltransferase domain containing 2 (MBOAT2), mRNA. | 0,7168 | 8,0666 | | 3,5291 | | 0,005852 | | | 0,02715 |
| LOC100130053 | PREDICTED: Homo sapiens hypothetical protein LOC100130053 (LOC100130053), mRNA. | 0,7164 | 7,9053 | | 4,5373 | | 0,001212 | | | 0,00925 |
| DAP | Homo sapiens death-associated protein (DAP), mRNA. | 0,7163 | 9,1992 | | 3,9610 | | 0,002933 | | | 0,01667 |
| MEX3C | Homo sapiens mex-3 homolog C (C. elegans) (MEX3C), mRNA. | 0,7163 | 8,3438 | | 7,5117 | | 0,000026 | | | 0,00121 |
| LOC400578 | PREDICTED: Homo sapiens similar to Keratin, type I cytoskeletal 14 (Cytokeratin-14) (CK-14) (Keratin-14) (K14) (LOC400578), mRNA. | 0,7161 | 7,8045 | | 4,5288 | | 0,001228 | | | 0,00931 |
| SAMD4A | Homo sapiens sterile alpha motif domain containing 4A (SAMD4A), mRNA. | 0,7156 | 7,6873 | | 4,4663 | | 0,001348 | | | 0,00993 |
| PEA15 | Homo sapiens phosphoprotein enriched in astrocytes 15 (PEA15), mRNA. | 0,7148 | 10,1316 | | 5,3896 | | 0,000358 | | | 0,00443 |
| JUP | Homo sapiens junction plakoglobin (JUP), transcript variant 1, mRNA. | 0,7138 | 8,7232 | | 4,5281 | | 0,001229 | | | 0,00932 |
| CLCN7 | Homo sapiens chloride channel 7 (CLCN7), mRNA. | 0,7137 | 10,2429 | | 4,0225 | | 0,002663 | | | 0,01564 |
| RAB2A | Homo sapiens RAB2A, member RAS oncogene family (RAB2A), mRNA. | 0,7133 | 9,0394 | | 3,7864 | | 0,003867 | | | 0,02010 |
| LOC286512 | PREDICTED: Homo sapiens misc_RNA (LOC286512), miscRNA. | 0,7117 | 10,5026 | | 3,2315 | | 0,009536 | | | 0,03838 |
| OKL38 | Homo sapiens pregnancy-induced growth inhibitor (OKL38), transcript variant 1, mRNA. | 0,7113 | 7,9860 | | 5,1539 | | 0,000496 | | | 0,00539 |
| FHL1 | Homo sapiens four and a half LIM domains 1 (FHL1), mRNA. | 0,7111 | 8,8688 | | 4,2517 | | 0,001867 | | | 0,01226 |
| GOLGA2 | Homo sapiens golgi autoantigen, golgin subfamily a, 2 (GOLGA2), mRNA. | 0,7110 | 8,3370 | | 4,8030 | | 0,000819 | | | 0,00726 |
| ASAP2 | Homo sapiens ArfGAP with SH3 domain, ankyrin repeat and PH domain 2 (ASAP2), transcript variant 1, mRNA. | 0,7096 | 8,8480 | | 5,0778 | | 0,000552 | | | 0,00573 |
| KBTBD2 | Homo sapiens kelch repeat and BTB (POZ) domain containing 2 (KBTBD2), mRNA. | 0,7092 | 9,8382 | | 4,2348 | | 0,001916 | | | 0,01249 |
| YRDC | Homo sapiens yrdC domain containing (E. coli) (YRDC), nuclear gene encoding mitochondrial protein, mRNA. | 0,7085 | 10,0984 | | 4,0256 | | 0,002650 | | | 0,01561 |
| DPH3 | Homo sapiens DPH3, KTI11 homolog (S. cerevisiae) (DPH3), transcript variant 2, mRNA. | 0,7083 | 8,4493 | | 7,6653 | | 0,000022 | | | 0,00113 |
| PTPLAD2 | Homo sapiens protein tyrosine phosphatase-like A domain containing 2 (PTPLAD2), mRNA. | 0,7068 | 8,0739 | | 5,2551 | | 0,000431 | | | 0,00497 |
| C11orf68 | Homo sapiens chromosome 11 open reading frame 68 (C11orf68), mRNA. | 0,7064 | 9,2362 | | 3,4975 | | 0,006161 | | | 0,02817 |
| ZYX | Homo sapiens zyxin (ZYX), transcript variant 1, mRNA. | 0,7054 | 9,1890 | | 6,3286 | | 0,000105 | | | 0,00227 |
| TUBB3 | Homo sapiens tubulin, beta 3 (TUBB3), mRNA. | 0,7042 | 10,2915 | | 4,3121 | | 0,001703 | | | 0,01155 |
| PDCD4 | Homo sapiens programmed cell death 4 (neoplastic transformation inhibitor) (PDCD4), transcript variant 2, mRNA. | 0,7039 | 8,6702 | | 4,1883 | | 0,002059 | | | 0,01311 |
| PCMTD1 | Homo sapiens protein-L-isoaspartate (D-aspartate) O-methyltransferase domain containing 1 (PCMTD1), mRNA. | 0,7035 | 8,2758 | | 5,8391 | | 0,000196 | | | 0,00322 |
| NFKB2 | Homo sapiens nuclear factor of kappa light polypeptide gene enhancer in B-cells 2 (p49/p100) (NFKB2), transcript variant 2, mRNA. | 0,7027 | 8,1926 | | 5,8522 | | 0,000193 | | | 0,00320 |
| LOC100132291 | PREDICTED: Homo sapiens similar to hCG2027326 (LOC100132291), mRNA. | 0,7023 | 11,0343 | | 3,9157 | | 0,003150 | | | 0,01744 |
| PCNX | Homo sapiens pecanex homolog (Drosophila) (PCNX), mRNA. | 0,7022 | 8,0644 | | 4,5660 | | 0,001161 | | | 0,00902 |
| TMEM106A | Homo sapiens transmembrane protein 106A (TMEM106A), mRNA. | 0,7004 | 7,8484 | | 5,8232 | | 0,000200 | | | 0,00327 |
| SNORD104 | Homo sapiens small nucleolar RNA, C/D box 104 (SNORD104), small nucleolar RNA. | 0,7000 | 8,5068 | | 3,1698 | | 0,010560 | | | 0,04133 |
| TPM4 | Homo sapiens tropomyosin 4 (TPM4), mRNA. | 0,6999 | 9,5569 | | 8,9489 | | 0,000006 | | | 0,00064 |
| SNHG8 | Homo sapiens small nucleolar RNA host gene 8 (non-protein coding) (SNHG8), non-coding RNA. | 0,6989 | 9,1748 | | 6,6144 | | 0,000074 | | | 0,00192 |
| PRKCZ | Homo sapiens protein kinase C, zeta (PRKCZ), transcript variant 1, mRNA. | 0,6983 | 8,7638 | | 9,6729 | | 0,000003 | | | 0,00049 |
| FLJ40722 | PREDICTED: Homo sapiens hypothetical protein FLJ40722, transcript variant 3 (FLJ40722), mRNA. | 0,6983 | 7,8406 | | 4,7538 | | 0,000880 | | | 0,00762 |
| TRPC4AP | Homo sapiens transient receptor potential cation channel, subfamily C, member 4 associated protein (TRPC4AP), transcript variant 1, mRNA. | 0,6983 | 9,2755 | | 5,4433 | | 0,000333 | | | 0,00430 |
| AGPAT2 | Homo sapiens 1-acylglycerol-3-phosphate O-acyltransferase 2 (lysophosphatidic acid acyltransferase, beta) (AGPAT2), transcript variant 2, mRNA. | 0,6977 | 8,5948 | | 3,3062 | | 0,008428 | | | 0,03523 |
| DPH3 | Homo sapiens DPH3, KTI11 homolog (S. cerevisiae) (DPH3), transcript variant 1, mRNA. | 0,6973 | 8,4223 | | 5,4799 | | 0,000316 | | | 0,00421 |
| SEC24D | Homo sapiens SEC24 related gene family, member D (S. cerevisiae) (SEC24D), mRNA. | 0,6972 | 8,1786 | | 6,4179 | | 0,000094 | | | 0,00216 |
| SLC35E1 | Homo sapiens solute carrier family 35, member E1 (SLC35E1), mRNA. | 0,6968 | 8,6816 | | 3,2488 | | 0,009266 | | | 0,03758 |
| SNX6 | Homo sapiens sorting nexin 6 (SNX6), transcript variant 2, mRNA. | 0,6968 | 8,3460 | | 3,3608 | | 0,007705 | | | 0,03316 |
| CLTB | Homo sapiens clathrin, light chain (Lcb) (CLTB), transcript variant 2, mRNA. | 0,6962 | 11,0684 | | 3,4464 | | 0,006697 | | | 0,02995 |
| NOTCH2NL | Homo sapiens Notch homolog 2 (Drosophila) N-terminal like (NOTCH2NL), mRNA. | 0,6959 | 8,1455 | | 3,4942 | | 0,006194 | | | 0,02827 |
| LOC389765 | PREDICTED: Homo sapiens similar to KIF27C (LOC389765), mRNA. | 0,6957 | 7,8216 | | 5,0895 | | 0,000543 | | | 0,00568 |
| BIRC2 | Homo sapiens baculoviral IAP repeat-containing 2 (BIRC2), mRNA. | 0,6957 | 9,5733 | | 4,4336 | | 0,001416 | | | 0,01024 |
| MYO5A | Homo sapiens myosin VA (heavy chain 12, myoxin) (MYO5A), mRNA. | 0,6935 | 8,1609 | | 3,7013 | | 0,004431 | | | 0,02216 |
| EFTUD1 | Homo sapiens elongation factor Tu GTP binding domain containing 1 (EFTUD1), transcript variant 1, mRNA. | 0,6932 | 8,1061 | | 5,1764 | | 0,000481 | | | 0,00531 |
| PLEKHB2 | Homo sapiens pleckstrin homology domain containing, family B (evectins) member 2 (PLEKHB2), transcript variant 1, mRNA. | 0,6930 | 8,6491 | | 4,4739 | | 0,001333 | | | 0,00985 |
| SOD2 | Homo sapiens superoxide dismutase 2, mitochondrial (SOD2), nuclear gene encoding mitochondrial protein, transcript variant 2, mRNA. | 0,6928 | 9,4852 | | 4,1162 | | 0,002301 | | | 0,01412 |
| IER2 | Homo sapiens immediate early response 2 (IER2), mRNA. | 0,6920 | 8,0702 | | 4,2460 | | 0,001884 | | | 0,01234 |
| PNPLA8 | Homo sapiens patatin-like phospholipase domain containing 8 (PNPLA8), mRNA. | 0,6919 | 7,8136 | | 8,2181 | | 0,000012 | | | 0,00088 |
| BCL10 | Homo sapiens B-cell CLL/lymphoma 10 (BCL10), mRNA. | 0,6918 | 8,1744 | | 5,4748 | | 0,000319 | | | 0,00422 |
| MNT | Homo sapiens MAX binding protein (MNT), mRNA. | 0,6916 | 8,5179 | | 5,2246 | | 0,000450 | | | 0,00509 |
| LOC645381 | PREDICTED: Homo sapiens misc_RNA (LOC645381), miscRNA. | 0,6913 | 8,2736 | | 5,5429 | | 0,000291 | | | 0,00400 |
| SCYL1 | Homo sapiens SCY1-like 1 (S. cerevisiae) (SCYL1), transcript variant A, mRNA. | 0,6905 | 9,6753 | | 8,0606 | | 0,000015 | | | 0,00095 |
| BCL2L1 | Homo sapiens BCL2-like 1 (BCL2L1), nuclear gene encoding mitochondrial protein, transcript variant 1, mRNA. | 0,6902 | 8,3174 | | 5,6442 | | 0,000254 | | | 0,00372 |
| CDH1 | Homo sapiens cadherin 1, type 1, E-cadherin (epithelial) (CDH1), mRNA. | 0,6898 | 10,8711 | | 3,3256 | | 0,008163 | | | 0,03444 |
| JHDM1D | Homo sapiens jumonji C domain containing histone demethylase 1 homolog D (S. cerevisiae) (JHDM1D), mRNA. | 0,6898 | 7,5910 | | 5,4444 | | 0,000332 | | | 0,00430 |
| PRKCZ | Homo sapiens protein kinase C, zeta (PRKCZ), transcript variant 1, mRNA. | 0,6898 | 8,4258 | | 16,5707 | | 0,000000 | | | 0,00008 |
| NCK2 | Homo sapiens NCK adaptor protein 2 (NCK2), transcript variant 2, mRNA. | 0,6892 | 9,6106 | | 9,6038 | | 0,000003 | | | 0,00050 |
| MYO6 | Homo sapiens myosin VI (MYO6), mRNA. | 0,6890 | 8,1570 | | 4,4578 | | 0,001365 | | | 0,01001 |
| CARS | Homo sapiens cysteinyl-tRNA synthetase (CARS), transcript variant 4, mRNA. | 0,6889 | 8,4905 | | 5,1319 | | 0,000512 | | | 0,00548 |
| PUM1 | Homo sapiens pumilio homolog 1 (Drosophila) (PUM1), transcript variant 1, mRNA. | 0,6883 | 9,8547 | | 6,3157 | | 0,000107 | | | 0,00230 |
| TIGA1 | Homo sapiens TIGA1 (TIGA1), mRNA. | 0,6876 | 9,5231 | | 8,1669 | | 0,000013 | | | 0,00090 |
| EFNA1 | Homo sapiens ephrin-A1 (EFNA1), transcript variant 1, mRNA. | 0,6873 | 7,9052 | | 6,1220 | | 0,000136 | | | 0,00264 |
| LOC100127975 | PREDICTED: Homo sapiens misc_RNA (LOC100127975), partial miscRNA. | 0,6873 | 8,2529 | | 4,6556 | | 0,001017 | | | 0,00832 |
| CIB1 | Homo sapiens calcium and integrin binding 1 (calmyrin) (CIB1), mRNA. | 0,6871 | 11,2382 | | 3,4276 | | 0,006906 | | | 0,03064 |
| GPR175 | Homo sapiens G protein-coupled receptor 175 (GPR175), mRNA. | 0,6868 | 8,3437 | | 11,0858 | | 0,000001 | | | 0,00031 |
| TLK2 | Homo sapiens tousled-like kinase 2 (TLK2), mRNA. | 0,6867 | 8,6292 | | 3,4059 | | 0,007156 | | | 0,03145 |
| ZBTB34 | Homo sapiens zinc finger and BTB domain containing 34 (ZBTB34), mRNA. | 0,6866 | 7,8541 | | 6,5604 | | 0,000079 | | | 0,00199 |
| DUSP6 | Homo sapiens dual specificity phosphatase 6 (DUSP6), transcript variant 1, mRNA. | 0,6866 | 8,0137 | | 3,5139 | | 0,005999 | | | 0,02763 |
| RNF216L | Homo sapiens ring finger protein 216-like (RNF216L), transcript variant 2, non-coding RNA. | 0,6864 | 9,0463 | | 5,2452 | | 0,000437 | | | 0,00500 |
| SOCS2 | Homo sapiens suppressor of cytokine signaling 2 (SOCS2), mRNA. | 0,6863 | 8,5918 | | 3,4086 | | 0,007124 | | | 0,03136 |
| ADAM17 | Homo sapiens ADAM metallopeptidase domain 17 (ADAM17), mRNA. | 0,6862 | 8,6038 | | 6,8640 | | 0,000055 | | | 0,00169 |
| ZDHHC9 | Homo sapiens zinc finger, DHHC-type containing 9 (ZDHHC9), transcript variant 1, mRNA. | 0,6855 | 9,7012 | | 5,6828 | | 0,000241 | | | 0,00361 |
| STX5 | Homo sapiens syntaxin 5 (STX5), mRNA. | 0,6854 | 8,8671 | | 5,2882 | | 0,000411 | | | 0,00485 |
| MED8 | Homo sapiens mediator of RNA polymerase II transcription, subunit 8 homolog (S. cerevisiae) (MED8), transcript variant 5, mRNA. | 0,6842 | 8,8815 | | 8,0126 | | 0,000015 | | | 0,00095 |
| PDLIM7 | Homo sapiens PDZ and LIM domain 7 (enigma) (PDLIM7), transcript variant 1, mRNA. | 0,6826 | 8,5918 | | 3,8772 | | 0,003347 | | | 0,01821 |
| ELL | Homo sapiens elongation factor RNA polymerase II (ELL), mRNA. | 0,6822 | 8,1505 | | 4,6611 | | 0,001009 | | | 0,00828 |
| TAX1BP1 | Homo sapiens Tax1 (human T-cell leukemia virus type I) binding protein 1 (TAX1BP1), transcript variant 2, mRNA. | 0,6815 | 11,3541 | | 6,2973 | | 0,000109 | | | 0,00232 |
| LOC220686 | Homo sapiens hypothetical protein LOC220686 (LOC220686), mRNA. | 0,6806 | 8,0787 | | 6,1073 | | 0,000139 | | | 0,00267 |
| COL17A1 | Homo sapiens collagen, type XVII, alpha 1 (COL17A1), mRNA. | 0,6802 | 9,5029 | | 3,0486 | | 0,012920 | | | 0,04829 |
| MTMR14 | Homo sapiens myotubularin related protein 14 (MTMR14), transcript variant 2, mRNA. | 0,6786 | 8,5092 | | 5,4760 | | 0,000318 | | | 0,00421 |
| MYD88 | Homo sapiens myeloid differentiation primary response gene (88) (MYD88), mRNA. | 0,6784 | 8,0717 | | 6,8202 | | 0,000058 | | | 0,00173 |
| RBMS1 | Homo sapiens RNA binding motif, single stranded interacting protein 1 (RBMS1), transcript variant 3, mRNA. | 0,6781 | 8,2541 | | 4,0905 | | 0,002395 | | | 0,01456 |
| ETS1 | Homo sapiens v-ets erythroblastosis virus E26 oncogene homolog 1 (avian) (ETS1), mRNA. | 0,6763 | 10,0696 | | 4,6492 | | 0,001027 | | | 0,00837 |
| PJA2 | Homo sapiens praja 2, RING-H2 motif containing (PJA2), mRNA. | 0,6762 | 9,9103 | | 5,4155 | | 0,000345 | | | 0,00436 |
| PTP4A2 | PREDICTED: Homo sapiens protein tyrosine phosphatase type IVA, member 2, transcript variant 9 (PTP4A2), mRNA. | 0,6760 | 7,8621 | | 4,5278 | | 0,001229 | | | 0,00932 |
| TLN1 | Homo sapiens talin 1 (TLN1), mRNA. | 0,6747 | 8,3204 | | 3,7729 | | 0,003951 | | | 0,02040 |
| DAPK3 | Homo sapiens death-associated protein kinase 3 (DAPK3), mRNA. | 0,6736 | 7,9980 | | 6,3295 | | 0,000105 | | | 0,00227 |
| ZC3H12C | Homo sapiens zinc finger CCCH-type containing 12C (ZC3H12C), mRNA. | 0,6734 | 8,0205 | | 5,0174 | | 0,000602 | | | 0,00602 |
| TRIM4 | Homo sapiens tripartite motif-containing 4 (TRIM4), transcript variant beta, mRNA. | 0,6730 | 9,4638 | | 4,4657 | | 0,001349 | | | 0,00993 |
| WDFY2 | Homo sapiens WD repeat and FYVE domain containing 2 (WDFY2), mRNA. | 0,6725 | 8,4284 | | 6,4957 | | 0,000085 | | | 0,00206 |
| ZNF577 | Homo sapiens zinc finger protein 577 (ZNF577), mRNA. | 0,6720 | 8,2028 | | 4,7769 | | 0,000851 | | | 0,00745 |
| MAFG | Homo sapiens v-maf musculoaponeurotic fibrosarcoma oncogene homolog G (avian) (MAFG), transcript variant 1, mRNA. | 0,6711 | 7,8775 | | 6,6587 | | 0,000070 | | | 0,00187 |
| MED8 | Homo sapiens mediator of RNA polymerase II transcription, subunit 8 homolog (S. cerevisiae) (MED8), transcript variant 3, mRNA. | 0,6707 | 8,6224 | | 9,1441 | | 0,000005 | | | 0,00059 |
| ZNF786 | Homo sapiens zinc finger protein 786 (ZNF786), mRNA. | 0,6701 | 7,9389 | | 5,2057 | | 0,000462 | | | 0,00519 |
| MET | Homo sapiens met proto-oncogene (hepatocyte growth factor receptor) (MET), transcript variant 2, mRNA. | 0,6699 | 8,9022 | | 4,1993 | | 0,002024 | | | 0,01296 |
| RHOG | Homo sapiens ras homolog gene family, member G (rho G) (RHOG), mRNA. | 0,6697 | 9,9557 | | 4,1858 | | 0,002066 | | | 0,01315 |
| GCLM | Homo sapiens glutamate-cysteine ligase, modifier subunit (GCLM), mRNA. | 0,6697 | 10,2000 | | 3,6895 | | 0,004516 | | | 0,02246 |
| OSBPL8 | Homo sapiens oxysterol binding protein-like 8 (OSBPL8), transcript variant 1, mRNA. | 0,6690 | 7,6957 | | 4,0173 | | 0,002685 | | | 0,01574 |
| UBQLN1 | Homo sapiens ubiquilin 1 (UBQLN1), transcript variant 2, mRNA. | 0,6690 | 9,5565 | | 4,1741 | | 0,002104 | | | 0,01331 |
| LOC648852 | PREDICTED: Homo sapiens hypothetical protein LOC648852 (LOC648852), mRNA. | 0,6687 | 7,9049 | | 3,5662 | | 0,005510 | | | 0,02590 |
| RAPH1 | Homo sapiens Ras association (RalGDS/AF-6) and pleckstrin homology domains 1 (RAPH1), transcript variant 1, mRNA. | 0,6687 | 8,0130 | | 3,2428 | | 0,009359 | | | 0,03788 |
| LOC100129502 | PREDICTED: Homo sapiens hypothetical protein LOC100129502 (LOC100129502), mRNA. | 0,6686 | 7,8393 | | 3,8230 | | 0,003648 | | | 0,01928 |
| PPP2R5B | Homo sapiens protein phosphatase 2, regulatory subunit B', beta isoform (PPP2R5B), mRNA. | 0,6685 | 7,7004 | | 9,6996 | | 0,000003 | | | 0,00049 |
| PSMD12 | PREDICTED: Homo sapiens proteasome (prosome, macropain) 26S subunit, non-ATPase, 12 (PSMD12), mRNA. | 0,6685 | 12,6950 | | 4,6156 | | 0,001079 | | | 0,00862 |
| OSTC | Homo sapiens oligosaccharyltransferase complex subunit (OSTC), mRNA. | 0,6682 | 9,6626 | | 4,7404 | | 0,000898 | | | 0,00771 |
| CRK | Homo sapiens v-crk sarcoma virus CT10 oncogene homolog (avian) (CRK), transcript variant II, mRNA. | 0,6668 | 8,5190 | | 3,8437 | | 0,003530 | | | 0,01887 |
| SLC25A6 | Homo sapiens solute carrier family 25 (mitochondrial carrier; adenine nucleotide translocator), member 6 (SLC25A6), nuclear gene encoding mitochondrial protein, mRNA. | 0,6668 | 9,4374 | | 8,3361 | | 0,000011 | | | 0,00083 |
| MAPKAPK2 | Homo sapiens mitogen-activated protein kinase-activated protein kinase 2 (MAPKAPK2), transcript variant 2, mRNA. | 0,6667 | 9,3857 | | 5,0259 | | 0,000595 | | | 0,00597 |
| POLD4 | Homo sapiens polymerase (DNA-directed), delta 4 (POLD4), mRNA. | 0,6663 | 8,3295 | | 4,0269 | | 0,002645 | | | 0,01558 |
| KDELR3 | Homo sapiens KDEL (Lys-Asp-Glu-Leu) endoplasmic reticulum protein retention receptor 3 (KDELR3), transcript variant 1, mRNA. | 0,6662 | 8,6931 | | 4,7605 | | 0,000872 | | | 0,00758 |
| NGEF | Homo sapiens neuronal guanine nucleotide exchange factor (NGEF), mRNA. | 0,6659 | 7,9906 | | 4,8560 | | 0,000759 | | | 0,00692 |
| LOC644745 | PREDICTED: Homo sapiens misc_RNA (LOC644745), miscRNA. | 0,6651 | 12,7684 | | 4,2673 | | 0,001823 | | | 0,01207 |
| AP1S2 | Homo sapiens adaptor-related protein complex 1, sigma 2 subunit (AP1S2), mRNA. | 0,6646 | 8,5212 | | 3,8933 | | 0,003263 | | | 0,01787 |
| ZNF223 | Homo sapiens zinc finger protein 223 (ZNF223), mRNA. | 0,6644 | 7,8787 | | 5,2666 | | 0,000424 | | | 0,00494 |
| PRNP | Homo sapiens prion protein (PRNP), transcript variant 3, mRNA. | 0,6643 | 12,6573 | | 5,9854 | | 0,000162 | | | 0,00291 |
| GJC2 | Homo sapiens gap junction protein, gamma 2, 47kDa (GJC2), mRNA. | 0,6638 | 7,5600 | | 4,1863 | | 0,002065 | | | 0,01314 |
| AUP1 | Homo sapiens ancient ubiquitous protein 1 (AUP1), mRNA. | 0,6624 | 8,8218 | | 3,1919 | | 0,010181 | | | 0,04029 |
| CRK | Homo sapiens v-crk sarcoma virus CT10 oncogene homolog (avian) (CRK), transcript variant II, mRNA. | 0,6623 | 9,9333 | | 3,9051 | | 0,003203 | | | 0,01765 |
| RNF38 | Homo sapiens ring finger protein 38 (RNF38), transcript variant 1, mRNA. | 0,6617 | 8,2794 | | 4,2658 | | 0,001827 | | | 0,01208 |
| SH2D3A | Homo sapiens SH2 domain containing 3A (SH2D3A), mRNA. | 0,6615 | 8,1763 | | 4,9536 | | 0,000659 | | | 0,00631 |
| IGF2BP2 | Homo sapiens insulin-like growth factor 2 mRNA binding protein 2 (IGF2BP2), transcript variant 1, mRNA. | 0,6605 | 11,4704 | | 5,5131 | | 0,000302 | | | 0,00409 |
| WDR26 | Homo sapiens WD repeat domain 26 (WDR26), mRNA. | 0,6603 | 8,2269 | | 3,5789 | | 0,005398 | | | 0,02551 |
| SYT7 | Homo sapiens synaptotagmin VII (SYT7), mRNA. | 0,6602 | 7,6934 | | 4,3684 | | 0,001563 | | | 0,01093 |
| AKAP13 | Homo sapiens A kinase (PRKA) anchor protein 13 (AKAP13), transcript variant 2, mRNA. | 0,6596 | 7,8533 | | 3,8903 | | 0,003279 | | | 0,01794 |
| TOR1AIP1 | Homo sapiens torsin A interacting protein 1 (TOR1AIP1), mRNA. | 0,6587 | 9,5330 | | 3,0469 | | 0,012957 | | | 0,04840 |
| PDIA5 | Homo sapiens protein disulfide isomerase family A, member 5 (PDIA5), mRNA. | 0,6587 | 8,8548 | | 4,0389 | | 0,002596 | | | 0,01540 |
| RAP1BL | Homo sapiens hCG1757335 (RAP1BL), mRNA. | 0,6583 | 8,0289 | | 7,0052 | | 0,000047 | | | 0,00154 |
| S100A13 | Homo sapiens S100 calcium binding protein A13 (S100A13), transcript variant 4, mRNA. | 0,6580 | 7,7623 | | 3,1264 | | 0,011350 | | | 0,04367 |
| SLC15A4 | Homo sapiens solute carrier family 15, member 4 (SLC15A4), mRNA. | 0,6572 | 9,0943 | | 8,9312 | | 0,000006 | | | 0,00064 |
| TINF2 | Homo sapiens TERF1 (TRF1)-interacting nuclear factor 2 (TINF2), mRNA. | 0,6570 | 8,9337 | | 4,8971 | | 0,000715 | | | 0,00663 |
| LOC400446 | PREDICTED: Homo sapiens misc_RNA (LOC400446), miscRNA. | 0,6569 | 8,2590 | | 4,1891 | | 0,002056 | | | 0,01311 |
| RNY3 | Homo sapiens RNA, Ro-associated Y3 (RNY3), small cytoplasmic RNA. | 0,6565 | 7,9845 | | 5,9792 | | 0,000163 | | | 0,00292 |
| SYPL1 | Homo sapiens synaptophysin-like 1 (SYPL1), transcript variant 1, mRNA. | 0,6554 | 10,1110 | | 3,2949 | | 0,008588 | | | 0,03573 |
| MFGE8 | Homo sapiens milk fat globule-EGF factor 8 protein (MFGE8), mRNA. | 0,6550 | 8,5985 | | 5,4596 | | 0,000325 | | | 0,00426 |
| SPPL2A | Homo sapiens signal peptide peptidase-like 2A (SPPL2A), mRNA. | 0,6548 | 8,8446 | | 3,7542 | | 0,004071 | | | 0,02087 |
| ACOT9 | Homo sapiens acyl-CoA thioesterase 9 (ACOT9), transcript variant 2, mRNA. | 0,6531 | 9,4031 | | 4,0641 | | 0,002496 | | | 0,01499 |
| PLA2G4B | Homo sapiens phospholipase A2, group IVB (cytosolic) (PLA2G4B), mRNA. | 0,6519 | 8,1845 | | 8,8850 | | 0,000006 | | | 0,00065 |
| TUBGCP2 | Homo sapiens tubulin, gamma complex associated protein 2 (TUBGCP2), mRNA. | 0,6517 | 9,2902 | | 3,6406 | | 0,004886 | | | 0,02374 |
| BTBD10 | Homo sapiens BTB (POZ) domain containing 10 (BTBD10), mRNA. | 0,6516 | 9,1324 | | 8,8727 | | 0,000006 | | | 0,00065 |
| HIC2 | Homo sapiens hypermethylated in cancer 2 (HIC2), mRNA. | 0,6505 | 8,2954 | | 4,1379 | | 0,002225 | | | 0,01381 |
| FAM89B | Homo sapiens family with sequence similarity 89, member B (FAM89B), transcript variant 3, mRNA. | 0,6497 | 8,5850 | | 4,7035 | | 0,000948 | | | 0,00800 |
| FAT1 | Homo sapiens FAT tumor suppressor homolog 1 (Drosophila) (FAT1), mRNA. | 0,6487 | 8,4004 | | 5,0549 | | 0,000571 | | | 0,00584 |
| LOC100131785 | PREDICTED: Homo sapiens misc_RNA (LOC100131785), miscRNA. | 0,6485 | 9,6174 | | 5,7347 | | 0,000225 | | | 0,00349 |
| LOC729500 | PREDICTED: Homo sapiens misc_RNA (LOC729500), partial miscRNA. | 0,6481 | 8,6016 | | 4,0758 | | 0,002451 | | | 0,01479 |
| SLC25A25 | Homo sapiens solute carrier family 25 (mitochondrial carrier; phosphate carrier), member 25 (SLC25A25), nuclear gene encoding mitochondrial protein, transcript variant 1, mRNA. | 0,6479 | 8,5316 | | 5,5086 | | 0,000304 | | | 0,00410 |
| PJA1 | Homo sapiens praja ring finger 1 (PJA1), transcript variant 2, mRNA. | 0,6473 | 9,0151 | | 5,1889 | | 0,000472 | | | 0,00526 |
| ZSWIM6 | PREDICTED: Homo sapiens zinc finger, SWIM-type containing 6 (ZSWIM6), mRNA. | 0,6459 | 7,9042 | | 7,3326 | | 0,000032 | | | 0,00129 |
| LIME1 | Homo sapiens Lck interacting transmembrane adaptor 1 (LIME1), mRNA. | 0,6455 | 8,1047 | | 5,9811 | | 0,000163 | | | 0,00292 |
| ARHGEF1 | Homo sapiens Rho guanine nucleotide exchange factor (GEF) 1 (ARHGEF1), transcript variant 2, mRNA. | 0,6453 | 8,3082 | | 3,8762 | | 0,003352 | | | 0,01823 |
| FAM18B | Homo sapiens family with sequence similarity 18, member B (FAM18B), mRNA. | 0,6452 | 9,8750 | | 4,7113 | | 0,000937 | | | 0,00796 |
| GLUD1 | Homo sapiens glutamate dehydrogenase 1 (GLUD1), mRNA. | 0,6450 | 10,2402 | | 3,8327 | | 0,003592 | | | 0,01912 |
| ELF4 | Homo sapiens E74-like factor 4 (ets domain transcription factor) (ELF4), mRNA. | 0,6446 | 9,5477 | | 3,3906 | | 0,007337 | | | 0,03202 |
| LOC388556 | PREDICTED: Homo sapiens misc_RNA (LOC388556), miscRNA. | 0,6439 | 8,6406 | | 5,8920 | | 0,000183 | | | 0,00310 |
| DENND4C | Homo sapiens DENN/MADD domain containing 4C (DENND4C), mRNA. | 0,6435 | 7,9575 | | 11,5190 | | 0,000001 | | | 0,00029 |
| TMSL3 | Homo sapiens thymosin-like 3 (TMSL3), mRNA. | 0,6430 | 10,5658 | | 4,4193 | | 0,001447 | | | 0,01040 |
| FNTA | Homo sapiens farnesyltransferase, CAAX box, alpha (FNTA), transcript variant 1, mRNA. | 0,6426 | 8,4772 | | 10,0620 | | 0,000002 | | | 0,00043 |
| HM13 | Homo sapiens histocompatibility (minor) 13 (HM13), transcript variant 2, mRNA. | 0,6421 | 8,6125 | | 7,6518 | | 0,000023 | | | 0,00113 |
| GOLGA3 | Homo sapiens golgi autoantigen, golgin subfamily a, 3 (GOLGA3), mRNA. | 0,6418 | 10,6314 | | 4,0121 | | 0,002707 | | | 0,01584 |
| SLU7 | Homo sapiens SLU7 splicing factor homolog (S. cerevisiae) (SLU7), mRNA. | 0,6408 | 8,9938 | | 3,7501 | | 0,004098 | | | 0,02095 |
| MAPK8IP3 | Homo sapiens mitogen-activated protein kinase 8 interacting protein 3 (MAPK8IP3), transcript variant 2, mRNA. | 0,6399 | 8,4705 | | 3,9305 | | 0,003077 | | | 0,01716 |
| MAFG | Homo sapiens v-maf musculoaponeurotic fibrosarcoma oncogene homolog G (avian) (MAFG), transcript variant 2, mRNA. | 0,6395 | 8,1418 | | 3,8965 | | 0,003247 | | | 0,01782 |
| CBL | Homo sapiens Cas-Br-M (murine) ecotropic retroviral transforming sequence (CBL), mRNA. | 0,6388 | 8,2009 | | 7,4740 | | 0,000027 | | | 0,00121 |
| VPS28 | Homo sapiens vacuolar protein sorting 28 homolog (S. cerevisiae) (VPS28), transcript variant 1, mRNA. | 0,6382 | 9,1870 | | 3,0291 | | 0,013348 | | | 0,04942 |
| DMTF1 | Homo sapiens cyclin D binding myb-like transcription factor 1 (DMTF1), mRNA. | 0,6380 | 8,2338 | | 4,3539 | | 0,001598 | | | 0,01107 |
| TNFSF14 | Homo sapiens tumor necrosis factor (ligand) superfamily, member 14 (TNFSF14), transcript variant 2, mRNA. | 0,6377 | 8,0004 | | 3,6222 | | 0,005033 | | | 0,02425 |
| LEP | Homo sapiens leptin (obesity homolog, mouse) (LEP), mRNA. | 0,6375 | 7,6623 | | 6,3956 | | 0,000097 | | | 0,00219 |
| FLII | Homo sapiens flightless I homolog (Drosophila) (FLII), mRNA. | 0,6375 | 8,4016 | | 4,9533 | | 0,000660 | | | 0,00631 |
| VAT1 | Homo sapiens vesicle amine transport protein 1 homolog (T. californica) (VAT1), mRNA. | 0,6353 | 8,8468 | | 3,1934 | | 0,010157 | | | 0,04022 |
| STX11 | Homo sapiens syntaxin 11 (STX11), mRNA. | 0,6347 | 7,5410 | | 5,1532 | | 0,000497 | | | 0,00539 |
| HEY1 | Homo sapiens hairy/enhancer-of-split related with YRPW motif 1 (HEY1), transcript variant 2, mRNA. | 0,6345 | 8,0386 | | 8,3955 | | 0,000010 | | | 0,00082 |
| RASA1 | Homo sapiens RAS p21 protein activator (GTPase activating protein) 1 (RASA1), transcript variant 1, mRNA. | 0,6344 | 9,6406 | | 4,7099 | | 0,000939 | | | 0,00797 |
| TDRD1 | Homo sapiens tudor domain containing 1 (TDRD1), mRNA. | 0,6342 | 7,9864 | | 4,8897 | | 0,000723 | | | 0,00668 |
| SAP30L | Homo sapiens SAP30-like (SAP30L), mRNA. | 0,6337 | 9,3935 | | 4,5645 | | 0,001164 | | | 0,00904 |
| ANG | Homo sapiens angiogenin, ribonuclease, RNase A family, 5 (ANG), transcript variant 2, mRNA. | 0,6335 | 7,7734 | | 3,8593 | | 0,003444 | | | 0,01855 |
| SNORD95 | Homo sapiens small nucleolar RNA, C/D box 95 (SNORD95), small nucleolar RNA. | 0,6329 | 7,5228 | | 5,1275 | | 0,000515 | | | 0,00550 |
| LOC200030 | Homo sapiens neuroblastoma breakpoint family, member 11-like (LOC200030), mRNA. | 0,6324 | 9,2770 | | 3,6622 | | 0,004719 | | | 0,02314 |
| ABHD5 | Homo sapiens abhydrolase domain containing 5 (ABHD5), mRNA. | 0,6322 | 7,8479 | | 3,8341 | | 0,003584 | | | 0,01909 |
| KIAA0907 | Homo sapiens KIAA0907 (KIAA0907), mRNA. | 0,6319 | 8,7075 | | 4,5536 | | 0,001183 | | | 0,00911 |
| RTTN | Homo sapiens rotatin (RTTN), mRNA. | 0,6312 | 8,4705 | | 3,4521 | | 0,006634 | | | 0,02973 |
| MYH9 | Homo sapiens myosin, heavy chain 9, non-muscle (MYH9), mRNA. | 0,6311 | 11,6337 | | 4,8632 | | 0,000751 | | | 0,00686 |
| IDS | Homo sapiens iduronate 2-sulfatase (Hunter syndrome) (IDS), transcript variant 1, mRNA. | 0,6308 | 9,3855 | | 5,2001 | | 0,000465 | | | 0,00521 |
| WDR33 | Homo sapiens WD repeat domain 33 (WDR33), transcript variant 2, mRNA. | 0,6308 | 10,0892 | | 5,8876 | | 0,000184 | | | 0,00311 |
| PTK2B | Homo sapiens PTK2B protein tyrosine kinase 2 beta (PTK2B), transcript variant 3, mRNA. | 0,6307 | 7,8561 | | 5,5613 | | 0,000283 | | | 0,00395 |
| ZNF143 | Homo sapiens zinc finger protein 143 (ZNF143), mRNA. | 0,6307 | 8,2863 | | 3,8143 | | 0,003699 | | | 0,01948 |
| BRSK1 | Homo sapiens BR serine/threonine kinase 1 (BRSK1), mRNA. | 0,6301 | 7,7257 | | 4,0877 | | 0,002406 | | | 0,01459 |
| ENTPD6 | Homo sapiens ectonucleoside triphosphate diphosphohydrolase 6 (putative function) (ENTPD6), mRNA. | 0,6300 | 8,8923 | | 4,7250 | | 0,000918 | | | 0,00784 |
| CSGALNACT2 | Homo sapiens chondroitin sulfate N-acetylgalactosaminyltransferase 2 (CSGALNACT2), mRNA. | 0,6287 | 7,6202 | | 4,1182 | | 0,002294 | | | 0,01409 |
| LOC646996 | Homo sapiens hCG2040201 (LOC646996), non-coding RNA. | 0,6282 | 8,9277 | | 3,1540 | | 0,010842 | | | 0,04216 |
| LOC644937 | PREDICTED: Homo sapiens similar to ribosomal protein L10 (LOC644937), mRNA. | 0,6282 | 8,1337 | | 3,9824 | | 0,002836 | | | 0,01630 |
| LDLR | Homo sapiens low density lipoprotein receptor (familial hypercholesterolemia) (LDLR), mRNA. | 0,6279 | 11,7333 | | 3,7310 | | 0,004225 | | | 0,02143 |
| RRAS2 | Homo sapiens related RAS viral (r-ras) oncogene homolog 2 (RRAS2), mRNA. | 0,6273 | 8,8653 | | 5,7252 | | 0,000228 | | | 0,00351 |
| LUZP1 | Homo sapiens leucine zipper protein 1 (LUZP1), mRNA. | 0,6265 | 10,1156 | | 3,8877 | | 0,003292 | | | 0,01800 |
| WWP2 | Homo sapiens WW domain containing E3 ubiquitin protein ligase 2 (WWP2), transcript variant 3, mRNA. | 0,6254 | 7,9028 | | 4,9221 | | 0,000690 | | | 0,00649 |
| ZFHX3 | Homo sapiens zinc finger homeobox 3 (ZFHX3), transcript variant A, mRNA. | 0,6249 | 7,9678 | | 4,6221 | | 0,001068 | | | 0,00856 |
| SDHALP1 | Homo sapiens succinate dehydrogenase complex, subunit A, flavoprotein pseudogene 1 (SDHALP1) on chromosome 3. | 0,6248 | 8,0684 | | 6,5385 | | 0,000081 | | | 0,00201 |
| LOC100132795 | PREDICTED: Homo sapiens misc_RNA (LOC100132795), miscRNA. | 0,6245 | 11,3735 | | 4,0793 | | 0,002437 | | | 0,01474 |
| SDCBP | Homo sapiens syndecan binding protein (syntenin) (SDCBP), transcript variant 2, mRNA. | 0,6240 | 9,0636 | | 3,6255 | | 0,005006 | | | 0,02417 |
| SLC6A9 | Homo sapiens solute carrier family 6 (neurotransmitter transporter, glycine), member 9 (SLC6A9), transcript variant 3, mRNA. | 0,6232 | 7,9720 | | 4,2422 | | 0,001895 | | | 0,01239 |
| SNAPC2 | Homo sapiens small nuclear RNA activating complex, polypeptide 2, 45kDa (SNAPC2), mRNA. | 0,6226 | 8,6909 | | 4,8354 | | 0,000782 | | | 0,00705 |
| CHMP5 | Homo sapiens chromatin modifying protein 5 (CHMP5), mRNA. | 0,6224 | 10,7502 | | 6,6385 | | 0,000072 | | | 0,00190 |
| SKIL | Homo sapiens SKI-like oncogene (SKIL), mRNA. | 0,6218 | 7,7413 | | 7,2290 | | 0,000036 | | | 0,00137 |
| ECGF1 | Homo sapiens endothelial cell growth factor 1 (platelet-derived) (ECGF1), mRNA. | 0,6202 | 7,8208 | | 10,0310 | | 0,000002 | | | 0,00044 |
| NDEL1 | Homo sapiens nudE nuclear distribution gene E homolog (A. nidulans)-like 1 (NDEL1), transcript variant 2, mRNA. | 0,6196 | 7,8117 | | 4,3178 | | 0,001688 | | | 0,01149 |
| RTKN | Homo sapiens rhotekin (RTKN), transcript variant 2, mRNA. | 0,6180 | 10,3562 | | 4,0461 | | 0,002567 | | | 0,01530 |
| SLC35C1 | Homo sapiens solute carrier family 35, member C1 (SLC35C1), mRNA. | 0,6177 | 8,5854 | | 7,2326 | | 0,000036 | | | 0,00137 |
| ITGB5 | Homo sapiens integrin, beta 5 (ITGB5), mRNA. XM_944688 XM_944693 | 0,6175 | 10,5357 | | 3,2771 | | 0,008843 | | | 0,03645 |
| RASSF1 | Homo sapiens Ras association (RalGDS/AF-6) domain family member 1 (RASSF1), transcript variant A, mRNA. | 0,6175 | 7,8798 | | 9,1163 | | 0,000005 | | | 0,00060 |
| LOC100132247 | Homo sapiens similar to Uncharacterized protein KIAA0220 (LOC100132247), mRNA. | 0,6172 | 8,8119 | | 4,9211 | | 0,000691 | | | 0,00649 |
| ACADVL | Homo sapiens acyl-Coenzyme A dehydrogenase, very long chain (ACADVL), nuclear gene encoding mitochondrial protein, transcript variant 1, mRNA. | 0,6164 | 8,6120 | | 4,7458 | | 0,000891 | | | 0,00769 |
| CSRNP2 | Homo sapiens cysteine-serine-rich nuclear protein 2 (CSRNP2), mRNA. | 0,6160 | 7,8305 | | 7,2326 | | 0,000036 | | | 0,00137 |
| FLNA | Homo sapiens filamin A, alpha (actin binding protein 280) (FLNA), mRNA. | 0,6150 | 8,6683 | | 3,7363 | | 0,004189 | | | 0,02132 |
| CLIP1 | Homo sapiens CAP-GLY domain containing linker protein 1 (CLIP1), transcript variant 1, mRNA. | 0,6148 | 8,9791 | | 4,9907 | | 0,000625 | | | 0,00613 |
| LOC100134868 | Homo sapiens hypothetical LOC100134868 (LOC100134868), non-coding RNA. | 0,6139 | 7,7882 | | 6,3051 | | 0,000108 | | | 0,00231 |
| LOC388789 | PREDICTED: Homo sapiens hypothetical gene supported by AF147354 (LOC388789), mRNA. | 0,6138 | 9,8422 | | 4,5716 | | 0,001152 | | | 0,00899 |
| DNASE1L1 | Homo sapiens deoxyribonuclease I-like 1 (DNASE1L1), transcript variant 4, mRNA. | 0,6134 | 8,5523 | | 3,7906 | | 0,003841 | | | 0,02002 |
| MAPKAPK3 | Homo sapiens mitogen-activated protein kinase-activated protein kinase 3 (MAPKAPK3), mRNA. | 0,6130 | 9,4344 | | 4,4569 | | 0,001367 | | | 0,01002 |
| RAB9A | Homo sapiens RAB9A, member RAS oncogene family (RAB9A), mRNA. | 0,6127 | 9,2220 | | 3,3076 | | 0,008409 | | | 0,03519 |
| QSOX1 | Homo sapiens quiescin Q6 sulfhydryl oxidase 1 (QSOX1), transcript variant 2, mRNA. | 0,6126 | 8,6912 | | 5,0261 | | 0,000594 | | | 0,00597 |
| RNMT | Homo sapiens RNA (guanine-7-) methyltransferase (RNMT), mRNA. | 0,6126 | 9,6242 | | 3,7071 | | 0,004390 | | | 0,02199 |
| DEF8 | Homo sapiens differentially expressed in FDCP 8 homolog (mouse) (DEF8), transcript variant 1, mRNA. | 0,6125 | 8,6811 | | 5,8231 | | 0,000200 | | | 0,00327 |
| EGFR | Homo sapiens epidermal growth factor receptor (erythroblastic leukemia viral (v-erb-b) oncogene homolog, avian) (EGFR), transcript variant 1, mRNA. | 0,6122 | 12,1758 | | 4,7304 | | 0,000911 | | | 0,00780 |
| LOC646786 | PREDICTED: Homo sapiens similar to Afadin (AF-6 protein) (LOC646786), mRNA. | 0,6116 | 8,2285 | | 6,3558 | | 0,000102 | | | 0,00225 |
| TBC1D2 | Homo sapiens TBC1 domain family, member 2 (TBC1D2), mRNA. | 0,6112 | 9,2054 | | 4,3749 | | 0,001547 | | | 0,01086 |
| RFFL | Homo sapiens ring finger and FYVE-like domain containing 1 (RFFL), transcript variant 1, mRNA. | 0,6105 | 8,0858 | | 5,8452 | | 0,000194 | | | 0,00321 |
| PLEKHG3 | Homo sapiens pleckstrin homology domain containing, family G (with RhoGef domain) member 3 (PLEKHG3), mRNA. | 0,6103 | 8,5125 | | 3,2912 | | 0,008640 | | | 0,03585 |
| LOC653103 | PREDICTED: Homo sapiens similar to Ankyrin repeat domain protein 11 (Ankyrin repeat-containing cofactor 1) (LOC653103), mRNA. | 0,6090 | 8,5928 | | 3,6908 | | 0,004507 | | | 0,02244 |
| RBBP6 | Homo sapiens retinoblastoma binding protein 6 (RBBP6), transcript variant 3, mRNA. | 0,6084 | 7,7743 | | 5,5523 | | 0,000287 | | | 0,00398 |
| LOC440353 | Homo sapiens nuclear pore complex interacting protein pseudogene (LOC440353), non-coding RNA. | 0,6083 | 10,0110 | | 4,4418 | | 0,001399 | | | 0,01016 |
| SMAGP | Homo sapiens small cell adhesion glycoprotein (SMAGP), transcript variant 2, mRNA. | 0,6081 | 8,8131 | | 4,2735 | | 0,001806 | | | 0,01201 |
| SLC2A3 | Homo sapiens solute carrier family 2 (facilitated glucose transporter), member 3 (SLC2A3), mRNA. | 0,6076 | 7,6438 | | 4,6838 | | 0,000975 | | | 0,00813 |
| DCP1A | Homo sapiens DCP1 decapping enzyme homolog A (S. cerevisiae) (DCP1A), mRNA. | 0,6074 | 8,0733 | | 4,5751 | | 0,001146 | | | 0,00896 |
| LOC100130516 | PREDICTED: Homo sapiens hypothetical protein LOC100130516 (LOC100130516), mRNA. | 0,6069 | 9,5380 | | 4,9469 | | 0,000666 | | | 0,00633 |
| POLR3A | Homo sapiens polymerase (RNA) III (DNA directed) polypeptide A, 155kDa (POLR3A), mRNA. | 0,6067 | 8,2665 | | 4,8252 | | 0,000793 | | | 0,00711 |
| SCYL1 | Homo sapiens SCY1-like 1 (S. cerevisiae) (SCYL1), mRNA. | 0,6060 | 8,5855 | | 6,6396 | | 0,000072 | | | 0,00190 |
| LOC100130070 | PREDICTED: Homo sapiens similar to metallopanstimulin (LOC100130070), mRNA. | 0,6054 | 8,9200 | | 3,9539 | | 0,002966 | | | 0,01677 |
| MGLL | Homo sapiens monoglyceride lipase (MGLL), transcript variant 1, mRNA. | 0,6041 | 7,7417 | | 3,0449 | | 0,013000 | | | 0,04849 |
| LOC391352 | PREDICTED: Homo sapiens similar to peptidylprolyl isomerase A isoform 1 (LOC391352), mRNA. | 0,6035 | 7,4623 | | 4,3769 | | 0,001543 | | | 0,01084 |
| EIF3A | Homo sapiens eukaryotic translation initiation factor 3, subunit A (EIF3A), mRNA. | 0,6020 | 9,2604 | | 4,4547 | | 0,001372 | | | 0,01004 |
| KLHL28 | Homo sapiens kelch-like 28 (Drosophila) (KLHL28), mRNA. | 0,6019 | 8,0426 | | 6,8583 | | 0,000055 | | | 0,00169 |
| SRRM2 | Homo sapiens serine/arginine repetitive matrix 2 (SRRM2), mRNA. | 0,6017 | 10,3785 | | 3,0750 | | 0,012364 | | | 0,04661 |
| VAMP4 | Homo sapiens vesicle-associated membrane protein 4 (VAMP4), mRNA. | 0,6006 | 7,8867 | | 4,8267 | | 0,000792 | | | 0,00710 |
| CPEB2 | Homo sapiens cytoplasmic polyadenylation element binding protein 2 (CPEB2), transcript variant A, mRNA. | 0,6000 | 7,6793 | | 6,0512 | | 0,000149 | | | 0,00277 |
| RRAS2 | Homo sapiens related RAS viral (r-ras) oncogene homolog 2 (RRAS2), mRNA. | 0,5999 | 9,0154 | | 5,9832 | | 0,000163 | | | 0,00292 |
| JMJD1A | Homo sapiens jumonji domain containing 1A (JMJD1A), mRNA. | 0,5991 | 7,9888 | | 3,3069 | | 0,008419 | | | 0,03521 |
| SEZ6L2 | Homo sapiens seizure related 6 homolog (mouse)-like 2 (SEZ6L2), transcript variant 2, mRNA. | 0,5989 | 8,5344 | | 6,5974 | | 0,000076 | | | 0,00194 |
| ORC6L | Homo sapiens origin recognition complex, subunit 6 like (yeast) (ORC6L), mRNA. | 0,5984 | 13,1673 | | 3,6394 | | 0,004895 | | | 0,02377 |
| TICAM1 | Homo sapiens toll-like receptor adaptor molecule 1 (TICAM1), transcript variant 2, mRNA. | 0,5982 | 7,7325 | | 4,6048 | | 0,001096 | | | 0,00871 |
| SEL1L3 | Homo sapiens sel-1 suppressor of lin-12-like 3 (C. elegans) (SEL1L3), mRNA. | 0,5977 | 8,8471 | | 3,3733 | | 0,007549 | | | 0,03265 |
| PPTC7 | Homo sapiens PTC7 protein phosphatase homolog (S. cerevisiae) (PPTC7), mRNA. | 0,5966 | 8,0946 | | 4,9909 | | 0,000625 | | | 0,00613 |
| CCL5 | Homo sapiens chemokine (C-C motif) ligand 5 (CCL5), mRNA. | 0,5962 | 7,5180 | | 4,4357 | | 0,001412 | | | 0,01022 |
| CCNK | Homo sapiens cyclin K (CCNK), transcript variant 2, mRNA. | 0,5954 | 9,8813 | | 4,9157 | | 0,000696 | | | 0,00652 |
| F2RL1 | Homo sapiens coagulation factor II (thrombin) receptor-like 1 (F2RL1), mRNA. | 0,5952 | 8,5728 | | 7,9517 | | 0,000016 | | | 0,00097 |
| CENTA1 | Homo sapiens centaurin, alpha 1 (CENTA1), mRNA. | 0,5951 | 10,2738 | | 4,1455 | | 0,002199 | | | 0,01372 |
| NUP50 | Homo sapiens nucleoporin 50kDa (NUP50), transcript variant 2, mRNA. | 0,5948 | 8,6292 | | 3,4586 | | 0,006565 | | | 0,02952 |
| dJ341D10.1 | Homo sapiens dJ341D10.1 (novel protein) (dJ341D10.1), mRNA. | 0,5947 | 7,8560 | | 3,0921 | | 0,012016 | | | 0,04564 |
| CLDN7 | Homo sapiens claudin 7 (CLDN7), mRNA. | 0,5946 | 7,8121 | | 4,4647 | | 0,001351 | | | 0,00994 |
| PVRL1 | Homo sapiens poliovirus receptor-related 1 (herpesvirus entry mediator C) (PVRL1), transcript variant 3, mRNA. | 0,5945 | 7,7912 | | 3,6481 | | 0,004827 | | | 0,02352 |
| TMEM51 | Homo sapiens transmembrane protein 51 (TMEM51), mRNA. | 0,5944 | 9,9931 | | 6,0176 | | 0,000156 | | | 0,00284 |
| KCTD13 | Homo sapiens potassium channel tetramerisation domain containing 13 (KCTD13), mRNA. | 0,5940 | 8,3212 | | 7,9509 | | 0,000016 | | | 0,00097 |
| MYO3B | Homo sapiens myosin IIIB (MYO3B), mRNA. | 0,5937 | 7,6758 | | 4,7954 | | 0,000828 | | | 0,00732 |
| SSTR2 | Homo sapiens somatostatin receptor 2 (SSTR2), mRNA. | 0,5931 | 7,6464 | | 5,1779 | | 0,000480 | | | 0,00531 |
| OCIAD2 | Homo sapiens OCIA domain containing 2 (OCIAD2), transcript variant 1, mRNA. | 0,5929 | 11,8717 | | 4,2475 | | 0,001879 | | | 0,01233 |
| PLEKHN1 | Homo sapiens pleckstrin homology domain containing, family N member 1 (PLEKHN1), mRNA. | 0,5929 | 7,7657 | | 3,7284 | | 0,004243 | | | 0,02149 |
| LRRFIP2 | Homo sapiens leucine rich repeat (in FLII) interacting protein 2 (LRRFIP2), transcript variant 2, mRNA. | 0,5927 | 9,0470 | | 5,1486 | | 0,000500 | | | 0,00541 |
| SNHG5 | Homo sapiens small nucleolar RNA host gene (non-protein coding) 5 (SNHG5) on chromosome 6. | 0,5918 | 12,7585 | | 8,9097 | | 0,000006 | | | 0,00064 |
| MGC70857 | Homo sapiens similar to RIKEN cDNA C030006K11 gene (MGC70857), mRNA. | 0,5916 | 8,4904 | | 4,3463 | | 0,001616 | | | 0,01115 |
| ACOT9 | Homo sapiens acyl-CoA thioesterase 9 (ACOT9), transcript variant 1, mRNA. | 0,5913 | 8,3308 | | 3,7347 | | 0,004200 | | | 0,02136 |
| CLIC4 | Homo sapiens chloride intracellular channel 4 (CLIC4), nuclear gene encoding mitochondrial protein, mRNA. | 0,5902 | 7,9254 | | 4,8194 | | 0,000800 | | | 0,00714 |
| TPD52L2 | Homo sapiens tumor protein D52-like 2 (TPD52L2), transcript variant 2, mRNA. | 0,5897 | 9,0014 | | 5,6102 | | 0,000265 | | | 0,00383 |
| CCNYL1 | Homo sapiens cyclin Y-like 1 (CCNYL1), mRNA. | 0,5896 | 8,2063 | | 3,9229 | | 0,003114 | | | 0,01729 |
| SPTLC1 | Homo sapiens serine palmitoyltransferase, long chain base subunit 1 (SPTLC1), transcript variant 2, mRNA. | 0,5894 | 8,8613 | | 3,5788 | | 0,005399 | | | 0,02551 |
| LOC643336 | PREDICTED: Homo sapiens similar to hCG1985303 (LOC643336), mRNA. | 0,5892 | 10,1964 | | 3,5779 | | 0,005407 | | | 0,02553 |
| WSB1 | Homo sapiens WD repeat and SOCS box-containing 1 (WSB1), transcript variant 3, mRNA. | 0,5889 | 7,7817 | | 3,1713 | | 0,010535 | | | 0,04128 |
| SYS1 | Homo sapiens SYS1 Golgi-localized integral membrane protein homolog (S. cerevisiae) (SYS1), transcript variant 1, mRNA. | 0,5888 | 8,2139 | | 5,7653 | | 0,000216 | | | 0,00340 |
| TMEM170A | Homo sapiens transmembrane protein 170A (TMEM170A), mRNA. | 0,5887 | 8,2401 | | 6,7003 | | 0,000067 | | | 0,00183 |
| ERCC1 | Homo sapiens excision repair cross-complementing rodent repair deficiency, complementation group 1 (includes overlapping antisense sequence) (ERCC1), transcript variant 2, mRNA. | 0,5886 | 9,7939 | | 3,1328 | | 0,011231 | | | 0,04330 |
| GCLC | Homo sapiens glutamate-cysteine ligase, catalytic subunit (GCLC), mRNA. | 0,5885 | 8,9908 | | 3,2857 | | 0,008719 | | | 0,03608 |
| FAM127A | Homo sapiens family with sequence similarity 127, member A (FAM127A), mRNA. | 0,5882 | 9,9147 | | 3,3948 | | 0,007286 | | | 0,03183 |
| TNFRSF10A | Homo sapiens tumor necrosis factor receptor superfamily, member 10a (TNFRSF10A), mRNA. | 0,5880 | 8,2992 | | 4,5939 | | 0,001114 | | | 0,00879 |
| PTPRF | Homo sapiens protein tyrosine phosphatase, receptor type, F (PTPRF), transcript variant 1, mRNA. | 0,5874 | 11,0671 | | 3,7923 | | 0,003831 | | | 0,01998 |
| BCAP29 | Homo sapiens B-cell receptor-associated protein 29 (BCAP29), transcript variant 2, mRNA. | 0,5873 | 8,6015 | | 3,4423 | | 0,006741 | | | 0,03010 |
| IFNGR1 | Homo sapiens interferon gamma receptor 1 (IFNGR1), mRNA. | 0,5870 | 8,9624 | | 5,7462 | | 0,000221 | | | 0,00345 |
| C9orf89 | Homo sapiens chromosome 9 open reading frame 89 (C9orf89), mRNA. | 0,5860 | 9,2373 | | 4,8302 | | 0,000788 | | | 0,00708 |
| LOC643167 | PREDICTED: Homo sapiens misc_RNA (LOC643167), miscRNA. | 0,5855 | 9,4194 | | 8,6036 | | 0,000008 | | | 0,00074 |
| FBXO11 | Homo sapiens F-box protein 11 (FBXO11), transcript variant 1, mRNA. | 0,5853 | 8,8259 | | 4,5181 | | 0,001247 | | | 0,00941 |
| NELF | Homo sapiens nasal embryonic LHRH factor (NELF), mRNA. | 0,5853 | 8,5570 | | 6,8149 | | 0,000058 | | | 0,00173 |
| NCOA3 | Homo sapiens nuclear receptor coactivator 3 (NCOA3), transcript variant 1, mRNA. | 0,5849 | 8,7214 | | 3,1005 | | 0,011850 | | | 0,04510 |
| STX7 | Homo sapiens syntaxin 7 (STX7), mRNA. | 0,5845 | 8,1945 | | 5,0498 | | 0,000575 | | | 0,00586 |
| WDR1 | Homo sapiens WD repeat domain 1 (WDR1), transcript variant 1, mRNA. | 0,5835 | 8,6667 | | 3,6391 | | 0,004897 | | | 0,02377 |
| RXRB | Homo sapiens retinoid X receptor, beta (RXRB), mRNA. | 0,5833 | 8,3272 | | 3,7281 | | 0,004244 | | | 0,02149 |
| RAB8B | Homo sapiens RAB8B, member RAS oncogene family (RAB8B), mRNA. | 0,5830 | 8,1167 | | 5,4410 | | 0,000334 | | | 0,00431 |
| TMEM167B | Homo sapiens transmembrane protein 167B (TMEM167B), mRNA. | 0,5829 | 8,3766 | | 6,3784 | | 0,000099 | | | 0,00222 |
| STAM | Homo sapiens signal transducing adaptor molecule (SH3 domain and ITAM motif) 1 (STAM), mRNA. | 0,5827 | 8,4064 | | 5,0362 | | 0,000586 | | | 0,00593 |
| BTG3 | Homo sapiens BTG family, member 3 (BTG3), mRNA. | 0,5826 | 9,3694 | | 4,5122 | | 0,001259 | | | 0,00947 |
| AHR | Homo sapiens aryl hydrocarbon receptor (AHR), mRNA. | 0,5824 | 10,0052 | | 4,5681 | | 0,001158 | | | 0,00901 |
| CDR2L | Homo sapiens cerebellar degeneration-related protein 2-like (CDR2L), mRNA. | 0,5822 | 8,6696 | | 8,6583 | | 0,000008 | | | 0,00073 |
| PIP4K2C | Homo sapiens phosphatidylinositol-5-phosphate 4-kinase, type II, gamma (PIP4K2C), mRNA. | 0,5820 | 8,6839 | | 3,5284 | | 0,005859 | | | 0,02718 |
| LRRC37B | Homo sapiens leucine rich repeat containing 37B (LRRC37B), mRNA. | 0,5818 | 8,1107 | | 4,4066 | | 0,001475 | | | 0,01053 |
| NBPF8 | PREDICTED: Homo sapiens neuroblastoma breakpoint family, member 8 (NBPF8), mRNA. | 0,5817 | 7,7707 | | 3,2104 | | 0,009874 | | | 0,03940 |
| ARRDC2 | Homo sapiens arrestin domain containing 2 (ARRDC2), transcript variant 1, mRNA. | 0,5816 | 7,8070 | | 4,8174 | | 0,000802 | | | 0,00716 |
| WAC | Homo sapiens WW domain containing adaptor with coiled-coil (WAC), transcript variant 1, mRNA. | 0,5815 | 9,9100 | | 6,6931 | | 0,000067 | | | 0,00184 |
| ZMIZ2 | Homo sapiens zinc finger, MIZ-type containing 2 (ZMIZ2), transcript variant 1, mRNA. | 0,5815 | 7,7351 | | 7,8343 | | 0,000019 | | | 0,00103 |
| ACSL4 | Homo sapiens acyl-CoA synthetase long-chain family member 4 (ACSL4), transcript variant 1, mRNA. | 0,5815 | 9,3272 | | 3,4262 | | 0,006921 | | | 0,03069 |
| RTN4R | Homo sapiens reticulon 4 receptor (RTN4R), mRNA. | 0,5811 | 7,9020 | | 7,3150 | | 0,000033 | | | 0,00130 |
| MYC | Homo sapiens v-myc myelocytomatosis viral oncogene homolog (avian) (MYC), mRNA. | 0,5804 | 10,5176 | | 3,6461 | | 0,004843 | | | 0,02357 |
| PELO | Homo sapiens pelota homolog (Drosophila) (PELO), mRNA. | 0,5796 | 10,5102 | | 3,0284 | | 0,013363 | | | 0,04943 |
| PPP2CB | Homo sapiens protein phosphatase 2 (formerly 2A), catalytic subunit, beta isoform (PPP2CB), transcript variant 1, mRNA. | 0,5790 | 9,8959 | | 5,5794 | | 0,000277 | | | 0,00389 |
| F2RL1 | Homo sapiens coagulation factor II (thrombin) receptor-like 1 (F2RL1), mRNA. | 0,5770 | 8,3548 | | 8,0466 | | 0,000015 | | | 0,00095 |
| TFG | Homo sapiens TRK-fused gene (TFG), transcript variant 1, mRNA. | 0,5766 | 11,2837 | | 6,7934 | | 0,000060 | | | 0,00175 |
| CUTL1 | Homo sapiens cut-like 1, CCAAT displacement protein (Drosophila) (CUTL1), transcript variant 1, mRNA. | 0,5764 | 8,6242 | | 3,4375 | | 0,006794 | | | 0,03029 |
| TMCC3 | Homo sapiens transmembrane and coiled-coil domain family 3 (TMCC3), mRNA. | 0,5743 | 7,5405 | | 3,7527 | | 0,004081 | | | 0,02089 |
| LRRC8E | Homo sapiens leucine rich repeat containing 8 family, member E (LRRC8E), mRNA. | 0,5740 | 7,8018 | | 7,2520 | | 0,000035 | | | 0,00135 |
| LOC729120 | PREDICTED: Homo sapiens hypothetical LOC729120 (LOC729120), mRNA. | 0,5739 | 7,9663 | | 4,1151 | | 0,002305 | | | 0,01414 |
| LOC651202 | PREDICTED: Homo sapiens similar to large subunit ribosomal protein L36a (LOC651202), mRNA. | 0,5736 | 11,4157 | | 4,1830 | | 0,002075 | | | 0,01318 |
| USP3 | Homo sapiens ubiquitin specific peptidase 3 (USP3), mRNA. | 0,5726 | 8,9043 | | 5,0936 | | 0,000540 | | | 0,00566 |
| STK19 | Homo sapiens serine/threonine kinase 19 (STK19), transcript variant 1, mRNA. | 0,5725 | 8,7731 | | 3,6752 | | 0,004621 | | | 0,02281 |
| GMPPA | Homo sapiens GDP-mannose pyrophosphorylase A (GMPPA), transcript variant 2, mRNA. | 0,5723 | 9,7078 | | 5,8285 | | 0,000199 | | | 0,00326 |
| COPB1 | Homo sapiens coatomer protein complex, subunit beta 1 (COPB1), mRNA. | 0,5723 | 10,5366 | | 4,6422 | | 0,001037 | | | 0,00841 |
| IDS | Homo sapiens iduronate 2-sulfatase (Hunter syndrome) (IDS), transcript variant 2, mRNA. | 0,5720 | 9,0959 | | 3,3404 | | 0,007967 | | | 0,03388 |
| PHLDB2 | Homo sapiens pleckstrin homology-like domain, family B, member 2 (PHLDB2), mRNA. | 0,5719 | 8,3706 | | 3,0468 | | 0,012960 | | | 0,04840 |
| ABR | Homo sapiens active BCR-related gene (ABR), transcript variant 2, mRNA. | 0,5713 | 9,2328 | | 3,4127 | | 0,007076 | | | 0,03120 |
| SLC26A6 | Homo sapiens solute carrier family 26, member 6 (SLC26A6), transcript variant 3, mRNA. | 0,5712 | 8,6254 | | 4,6350 | | 0,001048 | | | 0,00846 |
| RSL24D1 | Homo sapiens ribosomal L24 domain containing 1 (RSL24D1), mRNA. | 0,5701 | 10,8338 | | 6,4184 | | 0,000094 | | | 0,00216 |
| HIST1H2BG | Homo sapiens histone cluster 1, H2bg (HIST1H2BG), mRNA. | 0,5701 | 7,4829 | | 7,5205 | | 0,000026 | | | 0,00120 |
| MMD | Homo sapiens monocyte to macrophage differentiation-associated (MMD), mRNA. | 0,5690 | 9,3949 | | 4,7800 | | 0,000847 | | | 0,00743 |
| PPP4R1 | Homo sapiens protein phosphatase 4, regulatory subunit 1 (PPP4R1), transcript variant 2, mRNA. | 0,5689 | 11,0882 | | 3,1756 | | 0,010460 | | | 0,04106 |
| ARFGEF1 | Homo sapiens ADP-ribosylation factor guanine nucleotide-exchange factor 1(brefeldin A-inhibited) (ARFGEF1), mRNA. | 0,5687 | 8,6317 | | 8,3419 | | 0,000011 | | | 0,00083 |
| GADD45B | Homo sapiens growth arrest and DNA-damage-inducible, beta (GADD45B), mRNA. | 0,5681 | 8,0706 | | 4,3938 | | 0,001504 | | | 0,01066 |
| RHBDF2 | Homo sapiens rhomboid 5 homolog 2 (Drosophila) (RHBDF2), transcript variant 2, mRNA. | 0,5679 | 9,0817 | | 4,3267 | | 0,001665 | | | 0,01137 |
| LOC644590 | PREDICTED: Homo sapiens similar to EVIN1 (LOC644590), mRNA. | 0,5677 | 7,7594 | | 4,7832 | | 0,000843 | | | 0,00742 |
| CEP27 | Homo sapiens centrosomal protein 27kDa (CEP27), mRNA. | 0,5667 | 7,8606 | | 4,6725 | | 0,000992 | | | 0,00821 |
| LOC651149 | PREDICTED: Homo sapiens similar to 60S ribosomal protein L3 (L4) (LOC651149), mRNA. | 0,5665 | 9,2872 | | 5,5068 | | 0,000305 | | | 0,00411 |
| MIB2 | Homo sapiens mindbomb homolog 2 (Drosophila) (MIB2), mRNA. | 0,5652 | 8,3823 | | 4,8248 | | 0,000794 | | | 0,00711 |
| FOXJ2 | Homo sapiens forkhead box J2 (FOXJ2), mRNA. | 0,5644 | 9,1272 | | 3,6949 | | 0,004477 | | | 0,02234 |
| CNBP | Homo sapiens CCHC-type zinc finger, nucleic acid binding protein (CNBP), transcript variant 3, mRNA. | 0,5641 | 7,4572 | | 5,2279 | | 0,000447 | | | 0,00508 |
| ATG9A | Homo sapiens ATG9 autophagy related 9 homolog A (S. cerevisiae) (ATG9A), transcript variant 1, mRNA. | 0,5633 | 8,4536 | | 5,7999 | | 0,000206 | | | 0,00332 |
| KIFAP3 | Homo sapiens kinesin-associated protein 3 (KIFAP3), mRNA. | 0,5633 | 8,1813 | | 4,3839 | | 0,001526 | | | 0,01078 |
| LOC729500 | PREDICTED: Homo sapiens misc_RNA (LOC729500), partial miscRNA. | 0,5630 | 9,7613 | | 3,7709 | | 0,003964 | | | 0,02044 |
| BAT2D1 | Homo sapiens BAT2 domain containing 1 (BAT2D1), mRNA. | 0,5624 | 9,1530 | | 3,7726 | | 0,003953 | | | 0,02040 |
| SRGAP1 | Homo sapiens SLIT-ROBO Rho GTPase activating protein 1 (SRGAP1), mRNA. | 0,5619 | 7,7854 | | 6,1907 | | 0,000125 | | | 0,00249 |
| GTF2IRD1 | Homo sapiens GTF2I repeat domain containing 1 (GTF2IRD1), transcript variant 1, mRNA. | 0,5613 | 8,0465 | | 7,1914 | | 0,000038 | | | 0,00140 |
| RSPH3 | Homo sapiens radial spoke 3 homolog (Chlamydomonas) (RSPH3), mRNA. | 0,5611 | 7,9235 | | 5,1138 | | 0,000525 | | | 0,00556 |
| USO1 | Homo sapiens USO1 homolog, vesicle docking protein (yeast) (USO1), mRNA. | 0,5608 | 9,3723 | | 3,9953 | | 0,002779 | | | 0,01607 |
| RRBP1 | Homo sapiens ribosome binding protein 1 homolog 180kDa (dog) (RRBP1), transcript variant 1, mRNA. | 0,5595 | 10,5099 | | 3,1997 | | 0,010050 | | | 0,03994 |
| DOPEY2 | Homo sapiens dopey family member 2 (DOPEY2), mRNA. | 0,5594 | 7,8974 | | 6,9865 | | 0,000048 | | | 0,00156 |
| MBTD1 | Homo sapiens mbt domain containing 1 (MBTD1), mRNA. | 0,5589 | 7,7486 | | 4,9313 | | 0,000681 | | | 0,00643 |
| ZBTB17 | Homo sapiens zinc finger and BTB domain containing 17 (ZBTB17), mRNA. | 0,5588 | 8,1708 | | 6,1257 | | 0,000135 | | | 0,00263 |
| ZCCHC9 | Homo sapiens zinc finger, CCHC domain containing 9 (ZCCHC9), mRNA. | 0,5588 | 9,5100 | | 4,3088 | | 0,001711 | | | 0,01159 |
| TMEM57 | Homo sapiens transmembrane protein 57 (TMEM57), mRNA. | 0,5580 | 7,8220 | | 4,3536 | | 0,001598 | | | 0,01107 |
| CDK7 | Homo sapiens cyclin-dependent kinase 7 (MO15 homolog, Xenopus laevis, cdk-activating kinase) (CDK7), mRNA. | 0,5576 | 9,7507 | | 7,9306 | | 0,000017 | | | 0,00098 |
| FAM114A1 | Homo sapiens family with sequence similarity 114, member A1 (FAM114A1), mRNA. | 0,5565 | 8,1077 | | 6,2988 | | 0,000109 | | | 0,00232 |
| FBRS | Homo sapiens fibrosin (FBRS), mRNA. | 0,5557 | 9,6246 | | 3,9141 | | 0,003158 | | | 0,01747 |
| DVL1 | Homo sapiens dishevelled, dsh homolog 1 (Drosophila) (DVL1), transcript variant 2, mRNA. | 0,5555 | 7,8863 | | 4,3740 | | 0,001550 | | | 0,01086 |
| UBTD2 | Homo sapiens ubiquitin domain containing 2 (UBTD2), mRNA. | 0,5550 | 7,9718 | | 3,9561 | | 0,002955 | | | 0,01674 |
| NEU1 | Homo sapiens sialidase 1 (lysosomal sialidase) (NEU1), mRNA. | 0,5548 | 8,7719 | | 3,2883 | | 0,008682 | | | 0,03596 |
| SMTN | Homo sapiens smoothelin (SMTN), transcript variant 3, mRNA. | 0,5546 | 8,1042 | | 3,2845 | | 0,008735 | | | 0,03612 |
| RNASE4 | Homo sapiens ribonuclease, RNase A family, 4 (RNASE4), transcript variant 1, mRNA. | 0,5544 | 7,7458 | | 3,7210 | | 0,004293 | | | 0,02167 |
| ADAM8 | Homo sapiens ADAM metallopeptidase domain 8 (ADAM8), mRNA. | 0,5544 | 7,5937 | | 6,2903 | | 0,000110 | | | 0,00234 |
| MICALCL | Homo sapiens MICAL C-terminal like (MICALCL), mRNA. | 0,5541 | 7,6459 | | 12,0040 | | 0,000000 | | | 0,00026 |
| TRIM11 | Homo sapiens tripartite motif-containing 11 (TRIM11), mRNA. | 0,5539 | 8,4524 | | 3,6666 | | 0,004685 | | | 0,02302 |
| SYAP1 | Homo sapiens synapse associated protein 1, SAP47 homolog (Drosophila) (SYAP1), mRNA. | 0,5537 | 8,0512 | | 4,0413 | | 0,002586 | | | 0,01537 |
| GPRC5C | Homo sapiens G protein-coupled receptor, family C, group 5, member C (GPRC5C), transcript variant 2, mRNA. | 0,5536 | 7,8735 | | 3,3056 | | 0,008437 | | | 0,03526 |
| FAM18B | Homo sapiens family with sequence similarity 18, member B (FAM18B), mRNA. | 0,5532 | 8,8194 | | 4,1979 | | 0,002028 | | | 0,01297 |
| CCNYL1 | Homo sapiens cyclin Y-like 1 (CCNYL1), mRNA. | 0,5529 | 8,1610 | | 4,4273 | | 0,001430 | | | 0,01030 |
| SLK | Homo sapiens STE20-like kinase (yeast) (SLK), mRNA. | 0,5523 | 8,6282 | | 3,1360 | | 0,011170 | | | 0,04313 |
| KLHL36 | Homo sapiens kelch-like 36 (Drosophila) (KLHL36), mRNA. | 0,5522 | 9,1462 | | 4,8921 | | 0,000720 | | | 0,00666 |
| HECTD1 | Homo sapiens HECT domain containing 1 (HECTD1), mRNA. | 0,5521 | 9,6197 | | 4,0609 | | 0,002508 | | | 0,01505 |
| RNF181 | Homo sapiens ring finger protein 181 (RNF181), mRNA. | 0,5517 | 10,6452 | | 5,4698 | | 0,000321 | | | 0,00423 |
| TYMP | Homo sapiens thymidine phosphorylase (TYMP), transcript variant 3, mRNA. | 0,5512 | 7,8508 | | 5,9117 | | 0,000178 | | | 0,00306 |
| LCOR | Homo sapiens ligand dependent nuclear receptor corepressor (LCOR), mRNA. | 0,5509 | 8,2624 | | 8,3496 | | 0,000011 | | | 0,00083 |
| BCAP29 | Homo sapiens B-cell receptor-associated protein 29 (BCAP29), transcript variant 1, mRNA. | 0,5503 | 8,5716 | | 3,0914 | | 0,012031 | | | 0,04566 |
| TFAP2C | Homo sapiens transcription factor AP-2 gamma (activating enhancer binding protein 2 gamma) (TFAP2C), mRNA. | 0,5502 | 9,5917 | | 3,4188 | | 0,007006 | | | 0,03097 |
| LOC728672 | PREDICTED: Homo sapiens misc_RNA (LOC728672), miscRNA. | 0,5502 | 12,8017 | | 7,4929 | | 0,000027 | | | 0,00121 |
| TSC1 | Homo sapiens tuberous sclerosis 1 (TSC1), transcript variant 1, mRNA. | 0,5498 | 8,1771 | | 3,7466 | | 0,004121 | | | 0,02106 |
| TMEM206 | Homo sapiens transmembrane protein 206 (TMEM206), mRNA. | 0,5498 | 8,5142 | | 4,5179 | | 0,001248 | | | 0,00941 |
| IBTK | Homo sapiens inhibitor of Bruton agammaglobulinemia tyrosine kinase (IBTK), mRNA. | 0,5495 | 9,0065 | | 5,9798 | | 0,000163 | | | 0,00292 |
| DERL2 | Homo sapiens Der1-like domain family, member 2 (DERL2), mRNA. | 0,5495 | 8,5637 | | 4,4584 | | 0,001364 | | | 0,01001 |
| TPRG1L | Homo sapiens tumor protein p63 regulated 1-like (TPRG1L), mRNA. | 0,5491 | 10,4857 | | 3,7375 | | 0,004181 | | | 0,02129 |
| ATP2B4 | Homo sapiens ATPase, Ca++ transporting, plasma membrane 4 (ATP2B4), transcript variant 1, mRNA. | 0,5486 | 7,5222 | | 4,7813 | | 0,000846 | | | 0,00742 |
| AVL9 | Homo sapiens AVL9 homolog (S. cerevisiase) (AVL9), mRNA. | 0,5484 | 8,0664 | | 7,2819 | | 0,000034 | | | 0,00134 |
| OSTC | Homo sapiens oligosaccharyltransferase complex subunit (OSTC), mRNA. | 0,5483 | 11,3477 | | 4,2254 | | 0,001944 | | | 0,01262 |
| PPP4R4 | Homo sapiens protein phosphatase 4, regulatory subunit 4 (PPP4R4), transcript variant 2, mRNA. | 0,5478 | 7,5316 | | 5,1226 | | 0,000519 | | | 0,00552 |
| LOC387825 | PREDICTED: Homo sapiens misc_RNA (LOC387825), miscRNA. | 0,5472 | 8,8254 | | 6,5766 | | 0,000077 | | | 0,00198 |
| LOC100131336 | PREDICTED: Homo sapiens misc_RNA (LOC100131336), miscRNA. | 0,5471 | 10,0458 | | 3,1760 | | 0,010454 | | | 0,04104 |
| FRAT2 | Homo sapiens frequently rearranged in advanced T-cell lymphomas 2 (FRAT2), mRNA. | 0,5468 | 8,6521 | | 3,2157 | | 0,009787 | | | 0,03914 |
| TDP1 | Homo sapiens tyrosyl-DNA phosphodiesterase 1 (TDP1), transcript variant 1, mRNA. | 0,5460 | 9,8662 | | 3,4287 | | 0,006893 | | | 0,03060 |
| C7orf47 | Homo sapiens chromosome 7 open reading frame 47 (C7orf47), mRNA. | 0,5457 | 9,7026 | | 4,0050 | | 0,002737 | | | 0,01596 |
| RUFY3 | Homo sapiens RUN and FYVE domain containing 3 (RUFY3), transcript variant 1, mRNA. | 0,5456 | 8,0264 | | 9,0709 | | 0,000005 | | | 0,00061 |
| PHF3 | Homo sapiens PHD finger protein 3 (PHF3), mRNA. | 0,5455 | 9,9210 | | 4,2745 | | 0,001803 | | | 0,01200 |
| ZNF526 | Homo sapiens zinc finger protein 526 (ZNF526), mRNA. | 0,5453 | 8,0995 | | 3,8932 | | 0,003264 | | | 0,01787 |
| SNORA73A | Homo sapiens small nucleolar RNA, H/ACA box 73A (SNORA73A), small nucleolar RNA. | 0,5452 | 7,8443 | | 3,2119 | | 0,009849 | | | 0,03932 |
| SLC22A4 | Homo sapiens solute carrier family 22 (organic cation/ergothioneine transporter), member 4 (SLC22A4), mRNA. | 0,5450 | 7,7754 | | 6,3101 | | 0,000107 | | | 0,00231 |
| NUCB2 | Homo sapiens nucleobindin 2 (NUCB2), mRNA. | 0,5448 | 8,5310 | | 4,8072 | | 0,000814 | | | 0,00724 |
| GPR137B | Homo sapiens G protein-coupled receptor 137B (GPR137B), mRNA. | 0,5448 | 8,1587 | | 3,6029 | | 0,005192 | | | 0,02479 |
| METRNL | Homo sapiens meteorin, glial cell differentiation regulator-like (METRNL), mRNA. | 0,5436 | 7,4966 | | 5,3085 | | 0,000400 | | | 0,00477 |
| LOC440704 | PREDICTED: Homo sapiens hypothetical gene supported by BC042042 (LOC440704), mRNA. | 0,5430 | 8,5087 | | 4,6521 | | 0,001022 | | | 0,00834 |
| ATL3 | Homo sapiens atlastin GTPase 3 (ATL3), mRNA. | 0,5427 | 9,9206 | | 3,0996 | | 0,011867 | | | 0,04516 |
| FNDC3B | Homo sapiens fibronectin type III domain containing 3B (FNDC3B), transcript variant 1, mRNA. | 0,5423 | 10,2298 | | 4,9699 | | 0,000644 | | | 0,00623 |
| UBQLN1 | Homo sapiens ubiquilin 1 (UBQLN1), transcript variant 2, mRNA. | 0,5421 | 8,5906 | | 4,2123 | | 0,001984 | | | 0,01281 |
| HCCA2 | Homo sapiens HCCA2 protein (HCCA2), mRNA. | 0,5419 | 7,9121 | | 3,8137 | | 0,003702 | | | 0,01950 |
| LOC338758 | PREDICTED: Homo sapiens hypothetical protein LOC338758 (LOC338758), mRNA. | 0,5402 | 7,7447 | | 5,7428 | | 0,000222 | | | 0,00346 |
| WDR19 | Homo sapiens WD repeat domain 19 (WDR19), mRNA. | 0,5399 | 8,0221 | | 5,3458 | | 0,000380 | | | 0,00459 |
| DSE | Homo sapiens dermatan sulfate epimerase (DSE), transcript variant 1, mRNA. | 0,5398 | 10,2077 | | 3,9726 | | 0,002880 | | | 0,01648 |
| GEM | Homo sapiens GTP binding protein overexpressed in skeletal muscle (GEM), transcript variant 2, mRNA. | 0,5392 | 7,5666 | | 3,5758 | | 0,005425 | | | 0,02559 |
| GABPB2 | Homo sapiens GA binding protein transcription factor, beta subunit 2 (GABPB2), transcript variant gamma-2, mRNA. | 0,5390 | 8,3823 | | 6,5227 | | 0,000083 | | | 0,00203 |
| PHLDA3 | Homo sapiens pleckstrin homology-like domain, family A, member 3 (PHLDA3), mRNA. | 0,5389 | 9,2657 | | 3,1454 | | 0,010997 | | | 0,04261 |
| LPAR3 | Homo sapiens lysophosphatidic acid receptor 3 (LPAR3), mRNA. | 0,5380 | 8,1838 | | 4,7054 | | 0,000945 | | | 0,00800 |
| SNORA25 | Homo sapiens small nucleolar RNA, H/ACA box 25 (SNORA25), small nucleolar RNA. | 0,5379 | 8,0271 | | 4,5971 | | 0,001109 | | | 0,00878 |
| CLK1 | Homo sapiens CDC-like kinase 1 (CLK1), mRNA. | 0,5375 | 7,7053 | | 3,9187 | | 0,003135 | | | 0,01737 |
| GK | Homo sapiens glycerol kinase (GK), transcript variant 2, mRNA. | 0,5372 | 7,7086 | | 3,8668 | | 0,003403 | | | 0,01839 |
| EIF5A2 | Homo sapiens eukaryotic translation initiation factor 5A2 (EIF5A2), mRNA. | 0,5370 | 8,3876 | | 3,2225 | | 0,009678 | | | 0,03878 |
| EIF2AK3 | Homo sapiens eukaryotic translation initiation factor 2-alpha kinase 3 (EIF2AK3), mRNA. | 0,5369 | 7,7754 | | 8,0103 | | 0,000015 | | | 0,00095 |
| ZNF419 | Homo sapiens zinc finger protein 419 (ZNF419), transcript variant 6, mRNA. | 0,5364 | 8,4068 | | 3,2686 | | 0,008968 | | | 0,03681 |
| LOC645693 | PREDICTED: Homo sapiens misc_RNA (LOC645693), miscRNA. | 0,5361 | 8,3669 | | 4,9693 | | 0,000645 | | | 0,00623 |
| LZTR1 | Homo sapiens leucine-zipper-like transcription regulator 1 (LZTR1), mRNA. | 0,5359 | 9,9154 | | 3,1562 | | 0,010803 | | | 0,04206 |
| CORO1B | Homo sapiens coronin, actin binding protein, 1B (CORO1B), transcript variant 1, mRNA. | 0,5349 | 8,1897 | | 3,9672 | | 0,002904 | | | 0,01658 |
| ILK | Homo sapiens integrin-linked kinase (ILK), transcript variant 2, mRNA. | 0,5348 | 8,1008 | | 4,3927 | | 0,001506 | | | 0,01068 |
| ZFAND2B | Homo sapiens zinc finger, AN1-type domain 2B (ZFAND2B), mRNA. | 0,5337 | 8,8411 | | 4,3909 | | 0,001510 | | | 0,01070 |
| ARHGEF1 | Homo sapiens Rho guanine nucleotide exchange factor (GEF) 1 (ARHGEF1), transcript variant 1, mRNA. | 0,5337 | 8,3849 | | 3,6292 | | 0,004977 | | | 0,02407 |
| PROCR | Homo sapiens protein C receptor, endothelial (EPCR) (PROCR), mRNA. | 0,5335 | 8,8003 | | 3,0433 | | 0,013035 | | | 0,04857 |
| PDGFB | Homo sapiens platelet-derived growth factor beta polypeptide (simian sarcoma viral (v-sis) oncogene homolog) (PDGFB), transcript variant 1, mRNA. | 0,5333 | 8,2833 | | 4,0532 | | 0,002538 | | | 0,01519 |
| KCNN4 | Homo sapiens potassium intermediate/small conductance calcium-activated channel, subfamily N, member 4 (KCNN4), mRNA. | 0,5331 | 7,7955 | | 3,8463 | | 0,003516 | | | 0,01881 |
| TRPC4AP | Homo sapiens transient receptor potential cation channel, subfamily C, member 4 associated protein (TRPC4AP), transcript variant 1, mRNA. | 0,5328 | 9,3430 | | 4,6999 | | 0,000953 | | | 0,00803 |
| FAM39DP | Homo sapiens family with sequence similarity 39, member D pseudogene (FAM39DP) on chromosome 15. | 0,5327 | 8,7083 | | 3,3482 | | 0,007865 | | | 0,03365 |
| TIAF1 | Homo sapiens TGFB1-induced anti-apoptotic factor 1 (TIAF1), mRNA. | 0,5325 | 9,7168 | | 3,1688 | | 0,010578 | | | 0,04138 |
| LOC100133607 | PREDICTED: Homo sapiens hypothetical protein LOC100133607, transcript variant 2 (LOC100133607), mRNA. | 0,5325 | 13,0205 | | 3,7868 | | 0,003864 | | | 0,02010 |
| UGP2 | Homo sapiens UDP-glucose pyrophosphorylase 2 (UGP2), transcript variant 1, mRNA. | 0,5324 | 7,8021 | | 4,1496 | | 0,002185 | | | 0,01366 |
| VPS37C | Homo sapiens vacuolar protein sorting 37 homolog C (S. cerevisiae) (VPS37C), mRNA. | 0,5314 | 9,7447 | | 4,0134 | | 0,002701 | | | 0,01581 |
| POFUT2 | Homo sapiens protein O-fucosyltransferase 2 (POFUT2), transcript variant 3, mRNA. | 0,5311 | 9,0858 | | 6,0633 | | 0,000147 | | | 0,00276 |
| POFUT2 | Homo sapiens protein O-fucosyltransferase 2 (POFUT2), transcript variant 3, mRNA. | 0,5309 | 9,3196 | | 4,8163 | | 0,000804 | | | 0,00717 |
| LOC146517 | PREDICTED: Homo sapiens hypothetical protein LOC146517 (LOC146517), mRNA. | 0,5308 | 8,7433 | | 3,5738 | | 0,005443 | | | 0,02565 |
| ZFPM1 | Homo sapiens zinc finger protein, multitype 1 (ZFPM1), mRNA. | 0,5305 | 8,6212 | | 5,6032 | | 0,000268 | | | 0,00385 |
| C2orf30 | Homo sapiens chromosome 2 open reading frame 30 (C2orf30), mRNA. | 0,5302 | 9,5606 | | 8,3408 | | 0,000011 | | | 0,00083 |
| NR2C2 | Homo sapiens nuclear receptor subfamily 2, group C, member 2 (NR2C2), mRNA. | 0,5300 | 7,7475 | | 4,5591 | | 0,001173 | | | 0,00909 |
| GPRC5A | Homo sapiens G protein-coupled receptor, family C, group 5, member A (GPRC5A), mRNA. | 0,5297 | 7,8887 | | 3,6128 | | 0,005110 | | | 0,02451 |
| IL4R | Homo sapiens interleukin 4 receptor (IL4R), transcript variant 1, mRNA. | 0,5297 | 8,6757 | | 4,1256 | | 0,002268 | | | 0,01399 |
| PDCD7 | Homo sapiens programmed cell death 7 (PDCD7), mRNA. | 0,5289 | 13,0478 | | 3,3666 | | 0,007632 | | | 0,03292 |
| GNA13 | Homo sapiens guanine nucleotide binding protein (G protein), alpha 13 (GNA13), mRNA. | 0,5285 | 9,3885 | | 3,9496 | | 0,002986 | | | 0,01683 |
| RNU105A | Homo sapiens RNA, U105A small nucleolar (RNU105A), small nucleolar RNA. | 0,5284 | 7,8675 | | 3,5413 | | 0,005737 | | | 0,02672 |
| LOC730990 | PREDICTED: Homo sapiens hypothetical LOC730990 (LOC730990), mRNA. | 0,5282 | 7,7804 | | 4,0780 | | 0,002442 | | | 0,01476 |
| CAST | Homo sapiens calpastatin (CAST), transcript variant 11, mRNA. | 0,5280 | 8,7242 | | 5,3276 | | 0,000390 | | | 0,00469 |
| WDR33 | Homo sapiens WD repeat domain 33 (WDR33), transcript variant 2, mRNA. | 0,5276 | 8,0414 | | 6,9525 | | 0,000050 | | | 0,00160 |
| EDG4 | Homo sapiens endothelial differentiation, lysophosphatidic acid G-protein-coupled receptor, 4 (EDG4), mRNA. | 0,5273 | 9,0208 | | 9,4026 | | 0,000004 | | | 0,00052 |
| XPNPEP3 | Homo sapiens X-prolyl aminopeptidase (aminopeptidase P) 3, putative (XPNPEP3), mRNA. | 0,5272 | 13,0800 | | 3,9703 | | 0,002890 | | | 0,01653 |
| TNIP2 | Homo sapiens TNFAIP3 interacting protein 2 (TNIP2), mRNA. | 0,5271 | 8,0795 | | 5,6857 | | 0,000240 | | | 0,00360 |
| JARID1A | Homo sapiens Jumonji, AT rich interactive domain 1A (RBBP2-like) (JARID1A), mRNA. | 0,5261 | 8,2347 | | 3,2830 | | 0,008757 | | | 0,03618 |
| RN7SL1 | Homo sapiens RNA, 7SL, cytoplasmic 1 (RN7SL1), small cytoplasmic RNA. | 0,5260 | 13,1336 | | 4,2182 | | 0,001966 | | | 0,01272 |
| GOLT1B | Homo sapiens golgi transport 1 homolog B (S. cerevisiae) (GOLT1B), mRNA. | 0,5260 | 8,7232 | | 3,8901 | | 0,003280 | | | 0,01795 |
| PVRL2 | Homo sapiens poliovirus receptor-related 2 (herpesvirus entry mediator B) (PVRL2), transcript variant alpha, mRNA. | 0,5256 | 8,3137 | | 5,4300 | | 0,000339 | | | 0,00434 |
| SERP1 | Homo sapiens stress-associated endoplasmic reticulum protein 1 (SERP1), mRNA. | 0,5254 | 9,0825 | | 3,4577 | | 0,006574 | | | 0,02954 |
| IRF7 | Homo sapiens interferon regulatory factor 7 (IRF7), transcript variant b, mRNA. | 0,5248 | 7,5550 | | 3,9009 | | 0,003224 | | | 0,01773 |
| LOC648921 | PREDICTED: Homo sapiens similar to LOC283693 protein (LOC648921), mRNA. | 0,5244 | 7,7856 | | 4,1405 | | 0,002216 | | | 0,01377 |
| RFNG | PREDICTED: Homo sapiens radical fringe homolog (Drosophila) (RFNG), mRNA. | 0,5240 | 8,9648 | | 3,8022 | | 0,003771 | | | 0,01974 |
| RHOA | Homo sapiens ras homolog gene family, member A (RHOA), mRNA. | 0,5236 | 11,6970 | | 3,6807 | | 0,004580 | | | 0,02268 |
| NMD3 | Homo sapiens NMD3 homolog (S. cerevisiae) (NMD3), mRNA. | 0,5236 | 9,4038 | | 6,6113 | | 0,000074 | | | 0,00192 |
| TMEM214 | Homo sapiens transmembrane protein 214 (TMEM214), transcript variant 1, mRNA. | 0,5231 | 9,2088 | | 4,0552 | | 0,002530 | | | 0,01517 |
| PHF1 | Homo sapiens PHD finger protein 1 (PHF1), transcript variant 2, mRNA. | 0,5224 | 7,8595 | | 3,3317 | | 0,008082 | | | 0,03423 |
| LOC100134537 | PREDICTED: Homo sapiens misc_RNA (LOC100134537), miscRNA. | 0,5220 | 9,8862 | | 4,2555 | | 0,001856 | | | 0,01221 |
| TANK | Homo sapiens TRAF family member-associated NFKB activator (TANK), transcript variant 1, mRNA. | 0,5220 | 8,0821 | | 4,2295 | | 0,001932 | | | 0,01256 |
| LOC728772 | PREDICTED: Homo sapiens similar to transmembrane protein 106A, transcript variant 1 (LOC728772), mRNA. | 0,5216 | 8,1586 | | 4,7041 | | 0,000947 | | | 0,00800 |
| DUSP16 | Homo sapiens dual specificity phosphatase 16 (DUSP16), mRNA. | 0,5216 | 7,6807 | | 7,7828 | | 0,000020 | | | 0,00106 |
| ARFGAP1 | Homo sapiens ADP-ribosylation factor GTPase activating protein 1 (ARFGAP1), transcript variant 2, mRNA. | 0,5215 | 7,7841 | | 5,7101 | | 0,000232 | | | 0,00354 |
| RABGEF1 | Homo sapiens RAB guanine nucleotide exchange factor (GEF) 1 (RABGEF1), mRNA. | 0,5214 | 8,0672 | | 4,4552 | | 0,001371 | | | 0,01004 |
| SPRYD3 | Homo sapiens SPRY domain containing 3 (SPRYD3), mRNA. | 0,5209 | 8,2916 | | 6,7072 | | 0,000066 | | | 0,00183 |
| SHOC2 | Homo sapiens soc-2 suppressor of clear homolog (C. elegans) (SHOC2), mRNA. | 0,5208 | 8,5948 | | 3,3308 | | 0,008095 | | | 0,03426 |
| CHKB | Homo sapiens choline kinase beta (CHKB), transcript variant 1, mRNA. | 0,5205 | 8,0241 | | 4,3054 | | 0,001720 | | | 0,01164 |
| SSR2 | PREDICTED: Homo sapiens signal sequence receptor, beta (translocon-associated protein beta), transcript variant 4 (SSR2), mRNA. | 0,5204 | 8,0342 | | 7,2396 | | 0,000036 | | | 0,00136 |
| NAMPT | Homo sapiens nicotinamide phosphoribosyltransferase (NAMPT), mRNA. | 0,5197 | 7,8120 | | 5,2530 | | 0,000432 | | | 0,00498 |
| LOC389662 | PREDICTED: Homo sapiens misc_RNA (LOC389662), miscRNA. | 0,5192 | 8,2953 | | 3,7236 | | 0,004276 | | | 0,02160 |
| SLC33A1 | Homo sapiens solute carrier family 33 (acetyl-CoA transporter), member 1 (SLC33A1), mRNA. | 0,5187 | 8,8201 | | 5,3435 | | 0,000381 | | | 0,00460 |
| TDRD7 | Homo sapiens tudor domain containing 7 (TDRD7), mRNA. | 0,5182 | 8,2849 | | 4,1916 | | 0,002048 | | | 0,01307 |
| OCIAD2 | Homo sapiens OCIA domain containing 2 (OCIAD2), transcript variant 2, mRNA. | 0,5178 | 10,0268 | | 3,1674 | | 0,010603 | | | 0,04146 |
| MOAP1 | Homo sapiens modulator of apoptosis 1 (MOAP1), mRNA. | 0,5177 | 8,0264 | | 3,1505 | | 0,010905 | | | 0,04236 |
| RAB35 | Homo sapiens RAB35, member RAS oncogene family (RAB35), mRNA. | 0,5176 | 8,8357 | | 3,9115 | | 0,003171 | | | 0,01751 |
| LYST | Homo sapiens lysosomal trafficking regulator (LYST), mRNA. | 0,5176 | 7,6917 | | 4,3361 | | 0,001641 | | | 0,01127 |
| MZF1 | Homo sapiens myeloid zinc finger 1 (MZF1), transcript variant 2, mRNA. | 0,5167 | 7,8084 | | 3,3821 | | 0,007440 | | | 0,03229 |
| LOC653566 | Homo sapiens similar to Signal peptidase complex subunit 2 (Microsomal signal peptidase 25 kDa subunit) (SPase 25 kDa subunit) (LOC653566), mRNA. | 0,5166 | 8,2767 | | 3,7338 | | 0,004206 | | | 0,02138 |
| POLR3A | Homo sapiens polymerase (RNA) III (DNA directed) polypeptide A, 155kDa (POLR3A), mRNA. | 0,5166 | 7,9159 | | 5,0160 | | 0,000603 | | | 0,00603 |
| ARNTL | Homo sapiens aryl hydrocarbon receptor nuclear translocator-like (ARNTL), transcript variant 2, mRNA. | 0,5160 | 7,7681 | | 5,1248 | | 0,000517 | | | 0,00551 |
| ARG2 | Homo sapiens arginase, type II (ARG2), nuclear gene encoding mitochondrial protein, mRNA. | 0,5158 | 7,4816 | | 4,8888 | | 0,000724 | | | 0,00669 |
| ATG9A | Homo sapiens ATG9 autophagy related 9 homolog A (S. cerevisiae) (ATG9A), transcript variant 1, mRNA. | 0,5148 | 8,7126 | | 3,0532 | | 0,012823 | | | 0,04800 |
| DNAJC3 | Homo sapiens DnaJ (Hsp40) homolog, subfamily C, member 3 (DNAJC3), mRNA. | 0,5144 | 7,7405 | | 4,0144 | | 0,002697 | | | 0,01580 |
| UBXN1 | Homo sapiens UBX domain protein 1 (UBXN1), mRNA. | 0,5140 | 9,7450 | | 3,3827 | | 0,007433 | | | 0,03229 |
| EML2 | Homo sapiens echinoderm microtubule associated protein like 2 (EML2), mRNA. | 0,5137 | 7,8606 | | 6,2508 | | 0,000116 | | | 0,00241 |
| TRAF1 | Homo sapiens TNF receptor-associated factor 1 (TRAF1), mRNA. | 0,5136 | 7,4813 | | 3,9764 | | 0,002863 | | | 0,01640 |
| BRI3 | Homo sapiens brain protein I3 (BRI3), mRNA. | 0,5133 | 11,0048 | | 6,2383 | | 0,000118 | | | 0,00243 |
| FAM40B | Homo sapiens family with sequence similarity 40, member B (FAM40B), mRNA. | 0,5126 | 7,6498 | | 3,6945 | | 0,004480 | | | 0,02235 |
| PTP4A2 | PREDICTED: Homo sapiens protein tyrosine phosphatase type IVA, member 2, transcript variant 5 (PTP4A2), mRNA. | 0,5125 | 7,6342 | | 5,9412 | | 0,000172 | | | 0,00301 |
| SLC41A3 | Homo sapiens solute carrier family 41, member 3 (SLC41A3), transcript variant 1, mRNA. | 0,5116 | 8,5765 | | 3,5064 | | 0,006072 | | | 0,02790 |
| LOC728888 | PREDICTED: Homo sapiens similar to Protein KIAA0220 (LOC728888), mRNA. | 0,5115 | 9,6510 | | 4,0303 | | 0,002631 | | | 0,01555 |
| RPS27 | Homo sapiens ribosomal protein S27 (metallopanstimulin 1) (RPS27), mRNA. | 0,5112 | 11,3712 | | 3,4103 | | 0,007103 | | | 0,03128 |
| FAM65A | Homo sapiens family with sequence similarity 65, member A (FAM65A), mRNA. | 0,5106 | 8,7122 | | 3,6738 | | 0,004632 | | | 0,02284 |
| RAB31 | Homo sapiens RAB31, member RAS oncogene family (RAB31), mRNA. | 0,5106 | 11,8704 | | 4,4564 | | 0,001368 | | | 0,01002 |
| MTMR11 | Homo sapiens myotubularin related protein 11 (MTMR11), mRNA. | 0,5106 | 8,3887 | | 3,4918 | | 0,006219 | | | 0,02835 |
| PNPLA2 | Homo sapiens patatin-like phospholipase domain containing 2 (PNPLA2), mRNA. | 0,5104 | 8,2675 | | 6,9267 | | 0,000051 | | | 0,00163 |
| FBXO45 | Homo sapiens F-box protein 45 (FBXO45), mRNA. | 0,5103 | 8,1605 | | 5,2591 | | 0,000428 | | | 0,00496 |
| SUV420H1 | Homo sapiens suppressor of variegation 4-20 homolog 1 (Drosophila) (SUV420H1), transcript variant 2, mRNA. | 0,5091 | 8,1854 | | 3,0488 | | 0,012917 | | | 0,04829 |
| DENR | Homo sapiens density-regulated protein (DENR), mRNA. | 0,5087 | 7,7184 | | 4,0852 | | 0,002415 | | | 0,01464 |
| SHANK3 | Homo sapiens SH3 and multiple ankyrin repeat domains 3 (SHANK3), mRNA. | 0,5085 | 7,8262 | | 4,8123 | | 0,000808 | | | 0,00720 |
| SLC6A10P | Homo sapiens solute carrier family 6 (neurotransmitter transporter, creatine), member 10 (pseudogene) (SLC6A10P) on chromosome 16. | 0,5075 | 7,7112 | | 3,6097 | | 0,005136 | | | 0,02457 |
| RPS6KC1 | Homo sapiens ribosomal protein S6 kinase, 52kDa, polypeptide 1 (RPS6KC1), mRNA. | 0,5069 | 8,0611 | | 4,4600 | | 0,001361 | | | 0,00999 |
| LRWD1 | Homo sapiens leucine-rich repeats and WD repeat domain containing 1 (LRWD1), mRNA. | 0,5066 | 8,6082 | | 6,2755 | | 0,000112 | | | 0,00237 |
| LAPTM4A | Homo sapiens lysosomal-associated protein transmembrane 4 alpha (LAPTM4A), mRNA. | 0,5065 | 8,7400 | | 5,0883 | | 0,000544 | | | 0,00568 |
| STK24 | Homo sapiens serine/threonine kinase 24 (STE20 homolog, yeast) (STK24), transcript variant 1, mRNA. | 0,5065 | 11,9452 | | 5,0400 | | 0,000583 | | | 0,00591 |
| SGPP2 | PREDICTED: Homo sapiens sphingosine-1-phosphate phosphotase 2 (SGPP2), mRNA. | 0,5064 | 7,5553 | | 3,6833 | | 0,004561 | | | 0,02262 |
| HEATR5B | Homo sapiens HEAT repeat containing 5B (HEATR5B), mRNA. | 0,5064 | 8,1409 | | 8,6805 | | 0,000008 | | | 0,00073 |
| LOC644907 | Homo sapiens hCG18290 (LOC644907), mRNA. | 0,5064 | 10,6434 | | 3,8530 | | 0,003478 | | | 0,01868 |
| SEC14L1 | Homo sapiens SEC14-like 1 (S. cerevisiae) (SEC14L1), transcript variant 1, mRNA. | 0,5063 | 7,7162 | | 4,5368 | | 0,001213 | | | 0,00925 |
| SLK | Homo sapiens STE20-like kinase (yeast) (SLK), mRNA. | 0,5062 | 8,9091 | | 3,4390 | | 0,006778 | | | 0,03023 |
| ABI1 | Homo sapiens abl-interactor 1 (ABI1), transcript variant 3, mRNA. | 0,5059 | 7,9750 | | 8,5215 | | 0,000009 | | | 0,00078 |
| LOC646821 | PREDICTED: Homo sapiens similar to beta-actin (LOC646821), mRNA. | 0,5056 | 8,0612 | | 6,6943 | | 0,000067 | | | 0,00184 |
| LOC388339 | PREDICTED: Homo sapiens similar to ribosomal protein, transcript variant 4 (LOC388339), mRNA. | 0,5050 | 10,9183 | | 5,4666 | | 0,000322 | | | 0,00423 |
| FLJ20254 | Homo sapiens hypothetical protein FLJ20254 (FLJ20254), mRNA. | 0,5048 | 8,7432 | | 3,4391 | | 0,006777 | | | 0,03023 |
| LOC440157 | Homo sapiens hypothetical gene supported by AK096951; BC066547 (LOC440157), mRNA. | 0,5040 | 7,6900 | | 4,4782 | | 0,001324 | | | 0,00982 |
| STX4 | Homo sapiens syntaxin 4 (STX4), mRNA. | 0,5038 | 8,6044 | | 5,1006 | | 0,000535 | | | 0,00562 |
| SSR2 | Homo sapiens signal sequence receptor, beta (translocon-associated protein beta) (SSR2), mRNA. | 0,5035 | 9,9142 | | 7,1403 | | 0,000040 | | | 0,00144 |
| ASXL2 | Homo sapiens additional sex combs like 2 (Drosophila) (ASXL2), mRNA. | 0,5035 | 8,3765 | | 6,0524 | | 0,000149 | | | 0,00277 |
| DVL3 | Homo sapiens dishevelled, dsh homolog 3 (Drosophila) (DVL3), mRNA. | 0,5030 | 8,9555 | | 3,2916 | | 0,008633 | | | 0,03585 |
| FNDC3B | Homo sapiens fibronectin type III domain containing 3B (FNDC3B), transcript variant 2, mRNA. | 0,5027 | 10,1396 | | 3,1883 | | 0,010242 | | | 0,04047 |
| IL28RA | Homo sapiens interleukin 28 receptor, alpha (interferon, lambda receptor) (IL28RA), transcript variant 1, mRNA. | 0,5024 | 7,6861 | | 4,5062 | | 0,001270 | | | 0,00951 |
| CCR6 | Homo sapiens chemokine (C-C motif) receptor 6 (CCR6), transcript variant 2, mRNA. | 0,5024 | 12,9372 | | 4,4050 | | 0,001479 | | | 0,01055 |
| TP53INP1 | Homo sapiens tumor protein p53 inducible nuclear protein 1 (TP53INP1), mRNA. | 0,5023 | 7,7612 | | 3,2396 | | 0,009408 | | | 0,03803 |
| PAOX | Homo sapiens polyamine oxidase (exo-N4-amino) (PAOX), transcript variant 5, mRNA. | 0,5023 | 7,9888 | | 4,1053 | | 0,002341 | | | 0,01431 |
| OSBP2 | Homo sapiens oxysterol binding protein 2 (OSBP2), transcript variant 1, mRNA. | 0,5022 | 7,4369 | | 4,9936 | | 0,000623 | | | 0,00613 |
| SRP54 | Homo sapiens signal recognition particle 54kDa (SRP54), mRNA. | 0,5019 | 10,6042 | | 3,2519 | | 0,009219 | | | 0,03748 |
| LSR | Homo sapiens lipolysis stimulated lipoprotein receptor (LSR), transcript variant 3, mRNA. | 0,5018 | 8,5286 | | 3,6232 | | 0,005025 | | | 0,02423 |
| TMEM2 | Homo sapiens transmembrane protein 2 (TMEM2), mRNA. | 0,5018 | 8,4593 | | 3,3899 | | 0,007345 | | | 0,03204 |
| EHBP1 | Homo sapiens EH domain binding protein 1 (EHBP1), mRNA. | 0,5014 | 9,1497 | | 4,2347 | | 0,001917 | | | 0,01249 |
| INF2 | Homo sapiens inverted formin, FH2 and WH2 domain containing (INF2), transcript variant 3, mRNA. | 0,5011 | 7,8829 | | 3,3985 | | 0,007243 | | | 0,03167 |
| UBE2H | Homo sapiens ubiquitin-conjugating enzyme E2H (UBC8 homolog, yeast) (UBE2H), transcript variant 1, mRNA. | 0,5009 | 8,0512 | | 3,2369 | | 0,009451 | | | 0,03815 |
| PICALM | Homo sapiens phosphatidylinositol binding clathrin assembly protein (PICALM), transcript variant 1, mRNA. | 0,5005 | 10,3000 | | 3,9798 | | 0,002847 | | | 0,01634 |
| FAM189B | Homo sapiens family with sequence similarity 189, member B (FAM189B), transcript variant 1, mRNA. | -0,5002 | 9,1103 | | -5,2850 | | 0,000413 | | | 0,00486 |
| GPS1 | Homo sapiens G protein pathway suppressor 1 (GPS1), transcript variant 2, mRNA. | -0,5004 | 9,7392 | | -5,0664 | | 0,000561 | | | 0,00579 |
| NLRP2 | Homo sapiens NLR family, pyrin domain containing 2 (NLRP2), mRNA. | -0,5007 | 8,2277 | | -5,8008 | | 0,000206 | | | 0,00332 |
| VPS29 | Homo sapiens vacuolar protein sorting 29 homolog (S. cerevisiae) (VPS29), transcript variant 1, mRNA. | -0,5008 | 10,9669 | | -4,2014 | | 0,002017 | | | 0,01294 |
| DCK | Homo sapiens deoxycytidine kinase (DCK), mRNA. | -0,5011 | 8,1621 | | -4,2071 | | 0,002000 | | | 0,01286 |
| BID | Homo sapiens BH3 interacting domain death agonist (BID), transcript variant 1, mRNA. | -0,5020 | 8,1657 | | -4,6720 | | 0,000993 | | | 0,00821 |
| SC65 | Homo sapiens synaptonemal complex protein SC65 (SC65), mRNA. | -0,5021 | 7,7164 | | -3,6778 | | 0,004601 | | | 0,02275 |
| GPR177 | Homo sapiens G protein-coupled receptor 177 (GPR177), transcript variant 2, mRNA. | -0,5023 | 7,7942 | | -9,9538 | | 0,000002 | | | 0,00045 |
| UCRC | Homo sapiens ubiquinol-cytochrome c reductase complex (7.2 kD) (UCRC), transcript variant 2, mRNA. | -0,5026 | 8,7287 | | -4,6927 | | 0,000963 | | | 0,00807 |
| PLK2 | Homo sapiens polo-like kinase 2 (Drosophila) (PLK2), mRNA. | -0,5037 | 7,5785 | | -5,3242 | | 0,000391 | | | 0,00470 |
| CLUAP1 | Homo sapiens clusterin associated protein 1 (CLUAP1), transcript variant 2, mRNA. | -0,5038 | 8,0588 | | -4,2103 | | 0,001990 | | | 0,01282 |
| SMC3 | Homo sapiens structural maintenance of chromosomes 3 (SMC3), mRNA. | -0,5045 | 9,4385 | | -6,3448 | | 0,000103 | | | 0,00226 |
| LOC100133372 | PREDICTED: Homo sapiens misc_RNA (LOC100133372), miscRNA. | -0,5046 | 11,1998 | | -4,5433 | | 0,001201 | | | 0,00920 |
| ALG14 | Homo sapiens asparagine-linked glycosylation 14 homolog (S. cerevisiae) (ALG14), mRNA. | -0,5051 | 7,8120 | | -8,6300 | | 0,000008 | | | 0,00073 |
| PMS2L4 | Homo sapiens postmeiotic segregation increased 2-like 4 pseudogene (PMS2L4), non-coding RNA. | -0,5054 | 9,0960 | | -4,9156 | | 0,000696 | | | 0,00652 |
| HIGD1A | Homo sapiens HIG1 hypoxia inducible domain family, member 1A (HIGD1A), transcript variant 1, mRNA. | -0,5054 | 10,4777 | | -3,9119 | | 0,003169 | | | 0,01751 |
| COX5A | Homo sapiens cytochrome c oxidase subunit Va (COX5A), nuclear gene encoding mitochondrial protein, mRNA. | -0,5060 | 11,7117 | | -3,6176 | | 0,005071 | | | 0,02437 |
| MRPL32 | Homo sapiens mitochondrial ribosomal protein L32 (MRPL32), nuclear gene encoding mitochondrial protein, mRNA. | -0,5060 | 10,2153 | | -3,3607 | | 0,007706 | | | 0,03316 |
| FAM69A | Homo sapiens family with sequence similarity 69, member A (FAM69A), mRNA. | -0,5063 | 8,5241 | | -5,2497 | | 0,000434 | | | 0,00498 |
| AGK | Homo sapiens acylglycerol kinase (AGK), mRNA. | -0,5066 | 8,0440 | | -4,9565 | | 0,000657 | | | 0,00630 |
| CFH | Homo sapiens complement factor H (CFH), transcript variant 2, mRNA. | -0,5067 | 7,7346 | | -3,1886 | | 0,010237 | | | 0,04046 |
| ACD | Homo sapiens adrenocortical dysplasia homolog (mouse) (ACD), transcript variant 2, mRNA. | -0,5072 | 8,6159 | | -5,9944 | | 0,000160 | | | 0,00289 |
| PRMT5 | Homo sapiens protein arginine methyltransferase 5 (PRMT5), transcript variant 2, mRNA. | -0,5074 | 9,3502 | | -3,1747 | | 0,010475 | | | 0,04111 |
| SEPHS2 | Homo sapiens selenophosphate synthetase 2 (SEPHS2), mRNA. | -0,5075 | 9,0883 | | -3,8849 | | 0,003307 | | | 0,01805 |
| PHF14 | Homo sapiens PHD finger protein 14 (PHF14), transcript variant 1, mRNA. | -0,5076 | 8,3441 | | -4,3677 | | 0,001564 | | | 0,01093 |
| NT5DC1 | Homo sapiens 5'-nucleotidase domain containing 1 (NT5DC1), mRNA. | -0,5080 | 7,8048 | | -5,5875 | | 0,000274 | | | 0,00387 |
| FBL | Homo sapiens fibrillarin (FBL), mRNA. | -0,5081 | 10,4582 | | -5,0545 | | 0,000571 | | | 0,00584 |
| LOC100133489 | PREDICTED: Homo sapiens similar to hCG1983233 (LOC100133489), mRNA. | -0,5089 | 7,5947 | | -7,4123 | | 0,000029 | | | 0,00125 |
| PPIE | Homo sapiens peptidylprolyl isomerase E (cyclophilin E) (PPIE), transcript variant 1, mRNA. | -0,5089 | 8,6598 | | -3,4522 | | 0,006633 | | | 0,02973 |
| ZDHHC6 | Homo sapiens zinc finger, DHHC-type containing 6 (ZDHHC6), mRNA. | -0,5090 | 9,1206 | | -3,2470 | | 0,009293 | | | 0,03767 |
| CDC23 | Homo sapiens cell division cycle 23 homolog (S. cerevisiae) (CDC23), mRNA. | -0,5094 | 9,2254 | | -4,6107 | | 0,001087 | | | 0,00867 |
| RQCD1 | Homo sapiens RCD1 required for cell differentiation1 homolog (S. pombe) (RQCD1), mRNA. | -0,5094 | 8,9189 | | -6,3660 | | 0,000100 | | | 0,00224 |
| C6orf66 | Homo sapiens chromosome 6 open reading frame 66 (C6orf66), mRNA. | -0,5095 | 8,2184 | | -3,8669 | | 0,003402 | | | 0,01839 |
| THAP10 | Homo sapiens THAP domain containing 10 (THAP10), mRNA. | -0,5099 | 7,8531 | | -5,1362 | | 0,000509 | | | 0,00546 |
| INCENP | Homo sapiens inner centromere protein antigens 135/155kDa (INCENP), transcript variant 1, mRNA. | -0,5100 | 7,7788 | | -5,4049 | | 0,000350 | | | 0,00439 |
| LOC729057 | PREDICTED: Homo sapiens misc_RNA (LOC729057), miscRNA. | -0,5103 | 7,8234 | | -5,2599 | | 0,000428 | | | 0,00495 |
| LOC100128266 | PREDICTED: Homo sapiens misc_RNA (LOC100128266), miscRNA. | -0,5106 | 11,1531 | | -4,0035 | | 0,002744 | | | 0,01598 |
| TMEM138 | Homo sapiens transmembrane protein 138 (TMEM138), mRNA. | -0,5108 | 8,1158 | | -4,1445 | | 0,002203 | | | 0,01373 |
| C12orf45 | Homo sapiens chromosome 12 open reading frame 45 (C12orf45), mRNA. | -0,5113 | 8,4865 | | -3,8935 | | 0,003262 | | | 0,01787 |
| IPO9 | Homo sapiens importin 9 (IPO9), mRNA. | -0,5113 | 8,2240 | | -4,7659 | | 0,000865 | | | 0,00755 |
| N-PAC | Homo sapiens cytokine-like nuclear factor n-pac (N-PAC), mRNA. | -0,5114 | 8,3599 | | -8,1019 | | 0,000014 | | | 0,00093 |
| TMBIM4 | Homo sapiens transmembrane BAX inhibitor motif containing 4 (TMBIM4), mRNA. | -0,5114 | 10,1540 | | -3,2417 | | 0,009376 | | | 0,03793 |
| ARL6IP6 | Homo sapiens ADP-ribosylation-like factor 6 interacting protein 6 (ARL6IP6), mRNA. | -0,5115 | 9,4602 | | -4,3357 | | 0,001642 | | | 0,01127 |
| UBE2L6 | Homo sapiens ubiquitin-conjugating enzyme E2L 6 (UBE2L6), transcript variant 1, mRNA. | -0,5120 | 7,9943 | | -3,2455 | | 0,009317 | | | 0,03775 |
| MLEC | Homo sapiens malectin (MLEC), mRNA. | -0,5121 | 9,2119 | | -3,6224 | | 0,005032 | | | 0,02425 |
| EEF2K | Homo sapiens eukaryotic elongation factor-2 kinase (EEF2K), mRNA. | -0,5121 | 8,0890 | | -4,2658 | | 0,001827 | | | 0,01208 |
| ELAC2 | Homo sapiens elaC homolog 2 (E. coli) (ELAC2), mRNA. | -0,5124 | 8,0128 | | -5,6782 | | 0,000242 | | | 0,00362 |
| MRPL19 | Homo sapiens mitochondrial ribosomal protein L19 (MRPL19), nuclear gene encoding mitochondrial protein, mRNA. | -0,5128 | 8,6482 | | -6,9968 | | 0,000047 | | | 0,00155 |
| SIGMAR1 | Homo sapiens sigma non-opioid intracellular receptor 1 (SIGMAR1), transcript variant 2, mRNA. | -0,5128 | 7,9054 | | -4,3290 | | 0,001659 | | | 0,01135 |
| PMPCA | Homo sapiens peptidase (mitochondrial processing) alpha (PMPCA), nuclear gene encoding mitochondrial protein, mRNA. | -0,5129 | 8,5973 | | -4,2165 | | 0,001971 | | | 0,01275 |
| TAX1BP3 | Homo sapiens Tax1 (human T-cell leukemia virus type I) binding protein 3 (TAX1BP3), mRNA. | -0,5139 | 10,0965 | | -3,4325 | | 0,006851 | | | 0,03047 |
| ANKMY2 | Homo sapiens ankyrin repeat and MYND domain containing 2 (ANKMY2), mRNA. | -0,5145 | 8,3808 | | -5,1462 | | 0,000502 | | | 0,00542 |
| C8orf33 | Homo sapiens chromosome 8 open reading frame 33 (C8orf33), mRNA. | -0,5146 | 9,6470 | | -4,1658 | | 0,002131 | | | 0,01342 |
| EPN3 | Homo sapiens epsin 3 (EPN3), mRNA. | -0,5149 | 7,4320 | | -14,6010 | | 0,000000 | | | 0,00012 |
| COPS5 | Homo sapiens COP9 constitutive photomorphogenic homolog subunit 5 (Arabidopsis) (COPS5), mRNA. | -0,5150 | 10,7282 | | -4,1399 | | 0,002218 | | | 0,01378 |
| LDHA | Homo sapiens lactate dehydrogenase A (LDHA), transcript variant 2, mRNA. | -0,5152 | 13,2514 | | -4,8236 | | 0,000795 | | | 0,00712 |
| LEF1 | Homo sapiens lymphoid enhancer-binding factor 1 (LEF1), mRNA. | -0,5155 | 7,7882 | | -7,6605 | | 0,000022 | | | 0,00113 |
| RAN | Homo sapiens RAN, member RAS oncogene family (RAN), mRNA. | -0,5161 | 12,7466 | | -4,0230 | | 0,002661 | | | 0,01564 |
| UBP1 | Homo sapiens upstream binding protein 1 (LBP-1a) (UBP1), mRNA. | -0,5163 | 9,3734 | | -3,0817 | | 0,012226 | | | 0,04621 |
| C3orf31 | Homo sapiens chromosome 3 open reading frame 31 (C3orf31), mRNA. | -0,5165 | 7,9646 | | -3,5559 | | 0,005603 | | | 0,02622 |
| MRPL38 | Homo sapiens mitochondrial ribosomal protein L38 (MRPL38), nuclear gene encoding mitochondrial protein, mRNA. | -0,5169 | 9,3960 | | -4,6818 | | 0,000978 | | | 0,00814 |
| RAD54B | Homo sapiens RAD54 homolog B (S. cerevisiae) (RAD54B), mRNA. | -0,5172 | 7,5904 | | -8,0393 | | 0,000015 | | | 0,00095 |
| CWF19L2 | Homo sapiens CWF19-like 2, cell cycle control (S. pombe) (CWF19L2), mRNA. | -0,5180 | 8,2091 | | -6,7668 | | 0,000062 | | | 0,00178 |
| BCS1L | Homo sapiens BCS1-like (yeast) (BCS1L), nuclear gene encoding mitochondrial protein, transcript variant 2, mRNA. | -0,5182 | 8,2413 | | -3,6666 | | 0,004686 | | | 0,02302 |
| OSGEPL1 | Homo sapiens O-sialoglycoprotein endopeptidase-like 1 (OSGEPL1), mRNA. | -0,5182 | 7,7436 | | -6,4658 | | 0,000089 | | | 0,00211 |
| HDHD2 | Homo sapiens haloacid dehalogenase-like hydrolase domain containing 2 (HDHD2), mRNA. | -0,5182 | 7,9751 | | -3,6575 | | 0,004755 | | | 0,02325 |
| RAVER2 | Homo sapiens ribonucleoprotein, PTB-binding 2 (RAVER2), mRNA. | -0,5185 | 7,8043 | | -4,5357 | | 0,001215 | | | 0,00925 |
| ZWINT | Homo sapiens ZW10 interactor (ZWINT), transcript variant 3, mRNA. | -0,5188 | 7,5482 | | -5,7778 | | 0,000212 | | | 0,00337 |
| KIAA0146 | Homo sapiens KIAA0146 (KIAA0146), mRNA. | -0,5190 | 7,9731 | | -3,8448 | | 0,003524 | | | 0,01885 |
| APEH | Homo sapiens N-acylaminoacyl-peptide hydrolase (APEH), mRNA. | -0,5193 | 9,9277 | | -3,2568 | | 0,009144 | | | 0,03727 |
| TCTN3 | Homo sapiens tectonic family member 3 (TCTN3), mRNA. | -0,5193 | 8,0219 | | -5,1599 | | 0,000492 | | | 0,00537 |
| FARS2 | Homo sapiens phenylalanyl-tRNA synthetase 2, mitochondrial (FARS2), nuclear gene encoding mitochondrial protein, mRNA. | -0,5196 | 8,3687 | | -5,0292 | | 0,000592 | | | 0,00595 |
| RECQL4 | Homo sapiens RecQ protein-like 4 (RECQL4), mRNA. | -0,5202 | 7,9978 | | -3,3910 | | 0,007333 | | | 0,03201 |
| ZNF488 | Homo sapiens zinc finger protein 488 (ZNF488), mRNA. | -0,5202 | 7,4148 | | -13,4525 | | 0,000000 | | | 0,00014 |
| PLS1 | Homo sapiens plastin 1 (I isoform) (PLS1), mRNA. | -0,5202 | 7,7190 | | -8,5107 | | 0,000009 | | | 0,00078 |
| ZNHIT6 | Homo sapiens zinc finger, HIT type 6 (ZNHIT6), mRNA. | -0,5204 | 8,7919 | | -3,6198 | | 0,005052 | | | 0,02432 |
| CS | Homo sapiens citrate synthase (CS), nuclear gene encoding mitochondrial protein, mRNA. | -0,5207 | 10,3823 | | -7,0101 | | 0,000046 | | | 0,00154 |
| SNX27 | Homo sapiens sorting nexin family member 27 (SNX27), mRNA. | -0,5208 | 9,0142 | | -4,8791 | | 0,000734 | | | 0,00674 |
| MUDENG | Homo sapiens MU-2/AP1M2 domain containing, death-inducing (MUDENG), mRNA. | -0,5214 | 8,0654 | | -8,9111 | | 0,000006 | | | 0,00064 |
| ACSL3 | Homo sapiens acyl-CoA synthetase long-chain family member 3 (ACSL3), transcript variant 2, mRNA. | -0,5217 | 9,7037 | | -3,0311 | | 0,013304 | | | 0,04928 |
| TRAPPC2L | Homo sapiens trafficking protein particle complex 2-like (TRAPPC2L), mRNA. | -0,5221 | 9,4897 | | -5,1938 | | 0,000469 | | | 0,00523 |
| CLEC2D | Homo sapiens C-type lectin domain family 2, member D (CLEC2D), transcript variant 1, mRNA. | -0,5230 | 12,3746 | | -3,3733 | | 0,007548 | | | 0,03265 |
| LOC100130932 | PREDICTED: Homo sapiens similar to Sm protein G (LOC100130932), mRNA. | -0,5234 | 8,7968 | | -4,2815 | | 0,001784 | | | 0,01192 |
| NDUFS8 | Homo sapiens NADH dehydrogenase (ubiquinone) Fe-S protein 8, 23kDa (NADH-coenzyme Q reductase) (NDUFS8), mRNA. | -0,5235 | 10,7848 | | -3,7849 | | 0,003877 | | | 0,02013 |
| FSTL1 | Homo sapiens follistatin-like 1 (FSTL1), mRNA. | -0,5238 | 7,8790 | | -3,3137 | | 0,008325 | | | 0,03496 |
| DSN1 | Homo sapiens DSN1, MIND kinetochore complex component, homolog (S. cerevisiae) (DSN1), mRNA. | -0,5244 | 8,6424 | | -3,9857 | | 0,002821 | | | 0,01625 |
| TH1L | Homo sapiens TH1-like (Drosophila) (TH1L), transcript variant 2, mRNA. | -0,5246 | 9,8584 | | -3,4957 | | 0,006179 | | | 0,02823 |
| SNUPN | Homo sapiens snurportin 1 (SNUPN), transcript variant 3, mRNA. | -0,5248 | 8,3200 | | -5,6700 | | 0,000245 | | | 0,00364 |
| NDUFS5 | Homo sapiens NADH dehydrogenase (ubiquinone) Fe-S protein 5, 15kDa (NADH-coenzyme Q reductase) (NDUFS5), mRNA. | -0,5250 | 12,0466 | | -5,4113 | | 0,000347 | | | 0,00437 |
| TMEM45A | Homo sapiens transmembrane protein 45A (TMEM45A), mRNA. | -0,5254 | 7,9280 | | -3,2929 | | 0,008615 | | | 0,03581 |
| H2AFY | Homo sapiens H2A histone family, member Y (H2AFY), transcript variant 1, mRNA. | -0,5261 | 8,5526 | | -3,5584 | | 0,005581 | | | 0,02614 |
| SAMM50 | Homo sapiens sorting and assembly machinery component 50 homolog (S. cerevisiae) (SAMM50), mRNA. | -0,5262 | 8,9201 | | -4,5805 | | 0,001137 | | | 0,00891 |
| DNAJC15 | Homo sapiens DnaJ (Hsp40) homolog, subfamily C, member 15 (DNAJC15), mRNA. | -0,5267 | 7,8016 | | -5,8568 | | 0,000192 | | | 0,00319 |
| CKAP2 | Homo sapiens cytoskeleton associated protein 2 (CKAP2), mRNA. | -0,5273 | 7,8708 | | -4,0338 | | 0,002616 | | | 0,01549 |
| SSBP4 | Homo sapiens single stranded DNA binding protein 4 (SSBP4), transcript variant 1, mRNA. | -0,5273 | 8,0078 | | -6,8042 | | 0,000059 | | | 0,00174 |
| SNX17 | Homo sapiens sorting nexin 17 (SNX17), mRNA. | -0,5275 | 9,8419 | | -3,6285 | | 0,004982 | | | 0,02409 |
| AKR1B1 | Homo sapiens aldo-keto reductase family 1, member B1 (aldose reductase) (AKR1B1), mRNA. | -0,5276 | 9,9353 | | -3,4249 | | 0,006936 | | | 0,03074 |
| LOC728006 | PREDICTED: Homo sapiens hypothetical protein LOC728006 (LOC728006), mRNA. | -0,5277 | 8,7968 | | -3,4427 | | 0,006737 | | | 0,03009 |
| RBBP9 | Homo sapiens retinoblastoma binding protein 9 (RBBP9), mRNA. | -0,5283 | 7,5734 | | -7,8107 | | 0,000019 | | | 0,00104 |
| INTS9 | Homo sapiens integrator complex subunit 9 (INTS9), mRNA. | -0,5283 | 8,2989 | | -5,0618 | | 0,000565 | | | 0,00580 |
| TMEM54 | Homo sapiens transmembrane protein 54 (TMEM54), mRNA. | -0,5285 | 9,9260 | | -5,0897 | | 0,000543 | | | 0,00568 |
| LOC100131609 | PREDICTED: Homo sapiens misc_RNA (LOC100131609), miscRNA. | -0,5287 | 11,5403 | | -3,4785 | | 0,006355 | | | 0,02879 |
| ALDH3A2 | Homo sapiens aldehyde dehydrogenase 3 family, member A2 (ALDH3A2), transcript variant 1, mRNA. | -0,5288 | 7,8089 | | -8,1620 | | 0,000013 | | | 0,00090 |
| ARMC7 | Homo sapiens armadillo repeat containing 7 (ARMC7), mRNA. | -0,5292 | 7,8241 | | -6,0023 | | 0,000159 | | | 0,00287 |
| CNOT1 | Homo sapiens CCR4-NOT transcription complex, subunit 1 (CNOT1), transcript variant 1, mRNA. | -0,5295 | 9,7009 | | -3,0732 | | 0,012402 | | | 0,04674 |
| LOC347376 | PREDICTED: Homo sapiens similar to H3 histone, family 3B (LOC347376), mRNA. | -0,5297 | 8,4294 | | -6,0530 | | 0,000149 | | | 0,00277 |
| DHX35 | Homo sapiens DEAH (Asp-Glu-Ala-His) box polypeptide 35 (DHX35), mRNA. | -0,5304 | 7,9436 | | -6,1353 | | 0,000134 | | | 0,00262 |
| PPME1 | Homo sapiens protein phosphatase methylesterase 1 (PPME1), mRNA. | -0,5310 | 8,5905 | | -6,4054 | | 0,000095 | | | 0,00217 |
| LSG1 | Homo sapiens large subunit GTPase 1 homolog (S. cerevisiae) (LSG1), mRNA. | -0,5311 | 8,7653 | | -5,4478 | | 0,000330 | | | 0,00430 |
| ATP5L | Homo sapiens ATP synthase, H+ transporting, mitochondrial F0 complex, subunit G (ATP5L), nuclear gene encoding mitochondrial protein, mRNA. | -0,5314 | 12,1336 | | -6,7683 | | 0,000062 | | | 0,00178 |
| MEIS2 | Homo sapiens Meis homeobox 2 (MEIS2), transcript variant g, mRNA. | -0,5315 | 7,6359 | | -7,1208 | | 0,000041 | | | 0,00145 |
| CDK5 | Homo sapiens cyclin-dependent kinase 5 (CDK5), mRNA. | -0,5321 | 8,0466 | | -5,7788 | | 0,000212 | | | 0,00337 |
| DDX19A | Homo sapiens DEAD (Asp-Glu-Ala-As) box polypeptide 19A (DDX19A), mRNA. | -0,5322 | 8,1742 | | -6,3210 | | 0,000106 | | | 0,00229 |
| AFG3L2 | Homo sapiens AFG3 ATPase family gene 3-like 2 (yeast) (AFG3L2), nuclear gene encoding mitochondrial protein, mRNA. | -0,5325 | 10,8729 | | -4,3425 | | 0,001625 | | | 0,01120 |
| TBCD | Homo sapiens tubulin folding cofactor D (TBCD), mRNA. | -0,5329 | 8,2183 | | -4,0713 | | 0,002468 | | | 0,01488 |
| SLC19A1 | Homo sapiens solute carrier family 19 (folate transporter), member 1 (SLC19A1), mRNA. | -0,5333 | 7,8072 | | -4,3764 | | 0,001544 | | | 0,01084 |
| PREP | Homo sapiens prolyl endopeptidase (PREP), mRNA. | -0,5340 | 8,6339 | | -3,2829 | | 0,008759 | | | 0,03618 |
| GLO1 | Homo sapiens glyoxalase I (GLO1), mRNA. | -0,5341 | 10,7345 | | -5,5751 | | 0,000278 | | | 0,00390 |
| RASL11B | Homo sapiens RAS-like, family 11, member B (RASL11B), mRNA. | -0,5343 | 7,8710 | | -4,2333 | | 0,001921 | | | 0,01250 |
| DDX28 | Homo sapiens DEAD (Asp-Glu-Ala-Asp) box polypeptide 28 (DDX28), nuclear gene encoding mitochondrial protein, mRNA. | -0,5358 | 8,2493 | | -5,3683 | | 0,000368 | | | 0,00451 |
| EXOSC7 | Homo sapiens exosome component 7 (EXOSC7), mRNA. | -0,5360 | 9,2715 | | -4,0627 | | 0,002501 | | | 0,01502 |
| LOC647307 | PREDICTED: Homo sapiens misc_RNA (LOC647307), miscRNA. | -0,5363 | 9,0337 | | -3,3054 | | 0,008440 | | | 0,03526 |
| NEIL3 | Homo sapiens nei endonuclease VIII-like 3 (E. coli) (NEIL3), mRNA. | -0,5365 | 7,5732 | | -5,5799 | | 0,000276 | | | 0,00389 |
| CDK5RAP1 | Homo sapiens CDK5 regulatory subunit associated protein 1 (CDK5RAP1), transcript variant 1, mRNA. | -0,5369 | 8,9915 | | -5,7325 | | 0,000226 | | | 0,00349 |
| EIF4A3 | Homo sapiens eukaryotic translation initiation factor 4A, isoform 3 (EIF4A3), mRNA. | -0,5372 | 11,5983 | | -5,9581 | | 0,000168 | | | 0,00297 |
| GART | Homo sapiens phosphoribosylglycinamide formyltransferase, phosphoribosylglycinamide synthetase, phosphoribosylaminoimidazole synthetase (GART), transcript variant 2, mRNA. | -0,5372 | 8,6261 | | -4,5658 | | 0,001162 | | | 0,00903 |
| LOC728877 | PREDICTED: Homo sapiens misc_RNA (LOC728877), miscRNA. | -0,5374 | 9,2109 | | -3,8624 | | 0,003427 | | | 0,01848 |
| GPN3 | Homo sapiens GPN-loop GTPase 3 (GPN3), mRNA. | -0,5377 | 8,8130 | | -4,2270 | | 0,001939 | | | 0,01259 |
| POLR3B | Homo sapiens polymerase (RNA) III (DNA directed) polypeptide B (POLR3B), mRNA. | -0,5378 | 8,0831 | | -4,6061 | | 0,001094 | | | 0,00871 |
| GTF2IP1 | Homo sapiens general transcription factor II, i, pseudogene 1 (GTF2IP1) on chromosome 7. | -0,5380 | 7,9798 | | -4,4056 | | 0,001477 | | | 0,01054 |
| SAP30 | Homo sapiens Sin3A-associated protein, 30kDa (SAP30), mRNA. | -0,5382 | 7,7356 | | -7,4616 | | 0,000028 | | | 0,00122 |
| CKAP4 | Homo sapiens cytoskeleton-associated protein 4 (CKAP4), mRNA. | -0,5382 | 11,6498 | | -3,1172 | | 0,011525 | | | 0,04414 |
| AOF2 | Homo sapiens amine oxidase (flavin containing) domain 2 (AOF2), transcript variant 2, mRNA. | -0,5383 | 9,3671 | | -5,4154 | | 0,000345 | | | 0,00436 |
| C12orf4 | Homo sapiens chromosome 12 open reading frame 4 (C12orf4), mRNA. | -0,5390 | 7,7966 | | -8,5937 | | 0,000008 | | | 0,00074 |
| UBE1 | Homo sapiens ubiquitin-activating enzyme E1 (UBE1), transcript variant 1, mRNA. | -0,5390 | 9,8940 | | -3,0326 | | 0,013271 | | | 0,04919 |
| COX15 | Homo sapiens COX15 homolog, cytochrome c oxidase assembly protein (yeast) (COX15), nuclear gene encoding mitochondrial protein, transcript variant 1, mRNA. | -0,5393 | 7,8446 | | -7,4331 | | 0,000029 | | | 0,00124 |
| TMEM30A | Homo sapiens transmembrane protein 30A (TMEM30A), mRNA. | -0,5395 | 7,9854 | | -8,2181 | | 0,000012 | | | 0,00088 |
| DHX37 | Homo sapiens DEAH (Asp-Glu-Ala-His) box polypeptide 37 (DHX37), mRNA. | -0,5402 | 8,7102 | | -3,6908 | | 0,004507 | | | 0,02244 |
| PIN1 | Homo sapiens peptidylprolyl cis/trans isomerase, NIMA-interacting 1 (PIN1), mRNA. | -0,5404 | 10,1386 | | -3,3653 | | 0,007648 | | | 0,03297 |
| TEX2 | Homo sapiens testis expressed 2 (TEX2), mRNA. | -0,5408 | 8,5582 | | -3,1846 | | 0,010305 | | | 0,04060 |
| COBLL1 | Homo sapiens COBL-like 1 (COBLL1), mRNA. | -0,5409 | 7,9754 | | -4,6069 | | 0,001093 | | | 0,00870 |
| DDX10 | Homo sapiens DEAD (Asp-Glu-Ala-Asp) box polypeptide 10 (DDX10), mRNA. | -0,5414 | 9,1325 | | -3,8192 | | 0,003670 | | | 0,01938 |
| C10orf61 | Homo sapiens chromosome 10 open reading frame 61 (C10orf61), transcript variant 1, mRNA. | -0,5414 | 8,3766 | | -3,5453 | | 0,005700 | | | 0,02659 |
| CCNJL | Homo sapiens cyclin J-like (CCNJL), mRNA. | -0,5416 | 7,9351 | | -7,4249 | | 0,000029 | | | 0,00125 |
| PTP4A2 | Homo sapiens protein tyrosine phosphatase type IVA, member 2 (PTP4A2), transcript variant 2, mRNA. XM_944930 XM_944934 | -0,5420 | 10,8524 | | -3,3326 | | 0,008069 | | | 0,03421 |
| LYRM7 | Homo sapiens Lyrm7 homolog (mouse) (LYRM7), mRNA. | -0,5421 | 7,8562 | | -5,0931 | | 0,000541 | | | 0,00566 |
| RPL34 | Homo sapiens ribosomal protein L34 (RPL34), transcript variant 2, mRNA. | -0,5422 | 9,8590 | | -3,6108 | | 0,005126 | | | 0,02455 |
| MRRF | Homo sapiens mitochondrial ribosome recycling factor (MRRF), nuclear gene encoding mitochondrial protein, transcript variant 2, mRNA. | -0,5423 | 8,6161 | | -5,3947 | | 0,000355 | | | 0,00441 |
| TUSC3 | Homo sapiens tumor suppressor candidate 3 (TUSC3), transcript variant 1, mRNA. | -0,5426 | 9,3569 | | -3,6915 | | 0,004502 | | | 0,02242 |
| TMEM69 | Homo sapiens transmembrane protein 69 (TMEM69), mRNA. | -0,5428 | 9,2297 | | -4,2557 | | 0,001856 | | | 0,01221 |
| VBP1 | Homo sapiens von Hippel-Lindau binding protein 1 (VBP1), mRNA. | -0,5429 | 10,4613 | | -3,6702 | | 0,004658 | | | 0,02293 |
| PARL | Homo sapiens presenilin associated, rhomboid-like (PARL), nuclear gene encoding mitochondrial protein, transcript variant 2, mRNA. | -0,5432 | 10,8578 | | -4,0710 | | 0,002469 | | | 0,01488 |
| PSMB6 | Homo sapiens proteasome (prosome, macropain) subunit, beta type, 6 (PSMB6), mRNA. | -0,5436 | 12,0374 | | -7,7370 | | 0,000021 | | | 0,00108 |
| TMEM39B | Homo sapiens transmembrane protein 39B (TMEM39B), mRNA. | -0,5439 | 8,5552 | | -5,4338 | | 0,000337 | | | 0,00433 |
| RNASEN | Homo sapiens ribonuclease III, nuclear (RNASEN), mRNA. | -0,5439 | 9,0576 | | -5,6218 | | 0,000261 | | | 0,00379 |
| TUBA3D | Homo sapiens tubulin, alpha 3d (TUBA3D), mRNA. | -0,5441 | 8,6310 | | -4,5546 | | 0,001181 | | | 0,00911 |
| LOC652489 | PREDICTED: Homo sapiens similar to SMT3 suppressor of mif two 3 homolog 2 (LOC652489), mRNA. | -0,5448 | 9,9575 | | -3,2760 | | 0,008859 | | | 0,03648 |
| CIAPIN1 | Homo sapiens cytokine induced apoptosis inhibitor 1 (CIAPIN1), mRNA. | -0,5454 | 8,6599 | | -4,5560 | | 0,001179 | | | 0,00910 |
| PHB2 | Homo sapiens prohibitin 2 (PHB2), transcript variant 2, mRNA. | -0,5459 | 10,2916 | | -4,7563 | | 0,000877 | | | 0,00761 |
| GINS4 | Homo sapiens GINS complex subunit 4 (Sld5 homolog) (GINS4), mRNA. | -0,5459 | 7,6850 | | -6,5135 | | 0,000084 | | | 0,00204 |
| TOPBP1 | Homo sapiens topoisomerase (DNA) II binding protein 1 (TOPBP1), mRNA. | -0,5464 | 8,6176 | | -4,8852 | | 0,000727 | | | 0,00671 |
| CSTF3 | Homo sapiens cleavage stimulation factor, 3' pre-RNA, subunit 3, 77kDa (CSTF3), transcript variant 2, mRNA. | -0,5468 | 8,1527 | | -6,1891 | | 0,000125 | | | 0,00250 |
| LOC648210 | PREDICTED: Homo sapiens similar to Heterogeneous nuclear ribonucleoprotein A1 (Helix-destabilizing protein) (Single-strand RNA-binding protein) (hnRNP core protein A1) (HDP) (LOC648210), mRNA. | -0,5468 | 11,9770 | | -3,5074 | | 0,006062 | | | 0,02786 |
| DNM3 | Homo sapiens dynamin 3 (DNM3), mRNA. | -0,5474 | 7,8019 | | -4,4084 | | 0,001471 | | | 0,01052 |
| PELP1 | Homo sapiens proline, glutamic acid and leucine rich protein 1 (PELP1), mRNA. | -0,5475 | 8,8115 | | -3,9039 | | 0,003209 | | | 0,01766 |
| IDH3B | Homo sapiens isocitrate dehydrogenase 3 (NAD+) beta (IDH3B), nuclear gene encoding mitochondrial protein, transcript variant 3, mRNA. | -0,5477 | 10,6001 | | -5,3639 | | 0,000371 | | | 0,00453 |
| GTPBP3 | Homo sapiens GTP binding protein 3 (mitochondrial) (GTPBP3), nuclear gene encoding mitochondrial protein, transcript variant V, mRNA. | -0,5479 | 9,1418 | | -3,7186 | | 0,004310 | | | 0,02173 |
| IQCK | Homo sapiens IQ motif containing K (IQCK), mRNA. | -0,5485 | 7,9895 | | -3,9458 | | 0,003004 | | | 0,01690 |
| POP7 | Homo sapiens processing of precursor 7, ribonuclease P/MRP subunit (S. cerevisiae) (POP7), mRNA. | -0,5485 | 9,2276 | | -4,6214 | | 0,001070 | | | 0,00856 |
| RWDD4A | Homo sapiens RWD domain containing 4A (RWDD4A), mRNA. | -0,5488 | 8,2178 | | -4,3863 | | 0,001521 | | | 0,01075 |
| MRI1 | Homo sapiens methylthioribose-1-phosphate isomerase homolog (S. cerevisiae) (MRI1), transcript variant 2, mRNA. | -0,5489 | 8,6991 | | -4,2367 | | 0,001911 | | | 0,01246 |
| TMEM9 | Homo sapiens transmembrane protein 9 (TMEM9), mRNA. | -0,5492 | 8,0804 | | -5,3792 | | 0,000363 | | | 0,00447 |
| POLA1 | Homo sapiens polymerase (DNA directed), alpha 1, catalytic subunit (POLA1), mRNA. | -0,5492 | 7,7186 | | -4,6258 | | 0,001063 | | | 0,00853 |
| C8orf33 | Homo sapiens chromosome 8 open reading frame 33 (C8orf33), mRNA. | -0,5492 | 8,9321 | | -3,9417 | | 0,003023 | | | 0,01698 |
| MRPS15 | Homo sapiens mitochondrial ribosomal protein S15 (MRPS15), nuclear gene encoding mitochondrial protein, mRNA. | -0,5492 | 10,4485 | | -3,8065 | | 0,003745 | | | 0,01965 |
| ACO1 | Homo sapiens aconitase 1, soluble (ACO1), mRNA. | -0,5494 | 8,8638 | | -4,1459 | | 0,002198 | | | 0,01372 |
| NCAPD3 | Homo sapiens non-SMC condensin II complex, subunit D3 (NCAPD3), mRNA. | -0,5498 | 7,6863 | | -4,7408 | | 0,000897 | | | 0,00771 |
| VPS24 | Homo sapiens vacuolar protein sorting 24 homolog (S. cerevisiae) (VPS24), transcript variant 2, mRNA. | -0,5498 | 8,9553 | | -3,1719 | | 0,010525 | | | 0,04125 |
| ZBED5 | Homo sapiens zinc finger, BED-type containing 5 (ZBED5), mRNA. | -0,5499 | 8,9769 | | -5,4999 | | 0,000308 | | | 0,00413 |
| C1QTNF6 | Homo sapiens C1q and tumor necrosis factor related protein 6 (C1QTNF6), transcript variant 1, mRNA. | -0,5500 | 7,9755 | | -4,1850 | | 0,002069 | | | 0,01316 |
| CXCL16 | Homo sapiens chemokine (C-X-C motif) ligand 16 (CXCL16), mRNA. | -0,5501 | 7,7780 | | -4,4855 | | 0,001310 | | | 0,00974 |
| VARS | Homo sapiens valyl-tRNA synthetase (VARS), nuclear gene encoding mitochondrial protein, mRNA. | -0,5501 | 8,8114 | | -3,8238 | | 0,003643 | | | 0,01927 |
| CAV2 | Homo sapiens caveolin 2 (CAV2), transcript variant 1, mRNA. | -0,5503 | 8,4036 | | -3,2129 | | 0,009832 | | | 0,03927 |
| LOC643300 | PREDICTED: Homo sapiens similar to 60 kDa heat shock protein, mitochondrial precursor (Hsp60) (60 kDa chaperonin) (CPN60) (Heat shock protein 60) (HSP-60) (Mitochondrial matrix protein P1) (P60 lymphocyte protein) (HuCHA60) (LOC643300), mRNA. | -0,5504 | 8,1657 | | -3,1803 | | 0,010378 | | | 0,04078 |
| HPS3 | Homo sapiens Hermansky-Pudlak syndrome 3 (HPS3), mRNA. | -0,5505 | 7,8876 | | -7,6865 | | 0,000022 | | | 0,00112 |
| TSGA14 | Homo sapiens testis specific, 14 (TSGA14), mRNA. | -0,5509 | 7,8359 | | -6,8667 | | 0,000055 | | | 0,00169 |
| ATP5L | Homo sapiens ATP synthase, H+ transporting, mitochondrial F0 complex, subunit G (ATP5L), nuclear gene encoding mitochondrial protein, mRNA. | -0,5509 | 8,0989 | | -5,8611 | | 0,000190 | | | 0,00318 |
| PSMF1 | Homo sapiens proteasome (prosome, macropain) inhibitor subunit 1 (PI31) (PSMF1), transcript variant 3, mRNA. | -0,5511 | 9,2411 | | -3,8244 | | 0,003640 | | | 0,01926 |
| METTL4 | Homo sapiens methyltransferase like 4 (METTL4), mRNA. | -0,5511 | 8,0145 | | -3,7695 | | 0,003973 | | | 0,02048 |
| CDC25C | Homo sapiens cell division cycle 25 homolog C (S. pombe) (CDC25C), transcript variant 2, mRNA. | -0,5512 | 7,7372 | | -3,7656 | | 0,003998 | | | 0,02058 |
| PIGC | Homo sapiens phosphatidylinositol glycan anchor biosynthesis, class C (PIGC), transcript variant 1, mRNA. | -0,5517 | 8,8895 | | -11,8426 | | 0,000001 | | | 0,00028 |
| MRPL18 | Homo sapiens mitochondrial ribosomal protein L18 (MRPL18), nuclear gene encoding mitochondrial protein, mRNA. | -0,5518 | 10,4805 | | -4,9387 | | 0,000673 | | | 0,00638 |
| CD99L2 | Homo sapiens CD99 molecule-like 2 (CD99L2), transcript variant 3, mRNA. | -0,5519 | 8,4807 | | -4,2309 | | 0,001928 | | | 0,01254 |
| USP13 | Homo sapiens ubiquitin specific peptidase 13 (isopeptidase T-3) (USP13), mRNA. | -0,5521 | 7,6701 | | -6,7102 | | 0,000066 | | | 0,00183 |
| KHDRBS1 | Homo sapiens KH domain containing, RNA binding, signal transduction associated 1 (KHDRBS1), mRNA. | -0,5524 | 11,3947 | | -5,6876 | | 0,000239 | | | 0,00360 |
| TXLNA | Homo sapiens taxilin alpha (TXLNA), mRNA. | -0,5531 | 9,2917 | | -6,7212 | | 0,000065 | | | 0,00182 |
| DFFA | Homo sapiens DNA fragmentation factor, 45kDa, alpha polypeptide (DFFA), transcript variant 2, mRNA. | -0,5532 | 8,7058 | | -3,3457 | | 0,007898 | | | 0,03372 |
| FIGNL1 | Homo sapiens fidgetin-like 1 (FIGNL1), transcript variant 1, mRNA. | -0,5533 | 7,6145 | | -7,3256 | | 0,000032 | | | 0,00129 |
| PSMC3IP | Homo sapiens PSMC3 interacting protein (PSMC3IP), transcript variant 2, mRNA. | -0,5535 | 7,9274 | | -4,0682 | | 0,002480 | | | 0,01492 |
| MLEC | Homo sapiens malectin (MLEC), mRNA. | -0,5539 | 10,1031 | | -3,7263 | | 0,004257 | | | 0,02154 |
| SMYD4 | Homo sapiens SET and MYND domain containing 4 (SMYD4), mRNA. | -0,5541 | 8,2305 | | -5,4289 | | 0,000339 | | | 0,00434 |
| C19orf62 | Homo sapiens chromosome 19 open reading frame 62 (C19orf62), transcript variant 2, mRNA. | -0,5545 | 8,5788 | | -5,5924 | | 0,000272 | | | 0,00386 |
| MRPL42 | Homo sapiens mitochondrial ribosomal protein L42 (MRPL42), nuclear gene encoding mitochondrial protein, transcript variant 2, mRNA. | -0,5552 | 8,8828 | | -4,3126 | | 0,001701 | | | 0,01155 |
| LOC644101 | PREDICTED: Homo sapiens misc_RNA (LOC644101), miscRNA. | -0,5555 | 8,5882 | | -7,4272 | | 0,000029 | | | 0,00125 |
| C14orf142 | Homo sapiens chromosome 14 open reading frame 142 (C14orf142), mRNA. | -0,5557 | 8,0442 | | -6,0805 | | 0,000144 | | | 0,00272 |
| CS | Homo sapiens citrate synthase (CS), nuclear gene encoding mitochondrial protein, mRNA. | -0,5566 | 8,6176 | | -5,1744 | | 0,000482 | | | 0,00532 |
| CCT6P1 | Homo sapiens chaperonin containing TCP1, subunit 6 (zeta) pseudogene 1 (CCT6P1), non-coding RNA. | -0,5567 | 11,5016 | | -3,7537 | | 0,004074 | | | 0,02087 |
| ARMC1 | Homo sapiens armadillo repeat containing 1 (ARMC1), mRNA. | -0,5573 | 8,7111 | | -5,2598 | | 0,000428 | | | 0,00495 |
| RBM10 | Homo sapiens RNA binding motif protein 10 (RBM10), transcript variant 2, mRNA. | -0,5575 | 10,0671 | | -5,3794 | | 0,000363 | | | 0,00447 |
| SLFN11 | Homo sapiens schlafen family member 11 (SLFN11), mRNA. | -0,5578 | 9,2829 | | -3,3631 | | 0,007675 | | | 0,03306 |
| LSM3 | Homo sapiens LSM3 homolog, U6 small nuclear RNA associated (S. cerevisiae) (LSM3), mRNA. | -0,5586 | 10,5290 | | -4,1069 | | 0,002335 | | | 0,01428 |
| TRMT12 | Homo sapiens tRNA methyltransferase 12 homolog (S. cerevisiae) (TRMT12), mRNA. | -0,5587 | 9,1194 | | -5,0636 | | 0,000564 | | | 0,00580 |
| PSMA5 | Homo sapiens proteasome (prosome, macropain) subunit, alpha type, 5 (PSMA5), mRNA. | -0,5590 | 11,9269 | | -5,4635 | | 0,000323 | | | 0,00424 |
| MTHFD1 | Homo sapiens methylenetetrahydrofolate dehydrogenase (NADP+ dependent) 1, methenyltetrahydrofolate cyclohydrolase, formyltetrahydrofolate synthetase (MTHFD1), mRNA. | -0,5596 | 7,8294 | | -4,4497 | | 0,001382 | | | 0,01009 |
| LOC100128266 | PREDICTED: Homo sapiens misc_RNA (LOC100128266), miscRNA. | -0,5596 | 12,2798 | | -4,6049 | | 0,001096 | | | 0,00871 |
| WDR70 | Homo sapiens WD repeat domain 70 (WDR70), mRNA. | -0,5597 | 8,5369 | | -3,6160 | | 0,005084 | | | 0,02441 |
| C19orf70 | Homo sapiens chromosome 19 open reading frame 70 (C19orf70), mRNA. | -0,5599 | 10,2506 | | -3,1494 | | 0,010925 | | | 0,04240 |
| TPRKB | Homo sapiens TP53RK binding protein (TPRKB), mRNA. | -0,5599 | 8,8602 | | -5,2061 | | 0,000461 | | | 0,00519 |
| CENPB | Homo sapiens centromere protein B, 80kDa (CENPB), mRNA. | -0,5607 | 10,3509 | | -8,3116 | | 0,000011 | | | 0,00085 |
| PSMC6 | Homo sapiens proteasome (prosome, macropain) 26S subunit, ATPase, 6 (PSMC6), mRNA. | -0,5609 | 10,4505 | | -4,2028 | | 0,002013 | | | 0,01292 |
| LOC730256 | PREDICTED: Homo sapiens hypothetical protein LOC730256 (LOC730256), mRNA. | -0,5610 | 8,0916 | | -4,9923 | | 0,000624 | | | 0,00613 |
| HELLS | Homo sapiens helicase, lymphoid-specific (HELLS), mRNA. | -0,5613 | 7,6002 | | -7,4206 | | 0,000029 | | | 0,00125 |
| OPA1 | Homo sapiens optic atrophy 1 (autosomal dominant) (OPA1), nuclear gene encoding mitochondrial protein, transcript variant 1, mRNA. | -0,5615 | 8,5197 | | -4,0246 | | 0,002654 | | | 0,01562 |
| MRI1 | Homo sapiens methylthioribose-1-phosphate isomerase homolog (S. cerevisiae) (MRI1), transcript variant 1, mRNA. | -0,5623 | 9,1532 | | -3,0564 | | 0,012753 | | | 0,04781 |
| PRDX1 | Homo sapiens peroxiredoxin 1 (PRDX1), transcript variant 2, mRNA. | -0,5623 | 13,0628 | | -6,9495 | | 0,000050 | | | 0,00161 |
| PTCD2 | Homo sapiens pentatricopeptide repeat domain 2 (PTCD2), mRNA. | -0,5624 | 7,7760 | | -5,6831 | | 0,000241 | | | 0,00361 |
| DNAJA1 | Homo sapiens DnaJ (Hsp40) homolog, subfamily A, member 1 (DNAJA1), mRNA. | -0,5625 | 11,6323 | | -3,5785 | | 0,005402 | | | 0,02551 |
| ACACA | Homo sapiens acetyl-Coenzyme A carboxylase alpha (ACACA), transcript variant 2, mRNA. | -0,5629 | 8,5511 | | -3,9251 | | 0,003103 | | | 0,01724 |
| DNAJC8 | Homo sapiens DnaJ (Hsp40) homolog, subfamily C, member 8 (DNAJC8), mRNA. | -0,5633 | 10,9447 | | -4,2033 | | 0,002012 | | | 0,01292 |
| CLNS1A | Homo sapiens chloride channel, nucleotide-sensitive, 1A (CLNS1A), mRNA. | -0,5634 | 10,1146 | | -4,3171 | | 0,001690 | | | 0,01150 |
| BTF3L4 | Homo sapiens basic transcription factor 3-like 4 (BTF3L4), mRNA. | -0,5637 | 8,9461 | | -5,3708 | | 0,000367 | | | 0,00450 |
| BCCIP | Homo sapiens BRCA2 and CDKN1A interacting protein (BCCIP), transcript variant A, mRNA. | -0,5643 | 8,2782 | | -6,3658 | | 0,000100 | | | 0,00224 |
| METTL1 | Homo sapiens methyltransferase like 1 (METTL1), transcript variant 1, mRNA. | -0,5651 | 8,2164 | | -4,0394 | | 0,002594 | | | 0,01540 |
| CCT6A | Homo sapiens chaperonin containing TCP1, subunit 6A (zeta 1) (CCT6A), transcript variant 2, mRNA. | -0,5652 | 10,7507 | | -3,9638 | | 0,002920 | | | 0,01663 |
| NDUFA12 | Homo sapiens NADH dehydrogenase (ubiquinone) 1 alpha subcomplex, 12 (NDUFA12), mRNA. | -0,5655 | 11,3263 | | -4,2545 | | 0,001859 | | | 0,01223 |
| MLF1IP | Homo sapiens MLF1 interacting protein (MLF1IP), mRNA. | -0,5658 | 7,5579 | | -6,5320 | | 0,000082 | | | 0,00201 |
| TUFM | Homo sapiens Tu translation elongation factor, mitochondrial (TUFM), nuclear gene encoding mitochondrial protein, mRNA. | -0,5661 | 9,9430 | | -3,3639 | | 0,007665 | | | 0,03303 |
| HAUS8 | Homo sapiens HAUS augmin-like complex, subunit 8 (HAUS8), transcript variant 2, mRNA. | -0,5661 | 7,7455 | | -5,9192 | | 0,000177 | | | 0,00305 |
| DPYSL2 | Homo sapiens dihydropyrimidinase-like 2 (DPYSL2), mRNA. | -0,5668 | 8,9895 | | -3,3456 | | 0,007900 | | | 0,03372 |
| LOC92755 | PREDICTED: Homo sapiens misc_RNA (LOC92755), miscRNA. | -0,5671 | 10,8429 | | -4,6161 | | 0,001078 | | | 0,00862 |
| ANAPC5 | Homo sapiens anaphase promoting complex subunit 5 (ANAPC5), mRNA. | -0,5676 | 10,1850 | | -7,5349 | | 0,000026 | | | 0,00119 |
| AASDHPPT | Homo sapiens aminoadipate-semialdehyde dehydrogenase-phosphopantetheinyl transferase (AASDHPPT), mRNA. | -0,5677 | 8,0875 | | -5,8128 | | 0,000203 | | | 0,00330 |
| FAM120B | Homo sapiens family with sequence similarity 120B (FAM120B), mRNA. | -0,5678 | 9,4439 | | -5,5498 | | 0,000288 | | | 0,00398 |
| ILVBL | Homo sapiens ilvB (bacterial acetolactate synthase)-like (ILVBL), mRNA. | -0,5679 | 8,8194 | | -3,3415 | | 0,007953 | | | 0,03384 |
| LOC652595 | PREDICTED: Homo sapiens similar to U2 small nuclear ribonucleoprotein A (U2 snRNP-A) (LOC652595), mRNA. | -0,5680 | 9,6585 | | -4,9585 | | 0,000655 | | | 0,00630 |
| LOC646993 | PREDICTED: Homo sapiens similar to high-mobility group box 3 (LOC646993), mRNA. | -0,5681 | 8,2443 | | -3,8102 | | 0,003723 | | | 0,01957 |
| FASTKD3 | Homo sapiens FAST kinase domains 3 (FASTKD3), mRNA. | -0,5682 | 8,0924 | | -6,1695 | | 0,000128 | | | 0,00253 |
| CCT7 | Homo sapiens chaperonin containing TCP1, subunit 7 (eta) (CCT7), transcript variant 1, mRNA. | -0,5683 | 9,9812 | | -3,8915 | | 0,003273 | | | 0,01791 |
| CAP2 | Homo sapiens CAP, adenylate cyclase-associated protein, 2 (yeast) (CAP2), mRNA. | -0,5688 | 8,4547 | | -3,6450 | | 0,004851 | | | 0,02361 |
| EARS2 | Homo sapiens glutamyl-tRNA synthetase 2, mitochondrial (putative) (EARS2), transcript variant 2, non-coding RNA. | -0,5690 | 7,9764 | | -4,0455 | | 0,002569 | | | 0,01530 |
| CBX5 | Homo sapiens chromobox homolog 5 (HP1 alpha homolog, Drosophila) (CBX5), mRNA. | -0,5694 | 8,2064 | | -3,2516 | | 0,009224 | | | 0,03749 |
| ZNHIT6 | Homo sapiens zinc finger, HIT type 6 (ZNHIT6), mRNA. | -0,5694 | 8,4848 | | -5,5776 | | 0,000277 | | | 0,00389 |
| FDX1L | Homo sapiens ferredoxin 1-like (FDX1L), mRNA. | -0,5696 | 8,1459 | | -6,2614 | | 0,000114 | | | 0,00239 |
| TTC15 | Homo sapiens tetratricopeptide repeat domain 15 (TTC15), mRNA. | -0,5700 | 8,8746 | | -5,9355 | | 0,000173 | | | 0,00302 |
| FASTKD2 | Homo sapiens FAST kinase domains 2 (FASTKD2), mRNA. | -0,5705 | 8,0177 | | -6,3573 | | 0,000101 | | | 0,00225 |
| SNRPC | Homo sapiens small nuclear ribonucleoprotein polypeptide C (SNRPC), mRNA. | -0,5708 | 8,4764 | | -5,5889 | | 0,000273 | | | 0,00387 |
| HINT2 | Homo sapiens histidine triad nucleotide binding protein 2 (HINT2), mRNA. | -0,5708 | 9,0639 | | -3,5587 | | 0,005578 | | | 0,02613 |
| H2AFY | Homo sapiens H2A histone family, member Y (H2AFY), transcript variant 1, mRNA. | -0,5713 | 10,1266 | | -7,5139 | | 0,000026 | | | 0,00121 |
| LOC728037 | PREDICTED: Homo sapiens similar to Kinesin-like protein KIF22 (Kinesin-like DNA-binding protein) (Kinesin-like protein 4) (LOC728037), mRNA. | -0,5713 | 7,8541 | | -4,1487 | | 0,002188 | | | 0,01368 |
| ABCB9 | Homo sapiens ATP-binding cassette, sub-family B (MDR/TAP), member 9 (ABCB9), transcript variant 2, mRNA. | -0,5716 | 7,7286 | | -5,6020 | | 0,000268 | | | 0,00385 |
| LOC644422 | PREDICTED: Homo sapiens misc_RNA (LOC644422), miscRNA. | -0,5720 | 8,1769 | | -4,2029 | | 0,002013 | | | 0,01292 |
| ZMYM1 | Homo sapiens zinc finger, MYM-type 1 (ZMYM1), mRNA. | -0,5721 | 8,1439 | | -9,0621 | | 0,000005 | | | 0,00061 |
| PDPN | Homo sapiens podoplanin (PDPN), transcript variant 4, mRNA. | -0,5724 | 9,7816 | | -3,7799 | | 0,003908 | | | 0,02023 |
| ZNF544 | Homo sapiens zinc finger protein 544 (ZNF544), mRNA. | -0,5725 | 8,4453 | | -4,9416 | | 0,000671 | | | 0,00636 |
| MFF | Homo sapiens mitochondrial fission factor (MFF), nuclear gene encoding mitochondrial protein, mRNA. | -0,5725 | 8,3904 | | -3,7895 | | 0,003848 | | | 0,02004 |
| EYA2 | Homo sapiens eyes absent homolog 2 (Drosophila) (EYA2), transcript variant 4, mRNA. | -0,5728 | 7,5925 | | -6,3384 | | 0,000104 | | | 0,00227 |
| COASY | Homo sapiens Coenzyme A synthase (COASY), nuclear gene encoding mitochondrial protein, transcript variant 1, mRNA. | -0,5730 | 8,3407 | | -11,5437 | | 0,000001 | | | 0,00029 |
| SPAG5 | Homo sapiens sperm associated antigen 5 (SPAG5), mRNA. | -0,5732 | 7,6522 | | -5,6040 | | 0,000268 | | | 0,00385 |
| ERCC6L | Homo sapiens excision repair cross-complementing rodent repair deficiency, complementation group 6-like (ERCC6L), mRNA. | -0,5732 | 7,6478 | | -7,2752 | | 0,000034 | | | 0,00134 |
| PIK3R2 | Homo sapiens phosphoinositide-3-kinase, regulatory subunit 2 (beta) (PIK3R2), mRNA. | -0,5737 | 10,0193 | | -3,3417 | | 0,007951 | | | 0,03384 |
| BRP44 | Homo sapiens brain protein 44 (BRP44), transcript variant 2, mRNA. | -0,5749 | 8,7496 | | -3,1140 | | 0,011586 | | | 0,04432 |
| TMEM9B | Homo sapiens TMEM9 domain family, member B (TMEM9B), mRNA. | -0,5754 | 8,6596 | | -5,6717 | | 0,000244 | | | 0,00364 |
| SKIV2L2 | Homo sapiens superkiller viralicidic activity 2-like 2 (S. cerevisiae) (SKIV2L2), mRNA. | -0,5758 | 8,8268 | | -4,3277 | | 0,001662 | | | 0,01136 |
| GTF2A2 | Homo sapiens general transcription factor IIA, 2, 12kDa (GTF2A2), mRNA. | -0,5773 | 10,7894 | | -6,4533 | | 0,000090 | | | 0,00213 |
| HMGB1L1 | Homo sapiens high-mobility group box 1-like 1 (HMGB1L1), mRNA. | -0,5775 | 8,6904 | | -3,9353 | | 0,003054 | | | 0,01706 |
| CCT8 | Homo sapiens chaperonin containing TCP1, subunit 8 (theta) (CCT8), mRNA. | -0,5775 | 11,6254 | | -4,6493 | | 0,001026 | | | 0,00837 |
| PRPF38A | Homo sapiens PRP38 pre-mRNA processing factor 38 (yeast) domain containing A (PRPF38A), mRNA. | -0,5776 | 7,9706 | | -9,2864 | | 0,000004 | | | 0,00054 |
| NOC3L | Homo sapiens nucleolar complex associated 3 homolog (S. cerevisiae) (NOC3L), mRNA. | -0,5776 | 8,2271 | | -3,1531 | | 0,010858 | | | 0,04220 |
| EFHC1 | Homo sapiens EF-hand domain (C-terminal) containing 1 (EFHC1), mRNA. | -0,5783 | 8,1918 | | -4,3836 | | 0,001527 | | | 0,01078 |
| PSMB5 | Homo sapiens proteasome (prosome, macropain) subunit, beta type, 5 (PSMB5), mRNA. | -0,5784 | 11,3160 | | -5,5656 | | 0,000282 | | | 0,00394 |
| GTF3C6 | Homo sapiens general transcription factor IIIC, polypeptide 6, alpha 35kDa (GTF3C6), mRNA. | -0,5788 | 8,3268 | | -11,1230 | | 0,000001 | | | 0,00031 |
| PECI | Homo sapiens peroxisomal D3,D2-enoyl-CoA isomerase (PECI), transcript variant 1, mRNA. | -0,5791 | 9,4087 | | -5,7371 | | 0,000224 | | | 0,00348 |
| PSMD6 | Homo sapiens proteasome (prosome, macropain) 26S subunit, non-ATPase, 6 (PSMD6), mRNA. | -0,5793 | 11,2262 | | -5,0428 | | 0,000580 | | | 0,00590 |
| PIGM | Homo sapiens phosphatidylinositol glycan anchor biosynthesis, class M (PIGM), mRNA. | -0,5794 | 8,4752 | | -7,1737 | | 0,000038 | | | 0,00141 |
| URB2 | Homo sapiens URB2 ribosome biogenesis 2 homolog (S. cerevisiae) (URB2), mRNA. | -0,5797 | 7,9082 | | -5,7674 | | 0,000215 | | | 0,00340 |
| EXO1 | Homo sapiens exonuclease 1 (EXO1), transcript variant 1, mRNA. | -0,5807 | 7,5650 | | -7,8903 | | 0,000017 | | | 0,00101 |
| NEK2 | Homo sapiens NIMA (never in mitosis gene a)-related kinase 2 (NEK2), mRNA. | -0,5817 | 7,6574 | | -5,3860 | | 0,000360 | | | 0,00445 |
| ANAPC4 | Homo sapiens anaphase promoting complex subunit 4 (ANAPC4), mRNA. | -0,5819 | 8,5191 | | -4,6756 | | 0,000987 | | | 0,00819 |
| COX8A | Homo sapiens cytochrome c oxidase subunit 8A (ubiquitous) (COX8A), mRNA. | -0,5819 | 12,9513 | | -3,6116 | | 0,005120 | | | 0,02454 |
| ATG4C | Homo sapiens ATG4 autophagy related 4 homolog C (S. cerevisiae) (ATG4C), transcript variant 7, mRNA. | -0,5819 | 8,0398 | | -7,8716 | | 0,000018 | | | 0,00101 |
| G3BP1 | Homo sapiens GTPase activating protein (SH3 domain) binding protein 1 (G3BP1), transcript variant 2, mRNA. | -0,5822 | 8,3334 | | -8,3212 | | 0,000011 | | | 0,00084 |
| LOC728666 | PREDICTED: Homo sapiens misc_RNA (LOC728666), miscRNA. | -0,5823 | 10,5458 | | -4,6842 | | 0,000975 | | | 0,00813 |
| SIP1 | Homo sapiens survival of motor neuron protein interacting protein 1 (SIP1), transcript variant beta, mRNA. | -0,5828 | 8,0628 | | -6,6447 | | 0,000071 | | | 0,00190 |
| C11orf59 | Homo sapiens chromosome 11 open reading frame 59 (C11orf59), mRNA. | -0,5829 | 9,3420 | | -7,4131 | | 0,000029 | | | 0,00125 |
| CTGF | Homo sapiens connective tissue growth factor (CTGF), mRNA. | -0,5830 | 7,6705 | | -3,5716 | | 0,005462 | | | 0,02571 |
| LOC652481 | PREDICTED: Homo sapiens similar to Mitochondrial import inner membrane translocase subunit Tim23 (LOC652481), mRNA. | -0,5841 | 8,4041 | | -5,2803 | | 0,000416 | | | 0,00488 |
| EIF3K | Homo sapiens eukaryotic translation initiation factor 3, subunit K (EIF3K), mRNA. | -0,5843 | 11,5002 | | -3,5394 | | 0,005755 | | | 0,02677 |
| DPH5 | Homo sapiens DPH5 homolog (S. cerevisiae) (DPH5), transcript variant 1, mRNA. | -0,5852 | 8,7289 | | -3,9503 | | 0,002983 | | | 0,01682 |
| POLR3H | Homo sapiens polymerase (RNA) III (DNA directed) polypeptide H (22.9kD) (POLR3H), transcript variant 2, mRNA. | -0,5854 | 7,8610 | | -6,5645 | | 0,000079 | | | 0,00199 |
| LRRC45 | Homo sapiens leucine rich repeat containing 45 (LRRC45), mRNA. | -0,5857 | 7,5842 | | -16,7746 | | 0,000000 | | | 0,00008 |
| TPI1 | Homo sapiens triosephosphate isomerase 1 (TPI1), mRNA. | -0,5861 | 11,6320 | | -6,8686 | | 0,000055 | | | 0,00169 |
| RPL29 | Homo sapiens ribosomal protein L29 (RPL29), mRNA. | -0,5862 | 9,4756 | | -4,5355 | | 0,001215 | | | 0,00925 |
| ZC3H4 | Homo sapiens zinc finger CCCH-type containing 4 (ZC3H4), mRNA. | -0,5864 | 8,4419 | | -5,2249 | | 0,000449 | | | 0,00509 |
| NCOA5 | Homo sapiens nuclear receptor coactivator 5 (NCOA5), mRNA. | -0,5865 | 8,3511 | | -4,0415 | | 0,002585 | | | 0,01537 |
| ZNF462 | Homo sapiens zinc finger protein 462 (ZNF462), mRNA. | -0,5865 | 8,0184 | | -4,5654 | | 0,001162 | | | 0,00903 |
| IMMT | Homo sapiens inner membrane protein, mitochondrial (mitofilin) (IMMT), nuclear gene encoding mitochondrial protein, transcript variant 2, mRNA. | -0,5868 | 10,6849 | | -3,2858 | | 0,008718 | | | 0,03608 |
| C2orf44 | Homo sapiens chromosome 2 open reading frame 44 (C2orf44), mRNA. | -0,5869 | 8,0019 | | -7,5247 | | 0,000026 | | | 0,00120 |
| ARHGEF19 | Homo sapiens Rho guanine nucleotide exchange factor (GEF) 19 (ARHGEF19), mRNA. | -0,5884 | 7,7448 | | -6,7409 | | 0,000064 | | | 0,00181 |
| NOL8 | Homo sapiens nucleolar protein 8 (NOL8), mRNA. | -0,5891 | 9,0030 | | -5,2748 | | 0,000419 | | | 0,00491 |
| ARF3 | Homo sapiens ADP-ribosylation factor 3 (ARF3), mRNA. | -0,5897 | 8,8853 | | -7,0563 | | 0,000044 | | | 0,00151 |
| ARTN | Homo sapiens artemin (ARTN), transcript variant 2, mRNA. | -0,5899 | 8,9167 | | -4,9912 | | 0,000625 | | | 0,00613 |
| LDHA | Homo sapiens lactate dehydrogenase A (LDHA), mRNA. | -0,5907 | 13,1308 | | -4,3345 | | 0,001646 | | | 0,01129 |
| DARS2 | Homo sapiens aspartyl-tRNA synthetase 2, mitochondrial (DARS2), nuclear gene encoding mitochondrial protein, mRNA. | -0,5917 | 7,9585 | | -5,1941 | | 0,000469 | | | 0,00523 |
| SPC25 | Homo sapiens SPC25, NDC80 kinetochore complex component, homolog (S. cerevisiae) (SPC25), mRNA. | -0,5924 | 7,7090 | | -5,0403 | | 0,000583 | | | 0,00590 |
| C20orf11 | Homo sapiens chromosome 20 open reading frame 11 (C20orf11), mRNA. | -0,5924 | 9,3270 | | -4,8672 | | 0,000747 | | | 0,00683 |
| MRPS16 | Homo sapiens mitochondrial ribosomal protein S16 (MRPS16), nuclear gene encoding mitochondrial protein, mRNA. | -0,5928 | 8,5972 | | -4,9197 | | 0,000692 | | | 0,00650 |
| INO80E | Homo sapiens INO80 complex subunit E (INO80E), mRNA. | -0,5928 | 9,0974 | | -6,8033 | | 0,000059 | | | 0,00174 |
| CSRP2BP | Homo sapiens CSRP2 binding protein (CSRP2BP), transcript variant 1, mRNA. | -0,5929 | 8,7753 | | -4,2602 | | 0,001843 | | | 0,01215 |
| COASY | Homo sapiens Coenzyme A synthase (COASY), transcript variant 4, mRNA. | -0,5930 | 9,8967 | | -3,9625 | | 0,002926 | | | 0,01665 |
| TRAFD1 | Homo sapiens TRAF-type zinc finger domain containing 1 (TRAFD1), mRNA. | -0,5933 | 7,9665 | | -6,7184 | | 0,000065 | | | 0,00182 |
| PTCD1 | Homo sapiens pentatricopeptide repeat domain 1 (PTCD1), mRNA. | -0,5934 | 8,8236 | | -4,4514 | | 0,001379 | | | 0,01008 |
| C17orf58 | Homo sapiens chromosome 17 open reading frame 58 (C17orf58), transcript variant 2, mRNA. | -0,5936 | 8,3946 | | -4,8632 | | 0,000751 | | | 0,00686 |
| ALDOA | Homo sapiens aldolase A, fructose-bisphosphate (ALDOA), transcript variant 2, mRNA. | -0,5936 | 12,2439 | | -5,1964 | | 0,000468 | | | 0,00522 |
| POLE | Homo sapiens polymerase (DNA directed), epsilon (POLE), mRNA. | -0,5942 | 7,9872 | | -3,7437 | | 0,004140 | | | 0,02115 |
| COIL | Homo sapiens coilin (COIL), mRNA. | -0,5943 | 8,6695 | | -5,6305 | | 0,000258 | | | 0,00376 |
| SGOL2 | Homo sapiens shugoshin-like 2 (S. pombe) (SGOL2), mRNA. | -0,5945 | 7,7927 | | -4,2681 | | 0,001821 | | | 0,01206 |
| LOC642197 | PREDICTED: Homo sapiens similar to Protein FAM82B (LOC642197), mRNA. | -0,5949 | 9,1925 | | -3,6376 | | 0,004909 | | | 0,02381 |
| UQCC | Homo sapiens ubiquinol-cytochrome c reductase complex chaperone (UQCC), nuclear gene encoding mitochondrial protein, transcript variant 2, mRNA. | -0,5953 | 8,3872 | | -5,6712 | | 0,000245 | | | 0,00364 |
| PSMC3IP | Homo sapiens PSMC3 interacting protein (PSMC3IP), transcript variant 1, mRNA. | -0,5957 | 7,8280 | | -5,7461 | | 0,000222 | | | 0,00345 |
| ZRANB2 | Homo sapiens zinc finger, RAN-binding domain containing 2 (ZRANB2), transcript variant 2, mRNA. | -0,5958 | 9,4569 | | -3,5169 | | 0,005970 | | | 0,02753 |
| EIF5A | Homo sapiens eukaryotic translation initiation factor 5A (EIF5A), mRNA. | -0,5965 | 11,5307 | | -3,4205 | | 0,006986 | | | 0,03091 |
| ELOVL6 | Homo sapiens ELOVL family member 6, elongation of long chain fatty acids (FEN1/Elo2, SUR4/Elo3-like, yeast) (ELOVL6), mRNA. | -0,5966 | 8,3528 | | -7,0431 | | 0,000045 | | | 0,00152 |
| MRPL47 | Homo sapiens mitochondrial ribosomal protein L47 (MRPL47), nuclear gene encoding mitochondrial protein, transcript variant 2, mRNA. | -0,5972 | 8,4189 | | -3,7736 | | 0,003947 | | | 0,02038 |
| MGC40489 | PREDICTED: Homo sapiens hypothetical protein MGC40489 (MGC40489), misc RNA. | -0,5978 | 7,9592 | | -3,8005 | | 0,003781 | | | 0,01977 |
| GTSE1 | Homo sapiens G-2 and S-phase expressed 1 (GTSE1), mRNA. | -0,5978 | 7,7577 | | -4,3604 | | 0,001582 | | | 0,01101 |
| UTP11L | Homo sapiens UTP11-like, U3 small nucleolar ribonucleoprotein, (yeast) (UTP11L), mRNA. | -0,5980 | 9,6410 | | -3,6185 | | 0,005063 | | | 0,02434 |
| SNRPD1 | Homo sapiens small nuclear ribonucleoprotein D1 polypeptide 16kDa (SNRPD1), mRNA. | -0,5987 | 7,7948 | | -6,5685 | | 0,000078 | | | 0,00198 |
| LOC100132457 | PREDICTED: Homo sapiens similar to Sm protein G (LOC100132457), mRNA. | -0,5996 | 10,7317 | | -4,6812 | | 0,000979 | | | 0,00815 |
| LEF1 | Homo sapiens lymphoid enhancer-binding factor 1 (LEF1), mRNA. | -0,5997 | 7,8183 | | -7,3610 | | 0,000031 | | | 0,00128 |
| MARS2 | Homo sapiens methionyl-tRNA synthetase 2, mitochondrial (MARS2), nuclear gene encoding mitochondrial protein, mRNA. | -0,5997 | 7,9269 | | -4,6301 | | 0,001056 | | | 0,00849 |
| POLR2I | Homo sapiens polymerase (RNA) II (DNA directed) polypeptide I, 14.5kDa (POLR2I), mRNA. | -0,6002 | 10,0675 | | -3,6715 | | 0,004649 | | | 0,02291 |
| TXNDC12 | Homo sapiens thioredoxin domain containing 12 (endoplasmic reticulum) (TXNDC12), mRNA. | -0,6006 | 11,0989 | | -3,9826 | | 0,002835 | | | 0,01630 |
| NDUFC1 | Homo sapiens NADH dehydrogenase (ubiquinone) 1, subcomplex unknown, 1, 6kDa (NDUFC1), mRNA. | -0,6009 | 8,7834 | | -3,9653 | | 0,002913 | | | 0,01661 |
| MRPL16 | Homo sapiens mitochondrial ribosomal protein L16 (MRPL16), nuclear gene encoding mitochondrial protein, mRNA. | -0,6011 | 8,7805 | | -6,0689 | | 0,000146 | | | 0,00275 |
| POFUT1 | Homo sapiens protein O-fucosyltransferase 1 (POFUT1), transcript variant 1, mRNA. | -0,6015 | 8,2679 | | -7,4972 | | 0,000027 | | | 0,00121 |
| RAD54L | Homo sapiens RAD54-like (S. cerevisiae) (RAD54L), mRNA. | -0,6016 | 7,9263 | | -3,6644 | | 0,004702 | | | 0,02310 |
| BRCC3 | Homo sapiens BRCA1/BRCA2-containing complex, subunit 3 (BRCC3), transcript variant 1, mRNA. | -0,6018 | 7,7872 | | -8,7702 | | 0,000007 | | | 0,00068 |
| EZH2 | Homo sapiens enhancer of zeste homolog 2 (Drosophila) (EZH2), transcript variant 2, mRNA. | -0,6024 | 7,7591 | | -5,2049 | | 0,000462 | | | 0,00519 |
| FTO | Homo sapiens fat mass and obesity associated (FTO), mRNA. | -0,6026 | 7,9483 | | -7,1048 | | 0,000042 | | | 0,00147 |
| LOC100130707 | PREDICTED: Homo sapiens hypothetical protein LOC100130707 (LOC100130707), mRNA. | -0,6027 | 9,4962 | | -5,0772 | | 0,000553 | | | 0,00573 |
| LOC100131531 | PREDICTED: Homo sapiens similar to hCG1644658 (LOC100131531), mRNA. | -0,6029 | 11,0931 | | -5,9504 | | 0,000170 | | | 0,00299 |
| CCT3 | Homo sapiens chaperonin containing TCP1, subunit 3 (gamma) (CCT3), transcript variant 3, mRNA. | -0,6031 | 9,5659 | | -5,8194 | | 0,000201 | | | 0,00328 |
| LCMT2 | Homo sapiens leucine carboxyl methyltransferase 2 (LCMT2), mRNA. | -0,6040 | 8,2188 | | -7,5398 | | 0,000025 | | | 0,00119 |
| PARK7 | Homo sapiens Parkinson disease (autosomal recessive, early onset) 7 (PARK7), mRNA. | -0,6041 | 11,7168 | | -3,7256 | | 0,004262 | | | 0,02155 |
| KLHDC5 | Homo sapiens kelch domain containing 5 (KLHDC5), mRNA. | -0,6045 | 9,1806 | | -5,5378 | | 0,000293 | | | 0,00402 |
| MRPS23 | Homo sapiens mitochondrial ribosomal protein S23 (MRPS23), nuclear gene encoding mitochondrial protein, mRNA. | -0,6050 | 8,8557 | | -5,8657 | | 0,000189 | | | 0,00317 |
| PRIM2A | PREDICTED: Homo sapiens primase, polypeptide 2A, 58kDa (PRIM2A), mRNA. | -0,6050 | 7,9718 | | -10,5080 | | 0,000001 | | | 0,00038 |
| NUF2 | Homo sapiens NUF2, NDC80 kinetochore complex component, homolog (S. cerevisiae) (NUF2), transcript variant 2, mRNA. | -0,6050 | 7,8058 | | -3,1245 | | 0,011387 | | | 0,04376 |
| PRPF19 | Homo sapiens PRP19/PSO4 pre-mRNA processing factor 19 homolog (S. cerevisiae) (PRPF19), mRNA. | -0,6052 | 8,2176 | | -5,6968 | | 0,000236 | | | 0,00357 |
| ICT1 | Homo sapiens immature colon carcinoma transcript 1 (ICT1), mRNA. | -0,6052 | 9,0223 | | -5,0252 | | 0,000595 | | | 0,00597 |
| RPS26 | Homo sapiens ribosomal protein S26 (RPS26), mRNA. | -0,6058 | 7,7591 | | -6,8623 | | 0,000055 | | | 0,00169 |
| SLC25A4 | Homo sapiens solute carrier family 25 (mitochondrial carrier; adenine nucleotide translocator), member 4 (SLC25A4), nuclear gene encoding mitochondrial protein, mRNA. | -0,6058 | 8,4133 | | -4,4599 | | 0,001361 | | | 0,00999 |
| TAF15 | Homo sapiens TAF15 RNA polymerase II, TATA box binding protein (TBP)-associated factor, 68kDa (TAF15), transcript variant 1, mRNA. | -0,6060 | 10,3629 | | -3,2379 | | 0,009434 | | | 0,03810 |
| PSMB1 | Homo sapiens proteasome (prosome, macropain) subunit, beta type, 1 (PSMB1), mRNA. | -0,6063 | 12,6321 | | -6,7961 | | 0,000060 | | | 0,00175 |
| LOC440957 | Homo sapiens similar to CG32736-PA (LOC440957), mRNA. | -0,6063 | 8,2522 | | -5,2974 | | 0,000406 | | | 0,00481 |
| U2AF1 | Homo sapiens U2 small nuclear RNA auxiliary factor 1 (U2AF1), transcript variant b, mRNA. | -0,6066 | 9,0093 | | -5,3016 | | 0,000404 | | | 0,00479 |
| EPM2AIP1 | Homo sapiens EPM2A (laforin) interacting protein 1 (EPM2AIP1), mRNA. | -0,6068 | 7,9107 | | -10,2602 | | 0,000002 | | | 0,00041 |
| MRPL4 | Homo sapiens mitochondrial ribosomal protein L4 (MRPL4), nuclear gene encoding mitochondrial protein, transcript variant 2, mRNA. | -0,6069 | 8,5674 | | -4,4366 | | 0,001410 | | | 0,01022 |
| THOC3 | Homo sapiens THO complex 3 (THOC3), mRNA. | -0,6073 | 9,1189 | | -5,6441 | | 0,000254 | | | 0,00372 |
| MXRA5 | Homo sapiens matrix-remodelling associated 5 (MXRA5), mRNA. | -0,6080 | 7,6228 | | -4,7622 | | 0,000870 | | | 0,00757 |
| ATP1B3 | PREDICTED: Homo sapiens ATPase, Na+/K+ transporting, beta 3 polypeptide, transcript variant 2 (ATP1B3), mRNA. | -0,6083 | 10,2818 | | -5,3032 | | 0,000403 | | | 0,00479 |
| EIF2AK1 | Homo sapiens eukaryotic translation initiation factor 2-alpha kinase 1 (EIF2AK1), mRNA. | -0,6086 | 11,5740 | | -4,4933 | | 0,001295 | | | 0,00964 |
| COPS7B | Homo sapiens COP9 constitutive photomorphogenic homolog subunit 7B (Arabidopsis) (COPS7B), mRNA. | -0,6086 | 8,4705 | | -4,5721 | | 0,001151 | | | 0,00898 |
| LOC402175 | PREDICTED: Homo sapiens misc_RNA (LOC402175), miscRNA. | -0,6092 | 11,3190 | | -8,0198 | | 0,000015 | | | 0,00095 |
| ANAPC11 | Homo sapiens anaphase promoting complex subunit 11 (ANAPC11), transcript variant 4, mRNA. | -0,6093 | 8,7996 | | -6,2175 | | 0,000121 | | | 0,00245 |
| C20orf3 | Homo sapiens chromosome 20 open reading frame 3 (C20orf3), mRNA. | -0,6094 | 9,3086 | | -3,0508 | | 0,012874 | | | 0,04817 |
| ACACA | Homo sapiens acetyl-Coenzyme A carboxylase alpha (ACACA), transcript variant 3, mRNA. | -0,6102 | 8,8147 | | -3,2390 | | 0,009417 | | | 0,03805 |
| NSUN2 | Homo sapiens NOP2/Sun domain family, member 2 (NSUN2), mRNA. | -0,6102 | 11,0333 | | -5,3984 | | 0,000354 | | | 0,00441 |
| NUP62 | Homo sapiens nucleoporin 62kDa (NUP62), transcript variant 1, mRNA. | -0,6103 | 8,2909 | | -7,7251 | | 0,000021 | | | 0,00109 |
| TSEN34 | Homo sapiens tRNA splicing endonuclease 34 homolog (S. cerevisiae) (TSEN34), transcript variant 2, mRNA. | -0,6107 | 9,7379 | | -5,1758 | | 0,000481 | | | 0,00531 |
| LOC643668 | PREDICTED: Homo sapiens similar to peptidase (prosome, macropain) 26S subunit, ATPase 1 (LOC643668), mRNA. | -0,6109 | 10,5358 | | -5,1074 | | 0,000530 | | | 0,00560 |
| ATP5B | Homo sapiens ATP synthase, H+ transporting, mitochondrial F1 complex, beta polypeptide (ATP5B), nuclear gene encoding mitochondrial protein, mRNA. | -0,6110 | 13,1828 | | -4,3839 | | 0,001527 | | | 0,01078 |
| EIF2B1 | Homo sapiens eukaryotic translation initiation factor 2B, subunit 1 alpha, 26kDa (EIF2B1), mRNA. | -0,6116 | 8,7352 | | -4,0213 | | 0,002668 | | | 0,01567 |
| ALAD | Homo sapiens aminolevulinate, delta-, dehydratase (ALAD), transcript variant 1, mRNA. | -0,6117 | 7,9624 | | -6,3894 | | 0,000097 | | | 0,00220 |
| MUM1 | Homo sapiens melanoma associated antigen (mutated) 1 (MUM1), mRNA. | -0,6119 | 8,1606 | | -6,3088 | | 0,000108 | | | 0,00231 |
| EPHB4 | Homo sapiens EPH receptor B4 (EPHB4), mRNA. | -0,6121 | 8,9289 | | -3,5465 | | 0,005690 | | | 0,02655 |
| RNF121 | Homo sapiens ring finger protein 121 (RNF121), transcript variant 1, mRNA. | -0,6121 | 8,5754 | | -5,9237 | | 0,000176 | | | 0,00305 |
| NAGPA | Homo sapiens N-acetylglucosamine-1-phosphodiester alpha-N-acetylglucosaminidase (NAGPA), mRNA. | -0,6123 | 7,9724 | | -5,6865 | | 0,000240 | | | 0,00360 |
| SETMAR | Homo sapiens SET domain and mariner transposase fusion gene (SETMAR), mRNA. | -0,6123 | 7,7216 | | -7,6143 | | 0,000023 | | | 0,00115 |
| DGCR6L | Homo sapiens DiGeorge syndrome critical region gene 6-like (DGCR6L), mRNA. | -0,6128 | 9,0074 | | -3,4235 | | 0,006952 | | | 0,03079 |
| SLC2A8 | Homo sapiens solute carrier family 2 (facilitated glucose transporter), member 8 (SLC2A8), mRNA. | -0,6130 | 8,4542 | | -5,3488 | | 0,000378 | | | 0,00458 |
| PPIL5 | Homo sapiens peptidylprolyl isomerase (cyclophilin)-like 5 (PPIL5), transcript variant 1, mRNA. | -0,6131 | 7,8015 | | -9,7485 | | 0,000003 | | | 0,00049 |
| DHX29 | Homo sapiens DEAH (Asp-Glu-Ala-His) box polypeptide 29 (DHX29), mRNA. | -0,6133 | 9,3410 | | -6,0251 | | 0,000154 | | | 0,00282 |
| NOP56 | Homo sapiens NOP56 ribonucleoprotein homolog (yeast) (NOP56), transcript variant 1, mRNA. | -0,6136 | 8,3676 | | -3,5219 | | 0,005921 | | | 0,02738 |
| STOML2 | Homo sapiens stomatin (EPB72)-like 2 (STOML2), mRNA. | -0,6138 | 10,8644 | | -4,9249 | | 0,000687 | | | 0,00647 |
| MRPS31 | Homo sapiens mitochondrial ribosomal protein S31 (MRPS31), nuclear gene encoding mitochondrial protein, mRNA. | -0,6142 | 8,8214 | | -9,5035 | | 0,000004 | | | 0,00052 |
| GFM1 | Homo sapiens G elongation factor, mitochondrial 1 (GFM1), nuclear gene encoding mitochondrial protein, mRNA. | -0,6145 | 8,6911 | | -3,2766 | | 0,008850 | | | 0,03647 |
| PSMD5 | Homo sapiens proteasome (prosome, macropain) 26S subunit, non-ATPase, 5 (PSMD5), mRNA. | -0,6148 | 7,9532 | | -10,2141 | | 0,000002 | | | 0,00041 |
| KATNB1 | Homo sapiens katanin p80 (WD repeat containing) subunit B 1 (KATNB1), mRNA. | -0,6150 | 8,2830 | | -4,6354 | | 0,001048 | | | 0,00846 |
| ARL5A | Homo sapiens ADP-ribosylation factor-like 5A (ARL5A), transcript variant 3, mRNA. | -0,6152 | 10,7893 | | -5,4130 | | 0,000347 | | | 0,00437 |
| EZH2 | Homo sapiens enhancer of zeste homolog 2 (Drosophila) (EZH2), transcript variant 2, mRNA. | -0,6155 | 7,8063 | | -5,4374 | | 0,000335 | | | 0,00432 |
| CUTC | Homo sapiens cutC copper transporter homolog (E. coli) (CUTC), mRNA. | -0,6157 | 8,7070 | | -3,9355 | | 0,003053 | | | 0,01706 |
| C20orf29 | Homo sapiens chromosome 20 open reading frame 29 (C20orf29), mRNA. | -0,6161 | 8,2248 | | -6,7710 | | 0,000061 | | | 0,00178 |
| KAT2A | Homo sapiens K(lysine) acetyltransferase 2A (KAT2A), mRNA. | -0,6164 | 8,5329 | | -3,6578 | | 0,004752 | | | 0,02325 |
| HAUS8 | Homo sapiens HAUS augmin-like complex, subunit 8 (HAUS8), transcript variant 1, mRNA. | -0,6165 | 7,8015 | | -5,5929 | | 0,000272 | | | 0,00386 |
| PSMD6 | Homo sapiens proteasome (prosome, macropain) 26S subunit, non-ATPase, 6 (PSMD6), mRNA. | -0,6167 | 9,8514 | | -7,2664 | | 0,000035 | | | 0,00134 |
| TACO1 | Homo sapiens translational activator of mitochondrially encoded cytochrome c oxidase I (TACO1), nuclear gene encoding mitochondrial protein, mRNA. | -0,6174 | 8,8100 | | -5,7239 | | 0,000228 | | | 0,00351 |
| SNUPN | Homo sapiens snurportin 1 (SNUPN), transcript variant 2, mRNA. | -0,6177 | 8,3545 | | -6,6278 | | 0,000073 | | | 0,00191 |
| CEP78 | Homo sapiens centrosomal protein 78kDa (CEP78), transcript variant 2, mRNA. | -0,6179 | 7,8555 | | -4,3891 | | 0,001515 | | | 0,01072 |
| BRMS1L | Homo sapiens breast cancer metastasis-suppressor 1-like (BRMS1L), mRNA. | -0,6182 | 8,0368 | | -4,6924 | | 0,000963 | | | 0,00807 |
| DDX46 | Homo sapiens DEAD (Asp-Glu-Ala-Asp) box polypeptide 46 (DDX46), mRNA. | -0,6190 | 7,7682 | | -6,6958 | | 0,000067 | | | 0,00184 |
| LOC647150 | PREDICTED: Homo sapiens misc_RNA (LOC647150), miscRNA. | -0,6190 | 8,8853 | | -3,0702 | | 0,012463 | | | 0,04692 |
| MMACHC | Homo sapiens methylmalonic aciduria (cobalamin deficiency) cblC type, with homocystinuria (MMACHC), mRNA. | -0,6194 | 7,6891 | | -6,8443 | | 0,000056 | | | 0,00170 |
| SLC5A6 | Homo sapiens solute carrier family 5 (sodium-dependent vitamin transporter), member 6 (SLC5A6), mRNA. | -0,6203 | 8,8785 | | -3,4864 | | 0,006274 | | | 0,02851 |
| HSPA4 | Homo sapiens heat shock 70kDa protein 4 (HSPA4), mRNA. | -0,6203 | 7,7430 | | -5,1182 | | 0,000522 | | | 0,00554 |
| H3F3A | Homo sapiens H3 histone, family 3A (H3F3A), mRNA. | -0,6210 | 12,6122 | | -5,3758 | | 0,000365 | | | 0,00448 |
| NME1 | Homo sapiens non-metastatic cells 1, protein (NM23A) expressed in (NME1), transcript variant 2, mRNA. | -0,6213 | 8,0402 | | -3,9253 | | 0,003102 | | | 0,01724 |
| WDR75 | Homo sapiens WD repeat domain 75 (WDR75), mRNA. | -0,6217 | 9,4019 | | -4,6257 | | 0,001063 | | | 0,00853 |
| APITD1 | Homo sapiens apoptosis-inducing, TAF9-like domain 1 (APITD1), transcript variant B, mRNA. | -0,6218 | 7,6119 | | -10,8623 | | 0,000001 | | | 0,00032 |
| BRI3BP | Homo sapiens BRI3 binding protein (BRI3BP), mRNA. | -0,6226 | 7,8140 | | -7,0435 | | 0,000045 | | | 0,00152 |
| ITGB3BP | Homo sapiens integrin beta 3 binding protein (beta3-endonexin) (ITGB3BP), mRNA. | -0,6228 | 7,9764 | | -3,0209 | | 0,013531 | | | 0,04990 |
| C6orf153 | Homo sapiens chromosome 6 open reading frame 153 (C6orf153), mRNA. | -0,6228 | 9,2952 | | -4,1216 | | 0,002282 | | | 0,01404 |
| ZNF207 | Homo sapiens zinc finger protein 207 (ZNF207), transcript variant 2, mRNA. | -0,6231 | 10,5926 | | -4,3305 | | 0,001655 | | | 0,01133 |
| ANGEL2 | Homo sapiens angel homolog 2 (Drosophila) (ANGEL2), mRNA. | -0,6235 | 8,4960 | | -5,1193 | | 0,000521 | | | 0,00553 |
| SERTAD4 | Homo sapiens SERTA domain containing 4 (SERTAD4), mRNA. | -0,6238 | 8,0008 | | -4,3992 | | 0,001492 | | | 0,01062 |
| RFC2 | Homo sapiens replication factor C (activator 1) 2, 40kDa (RFC2), transcript variant 1, mRNA. | -0,6238 | 7,7933 | | -6,4841 | | 0,000087 | | | 0,00208 |
| FLJ20718 | Homo sapiens hypothetical protein FLJ20718 (FLJ20718), transcript variant 1, mRNA. | -0,6241 | 8,1532 | | -4,2135 | | 0,001980 | | | 0,01279 |
| LOC100134189 | PREDICTED: Homo sapiens similar to cofactor required for Sp1 transcriptional activation, subunit 8, 34kDa, transcript variant 2 (LOC100134189), mRNA. | -0,6241 | 8,4621 | | -6,7785 | | 0,000061 | | | 0,00178 |
| OMA1 | Homo sapiens OMA1 homolog, zinc metallopeptidase (S. cerevisiae) (OMA1), mRNA. | -0,6243 | 8,0948 | | -3,4827 | | 0,006312 | | | 0,02863 |
| TNFRSF21 | Homo sapiens tumor necrosis factor receptor superfamily, member 21 (TNFRSF21), mRNA. | -0,6244 | 10,6040 | | -3,0843 | | 0,012175 | | | 0,04608 |
| MND1 | Homo sapiens meiotic nuclear divisions 1 homolog (S. cerevisiae) (MND1), mRNA. | -0,6244 | 7,8052 | | -3,3325 | | 0,008071 | | | 0,03421 |
| OTUB1 | Homo sapiens OTU domain, ubiquitin aldehyde binding 1 (OTUB1), mRNA. | -0,6245 | 9,1638 | | -6,9728 | | 0,000048 | | | 0,00158 |
| C10orf78 | Homo sapiens chromosome 10 open reading frame 78 (C10orf78), transcript variant 2, mRNA. | -0,6247 | 8,3970 | | -5,8159 | | 0,000202 | | | 0,00329 |
| TIMM23 | Homo sapiens translocase of inner mitochondrial membrane 23 homolog (yeast) (TIMM23), nuclear gene encoding mitochondrial protein, mRNA. | -0,6252 | 9,4959 | | -4,9005 | | 0,000711 | | | 0,00662 |
| SDHD | Homo sapiens succinate dehydrogenase complex, subunit D, integral membrane protein (SDHD), nuclear gene encoding mitochondrial protein, mRNA. | -0,6253 | 9,1876 | | -3,7881 | | 0,003856 | | | 0,02007 |
| ATP6V1A | Homo sapiens ATPase, H+ transporting, lysosomal 70kDa, V1 subunit A (ATP6V1A), mRNA. | -0,6254 | 9,6329 | | -4,0275 | | 0,002643 | | | 0,01558 |
| C17orf58 | Homo sapiens chromosome 17 open reading frame 58 (C17orf58), transcript variant 2, mRNA. | -0,6260 | 8,8672 | | -5,4503 | | 0,000329 | | | 0,00429 |
| LOC100130003 | PREDICTED: Homo sapiens misc_RNA (LOC100130003), miscRNA. | -0,6263 | 11,8999 | | -4,5913 | | 0,001118 | | | 0,00881 |
| DNAJC9 | Homo sapiens DnaJ (Hsp40) homolog, subfamily C, member 9 (DNAJC9), mRNA. | -0,6265 | 10,2210 | | -4,3818 | | 0,001532 | | | 0,01079 |
| NUAK1 | Homo sapiens NUAK family, SNF1-like kinase, 1 (NUAK1), mRNA. | -0,6268 | 8,3505 | | -3,8059 | | 0,003749 | | | 0,01966 |
| HSPA2 | Homo sapiens heat shock 70kDa protein 2 (HSPA2), mRNA. | -0,6269 | 7,6095 | | -10,9188 | | 0,000001 | | | 0,00031 |
| ANAPC7 | Homo sapiens anaphase promoting complex subunit 7 (ANAPC7), mRNA. | -0,6270 | 7,9311 | | -8,0915 | | 0,000014 | | | 0,00093 |
| CHPT1 | Homo sapiens choline phosphotransferase 1 (CHPT1), mRNA. | -0,6270 | 8,6267 | | -4,3979 | | 0,001495 | | | 0,01062 |
| ATP5J | Homo sapiens ATP synthase, H+ transporting, mitochondrial F0 complex, subunit F6 (ATP5J), nuclear gene encoding mitochondrial protein, transcript variant 1, mRNA. | -0,6272 | 8,5652 | | -5,7671 | | 0,000215 | | | 0,00340 |
| PGK1 | Homo sapiens phosphoglycerate kinase 1 (PGK1), mRNA. | -0,6275 | 11,7438 | | -4,9233 | | 0,000689 | | | 0,00648 |
| TUBA1C | Homo sapiens tubulin, alpha 1c (TUBA1C), mRNA. | -0,6276 | 13,5273 | | -5,0456 | | 0,000578 | | | 0,00588 |
| PSMB8 | Homo sapiens proteasome (prosome, macropain) subunit, beta type, 8 (large multifunctional peptidase 7) (PSMB8), transcript variant 2, mRNA. | -0,6277 | 7,8887 | | -7,8417 | | 0,000018 | | | 0,00103 |
| BLCAP | Homo sapiens bladder cancer associated protein (BLCAP), mRNA. | -0,6283 | 10,0754 | | -4,4327 | | 0,001418 | | | 0,01024 |
| LOC440926 | Homo sapiens H3 histone, family 3A pseudogene (LOC440926), non-coding RNA. | -0,6288 | 12,6199 | | -4,5735 | | 0,001148 | | | 0,00897 |
| CISD1 | Homo sapiens CDGSH iron sulfur domain 1 (CISD1), mRNA. | -0,6302 | 10,4026 | | -5,0195 | | 0,000600 | | | 0,00600 |
| SUMO2 | Homo sapiens SMT3 suppressor of mif two 3 homolog 2 (S. cerevisiae) (SUMO2), transcript variant 2, mRNA. | -0,6307 | 11,0460 | | -5,0355 | | 0,000587 | | | 0,00593 |
| GBA | Homo sapiens glucosidase, beta; acid (includes glucosylceramidase) (GBA), transcript variant 3, mRNA. | -0,6308 | 8,3923 | | -5,1588 | | 0,000493 | | | 0,00538 |
| FAM171A1 | Homo sapiens family with sequence similarity 171, member A1 (FAM171A1), mRNA. | -0,6308 | 9,5954 | | -9,3165 | | 0,000004 | | | 0,00054 |
| JAG2 | Homo sapiens jagged 2 (JAG2), transcript variant 2, mRNA. | -0,6309 | 7,9816 | | -5,2867 | | 0,000412 | | | 0,00485 |
| APOA1BP | Homo sapiens apolipoprotein A-I binding protein (APOA1BP), mRNA. | -0,6310 | 9,7199 | | -5,5922 | | 0,000272 | | | 0,00386 |
| SDHB | Homo sapiens succinate dehydrogenase complex, subunit B, iron sulfur (Ip) (SDHB), nuclear gene encoding mitochondrial protein, mRNA. | -0,6311 | 11,4936 | | -6,1067 | | 0,000139 | | | 0,00267 |
| ATP1B3 | PREDICTED: Homo sapiens ATPase, Na+/K+ transporting, beta 3 polypeptide, transcript variant 2 (ATP1B3), mRNA. | -0,6313 | 11,1412 | | -4,5966 | | 0,001110 | | | 0,00878 |
| GALNT11 | Homo sapiens UDP-N-acetyl-alpha-D-galactosamine:polypeptide N-acetylgalactosaminyltransferase 11 (GalNAc-T11) (GALNT11), mRNA. | -0,6315 | 8,8435 | | -4,8507 | | 0,000765 | | | 0,00695 |
| ATP5J2 | Homo sapiens ATP synthase, H+ transporting, mitochondrial F0 complex, subunit F2 (ATP5J2), nuclear gene encoding mitochondrial protein, transcript variant 3, mRNA. | -0,6319 | 11,3007 | | -5,4694 | | 0,000321 | | | 0,00423 |
| EIF3B | Homo sapiens eukaryotic translation initiation factor 3, subunit B (EIF3B), transcript variant 1, mRNA. | -0,6322 | 10,9862 | | -4,8042 | | 0,000818 | | | 0,00725 |
| TCP1 | Homo sapiens t-complex 1 (TCP1), transcript variant 1, mRNA. | -0,6323 | 9,5827 | | -7,3297 | | 0,000032 | | | 0,00129 |
| LSM7 | Homo sapiens LSM7 homolog, U6 small nuclear RNA associated (S. cerevisiae) (LSM7), mRNA. | -0,6327 | 10,3171 | | -3,3425 | | 0,007940 | | | 0,03382 |
| POLQ | Homo sapiens polymerase (DNA directed), theta (POLQ), mRNA. | -0,6336 | 7,8790 | | -4,2853 | | 0,001774 | | | 0,01187 |
| FAM195A | Homo sapiens family with sequence similarity 195, member A (FAM195A), mRNA. | -0,6338 | 8,5520 | | -5,7938 | | 0,000208 | | | 0,00333 |
| AP2S1 | Homo sapiens adaptor-related protein complex 2, sigma 1 subunit (AP2S1), transcript variant AP17, mRNA. | -0,6341 | 12,5308 | | -4,2971 | | 0,001742 | | | 0,01175 |
| ACP1 | Homo sapiens acid phosphatase 1, soluble (ACP1), transcript variant 2, mRNA. | -0,6343 | 9,8454 | | -4,9770 | | 0,000638 | | | 0,00620 |
| C20orf177 | Homo sapiens chromosome 20 open reading frame 177 (C20orf177), mRNA. | -0,6344 | 8,4242 | | -3,2304 | | 0,009552 | | | 0,03842 |
| LOC93622 | PREDICTED: Homo sapiens hypothetical protein BC006130 (LOC93622), misc RNA. | -0,6348 | 8,0655 | | -7,6281 | | 0,000023 | | | 0,00115 |
| TOMM6 | Homo sapiens translocase of outer mitochondrial membrane 6 homolog (yeast) (TOMM6), nuclear gene encoding mitochondrial protein, mRNA. | -0,6354 | 10,5676 | | -3,6129 | | 0,005109 | | | 0,02451 |
| BUB3 | Homo sapiens BUB3 budding uninhibited by benzimidazoles 3 homolog (yeast) (BUB3), transcript variant 2, mRNA. | -0,6356 | 8,4334 | | -3,3479 | | 0,007869 | | | 0,03365 |
| ATP5H | Homo sapiens ATP synthase, H+ transporting, mitochondrial F0 complex, subunit d (ATP5H), nuclear gene encoding mitochondrial protein, transcript variant 2, mRNA. | -0,6363 | 12,4601 | | -3,1406 | | 0,011086 | | | 0,04288 |
| SNRPG | Homo sapiens small nuclear ribonucleoprotein polypeptide G (SNRPG), mRNA. | -0,6364 | 12,3222 | | -4,4136 | | 0,001459 | | | 0,01046 |
| TSNAX | Homo sapiens translin-associated factor X (TSNAX), mRNA. | -0,6364 | 8,4791 | | -8,9975 | | 0,000006 | | | 0,00064 |
| GTF3C2 | Homo sapiens general transcription factor IIIC, polypeptide 2, beta 110kDa (GTF3C2), transcript variant 1, mRNA. | -0,6369 | 9,4997 | | -7,0253 | | 0,000046 | | | 0,00153 |
| SREBF1 | Homo sapiens sterol regulatory element binding transcription factor 1 (SREBF1), transcript variant 1, mRNA. | -0,6373 | 8,1355 | | -5,1407 | | 0,000506 | | | 0,00544 |
| BANF1 | Homo sapiens barrier to autointegration factor 1 (BANF1), mRNA. | -0,6383 | 8,7546 | | -5,2564 | | 0,000430 | | | 0,00497 |
| C11orf74 | Homo sapiens chromosome 11 open reading frame 74 (C11orf74), mRNA. | -0,6383 | 8,3117 | | -4,3383 | | 0,001636 | | | 0,01123 |
| GALK1 | Homo sapiens galactokinase 1 (GALK1), mRNA. | -0,6384 | 8,1709 | | -3,4133 | | 0,007069 | | | 0,03118 |
| TMEM48 | Homo sapiens transmembrane protein 48 (TMEM48), mRNA. | -0,6384 | 8,3439 | | -6,0579 | | 0,000148 | | | 0,00276 |
| TBC1D7 | Homo sapiens TBC1 domain family, member 7 (TBC1D7), mRNA. | -0,6387 | 8,9572 | | -4,3759 | | 0,001545 | | | 0,01085 |
| MRPS35 | Homo sapiens mitochondrial ribosomal protein S35 (MRPS35), nuclear gene encoding mitochondrial protein, mRNA. | -0,6388 | 9,2064 | | -4,2506 | | 0,001870 | | | 0,01228 |
| PAAF1 | Homo sapiens proteasomal ATPase-associated factor 1 (PAAF1), mRNA. | -0,6397 | 8,0417 | | -5,0040 | | 0,000613 | | | 0,00609 |
| KCTD3 | Homo sapiens potassium channel tetramerisation domain containing 3 (KCTD3), mRNA. | -0,6397 | 8,5245 | | -6,7549 | | 0,000063 | | | 0,00180 |
| PPP1CC | Homo sapiens protein phosphatase 1, catalytic subunit, gamma isoform (PPP1CC), mRNA. | -0,6399 | 11,1322 | | -5,0800 | | 0,000551 | | | 0,00572 |
| KTELC1 | Homo sapiens KTEL (Lys-Tyr-Glu-Leu) containing 1 (KTELC1), mRNA. | -0,6400 | 8,6821 | | -4,6638 | | 0,001005 | | | 0,00827 |
| CP110 | Homo sapiens CP110 protein (CP110), mRNA. | -0,6400 | 7,9953 | | -4,7842 | | 0,000842 | | | 0,00741 |
| IARS2 | Homo sapiens isoleucyl-tRNA synthetase 2, mitochondrial (IARS2), nuclear gene encoding mitochondrial protein, mRNA. | -0,6412 | 10,6638 | | -4,9001 | | 0,000712 | | | 0,00662 |
| NOP58 | Homo sapiens NOP58 ribonucleoprotein homolog (yeast) (NOP58), mRNA. | -0,6413 | 9,8965 | | -3,2797 | | 0,008805 | | | 0,03633 |
| POLR2F | Homo sapiens polymerase (RNA) II (DNA directed) polypeptide F (POLR2F), mRNA. | -0,6415 | 10,5307 | | -4,9293 | | 0,000683 | | | 0,00644 |
| DNAJA3 | Homo sapiens DnaJ (Hsp40) homolog, subfamily A, member 3 (DNAJA3), mRNA. | -0,6419 | 10,0507 | | -3,4857 | | 0,006280 | | | 0,02853 |
| CDC2 | Homo sapiens cell division cycle 2, G1 to S and G2 to M (CDC2), transcript variant 1, mRNA. | -0,6422 | 7,7585 | | -4,5697 | | 0,001155 | | | 0,00899 |
| EXTL2 | Homo sapiens exostoses (multiple)-like 2 (EXTL2), transcript variant 1, mRNA. | -0,6425 | 7,9902 | | -6,4646 | | 0,000089 | | | 0,00211 |
| LOC400013 | PREDICTED: Homo sapiens misc_RNA (LOC400013), miscRNA. | -0,6425 | 10,5184 | | -3,9836 | | 0,002830 | | | 0,01628 |
| NIP7 | Homo sapiens nuclear import 7 homolog (S. cerevisiae) (NIP7), mRNA. | -0,6426 | 9,9575 | | -3,7963 | | 0,003806 | | | 0,01987 |
| DIS3L | Homo sapiens DIS3 mitotic control homolog (S. cerevisiae)-like (DIS3L), mRNA. | -0,6430 | 8,0647 | | -7,5349 | | 0,000026 | | | 0,00119 |
| HIRIP3 | Homo sapiens HIRA interacting protein 3 (HIRIP3), mRNA. | -0,6433 | 7,7264 | | -7,3622 | | 0,000031 | | | 0,00128 |
| RAB34 | Homo sapiens RAB34, member RAS oncogene family (RAB34), mRNA. | -0,6433 | 9,6016 | | -7,1451 | | 0,000040 | | | 0,00144 |
| NCL | Homo sapiens nucleolin (NCL), mRNA. | -0,6435 | 11,7663 | | -3,6407 | | 0,004885 | | | 0,02373 |
| PDGFC | Homo sapiens platelet derived growth factor C (PDGFC), mRNA. | -0,6436 | 7,9948 | | -6,4450 | | 0,000091 | | | 0,00214 |
| KCTD3 | Homo sapiens potassium channel tetramerisation domain containing 3 (KCTD3), mRNA. | -0,6436 | 9,0251 | | -7,1664 | | 0,000039 | | | 0,00141 |
| MRPS26 | Homo sapiens mitochondrial ribosomal protein S26 (MRPS26), nuclear gene encoding mitochondrial protein, mRNA. | -0,6444 | 8,8416 | | -5,0342 | | 0,000588 | | | 0,00593 |
| JAG2 | Homo sapiens jagged 2 (JAG2), transcript variant 1, mRNA. | -0,6446 | 8,3118 | | -4,0488 | | 0,002556 | | | 0,01526 |
| HDAC2 | Homo sapiens histone deacetylase 2 (HDAC2), mRNA. | -0,6462 | 10,6323 | | -3,8743 | | 0,003363 | | | 0,01826 |
| NDUFB2 | Homo sapiens NADH dehydrogenase (ubiquinone) 1 beta subcomplex, 2, 8kDa (NDUFB2), nuclear gene encoding mitochondrial protein, mRNA. | -0,6463 | 10,7243 | | -3,9884 | | 0,002809 | | | 0,01620 |
| LRRC20 | Homo sapiens leucine rich repeat containing 20 (LRRC20), transcript variant 2, mRNA. | -0,6470 | 7,6477 | | -5,7123 | | 0,000232 | | | 0,00354 |
| ORC1L | Homo sapiens origin recognition complex, subunit 1-like (yeast) (ORC1L), mRNA. | -0,6471 | 7,6148 | | -9,2571 | | 0,000004 | | | 0,00055 |
| ENSA | Homo sapiens endosulfine alpha (ENSA), transcript variant 8, mRNA. | -0,6474 | 8,7994 | | -4,7628 | | 0,000869 | | | 0,00757 |
| CIRH1A | Homo sapiens cirrhosis, autosomal recessive 1A (cirhin) (CIRH1A), mRNA. | -0,6475 | 10,2598 | | -4,0310 | | 0,002628 | | | 0,01554 |
| POLR2L | Homo sapiens polymerase (RNA) II (DNA directed) polypeptide L, 7.6kDa (POLR2L), mRNA. | -0,6478 | 7,6531 | | -6,2055 | | 0,000122 | | | 0,00247 |
| ARL6IP1 | Homo sapiens ADP-ribosylation factor-like 6 interacting protein 1 (ARL6IP1), mRNA. | -0,6485 | 11,0878 | | -3,1351 | | 0,011187 | | | 0,04317 |
| C20orf72 | Homo sapiens chromosome 20 open reading frame 72 (C20orf72), mRNA. | -0,6488 | 9,1314 | | -3,9463 | | 0,003001 | | | 0,01690 |
| IMPDH2 | Homo sapiens IMP (inosine monophosphate) dehydrogenase 2 (IMPDH2), mRNA. | -0,6489 | 11,2198 | | -3,6227 | | 0,005029 | | | 0,02424 |
| HNRNPC | Homo sapiens heterogeneous nuclear ribonucleoprotein C (C1/C2) (HNRNPC), transcript variant 3, mRNA. | -0,6492 | 8,1753 | | -4,3841 | | 0,001526 | | | 0,01078 |
| LOC649553 | PREDICTED: Homo sapiens misc_RNA (LOC649553), miscRNA. | -0,6506 | 12,6531 | | -5,8386 | | 0,000196 | | | 0,00322 |
| NDUFB10 | Homo sapiens NADH dehydrogenase (ubiquinone) 1 beta subcomplex, 10, 22kDa (NDUFB10), mRNA. | -0,6511 | 10,1883 | | -3,9618 | | 0,002929 | | | 0,01666 |
| C1orf112 | Homo sapiens chromosome 1 open reading frame 112 (C1orf112), mRNA. | -0,6513 | 7,8691 | | -4,2657 | | 0,001828 | | | 0,01208 |
| TIMM23 | Homo sapiens translocase of inner mitochondrial membrane 23 homolog (yeast) (TIMM23), nuclear gene encoding mitochondrial protein, mRNA. | -0,6517 | 9,5588 | | -5,4425 | | 0,000333 | | | 0,00431 |
| SLC39A11 | Homo sapiens solute carrier family 39 (metal ion transporter), member 11 (SLC39A11), mRNA. | -0,6519 | 8,2330 | | -4,3994 | | 0,001491 | | | 0,01062 |
| EMG1 | Homo sapiens EMG1 nucleolar protein homolog (S. cerevisiae) (EMG1), mRNA. | -0,6520 | 10,1862 | | -3,7380 | | 0,004178 | | | 0,02128 |
| MPPE1 | Homo sapiens metallophosphoesterase 1 (MPPE1), mRNA. | -0,6521 | 9,1216 | | -3,1704 | | 0,010551 | | | 0,04132 |
| GRSF1 | Homo sapiens G-rich RNA sequence binding factor 1 (GRSF1), transcript variant 2, mRNA. | -0,6531 | 8,7459 | | -3,1978 | | 0,010082 | | | 0,04003 |
| LOC402112 | PREDICTED: Homo sapiens misc_RNA (LOC402112), miscRNA. | -0,6540 | 10,7279 | | -3,6872 | | 0,004532 | | | 0,02253 |
| MAGEA1 | Homo sapiens melanoma antigen family A, 1 (directs expression of antigen MZ2-E) (MAGEA1), mRNA. | -0,6543 | 8,4959 | | -4,9207 | | 0,000691 | | | 0,00650 |
| ZMPSTE24 | Homo sapiens zinc metallopeptidase (STE24 homolog, S. cerevisiae) (ZMPSTE24), mRNA. | -0,6546 | 10,0279 | | -3,4981 | | 0,006155 | | | 0,02816 |
| RNF20 | Homo sapiens ring finger protein 20 (RNF20), mRNA. | -0,6547 | 8,9248 | | -4,4476 | | 0,001386 | | | 0,01011 |
| DCXR | Homo sapiens dicarbonyl/L-xylulose reductase (DCXR), mRNA. | -0,6549 | 8,4672 | | -4,4740 | | 0,001333 | | | 0,00985 |
| H3F3A | Homo sapiens H3 histone, family 3A (H3F3A), mRNA. | -0,6550 | 12,3169 | | -5,3049 | | 0,000402 | | | 0,00478 |
| PIGS | Homo sapiens phosphatidylinositol glycan anchor biosynthesis, class S (PIGS), mRNA. | -0,6551 | 8,5473 | | -5,3998 | | 0,000353 | | | 0,00441 |
| MYLK | Homo sapiens myosin light chain kinase (MYLK), transcript variant 8, mRNA. | -0,6555 | 7,6895 | | -5,2479 | | 0,000435 | | | 0,00499 |
| BRCA1 | Homo sapiens breast cancer 1, early onset (BRCA1), transcript variant BRCA1-delta11b, mRNA. | -0,6557 | 7,6359 | | -5,9962 | | 0,000160 | | | 0,00289 |
| TP53 | Homo sapiens tumor protein p53 (TP53), mRNA. | -0,6563 | 7,8013 | | -3,2062 | | 0,009943 | | | 0,03963 |
| GPAA1 | Homo sapiens glycosylphosphatidylinositol anchor attachment protein 1 homolog (yeast) (GPAA1), mRNA. | -0,6565 | 9,6888 | | -4,3411 | | 0,001629 | | | 0,01120 |
| NAP1L4 | Homo sapiens nucleosome assembly protein 1-like 4 (NAP1L4), mRNA. | -0,6568 | 10,4843 | | -5,3908 | | 0,000357 | | | 0,00443 |
| TOR3A | Homo sapiens torsin family 3, member A (TOR3A), mRNA. | -0,6575 | 7,8108 | | -6,0273 | | 0,000154 | | | 0,00281 |
| LRPPRC | Homo sapiens leucine-rich PPR-motif containing (LRPPRC), mRNA. | -0,6578 | 8,2962 | | -3,1360 | | 0,011171 | | | 0,04313 |
| TOMM20 | Homo sapiens translocase of outer mitochondrial membrane 20 homolog (yeast) (TOMM20), nuclear gene encoding mitochondrial protein, mRNA. | -0,6579 | 10,9936 | | -3,2303 | | 0,009554 | | | 0,03843 |
| ISOC2 | Homo sapiens isochorismatase domain containing 2 (ISOC2), mRNA. | -0,6579 | 9,1027 | | -4,1174 | | 0,002297 | | | 0,01411 |
| MRPL22 | Homo sapiens mitochondrial ribosomal protein L22 (MRPL22), nuclear gene encoding mitochondrial protein, transcript variant 2, mRNA. | -0,6580 | 10,0334 | | -6,6125 | | 0,000074 | | | 0,00192 |
| NDUFB8 | Homo sapiens NADH dehydrogenase (ubiquinone) 1 beta subcomplex, 8, 19kDa (NDUFB8), mRNA. | -0,6584 | 11,2193 | | -3,2435 | | 0,009347 | | | 0,03785 |
| ADSL | Homo sapiens adenylosuccinate lyase (ADSL), mRNA. | -0,6590 | 10,3303 | | -3,1055 | | 0,011751 | | | 0,04483 |
| PSMD10 | Homo sapiens proteasome (prosome, macropain) 26S subunit, non-ATPase, 10 (PSMD10), transcript variant 1, mRNA. | -0,6597 | 9,1138 | | -5,5083 | | 0,000304 | | | 0,00410 |
| PARL | Homo sapiens presenilin associated, rhomboid-like (PARL), nuclear gene encoding mitochondrial protein, transcript variant 1, mRNA. | -0,6597 | 10,6994 | | -8,8106 | | 0,000007 | | | 0,00067 |
| LOC728620 | PREDICTED: Homo sapiens misc_RNA (LOC728620), miscRNA. | -0,6597 | 9,5302 | | -6,8206 | | 0,000058 | | | 0,00173 |
| C7orf36 | Homo sapiens chromosome 7 open reading frame 36 (C7orf36), mRNA. | -0,6601 | 8,2674 | | -5,5707 | | 0,000280 | | | 0,00392 |
| HSPD1 | Homo sapiens heat shock 60kDa protein 1 (chaperonin) (HSPD1), nuclear gene encoding mitochondrial protein, transcript variant 1, mRNA. | -0,6602 | 7,9237 | | -6,1343 | | 0,000134 | | | 0,00262 |
| LOC339804 | PREDICTED: Homo sapiens hypothetical gene supported by AK075484; BC014578 (LOC339804), mRNA. | -0,6605 | 8,4227 | | -4,7519 | | 0,000883 | | | 0,00763 |
| NDUFA11 | Homo sapiens NADH dehydrogenase (ubiquinone) 1 alpha subcomplex, 11, 14.7kDa (NDUFA11), nuclear gene encoding mitochondrial protein, mRNA. | -0,6605 | 10,4886 | | -4,0147 | | 0,002696 | | | 0,01580 |
| MRPL51 | Homo sapiens mitochondrial ribosomal protein L51 (MRPL51), nuclear gene encoding mitochondrial protein, mRNA. | -0,6608 | 11,3300 | | -7,4462 | | 0,000028 | | | 0,00124 |
| SLC25A3 | Homo sapiens solute carrier family 25 (mitochondrial carrier; phosphate carrier), member 3 (SLC25A3), nuclear gene encoding mitochondrial protein, transcript variant 2, mRNA. | -0,6613 | 12,1622 | | -6,4244 | | 0,000093 | | | 0,00216 |
| PTGFRN | Homo sapiens prostaglandin F2 receptor negative regulator (PTGFRN), mRNA. | -0,6613 | 9,1363 | | -3,3399 | | 0,007974 | | | 0,03390 |
| BSCL2 | Homo sapiens Bernardinelli-Seip congenital lipodystrophy 2 (seipin) (BSCL2), mRNA. | -0,6614 | 8,0952 | | -5,9080 | | 0,000179 | | | 0,00307 |
| C2orf64 | Homo sapiens chromosome 2 open reading frame 64 (C2orf64), mRNA. | -0,6615 | 8,0607 | | -6,1916 | | 0,000125 | | | 0,00249 |
| RBM17 | Homo sapiens RNA binding motif protein 17 (RBM17), mRNA. | -0,6617 | 8,2529 | | -3,7162 | | 0,004326 | | | 0,02177 |
| TUBG1 | Homo sapiens tubulin, gamma 1 (TUBG1), mRNA. | -0,6621 | 8,8552 | | -4,4049 | | 0,001479 | | | 0,01055 |
| PGM2 | Homo sapiens phosphoglucomutase 2 (PGM2), mRNA. | -0,6623 | 8,1321 | | -3,1965 | | 0,010104 | | | 0,04009 |
| CETN3 | Homo sapiens centrin, EF-hand protein, 3 (CDC31 homolog, yeast) (CETN3), mRNA. | -0,6628 | 8,7381 | | -4,0377 | | 0,002600 | | | 0,01543 |
| RPL15 | Homo sapiens ribosomal protein L15 (RPL15), mRNA. | -0,6632 | 10,8034 | | -4,1849 | | 0,002069 | | | 0,01316 |
| PGRMC1 | Homo sapiens progesterone receptor membrane component 1 (PGRMC1), mRNA. | -0,6632 | 10,6682 | | -7,4565 | | 0,000028 | | | 0,00123 |
| SLC25A15 | Homo sapiens solute carrier family 25 (mitochondrial carrier; ornithine transporter) member 15 (SLC25A15), nuclear gene encoding mitochondrial protein, mRNA. | -0,6633 | 8,0826 | | -4,7265 | | 0,000916 | | | 0,00783 |
| MGC3731 | Homo sapiens hypothetical protein MGC3731 (MGC3731), mRNA. | -0,6636 | 8,6919 | | -3,2896 | | 0,008663 | | | 0,03593 |
| JUB | Homo sapiens jub, ajuba homolog (Xenopus laevis) (JUB), transcript variant 1, mRNA. | -0,6638 | 8,3395 | | -4,1013 | | 0,002355 | | | 0,01438 |
| SARS2 | Homo sapiens seryl-tRNA synthetase 2, mitochondrial (SARS2), nuclear gene encoding mitochondrial protein, mRNA. | -0,6639 | 8,7621 | | -4,6400 | | 0,001041 | | | 0,00842 |
| ATP1B3 | Homo sapiens ATPase, Na+/K+ transporting, beta 3 polypeptide (ATP1B3), mRNA. XM_945518 | -0,6645 | 9,2820 | | -4,4229 | | 0,001439 | | | 0,01036 |
| ZNF362 | Homo sapiens zinc finger protein 362 (ZNF362), mRNA. | -0,6648 | 7,6196 | | -7,6570 | | 0,000022 | | | 0,00113 |
| C12orf52 | Homo sapiens chromosome 12 open reading frame 52 (C12orf52), mRNA. | -0,6654 | 8,2271 | | -7,8419 | | 0,000018 | | | 0,00103 |
| NAV2 | Homo sapiens neuron navigator 2 (NAV2), transcript variant 2, mRNA. | -0,6657 | 7,9643 | | -3,0326 | | 0,013270 | | | 0,04919 |
| UROD | Homo sapiens uroporphyrinogen decarboxylase (UROD), mRNA. | -0,6661 | 9,8820 | | -4,3288 | | 0,001660 | | | 0,01135 |
| MRPL22 | Homo sapiens mitochondrial ribosomal protein L22 (MRPL22), nuclear gene encoding mitochondrial protein, transcript variant 1, mRNA. | -0,6673 | 9,4050 | | -5,4431 | | 0,000333 | | | 0,00430 |
| TTC5 | Homo sapiens tetratricopeptide repeat domain 5 (TTC5), mRNA. | -0,6674 | 7,9599 | | -5,0995 | | 0,000536 | | | 0,00562 |
| DDX55 | Homo sapiens DEAD (Asp-Glu-Ala-Asp) box polypeptide 55 (DDX55), mRNA. | -0,6681 | 9,3716 | | -3,4020 | | 0,007201 | | | 0,03158 |
| USP13 | Homo sapiens ubiquitin specific peptidase 13 (isopeptidase T-3) (USP13), mRNA. | -0,6683 | 7,9610 | | -5,7999 | | 0,000206 | | | 0,00332 |
| HELLS | Homo sapiens helicase, lymphoid-specific (HELLS), mRNA. | -0,6691 | 7,7185 | | -6,6923 | | 0,000067 | | | 0,00184 |
| RPL7L1 | Homo sapiens ribosomal protein L7-like 1 (RPL7L1), mRNA. | -0,6691 | 9,8947 | | -5,1969 | | 0,000467 | | | 0,00522 |
| MRPL35 | Homo sapiens mitochondrial ribosomal protein L35 (MRPL35), nuclear gene encoding mitochondrial protein, transcript variant 1, mRNA. | -0,6700 | 8,0782 | | -8,6314 | | 0,000008 | | | 0,00073 |
| CHAF1B | Homo sapiens chromatin assembly factor 1, subunit B (p60) (CHAF1B), mRNA. | -0,6703 | 7,8220 | | -7,6125 | | 0,000024 | | | 0,00115 |
| NUCKS1 | Homo sapiens nuclear casein kinase and cyclin-dependent kinase substrate 1 (NUCKS1), mRNA. | -0,6704 | 11,7277 | | -3,0991 | | 0,011879 | | | 0,04518 |
| THOP1 | Homo sapiens thimet oligopeptidase 1 (THOP1), mRNA. | -0,6705 | 8,5903 | | -6,2347 | | 0,000118 | | | 0,00243 |
| WDR5 | Homo sapiens WD repeat domain 5 (WDR5), transcript variant 1, mRNA. | -0,6708 | 8,6449 | | -5,7962 | | 0,000207 | | | 0,00333 |
| DCAF7 | Homo sapiens DDB1 and CUL4 associated factor 7 (DCAF7), mRNA. | -0,6711 | 9,9188 | | -3,4954 | | 0,006182 | | | 0,02824 |
| BLMH | Homo sapiens bleomycin hydrolase (BLMH), mRNA. | -0,6726 | 8,2308 | | -6,0236 | | 0,000154 | | | 0,00282 |
| LOC100134304 | PREDICTED: Homo sapiens similar to hCG1983233 (LOC100134304), mRNA. | -0,6727 | 8,0077 | | -4,0483 | | 0,002558 | | | 0,01527 |
| GMDS | Homo sapiens GDP-mannose 4,6-dehydratase (GMDS), mRNA. | -0,6729 | 9,0444 | | -3,5244 | | 0,005897 | | | 0,02730 |
| SRP14 | Homo sapiens signal recognition particle 14kDa (homologous Alu RNA binding protein) (SRP14), mRNA. | -0,6730 | 9,9241 | | -4,0272 | | 0,002644 | | | 0,01558 |
| GTF2IP1 | Homo sapiens general transcription factor II, i, pseudogene 1 (GTF2IP1) on chromosome 7. | -0,6735 | 8,9047 | | -4,2944 | | 0,001749 | | | 0,01178 |
| AGPAT5 | Homo sapiens 1-acylglycerol-3-phosphate O-acyltransferase 5 (lysophosphatidic acid acyltransferase, epsilon) (AGPAT5), mRNA. | -0,6736 | 8,1233 | | -6,4341 | | 0,000092 | | | 0,00215 |
| RPL26L1 | Homo sapiens ribosomal protein L26-like 1 (RPL26L1), mRNA. | -0,6736 | 10,3643 | | -3,7554 | | 0,004063 | | | 0,02085 |
| BCL11A | Homo sapiens B-cell CLL/lymphoma 11A (zinc finger protein) (BCL11A), transcript variant 1, mRNA. | -0,6737 | 7,5871 | | -7,4679 | | 0,000028 | | | 0,00122 |
| AP2M1 | Homo sapiens adaptor-related protein complex 2, mu 1 subunit (AP2M1), transcript variant 1, mRNA. | -0,6737 | 11,1612 | | -3,6819 | | 0,004572 | | | 0,02266 |
| FAT2 | Homo sapiens FAT tumor suppressor homolog 2 (Drosophila) (FAT2), mRNA. | -0,6741 | 7,6212 | | -4,7787 | | 0,000849 | | | 0,00744 |
| CWF19L1 | Homo sapiens CWF19-like 1, cell cycle control (S. pombe) (CWF19L1), mRNA. | -0,6743 | 9,5300 | | -6,2543 | | 0,000115 | | | 0,00241 |
| MRPL30 | Homo sapiens mitochondrial ribosomal protein L30 (MRPL30), nuclear gene encoding mitochondrial protein, transcript variant 1, mRNA. | -0,6743 | 8,6411 | | -6,3295 | | 0,000105 | | | 0,00227 |
| TSEN34 | Homo sapiens tRNA splicing endonuclease 34 homolog (S. cerevisiae) (TSEN34), transcript variant 2, mRNA. | -0,6743 | 10,5840 | | -6,8823 | | 0,000054 | | | 0,00168 |
| AP2M1 | Homo sapiens adaptor-related protein complex 2, mu 1 subunit (AP2M1), transcript variant 1, mRNA. | -0,6744 | 10,9737 | | -7,4130 | | 0,000029 | | | 0,00125 |
| MNS1 | Homo sapiens meiosis-specific nuclear structural 1 (MNS1), mRNA. | -0,6745 | 7,8553 | | -3,4822 | | 0,006316 | | | 0,02865 |
| SRP9 | Homo sapiens signal recognition particle 9kDa (SRP9), mRNA. | -0,6747 | 8,8122 | | -7,1132 | | 0,000041 | | | 0,00146 |
| C8orf38 | Homo sapiens chromosome 8 open reading frame 38 (C8orf38), mRNA. | -0,6754 | 8,0163 | | -5,6571 | | 0,000249 | | | 0,00368 |
| BMP7 | Homo sapiens bone morphogenetic protein 7 (osteogenic protein 1) (BMP7), mRNA. | -0,6757 | 7,9229 | | -4,9830 | | 0,000632 | | | 0,00617 |
| BRI3BP | Homo sapiens BRI3 binding protein (BRI3BP), mRNA. | -0,6758 | 8,0787 | | -5,9123 | | 0,000178 | | | 0,00306 |
| MLH1 | Homo sapiens mutL homolog 1, colon cancer, nonpolyposis type 2 (E. coli) (MLH1), mRNA. | -0,6760 | 8,3771 | | -4,9963 | | 0,000620 | | | 0,00612 |
| ANXA8 | Homo sapiens annexin A8 (ANXA8), mRNA. XM_931361 XM_931369 XM_931374 XM_931375 XM_931378 XM_931383 XM_931388 XM_931391 XM_931399 XM_931404 XM_931411 | -0,6772 | 9,1803 | | -5,5297 | | 0,000296 | | | 0,00404 |
| SSBP1 | Homo sapiens single-stranded DNA binding protein 1 (SSBP1), mRNA. | -0,6778 | 10,7351 | | -4,5866 | | 0,001126 | | | 0,00884 |
| LOC374395 | Homo sapiens similar to RIKEN cDNA 1810059G22 (LOC374395), mRNA. | -0,6782 | 9,7133 | | -3,1408 | | 0,011081 | | | 0,04287 |
| CYB5B | Homo sapiens cytochrome b5 type B (outer mitochondrial membrane) (CYB5B), nuclear gene encoding mitochondrial protein, mRNA. | -0,6785 | 11,6741 | | -7,8792 | | 0,000018 | | | 0,00101 |
| SERBP1 | Homo sapiens SERPINE1 mRNA binding protein 1 (SERBP1), transcript variant 3, mRNA. | -0,6788 | 8,4737 | | -5,5817 | | 0,000276 | | | 0,00388 |
| GPR177 | Homo sapiens G protein-coupled receptor 177 (GPR177), transcript variant 2, mRNA. | -0,6798 | 8,2344 | | -6,9654 | | 0,000049 | | | 0,00159 |
| ZMAT2 | Homo sapiens zinc finger, matrin type 2 (ZMAT2), mRNA. | -0,6801 | 9,2121 | | -4,0390 | | 0,002595 | | | 0,01540 |
| MRPL37 | Homo sapiens mitochondrial ribosomal protein L37 (MRPL37), nuclear gene encoding mitochondrial protein, mRNA. | -0,6802 | 10,8803 | | -4,4419 | | 0,001398 | | | 0,01016 |
| SNRPA1 | Homo sapiens small nuclear ribonucleoprotein polypeptide A' (SNRPA1), mRNA. | -0,6804 | 10,8674 | | -6,2696 | | 0,000113 | | | 0,00238 |
| PSMB8 | Homo sapiens proteasome (prosome, macropain) subunit, beta type, 8 (large multifunctional peptidase 7) (PSMB8), transcript variant 2, mRNA. | -0,6805 | 8,0683 | | -6,3375 | | 0,000104 | | | 0,00227 |
| ZNF828 | Homo sapiens zinc finger protein 828 (ZNF828), mRNA. | -0,6809 | 8,5788 | | -6,6899 | | 0,000068 | | | 0,00184 |
| KLHL12 | Homo sapiens kelch-like 12 (Drosophila) (KLHL12), mRNA. | -0,6810 | 9,3406 | | -3,8677 | | 0,003398 | | | 0,01837 |
| ACAD9 | Homo sapiens acyl-Coenzyme A dehydrogenase family, member 9 (ACAD9), mRNA. | -0,6811 | 8,5445 | | -4,2209 | | 0,001958 | | | 0,01268 |
| FAM181B | Homo sapiens family with sequence similarity 181, member B (FAM181B), mRNA. | -0,6813 | 8,0753 | | -7,3407 | | 0,000032 | | | 0,00129 |
| SUOX | Homo sapiens sulfite oxidase (SUOX), nuclear gene encoding mitochondrial protein, transcript variant 1, mRNA. | -0,6820 | 7,8347 | | -9,5239 | | 0,000003 | | | 0,00051 |
| BMP4 | Homo sapiens bone morphogenetic protein 4 (BMP4), transcript variant 3, mRNA. | -0,6828 | 7,6179 | | -7,0679 | | 0,000043 | | | 0,00150 |
| NSMCE4A | Homo sapiens non-SMC element 4 homolog A (S. cerevisiae) (NSMCE4A), mRNA. | -0,6830 | 9,6393 | | -4,2815 | | 0,001784 | | | 0,01192 |
| TMEM126B | Homo sapiens transmembrane protein 126B (TMEM126B), mRNA. | -0,6831 | 9,3839 | | -4,2902 | | 0,001761 | | | 0,01183 |
| LOC100134189 | PREDICTED: Homo sapiens similar to LOC441089 protein, transcript variant 1 (LOC100134189), mRNA. | -0,6832 | 8,6160 | | -9,8103 | | 0,000003 | | | 0,00048 |
| TSR2 | Homo sapiens TSR2, 20S rRNA accumulation, homolog (S. cerevisiae) (TSR2), mRNA. | -0,6836 | 9,1625 | | -6,8912 | | 0,000053 | | | 0,00167 |
| CTSC | Homo sapiens cathepsin C (CTSC), transcript variant 1, mRNA. | -0,6837 | 9,3057 | | -3,1401 | | 0,011094 | | | 0,04290 |
| POLD1 | Homo sapiens polymerase (DNA directed), delta 1, catalytic subunit 125kDa (POLD1), mRNA. | -0,6839 | 8,0465 | | -3,8445 | | 0,003525 | | | 0,01885 |
| PIGK | Homo sapiens phosphatidylinositol glycan anchor biosynthesis, class K (PIGK), mRNA. | -0,6843 | 8,7523 | | -3,8536 | | 0,003475 | | | 0,01867 |
| COMMD9 | Homo sapiens COMM domain containing 9 (COMMD9), mRNA. | -0,6846 | 8,5318 | | -6,2997 | | 0,000109 | | | 0,00232 |
| SULF2 | Homo sapiens sulfatase 2 (SULF2), transcript variant 1, mRNA. | -0,6848 | 8,4007 | | -3,7857 | | 0,003871 | | | 0,02011 |
| BUB3 | Homo sapiens BUB3 budding uninhibited by benzimidazoles 3 homolog (yeast) (BUB3), transcript variant 1, mRNA. | -0,6855 | 11,3929 | | -3,7111 | | 0,004362 | | | 0,02190 |
| ECHS1 | Homo sapiens enoyl Coenzyme A hydratase, short chain, 1, mitochondrial (ECHS1), nuclear gene encoding mitochondrial protein, mRNA. | -0,6860 | 10,2970 | | -3,6529 | | 0,004790 | | | 0,02338 |
| ZNF618 | Homo sapiens zinc finger protein 618 (ZNF618), mRNA. | -0,6870 | 7,7087 | | -11,3177 | | 0,000001 | | | 0,00029 |
| RPL29 | Homo sapiens ribosomal protein L29 (RPL29), mRNA. | -0,6871 | 9,5130 | | -4,6837 | | 0,000976 | | | 0,00813 |
| ILF3 | Homo sapiens interleukin enhancer binding factor 3, 90kDa (ILF3), transcript variant 2, mRNA. | -0,6876 | 8,8037 | | -5,5423 | | 0,000291 | | | 0,00400 |
| IDH3B | Homo sapiens isocitrate dehydrogenase 3 (NAD+) beta (IDH3B), nuclear gene encoding mitochondrial protein, transcript variant 1, mRNA. | -0,6884 | 9,9703 | | -4,5881 | | 0,001124 | | | 0,00883 |
| EID2 | Homo sapiens EP300 interacting inhibitor of differentiation 2 (EID2), mRNA. | -0,6885 | 8,4680 | | -4,4979 | | 0,001286 | | | 0,00961 |
| MRPS18B | Homo sapiens mitochondrial ribosomal protein S18B (MRPS18B), nuclear gene encoding mitochondrial protein, mRNA. | -0,6887 | 9,8226 | | -6,2502 | | 0,000116 | | | 0,00241 |
| MRPL50 | Homo sapiens mitochondrial ribosomal protein L50 (MRPL50), nuclear gene encoding mitochondrial protein, mRNA. | -0,6894 | 9,9336 | | -5,1927 | | 0,000470 | | | 0,00523 |
| MED20 | Homo sapiens mediator complex subunit 20 (MED20), mRNA. | -0,6896 | 9,1308 | | -9,4040 | | 0,000004 | | | 0,00052 |
| TTC27 | Homo sapiens tetratricopeptide repeat domain 27 (TTC27), mRNA. | -0,6904 | 8,5600 | | -3,1180 | | 0,011509 | | | 0,04410 |
| RPL26L1 | Homo sapiens ribosomal protein L26-like 1 (RPL26L1), mRNA. | -0,6910 | 9,4505 | | -3,9760 | | 0,002864 | | | 0,01641 |
| FAM53B | Homo sapiens family with sequence similarity 53, member B (FAM53B), mRNA. | -0,6912 | 7,9133 | | -6,0384 | | 0,000151 | | | 0,00280 |
| MPV17 | Homo sapiens MpV17 mitochondrial inner membrane protein (MPV17), nuclear gene encoding mitochondrial protein, mRNA. | -0,6916 | 9,0462 | | -3,5812 | | 0,005378 | | | 0,02543 |
| MRPS21 | Homo sapiens mitochondrial ribosomal protein S21 (MRPS21), nuclear gene encoding mitochondrial protein, transcript variant 2, mRNA. | -0,6920 | 10,5812 | | -3,7138 | | 0,004343 | | | 0,02183 |
| U2AF2 | Homo sapiens U2 small nuclear RNA auxiliary factor 2 (U2AF2), transcript variant 1, mRNA. | -0,6923 | 10,7106 | | -6,0608 | | 0,000147 | | | 0,00276 |
| LOC441089 | Homo sapiens CRSP8 pseudogene (LOC441089), non-coding RNA. | -0,6925 | 9,6233 | | -14,3604 | | 0,000000 | | | 0,00012 |
| NUP155 | Homo sapiens nucleoporin 155kDa (NUP155), transcript variant 1, mRNA. | -0,6934 | 8,7416 | | -4,3570 | | 0,001590 | | | 0,01105 |
| PDHB | Homo sapiens pyruvate dehydrogenase (lipoamide) beta (PDHB), mRNA. | -0,6935 | 10,2666 | | -6,7632 | | 0,000062 | | | 0,00178 |
| RAB7L1 | Homo sapiens RAB7, member RAS oncogene family-like 1 (RAB7L1), mRNA. | -0,6935 | 8,2946 | | -6,9265 | | 0,000051 | | | 0,00163 |
| G6PC3 | Homo sapiens glucose 6 phosphatase, catalytic, 3 (G6PC3), mRNA. | -0,6937 | 8,6511 | | -4,2004 | | 0,002020 | | | 0,01294 |
| MRPS11 | Homo sapiens mitochondrial ribosomal protein S11 (MRPS11), nuclear gene encoding mitochondrial protein, transcript variant 2, mRNA. | -0,6937 | 8,8830 | | -5,3355 | | 0,000385 | | | 0,00464 |
| TRIM28 | Homo sapiens tripartite motif-containing 28 (TRIM28), mRNA. | -0,6938 | 9,5190 | | -5,5282 | | 0,000296 | | | 0,00405 |
| SEPHS1 | Homo sapiens selenophosphate synthetase 1 (SEPHS1), mRNA. | -0,6941 | 8,0819 | | -6,2507 | | 0,000116 | | | 0,00241 |
| ZW10 | Homo sapiens ZW10, kinetochore associated, homolog (Drosophila) (ZW10), mRNA. | -0,6942 | 9,3159 | | -8,3699 | | 0,000011 | | | 0,00083 |
| MTP18 | Homo sapiens mitochondrial protein 18 kDa (MTP18), nuclear gene encoding mitochondrial protein, transcript variant 1, mRNA. | -0,6948 | 8,8044 | | -7,3449 | | 0,000032 | | | 0,00129 |
| BCL11A | Homo sapiens B-cell CLL/lymphoma 11A (zinc finger protein) (BCL11A), transcript variant 1, mRNA. | -0,6949 | 7,6529 | | -6,5878 | | 0,000076 | | | 0,00196 |
| TMEM4 | Homo sapiens transmembrane protein 4 (TMEM4), mRNA. | -0,6951 | 8,3944 | | -11,1778 | | 0,000001 | | | 0,00031 |
| LOC653147 | Homo sapiens hCG1995786 (LOC653147), mRNA. | -0,6953 | 10,4825 | | -4,7113 | | 0,000937 | | | 0,00796 |
| SLC35A4 | Homo sapiens solute carrier family 35, member A4 (SLC35A4), mRNA. | -0,6955 | 8,8640 | | -6,8615 | | 0,000055 | | | 0,00169 |
| KCNMB4 | Homo sapiens potassium large conductance calcium-activated channel, subfamily M, beta member 4 (KCNMB4), mRNA. | -0,6956 | 7,6313 | | -6,2232 | | 0,000120 | | | 0,00245 |
| AMZ2 | Homo sapiens archaelysin family metallopeptidase 2 (AMZ2), transcript variant 2, mRNA. | -0,6958 | 10,5474 | | -4,0272 | | 0,002644 | | | 0,01558 |
| LOC643287 | PREDICTED: Homo sapiens similar to prothymosin alpha, transcript variant 1 (LOC643287), mRNA. | -0,6963 | 8,6964 | | -3,4133 | | 0,007069 | | | 0,03118 |
| MRPL24 | Homo sapiens mitochondrial ribosomal protein L24 (MRPL24), nuclear gene encoding mitochondrial protein, transcript variant 2, mRNA. | -0,6964 | 8,9851 | | -4,5073 | | 0,001268 | | | 0,00951 |
| LOC653226 | PREDICTED: Homo sapiens similar to Signal recognition particle 9 kDa protein (SRP9) (LOC653226), mRNA. | -0,6967 | 11,7242 | | -3,4042 | | 0,007175 | | | 0,03151 |
| UTP14A | Homo sapiens UTP14, U3 small nucleolar ribonucleoprotein, homolog A (yeast) (UTP14A), mRNA. | -0,6975 | 9,1059 | | -5,0409 | | 0,000582 | | | 0,00590 |
| GPI | Homo sapiens glucose phosphate isomerase (GPI), mRNA. | -0,6977 | 10,4907 | | -3,6837 | | 0,004559 | | | 0,02261 |
| RBM23 | Homo sapiens RNA binding motif protein 23 (RBM23), transcript variant 2, mRNA. | -0,6981 | 9,5547 | | -4,5319 | | 0,001222 | | | 0,00928 |
| LOC653884 | PREDICTED: Homo sapiens similar to FUS interacting protein (serine-arginine rich) 1 (LOC653884), mRNA. | -0,6984 | 8,9323 | | -5,6093 | | 0,000266 | | | 0,00383 |
| PSRC1 | Homo sapiens proline/serine-rich coiled-coil 1 (PSRC1), transcript variant 3, mRNA. | -0,6985 | 7,8372 | | -6,7451 | | 0,000063 | | | 0,00180 |
| BCHE | Homo sapiens butyrylcholinesterase (BCHE), mRNA. | -0,6986 | 7,6889 | | -5,4522 | | 0,000329 | | | 0,00428 |
| CENPM | Homo sapiens centromere protein M (CENPM), transcript variant 2, mRNA. | -0,6992 | 7,6513 | | -6,5550 | | 0,000080 | | | 0,00200 |
| AK3L1 | Homo sapiens adenylate kinase 3-like 1 (AK3L1), nuclear gene encoding mitochondrial protein, transcript variant 6, mRNA. | -0,6995 | 9,0119 | | -3,4783 | | 0,006357 | | | 0,02879 |
| SMAP2 | Homo sapiens small ArfGAP2 (SMAP2), mRNA. | -0,6995 | 8,6731 | | -5,4548 | | 0,000327 | | | 0,00427 |
| MFN2 | Homo sapiens mitofusin 2 (MFN2), nuclear gene encoding mitochondrial protein, mRNA. | -0,6997 | 9,0216 | | -6,4113 | | 0,000095 | | | 0,00216 |
| TUBG1 | Homo sapiens tubulin, gamma 1 (TUBG1), mRNA. | -0,7001 | 9,9790 | | -4,4784 | | 0,001324 | | | 0,00982 |
| ENSA | Homo sapiens endosulfine alpha (ENSA), transcript variant 2, mRNA. | -0,7003 | 8,5515 | | -3,5037 | | 0,006099 | | | 0,02798 |
| CYCSL1 | Homo sapiens cytochrome c, somatic-like 1 (CYCSL1) on chromosome 6. | -0,7008 | 10,8524 | | -4,1210 | | 0,002284 | | | 0,01405 |
| S1PR5 | Homo sapiens sphingosine-1-phosphate receptor 5 (S1PR5), mRNA. | -0,7009 | 7,5671 | | -5,8918 | | 0,000183 | | | 0,00310 |
| MED27 | Homo sapiens mediator complex subunit 27 (MED27), mRNA. | -0,7010 | 8,8086 | | -7,6983 | | 0,000021 | | | 0,00111 |
| LOC653377 | PREDICTED: Homo sapiens similar to family with sequence similarity 36, member A (LOC653377), mRNA. | -0,7020 | 8,6801 | | -3,4474 | | 0,006685 | | | 0,02991 |
| FANCI | Homo sapiens Fanconi anemia, complementation group I (FANCI), transcript variant 2, mRNA. | -0,7024 | 7,8261 | | -4,8679 | | 0,000746 | | | 0,00683 |
| TUBA1B | Homo sapiens tubulin, alpha 1b (TUBA1B), mRNA. | -0,7030 | 13,1163 | | -5,5516 | | 0,000287 | | | 0,00398 |
| ZMYM3 | Homo sapiens zinc finger, MYM-type 3 (ZMYM3), transcript variant 2, mRNA. | -0,7031 | 7,8550 | | -7,0118 | | 0,000046 | | | 0,00154 |
| PDSS1 | Homo sapiens prenyl (decaprenyl) diphosphate synthase, subunit 1 (PDSS1), mRNA. | -0,7034 | 7,8725 | | -5,3621 | | 0,000372 | | | 0,00453 |
| PGAM5 | PREDICTED: Homo sapiens phosphoglycerate mutase family member 5 (PGAM5), mRNA. | -0,7039 | 8,4583 | | -6,4500 | | 0,000090 | | | 0,00214 |
| CBX2 | Homo sapiens chromobox homolog 2 (Pc class homolog, Drosophila) (CBX2), transcript variant 1, mRNA. | -0,7039 | 7,9240 | | -6,6416 | | 0,000072 | | | 0,00190 |
| PSMC5 | Homo sapiens proteasome (prosome, macropain) 26S subunit, ATPase, 5 (PSMC5), mRNA. | -0,7041 | 10,9766 | | -6,4372 | | 0,000092 | | | 0,00215 |
| MRPS12 | Homo sapiens mitochondrial ribosomal protein S12 (MRPS12), nuclear gene encoding mitochondrial protein, transcript variant 3, mRNA. | -0,7045 | 11,1266 | | -5,0388 | | 0,000584 | | | 0,00591 |
| SEPX1 | Homo sapiens selenoprotein X, 1 (SEPX1), mRNA. | -0,7060 | 9,2387 | | -4,9762 | | 0,000638 | | | 0,00620 |
| COPS3 | Homo sapiens COP9 constitutive photomorphogenic homolog subunit 3 (Arabidopsis) (COPS3), mRNA. | -0,7061 | 10,2765 | | -5,4198 | | 0,000343 | | | 0,00435 |
| SIVA | Homo sapiens CD27-binding (Siva) protein (SIVA), transcript variant 2, mRNA. | -0,7061 | 9,2195 | | -3,1851 | | 0,010297 | | | 0,04058 |
| G3BP1 | Homo sapiens GTPase activating protein (SH3 domain) binding protein 1 (G3BP1), transcript variant 1, mRNA. | -0,7062 | 8,8392 | | -4,0099 | | 0,002716 | | | 0,01586 |
| MED27 | Homo sapiens mediator complex subunit 27 (MED27), mRNA. | -0,7063 | 8,8988 | | -9,3185 | | 0,000004 | | | 0,00054 |
| C3orf75 | Homo sapiens chromosome 3 open reading frame 75 (C3orf75), mRNA. | -0,7067 | 8,0995 | | -7,0387 | | 0,000045 | | | 0,00152 |
| LOC647000 | PREDICTED: Homo sapiens similar to tubulin, beta 5 (LOC647000), mRNA. | -0,7073 | 13,0163 | | -4,4387 | | 0,001405 | | | 0,01020 |
| EBAG9 | Homo sapiens estrogen receptor binding site associated, antigen, 9 (EBAG9), transcript variant 2, mRNA. | -0,7074 | 9,6884 | | -3,5418 | | 0,005733 | | | 0,02672 |
| ABCB9 | Homo sapiens ATP-binding cassette, sub-family B (MDR/TAP), member 9 (ABCB9), transcript variant 2, mRNA. | -0,7075 | 7,8742 | | -6,5706 | | 0,000078 | | | 0,00198 |
| DLD | Homo sapiens dihydrolipoamide dehydrogenase (DLD), mRNA. | -0,7078 | 8,6507 | | -7,8119 | | 0,000019 | | | 0,00104 |
| C19orf62 | Homo sapiens chromosome 19 open reading frame 62 (C19orf62), transcript variant 2, mRNA. | -0,7085 | 9,8996 | | -4,9662 | | 0,000647 | | | 0,00625 |
| C3orf75 | Homo sapiens chromosome 3 open reading frame 75 (C3orf75), mRNA. | -0,7085 | 8,2565 | | -6,9157 | | 0,000052 | | | 0,00165 |
| EIF5 | Homo sapiens eukaryotic translation initiation factor 5 (EIF5), transcript variant 1, mRNA. | -0,7086 | 8,7163 | | -4,4259 | | 0,001433 | | | 0,01032 |
| HSPA8 | Homo sapiens heat shock 70kDa protein 8 (HSPA8), transcript variant 2, mRNA. | -0,7098 | 12,3784 | | -3,4018 | | 0,007203 | | | 0,03158 |
| DPH5 | Homo sapiens DPH5 homolog (S. cerevisiae) (DPH5), transcript variant 3, mRNA. | -0,7103 | 8,5587 | | -4,8047 | | 0,000817 | | | 0,00725 |
| TOMM20 | Homo sapiens translocase of outer mitochondrial membrane 20 homolog (yeast) (TOMM20), nuclear gene encoding mitochondrial protein, mRNA. | -0,7104 | 10,2775 | | -3,8517 | | 0,003485 | | | 0,01871 |
| CDK2 | Homo sapiens cyclin-dependent kinase 2 (CDK2), transcript variant 1, mRNA. | -0,7114 | 9,1106 | | -5,4319 | | 0,000338 | | | 0,00433 |
| AFAP1L2 | Homo sapiens actin filament associated protein 1-like 2 (AFAP1L2), transcript variant 1, mRNA. | -0,7127 | 9,5037 | | -4,3512 | | 0,001604 | | | 0,01110 |
| DCTN3 | Homo sapiens dynactin 3 (p22) (DCTN3), transcript variant 1, mRNA. | -0,7135 | 9,3747 | | -4,8530 | | 0,000762 | | | 0,00694 |
| TMEM126B | Homo sapiens transmembrane protein 126B (TMEM126B), mRNA. | -0,7150 | 9,6741 | | -3,7238 | | 0,004274 | | | 0,02160 |
| IRS1 | Homo sapiens insulin receptor substrate 1 (IRS1), mRNA. | -0,7154 | 8,5028 | | -4,9462 | | 0,000666 | | | 0,00634 |
| ATPAF1 | Homo sapiens ATP synthase mitochondrial F1 complex assembly factor 1 (ATPAF1), nuclear gene encoding mitochondrial protein, transcript variant 1, mRNA. | -0,7157 | 8,3731 | | -5,3771 | | 0,000364 | | | 0,00448 |
| PREI3 | Homo sapiens preimplantation protein 3 (PREI3), transcript variant 2, mRNA. | -0,7157 | 8,7737 | | -6,5414 | | 0,000081 | | | 0,00201 |
| PSMA1 | Homo sapiens proteasome (prosome, macropain) subunit, alpha type, 1 (PSMA1), transcript variant 1, mRNA. | -0,7160 | 11,0033 | | -3,9808 | | 0,002843 | | | 0,01633 |
| MRPS17 | Homo sapiens mitochondrial ribosomal protein S17 (MRPS17), nuclear gene encoding mitochondrial protein, mRNA. | -0,7168 | 10,9961 | | -4,2678 | | 0,001822 | | | 0,01206 |
| LOC642946 | PREDICTED: Homo sapiens hypothetical protein LOC642945, transcript variant 2 (LOC642946), mRNA. | -0,7173 | 7,8700 | | -5,7906 | | 0,000209 | | | 0,00333 |
| SAC3D1 | Homo sapiens SAC3 domain containing 1 (SAC3D1), mRNA. | -0,7174 | 9,2643 | | -5,9402 | | 0,000172 | | | 0,00301 |
| PIGW | Homo sapiens phosphatidylinositol glycan anchor biosynthesis, class W (PIGW), mRNA. | -0,7185 | 8,2245 | | -5,6551 | | 0,000250 | | | 0,00369 |
| FIGNL1 | Homo sapiens fidgetin-like 1 (FIGNL1), transcript variant 1, mRNA. | -0,7189 | 7,6606 | | -10,1515 | | 0,000002 | | | 0,00042 |
| C1GALT1C1 | Homo sapiens C1GALT1-specific chaperone 1 (C1GALT1C1), transcript variant 1, mRNA. | -0,7193 | 9,2019 | | -6,8796 | | 0,000054 | | | 0,00168 |
| PEBP1 | Homo sapiens phosphatidylethanolamine binding protein 1 (PEBP1), mRNA. | -0,7193 | 11,1856 | | -3,8274 | | 0,003623 | | | 0,01921 |
| LOC729082 | PREDICTED: Homo sapiens misc_RNA (LOC729082), miscRNA. | -0,7197 | 8,7195 | | -3,8939 | | 0,003260 | | | 0,01787 |
| RP11-529I10.4 | Homo sapiens deleted in a mouse model of primary ciliary dyskinesia (RP11-529I10.4), mRNA. | -0,7198 | 8,3808 | | -5,3495 | | 0,000378 | | | 0,00458 |
| PHLDB1 | Homo sapiens pleckstrin homology-like domain, family B, member 1 (PHLDB1), mRNA. | -0,7199 | 8,0179 | | -7,0257 | | 0,000045 | | | 0,00153 |
| CEP192 | Homo sapiens centrosomal protein 192kDa (CEP192), mRNA. | -0,7202 | 8,3624 | | -7,0655 | | 0,000043 | | | 0,00150 |
| ACAT1 | Homo sapiens acetyl-Coenzyme A acetyltransferase 1 (ACAT1), nuclear gene encoding mitochondrial protein, mRNA. | -0,7205 | 10,0307 | | -3,3285 | | 0,008125 | | | 0,03434 |
| PRMT3 | Homo sapiens protein arginine methyltransferase 3 (PRMT3), mRNA. | -0,7208 | 8,7868 | | -3,9288 | | 0,003085 | | | 0,01719 |
| ATP5C1 | Homo sapiens ATP synthase, H+ transporting, mitochondrial F1 complex, gamma polypeptide 1 (ATP5C1), nuclear gene encoding mitochondrial protein, transcript variant 2, mRNA. | -0,7209 | 10,1261 | | -3,6667 | | 0,004685 | | | 0,02302 |
| MLLT11 | Homo sapiens myeloid/lymphoid or mixed-lineage leukemia (trithorax homolog, Drosophila); translocated to, 11 (MLLT11), mRNA. | -0,7216 | 10,2765 | | -4,0966 | | 0,002372 | | | 0,01447 |
| CCDC5 | Homo sapiens coiled-coil domain containing 5 (spindle associated) (CCDC5), mRNA. | -0,7222 | 8,4325 | | -5,7958 | | 0,000207 | | | 0,00333 |
| MRPS27 | Homo sapiens mitochondrial ribosomal protein S27 (MRPS27), nuclear gene encoding mitochondrial protein, mRNA. | -0,7234 | 9,0071 | | -4,1931 | | 0,002043 | | | 0,01305 |
| BLOC1S1 | Homo sapiens biogenesis of lysosome-related organelles complex-1, subunit 1 (BLOC1S1), mRNA. | -0,7239 | 8,3363 | | -4,4834 | | 0,001314 | | | 0,00976 |
| NUDT5 | Homo sapiens nudix (nucleoside diphosphate linked moiety X)-type motif 5 (NUDT5), mRNA. | -0,7239 | 9,6422 | | -7,2600 | | 0,000035 | | | 0,00134 |
| WDR92 | Homo sapiens WD repeat domain 92 (WDR92), mRNA. | -0,7245 | 8,0331 | | -9,2223 | | 0,000005 | | | 0,00056 |
| NDUFA6 | Homo sapiens NADH dehydrogenase (ubiquinone) 1 alpha subcomplex, 6, 14kDa (NDUFA6), nuclear gene encoding mitochondrial protein, mRNA. | -0,7247 | 8,5582 | | -6,6132 | | 0,000074 | | | 0,00192 |
| ATP2A2 | Homo sapiens ATPase, Ca++ transporting, cardiac muscle, slow twitch 2 (ATP2A2), transcript variant 2, mRNA. | -0,7250 | 9,3289 | | -4,0491 | | 0,002555 | | | 0,01526 |
| PFKM | Homo sapiens phosphofructokinase, muscle (PFKM), mRNA. | -0,7251 | 8,0890 | | -4,5239 | | 0,001237 | | | 0,00936 |
| TMX1 | Homo sapiens thioredoxin-related transmembrane protein 1 (TMX1), mRNA. | -0,7252 | 9,5247 | | -5,1624 | | 0,000490 | | | 0,00537 |
| LOC644214 | PREDICTED: Homo sapiens misc_RNA (LOC644214), miscRNA. | -0,7252 | 8,8269 | | -4,5331 | | 0,001220 | | | 0,00927 |
| ARL5A | Homo sapiens ADP-ribosylation factor-like 5A (ARL5A), transcript variant 3, mRNA. | -0,7259 | 8,5523 | | -4,4323 | | 0,001419 | | | 0,01024 |
| NDUFA3 | Homo sapiens NADH dehydrogenase (ubiquinone) 1 alpha subcomplex, 3, 9kDa (NDUFA3), mRNA. | -0,7260 | 10,7481 | | -3,7867 | | 0,003865 | | | 0,02010 |
| TIMM23 | Homo sapiens translocase of inner mitochondrial membrane 23 homolog (yeast) (TIMM23), nuclear gene encoding mitochondrial protein, mRNA. | -0,7265 | 11,3023 | | -5,5995 | | 0,000269 | | | 0,00386 |
| CCT7 | Homo sapiens chaperonin containing TCP1, subunit 7 (eta) (CCT7), transcript variant 2, mRNA. | -0,7269 | 11,9866 | | -3,9850 | | 0,002824 | | | 0,01626 |
| CES2 | Homo sapiens carboxylesterase 2 (intestine, liver) (CES2), transcript variant 1, mRNA. | -0,7277 | 8,8338 | | -4,6748 | | 0,000989 | | | 0,00819 |
| SMC4 | Homo sapiens structural maintenance of chromosomes 4 (SMC4), transcript variant 2, mRNA. | -0,7280 | 7,9573 | | -5,8978 | | 0,000182 | | | 0,00309 |
| C4orf27 | Homo sapiens chromosome 4 open reading frame 27 (C4orf27), mRNA. | -0,7285 | 7,9816 | | -6,0488 | | 0,000149 | | | 0,00277 |
| ATP5J2 | Homo sapiens ATP synthase, H+ transporting, mitochondrial F0 complex, subunit F2 (ATP5J2), nuclear gene encoding mitochondrial protein, transcript variant 1, mRNA. | -0,7285 | 11,3532 | | -6,2863 | | 0,000111 | | | 0,00235 |
| ETFB | Homo sapiens electron-transfer-flavoprotein, beta polypeptide (ETFB), transcript variant 2, mRNA. | -0,7290 | 9,2692 | | -3,2338 | | 0,009498 | | | 0,03829 |
| TSGA14 | Homo sapiens testis specific, 14 (TSGA14), mRNA. | -0,7291 | 8,0934 | | -5,6393 | | 0,000255 | | | 0,00373 |
| GLE1 | Homo sapiens GLE1 RNA export mediator homolog (yeast) (GLE1), transcript variant 1, mRNA. | -0,7310 | 9,6614 | | -5,1509 | | 0,000498 | | | 0,00540 |
| GJA1 | Homo sapiens gap junction protein, alpha 1, 43kDa (GJA1), mRNA. | -0,7325 | 8,7472 | | -3,9152 | | 0,003152 | | | 0,01745 |
| LOC399988 | PREDICTED: Homo sapiens misc_RNA (LOC399988), miscRNA. | -0,7333 | 11,8687 | | -3,2745 | | 0,008881 | | | 0,03654 |
| NAE1 | Homo sapiens NEDD8 activating enzyme E1 subunit 1 (NAE1), transcript variant 3, mRNA. | -0,7347 | 8,9904 | | -5,1231 | | 0,000518 | | | 0,00551 |
| WDR12 | Homo sapiens WD repeat domain 12 (WDR12), mRNA. | -0,7351 | 8,6759 | | -4,3573 | | 0,001589 | | | 0,01105 |
| USP39 | Homo sapiens ubiquitin specific peptidase 39 (USP39), mRNA. | -0,7358 | 8,9328 | | -5,4449 | | 0,000332 | | | 0,00430 |
| BANF1 | Homo sapiens barrier to autointegration factor 1 (BANF1), mRNA. | -0,7358 | 11,7896 | | -4,0078 | | 0,002725 | | | 0,01590 |
| UBAC1 | Homo sapiens UBA domain containing 1 (UBAC1), mRNA. | -0,7360 | 10,0576 | | -4,6042 | | 0,001097 | | | 0,00872 |
| ORC5L | Homo sapiens origin recognition complex, subunit 5-like (yeast) (ORC5L), transcript variant 1, mRNA. | -0,7369 | 9,5825 | | -4,9717 | | 0,000642 | | | 0,00622 |
| TPD52L1 | Homo sapiens tumor protein D52-like 1 (TPD52L1), transcript variant 4, mRNA. | -0,7373 | 9,8850 | | -3,2677 | | 0,008981 | | | 0,03684 |
| SET | Homo sapiens SET translocation (myeloid leukemia-associated) (SET), mRNA. | -0,7377 | 11,4004 | | -3,1921 | | 0,010178 | | | 0,04029 |
| GPATCH4 | Homo sapiens G patch domain containing 4 (GPATCH4), transcript variant 2, mRNA. | -0,7383 | 8,5444 | | -3,9298 | | 0,003080 | | | 0,01717 |
| RARA | Homo sapiens retinoic acid receptor, alpha (RARA), transcript variant 1, mRNA. | -0,7387 | 8,5230 | | -3,2864 | | 0,008708 | | | 0,03605 |
| PSMB9 | Homo sapiens proteasome (prosome, macropain) subunit, beta type, 9 (large multifunctional peptidase 2) (PSMB9), transcript variant 1, mRNA. | -0,7388 | 7,7773 | | -4,8320 | | 0,000786 | | | 0,00707 |
| FOXM1 | Homo sapiens forkhead box M1 (FOXM1), transcript variant 3, mRNA. | -0,7389 | 7,9257 | | -4,1089 | | 0,002328 | | | 0,01425 |
| FAM122B | Homo sapiens family with sequence similarity 122B (FAM122B), mRNA. | -0,7397 | 8,2674 | | -4,5108 | | 0,001261 | | | 0,00948 |
| KHSRP | Homo sapiens KH-type splicing regulatory protein (KHSRP), mRNA. | -0,7399 | 8,9746 | | -13,4850 | | 0,000000 | | | 0,00014 |
| ADORA2B | Homo sapiens adenosine A2b receptor (ADORA2B), mRNA. | -0,7402 | 10,1888 | | -3,6109 | | 0,005126 | | | 0,02455 |
| LOC644774 | PREDICTED: Homo sapiens similar to Phosphoglycerate kinase 1 (LOC644774), mRNA. | -0,7407 | 10,8172 | | -6,2428 | | 0,000117 | | | 0,00242 |
| ATP5D | Homo sapiens ATP synthase, H+ transporting, mitochondrial F1 complex, delta subunit (ATP5D), nuclear gene encoding mitochondrial protein, transcript variant 1, mRNA. | -0,7412 | 10,3102 | | -7,2540 | | 0,000035 | | | 0,00135 |
| DGCR6 | Homo sapiens DiGeorge syndrome critical region gene 6 (DGCR6), mRNA. | -0,7416 | 9,3351 | | -4,4239 | | 0,001437 | | | 0,01034 |
| ALDH1B1 | Homo sapiens aldehyde dehydrogenase 1 family, member B1 (ALDH1B1), nuclear gene encoding mitochondrial protein, mRNA. | -0,7416 | 8,0045 | | -7,3231 | | 0,000032 | | | 0,00130 |
| SPCS1 | Homo sapiens signal peptidase complex subunit 1 homolog (S. cerevisiae) (SPCS1), mRNA. | -0,7418 | 11,2985 | | -5,6442 | | 0,000254 | | | 0,00372 |
| PPAT | Homo sapiens phosphoribosyl pyrophosphate amidotransferase (PPAT), mRNA. | -0,7419 | 8,3192 | | -6,1485 | | 0,000132 | | | 0,00258 |
| ASF1A | Homo sapiens ASF1 anti-silencing function 1 homolog A (S. cerevisiae) (ASF1A), mRNA. | -0,7420 | 7,8749 | | -5,8319 | | 0,000198 | | | 0,00324 |
| LSM2 | Homo sapiens LSM2 homolog, U6 small nuclear RNA associated (S. cerevisiae) (LSM2), mRNA. | -0,7421 | 9,8624 | | -5,9423 | | 0,000171 | | | 0,00301 |
| C6orf108 | Homo sapiens chromosome 6 open reading frame 108 (C6orf108), transcript variant 2, mRNA. | -0,7424 | 8,8827 | | -3,0821 | | 0,012219 | | | 0,04619 |
| BARD1 | Homo sapiens BRCA1 associated RING domain 1 (BARD1), mRNA. | -0,7427 | 7,9559 | | -5,4348 | | 0,000336 | | | 0,00433 |
| OBFC2B | Homo sapiens oligonucleotide/oligosaccharide-binding fold containing 2B (OBFC2B), mRNA. | -0,7429 | 8,5399 | | -7,4369 | | 0,000029 | | | 0,00124 |
| LYPLAL1 | Homo sapiens lysophospholipase-like 1 (LYPLAL1), mRNA. | -0,7431 | 8,7789 | | -3,8842 | | 0,003310 | | | 0,01806 |
| C9orf142 | Homo sapiens chromosome 9 open reading frame 142 (C9orf142), mRNA. | -0,7432 | 9,2946 | | -3,1912 | | 0,010194 | | | 0,04033 |
| NSF | Homo sapiens N-ethylmaleimide-sensitive factor (NSF), mRNA. | -0,7432 | 9,0177 | | -3,9607 | | 0,002934 | | | 0,01667 |
| MRPL23 | Homo sapiens mitochondrial ribosomal protein L23 (MRPL23), nuclear gene encoding mitochondrial protein, mRNA. | -0,7439 | 9,9433 | | -8,0079 | | 0,000015 | | | 0,00095 |
| COMMD8 | Homo sapiens COMM domain containing 8 (COMMD8), mRNA. | -0,7442 | 9,4689 | | -4,7837 | | 0,000843 | | | 0,00742 |
| ZNF252 | Homo sapiens zinc finger protein 252 (ZNF252), non-coding RNA. | -0,7445 | 8,5515 | | -5,5652 | | 0,000282 | | | 0,00394 |
| TYSND1 | Homo sapiens trypsin domain containing 1 (TYSND1), transcript variant 2, mRNA. | -0,7451 | 9,0412 | | -5,1233 | | 0,000518 | | | 0,00551 |
| PPA2 | Homo sapiens pyrophosphatase (inorganic) 2 (PPA2), nuclear gene encoding mitochondrial protein, transcript variant 3, mRNA. | -0,7456 | 8,8628 | | -3,8654 | | 0,003411 | | | 0,01841 |
| CCT7 | Homo sapiens chaperonin containing TCP1, subunit 7 (eta) (CCT7), transcript variant 1, mRNA. | -0,7458 | 10,0558 | | -5,4410 | | 0,000334 | | | 0,00431 |
| TPI1 | Homo sapiens triosephosphate isomerase 1 (TPI1), mRNA. | -0,7464 | 12,5332 | | -8,1318 | | 0,000014 | | | 0,00092 |
| DAG1 | Homo sapiens dystroglycan 1 (dystrophin-associated glycoprotein 1) (DAG1), mRNA. | -0,7465 | 8,9931 | | -3,1097 | | 0,011670 | | | 0,04454 |
| TIPIN | Homo sapiens TIMELESS interacting protein (TIPIN), mRNA. | -0,7470 | 7,8006 | | -6,5820 | | 0,000077 | | | 0,00197 |
| UQCRQ | Homo sapiens ubiquinol-cytochrome c reductase, complex III subunit VII, 9.5kDa (UQCRQ), nuclear gene encoding mitochondrial protein, mRNA. | -0,7476 | 12,2737 | | -3,1306 | | 0,011272 | | | 0,04343 |
| ATP5J | Homo sapiens ATP synthase, H+ transporting, mitochondrial F0 complex, subunit F6 (ATP5J), nuclear gene encoding mitochondrial protein, transcript variant 5, mRNA. | -0,7489 | 11,1319 | | -6,3497 | | 0,000102 | | | 0,00226 |
| LSMD1 | Homo sapiens LSM domain containing 1 (LSMD1), mRNA. | -0,7496 | 8,4029 | | -6,6561 | | 0,000070 | | | 0,00188 |
| TUBB | Homo sapiens tubulin, beta (TUBB), mRNA. | -0,7508 | 11,3437 | | -4,8317 | | 0,000786 | | | 0,00707 |
| LOC728026 | PREDICTED: Homo sapiens hypothetical LOC728026 (LOC728026), mRNA. | -0,7511 | 8,9014 | | -3,6481 | | 0,004827 | | | 0,02352 |
| PIGY | Homo sapiens phosphatidylinositol glycan anchor biosynthesis, class Y (PIGY), transcript variant 2, mRNA. | -0,7512 | 10,6548 | | -3,9218 | | 0,003120 | | | 0,01731 |
| PRPF4 | Homo sapiens PRP4 pre-mRNA processing factor 4 homolog (yeast) (PRPF4), mRNA. | -0,7514 | 10,4168 | | -5,8562 | | 0,000192 | | | 0,00319 |
| ESD | Homo sapiens esterase D/formylglutathione hydrolase (ESD), mRNA. | -0,7533 | 10,7530 | | -4,2390 | | 0,001904 | | | 0,01243 |
| METTL1 | Homo sapiens methyltransferase like 1 (METTL1), transcript variant 1, mRNA. | -0,7539 | 8,7283 | | -3,8019 | | 0,003773 | | | 0,01974 |
| OAT | Homo sapiens ornithine aminotransferase (gyrate atrophy) (OAT), nuclear gene encoding mitochondrial protein, mRNA. | -0,7541 | 10,4495 | | -5,7057 | | 0,000234 | | | 0,00355 |
| RBBP8 | Homo sapiens retinoblastoma binding protein 8 (RBBP8), transcript variant 2, mRNA. | -0,7543 | 8,7604 | | -4,2036 | | 0,002010 | | | 0,01291 |
| MCCC1 | Homo sapiens methylcrotonoyl-Coenzyme A carboxylase 1 (alpha) (MCCC1), nuclear gene encoding mitochondrial protein, mRNA. | -0,7545 | 8,7169 | | -3,4336 | | 0,006838 | | | 0,03043 |
| LOC647302 | PREDICTED: Homo sapiens misc_RNA (LOC647302), miscRNA. | -0,7550 | 10,2793 | | -5,1416 | | 0,000505 | | | 0,00544 |
| C7orf27 | Homo sapiens chromosome 7 open reading frame 27 (C7orf27), mRNA. | -0,7553 | 8,9610 | | -4,3949 | | 0,001501 | | | 0,01065 |
| MRPL11 | Homo sapiens mitochondrial ribosomal protein L11 (MRPL11), nuclear gene encoding mitochondrial protein, transcript variant 2, mRNA. | -0,7553 | 9,2549 | | -3,7048 | | 0,004406 | | | 0,02206 |
| NUP93 | Homo sapiens nucleoporin 93kDa (NUP93), mRNA. | -0,7557 | 10,0050 | | -3,8747 | | 0,003361 | | | 0,01826 |
| ENDOD1 | Homo sapiens endonuclease domain containing 1 (ENDOD1), mRNA. | -0,7565 | 8,3262 | | -5,1004 | | 0,000535 | | | 0,00562 |
| PIGU | Homo sapiens phosphatidylinositol glycan anchor biosynthesis, class U (PIGU), mRNA. | -0,7567 | 8,5406 | | -4,0054 | | 0,002735 | | | 0,01595 |
| POLR3GL | Homo sapiens polymerase (RNA) III (DNA directed) polypeptide G (32kD)-like (POLR3GL), mRNA. | -0,7567 | 9,0063 | | -3,6722 | | 0,004643 | | | 0,02289 |
| SNRPB | Homo sapiens small nuclear ribonucleoprotein polypeptides B and B1 (SNRPB), transcript variant 2, mRNA. | -0,7568 | 11,6675 | | -4,3401 | | 0,001631 | | | 0,01121 |
| PCNA | Homo sapiens proliferating cell nuclear antigen (PCNA), transcript variant 2, mRNA. | -0,7568 | 7,8108 | | -6,4267 | | 0,000093 | | | 0,00216 |
| KIAA0020 | Homo sapiens KIAA0020 (KIAA0020), mRNA. | -0,7569 | 9,2595 | | -4,4966 | | 0,001288 | | | 0,00962 |
| UQCRB | Homo sapiens ubiquinol-cytochrome c reductase binding protein (UQCRB), mRNA. | -0,7569 | 9,4763 | | -5,7038 | | 0,000234 | | | 0,00356 |
| PSMB2 | Homo sapiens proteasome (prosome, macropain) subunit, beta type, 2 (PSMB2), mRNA. | -0,7579 | 11,5958 | | -9,8266 | | 0,000003 | | | 0,00048 |
| RPS24 | Homo sapiens ribosomal protein S24 (RPS24), transcript variant 2, mRNA. | -0,7582 | 10,4835 | | -4,4324 | | 0,001419 | | | 0,01024 |
| MRPL1 | Homo sapiens mitochondrial ribosomal protein L1 (MRPL1), nuclear gene encoding mitochondrial protein, mRNA. | -0,7586 | 8,3416 | | -4,8849 | | 0,000728 | | | 0,00671 |
| ADAT1 | Homo sapiens adenosine deaminase, tRNA-specific 1 (ADAT1), mRNA. | -0,7600 | 8,4867 | | -6,9400 | | 0,000050 | | | 0,00162 |
| EEF1E1 | Homo sapiens eukaryotic translation elongation factor 1 epsilon 1 (EEF1E1), mRNA. | -0,7601 | 9,9294 | | -3,8719 | | 0,003376 | | | 0,01829 |
| BRCA1 | Homo sapiens breast cancer 1, early onset (BRCA1), transcript variant BRCA1-delta14-17, mRNA. | -0,7603 | 7,7884 | | -7,3497 | | 0,000031 | | | 0,00129 |
| C17orf42 | Homo sapiens chromosome 17 open reading frame 42 (C17orf42), mRNA. | -0,7605 | 8,6945 | | -6,4742 | | 0,000088 | | | 0,00210 |
| TEAD2 | Homo sapiens TEA domain family member 2 (TEAD2), mRNA. | -0,7616 | 8,8958 | | -3,4630 | | 0,006517 | | | 0,02937 |
| NDUFA2 | Homo sapiens NADH dehydrogenase (ubiquinone) 1 alpha subcomplex, 2, 8kDa (NDUFA2), mRNA. | -0,7621 | 10,4491 | | -4,2521 | | 0,001866 | | | 0,01226 |
| TMEM203 | Homo sapiens transmembrane protein 203 (TMEM203), mRNA. | -0,7637 | 9,3955 | | -6,3209 | | 0,000106 | | | 0,00229 |
| SPRY1 | Homo sapiens sprouty homolog 1, antagonist of FGF signaling (Drosophila) (SPRY1), transcript variant 2, mRNA. | -0,7637 | 7,7023 | | -6,5964 | | 0,000076 | | | 0,00194 |
| CNTNAP2 | Homo sapiens contactin associated protein-like 2 (CNTNAP2), mRNA. | -0,7638 | 7,5674 | | -5,9303 | | 0,000174 | | | 0,00303 |
| TM2D3 | Homo sapiens TM2 domain containing 3 (TM2D3), transcript variant 1, mRNA. | -0,7639 | 8,9371 | | -6,8067 | | 0,000059 | | | 0,00174 |
| TSPAN6 | Homo sapiens tetraspanin 6 (TSPAN6), mRNA. | -0,7645 | 8,1623 | | -3,7806 | | 0,003903 | | | 0,02022 |
| APOO | Homo sapiens apolipoprotein O (APOO), mRNA. | -0,7648 | 8,6478 | | -4,2527 | | 0,001864 | | | 0,01225 |
| NUP62 | Homo sapiens nucleoporin 62kDa (NUP62), transcript variant 2, mRNA. | -0,7653 | 11,1262 | | -5,5265 | | 0,000297 | | | 0,00405 |
| UROS | Homo sapiens uroporphyrinogen III synthase (congenital erythropoietic porphyria) (UROS), mRNA. | -0,7654 | 8,0348 | | -5,4768 | | 0,000318 | | | 0,00421 |
| RAVER1 | Homo sapiens RAVER1 (RAVER1), mRNA. | -0,7656 | 8,5214 | | -10,0765 | | 0,000002 | | | 0,00043 |
| TMEM14D | PREDICTED: Homo sapiens transmembrane protein 14D (TMEM14D), mRNA. | -0,7662 | 9,6917 | | -5,8762 | | 0,000187 | | | 0,00313 |
| LAP3 | Homo sapiens leucine aminopeptidase 3 (LAP3), mRNA. | -0,7666 | 9,0336 | | -4,1683 | | 0,002123 | | | 0,01339 |
| NCL | Homo sapiens nucleolin (NCL), mRNA. | -0,7668 | 9,1628 | | -6,3379 | | 0,000104 | | | 0,00227 |
| CCDC58 | Homo sapiens coiled-coil domain containing 58 (CCDC58), mRNA. | -0,7679 | 7,8666 | | -5,4941 | | 0,000310 | | | 0,00416 |
| MPHOSPH10 | Homo sapiens M-phase phosphoprotein 10 (U3 small nucleolar ribonucleoprotein) (MPHOSPH10), mRNA. | -0,7679 | 9,1748 | | -7,6713 | | 0,000022 | | | 0,00113 |
| RAB3IP | Homo sapiens RAB3A interacting protein (rabin3) (RAB3IP), transcript variant A, mRNA. | -0,7680 | 9,3180 | | -6,6716 | | 0,000069 | | | 0,00186 |
| CXorf57 | Homo sapiens chromosome X open reading frame 57 (CXorf57), mRNA. | -0,7680 | 7,9927 | | -6,2217 | | 0,000120 | | | 0,00245 |
| EI24 | Homo sapiens etoposide induced 2.4 mRNA (EI24), transcript variant 2, mRNA. | -0,7684 | 10,2673 | | -8,0544 | | 0,000015 | | | 0,00095 |
| VDAC3 | Homo sapiens voltage-dependent anion channel 3 (VDAC3), mRNA. | -0,7686 | 11,0458 | | -7,4414 | | 0,000028 | | | 0,00124 |
| TMEM19 | Homo sapiens transmembrane protein 19 (TMEM19), mRNA. | -0,7686 | 7,9373 | | -6,5522 | | 0,000080 | | | 0,00200 |
| ATP5J | Homo sapiens ATP synthase, H+ transporting, mitochondrial F0 complex, subunit F6 (ATP5J), nuclear gene encoding mitochondrial protein, transcript variant 3, mRNA. | -0,7699 | 11,2008 | | -5,5904 | | 0,000273 | | | 0,00386 |
| JMJD8 | Homo sapiens jumonji domain containing 8 (JMJD8), mRNA. | -0,7701 | 9,6333 | | -5,1196 | | 0,000521 | | | 0,00553 |
| LOC644877 | PREDICTED: Homo sapiens misc_RNA (LOC644877), miscRNA. | -0,7707 | 8,3781 | | -8,2725 | | 0,000012 | | | 0,00086 |
| SFRS9 | Homo sapiens splicing factor, arginine/serine-rich 9 (SFRS9), mRNA. | -0,7709 | 11,5565 | | -7,0540 | | 0,000044 | | | 0,00151 |
| C7orf30 | Homo sapiens chromosome 7 open reading frame 30 (C7orf30), mRNA. | -0,7715 | 10,6581 | | -5,8522 | | 0,000193 | | | 0,00320 |
| LOC389049 | PREDICTED: Homo sapiens misc_RNA (LOC389049), miscRNA. | -0,7718 | 8,2975 | | -4,3833 | | 0,001528 | | | 0,01078 |
| PTGES2 | Homo sapiens prostaglandin E synthase 2 (PTGES2), transcript variant 2, mRNA. | -0,7724 | 9,1341 | | -5,5648 | | 0,000282 | | | 0,00394 |
| THYN1 | Homo sapiens thymocyte nuclear protein 1 (THYN1), transcript variant 2, mRNA. | -0,7726 | 8,2787 | | -5,5512 | | 0,000287 | | | 0,00398 |
| RPL36A | Homo sapiens ribosomal protein L36a (RPL36A), mRNA. | -0,7726 | 9,3360 | | -3,6929 | | 0,004491 | | | 0,02239 |
| MFSD3 | Homo sapiens major facilitator superfamily domain containing 3 (MFSD3), mRNA. | -0,7738 | 8,7756 | | -4,1924 | | 0,002046 | | | 0,01306 |
| NUPL2 | Homo sapiens nucleoporin like 2 (NUPL2), mRNA. | -0,7742 | 8,7950 | | -6,3801 | | 0,000099 | | | 0,00222 |
| USP1 | Homo sapiens ubiquitin specific peptidase 1 (USP1), transcript variant 3, mRNA. | -0,7772 | 8,8235 | | -3,9113 | | 0,003171 | | | 0,01751 |
| TXNDC14 | Homo sapiens thioredoxin domain containing 14 (TXNDC14), mRNA. | -0,7777 | 9,5796 | | -4,5178 | | 0,001248 | | | 0,00941 |
| GLA | Homo sapiens galactosidase, alpha (GLA), mRNA. | -0,7777 | 9,5289 | | -5,7909 | | 0,000209 | | | 0,00333 |
| PSMA4 | Homo sapiens proteasome (prosome, macropain) subunit, alpha type, 4 (PSMA4), mRNA. | -0,7778 | 9,5322 | | -9,4266 | | 0,000004 | | | 0,00052 |
| CBY1 | Homo sapiens chibby homolog 1 (Drosophila) (CBY1), transcript variant 1, mRNA. | -0,7780 | 8,4784 | | -4,0930 | | 0,002386 | | | 0,01452 |
| ATP5O | Homo sapiens ATP synthase, H+ transporting, mitochondrial F1 complex, O subunit (ATP5O), nuclear gene encoding mitochondrial protein, mRNA. | -0,7781 | 11,5345 | | -4,9711 | | 0,000643 | | | 0,00623 |
| WDR67 | Homo sapiens WD repeat domain 67 (WDR67), mRNA. | -0,7783 | 8,0112 | | -4,3275 | | 0,001663 | | | 0,01136 |
| SIGMAR1 | Homo sapiens sigma non-opioid intracellular receptor 1 (SIGMAR1), transcript variant 2, mRNA. | -0,7790 | 8,5095 | | -3,6112 | | 0,005123 | | | 0,02454 |
| CPT2 | Homo sapiens carnitine palmitoyltransferase II (CPT2), nuclear gene encoding mitochondrial protein, mRNA. | -0,7792 | 8,6034 | | -8,1631 | | 0,000013 | | | 0,00090 |
| AKR7A3 | Homo sapiens aldo-keto reductase family 7, member A3 (aflatoxin aldehyde reductase) (AKR7A3), mRNA. | -0,7792 | 8,7591 | | -6,6250 | | 0,000073 | | | 0,00191 |
| EFTUD2 | Homo sapiens elongation factor Tu GTP binding domain containing 2 (EFTUD2), mRNA. | -0,7796 | 9,7767 | | -7,2077 | | 0,000037 | | | 0,00139 |
| WDR18 | Homo sapiens WD repeat domain 18 (WDR18), mRNA. | -0,7798 | 9,9679 | | -3,4319 | | 0,006857 | | | 0,03049 |
| C14orf106 | Homo sapiens chromosome 14 open reading frame 106 (C14orf106), mRNA. | -0,7799 | 8,1655 | | -5,1522 | | 0,000497 | | | 0,00540 |
| NOP56 | Homo sapiens NOP56 ribonucleoprotein homolog (yeast) (NOP56), transcript variant 1, mRNA. | -0,7800 | 9,0135 | | -5,2858 | | 0,000413 | | | 0,00486 |
| AUTS2 | Homo sapiens autism susceptibility candidate 2 (AUTS2), mRNA. | -0,7810 | 9,0293 | | -5,8949 | | 0,000182 | | | 0,00309 |
| RAN | Homo sapiens RAN, member RAS oncogene family (RAN), mRNA. | -0,7812 | 9,2261 | | -4,0691 | | 0,002476 | | | 0,01491 |
| TIMM8A | Homo sapiens translocase of inner mitochondrial membrane 8 homolog A (yeast) (TIMM8A), nuclear gene encoding mitochondrial protein, mRNA. | -0,7813 | 8,0518 | | -5,8896 | | 0,000184 | | | 0,00310 |
| RFC3 | Homo sapiens replication factor C (activator 1) 3, 38kDa (RFC3), transcript variant 1, mRNA. | -0,7815 | 7,8493 | | -6,1325 | | 0,000134 | | | 0,00262 |
| HMGB1L1 | Homo sapiens high-mobility group box 1-like 1 (HMGB1L1), mRNA. | -0,7823 | 9,0127 | | -4,0701 | | 0,002473 | | | 0,01490 |
| ATP5H | Homo sapiens ATP synthase, H+ transporting, mitochondrial F0 complex, subunit d (ATP5H), nuclear gene encoding mitochondrial protein, transcript variant 1, mRNA. | -0,7834 | 12,1729 | | -3,6392 | | 0,004897 | | | 0,02377 |
| NONO | Homo sapiens non-POU domain containing, octamer-binding (NONO), mRNA. | -0,7835 | 11,8938 | | -5,0597 | | 0,000567 | | | 0,00582 |
| M6PR | Homo sapiens mannose-6-phosphate receptor (cation dependent) (M6PR), mRNA. | -0,7837 | 9,4991 | | -7,3505 | | 0,000031 | | | 0,00129 |
| NFIB | Homo sapiens nuclear factor I/B (NFIB), mRNA. | -0,7854 | 9,0586 | | -3,5184 | | 0,005955 | | | 0,02751 |
| PKMYT1 | Homo sapiens protein kinase, membrane associated tyrosine/threonine 1 (PKMYT1), transcript variant 2, mRNA. | -0,7855 | 7,9513 | | -5,0102 | | 0,000608 | | | 0,00606 |
| LOC644877 | PREDICTED: Homo sapiens misc_RNA (LOC644877), miscRNA. | -0,7861 | 8,8236 | | -8,1063 | | 0,000014 | | | 0,00093 |
| LOC375295 | PREDICTED: Homo sapiens hypothetical gene supported by BC013438 (LOC375295), mRNA. | -0,7872 | 8,4069 | | -6,5312 | | 0,000082 | | | 0,00201 |
| NUPL2 | Homo sapiens nucleoporin like 2 (NUPL2), mRNA. | -0,7876 | 8,7575 | | -7,6155 | | 0,000023 | | | 0,00115 |
| ERH | Homo sapiens enhancer of rudimentary homolog (Drosophila) (ERH), mRNA. | -0,7889 | 10,0130 | | -4,0676 | | 0,002482 | | | 0,01493 |
| ALG8 | Homo sapiens asparagine-linked glycosylation 8, alpha-1,3-glucosyltransferase homolog (S. cerevisiae) (ALG8), transcript variant 1, mRNA. | -0,7896 | 8,5993 | | -3,8967 | | 0,003246 | | | 0,01782 |
| C17orf90 | Homo sapiens chromosome 17 open reading frame 90 (C17orf90), mRNA. | -0,7899 | 8,7970 | | -5,7023 | | 0,000235 | | | 0,00356 |
| UCHL5 | Homo sapiens ubiquitin carboxyl-terminal hydrolase L5 (UCHL5), mRNA. | -0,7909 | 9,0938 | | -5,0806 | | 0,000550 | | | 0,00572 |
| BASP1 | Homo sapiens brain abundant, membrane attached signal protein 1 (BASP1), mRNA. | -0,7913 | 12,8175 | | -5,9452 | | 0,000171 | | | 0,00301 |
| PPP2CA | Homo sapiens protein phosphatase 2 (formerly 2A), catalytic subunit, alpha isoform (PPP2CA), mRNA. | -0,7922 | 9,6409 | | -4,5923 | | 0,001117 | | | 0,00880 |
| GGCT | Homo sapiens gamma-glutamyl cyclotransferase (GGCT), mRNA. | -0,7930 | 9,0096 | | -5,5437 | | 0,000290 | | | 0,00400 |
| CDCA2 | Homo sapiens cell division cycle associated 2 (CDCA2), mRNA. | -0,7933 | 7,7635 | | -6,6989 | | 0,000067 | | | 0,00183 |
| CASP1 | Homo sapiens caspase 1, apoptosis-related cysteine peptidase (interleukin 1, beta, convertase) (CASP1), transcript variant delta, mRNA. | -0,7939 | 8,0759 | | -3,9308 | | 0,003076 | | | 0,01715 |
| HSPH1 | Homo sapiens heat shock 105kDa/110kDa protein 1 (HSPH1), mRNA. | -0,7953 | 11,0248 | | -3,1421 | | 0,011058 | | | 0,04281 |
| SLBP | Homo sapiens stem-loop binding protein (SLBP), mRNA. | -0,7960 | 8,9663 | | -6,4157 | | 0,000094 | | | 0,00216 |
| C20orf27 | Homo sapiens chromosome 20 open reading frame 27 (C20orf27), mRNA. | -0,7961 | 8,5907 | | -6,2054 | | 0,000122 | | | 0,00247 |
| NBN | Homo sapiens nibrin (NBN), mRNA. | -0,7963 | 8,4677 | | -3,2486 | | 0,009270 | | | 0,03759 |
| RPUSD2 | Homo sapiens RNA pseudouridylate synthase domain containing 2 (RPUSD2), mRNA. | -0,7964 | 8,6312 | | -5,0646 | | 0,000563 | | | 0,00579 |
| FAM173B | Homo sapiens family with sequence similarity 173, member B (FAM173B), mRNA. | -0,7964 | 7,9320 | | -5,6221 | | 0,000261 | | | 0,00379 |
| CTPS | Homo sapiens CTP synthase (CTPS), mRNA. | -0,7966 | 9,1640 | | -3,6099 | | 0,005134 | | | 0,02456 |
| PCGF6 | Homo sapiens polycomb group ring finger 6 (PCGF6), transcript variant 2, mRNA. | -0,7972 | 8,7539 | | -5,5871 | | 0,000274 | | | 0,00387 |
| TFAM | Homo sapiens transcription factor A, mitochondrial (TFAM), nuclear gene encoding mitochondrial protein, mRNA. | -0,7972 | 8,3784 | | -6,9247 | | 0,000051 | | | 0,00163 |
| XPO1 | Homo sapiens exportin 1 (CRM1 homolog, yeast) (XPO1), mRNA. | -0,7974 | 9,9267 | | -5,3945 | | 0,000355 | | | 0,00441 |
| NUDT9 | Homo sapiens nudix (nucleoside diphosphate linked moiety X)-type motif 9 (NUDT9), transcript variant 3, mRNA. | -0,7974 | 8,4733 | | -7,7745 | | 0,000020 | | | 0,00107 |
| NIF3L1 | Homo sapiens NIF3 NGG1 interacting factor 3-like 1 (S. pombe) (NIF3L1), mRNA. | -0,7981 | 9,8107 | | -5,4335 | | 0,000337 | | | 0,00433 |
| TMEM5 | Homo sapiens transmembrane protein 5 (TMEM5), mRNA. | -0,7981 | 8,8118 | | -5,5964 | | 0,000270 | | | 0,00386 |
| PSMC2 | Homo sapiens proteasome (prosome, macropain) 26S subunit, ATPase, 2 (PSMC2), mRNA. | -0,7984 | 11,9559 | | -7,4814 | | 0,000027 | | | 0,00121 |
| TPM1 | Homo sapiens tropomyosin 1 (alpha) (TPM1), transcript variant 7, mRNA. | -0,7989 | 8,4107 | | -3,0637 | | 0,012600 | | | 0,04736 |
| NUDCD2 | Homo sapiens NudC domain containing 2 (NUDCD2), mRNA. | -0,7992 | 9,1194 | | -5,3391 | | 0,000384 | | | 0,00463 |
| C3orf14 | Homo sapiens chromosome 3 open reading frame 14 (C3orf14), mRNA. | -0,8001 | 8,5355 | | -5,1561 | | 0,000495 | | | 0,00538 |
| NMU | Homo sapiens neuromedin U (NMU), mRNA. | -0,8004 | 7,7383 | | -4,6762 | | 0,000986 | | | 0,00819 |
| LSM3 | Homo sapiens LSM3 homolog, U6 small nuclear RNA associated (S. cerevisiae) (LSM3), mRNA. | -0,8013 | 10,4569 | | -7,2464 | | 0,000035 | | | 0,00135 |
| DLEU1 | Homo sapiens deleted in lymphocytic leukemia 1 (non-protein coding) (DLEU1), non-coding RNA. | -0,8015 | 8,0297 | | -5,9412 | | 0,000172 | | | 0,00301 |
| PSMA3 | Homo sapiens proteasome (prosome, macropain) subunit, alpha type, 3 (PSMA3), transcript variant 1, mRNA. | -0,8054 | 9,1807 | | -6,8225 | | 0,000058 | | | 0,00173 |
| AP2S1 | Homo sapiens adaptor-related protein complex 2, sigma 1 subunit (AP2S1), transcript variant AP17, mRNA. | -0,8055 | 11,4483 | | -4,1640 | | 0,002137 | | | 0,01344 |
| WBP11 | Homo sapiens WW domain binding protein 11 (WBP11), mRNA. | -0,8055 | 10,4540 | | -6,7194 | | 0,000065 | | | 0,00182 |
| TSEN2 | Homo sapiens tRNA splicing endonuclease 2 homolog (S. cerevisiae) (TSEN2), mRNA. | -0,8061 | 8,3594 | | -4,1725 | | 0,002109 | | | 0,01333 |
| NUP85 | Homo sapiens nucleoporin 85kDa (NUP85), mRNA. | -0,8062 | 9,7411 | | -5,8947 | | 0,000182 | | | 0,00309 |
| DDX18 | Homo sapiens DEAD (Asp-Glu-Ala-Asp) box polypeptide 18 (DDX18), mRNA. | -0,8072 | 10,1330 | | -7,2662 | | 0,000035 | | | 0,00134 |
| C5orf13 | Homo sapiens chromosome 5 open reading frame 13 (C5orf13), mRNA. | -0,8080 | 7,8182 | | -4,5863 | | 0,001127 | | | 0,00884 |
| SNRPC | Homo sapiens small nuclear ribonucleoprotein polypeptide C (SNRPC), mRNA. | -0,8081 | 9,6424 | | -6,8173 | | 0,000058 | | | 0,00173 |
| LOC653381 | PREDICTED: Homo sapiens similar to Sorbitol dehydrogenase (L-iditol 2-dehydrogenase) (LOC653381), mRNA. | -0,8082 | 10,3195 | | -4,1607 | | 0,002148 | | | 0,01348 |
| CDCA7L | Homo sapiens cell division cycle associated 7-like (CDCA7L), mRNA. | -0,8083 | 8,5382 | | -6,0104 | | 0,000157 | | | 0,00285 |
| RABEPK | Homo sapiens Rab9 effector protein with kelch motifs (RABEPK), mRNA. | -0,8093 | 10,3201 | | -3,3644 | | 0,007659 | | | 0,03301 |
| LAGE3 | Homo sapiens L antigen family, member 3 (LAGE3), mRNA. | -0,8094 | 10,2583 | | -4,4805 | | 0,001320 | | | 0,00979 |
| LOC644563 | PREDICTED: Homo sapiens misc_RNA (LOC644563), miscRNA. | -0,8110 | 9,5630 | | -5,2106 | | 0,000458 | | | 0,00517 |
| C3orf37 | Homo sapiens chromosome 3 open reading frame 37 (C3orf37), transcript variant 2, mRNA. | -0,8117 | 8,7180 | | -7,8053 | | 0,000019 | | | 0,00105 |
| TOMM22 | Homo sapiens translocase of outer mitochondrial membrane 22 homolog (yeast) (TOMM22), nuclear gene encoding mitochondrial protein, mRNA. | -0,8145 | 9,3287 | | -6,8624 | | 0,000055 | | | 0,00169 |
| NHP2 | Homo sapiens NHP2 ribonucleoprotein homolog (yeast) (NHP2), transcript variant 2, mRNA. | -0,8148 | 10,6046 | | -3,6359 | | 0,004923 | | | 0,02387 |
| RBM23 | Homo sapiens RNA binding motif protein 23 (RBM23), transcript variant 3, mRNA. | -0,8157 | 9,5000 | | -10,7536 | | 0,000001 | | | 0,00034 |
| ISOC1 | Homo sapiens isochorismatase domain containing 1 (ISOC1), mRNA. | -0,8161 | 8,5144 | | -5,9693 | | 0,000166 | | | 0,00294 |
| MRPS7 | Homo sapiens mitochondrial ribosomal protein S7 (MRPS7), nuclear gene encoding mitochondrial protein, mRNA. | -0,8165 | 9,1145 | | -6,1684 | | 0,000128 | | | 0,00254 |
| BCL2L12 | Homo sapiens BCL2-like 12 (proline rich) (BCL2L12), transcript variant 3, mRNA. | -0,8175 | 9,3235 | | -7,0211 | | 0,000046 | | | 0,00153 |
| HNRNPH3 | Homo sapiens heterogeneous nuclear ribonucleoprotein H3 (2H9) (HNRNPH3), transcript variant 2H9, mRNA. | -0,8189 | 8,3519 | | -3,8750 | | 0,003359 | | | 0,01825 |
| FANCG | Homo sapiens Fanconi anemia, complementation group G (FANCG), mRNA. | -0,8190 | 8,3841 | | -3,9086 | | 0,003186 | | | 0,01757 |
| PRICKLE1 | Homo sapiens prickle homolog 1 (Drosophila) (PRICKLE1), mRNA. | -0,8198 | 7,9931 | | -10,9144 | | 0,000001 | | | 0,00031 |
| BEX1 | Homo sapiens brain expressed, X-linked 1 (BEX1), mRNA. | -0,8198 | 8,0820 | | -7,3218 | | 0,000032 | | | 0,00130 |
| CHEK1 | Homo sapiens CHK1 checkpoint homolog (S. pombe) (CHEK1), mRNA. | -0,8202 | 8,3382 | | -4,9673 | | 0,000646 | | | 0,00624 |
| DHX9 | Homo sapiens DEAH (Asp-Glu-Ala-His) box polypeptide 9 (DHX9), mRNA. | -0,8208 | 8,2539 | | -5,6562 | | 0,000250 | | | 0,00368 |
| C13orf34 | Homo sapiens chromosome 13 open reading frame 34 (C13orf34), mRNA. | -0,8211 | 8,4124 | | -4,5190 | | 0,001246 | | | 0,00940 |
| PSMD10 | Homo sapiens proteasome (prosome, macropain) 26S subunit, non-ATPase, 10 (PSMD10), transcript variant 1, mRNA. | -0,8216 | 10,5631 | | -7,2556 | | 0,000035 | | | 0,00135 |
| LOC647081 | PREDICTED: Homo sapiens misc_RNA (LOC647081), miscRNA. | -0,8220 | 9,0372 | | -5,4252 | | 0,000341 | | | 0,00434 |
| LOC387703 | PREDICTED: Homo sapiens misc_RNA (LOC387703), miscRNA. | -0,8223 | 8,8848 | | -4,9205 | | 0,000691 | | | 0,00650 |
| NAT10 | Homo sapiens N-acetyltransferase 10 (GCN5-related) (NAT10), mRNA. | -0,8239 | 8,7680 | | -5,0149 | | 0,000604 | | | 0,00603 |
| ACTL6A | Homo sapiens actin-like 6A (ACTL6A), transcript variant 1, mRNA. | -0,8240 | 10,7480 | | -5,1010 | | 0,000535 | | | 0,00562 |
| PSMD1 | Homo sapiens proteasome (prosome, macropain) 26S subunit, non-ATPase, 1 (PSMD1), mRNA. | -0,8243 | 9,7009 | | -4,9488 | | 0,000664 | | | 0,00633 |
| CASP1 | Homo sapiens caspase 1, apoptosis-related cysteine peptidase (interleukin 1, beta, convertase) (CASP1), transcript variant delta, mRNA. | -0,8248 | 8,0456 | | -3,6949 | | 0,004477 | | | 0,02234 |
| HDDC2 | Homo sapiens HD domain containing 2 (HDDC2), mRNA. | -0,8250 | 10,5614 | | -4,4360 | | 0,001411 | | | 0,01022 |
| CYC1 | Homo sapiens cytochrome c-1 (CYC1), mRNA. | -0,8250 | 12,0743 | | -4,5586 | | 0,001174 | | | 0,00909 |
| ACADM | Homo sapiens acyl-Coenzyme A dehydrogenase, C-4 to C-12 straight chain (ACADM), nuclear gene encoding mitochondrial protein, mRNA. | -0,8256 | 9,0688 | | -5,3776 | | 0,000364 | | | 0,00448 |
| UQCRFS1 | Homo sapiens ubiquinol-cytochrome c reductase, Rieske iron-sulfur polypeptide 1 (UQCRFS1), mRNA. | -0,8271 | 11,8626 | | -6,6757 | | 0,000069 | | | 0,00186 |
| ERAL1 | Homo sapiens Era G-protein-like 1 (E. coli) (ERAL1), mRNA. | -0,8272 | 10,1970 | | -4,9578 | | 0,000655 | | | 0,00630 |
| EXOSC2 | Homo sapiens exosome component 2 (EXOSC2), mRNA. | -0,8290 | 8,0173 | | -6,7210 | | 0,000065 | | | 0,00182 |
| NUP43 | Homo sapiens nucleoporin 43kDa (NUP43), transcript variant 2, mRNA. | -0,8292 | 9,0857 | | -3,8053 | | 0,003752 | | | 0,01967 |
| C15orf23 | Homo sapiens chromosome 15 open reading frame 23 (C15orf23), mRNA. | -0,8295 | 8,1518 | | -4,8231 | | 0,000796 | | | 0,00712 |
| APEX1 | Homo sapiens APEX nuclease (multifunctional DNA repair enzyme) 1 (APEX1), transcript variant 1, mRNA. | -0,8297 | 9,9074 | | -5,4640 | | 0,000323 | | | 0,00424 |
| HMGN1 | Homo sapiens high-mobility group nucleosome binding domain 1 (HMGN1), mRNA. | -0,8312 | 12,0000 | | -6,7731 | | 0,000061 | | | 0,00178 |
| HNRNPM | Homo sapiens heterogeneous nuclear ribonucleoprotein M (HNRNPM), transcript variant 1, mRNA. | -0,8313 | 11,3499 | | -5,4008 | | 0,000352 | | | 0,00440 |
| ACTL6A | Homo sapiens actin-like 6A (ACTL6A), transcript variant 2, mRNA. | -0,8318 | 11,2847 | | -5,7368 | | 0,000224 | | | 0,00348 |
| EBPL | Homo sapiens emopamil binding protein-like (EBPL), mRNA. | -0,8326 | 8,8816 | | -3,8688 | | 0,003392 | | | 0,01835 |
| RUVBL2 | Homo sapiens RuvB-like 2 (E. coli) (RUVBL2), mRNA. | -0,8327 | 9,3862 | | -6,3832 | | 0,000098 | | | 0,00221 |
| VIPR1 | Homo sapiens vasoactive intestinal peptide receptor 1 (VIPR1), mRNA. | -0,8334 | 7,9487 | | -4,3646 | | 0,001572 | | | 0,01095 |
| DBNL | Homo sapiens drebrin-like (DBNL), transcript variant 1, mRNA. | -0,8336 | 8,8486 | | -5,7216 | | 0,000229 | | | 0,00352 |
| ORC3L | Homo sapiens origin recognition complex, subunit 3-like (yeast) (ORC3L), transcript variant 1, mRNA. | -0,8339 | 8,7510 | | -3,1739 | | 0,010490 | | | 0,04116 |
| CCT6A | Homo sapiens chaperonin containing TCP1, subunit 6A (zeta 1) (CCT6A), transcript variant 1, mRNA. | -0,8342 | 10,4853 | | -6,3404 | | 0,000103 | | | 0,00227 |
| UBE2G2 | Homo sapiens ubiquitin-conjugating enzyme E2G 2 (UBC7 homolog, yeast) (UBE2G2), transcript variant 1, mRNA. | -0,8343 | 8,9977 | | -3,9267 | | 0,003095 | | | 0,01723 |
| LOC728188 | PREDICTED: Homo sapiens similar to phosphoglycerate mutase processed protein (LOC728188), mRNA. | -0,8344 | 9,4137 | | -9,1410 | | 0,000005 | | | 0,00059 |
| EXOSC3 | Homo sapiens exosome component 3 (EXOSC3), transcript variant 1, mRNA. | -0,8345 | 9,5007 | | -7,8727 | | 0,000018 | | | 0,00101 |
| ALS2CR4 | Homo sapiens amyotrophic lateral sclerosis 2 (juvenile) chromosome region, candidate 4 (ALS2CR4), transcript variant 1, mRNA. | -0,8353 | 9,5610 | | -3,6814 | | 0,004575 | | | 0,02266 |
| SKP1A | Homo sapiens S-phase kinase-associated protein 1A (p19A) (SKP1A), transcript variant 1, mRNA. | -0,8355 | 9,6963 | | -4,8944 | | 0,000718 | | | 0,00665 |
| APEX1 | Homo sapiens APEX nuclease (multifunctional DNA repair enzyme) 1 (APEX1), transcript variant 3, mRNA. | -0,8358 | 10,7311 | | -6,4240 | | 0,000093 | | | 0,00216 |
| PAK1IP1 | Homo sapiens PAK1 interacting protein 1 (PAK1IP1), mRNA. | -0,8363 | 8,8940 | | -6,7741 | | 0,000061 | | | 0,00178 |
| DBI | Homo sapiens diazepam binding inhibitor (GABA receptor modulator, acyl-Coenzyme A binding protein) (DBI), transcript variant 2, mRNA. | -0,8365 | 12,0619 | | -3,9721 | | 0,002882 | | | 0,01649 |
| NDUFAB1 | Homo sapiens NADH dehydrogenase (ubiquinone) 1, alpha/beta subcomplex, 1, 8kDa (NDUFAB1), mRNA. | -0,8366 | 10,9130 | | -5,1454 | | 0,000502 | | | 0,00542 |
| C16orf35 | Homo sapiens chromosome 16 open reading frame 35 (C16orf35), transcript variant 2, mRNA. | -0,8378 | 8,9381 | | -4,0027 | | 0,002747 | | | 0,01598 |
| ZDHHC12 | Homo sapiens zinc finger, DHHC-type containing 12 (ZDHHC12), mRNA. | -0,8380 | 9,1095 | | -5,4684 | | 0,000321 | | | 0,00423 |
| SMC2 | Homo sapiens structural maintenance of chromosomes 2 (SMC2), transcript variant 1, mRNA. | -0,8382 | 8,2633 | | -4,2686 | | 0,001819 | | | 0,01206 |
| SYNCRIP | Homo sapiens synaptotagmin binding, cytoplasmic RNA interacting protein (SYNCRIP), mRNA. | -0,8385 | 10,3559 | | -4,7430 | | 0,000894 | | | 0,00770 |
| RBBP7 | Homo sapiens retinoblastoma binding protein 7 (RBBP7), mRNA. | -0,8385 | 8,9348 | | -3,5644 | | 0,005527 | | | 0,02595 |
| C15orf23 | Homo sapiens chromosome 15 open reading frame 23 (C15orf23), mRNA. | -0,8391 | 8,0603 | | -6,3339 | | 0,000104 | | | 0,00227 |
| CTDSPL | Homo sapiens CTD (carboxy-terminal domain, RNA polymerase II, polypeptide A) small phosphatase-like (CTDSPL), transcript variant 2, mRNA. | -0,8403 | 8,3719 | | -4,3910 | | 0,001510 | | | 0,01070 |
| BCL2L12 | Homo sapiens BCL2-like 12 (proline rich) (BCL2L12), transcript variant 3, mRNA. | -0,8404 | 9,4892 | | -6,9582 | | 0,000049 | | | 0,00160 |
| NDUFA8 | Homo sapiens NADH dehydrogenase (ubiquinone) 1 alpha subcomplex, 8, 19kDa (NDUFA8), nuclear gene encoding mitochondrial protein, mRNA. | -0,8409 | 10,8472 | | -5,5874 | | 0,000274 | | | 0,00387 |
| MRPS34 | Homo sapiens mitochondrial ribosomal protein S34 (MRPS34), nuclear gene encoding mitochondrial protein, mRNA. | -0,8413 | 8,3168 | | -6,9665 | | 0,000049 | | | 0,00159 |
| NHP2 | Homo sapiens NHP2 ribonucleoprotein homolog (yeast) (NHP2), transcript variant 2, mRNA. | -0,8415 | 10,4743 | | -3,8802 | | 0,003332 | | | 0,01815 |
| ASH2L | Homo sapiens ash2 (absent, small, or homeotic)-like (Drosophila) (ASH2L), mRNA. | -0,8418 | 8,8220 | | -5,7948 | | 0,000208 | | | 0,00333 |
| NDUFA9 | Homo sapiens NADH dehydrogenase (ubiquinone) 1 alpha subcomplex, 9, 39kDa (NDUFA9), mRNA. | -0,8422 | 9,8845 | | -4,6991 | | 0,000954 | | | 0,00803 |
| RPE | Homo sapiens ribulose-5-phosphate-3-epimerase (RPE), transcript variant 1, mRNA. | -0,8422 | 8,9250 | | -3,9532 | | 0,002969 | | | 0,01678 |
| TNFSF10 | Homo sapiens tumor necrosis factor (ligand) superfamily, member 10 (TNFSF10), mRNA. | -0,8427 | 8,0518 | | -3,8583 | | 0,003449 | | | 0,01857 |
| CCDC86 | Homo sapiens coiled-coil domain containing 86 (CCDC86), mRNA. | -0,8438 | 9,3754 | | -3,0917 | | 0,012026 | | | 0,04566 |
| SLC2A5 | Homo sapiens solute carrier family 2 (facilitated glucose/fructose transporter), member 5 (SLC2A5), mRNA. | -0,8441 | 7,5633 | | -5,5583 | | 0,000285 | | | 0,00396 |
| MRPL35 | Homo sapiens mitochondrial ribosomal protein L35 (MRPL35), nuclear gene encoding mitochondrial protein, transcript variant 1, mRNA. | -0,8454 | 8,4026 | | -6,3416 | | 0,000103 | | | 0,00227 |
| KLHL13 | Homo sapiens kelch-like 13 (Drosophila) (KLHL13), mRNA. | -0,8454 | 7,6486 | | -7,7543 | | 0,000020 | | | 0,00107 |
| CDCA4 | Homo sapiens cell division cycle associated 4 (CDCA4), transcript variant 13, mRNA. | -0,8458 | 8,7630 | | -5,7280 | | 0,000227 | | | 0,00350 |
| CCDC77 | Homo sapiens coiled-coil domain containing 77 (CCDC77), mRNA. | -0,8469 | 8,0984 | | -5,8098 | | 0,000204 | | | 0,00331 |
| BCCIP | Homo sapiens BRCA2 and CDKN1A interacting protein (BCCIP), transcript variant A, mRNA. | -0,8471 | 9,2935 | | -7,6890 | | 0,000022 | | | 0,00112 |
| FN3KRP | Homo sapiens fructosamine-3-kinase-related protein (FN3KRP), mRNA. | -0,8474 | 9,3088 | | -7,3831 | | 0,000030 | | | 0,00127 |
| C14orf143 | Homo sapiens chromosome 14 open reading frame 143 (C14orf143), mRNA. | -0,8480 | 8,3213 | | -4,3024 | | 0,001728 | | | 0,01168 |
| SFRS6 | Homo sapiens splicing factor, arginine/serine-rich 6 (SFRS6), mRNA. | -0,8488 | 10,7384 | | -4,1380 | | 0,002225 | | | 0,01381 |
| GPR177 | Homo sapiens G protein-coupled receptor 177 (GPR177), transcript variant 1, mRNA. | -0,8489 | 9,6146 | | -6,5101 | | 0,000084 | | | 0,00204 |
| PDCD2 | Homo sapiens programmed cell death 2 (PDCD2), transcript variant 2, mRNA. | -0,8492 | 9,0125 | | -7,0161 | | 0,000046 | | | 0,00153 |
| ALDH3A2 | Homo sapiens aldehyde dehydrogenase 3 family, member A2 (ALDH3A2), transcript variant 2, mRNA. | -0,8503 | 8,7310 | | -5,6582 | | 0,000249 | | | 0,00368 |
| TMEM14B | Homo sapiens transmembrane protein 14B (TMEM14B), mRNA. | -0,8504 | 9,6934 | | -5,7280 | | 0,000227 | | | 0,00350 |
| SFXN1 | Homo sapiens sideroflexin 1 (SFXN1), mRNA. | -0,8507 | 8,9700 | | -5,0283 | | 0,000593 | | | 0,00595 |
| NUP107 | Homo sapiens nucleoporin 107kDa (NUP107), mRNA. | -0,8510 | 8,7160 | | -4,9426 | | 0,000670 | | | 0,00635 |
| HPRT1 | Homo sapiens hypoxanthine phosphoribosyltransferase 1 (Lesch-Nyhan syndrome) (HPRT1), mRNA. | -0,8520 | 9,9764 | | -3,0766 | | 0,012332 | | | 0,04651 |
| HSPA14 | Homo sapiens heat shock 70kDa protein 14 (HSPA14), transcript variant 1, mRNA. | -0,8530 | 8,1867 | | -6,8681 | | 0,000055 | | | 0,00169 |
| NCLN | Homo sapiens nicalin homolog (zebrafish) (NCLN), mRNA. | -0,8531 | 8,9857 | | -5,7976 | | 0,000207 | | | 0,00333 |
| CHCHD4 | Homo sapiens coiled-coil-helix-coiled-coil-helix domain containing 4 (CHCHD4), nuclear gene encoding mitochondrial protein, transcript variant 2, mRNA. | -0,8533 | 8,8162 | | -5,0543 | | 0,000571 | | | 0,00584 |
| LPHN1 | Homo sapiens latrophilin 1 (LPHN1), transcript variant 2, mRNA. | -0,8535 | 8,0467 | | -7,1305 | | 0,000040 | | | 0,00145 |
| RRP15 | Homo sapiens ribosomal RNA processing 15 homolog (S. cerevisiae) (RRP15), mRNA. | -0,8536 | 8,9875 | | -4,5439 | | 0,001200 | | | 0,00919 |
| SPRY1 | Homo sapiens sprouty homolog 1, antagonist of FGF signaling (Drosophila) (SPRY1), transcript variant 1, mRNA. | -0,8537 | 7,9072 | | -4,6936 | | 0,000962 | | | 0,00806 |
| API5 | Homo sapiens apoptosis inhibitor 5 (API5), mRNA. | -0,8538 | 9,5034 | | -5,7392 | | 0,000224 | | | 0,00348 |
| C21orf45 | Homo sapiens chromosome 21 open reading frame 45 (C21orf45), mRNA. | -0,8539 | 8,2954 | | -5,6568 | | 0,000249 | | | 0,00368 |
| ATP5F1 | Homo sapiens ATP synthase, H+ transporting, mitochondrial F0 complex, subunit B1 (ATP5F1), nuclear gene encoding mitochondrial protein, mRNA. | -0,8548 | 11,9037 | | -5,7263 | | 0,000227 | | | 0,00351 |
| PRDX6 | Homo sapiens peroxiredoxin 6 (PRDX6), mRNA. | -0,8548 | 10,1509 | | -3,7321 | | 0,004218 | | | 0,02142 |
| NDUFB3 | Homo sapiens NADH dehydrogenase (ubiquinone) 1 beta subcomplex, 3, 12kDa (NDUFB3), mRNA. | -0,8553 | 11,4005 | | -4,4451 | | 0,001392 | | | 0,01014 |
| TMEM160 | Homo sapiens transmembrane protein 160 (TMEM160), mRNA. | -0,8576 | 9,4446 | | -4,9764 | | 0,000638 | | | 0,00620 |
| CCDC56 | Homo sapiens coiled-coil domain containing 56 (CCDC56), mRNA. | -0,8578 | 10,2438 | | -4,1148 | | 0,002306 | | | 0,01414 |
| DECR1 | Homo sapiens 2,4-dienoyl CoA reductase 1, mitochondrial (DECR1), nuclear gene encoding mitochondrial protein, mRNA. | -0,8598 | 9,6216 | | -3,1139 | | 0,011589 | | | 0,04432 |
| SLC25A4 | Homo sapiens solute carrier family 25 (mitochondrial carrier; adenine nucleotide translocator), member 4 (SLC25A4), nuclear gene encoding mitochondrial protein, mRNA. | -0,8599 | 9,8901 | | -5,1999 | | 0,000465 | | | 0,00521 |
| ATP5A1 | Homo sapiens ATP synthase, H+ transporting, mitochondrial F1 complex, alpha subunit 1, cardiac muscle (ATP5A1), nuclear gene encoding mitochondrial protein, transcript variant 2, mRNA. | -0,8606 | 12,3080 | | -5,8493 | | 0,000193 | | | 0,00320 |
| NDUFV2 | Homo sapiens NADH dehydrogenase (ubiquinone) flavoprotein 2, 24kDa (NDUFV2), mRNA. | -0,8609 | 11,8775 | | -6,3785 | | 0,000099 | | | 0,00222 |
| SMC2 | Homo sapiens structural maintenance of chromosomes 2 (SMC2), transcript variant 1, mRNA. | -0,8609 | 8,2643 | | -3,4508 | | 0,006648 | | | 0,02978 |
| HNRPH3 | Homo sapiens heterogeneous nuclear ribonucleoprotein H3 (2H9) (HNRPH3), transcript variant 2H9, mRNA. | -0,8632 | 8,5788 | | -3,1716 | | 0,010530 | | | 0,04126 |
| BCCIP | Homo sapiens BRCA2 and CDKN1A interacting protein (BCCIP), transcript variant B, mRNA. | -0,8633 | 10,2916 | | -5,4706 | | 0,000320 | | | 0,00423 |
| COPS6 | Homo sapiens COP9 constitutive photomorphogenic homolog subunit 6 (Arabidopsis) (COPS6), mRNA. | -0,8634 | 9,2124 | | -5,2226 | | 0,000451 | | | 0,00510 |
| DGCR6 | Homo sapiens DiGeorge syndrome critical region gene 6 (DGCR6), mRNA. | -0,8643 | 9,4627 | | -3,6630 | | 0,004713 | | | 0,02312 |
| LOC100134648 | PREDICTED: Homo sapiens similar to hCG2024106, transcript variant 2 (LOC100134648), mRNA. | -0,8651 | 10,5595 | | -4,0251 | | 0,002652 | | | 0,01561 |
| H2AFY2 | Homo sapiens H2A histone family, member Y2 (H2AFY2), mRNA. | -0,8663 | 8,0385 | | -6,3294 | | 0,000105 | | | 0,00227 |
| DENR | Homo sapiens density-regulated protein (DENR), mRNA. | -0,8669 | 10,5300 | | -3,0489 | | 0,012914 | | | 0,04829 |
| EFEMP1 | Homo sapiens EGF-containing fibulin-like extracellular matrix protein 1 (EFEMP1), transcript variant 1, mRNA. | -0,8679 | 7,8168 | | -3,9573 | | 0,002950 | | | 0,01672 |
| CDC7 | Homo sapiens cell division cycle 7 homolog (S. cerevisiae) (CDC7), mRNA. | -0,8680 | 7,7843 | | -11,3575 | | 0,000001 | | | 0,00029 |
| SLC39A3 | Homo sapiens solute carrier family 39 (zinc transporter), member 3 (SLC39A3), transcript variant 1, mRNA. | -0,8693 | 8,8883 | | -5,3485 | | 0,000379 | | | 0,00458 |
| C16orf59 | Homo sapiens chromosome 16 open reading frame 59 (C16orf59), mRNA. | -0,8700 | 7,7414 | | -9,9180 | | 0,000002 | | | 0,00046 |
| PSMB10 | Homo sapiens proteasome (prosome, macropain) subunit, beta type, 10 (PSMB10), mRNA. | -0,8701 | 9,8777 | | -6,0717 | | 0,000145 | | | 0,00274 |
| FANCD2 | Homo sapiens Fanconi anemia, complementation group D2 (FANCD2), transcript variant 2, mRNA. | -0,8703 | 8,2382 | | -3,3322 | | 0,008076 | | | 0,03422 |
| SULF2 | Homo sapiens sulfatase 2 (SULF2), transcript variant 1, mRNA. | -0,8703 | 8,0626 | | -4,3053 | | 0,001720 | | | 0,01164 |
| NFIX | Homo sapiens nuclear factor I/X (CCAAT-binding transcription factor) (NFIX), mRNA. | -0,8711 | 8,7110 | | -3,9127 | | 0,003165 | | | 0,01750 |
| GOT2 | Homo sapiens glutamic-oxaloacetic transaminase 2, mitochondrial (aspartate aminotransferase 2) (GOT2), nuclear gene encoding mitochondrial protein, mRNA. | -0,8719 | 11,2308 | | -7,3101 | | 0,000033 | | | 0,00130 |
| RPL8 | Homo sapiens ribosomal protein L8 (RPL8), transcript variant 2, mRNA. | -0,8719 | 9,4726 | | -3,8990 | | 0,003234 | | | 0,01777 |
| HNRPM | Homo sapiens heterogeneous nuclear ribonucleoprotein M (HNRPM), transcript variant 1, mRNA. | -0,8719 | 11,7346 | | -6,5469 | | 0,000080 | | | 0,00200 |
| XRCC3 | Homo sapiens X-ray repair complementing defective repair in Chinese hamster cells 3 (XRCC3), transcript variant 3, mRNA. | -0,8720 | 7,9966 | | -5,3810 | | 0,000362 | | | 0,00447 |
| SIP1 | Homo sapiens survival of motor neuron protein interacting protein 1 (SIP1), transcript variant beta, mRNA. | -0,8733 | 8,6711 | | -7,8184 | | 0,000019 | | | 0,00104 |
| TGIF2 | Homo sapiens TGFB-induced factor homeobox 2 (TGIF2), mRNA. | -0,8739 | 7,8910 | | -12,2705 | | 0,000000 | | | 0,00024 |
| TMEM106C | Homo sapiens transmembrane protein 106C (TMEM106C), mRNA. | -0,8747 | 8,4330 | | -3,2601 | | 0,009094 | | | 0,03714 |
| AASDHPPT | Homo sapiens aminoadipate-semialdehyde dehydrogenase-phosphopantetheinyl transferase (AASDHPPT), mRNA. | -0,8749 | 8,6519 | | -5,8109 | | 0,000203 | | | 0,00330 |
| C9orf140 | Homo sapiens chromosome 9 open reading frame 140 (C9orf140), mRNA. | -0,8752 | 7,9702 | | -3,5786 | | 0,005401 | | | 0,02551 |
| LPCAT3 | Homo sapiens lysophosphatidylcholine acyltransferase 3 (LPCAT3), mRNA. | -0,8760 | 8,5219 | | -6,7410 | | 0,000064 | | | 0,00181 |
| PDXP | Homo sapiens pyridoxal (pyridoxine, vitamin B6) phosphatase (PDXP), mRNA. | -0,8765 | 8,7834 | | -5,2341 | | 0,000444 | | | 0,00505 |
| C11orf73 | Homo sapiens chromosome 11 open reading frame 73 (C11orf73), mRNA. | -0,8768 | 8,9500 | | -4,8025 | | 0,000820 | | | 0,00726 |
| SNCA | Homo sapiens synuclein, alpha (non A4 component of amyloid precursor) (SNCA), transcript variant NACP140, mRNA. | -0,8769 | 7,9174 | | -4,7452 | | 0,000891 | | | 0,00769 |
| LOC647340 | PREDICTED: Homo sapiens similar to ATP synthase, H+ transporting, mitochondrial F1 complex, gamma subunit isoform H (heart) precursor (LOC647340), mRNA. | -0,8778 | 11,3465 | | -13,9016 | | 0,000000 | | | 0,00012 |
| MRPS18C | Homo sapiens mitochondrial ribosomal protein S18C (MRPS18C), nuclear gene encoding mitochondrial protein, mRNA. | -0,8782 | 10,2960 | | -5,7010 | | 0,000235 | | | 0,00356 |
| WDR57 | Homo sapiens WD repeat domain 57 (U5 snRNP specific) (WDR57), mRNA. | -0,8797 | 9,0109 | | -11,8482 | | 0,000001 | | | 0,00028 |
| PKM2 | Homo sapiens pyruvate kinase, muscle (PKM2), transcript variant 3, mRNA. | -0,8799 | 11,3392 | | -3,3444 | | 0,007915 | | | 0,03377 |
| SSB | Homo sapiens Sjogren syndrome antigen B (autoantigen La) (SSB), mRNA. | -0,8800 | 9,9286 | | -4,8919 | | 0,000720 | | | 0,00666 |
| PRPF31 | Homo sapiens PRP31 pre-mRNA processing factor 31 homolog (S. cerevisiae) (PRPF31), mRNA. | -0,8807 | 9,3074 | | -7,5913 | | 0,000024 | | | 0,00116 |
| FANCL | Homo sapiens Fanconi anemia, complementation group L (FANCL), mRNA. | -0,8808 | 7,9192 | | -6,6441 | | 0,000071 | | | 0,00190 |
| ALG6 | Homo sapiens asparagine-linked glycosylation 6 homolog (S. cerevisiae, alpha-1,3-glucosyltransferase) (ALG6), mRNA. | -0,8811 | 8,6183 | | -4,5091 | | 0,001264 | | | 0,00949 |
| COQ5 | Homo sapiens coenzyme Q5 homolog, methyltransferase (S. cerevisiae) (COQ5), mRNA. | -0,8818 | 9,5143 | | -3,3455 | | 0,007900 | | | 0,03372 |
| VSNL1 | Homo sapiens visinin-like 1 (VSNL1), mRNA. | -0,8823 | 7,7849 | | -4,7831 | | 0,000843 | | | 0,00742 |
| NHP2 | Homo sapiens NHP2 ribonucleoprotein homolog (yeast) (NHP2), transcript variant 2, mRNA. | -0,8827 | 10,0366 | | -4,8357 | | 0,000781 | | | 0,00705 |
| NDUFB6 | Homo sapiens NADH dehydrogenase (ubiquinone) 1 beta subcomplex, 6, 17kDa (NDUFB6), nuclear gene encoding mitochondrial protein, transcript variant 1, mRNA. | -0,8845 | 9,4511 | | -5,0053 | | 0,000612 | | | 0,00608 |
| WDR34 | Homo sapiens WD repeat domain 34 (WDR34), mRNA. | -0,8845 | 8,3705 | | -5,7502 | | 0,000220 | | | 0,00345 |
| HSPA2 | Homo sapiens heat shock 70kDa protein 2 (HSPA2), mRNA. | -0,8850 | 7,8547 | | -9,4242 | | 0,000004 | | | 0,00052 |
| CCDC14 | Homo sapiens coiled-coil domain containing 14 (CCDC14), mRNA. | -0,8851 | 8,6959 | | -6,3176 | | 0,000106 | | | 0,00229 |
| LOC440043 | PREDICTED: Homo sapiens misc_RNA (LOC440043), miscRNA. | -0,8854 | 12,3715 | | -9,3551 | | 0,000004 | | | 0,00053 |
| WDR77 | Homo sapiens WD repeat domain 77 (WDR77), mRNA. | -0,8854 | 8,1619 | | -6,8667 | | 0,000055 | | | 0,00169 |
| THOC4 | Homo sapiens THO complex 4 (THOC4), mRNA. | -0,8857 | 8,5367 | | -6,3987 | | 0,000096 | | | 0,00218 |
| PSMA3 | Homo sapiens proteasome (prosome, macropain) subunit, alpha type, 3 (PSMA3), transcript variant 2, mRNA. | -0,8860 | 10,3951 | | -8,0250 | | 0,000015 | | | 0,00095 |
| PSMB3 | Homo sapiens proteasome (prosome, macropain) subunit, beta type, 3 (PSMB3), mRNA. | -0,8865 | 11,6976 | | -5,0464 | | 0,000578 | | | 0,00587 |
| KNTC1 | Homo sapiens kinetochore associated 1 (KNTC1), mRNA. | -0,8875 | 8,2105 | | -3,9503 | | 0,002983 | | | 0,01682 |
| OAT | Homo sapiens ornithine aminotransferase (gyrate atrophy) (OAT), nuclear gene encoding mitochondrial protein, mRNA. | -0,8875 | 10,8542 | | -6,2676 | | 0,000113 | | | 0,00238 |
| PSMG1 | Homo sapiens proteasome (prosome, macropain) assembly chaperone 1 (PSMG1), transcript variant 2, mRNA. | -0,8883 | 10,1974 | | -3,7180 | | 0,004314 | | | 0,02174 |
| NHP2 | Homo sapiens NHP2 ribonucleoprotein homolog (yeast) (NHP2), transcript variant 1, mRNA. | -0,8888 | 11,5349 | | -3,6439 | | 0,004860 | | | 0,02364 |
| RNASEH1 | Homo sapiens ribonuclease H1 (RNASEH1), mRNA. | -0,8888 | 9,6450 | | -6,9021 | | 0,000053 | | | 0,00166 |
| JMJD8 | Homo sapiens jumonji domain containing 8 (JMJD8), mRNA. | -0,8894 | 10,3247 | | -6,8416 | | 0,000056 | | | 0,00170 |
| RFX5 | Homo sapiens regulatory factor X, 5 (influences HLA class II expression) (RFX5), transcript variant 2, mRNA. | -0,8899 | 8,5010 | | -5,2872 | | 0,000412 | | | 0,00485 |
| BCLAF1 | Homo sapiens BCL2-associated transcription factor 1 (BCLAF1), transcript variant 2, mRNA. | -0,8923 | 9,2061 | | -6,3359 | | 0,000104 | | | 0,00227 |
| PRELID1 | Homo sapiens PRELI domain containing 1 (PRELID1), mRNA. | -0,8925 | 9,9475 | | -4,8267 | | 0,000792 | | | 0,00710 |
| FAM96A | Homo sapiens family with sequence similarity 96, member A (FAM96A), transcript variant 2, mRNA. | -0,8931 | 8,6789 | | -8,6093 | | 0,000008 | | | 0,00074 |
| C12orf48 | Homo sapiens chromosome 12 open reading frame 48 (C12orf48), mRNA. | -0,8935 | 8,0995 | | -3,3447 | | 0,007912 | | | 0,03376 |
| WDR12 | Homo sapiens WD repeat domain 12 (WDR12), mRNA. | -0,8938 | 8,8825 | | -6,9956 | | 0,000047 | | | 0,00155 |
| WDR51A | Homo sapiens WD repeat domain 51A (WDR51A), mRNA. | -0,8950 | 8,5679 | | -3,4766 | | 0,006374 | | | 0,02885 |
| LOC643856 | PREDICTED: Homo sapiens similar to hCG2026922 (LOC643856), miscRNA. | -0,8952 | 8,3368 | | -5,1263 | | 0,000516 | | | 0,00550 |
| SNRNP40 | Homo sapiens small nuclear ribonucleoprotein 40kDa (U5) (SNRNP40), mRNA. | -0,8952 | 9,7024 | | -6,4262 | | 0,000093 | | | 0,00216 |
| PSMA4 | Homo sapiens proteasome (prosome, macropain) subunit, alpha type, 4 (PSMA4), mRNA. | -0,8971 | 10,8789 | | -9,8452 | | 0,000003 | | | 0,00047 |
| THOC7 | Homo sapiens THO complex 7 homolog (Drosophila) (THOC7), mRNA. | -0,8972 | 10,7419 | | -5,6634 | | 0,000247 | | | 0,00366 |
| CENPM | Homo sapiens centromere protein M (CENPM), transcript variant 1, mRNA. | -0,8973 | 7,8110 | | -6,0681 | | 0,000146 | | | 0,00275 |
| PON2 | Homo sapiens paraoxonase 2 (PON2), transcript variant 1, mRNA. | -0,8979 | 9,5984 | | -3,9276 | | 0,003091 | | | 0,01721 |
| SUV39H1 | Homo sapiens suppressor of variegation 3-9 homolog 1 (Drosophila) (SUV39H1), mRNA. | -0,8982 | 8,3250 | | -5,8784 | | 0,000186 | | | 0,00313 |
| RMI1 | Homo sapiens RMI1, RecQ mediated genome instability 1, homolog (S. cerevisiae) (RMI1), mRNA. | -0,8983 | 8,0903 | | -8,1051 | | 0,000014 | | | 0,00093 |
| DOLK | Homo sapiens dolichol kinase (DOLK), mRNA. | -0,8996 | 8,5333 | | -8,4434 | | 0,000010 | | | 0,00080 |
| PHKB | Homo sapiens phosphorylase kinase, beta (PHKB), transcript variant 2, mRNA. | -0,9001 | 8,7913 | | -3,6242 | | 0,005017 | | | 0,02420 |
| OIP5 | Homo sapiens Opa interacting protein 5 (OIP5), mRNA. | -0,9007 | 7,9229 | | -4,7281 | | 0,000914 | | | 0,00782 |
| SLC25A3 | Homo sapiens solute carrier family 25 (mitochondrial carrier; phosphate carrier), member 3 (SLC25A3), nuclear gene encoding mitochondrial protein, transcript variant 2, mRNA. | -0,9013 | 11,6319 | | -7,0935 | | 0,000042 | | | 0,00148 |
| RAD51C | Homo sapiens RAD51 homolog C (S. cerevisiae) (RAD51C), transcript variant 1, mRNA. | -0,9020 | 7,9927 | | -4,8837 | | 0,000729 | | | 0,00672 |
| CENPA | Homo sapiens centromere protein A (CENPA), transcript variant 2, mRNA. | -0,9030 | 8,0268 | | -3,8662 | | 0,003406 | | | 0,01839 |
| CNPY4 | Homo sapiens canopy 4 homolog (zebrafish) (CNPY4), mRNA. | -0,9033 | 8,0132 | | -7,7191 | | 0,000021 | | | 0,00110 |
| LOC100132992 | PREDICTED: Homo sapiens misc_RNA (LOC100132992), miscRNA. | -0,9045 | 10,2821 | | -9,0396 | | 0,000005 | | | 0,00062 |
| PTGES2 | Homo sapiens prostaglandin E synthase 2 (PTGES2), transcript variant 2, mRNA. | -0,9046 | 9,0386 | | -5,7223 | | 0,000229 | | | 0,00352 |
| CENPK | Homo sapiens centromere protein K (CENPK), mRNA. | -0,9062 | 8,3717 | | -3,2914 | | 0,008637 | | | 0,03585 |
| ATP5A1 | Homo sapiens ATP synthase, H+ transporting, mitochondrial F1 complex, alpha subunit 1, cardiac muscle (ATP5A1), nuclear gene encoding mitochondrial protein, transcript variant 2, mRNA. | -0,9069 | 11,9556 | | -5,9211 | | 0,000176 | | | 0,00305 |
| LOC100130919 | PREDICTED: Homo sapiens hypothetical protein LOC100130919 (LOC100130919), mRNA. | -0,9070 | 10,8630 | | -3,5279 | | 0,005864 | | | 0,02719 |
| AIFM1 | Homo sapiens apoptosis-inducing factor, mitochondrion-associated, 1 (AIFM1), nuclear gene encoding mitochondrial protein, transcript variant 3, mRNA. | -0,9075 | 8,9873 | | -6,8554 | | 0,000056 | | | 0,00169 |
| MRPS27 | Homo sapiens mitochondrial ribosomal protein S27 (MRPS27), nuclear gene encoding mitochondrial protein, mRNA. | -0,9078 | 8,9192 | | -5,0245 | | 0,000596 | | | 0,00597 |
| PLOD2 | Homo sapiens procollagen-lysine, 2-oxoglutarate 5-dioxygenase 2 (PLOD2), transcript variant 2, mRNA. | -0,9081 | 9,7017 | | -3,7469 | | 0,004119 | | | 0,02105 |
| B3GNT1 | Homo sapiens UDP-GlcNAc:betaGal beta-1,3-N-acetylglucosaminyltransferase 1 (B3GNT1), mRNA. | -0,9081 | 7,8941 | | -7,6736 | | 0,000022 | | | 0,00113 |
| ATP1B1 | Homo sapiens ATPase, Na+/K+ transporting, beta 1 polypeptide (ATP1B1), transcript variant 1, mRNA. | -0,9088 | 9,5057 | | -5,3015 | | 0,000404 | | | 0,00479 |
| ATIC | Homo sapiens 5-aminoimidazole-4-carboxamide ribonucleotide formyltransferase/IMP cyclohydrolase (ATIC), mRNA. | -0,9099 | 10,9636 | | -3,6581 | | 0,004750 | | | 0,02324 |
| C3orf21 | Homo sapiens chromosome 3 open reading frame 21 (C3orf21), mRNA. | -0,9101 | 9,7177 | | -7,8340 | | 0,000019 | | | 0,00103 |
| THOC3 | Homo sapiens THO complex 3 (THOC3), mRNA. | -0,9106 | 9,6550 | | -7,1255 | | 0,000041 | | | 0,00145 |
| LRRCC1 | Homo sapiens leucine rich repeat and coiled-coil domain containing 1 (LRRCC1), transcript variant 1, mRNA. | -0,9109 | 8,0939 | | -4,8985 | | 0,000714 | | | 0,00663 |
| SLC38A10 | Homo sapiens solute carrier family 38, member 10 (SLC38A10), transcript variant 2, mRNA. | -0,9110 | 8,4625 | | -4,0000 | | 0,002758 | | | 0,01601 |
| LANCL1 | Homo sapiens LanC lantibiotic synthetase component C-like 1 (bacterial) (LANCL1), mRNA. | -0,9115 | 8,8033 | | -3,3387 | | 0,007989 | | | 0,03394 |
| SRM | Homo sapiens spermidine synthase (SRM), mRNA. | -0,9117 | 9,2510 | | -4,2388 | | 0,001905 | | | 0,01243 |
| PSMB8 | Homo sapiens proteasome (prosome, macropain) subunit, beta type, 8 (large multifunctional peptidase 7) (PSMB8), transcript variant 1, mRNA. | -0,9128 | 8,5363 | | -4,9453 | | 0,000667 | | | 0,00634 |
| RFWD3 | Homo sapiens ring finger and WD repeat domain 3 (RFWD3), mRNA. | -0,9133 | 8,7947 | | -6,7067 | | 0,000066 | | | 0,00183 |
| MRTO4 | Homo sapiens mRNA turnover 4 homolog (S. cerevisiae) (MRTO4), mRNA. | -0,9141 | 8,5045 | | -6,0786 | | 0,000144 | | | 0,00273 |
| LMAN2L | Homo sapiens lectin, mannose-binding 2-like (LMAN2L), mRNA. | -0,9146 | 8,7145 | | -6,4196 | | 0,000094 | | | 0,00216 |
| CCT2 | Homo sapiens chaperonin containing TCP1, subunit 2 (beta) (CCT2), mRNA. | -0,9147 | 10,6811 | | -3,4220 | | 0,006969 | | | 0,03085 |
| GCNT1 | Homo sapiens glucosaminyl (N-acetyl) transferase 1, core 2 (beta-1,6-N-acetylglucosaminyltransferase) (GCNT1), transcript variant 4, mRNA. | -0,9149 | 7,9052 | | -7,7628 | | 0,000020 | | | 0,00107 |
| HMGN1 | Homo sapiens high-mobility group nucleosome binding domain 1 (HMGN1), mRNA. | -0,9151 | 11,2071 | | -8,8438 | | 0,000007 | | | 0,00066 |
| RAD51C | Homo sapiens RAD51 homolog C (S. cerevisiae) (RAD51C), transcript variant 1, mRNA. | -0,9157 | 8,0520 | | -5,9866 | | 0,000162 | | | 0,00291 |
| C11orf82 | Homo sapiens chromosome 11 open reading frame 82 (C11orf82), mRNA. | -0,9165 | 8,1543 | | -3,8301 | | 0,003607 | | | 0,01917 |
| ACADM | Homo sapiens acyl-Coenzyme A dehydrogenase, C-4 to C-12 straight chain (ACADM), nuclear gene encoding mitochondrial protein, mRNA. | -0,9168 | 8,8949 | | -5,1581 | | 0,000493 | | | 0,00538 |
| MRPS9 | Homo sapiens mitochondrial ribosomal protein S9 (MRPS9), nuclear gene encoding mitochondrial protein, mRNA. | -0,9176 | 9,3708 | | -7,1105 | | 0,000041 | | | 0,00146 |
| LOC100130561 | PREDICTED: Homo sapiens similar to high-mobility group (nonhistone chromosomal) protein 1-like 10, transcript variant 2 (LOC100130561), mRNA. | -0,9182 | 10,1986 | | -4,2337 | | 0,001920 | | | 0,01250 |
| NUP35 | Homo sapiens nucleoporin 35kDa (NUP35), mRNA. | -0,9183 | 8,3632 | | -5,9650 | | 0,000166 | | | 0,00295 |
| CTNNAL1 | Homo sapiens catenin (cadherin-associated protein), alpha-like 1 (CTNNAL1), mRNA. | -0,9184 | 11,0002 | | -3,1018 | | 0,011824 | | | 0,04502 |
| XRCC6 | Homo sapiens X-ray repair complementing defective repair in Chinese hamster cells 6 (XRCC6), mRNA. | -0,9188 | 10,4484 | | -4,7035 | | 0,000948 | | | 0,00800 |
| FABP5L2 | PREDICTED: Homo sapiens fatty acid binding protein 5-like 2 (FABP5L2), mRNA. | -0,9198 | 11,4311 | | -3,1897 | | 0,010218 | | | 0,04041 |
| RCC2 | Homo sapiens regulator of chromosome condensation 2 (RCC2), mRNA. | -0,9201 | 10,7931 | | -3,1649 | | 0,010647 | | | 0,04160 |
| LOC100129585 | PREDICTED: Homo sapiens similar to hCG2011544 (LOC100129585), mRNA. | -0,9210 | 8,9913 | | -3,6421 | | 0,004874 | | | 0,02369 |
| BZW2 | Homo sapiens basic leucine zipper and W2 domains 2 (BZW2), mRNA. | -0,9216 | 10,2564 | | -3,7079 | | 0,004385 | | | 0,02198 |
| CSTF3 | Homo sapiens cleavage stimulation factor, 3' pre-RNA, subunit 3, 77kDa (CSTF3), transcript variant 1, mRNA. | -0,9218 | 8,6260 | | -6,2039 | | 0,000123 | | | 0,00247 |
| PRMT1 | Homo sapiens protein arginine methyltransferase 1 (PRMT1), transcript variant 2, mRNA. | -0,9221 | 11,1602 | | -3,2096 | | 0,009886 | | | 0,03944 |
| RUVBL1 | Homo sapiens RuvB-like 1 (E. coli) (RUVBL1), mRNA. | -0,9223 | 8,6665 | | -5,5053 | | 0,000306 | | | 0,00411 |
| C7orf50 | Homo sapiens chromosome 7 open reading frame 50 (C7orf50), mRNA. | -0,9236 | 10,8189 | | -5,1758 | | 0,000481 | | | 0,00531 |
| STIL | Homo sapiens SCL/TAL1 interrupting locus (STIL), transcript variant 2, mRNA. | -0,9243 | 8,4604 | | -3,8980 | | 0,003239 | | | 0,01779 |
| ANXA8L2 | Homo sapiens annexin A8-like 2 (ANXA8L2), mRNA. | -0,9246 | 9,2035 | | -6,3361 | | 0,000104 | | | 0,00227 |
| XRCC6 | Homo sapiens X-ray repair complementing defective repair in Chinese hamster cells 6 (XRCC6), mRNA. | -0,9260 | 9,6880 | | -4,3785 | | 0,001539 | | | 0,01083 |
| FLJ12684 | Homo sapiens hypothetical protein FLJ12684 (FLJ12684), mRNA. XR_001254 | -0,9264 | 8,1022 | | -6,2430 | | 0,000117 | | | 0,00242 |
| CAMK2N2 | Homo sapiens calcium/calmodulin-dependent protein kinase II inhibitor 2 (CAMK2N2), mRNA. | -0,9267 | 8,3805 | | -5,9735 | | 0,000165 | | | 0,00293 |
| NDUFB5 | Homo sapiens NADH dehydrogenase (ubiquinone) 1 beta subcomplex, 5, 16kDa (NDUFB5), nuclear gene encoding mitochondrial protein, mRNA. | -0,9272 | 11,7465 | | -4,1442 | | 0,002204 | | | 0,01373 |
| XRCC6BP1 | Homo sapiens XRCC6 binding protein 1 (XRCC6BP1), mRNA. | -0,9278 | 8,0362 | | -6,9907 | | 0,000047 | | | 0,00156 |
| BRIX1 | Homo sapiens BRX1, biogenesis of ribosomes, homolog (S. cerevisiae) (BRIX1), mRNA. | -0,9283 | 9,2428 | | -5,1765 | | 0,000481 | | | 0,00531 |
| ZC3HC1 | Homo sapiens zinc finger, C3HC-type containing 1 (ZC3HC1), mRNA. | -0,9285 | 8,5438 | | -11,7159 | | 0,000001 | | | 0,00028 |
| FABP5 | Homo sapiens fatty acid binding protein 5 (psoriasis-associated) (FABP5), mRNA. | -0,9285 | 10,2723 | | -3,4241 | | 0,006946 | | | 0,03077 |
| ATP5F1 | Homo sapiens ATP synthase, H+ transporting, mitochondrial F0 complex, subunit B1 (ATP5F1), nuclear gene encoding mitochondrial protein, mRNA. | -0,9296 | 9,7960 | | -5,1327 | | 0,000511 | | | 0,00548 |
| CISD1 | Homo sapiens CDGSH iron sulfur domain 1 (CISD1), mRNA. | -0,9298 | 10,6762 | | -5,7842 | | 0,000211 | | | 0,00335 |
| RFC3 | Homo sapiens replication factor C (activator 1) 3, 38kDa (RFC3), transcript variant 1, mRNA. | -0,9305 | 7,8507 | | -7,5665 | | 0,000025 | | | 0,00118 |
| LYAR | Homo sapiens Ly1 antibody reactive homolog (mouse) (LYAR), mRNA. | -0,9306 | 9,7705 | | -3,8694 | | 0,003389 | | | 0,01834 |
| CCT3 | Homo sapiens chaperonin containing TCP1, subunit 3 (gamma) (CCT3), transcript variant 1, mRNA. | -0,9307 | 10,5665 | | -5,7164 | | 0,000230 | | | 0,00352 |
| H2AFZ | Homo sapiens H2A histone family, member Z (H2AFZ), mRNA. | -0,9307 | 12,6741 | | -4,7389 | | 0,000900 | | | 0,00772 |
| YWHAB | Homo sapiens tyrosine 3-monooxygenase/tryptophan 5-monooxygenase activation protein, beta polypeptide (YWHAB), transcript variant 2, mRNA. | -0,9316 | 11,9207 | | -3,9294 | | 0,003083 | | | 0,01718 |
| SLBP | Homo sapiens stem-loop binding protein (SLBP), mRNA. | -0,9319 | 9,5107 | | -8,7961 | | 0,000007 | | | 0,00068 |
| MRPL35 | Homo sapiens mitochondrial ribosomal protein L35 (MRPL35), nuclear gene encoding mitochondrial protein, transcript variant 2, mRNA. | -0,9323 | 9,8915 | | -8,5057 | | 0,000009 | | | 0,00078 |
| PGK1 | Homo sapiens phosphoglycerate kinase 1 (PGK1), mRNA. | -0,9325 | 9,9207 | | -5,2332 | | 0,000444 | | | 0,00506 |
| C1orf135 | Homo sapiens chromosome 1 open reading frame 135 (C1orf135), mRNA. | -0,9325 | 8,3202 | | -4,5722 | | 0,001151 | | | 0,00898 |
| CNPY2 | Homo sapiens canopy 2 homolog (zebrafish) (CNPY2), mRNA. | -0,9327 | 9,0079 | | -5,9190 | | 0,000177 | | | 0,00305 |
| MOCS2 | Homo sapiens molybdenum cofactor synthesis 2 (MOCS2), transcript variant 1, mRNA. | -0,9335 | 8,4006 | | -5,8580 | | 0,000191 | | | 0,00319 |
| C8orf55 | Homo sapiens chromosome 8 open reading frame 55 (C8orf55), mRNA. | -0,9346 | 8,4477 | | -3,3322 | | 0,008075 | | | 0,03422 |
[truncated: 73,467 more chars]
