# Supplementary material for: Induction of ER and mitochondrial stress by the alkylphosphocholine erufosine in oral squamous cell carcinoma cells
Source: Cell Death Dis. 2018 Feb 20;9(3):296. doi: 10.1038/s41419-018-0342-2 (PMC5833417; doi:10.1038/s41419-018-0342-2)
Supplement: Supplementary file 4 — Supplementary Table 2a [file 41419_2018_342_MOESM4_ESM.docx]

| Table S2a: Positive enrichment of Hallmarks of cancer at IC25 concentration of erufosine | | | | |
| --- | --- | --- | --- | --- |
| **Hallmarks of cancer** | **SIZE** | **Normalized Enrichment Score** | **FDR.q.val** | **TYPE** |
| HALLMARK_TNFA_SIGNALING_VIA_NFKB | 182 | 5,046 | 0 | High_in_IC25 |
| HALLMARK_PROTEIN_SECRETION | 94 | 3,944 | 0 | High_in_IC25 |
| HALLMARK_KRAS_SIGNALING_UP | 146 | 3,867 | 0 | High_in_IC25 |
| HALLMARK_EPITHELIAL_MESENCHYMAL_TRANSITION | 164 | 3,668 | 0 | High_in_IC25 |
| HALLMARK_HYPOXIA | 172 | 3,647 | 0 | High_in_IC25 |
| HALLMARK_APOPTOSIS | 144 | 3,64 | 0 | High_in_IC25 |
| HALLMARK_P53_PATHWAY | 188 | 3,426 | 0 | High_in_IC25 |
| HALLMARK_CHOLESTEROL_HOMEOSTASIS | 69 | 3,181 | 0 | High_in_IC25 |
| HALLMARK_INFLAMMATORY_RESPONSE | 145 | 3,104 | 0 | High_in_IC25 |
| HALLMARK_ANDROGEN_RESPONSE | 92 | 2,958 | 0 | High_in_IC25 |
| HALLMARK_IL2_STAT5_SIGNALING | 165 | 2,773 | 0,0001 | High_in_IC25 |
| HALLMARK_GLYCOLYSIS | 176 | 2,652 | 0,0001 | High_in_IC25 |
| HALLMARK_UNFOLDED_PROTEIN_RESPONSE | 111 | 2,614 | 0,0002 | High_in_IC25 |
| HALLMARK_COMPLEMENT | 153 | 2,583 | 0,0002 | High_in_IC25 |
| HALLMARK_IL6_JAK_STAT3_SIGNALING | 59 | 2,553 | 0,0003 | High_in_IC25 |
| HALLMARK_HEME_METABOLISM | 166 | 2,473 | 0,0004 | High_in_IC25 |
| HALLMARK_TGF_BETA_SIGNALING | 50 | 2,437 | 0,0003 | High_in_IC25 |
| HALLMARK_APICAL_JUNCTION | 163 | 2,318 | 0,001 | High_in_IC25 |
| HALLMARK_COAGULATION | 99 | 2,24 | 0,002 | High_in_IC25 |
| HALLMARK_PI3K_AKT_MTOR_SIGNALING | 92 | 2,121 | 0,003 | High_in_IC25 |
| HALLMARK_UV_RESPONSE_DN | 127 | 2,014 | 0,006 | High_in_IC25 |
| HALLMARK_MYOGENESIS | 143 | 1,824 | 0,02 | High_in_IC25 |
| HALLMARK_E2F_TARGETS | 187 | -9,155 | 0 | Low_in_IC25 |
| HALLMARK_MYC_TARGETS_V1 | 192 | -7,737 | 0 | Low_in_IC25 |
| HALLMARK_OXIDATIVE_PHOSPHORYLATION | 187 | -6,701 | 0 | Low_in_IC25 |
| HALLMARK_G2M_CHECKPOINT | 183 | -6,053 | 0 | Low_in_IC25 |
| HALLMARK_MYC_TARGETS_V2 | 56 | -4,619 | 0 | Low_in_IC25 |
| HALLMARK_INTERFERON_ALPHA_RESPONSE | 86 | -3,523 | 0 | Low_in_IC25 |
| HALLMARK_DNA_REPAIR | 144 | -3,474 | 0 | Low_in_IC25 |
| HALLMARK_FATTY_ACID_METABOLISM | 135 | -3,048 | 0 | Low_in_IC25 |
| HALLMARK_ADIPOGENESIS | 177 | -2,847 | 0,00008 | Low_in_IC25 |
| HALLMARK_MTORC1_SIGNALING | 194 | -2,563 | 0,0003 | Low_in_IC25 |
| HALLMARK_INTERFERON_GAMMA_RESPONSE | 167 | -2,433 | 0,0006 | Low_in_IC25 |
| HALLMARK_ESTROGEN_RESPONSE_LATE | 170 | -2,225 | 0,003 | Low_in_IC25 |
| HALLMARK_KRAS_SIGNALING_DN | 111 | -2,046 | 0,007 | Low_in_IC25 |
| HALLMARK_MITOTIC_SPINDLE | 189 | -1,963 | 0,01 | Low_in_IC25 |
| HALLMARK_PEROXISOME | 90 | -1,946 | 0,01 | Low_in_IC25 |
| HALLMARK_NOTCH_SIGNALING | 30 | -1,934 | 0,01 | Low_in_IC25 |
| HALLMARK_UV_RESPONSE_UP | 139 | -1,891 | 0,01 | Low_in_IC25 |
| HALLMARK_SPERMATOGENESIS | 89 | -1,828 | 0,02 | Low_in_IC25 |
| HALLMARK_REACTIVE_OXIGEN_SPECIES_PATHWAY | 46 | -1,726 | 0,03 | Low_in_IC25 |
| HALLMARK_APICAL_SURFACE | 29 | -1,676 | 0,03 | Low_in_IC25 |
