# Supplementary material for: Induction of ER and mitochondrial stress by the alkylphosphocholine erufosine in oral squamous cell carcinoma cells
Source: Cell Death Dis. 2018 Feb 20;9(3):296. doi: 10.1038/s41419-018-0342-2 (PMC5833417; doi:10.1038/s41419-018-0342-2)
Supplement: Supplementary file 5 — Supplementary Table 2b [file 41419_2018_342_MOESM5_ESM.docx]

Table S2b: Positive enrichment of Hallmarks of cancer at IC50 concentration of erufosine

| **Hallmarks of cancer** | **SIZE** | **Normalized Enrichment Score** | **FDR.q.val** | **TYPE** |
| --- | --- | --- | --- | --- |
| HALLMARK_TNFA_SIGNALING_VIA_NFKB | 182 | 6,789 | 0 | High_in_IC50 |
| HALLMARK_APOPTOSIS | 144 | 4,278 | 0 | High_in_IC50 |
| HALLMARK_KRAS_SIGNALING_UP | 146 | 4,208 | 0 | High_in_IC50 |
| HALLMARK_HYPOXIA | 172 | 3,952 | 0 | High_in_IC50 |
| HALLMARK_P53_PATHWAY | 188 | 3,698 | 0 | High_in_IC50 |
| HALLMARK_PROTEIN_SECRETION | 94 | 3,598 | 0 | High_in_IC50 |
| HALLMARK_INFLAMMATORY_RESPONSE | 145 | 3,28 | 0 | High_in_IC50 |
| HALLMARK_EPITHELIAL_MESENCHYMAL_TRANSITION | 164 | 3,256 | 0 | High_in_IC50 |
| HALLMARK_APICAL_JUNCTION | 163 | 3,193 | 0 | High_in_IC50 |
| HALLMARK_IL2_STAT5_SIGNALING | 165 | 3,16 | 0 | High_in_IC50 |
| HALLMARK_TGF_BETA_SIGNALING | 50 | 2,973 | 0 | High_in_IC50 |
| HALLMARK_UNFOLDED_PROTEIN_RESPONSE | 111 | 2,938 | 0 | High_in_IC50 |
| HALLMARK_HEME_METABOLISM | 166 | 2,889 | 0 | High_in_IC50 |
| HALLMARK_ANDROGEN_RESPONSE | 92 | 2,869 | 0 | High_in_IC50 |
| HALLMARK_CHOLESTEROL_HOMEOSTASIS | 69 | 2,847 | 0,00008 | High_in_IC50 |
| HALLMARK_IL6_JAK_STAT3_SIGNALING | 59 | 2,753 | 0,00008 | High_in_IC50 |
| HALLMARK_COMPLEMENT | 153 | 2,511 | 0,0002 | High_in_IC50 |
| HALLMARK_GLYCOLYSIS | 176 | 2,397 | 0,0005 | High_in_IC50 |
| HALLMARK_UV_RESPONSE_DN | 127 | 2,194 | 0,002 | High_in_IC50 |
| HALLMARK_ESTROGEN_RESPONSE_EARLY | 163 | 2,153 | 0,003 | High_in_IC50 |
| HALLMARK_ALLOGRAFT_REJECTION | 129 | 2,052 | 0,005 | High_in_IC50 |
| HALLMARK_COAGULATION | 99 | 1,934 | 0,01 | High_in_IC50 |
| HALLMARK_MYOGENESIS | 143 | 1,836 | 0,02 | High_in_IC50 |
| HALLMARK_XENOBIOTIC_METABOLISM | 157 | 1,714 | 0,03 | High_in_IC50 |
| HALLMARK_WNT_BETA_CATENIN_SIGNALING | 34 | 1,685 | 0,03 | High_in_IC50 |
| HALLMARK_PI3K_AKT_MTOR_SIGNALING | 92 | 1,651 | 0,04 | High_in_IC50 |
| HALLMARK_E2F_TARGETS | 187 | -10,565 | 0 | Low_in_IC50 |
| HALLMARK_MYC_TARGETS_V1 | 192 | -9,236 | 0 | Low_in_IC50 |
| HALLMARK_OXIDATIVE_PHOSPHORYLATION | 187 | -7,612 | 0 | Low_in_IC50 |
| HALLMARK_G2M_CHECKPOINT | 183 | -7,283 | 0 | Low_in_IC50 |
| HALLMARK_MYC_TARGETS_V2 | 56 | -5,257 | 0 | Low_in_IC50 |
| HALLMARK_FATTY_ACID_METABOLISM | 135 | -3,985 | 0 | Low_in_IC50 |
| HALLMARK_MTORC1_SIGNALING | 194 | -3,82 | 0 | Low_in_IC50 |
| HALLMARK_DNA_REPAIR | 144 | -3,683 | 0 | Low_in_IC50 |
| HALLMARK_ADIPOGENESIS | 177 | -3,674 | 0 | Low_in_IC50 |
| HALLMARK_MITOTIC_SPINDLE | 189 | -2,516 | 0,0004 | Low_in_IC50 |
| HALLMARK_INTERFERON_ALPHA_RESPONSE | 86 | -2,448 | 0,0006 | Low_in_IC50 |
| HALLMARK_SPERMATOGENESIS | 89 | -1,994 | 0,01 | Low_in_IC50 |
| HALLMARK_UV_RESPONSE_UP | 139 | -1,971 | 0,01 | Low_in_IC50 |
| HALLMARK_ESTROGEN_RESPONSE_LATE | 170 | -1,844 | 0,02 | Low_in_IC50 |
| HALLMARK_PEROXISOME | 90 | -1,843 | 0,02 | Low_in_IC50 |
| HALLMARK_INTERFERON_GAMMA_RESPONSE | 167 | -1,749 | 0,03 | Low_in_IC50 |
