# Supplementary material for: Induction of ER and mitochondrial stress by the alkylphosphocholine erufosine in oral squamous cell carcinoma cells
Source: Cell Death Dis. 2018 Feb 20;9(3):296. doi: 10.1038/s41419-018-0342-2 (PMC5833417; doi:10.1038/s41419-018-0342-2)
Supplement: Supplementary file 6 — Supplementary Table 2c [file 41419_2018_342_MOESM6_ESM.docx]

Table S2c: Positive enrichment of Hallmarks of cancer at IC75 concentration of erufosine

| **Hallmarks of cancer** | **SIZE** | **Normalized Enrichment Score** | **FDR.q.val** | **TYPE** |
| --- | --- | --- | --- | --- |
| HALLMARK_TNFA_SIGNALING_VIA_NFKB | 182 | 7,888 | 0 | High_in_IC75 |
| HALLMARK_KRAS_SIGNALING_UP | 146 | 4,22 | 0 | High_in_IC75 |
| HALLMARK_P53_PATHWAY | 188 | 4,139 | 0 | High_in_IC75 |
| HALLMARK_HYPOXIA | 172 | 3,76 | 0 | High_in_IC75 |
| HALLMARK_APOPTOSIS | 144 | 3,614 | 0 | High_in_IC75 |
| HALLMARK_INFLAMMATORY_RESPONSE | 145 | 3,393 | 0 | High_in_IC75 |
| HALLMARK_TGF_BETA_SIGNALING | 50 | 3,081 | 0 | High_in_IC75 |
| HALLMARK_HEME_METABOLISM | 166 | 2,868 | 0,0002 | High_in_IC75 |
| HALLMARK_EPITHELIAL_MESENCHYMAL_TRANSITION | 164 | 2,809 | 0,0001 | High_in_IC75 |
| HALLMARK_IL2_STAT5_SIGNALING | 165 | 2,692 | 0,0001 | High_in_IC75 |
| HALLMARK_PROTEIN_SECRETION | 94 | 2,652 | 0,0001 | High_in_IC75 |
| HALLMARK_ANDROGEN_RESPONSE | 92 | 2,493 | 0,0005 | High_in_IC75 |
| HALLMARK_IL6_JAK_STAT3_SIGNALING | 59 | 2,447 | 0,0005 | High_in_IC75 |
| HALLMARK_APICAL_JUNCTION | 163 | 2,372 | 0,0009 | High_in_IC75 |
| HALLMARK_UNFOLDED_PROTEIN_RESPONSE | 111 | 2,236 | 0,002 | High_in_IC75 |
| HALLMARK_COMPLEMENT | 153 | 2,127 | 0,004 | High_in_IC75 |
| HALLMARK_COAGULATION | 99 | 2,001 | 0,009 | High_in_IC75 |
| HALLMARK_ALLOGRAFT_REJECTION | 129 | 1,897 | 0,02 | High_in_IC75 |
| HALLMARK_UV_RESPONSE_UP | 139 | 1,889 | 0,02 | High_in_IC75 |
| HALLMARK_UV_RESPONSE_DN | 127 | 1,883 | 0,02 | High_in_IC75 |
| HALLMARK_CHOLESTEROL_HOMEOSTASIS | 69 | 1,855 | 0,02 | High_in_IC75 |
| HALLMARK_MYOGENESIS | 143 | 1,852 | 0,02 | High_in_IC75 |
| HALLMARK_HEDGEHOG_SIGNALING | 27 | 1,781 | 0,02 | High_in_IC75 |
| HALLMARK_ESTROGEN_RESPONSE_EARLY | 163 | 1,758 | 0,02 | High_in_IC75 |
| HALLMARK_ESTROGEN_RESPONSE_LATE | 170 | 1,644 | 0,04 | High_in_IC75 |
| HALLMARK_E2F_TARGETS | 187 | -10,034 | 0 | Low_in_IC75 |
| HALLMARK_MYC_TARGETS_V1 | 192 | -9,289 | 0 | Low_in_IC75 |
| HALLMARK_OXIDATIVE_PHOSPHORYLATION | 187 | -7,88 | 0 | Low_in_IC75 |
| HALLMARK_G2M_CHECKPOINT | 183 | -7,051 | 0 | Low_in_IC75 |
| HALLMARK_MYC_TARGETS_V2 | 56 | -4,796 | 0 | Low_in_IC75 |
| HALLMARK_ADIPOGENESIS | 177 | -4,376 | 0 | Low_in_IC75 |
| HALLMARK_DNA_REPAIR | 144 | -4,294 | 0 | Low_in_IC75 |
| HALLMARK_FATTY_ACID_METABOLISM | 135 | -4,282 | 0 | Low_in_IC75 |
| HALLMARK_MTORC1_SIGNALING | 194 | -4,115 | 0 | Low_in_IC75 |
| HALLMARK_GLYCOLYSIS | 176 | -2,361 | 0,001 | Low_in_IC75 |
| HALLMARK_PEROXISOME | 90 | -2,31 | 0,002 | Low_in_IC75 |
| HALLMARK_MITOTIC_SPINDLE | 189 | -2,239 | 0,003 | Low_in_IC75 |
| HALLMARK_BILE_ACID_METABOLISM | 85 | -2,185 | 0,003 | Low_in_IC75 |
| HALLMARK_SPERMATOGENESIS | 89 | -2,159 | 0,003 | Low_in_IC75 |
| HALLMARK_KRAS_SIGNALING_DN | 111 | -1,971 | 0,008 | Low_in_IC75 |
| HALLMARK_INTERFERON_ALPHA_RESPONSE | 86 | -1,771 | 0,03 | Low_in_IC75 |
| HALLMARK_XENOBIOTIC_METABOLISM | 157 | -1,769 | 0,02 | Low_in_IC75 |
| HALLMARK_NOTCH_SIGNALING | 30 | -1,651 | 0,04 | Low_in_IC75 |
