# Supplementary material for: Induction of ER and mitochondrial stress by the alkylphosphocholine erufosine in oral squamous cell carcinoma cells
Source: Cell Death Dis. 2018 Feb 20;9(3):296. doi: 10.1038/s41419-018-0342-2 (PMC5833417; doi:10.1038/s41419-018-0342-2)
Supplement: Supplementary file 7 — Supplementary Table 3a [file 41419_2018_342_MOESM7_ESM.docx]

Table S3a: Differential regulation of ER stress related genes upon IC25 exposure of erufosine in HN-5 cells

| **Symbol** | **Definition** | **Log Fold Change** | **Average Expression** | **t-statistics** | **P.Value** | **adj.P.Val** |
| --- | --- | --- | --- | --- | --- | --- |
| SELS | Homo sapiens selenoprotein S (SELS), transcript variant 2, mRNA. | 0,95053 | 10,87473 | 6,30980 | 0,0001075 | 0,0368394 |
| GFPT1 | Homo sapiens glutamine-fructose-6-phosphate transaminase 1 (GFPT1), mRNA. | 0,62844 | 9,08266 | 6,48513 | 0,0000866 | 0,0338695 |
| GFPT1 | Homo sapiens glutamine-fructose-6-phosphate transaminase 1 (GFPT1), mRNA. | 0,51541 | 9,30569 | 6,10964 | 0,0001383 | 0,0417472 |
