# Supplementary material for: Induction of ER and mitochondrial stress by the alkylphosphocholine erufosine in oral squamous cell carcinoma cells
Source: Cell Death Dis. 2018 Feb 20;9(3):296. doi: 10.1038/s41419-018-0342-2 (PMC5833417; doi:10.1038/s41419-018-0342-2)
Supplement: Supplementary file 8 — Supplementary Table 3b [file 41419_2018_342_MOESM8_ESM.docx]

Table S3b: Differential regulation of ER stress related genes upon IC50 exposure of erufosine in HN-5 cells

| **Symbol** | **Definition** | **Log Fold Change** | **Average Expression** | **t-statistics** | **P.Value** | **adj.P.Val** |
| --- | --- | --- | --- | --- | --- | --- |
| TRIB3 | Homo sapiens tribbles homolog 3 (Drosophila) (TRIB3), mRNA. | 2,56496 | 9,95780 | 6,86224 | 5,51E-05 | 4,75E-03 |
| PPP1R15A | Homo sapiens protein phosphatase 1, regulatory (inhibitor) subunit 15A (PPP1R15A), mRNA. | 2,35403 | 10,97953 | 7,73701 | 2,05E-05 | 3,26E-03 |
| ASNS | Homo sapiens asparagine synthetase (ASNS), transcript variant 1, mRNA. | 1,80628 | 11,28671 | 4,09918 | 2,36E-03 | 2,94E-02 |
| ASNS | Homo sapiens asparagine synthetase (ASNS), transcript variant 1, mRNA. | 1,65656 | 9,47989 | 4,75254 | 8,82E-04 | 1,72E-02 |
| SELS | Homo sapiens selenoprotein S (SELS), transcript variant 2, mRNA. | 1,57801 | 10,87473 | 10,47515 | 1,51E-06 | 1,12E-03 |
| WIPI1 | Homo sapiens WD repeat domain, phosphoinositide interacting 1 (WIPI1), mRNA. | 1,53398 | 8,74354 | 8,90456 | 6,23E-06 | 1,98E-03 |
| CEBPB | Homo sapiens CCAAT/enhancer binding protein (C/EBP), beta (CEBPB), mRNA. | 1,50109 | 12,10256 | 7,74330 | 2,04E-05 | 3,25E-03 |
| JUN | Homo sapiens jun oncogene (JUN), mRNA. | 1,45151 | 10,79761 | 7,10243 | 4,16E-05 | 4,22E-03 |
| SELS | Homo sapiens selenoprotein S (SELS), transcript variant 2, mRNA. | 1,37661 | 9,46883 | 9,26828 | 4,41E-06 | 1,88E-03 |
| HERPUD1 | Homo sapiens homocysteine-inducible, endoplasmic reticulum stress-inducible, ubiquitin-like domain member 1 (HERPUD1), transcript variant 3, mRNA. | 1,36312 | 9,74352 | 7,43787 | 2,85E-05 | 3,68E-03 |
| HERPUD1 | Homo sapiens homocysteine-inducible, endoplasmic reticulum stress-inducible, ubiquitin-like domain member 1 (HERPUD1), transcript variant 3, mRNA. | 1,34188 | 9,85245 | 7,60528 | 2,37E-05 | 3,41E-03 |
| IL8 | Homo sapiens interleukin 8 (IL8), mRNA. | 1,21233 | 8,67154 | 6,24053 | 1,17E-04 | 6,68E-03 |
| GFPT1 | Homo sapiens glutamine-fructose-6-phosphate transaminase 1 (GFPT1), mRNA. | 1,17045 | 9,08266 | 12,07828 | 4,22E-07 | 5,94E-04 |
| GSK3B | Homo sapiens glycogen synthase kinase 3 beta (GSK3B), mRNA. | 1,14957 | 9,07713 | 4,11273 | 2,31E-03 | 2,90E-02 |
| GFPT1 | Homo sapiens glutamine-fructose-6-phosphate transaminase 1 (GFPT1), mRNA. | 1,13260 | 9,30569 | 13,42574 | 1,62E-07 | 5,64E-04 |
| DNAJB2 | Homo sapiens DnaJ (Hsp40) homolog, subfamily B, member 2 (DNAJB2), transcript variant 2, mRNA. | 1,09707 | 10,65620 | 4,38482 | 1,52E-03 | 2,31E-02 |
| KDELR3 | Homo sapiens KDEL (Lys-Asp-Glu-Leu) endoplasmic reticulum protein retention receptor 3 (KDELR3), transcript variant 2, mRNA. | 1,06585 | 8,93893 | 5,07936 | 5,51E-04 | 1,35E-02 |
| XBP1 | Homo sapiens X-box binding protein 1 (XBP1), transcript variant 1, mRNA. | 1,05119 | 10,85507 | 6,71209 | 6,58E-05 | 4,94E-03 |
| STC2 | Homo sapiens stanniocalcin 2 (STC2), mRNA. | 1,04724 | 8,97259 | 6,04444 | 1,50E-04 | 7,28E-03 |
| XBP1 | Homo sapiens X-box binding protein 1 (XBP1), transcript variant 2, mRNA. | 1,02050 | 10,79362 | 5,35889 | 3,73E-04 | 1,12E-02 |
| KDELR3 | Homo sapiens KDEL (Lys-Asp-Glu-Leu) endoplasmic reticulum protein retention receptor 3 (KDELR3), transcript variant 1, mRNA. | 1,01033 | 8,69308 | 7,21903 | 3,64E-05 | 4,09E-03 |
| DNAJB9 | Homo sapiens DnaJ (Hsp40) homolog, subfamily B, member 9 (DNAJB9), mRNA. | 0,87208 | 8,19112 | 19,06914 | 6,33E-09 | 1,61E-04 |
| C19orf10 | Homo sapiens chromosome 19 open reading frame 10 (C19orf10), mRNA. | 0,83972 | 9,96635 | 4,16856 | 2,12E-03 | 2,75E-02 |
| SEC31A | Homo sapiens SEC31 homolog A (S. cerevisiae) (SEC31A), transcript variant 1, mRNA. | 0,83777 | 9,70885 | 6,89538 | 5,30E-05 | 4,63E-03 |
| EDEM1 | Homo sapiens ER degradation enhancer, mannosidase alpha-like 1 (EDEM1), mRNA. | 0,82308 | 8,63175 | 6,02825 | 1,53E-04 | 7,32E-03 |
| ATF3 | Homo sapiens activating transcription factor 3 (ATF3), transcript variant 4, mRNA. | 0,79794 | 8,58906 | 5,38991 | 3,58E-04 | 1,10E-02 |
| YIF1A | Homo sapiens Yip1 interacting factor homolog A (S. cerevisiae) (YIF1A), mRNA. | 0,79761 | 10,36793 | 4,61538 | 1,08E-03 | 1,92E-02 |
| DDIT3 | Homo sapiens DNA-damage-inducible transcript 3 (DDIT3), mRNA. | 0,76275 | 8,13181 | 5,45107 | 3,29E-04 | 1,05E-02 |
| ATF4 | Homo sapiens activating transcription factor 4 (tax-responsive enhancer element B67) (ATF4), transcript variant 2, mRNA. | 0,74003 | 9,15814 | 3,94350 | 3,01E-03 | 3,38E-02 |
| SEC31A | Homo sapiens SEC31 homolog A (S. cerevisiae) (SEC31A), transcript variant 1, mRNA. | 0,73945 | 8,86463 | 5,63317 | 2,57E-04 | 9,33E-03 |
| ATF4 | Homo sapiens activating transcription factor 4 (tax-responsive enhancer element B67) (ATF4), transcript variant 1, mRNA. | 0,71098 | 12,56939 | 4,40965 | 1,47E-03 | 2,26E-02 |
| PDIA5 | Homo sapiens protein disulfide isomerase family A, member 5 (PDIA5), mRNA. | 0,70864 | 8,85485 | 4,34547 | 1,62E-03 | 2,39E-02 |
| SERP1 | Homo sapiens stress-associated endoplasmic reticulum protein 1 (SERP1), mRNA. | 0,70724 | 9,08245 | 4,65457 | 1,02E-03 | 1,84E-02 |
| CREB3L2 | Homo sapiens cAMP responsive element binding protein 3-like 2 (CREB3L2), mRNA. | 0,62430 | 9,08561 | 4,30812 | 1,71E-03 | 2,46E-02 |
| GOSR2 | Homo sapiens golgi SNAP receptor complex member 2 (GOSR2), transcript variant A, mRNA. | 0,60634 | 8,90755 | 3,62362 | 5,02E-03 | 4,59E-02 |
| IL8 | Homo sapiens interleukin 8 (IL8), mRNA. | 0,60057 | 8,13344 | 4,76605 | 8,65E-04 | 1,71E-02 |
| UFM1 | Homo sapiens ubiquitin-fold modifier 1 (UFM1), mRNA. | 0,59906 | 9,27631 | 3,91950 | 3,13E-03 | 3,45E-02 |
| SYVN1 | Homo sapiens synovial apoptosis inhibitor 1, synoviolin (SYVN1), transcript variant 1, mRNA. | 0,59041 | 8,72269 | 4,32578 | 1,67E-03 | 2,43E-02 |
| HM13 | Homo sapiens histocompatibility (minor) 13 (HM13), transcript variant 2, mRNA. | 0,57037 | 8,61247 | 6,79660 | 5,95E-05 | 4,79E-03 |
| NCK2 | Homo sapiens NCK adaptor protein 2 (NCK2), transcript variant 2, mRNA. | 0,55709 | 9,61059 | 7,76273 | 2,00E-05 | 3,25E-03 |
| TNFRSF10B | Homo sapiens tumor necrosis factor receptor superfamily, member 10b (TNFRSF10B), transcript variant 1, mRNA. | 0,53794 | 9,65035 | 3,80305 | 3,77E-03 | 3,86E-02 |
| ARFGAP1 | Homo sapiens ADP-ribosylation factor GTPase activating protein 1 (ARFGAP1), transcript variant 1, mRNA. | 0,53430 | 9,16085 | 3,95789 | 2,95E-03 | 3,35E-02 |
| NRBF2 | Homo sapiens nuclear receptor binding factor 2 (NRBF2), mRNA. | 0,53105 | 8,17743 | 4,38682 | 1,52E-03 | 2,31E-02 |
| USP13 | Homo sapiens ubiquitin specific peptidase 13 (isopeptidase T-3) (USP13), mRNA. | -0,53380 | 7,96105 | -4,63255 | 1,05E-03 | 1,89E-02 |
| AIFM1 | Homo sapiens apoptosis-inducing factor, mitochondrion-associated, 1 (AIFM1), nuclear gene encoding mitochondrial protein, transcript variant 3, mRNA. | -0,60911 | 8,98728 | -4,60107 | 1,10E-03 | 1,94E-02 |
| KLHDC3 | Homo sapiens kelch domain containing 3 (KLHDC3), mRNA. | -0,65844 | 9,54778 | -4,45017 | 1,38E-03 | 2,18E-02 |
