# Supplementary material for: Induction of ER and mitochondrial stress by the alkylphosphocholine erufosine in oral squamous cell carcinoma cells
Source: Cell Death Dis. 2018 Feb 20;9(3):296. doi: 10.1038/s41419-018-0342-2 (PMC5833417; doi:10.1038/s41419-018-0342-2)
Supplement: Supplementary file 9 — Supplementary Table 3c [file 41419_2018_342_MOESM9_ESM.docx]

Table S3c: Differential regulation of ER stress related genes upon IC75 exposure of erufosine in HN-5 cells

| **Symbol** | **Definition** | **Log Fold Change** | **Average Expression** | **t-statistics** | **P.Value** | **adj.P.Val** |
| --- | --- | --- | --- | --- | --- | --- |
| PPP1R15A | Homo sapiens protein phosphatase 1, regulatory (inhibitor) subunit 15A (PPP1R15A), mRNA. | 3,03216 | 10,97953 | 9,96583 | 2,33862E-06 | 0,00045407 |
| IL8 | Homo sapiens interleukin 8 (IL8), mRNA. | 3,00271 | 8,67154 | 15,45662 | 4,44446E-08 | 0,00012055 |
| ATF3 | Homo sapiens activating transcription factor 3 (ATF3), transcript variant 4, mRNA. | 2,74294 | 8,58906 | 18,52805 | 8,281E-09 | 7,330E-05 |
| TRIB3 | Homo sapiens tribbles homolog 3 (Drosophila) (TRIB3), mRNA. | 2,37294 | 9,95780 | 6,34849 | 1,024E-04 | 2,263E-03 |
| JUN | Homo sapiens jun oncogene (JUN), mRNA. | 2,24307 | 10,79761 | 10,97564 | 9,953E-07 | 3,085E-04 |
| IL8 | Homo sapiens interleukin 8 (IL8), mRNA. | 1,94885 | 8,13344 | 15,46580 | 4,420E-08 | 1,205E-04 |
| PMAIP1 | Homo sapiens phorbol-12-myristate-13-acetate-induced protein 1 (PMAIP1), mRNA. | 1,90872 | 8,58437 | 5,55270 | 2,867E-04 | 3,980E-03 |
| CEBPB | Homo sapiens CCAAT/enhancer binding protein (C/EBP), beta (CEBPB), mRNA. | 1,83666 | 12,10256 | 9,47436 | 3,640E-06 | 5,236E-04 |
| HERPUD1 | Homo sapiens homocysteine-inducible, endoplasmic reticulum stress-inducible, ubiquitin-like domain member 1 (HERPUD1), transcript variant 3, mRNA. | 1,74711 | 9,74352 | 9,53316 | 3,449E-06 | 5,136E-04 |
| WIPI1 | Homo sapiens WD repeat domain, phosphoinositide interacting 1 (WIPI1), mRNA. | 1,73053 | 8,74354 | 10,04553 | 2,180E-06 | 4,354E-04 |
| HERPUD1 | Homo sapiens homocysteine-inducible, endoplasmic reticulum stress-inducible, ubiquitin-like domain member 1 (HERPUD1), transcript variant 3, mRNA. | 1,70913 | 9,85245 | 9,68670 | 3,000E-06 | 4,929E-04 |
| SELS | Homo sapiens selenoprotein S (SELS), transcript variant 2, mRNA. | 1,63125 | 9,46883 | 10,98267 | 9,896E-07 | 3,085E-04 |
| SELS | Homo sapiens selenoprotein S (SELS), transcript variant 2, mRNA. | 1,57957 | 10,87473 | 10,48548 | 1,494E-06 | 3,788E-04 |
| FKBP14 | Homo sapiens FK506 binding protein 14, 22 kDa (FKBP14), mRNA. | 1,48669 | 9,45971 | 5,25433 | 4,313E-04 | 4,971E-03 |
| GSK3B | Homo sapiens glycogen synthase kinase 3 beta (GSK3B), mRNA. | 1,46200 | 9,07713 | 5,23045 | 4,459E-04 | 5,066E-03 |
| ASNS | Homo sapiens asparagine synthetase (ASNS), transcript variant 1, mRNA. | 1,45280 | 11,28671 | 3,29700 | 8,557E-03 | 3,563E-02 |
| GFPT1 | Homo sapiens glutamine-fructose-6-phosphate transaminase 1 (GFPT1), mRNA. | 1,35568 | 9,08266 | 13,98968 | 1,111E-07 | 1,224E-04 |
| ATF4 | Homo sapiens activating transcription factor 4 (tax-responsive enhancer element B67) (ATF4), transcript variant 2, mRNA. | 1,33551 | 9,15814 | 7,11674 | 4,096E-05 | 1,459E-03 |
| DNAJB2 | Homo sapiens DnaJ (Hsp40) homolog, subfamily B, member 2 (DNAJB2), transcript variant 2, mRNA. | 1,32168 | 10,65620 | 5,28256 | 4,147E-04 | 4,873E-03 |
| YOD1 | Homo sapiens YOD1 OTU deubiquinating enzyme 1 homolog (S. cerevisiae) (YOD1), mRNA. | 1,25859 | 8,85447 | 4,24395 | 1,890E-03 | 1,237E-02 |
| ASNS | Homo sapiens asparagine synthetase (ASNS), transcript variant 1, mRNA. | 1,25780 | 9,47989 | 3,60854 | 5,145E-03 | 2,461E-02 |
| XBP1 | Homo sapiens X-box binding protein 1 (XBP1), transcript variant 1, mRNA. | 1,23546 | 10,85507 | 7,88873 | 1,746E-05 | 1,006E-03 |
| GFPT1 | Homo sapiens glutamine-fructose-6-phosphate transaminase 1 (GFPT1), mRNA. | 1,20712 | 9,30569 | 14,30903 | 9,032E-08 | 1,205E-04 |
| DDIT3 | Homo sapiens DNA-damage-inducible transcript 3 (DDIT3), mRNA. | 1,16799 | 8,13181 | 8,34717 | 1,083E-05 | 8,344E-04 |
| DNAJB9 | Homo sapiens DnaJ (Hsp40) homolog, subfamily B, member 9 (DNAJB9), mRNA. | 1,16438 | 8,19112 | 25,46062 | 4,203E-10 | 1,066E-05 |
| C5orf41 | Homo sapiens chromosome 5 open reading frame 41 (C5orf41), mRNA. | 1,14260 | 7,77926 | 6,74856 | 6,299E-05 | 1,799E-03 |
| C5orf41 | Homo sapiens chromosome 5 open reading frame 41 (C5orf41), mRNA. | 1,14097 | 7,73681 | 8,37166 | 1,057E-05 | 8,260E-04 |
| XBP1 | Homo sapiens X-box binding protein 1 (XBP1), transcript variant 2, mRNA. | 1,12677 | 10,79362 | 5,91694 | 1,771E-04 | 3,056E-03 |
| EDEM1 | Homo sapiens ER degradation enhancer, mannosidase alpha-like 1 (EDEM1), mRNA. | 1,02231 | 8,63175 | 7,48736 | 2,698E-05 | 1,214E-03 |
| TRIM13 | Homo sapiens tripartite motif-containing 13 (TRIM13), transcript variant 4, mRNA. | 0,98132 | 8,32436 | 6,12609 | 1,354E-04 | 2,631E-03 |
| ATF4 | Homo sapiens activating transcription factor 4 (tax-responsive enhancer element B67) (ATF4), transcript variant 1, mRNA. | 0,96861 | 12,56939 | 6,00747 | 1,576E-04 | 2,856E-03 |
| SEC31A | Homo sapiens SEC31 homolog A (S. cerevisiae) (SEC31A), transcript variant 1, mRNA. | 0,96519 | 9,70885 | 7,94415 | 1,646E-05 | 9,753E-04 |
| SYVN1 | Homo sapiens synovial apoptosis inhibitor 1, synoviolin (SYVN1), transcript variant 1, mRNA. | 0,92183 | 8,72269 | 6,75406 | 6,258E-05 | 1,795E-03 |
| STC2 | Homo sapiens stanniocalcin 2 (STC2), mRNA. | 0,91112 | 8,97259 | 5,25883 | 4,286E-04 | 4,956E-03 |
| ERN1 | Homo sapiens endoplasmic reticulum to nucleus signalling 1 (ERN1), transcript variant 2, mRNA. | 0,89880 | 7,94183 | 4,83575 | 7,813E-04 | 7,047E-03 |
| RNF103 | Homo sapiens ring finger protein 103 (RNF103), mRNA. | 0,88329 | 8,74942 | 3,66352 | 4,709E-03 | 2,311E-02 |
| SEC31A | Homo sapiens SEC31 homolog A (S. cerevisiae) (SEC31A), transcript variant 1, mRNA. | 0,86317 | 8,86463 | 6,57572 | 7,753E-05 | 1,976E-03 |
| C19orf10 | Homo sapiens chromosome 19 open reading frame 10 (C19orf10), mRNA. | 0,84227 | 9,96635 | 4,18123 | 2,081E-03 | 1,321E-02 |
| ACADVL | Homo sapiens acyl-Coenzyme A dehydrogenase, very long chain (ACADVL), nuclear gene encoding mitochondrial protein, transcript variant 2, mRNA. | 0,82605 | 11,05460 | 5,45207 | 3,286E-04 | 4,282E-03 |
| NRBF2 | Homo sapiens nuclear receptor binding factor 2 (NRBF2), mRNA. | 0,81733 | 8,17743 | 6,75174 | 6,275E-05 | 1,796E-03 |
| ARFGAP1 | Homo sapiens ADP-ribosylation factor GTPase activating protein 1 (ARFGAP1), transcript variant 1, mRNA. | 0,81389 | 9,16085 | 6,02900 | 1,533E-04 | 2,813E-03 |
| UFM1 | Homo sapiens ubiquitin-fold modifier 1 (UFM1), mRNA. | 0,78452 | 9,27631 | 5,13294 | 5,111E-04 | 5,476E-03 |
| PMAIP1 | Homo sapiens phorbol-12-myristate-13-acetate-induced protein 1 (PMAIP1), mRNA. | 0,76897 | 7,68132 | 3,83366 | 3,587E-03 | 1,910E-02 |
| SELK | Homo sapiens selenoprotein K (SELK), mRNA. | 0,73107 | 8,81826 | 7,99753 | 1,556E-05 | 9,554E-04 |
| CREB3L2 | Homo sapiens cAMP responsive element binding protein 3-like 2 (CREB3L2), mRNA. | 0,71882 | 9,08561 | 4,96040 | 6,529E-04 | 6,286E-03 |
| NCK2 | Homo sapiens NCK adaptor protein 2 (NCK2), transcript variant 2, mRNA. | 0,68922 | 9,61059 | 9,60384 | 3,234E-06 | 5,039E-04 |
| TLN1 | Homo sapiens talin 1 (TLN1), mRNA. | 0,67470 | 8,32044 | 3,77293 | 3,951E-03 | 2,040E-02 |
| UBQLN1 | Homo sapiens ubiquilin 1 (UBQLN1), transcript variant 2, mRNA. | 0,66896 | 9,55650 | 4,17415 | 2,104E-03 | 1,331E-02 |
| PPP2R5B | Homo sapiens protein phosphatase 2, regulatory subunit B', beta isoform (PPP2R5B), mRNA. | 0,66852 | 7,70041 | 9,69957 | 2,965E-06 | 4,915E-04 |
| KDELR3 | Homo sapiens KDEL (Lys-Asp-Glu-Leu) endoplasmic reticulum protein retention receptor 3 (KDELR3), transcript variant 1, mRNA. | 0,66625 | 8,69308 | 4,76050 | 8,717E-04 | 7,581E-03 |
| AUP1 | Homo sapiens ancient ubiquitous protein 1 (AUP1), mRNA. | 0,66238 | 8,82183 | 3,19188 | 1,018E-02 | 4,029E-02 |
| PDIA5 | Homo sapiens protein disulfide isomerase family A, member 5 (PDIA5), mRNA. | 0,65865 | 8,85485 | 4,03891 | 2,596E-03 | 1,540E-02 |
| PLA2G4B | Homo sapiens phospholipase A2, group IVB (cytosolic) (PLA2G4B), mRNA. | 0,65186 | 8,18455 | 8,88500 | 6,350E-06 | 6,519E-04 |
| HM13 | Homo sapiens histocompatibility (minor) 13 (HM13), transcript variant 2, mRNA. | 0,64214 | 8,61247 | 7,65181 | 2,253E-05 | 1,131E-03 |
| ACADVL | Homo sapiens acyl-Coenzyme A dehydrogenase, very long chain (ACADVL), nuclear gene encoding mitochondrial protein, transcript variant 1, mRNA. | 0,61638 | 8,61197 | 4,74580 | 8,907E-04 | 7,686E-03 |
| PPP2CB | Homo sapiens protein phosphatase 2 (formerly 2A), catalytic subunit, beta isoform (PPP2CB), transcript variant 1, mRNA. | 0,57902 | 9,89591 | 5,57944 | 2,766E-04 | 3,890E-03 |
| ZBTB17 | Homo sapiens zinc finger and BTB domain containing 17 (ZBTB17), mRNA. | 0,55879 | 8,17083 | 6,12566 | 1,355E-04 | 2,631E-03 |
| DERL2 | Homo sapiens Der1-like domain family, member 2 (DERL2), mRNA. | 0,54950 | 8,56371 | 4,45842 | 1,364E-03 | 1,001E-02 |
| UBQLN1 | Homo sapiens ubiquilin 1 (UBQLN1), transcript variant 2, mRNA. | 0,54208 | 8,59062 | 4,21232 | 1,984E-03 | 1,281E-02 |
| EIF2AK3 | Homo sapiens eukaryotic translation initiation factor 2-alpha kinase 3 (EIF2AK3), mRNA. | 0,53691 | 7,77542 | 8,01033 | 1,535E-05 | 9,497E-04 |
| SERP1 | Homo sapiens stress-associated endoplasmic reticulum protein 1 (SERP1), mRNA. | 0,52538 | 9,08245 | 3,45768 | 6,574E-03 | 2,954E-02 |
| ARFGAP1 | Homo sapiens ADP-ribosylation factor GTPase activating protein 1 (ARFGAP1), transcript variant 2, mRNA. | 0,52147 | 7,78413 | 5,71013 | 2,323E-04 | 3,542E-03 |
| DNAJC3 | Homo sapiens DnaJ (Hsp40) homolog, subfamily C, member 3 (DNAJC3), mRNA. | 0,51436 | 7,74047 | 4,01436 | 2,697E-03 | 1,580E-02 |
| USP13 | Homo sapiens ubiquitin specific peptidase 13 (isopeptidase T-3) (USP13), mRNA. | -0,55208 | 7,67014 | -6,71020 | 6,594E-05 | 1,831E-03 |
| PSMC6 | Homo sapiens proteasome (prosome, macropain) 26S subunit, ATPase, 6 (PSMC6), mRNA. | -0,56089 | 10,45053 | -4,20281 | 2,013E-03 | 1,292E-02 |
| PIK3R2 | Homo sapiens phosphoinositide-3-kinase, regulatory subunit 2 (beta) (PIK3R2), mRNA. | -0,57374 | 10,01927 | -3,34167 | 7,951E-03 | 3,384E-02 |
| RNF121 | Homo sapiens ring finger protein 121 (RNF121), transcript variant 1, mRNA. | -0,61211 | 8,57540 | -5,92373 | 1,756E-04 | 3,049E-03 |
| TP53 | Homo sapiens tumor protein p53 (TP53), mRNA. | -0,65633 | 7,80131 | -3,20617 | 9,943E-03 | 3,963E-02 |
| USP13 | Homo sapiens ubiquitin specific peptidase 13 (isopeptidase T-3) (USP13), mRNA. | -0,66831 | 7,96105 | -5,79986 | 2,064E-04 | 3,325E-03 |
| PSMC5 | Homo sapiens proteasome (prosome, macropain) 26S subunit, ATPase, 5 (PSMC5), mRNA. | -0,70413 | 10,97663 | -6,43722 | 9,181E-05 | 2,150E-03 |
| ATP2A2 | Homo sapiens ATPase, Ca++ transporting, cardiac muscle, slow twitch 2 (ATP2A2), transcript variant 2, mRNA. | -0,72496 | 9,32887 | -4,04906 | 2,555E-03 | 1,526E-02 |
| TMX1 | Homo sapiens thioredoxin-related transmembrane protein 1 (TMX1), mRNA. | -0,72523 | 9,52470 | -5,16237 | 4,904E-04 | 5,371E-03 |
| PSMC2 | Homo sapiens proteasome (prosome, macropain) 26S subunit, ATPase, 2 (PSMC2), mRNA. | -0,79837 | 11,95589 | -7,48137 | 2,716E-05 | 1,215E-03 |
| UBE2G2 | Homo sapiens ubiquitin-conjugating enzyme E2G 2 (UBC7 homolog, yeast) (UBE2G2), transcript variant 1, mRNA. | -0,83432 | 8,99773 | -3,92673 | 0,003095458 | 0,017229527 |
| AIFM1 | Homo sapiens apoptosis-inducing factor, mitochondrion-associated, 1 (AIFM1), nuclear gene encoding mitochondrial protein, transcript variant 3, mRNA. | -0,90755 | 8,98728 | -6,85537 | 5,55038E-05 | 0,001689699 |
