# Supplementary material for: Induction of ER and mitochondrial stress by the alkylphosphocholine erufosine in oral squamous cell carcinoma cells
Source: Cell Death Dis. 2018 Feb 20;9(3):296. doi: 10.1038/s41419-018-0342-2 (PMC5833417; doi:10.1038/s41419-018-0342-2)
Supplement: Supplementary file 10 — Supplementary Table 4a [file 41419_2018_342_MOESM10_ESM.docx]

Table S4a: Gene Ontology terms relating to positive enrichment of Biological processes at IC25 concentration of erufosine

| **Biological processes** | **SIZE** | **Normalized Enrichment Score** | **FDR.q.val** | **TYPE** |
| --- | --- | --- | --- | --- |
| GO_CELLULAR_RESPONSE_TO_TOPOLOGICALLY_INCORRECT_PROTEIN | 110 | 4,579 | 0 | High_in_IC25 |
| GO_VACUOLAR_TRANSPORT | 227 | 4,573 | 0 | High_in_IC25 |
| GO_GOLGI_VESICLE_TRANSPORT | 280 | 4,38 | 0 | High_in_IC25 |
| GO_VESICLE_ORGANIZATION | 231 | 4,114 | 0 | High_in_IC25 |
| GO_RESPONSE_TO_ENDOPLASMIC_RETICULUM_STRESS | 209 | 4,019 | 0 | High_in_IC25 |
| GO_ER_TO_GOLGI_VESICLE_MEDIATED_TRANSPORT | 142 | 3,99 | 0 | High_in_IC25 |
| GO_IRE1_MEDIATED_UNFOLDED_PROTEIN_RESPONSE | 56 | 3,937 | 0 | High_in_IC25 |
| GO_ENDOCYTOSIS | 351 | 3,868 | 0 | High_in_IC25 |
| GO_REGULATION_OF_GTPASE_ACTIVITY | 494 | 3,849 | 0 | High_in_IC25 |
| GO_POSITIVE_REGULATION_OF_LOCOMOTION | 312 | 3,834 | 0 | High_in_IC25 |
| GO_RESPONSE_TO_TOPOLOGICALLY_INCORRECT_PROTEIN | 147 | 3,81 | 0 | High_in_IC25 |
| GO_ENDOMEMBRANE_SYSTEM_ORGANIZATION | 393 | 3,748 | 0 | High_in_IC25 |
| GO_MEMBRANE_BUDDING | 98 | 3,717 | 0 | High_in_IC25 |
| GO_NEGATIVE_REGULATION_OF_PHOSPHORUS_METABOLIC_PROCESS | 414 | 3,602 | 0 | High_in_IC25 |
| GO_EXOCYTOSIS | 219 | 3,487 | 0 | High_in_IC25 |
| GO_ACTIN_FILAMENT_BASED_PROCESS | 337 | 3,481 | 0 | High_in_IC25 |
| GO_VACUOLE_ORGANIZATION | 146 | 3,447 | 0 | High_in_IC25 |
| GO_REGULATION_OF_CELL_MORPHOGENESIS | 418 | 3,446 | 0 | High_in_IC25 |
| GO_NEGATIVE_REGULATION_OF_INTRACELLULAR_SIGNAL_TRANSDUCTION | 350 | 3,412 | 0 | High_in_IC25 |
| GO_WOUND_HEALING | 345 | 3,409 | 0 | High_in_IC25 |
| GO_ENDOSOME_ORGANIZATION | 57 | 3,404 | 0 | High_in_IC25 |
| GO_REGULATION_OF_CELL_ADHESION | 462 | 3,401 | 0 | High_in_IC25 |
| GO_REGULATION_OF_MAPK_CASCADE | 481 | 3,397 | 0 | High_in_IC25 |
| GO_NEGATIVE_REGULATION_OF_PHOSPHORYLATION | 331 | 3,36 | 0 | High_in_IC25 |
| GO_REGULATION_OF_ENDOCRINE_PROCESS | 32 | 3,349 | 0 | High_in_IC25 |
| GO_SECRETION | 396 | 3,323 | 0 | High_in_IC25 |
| GO_SINGLE_ORGANISM_MEMBRANE_BUDDING | 61 | 3,316 | 0 | High_in_IC25 |
| GO_CELL_ACTIVATION | 396 | 3,296 | 0 | High_in_IC25 |
| GO_POSITIVE_REGULATION_OF_SECRETION | 253 | 3,293 | 0 | High_in_IC25 |
| GO_SECRETION_BY_CELL | 330 | 3,29 | 0 | High_in_IC25 |
| GO_REGULATION_OF_CELL_PROJECTION_ORGANIZATION | 412 | 3,283 | 0 | High_in_IC25 |
| GO_DIVALENT_INORGANIC_CATION_HOMEOSTASIS | 217 | 3,269 | 0 | High_in_IC25 |
| GO_CELLULAR_HOMEOSTASIS | 459 | 3,194 | 0,00004 | High_in_IC25 |
| GO_RESPONSE_TO_WOUNDING | 408 | 3,192 | 0,00003 | High_in_IC25 |
| GO_POSITIVE_REGULATION_OF_CELL_ADHESION | 262 | 3,179 | 0,00003 | High_in_IC25 |
| GO_POSITIVE_REGULATION_OF_RESPONSE_TO_EXTERNAL_STIMULUS | 194 | 3,178 | 0,00003 | High_in_IC25 |
| GO_VESICLE_TARGETING | 64 | 3,171 | 0,00003 | High_in_IC25 |
| GO_LOCALIZATION_WITHIN_MEMBRANE | 97 | 3,163 | 0,00003 | High_in_IC25 |
| GO_REGULATION_OF_CELL_ACTIVATION | 321 | 3,143 | 0,00003 | High_in_IC25 |
| GO_RESPONSE_TO_OXYGEN_LEVELS | 255 | 3,141 | 0,00003 | High_in_IC25 |
| GO_REGULATION_OF_SECRETION | 463 | 3,127 | 0,00006 | High_in_IC25 |
| GO_REGULATED_EXOCYTOSIS | 152 | 3,126 | 0,00006 | High_in_IC25 |
| GO_VESICLE_COATING | 62 | 3,108 | 0,00006 | High_in_IC25 |
| GO_REGULATION_OF_AUTOPHAGY | 215 | 3,106 | 0,00006 | High_in_IC25 |
| GO_CELLULAR_RESPONSE_TO_OXYGEN_LEVELS | 125 | 3,103 | 0,00005 | High_in_IC25 |
| GO_REGULATION_OF_PROTEIN_SERINE_THREONINE_KINASE_ACTIVITY | 380 | 3,1 | 0,00005 | High_in_IC25 |
| GO_PHAGOCYTOSIS | 128 | 3,094 | 0,00005 | High_in_IC25 |
| GO_REGULATION_OF_BODY_FLUID_LEVELS | 359 | 3,087 | 0,00005 | High_in_IC25 |
| GO_REGULATION_OF_CELLULAR_COMPONENT_SIZE | 259 | 3,081 | 0,00005 | High_in_IC25 |
| GO_ORGANELLE_LOCALIZATION | 342 | 3,079 | 0,00005 | High_in_IC25 |
| GO_NEGATIVE_REGULATION_OF_KINASE_ACTIVITY | 200 | 3,078 | 0,00005 | High_in_IC25 |
| GO_REGULATION_OF_VESICLE_MEDIATED_TRANSPORT | 344 | 3,047 | 0,00007 | High_in_IC25 |
| GO_REGULATION_OF_SMALL_GTPASE_MEDIATED_SIGNAL_TRANSDUCTION | 213 | 3,043 | 0,00007 | High_in_IC25 |
| GO_POSITIVE_REGULATION_OF_PROTEIN_SERINE_THREONINE_KINASE_ACTIVITY | 227 | 3,039 | 0,00009 | High_in_IC25 |
| GO_NUCLEOTIDE_SUGAR_METABOLIC_PROCESS | 29 | 3,025 | 0,0001 | High_in_IC25 |
| GO_SMALL_GTPASE_MEDIATED_SIGNAL_TRANSDUCTION | 297 | 3,012 | 0,0001 | High_in_IC25 |
| GO_POSITIVE_REGULATION_OF_CELL_MORPHOGENESIS_INVOLVED_IN_DIFFERENTIATION | 129 | 3,004 | 0,0001 | High_in_IC25 |
| GO_METAL_ION_TRANSPORT | 348 | 2,996 | 0,0001 | High_in_IC25 |
| GO_REGULATION_OF_CELL_CELL_ADHESION | 265 | 2,992 | 0,0001 | High_in_IC25 |
| GO_REGULATION_OF_PLASMA_MEMBRANE_ORGANIZATION | 63 | 2,99 | 0,0001 | High_in_IC25 |
| GO_VESICLE_LOCALIZATION | 182 | 2,989 | 0,0001 | High_in_IC25 |
| GO_ACTIN_FILAMENT_BUNDLE_ORGANIZATION | 44 | 2,968 | 0,0001 | High_in_IC25 |
| GO_CELLULAR_CHEMICAL_HOMEOSTASIS | 372 | 2,961 | 0,0001 | High_in_IC25 |
| GO_REGULATION_OF_ANATOMICAL_STRUCTURE_SIZE | 341 | 2,96 | 0,0001 | High_in_IC25 |
| GO_REGULATION_OF_ACTIN_FILAMENT_BASED_PROCESS | 238 | 2,956 | 0,0001 | High_in_IC25 |
| GO_POSITIVE_REGULATION_OF_CELL_DEATH | 496 | 2,938 | 0,0001 | High_in_IC25 |
| GO_ACTIN_FILAMENT_ORGANIZATION | 140 | 2,936 | 0,0001 | High_in_IC25 |
| GO_HEMOSTASIS | 222 | 2,934 | 0,0001 | High_in_IC25 |
| GO_POSITIVE_REGULATION_OF_I_KAPPAB_KINASE_NF_KAPPAB_SIGNALING | 148 | 2,913 | 0,0001 | High_in_IC25 |
| GO_REGULATION_OF_RESPONSE_TO_WOUNDING | 276 | 2,904 | 0,0001 | High_in_IC25 |
| GO_POSITIVE_REGULATION_OF_CELL_PROJECTION_ORGANIZATION | 224 | 2,898 | 0,0001 | High_in_IC25 |
| GO_REGULATION_OF_GROWTH | 462 | 2,894 | 0,0001 | High_in_IC25 |
| GO_DIVALENT_INORGANIC_CATION_TRANSPORT | 165 | 2,891 | 0,0001 | High_in_IC25 |
| GO_NUCLEOTIDE_SUGAR_BIOSYNTHETIC_PROCESS | 18 | 2,884 | 0,0001 | High_in_IC25 |
| GO_CELLULAR_RESPONSE_TO_NITROGEN_COMPOUND | 375 | 2,883 | 0,0001 | High_in_IC25 |
| GO_POSITIVE_REGULATION_OF_KINASE_ACTIVITY | 373 | 2,878 | 0,0001 | High_in_IC25 |
| GO_REGULATION_OF_CYTOSOLIC_CALCIUM_ION_CONCENTRATION | 117 | 2,877 | 0,0001 | High_in_IC25 |
| GO_GOLGI_ORGANIZATION | 75 | 2,869 | 0,0001 | High_in_IC25 |
| GO_NEGATIVE_REGULATION_OF_TRANSPORT | 315 | 2,866 | 0,0001 | High_in_IC25 |
| GO_REGULATION_OF_HOMOTYPIC_CELL_CELL_ADHESION | 209 | 2,849 | 0,0002 | High_in_IC25 |
| GO_REGULATION_OF_CELL_MORPHOGENESIS_INVOLVED_IN_DIFFERENTIATION | 251 | 2,845 | 0,0002 | High_in_IC25 |
| GO_REGULATION_OF_RAS_PROTEIN_SIGNAL_TRANSDUCTION | 142 | 2,835 | 0,0002 | High_in_IC25 |
| GO_IMMUNE_EFFECTOR_PROCESS | 326 | 2,822 | 0,0003 | High_in_IC25 |
| GO_RESPONSE_TO_GROWTH_FACTOR | 353 | 2,809 | 0,0003 | High_in_IC25 |
| GO_REGULATION_OF_STRESS_ACTIVATED_PROTEIN_KINASE_SIGNALING_CASCADE | 164 | 2,804 | 0,0003 | High_in_IC25 |
| GO_REGULATION_OF_MAP_KINASE_ACTIVITY | 246 | 2,793 | 0,0004 | High_in_IC25 |
| GO_CELL_JUNCTION_ORGANIZATION | 144 | 2,791 | 0,0004 | High_in_IC25 |
| GO_REGULATION_OF_NEURON_PROJECTION_DEVELOPMENT | 290 | 2,79 | 0,0004 | High_in_IC25 |
| GO_POSITIVE_REGULATION_OF_CHEMOTAXIS | 77 | 2,786 | 0,0004 | High_in_IC25 |
| GO_VASCULAR_ENDOTHELIAL_GROWTH_FACTOR_RECEPTOR_SIGNALING_PATHWAY | 64 | 2,777 | 0,0004 | High_in_IC25 |
| GO_SINGLE_ORGANISM_CELL_ADHESION | 327 | 2,776 | 0,0004 | High_in_IC25 |
| GO_REGULATION_OF_LEUKOCYTE_MIGRATION | 98 | 2,775 | 0,0004 | High_in_IC25 |
| GO_REGULATION_OF_ION_TRANSPORT | 357 | 2,775 | 0,0004 | High_in_IC25 |
| GO_MEMBRANE_DOCKING | 62 | 2,775 | 0,0004 | High_in_IC25 |
| GO_POST_GOLGI_VESICLE_MEDIATED_TRANSPORT | 78 | 2,775 | 0,0004 | High_in_IC25 |
| GO_EPIDERMAL_GROWTH_FACTOR_RECEPTOR_SIGNALING_PATHWAY | 48 | 2,772 | 0,0004 | High_in_IC25 |
| GO_NEURON_PROJECTION_DEVELOPMENT | 384 | 2,771 | 0,0004 | High_in_IC25 |
| GO_FC_GAMMA_RECEPTOR_SIGNALING_PATHWAY | 65 | 2,766 | 0,0004 | High_in_IC25 |
| GO_LEUKOCYTE_DIFFERENTIATION | 210 | 2,765 | 0,0004 | High_in_IC25 |
| GO_MYELOID_LEUKOCYTE_ACTIVATION | 65 | 2,757 | 0,0004 | High_in_IC25 |
| GO_POSITIVE_REGULATION_OF_NEURON_PROJECTION_DEVELOPMENT | 167 | 2,755 | 0,0004 | High_in_IC25 |
| GO_CELL_MORPHOGENESIS_INVOLVED_IN_DIFFERENTIATION | 354 | 2,753 | 0,0004 | High_in_IC25 |
| GO_ACTIVATION_OF_PROTEIN_KINASE_ACTIVITY | 218 | 2,74 | 0,0004 | High_in_IC25 |
| GO_REGULATION_OF_CHEMOTAXIS | 120 | 2,735 | 0,0005 | High_in_IC25 |
| GO_REGULATION_OF_EPITHELIAL_CELL_MIGRATION | 127 | 2,725 | 0,0005 | High_in_IC25 |
| GO_PLATELET_ACTIVATION | 106 | 2,707 | 0,0006 | High_in_IC25 |
| GO_ERBB_SIGNALING_PATHWAY | 70 | 2,699 | 0,0006 | High_in_IC25 |
| GO_CELLULAR_RESPONSE_TO_EXTERNAL_STIMULUS | 214 | 2,697 | 0,0007 | High_in_IC25 |
| GO_REGULATION_OF_RESPONSE_TO_EXTRACELLULAR_STIMULUS | 150 | 2,693 | 0,0007 | High_in_IC25 |
| GO_EPITHELIAL_CELL_DIFFERENTIATION | 351 | 2,687 | 0,0007 | High_in_IC25 |
| GO_LEUKOCYTE_ACTIVATION | 288 | 2,684 | 0,0007 | High_in_IC25 |
| GO_POSITIVE_REGULATION_OF_EPITHELIAL_CELL_MIGRATION | 80 | 2,676 | 0,0007 | High_in_IC25 |
| GO_MYELOID_CELL_DIFFERENTIATION | 148 | 2,675 | 0,0007 | High_in_IC25 |
| GO_NEGATIVE_REGULATION_OF_PROTEIN_MODIFICATION_PROCESS | 497 | 2,673 | 0,0007 | High_in_IC25 |
| GO_REGULATION_OF_JNK_CASCADE | 131 | 2,667 | 0,0008 | High_in_IC25 |
| GO_LEUKOCYTE_MIGRATION | 177 | 2,659 | 0,0008 | High_in_IC25 |
| GO_EPHRIN_RECEPTOR_SIGNALING_PATHWAY | 72 | 2,655 | 0,0008 | High_in_IC25 |
| GO_ESTABLISHMENT_OR_MAINTENANCE_OF_CELL_POLARITY | 114 | 2,654 | 0,0008 | High_in_IC25 |
| GO_VESICLE_DOCKING | 52 | 2,653 | 0,0008 | High_in_IC25 |
| GO_ION_HOMEOSTASIS | 378 | 2,65 | 0,0008 | High_in_IC25 |
| GO_MULTIVESICULAR_BODY_ORGANIZATION | 29 | 2,642 | 0,0009 | High_in_IC25 |
| GO_REGULATION_OF_LEUKOCYTE_DEGRANULATION | 34 | 2,642 | 0,0009 | High_in_IC25 |
| GO_PLATELET_AGGREGATION | 29 | 2,641 | 0,0009 | High_in_IC25 |
| GO_REGULATION_OF_EXOSOMAL_SECRETION | 15 | 2,635 | 0,0009 | High_in_IC25 |
| GO_REGULATION_OF_EXTRINSIC_APOPTOTIC_SIGNALING_PATHWAY_VIA_DEATH_DOMAIN_RECEPTORS | 45 | 2,632 | 0,001 | High_in_IC25 |
| GO_MYELOID_LEUKOCYTE_DIFFERENTIATION | 70 | 2,623 | 0,001 | High_in_IC25 |
| GO_TRANSFORMING_GROWTH_FACTOR_BETA_RECEPTOR_SIGNALING_PATHWAY | 73 | 2,622 | 0,001 | High_in_IC25 |
| GO_G_PROTEIN_COUPLED_RECEPTOR_SIGNALING_PATHWAY | 488 | 2,622 | 0,001 | High_in_IC25 |
| GO_CYTOSOLIC_TRANSPORT | 179 | 2,619 | 0,001 | High_in_IC25 |
| GO_EPITHELIAL_CELL_PROLIFERATION | 67 | 2,607 | 0,001 | High_in_IC25 |
| GO_CALCIUM_ION_TRANSMEMBRANE_TRANSPORT | 94 | 2,605 | 0,001 | High_in_IC25 |
| GO_POSITIVE_REGULATION_OF_CELL_DEVELOPMENT | 336 | 2,602 | 0,001 | High_in_IC25 |
| GO_PROTEIN_EXIT_FROM_ENDOPLASMIC_RETICULUM | 18 | 2,601 | 0,001 | High_in_IC25 |
| GO_IMMUNE_SYSTEM_DEVELOPMENT | 436 | 2,6 | 0,001 | High_in_IC25 |
| GO_STEROL_BIOSYNTHETIC_PROCESS | 34 | 2,594 | 0,001 | High_in_IC25 |
| GO_REGULATION_OF_RHO_PROTEIN_SIGNAL_TRANSDUCTION | 81 | 2,593 | 0,001 | High_in_IC25 |
| GO_INTRINSIC_APOPTOTIC_SIGNALING_PATHWAY_IN_RESPONSE_TO_ENDOPLASMIC_RETICULUM_STRESS | 29 | 2,59 | 0,001 | High_in_IC25 |
| GO_REGULATION_OF_CELL_SUBSTRATE_ADHESION | 132 | 2,585 | 0,001 | High_in_IC25 |
| GO_POSITIVE_REGULATION_OF_AUTOPHAGY | 66 | 2,576 | 0,001 | High_in_IC25 |
| GO_POSITIVE_REGULATION_OF_APOPTOTIC_SIGNALING_PATHWAY | 151 | 2,575 | 0,001 | High_in_IC25 |
| GO_REGULATION_OF_APOPTOTIC_SIGNALING_PATHWAY | 301 | 2,573 | 0,001 | High_in_IC25 |
| GO_RESPONSE_TO_BACTERIUM | 316 | 2,572 | 0,001 | High_in_IC25 |
| GO_TRANSMEMBRANE_RECEPTOR_PROTEIN_TYROSINE_KINASE_SIGNALING_PATHWAY | 391 | 2,57 | 0,001 | High_in_IC25 |
| GO_POSITIVE_REGULATION_OF_MAPK_CASCADE | 337 | 2,567 | 0,001 | High_in_IC25 |
| GO_REGULATION_OF_PEPTIDYL_TYROSINE_PHOSPHORYLATION | 147 | 2,563 | 0,001 | High_in_IC25 |
| GO_REGULATION_OF_SEQUESTERING_OF_CALCIUM_ION | 71 | 2,558 | 0,001 | High_in_IC25 |
| GO_NEGATIVE_REGULATION_OF_CELL_PROLIFERATION | 490 | 2,555 | 0,001 | High_in_IC25 |
| GO_CALCIUM_ION_TRANSPORT | 136 | 2,553 | 0,001 | High_in_IC25 |
| GO_REGULATION_OF_TRANSMEMBRANE_TRANSPORT | 257 | 2,547 | 0,001 | High_in_IC25 |
| GO_REGULATION_OF_CALCIUM_ION_TRANSPORT | 127 | 2,541 | 0,001 | High_in_IC25 |
| GO_TISSUE_MORPHOGENESIS | 390 | 2,54 | 0,001 | High_in_IC25 |
| GO_REGULATION_OF_WOUND_HEALING | 89 | 2,537 | 0,001 | High_in_IC25 |
| GO_EXTRACELLULAR_STRUCTURE_ORGANIZATION | 214 | 2,533 | 0,002 | High_in_IC25 |
| GO_MORPHOGENESIS_OF_AN_EPITHELIUM | 304 | 2,529 | 0,002 | High_in_IC25 |
| GO_CELLULAR_RESPONSE_TO_EXTRACELLULAR_STIMULUS | 153 | 2,528 | 0,002 | High_in_IC25 |
| GO_POSITIVE_REGULATION_OF_STRESS_ACTIVATED_PROTEIN_KINASE_SIGNALING_CASCADE | 114 | 2,527 | 0,002 | High_in_IC25 |
| GO_NEGATIVE_REGULATION_OF_CELL_ADHESION | 170 | 2,527 | 0,002 | High_in_IC25 |
| GO_REGULATION_OF_CALCIUM_ION_TRANSMEMBRANE_TRANSPORT | 77 | 2,52 | 0,002 | High_in_IC25 |
| GO_PROTEIN_LOCALIZATION_TO_MEMBRANE | 322 | 2,52 | 0,002 | High_in_IC25 |
| GO_CELL_CELL_ADHESION | 400 | 2,519 | 0,002 | High_in_IC25 |
| GO_NEGATIVE_REGULATION_OF_HYDROLASE_ACTIVITY | 271 | 2,519 | 0,002 | High_in_IC25 |
| GO_G_PROTEIN_COUPLED_RECEPTOR_SIGNALING_PATHWAY_COUPLED_TO_CYCLIC_NUCLEOTIDE_SECOND_MESSENGER | 88 | 2,517 | 0,002 | High_in_IC25 |
| GO_POSITIVE_REGULATION_OF_CELL_GROWTH | 109 | 2,509 | 0,002 | High_in_IC25 |
| GO_POSITIVE_REGULATION_OF_NEURON_DIFFERENTIATION | 214 | 2,504 | 0,002 | High_in_IC25 |
| GO_POSITIVE_REGULATION_OF_CELL_CELL_ADHESION | 164 | 2,503 | 0,002 | High_in_IC25 |
| GO_DEVELOPMENTAL_CELL_GROWTH | 55 | 2,498 | 0,002 | High_in_IC25 |
| GO_RESPONSE_TO_METAL_ION | 245 | 2,495 | 0,002 | High_in_IC25 |
| GO_POSITIVE_REGULATION_OF_RESPONSE_TO_EXTRACELLULAR_STIMULUS | 42 | 2,493 | 0,002 | High_in_IC25 |
| GO_REGULATION_OF_LEUKOCYTE_CHEMOTAXIS | 64 | 2,482 | 0,002 | High_in_IC25 |
| GO_POSITIVE_REGULATION_OF_GROWTH | 166 | 2,479 | 0,002 | High_in_IC25 |
| GO_ENDOTHELIAL_CELL_DEVELOPMENT | 40 | 2,472 | 0,002 | High_in_IC25 |
| GO_REGULATION_OF_CELLULAR_PROTEIN_LOCALIZATION | 458 | 2,471 | 0,002 | High_in_IC25 |
| GO_REGULATION_OF_INTRACELLULAR_TRANSPORT | 494 | 2,468 | 0,002 | High_in_IC25 |
| GO_NEGATIVE_REGULATION_OF_SMALL_GTPASE_MEDIATED_SIGNAL_TRANSDUCTION | 33 | 2,467 | 0,002 | High_in_IC25 |
| GO_ACTIVATION_OF_MAPK_ACTIVITY | 102 | 2,466 | 0,002 | High_in_IC25 |
| GO_LYSOSOMAL_TRANSPORT | 65 | 2,463 | 0,002 | High_in_IC25 |
| GO_POSITIVE_REGULATION_OF_CELL_SUBSTRATE_ADHESION | 72 | 2,463 | 0,002 | High_in_IC25 |
| GO_POSITIVE_REGULATION_OF_CELL_ACTIVATION | 194 | 2,463 | 0,002 | High_in_IC25 |
| GO_RESPONSE_TO_INORGANIC_SUBSTANCE | 365 | 2,453 | 0,002 | High_in_IC25 |
| GO_PLASMA_MEMBRANE_ORGANIZATION | 164 | 2,453 | 0,002 | High_in_IC25 |
| GO_REGULATION_OF_CELL_GROWTH | 291 | 2,451 | 0,002 | High_in_IC25 |
| GO_PLATELET_DEGRANULATION | 66 | 2,449 | 0,003 | High_in_IC25 |
| GO_VASCULATURE_DEVELOPMENT | 351 | 2,443 | 0,003 | High_in_IC25 |
| GO_REGULATION_OF_PROTEIN_TYROSINE_KINASE_ACTIVITY | 50 | 2,439 | 0,003 | High_in_IC25 |
| GO_REGULATION_OF_EXOCYTOSIS | 131 | 2,436 | 0,003 | High_in_IC25 |
| GO_RAS_PROTEIN_SIGNAL_TRANSDUCTION | 125 | 2,435 | 0,003 | High_in_IC25 |
| GO_REGULATION_OF_CALCIUM_ION_IMPORT | 64 | 2,424 | 0,003 | High_in_IC25 |
| GO_EPITHELIAL_CELL_DEVELOPMENT | 140 | 2,417 | 0,003 | High_in_IC25 |
| GO_REGULATION_OF_ERBB_SIGNALING_PATHWAY | 73 | 2,409 | 0,003 | High_in_IC25 |
| GO_POSITIVE_REGULATION_OF_MAP_KINASE_ACTIVITY | 153 | 2,404 | 0,003 | High_in_IC25 |
| GO_REGULATION_OF_EXTRINSIC_APOPTOTIC_SIGNALING_PATHWAY | 122 | 2,402 | 0,003 | High_in_IC25 |
| GO_POSITIVE_REGULATION_OF_LIPASE_ACTIVITY | 40 | 2,401 | 0,003 | High_in_IC25 |
| GO_RESPONSE_TO_MOLECULE_OF_BACTERIAL_ORIGIN | 226 | 2,4 | 0,003 | High_in_IC25 |
| GO_REGULATION_OF_SYSTEM_PROCESS | 310 | 2,397 | 0,003 | High_in_IC25 |
| GO_REGULATION_OF_VACUOLE_ORGANIZATION | 38 | 2,396 | 0,003 | High_in_IC25 |
| GO_POSITIVE_REGULATION_OF_CELLULAR_COMPONENT_BIOGENESIS | 317 | 2,394 | 0,003 | High_in_IC25 |
| GO_ENDOSOME_TO_LYSOSOME_TRANSPORT | 38 | 2,393 | 0,003 | High_in_IC25 |
| GO_CELLULAR_RESPONSE_TO_PEPTIDE | 217 | 2,389 | 0,004 | High_in_IC25 |
| GO_REGULATION_OF_JUN_KINASE_ACTIVITY | 67 | 2,383 | 0,004 | High_in_IC25 |
| GO_POSITIVE_REGULATION_OF_LEUKOCYTE_CHEMOTAXIS | 53 | 2,381 | 0,004 | High_in_IC25 |
| GO_REGULATION_OF_I_KAPPAB_KINASE_NF_KAPPAB_SIGNALING | 198 | 2,38 | 0,004 | High_in_IC25 |
| GO_POSITIVE_REGULATION_OF_AXON_EXTENSION | 29 | 2,38 | 0,004 | High_in_IC25 |
| GO_REGULATION_OF_NEURON_DIFFERENTIATION | 392 | 2,38 | 0,004 | High_in_IC25 |
| GO_MULTICELLULAR_ORGANISMAL_HOMEOSTASIS | 182 | 2,377 | 0,004 | High_in_IC25 |
| GO_TAXIS | 307 | 2,375 | 0,004 | High_in_IC25 |
| GO_REGULATION_OF_ACTIN_FILAMENT_LENGTH | 125 | 2,373 | 0,004 | High_in_IC25 |
| GO_PROTEIN_CATABOLIC_PROCESS | 484 | 2,368 | 0,004 | High_in_IC25 |
| GO_CELLULAR_RESPONSE_TO_GLUCOSE_STARVATION | 27 | 2,366 | 0,004 | High_in_IC25 |
| GO_ENDOPLASMIC_RETICULUM_ORGANIZATION | 32 | 2,366 | 0,004 | High_in_IC25 |
| GO_ADENYLATE_CYCLASE_MODULATING_G_PROTEIN_COUPLED_RECEPTOR_SIGNALING_PATHWAY | 78 | 2,363 | 0,004 | High_in_IC25 |
| GO_CELL_JUNCTION_ASSEMBLY | 102 | 2,362 | 0,004 | High_in_IC25 |
| GO_POSITIVE_REGULATION_OF_LEUKOCYTE_MIGRATION | 71 | 2,362 | 0,004 | High_in_IC25 |
| GO_REGULATION_OF_COAGULATION | 59 | 2,361 | 0,004 | High_in_IC25 |
| GO_POSITIVE_REGULATION_OF_VASCULATURE_DEVELOPMENT | 97 | 2,35 | 0,004 | High_in_IC25 |
| GO_CARBOHYDRATE_METABOLIC_PROCESS | 482 | 2,348 | 0,004 | High_in_IC25 |
| GO_REGULATION_OF_MYELOID_LEUKOCYTE_DIFFERENTIATION | 78 | 2,347 | 0,004 | High_in_IC25 |
| GO_NEGATIVE_REGULATION_OF_AUTOPHAGY | 42 | 2,343 | 0,005 | High_in_IC25 |
| GO_TISSUE_REMODELING | 61 | 2,342 | 0,005 | High_in_IC25 |
| GO_MYELOID_LEUKOCYTE_MEDIATED_IMMUNITY | 30 | 2,342 | 0,005 | High_in_IC25 |
| GO_TRANSMEMBRANE_RECEPTOR_PROTEIN_SERINE_THREONINE_KINASE_SIGNALING_PATHWAY | 136 | 2,34 | 0,005 | High_in_IC25 |
| GO_REGULATION_OF_CELLULAR_RESPONSE_TO_TRANSFORMING_GROWTH_FACTOR_BETA_STIMULUS | 79 | 2,335 | 0,005 | High_in_IC25 |
| GO_REGULATION_OF_VASCULATURE_DEVELOPMENT | 168 | 2,334 | 0,005 | High_in_IC25 |
| GO_REGULATION_OF_MAST_CELL_ACTIVATION_INVOLVED_IN_IMMUNE_RESPONSE | 25 | 2,327 | 0,005 | High_in_IC25 |
| GO_ORGANELLE_MEMBRANE_FUSION | 75 | 2,326 | 0,005 | High_in_IC25 |
| GO_POSITIVE_REGULATION_OF_PEPTIDYL_SERINE_PHOSPHORYLATION | 65 | 2,325 | 0,005 | High_in_IC25 |
| GO_RESPONSE_TO_EXTRACELLULAR_STIMULUS | 319 | 2,322 | 0,005 | High_in_IC25 |
| GO_REGULATION_OF_CYTOKINE_PRODUCTION | 412 | 2,321 | 0,005 | High_in_IC25 |
| GO_ESTABLISHMENT_OF_CELL_POLARITY | 75 | 2,32 | 0,005 | High_in_IC25 |
| GO_CELLULAR_RESPONSE_TO_HORMONE_STIMULUS | 414 | 2,319 | 0,005 | High_in_IC25 |
| GO_ION_TRANSMEMBRANE_TRANSPORT | 494 | 2,318 | 0,005 | High_in_IC25 |
| GO_NEGATIVE_REGULATION_OF_MAPK_CASCADE | 120 | 2,315 | 0,005 | High_in_IC25 |
| GO_ESTABLISHMENT_OF_PROTEIN_LOCALIZATION_TO_MEMBRANE | 240 | 2,314 | 0,005 | High_in_IC25 |
| GO_REGULATION_OF_METAL_ION_TRANSPORT | 200 | 2,312 | 0,005 | High_in_IC25 |
| GO_GRANULOCYTE_ACTIVATION | 16 | 2,31 | 0,005 | High_in_IC25 |
| GO_HOMOTYPIC_CELL_CELL_ADHESION | 38 | 2,308 | 0,005 | High_in_IC25 |
| GO_CELL_CELL_SIGNALING | 442 | 2,308 | 0,005 | High_in_IC25 |
| GO_REGULATION_OF_INFLAMMATORY_RESPONSE | 191 | 2,305 | 0,005 | High_in_IC25 |
| GO_LEUKOCYTE_DEGRANULATION | 24 | 2,304 | 0,005 | High_in_IC25 |
| GO_STRESS_ACTIVATED_PROTEIN_KINASE_SIGNALING_CASCADE | 85 | 2,301 | 0,005 | High_in_IC25 |
| GO_CELL_ACTIVATION_INVOLVED_IN_IMMUNE_RESPONSE | 93 | 2,299 | 0,005 | High_in_IC25 |
| GO_ANGIOGENESIS | 219 | 2,299 | 0,005 | High_in_IC25 |
| GO_BONE_REMODELING | 26 | 2,299 | 0,005 | High_in_IC25 |
| GO_POSITIVE_REGULATION_OF_FAT_CELL_DIFFERENTIATION | 39 | 2,292 | 0,006 | High_in_IC25 |
| GO_SIGNAL_TRANSDUCTION_BY_PROTEIN_PHOSPHORYLATION | 316 | 2,29 | 0,006 | High_in_IC25 |
| GO_ANION_TRANSPORT | 323 | 2,288 | 0,006 | High_in_IC25 |
| GO_REGULATION_OF_CYTOSKELETON_ORGANIZATION | 408 | 2,287 | 0,006 | High_in_IC25 |
| GO_REGULATION_OF_HORMONE_SECRETION | 167 | 2,285 | 0,006 | High_in_IC25 |
| GO_DENDRITIC_CELL_DIFFERENTIATION | 21 | 2,283 | 0,006 | High_in_IC25 |
| GO_REGULATION_OF_VACUOLAR_TRANSPORT | 27 | 2,282 | 0,006 | High_in_IC25 |
| GO_ORGANELLE_FUSION | 105 | 2,28 | 0,006 | High_in_IC25 |
| GO_NEGATIVE_REGULATION_OF_PEPTIDYL_TYROSINE_PHOSPHORYLATION | 30 | 2,279 | 0,006 | High_in_IC25 |
| GO_REGULATION_OF_CYTOPLASMIC_TRANSPORT | 375 | 2,279 | 0,006 | High_in_IC25 |
| GO_REGULATION_OF_EPIDERMAL_GROWTH_FACTOR_ACTIVATED_RECEPTOR_ACTIVITY | 19 | 2,276 | 0,006 | High_in_IC25 |
| GO_REGULATION_OF_CELL_SHAPE | 106 | 2,274 | 0,006 | High_in_IC25 |
| GO_ESTABLISHMENT_OF_PROTEIN_LOCALIZATION_TO_PLASMA_MEMBRANE | 78 | 2,272 | 0,006 | High_in_IC25 |
| GO_EXTRACELLULAR_MATRIX_DISASSEMBLY | 52 | 2,27 | 0,006 | High_in_IC25 |
| GO_REGULATION_OF_HORMONE_LEVELS | 309 | 2,27 | 0,006 | High_in_IC25 |
| GO_REGULATION_OF_PHOSPHOLIPASE_C_ACTIVITY | 24 | 2,27 | 0,006 | High_in_IC25 |
| GO_REGULATION_OF_RELEASE_OF_SEQUESTERED_CALCIUM_ION_INTO_CYTOSOL | 49 | 2,27 | 0,006 | High_in_IC25 |
| GO_REGULATION_OF_CELL_MATRIX_ADHESION | 72 | 2,267 | 0,006 | High_in_IC25 |
| GO_CELLULAR_RESPONSE_TO_STARVATION | 97 | 2,266 | 0,006 | High_in_IC25 |
| GO_BLOOD_VESSEL_MORPHOGENESIS | 266 | 2,265 | 0,006 | High_in_IC25 |
| GO_RESPONSE_TO_ALCOHOL | 259 | 2,262 | 0,006 | High_in_IC25 |
| GO_MULTI_ORGANISM_ORGANELLE_ORGANIZATION | 23 | 2,26 | 0,006 | High_in_IC25 |
| GO_REGULATION_OF_REGULATED_SECRETORY_PATHWAY | 86 | 2,258 | 0,007 | High_in_IC25 |
| GO_NEGATIVE_REGULATION_OF_GROWTH | 177 | 2,257 | 0,007 | High_in_IC25 |
| GO_REGULATION_OF_ORGANELLE_ASSEMBLY | 126 | 2,255 | 0,007 | High_in_IC25 |
| GO_RETROGRADE_TRANSPORT_ENDOSOME_TO_GOLGI | 66 | 2,252 | 0,007 | High_in_IC25 |
| GO_MULTI_ORGANISM_MEMBRANE_ORGANIZATION | 27 | 2,25 | 0,007 | High_in_IC25 |
| GO_RESPONSE_TO_PEPTIDE | 305 | 2,249 | 0,007 | High_in_IC25 |
| GO_POSITIVE_REGULATION_OF_JUN_KINASE_ACTIVITY | 55 | 2,244 | 0,007 | High_in_IC25 |
| GO_CATION_TRANSPORT | 495 | 2,244 | 0,007 | High_in_IC25 |
| GO_REGULATION_OF_FAT_CELL_DIFFERENTIATION | 80 | 2,243 | 0,007 | High_in_IC25 |
| GO_NEURON_DEVELOPMENT | 480 | 2,237 | 0,007 | High_in_IC25 |
| GO_POSITIVE_REGULATION_OF_RESPONSE_TO_WOUNDING | 98 | 2,234 | 0,007 | High_in_IC25 |
| GO_REGULATION_OF_CYTOKINE_BIOSYNTHETIC_PROCESS | 61 | 2,23 | 0,008 | High_in_IC25 |
| GO_POSITIVE_REGULATION_OF_CELL_MATRIX_ADHESION | 31 | 2,228 | 0,008 | High_in_IC25 |
| GO_POSITIVE_REGULATION_OF_IMMUNE_RESPONSE | 383 | 2,224 | 0,008 | High_in_IC25 |
| GO_RESPONSE_TO_FLUID_SHEAR_STRESS | 28 | 2,224 | 0,008 | High_in_IC25 |
| GO_REGULATION_OF_PROTEIN_KINASE_B_SIGNALING | 95 | 2,223 | 0,008 | High_in_IC25 |
| GO_RESPONSE_TO_STARVATION | 121 | 2,22 | 0,008 | High_in_IC25 |
| GO_REGULATION_OF_CATION_TRANSMEMBRANE_TRANSPORT | 131 | 2,219 | 0,008 | High_in_IC25 |
| GO_POSITIVE_REGULATION_OF_PHOSPHOLIPASE_ACTIVITY | 34 | 2,218 | 0,008 | High_in_IC25 |
| GO_REGULATION_OF_TUMOR_NECROSIS_FACTOR_SUPERFAMILY_CYTOKINE_PRODUCTION | 71 | 2,217 | 0,008 | High_in_IC25 |
| GO_EPIBOLY | 20 | 2,213 | 0,008 | High_in_IC25 |
| GO_ERAD_PATHWAY | 65 | 2,21 | 0,008 | High_in_IC25 |
| GO_NEGATIVE_REGULATION_OF_CATABOLIC_PROCESS | 160 | 2,209 | 0,008 | High_in_IC25 |
| GO_REGULATION_OF_LEUKOCYTE_MEDIATED_IMMUNITY | 103 | 2,201 | 0,009 | High_in_IC25 |
| GO_NEGATIVE_REGULATION_OF_CELL_CELL_ADHESION | 100 | 2,201 | 0,009 | High_in_IC25 |
| GO_DEPHOSPHORYLATION | 237 | 2,201 | 0,009 | High_in_IC25 |
| GO_RESPONSE_TO_OXIDATIVE_STRESS | 287 | 2,198 | 0,009 | High_in_IC25 |
| GO_REGULATION_OF_ION_HOMEOSTASIS | 125 | 2,197 | 0,009 | High_in_IC25 |
| GO_REGULATION_OF_T_CELL_PROLIFERATION | 95 | 2,197 | 0,009 | High_in_IC25 |
| GO_POSITIVE_REGULATION_OF_PROTEIN_KINASE_B_SIGNALING | 63 | 2,194 | 0,009 | High_in_IC25 |
| GO_LIPID_BIOSYNTHETIC_PROCESS | 403 | 2,188 | 0,009 | High_in_IC25 |
| GO_REGULATION_OF_PROTEIN_SECRETION | 266 | 2,188 | 0,009 | High_in_IC25 |
| GO_CELLULAR_RESPONSE_TO_ORGANIC_CYCLIC_COMPOUND | 346 | 2,187 | 0,009 | High_in_IC25 |
| GO_REPRODUCTIVE_SYSTEM_DEVELOPMENT | 311 | 2,184 | 0,01 | High_in_IC25 |
| GO_REGULATION_OF_ADHERENS_JUNCTION_ORGANIZATION | 41 | 2,184 | 0,01 | High_in_IC25 |
| GO_NEUTROPHIL_MEDIATED_IMMUNITY | 15 | 2,181 | 0,01 | High_in_IC25 |
| GO_JNK_CASCADE | 66 | 2,18 | 0,01 | High_in_IC25 |
| GO_CELLULAR_TRANSITION_METAL_ION_HOMEOSTASIS | 57 | 2,18 | 0,01 | High_in_IC25 |
| GO_NEGATIVE_REGULATION_OF_EXTRINSIC_APOPTOTIC_SIGNALING_PATHWAY | 77 | 2,177 | 0,01 | High_in_IC25 |
| GO_POSITIVE_REGULATION_OF_TRANSFERASE_ACTIVITY | 497 | 2,177 | 0,01 | High_in_IC25 |
| GO_CELLULAR_RESPONSE_TO_CARBOHYDRATE_STIMULUS | 57 | 2,175 | 0,01 | High_in_IC25 |
| GO_UBIQUITIN_DEPENDENT_PROTEIN_CATABOLIC_PROCESS_VIA_THE_MULTIVESICULAR_BODY_SORTING_PATHWAY | 18 | 2,174 | 0,01 | High_in_IC25 |
| GO_NEGATIVE_REGULATION_OF_CELL_ACTIVATION | 111 | 2,174 | 0,01 | High_in_IC25 |
| GO_POSITIVE_REGULATION_OF_ESTABLISHMENT_OF_PROTEIN_LOCALIZATION | 391 | 2,172 | 0,01 | High_in_IC25 |
| GO_OSTEOCLAST_DIFFERENTIATION | 24 | 2,169 | 0,01 | High_in_IC25 |
| GO_REGULATION_OF_LIPASE_ACTIVITY | 54 | 2,168 | 0,01 | High_in_IC25 |
| GO_POSITIVE_REGULATION_OF_SEQUENCE_SPECIFIC_DNA_BINDING_TRANSCRIPTION_FACTOR_ACTIVITY | 179 | 2,168 | 0,01 | High_in_IC25 |
| GO_GLYCOSYLATION | 194 | 2,162 | 0,01 | High_in_IC25 |
| GO_CELL_SUBSTRATE_JUNCTION_ASSEMBLY | 34 | 2,152 | 0,01 | High_in_IC25 |
| GO_CELLULAR_RESPONSE_TO_LIPID | 338 | 2,152 | 0,01 | High_in_IC25 |
| GO_ACTIVATION_OF_IMMUNE_RESPONSE | 297 | 2,15 | 0,01 | High_in_IC25 |
| GO_POSITIVE_REGULATION_OF_IMMUNE_EFFECTOR_PROCESS | 105 | 2,148 | 0,01 | High_in_IC25 |
| GO_SENSORY_PERCEPTION | 377 | 2,146 | 0,01 | High_in_IC25 |
| GO_REGULATION_OF_PEPTIDYL_SERINE_PHOSPHORYLATION | 91 | 2,145 | 0,01 | High_in_IC25 |
| GO_ENDOTHELIAL_CELL_DIFFERENTIATION | 56 | 2,14 | 0,01 | High_in_IC25 |
| GO_ADENYLATE_CYCLASE_INHIBITING_G_PROTEIN_COUPLED_RECEPTOR_SIGNALING_PATHWAY | 35 | 2,14 | 0,01 | High_in_IC25 |
| GO_AUTOPHAGY | 326 | 2,138 | 0,01 | High_in_IC25 |
| GO_CELL_GROWTH | 99 | 2,137 | 0,01 | High_in_IC25 |
| GO_LYMPHOCYTE_ACTIVATION | 241 | 2,135 | 0,01 | High_in_IC25 |
| GO_INFLAMMATORY_RESPONSE | 289 | 2,131 | 0,01 | High_in_IC25 |
| GO_PROTEIN_DEPHOSPHORYLATION | 161 | 2,131 | 0,01 | High_in_IC25 |
| GO_PROTEIN_N_LINKED_GLYCOSYLATION | 59 | 2,123 | 0,01 | High_in_IC25 |
| GO_RETROGRADE_PROTEIN_TRANSPORT_ER_TO_CYTOSOL | 15 | 2,119 | 0,01 | High_in_IC25 |
| GO_ENDOTHELIUM_DEVELOPMENT | 71 | 2,119 | 0,01 | High_in_IC25 |
| GO_ORGANIC_ANION_TRANSPORT | 255 | 2,116 | 0,01 | High_in_IC25 |
| GO_ACTIVATION_OF_PHOSPHOLIPASE_C_ACTIVITY | 18 | 2,116 | 0,01 | High_in_IC25 |
| GO_GASTRULATION | 116 | 2,115 | 0,01 | High_in_IC25 |
| GO_CERAMIDE_BIOSYNTHETIC_PROCESS | 23 | 2,114 | 0,01 | High_in_IC25 |
| GO_REGULATION_OF_RECEPTOR_ACTIVITY | 85 | 2,112 | 0,01 | High_in_IC25 |
| GO_NEGATIVE_REGULATION_OF_MACROAUTOPHAGY | 19 | 2,111 | 0,01 | High_in_IC25 |
| GO_RESPONSE_TO_TRANSITION_METAL_NANOPARTICLE | 107 | 2,11 | 0,01 | High_in_IC25 |
| GO_NEGATIVE_REGULATION_OF_CELLULAR_CATABOLIC_PROCESS | 123 | 2,106 | 0,01 | High_in_IC25 |
| GO_CELLULAR_RESPONSE_TO_BIOTIC_STIMULUS | 120 | 2,106 | 0,01 | High_in_IC25 |
| GO_REGULATION_OF_AUTOPHAGOSOME_ASSEMBLY | 31 | 2,103 | 0,01 | High_in_IC25 |
| GO_NEGATIVE_REGULATION_OF_TRANSFORMING_GROWTH_FACTOR_BETA_RECEPTOR_SIGNALING_PATHWAY | 54 | 2,102 | 0,01 | High_in_IC25 |
| GO_REGULATION_OF_ERAD_PATHWAY | 23 | 2,102 | 0,01 | High_in_IC25 |
| GO_REGULATION_OF_SEQUENCE_SPECIFIC_DNA_BINDING_TRANSCRIPTION_FACTOR_ACTIVITY | 285 | 2,1 | 0,01 | High_in_IC25 |
| GO_AMINO_SUGAR_METABOLIC_PROCESS | 31 | 2,099 | 0,01 | High_in_IC25 |
| GO_GLYCOPROTEIN_METABOLIC_PROCESS | 257 | 2,095 | 0,02 | High_in_IC25 |
| GO_NEGATIVE_REGULATION_OF_RESPONSE_TO_EXTERNAL_STIMULUS | 198 | 2,091 | 0,02 | High_in_IC25 |
| GO_ESTABLISHMENT_OF_ENDOTHELIAL_BARRIER | 29 | 2,09 | 0,02 | High_in_IC25 |
| GO_SECOND_MESSENGER_MEDIATED_SIGNALING | 96 | 2,09 | 0,02 | High_in_IC25 |
| GO_VESICLE_DOCKING_INVOLVED_IN_EXOCYTOSIS | 32 | 2,088 | 0,02 | High_in_IC25 |
| GO_PROTEIN_LOCALIZATION_TO_CELL_PERIPHERY | 125 | 2,087 | 0,02 | High_in_IC25 |
| GO_CARBOHYDRATE_DERIVATIVE_BIOSYNTHETIC_PROCESS | 459 | 2,085 | 0,02 | High_in_IC25 |
| GO_REGULATION_OF_PHOSPHOLIPASE_ACTIVITY | 44 | 2,084 | 0,02 | High_in_IC25 |
| GO_ER_NUCLEUS_SIGNALING_PATHWAY | 33 | 2,082 | 0,02 | High_in_IC25 |
| GO_REGULATION_OF_LEUKOCYTE_PROLIFERATION | 133 | 2,08 | 0,02 | High_in_IC25 |
| GO_MICROVILLUS_ORGANIZATION | 19 | 2,08 | 0,02 | High_in_IC25 |
| GO_BONE_RESORPTION | 18 | 2,079 | 0,02 | High_in_IC25 |
| GO_POSITIVE_REGULATION_OF_TRANSCRIPTION_FACTOR_IMPORT_INTO_NUCLEUS | 36 | 2,077 | 0,02 | High_in_IC25 |
| GO_MEMBRANE_FUSION | 128 | 2,077 | 0,02 | High_in_IC25 |
| GO_LEUKOCYTE_CELL_CELL_ADHESION | 181 | 2,077 | 0,02 | High_in_IC25 |
| GO_NECROPTOTIC_PROCESS | 16 | 2,074 | 0,02 | High_in_IC25 |
| GO_RESPONSE_TO_TRANSFORMING_GROWTH_FACTOR_BETA | 108 | 2,073 | 0,02 | High_in_IC25 |
| GO_TRANSITION_METAL_ION_HOMEOSTASIS | 81 | 2,073 | 0,02 | High_in_IC25 |
| GO_MEMBRANE_INVAGINATION | 22 | 2,071 | 0,02 | High_in_IC25 |
| GO_REGULATION_OF_MAST_CELL_ACTIVATION | 29 | 2,061 | 0,02 | High_in_IC25 |
| GO_POSITIVE_REGULATION_OF_INFLAMMATORY_RESPONSE | 64 | 2,058 | 0,02 | High_in_IC25 |
| GO_LYMPHOCYTE_COSTIMULATION | 45 | 2,057 | 0,02 | High_in_IC25 |
| GO_REGULATION_OF_ENDOCYTOSIS | 147 | 2,057 | 0,02 | High_in_IC25 |
| GO_SPROUTING_ANGIOGENESIS | 37 | 2,055 | 0,02 | High_in_IC25 |
| GO_REGULATION_OF_TRANSPORTER_ACTIVITY | 130 | 2,05 | 0,02 | High_in_IC25 |
| GO_POSITIVE_REGULATION_OF_ORGANELLE_ASSEMBLY | 41 | 2,046 | 0,02 | High_in_IC25 |
| GO_CELLULAR_RESPONSE_TO_ALCOHOL | 88 | 2,043 | 0,02 | High_in_IC25 |
| GO_FORMATION_OF_PRIMARY_GERM_LAYER | 81 | 2,041 | 0,02 | High_in_IC25 |
| GO_NEGATIVE_REGULATION_OF_ERBB_SIGNALING_PATHWAY | 42 | 2,038 | 0,02 | High_in_IC25 |
| GO_NEGATIVE_REGULATION_OF_ESTABLISHMENT_OF_PROTEIN_LOCALIZATION | 154 | 2,034 | 0,02 | High_in_IC25 |
| GO_CELL_MORPHOGENESIS_INVOLVED_IN_NEURON_DIFFERENTIATION | 254 | 2,033 | 0,02 | High_in_IC25 |
| GO_RESPONSE_TO_HYDROGEN_PEROXIDE | 98 | 2,028 | 0,02 | High_in_IC25 |
| GO_NEGATIVE_REGULATION_OF_ACTIN_FILAMENT_DEPOLYMERIZATION | 27 | 2,027 | 0,02 | High_in_IC25 |
| GO_ARP2_3_COMPLEX_MEDIATED_ACTIN_NUCLEATION | 15 | 2,027 | 0,02 | High_in_IC25 |
| GO_POSITIVE_REGULATION_OF_SUBSTRATE_ADHESION_DEPENDENT_CELL_SPREADING | 23 | 2,025 | 0,02 | High_in_IC25 |
| GO_ZINC_ION_HOMEOSTASIS | 16 | 2,023 | 0,02 | High_in_IC25 |
| GO_CALCIUM_MEDIATED_SIGNALING | 56 | 2,022 | 0,02 | High_in_IC25 |
| GO_CALCIUM_ION_IMPORT_INTO_CYTOSOL | 27 | 2,019 | 0,02 | High_in_IC25 |
| GO_DEVELOPMENTAL_MATURATION | 138 | 2,016 | 0,02 | High_in_IC25 |
| GO_REGULATION_OF_NATURAL_KILLER_CELL_ACTIVATION | 19 | 2,015 | 0,02 | High_in_IC25 |
| GO_FIBRIL_ORGANIZATION | 15 | 2,015 | 0,02 | High_in_IC25 |
| GO_PROTEIN_AUTOPHOSPHORYLATION | 150 | 2,014 | 0,02 | High_in_IC25 |
| GO_SIGNAL_RELEASE | 116 | 2,013 | 0,02 | High_in_IC25 |
| GO_REGULATION_OF_RESPONSE_TO_CYTOKINE_STIMULUS | 113 | 2,012 | 0,02 | High_in_IC25 |
| GO_RESPONSE_TO_LIPOPROTEIN_PARTICLE | 15 | 2,012 | 0,02 | High_in_IC25 |
| GO_DEVELOPMENTAL_PROCESS_INVOLVED_IN_REPRODUCTION | 431 | 2,009 | 0,02 | High_in_IC25 |
| GO_NEGATIVE_REGULATION_OF_MAP_KINASE_ACTIVITY | 63 | 2,008 | 0,02 | High_in_IC25 |
| GO_CELL_CHEMOTAXIS | 101 | 2,008 | 0,02 | High_in_IC25 |
| GO_POSITIVE_REGULATION_OF_CELLULAR_PROTEIN_LOCALIZATION | 295 | 2,006 | 0,02 | High_in_IC25 |
| GO_LIPID_LOCALIZATION | 178 | 2,005 | 0,02 | High_in_IC25 |
| GO_RETROGRADE_VESICLE_MEDIATED_TRANSPORT_GOLGI_TO_ER | 74 | 2,005 | 0,02 | High_in_IC25 |
| GO_REGULATION_OF_ACTIN_FILAMENT_DEPOLYMERIZATION | 39 | 2,001 | 0,02 | High_in_IC25 |
| GO_BROWN_FAT_CELL_DIFFERENTIATION | 19 | 1,999 | 0,02 | High_in_IC25 |
| GO_CELLULAR_RESPONSE_TO_FLUID_SHEAR_STRESS | 18 | 1,997 | 0,02 | High_in_IC25 |
| GO_NEGATIVE_REGULATION_OF_PROTEOLYSIS | 232 | 1,995 | 0,02 | High_in_IC25 |
| GO_POSITIVE_REGULATION_OF_INTRACELLULAR_TRANSPORT | 294 | 1,994 | 0,02 | High_in_IC25 |
| GO_NEURON_PROJECTION_MORPHOGENESIS | 281 | 1,992 | 0,02 | High_in_IC25 |
| GO_REGULATION_OF_NECROTIC_CELL_DEATH | 22 | 1,991 | 0,02 | High_in_IC25 |
| GO_HOMEOSTASIS_OF_NUMBER_OF_CELLS_WITHIN_A_TISSUE | 21 | 1,986 | 0,03 | High_in_IC25 |
| GO_ACTIN_NUCLEATION | 18 | 1,984 | 0,03 | High_in_IC25 |
| GO_IMMUNE_RESPONSE_REGULATING_CELL_SURFACE_RECEPTOR_SIGNALING_PATHWAY | 226 | 1,983 | 0,03 | High_in_IC25 |
| GO_INACTIVATION_OF_MAPK_ACTIVITY | 24 | 1,983 | 0,03 | High_in_IC25 |
| GO_HORMONE_TRANSPORT | 49 | 1,983 | 0,03 | High_in_IC25 |
| GO_POSITIVE_REGULATION_OF_EXTRINSIC_APOPTOTIC_SIGNALING_PATHWAY_VIA_DEATH_DOMAIN_RECEPTORS | 15 | 1,982 | 0,03 | High_in_IC25 |
| GO_REGULATION_OF_INTRACELLULAR_PROTEIN_TRANSPORT | 305 | 1,978 | 0,03 | High_in_IC25 |
| GO_CELLULAR_RESPONSE_TO_HYDROGEN_PEROXIDE | 56 | 1,975 | 0,03 | High_in_IC25 |
| GO_ALCOHOL_BIOSYNTHETIC_PROCESS | 89 | 1,975 | 0,03 | High_in_IC25 |
| GO_DENDRITE_DEVELOPMENT | 55 | 1,975 | 0,03 | High_in_IC25 |
| GO_ENDODERM_FORMATION | 40 | 1,972 | 0,03 | High_in_IC25 |
| GO_INTEGRIN_MEDIATED_SIGNALING_PATHWAY | 65 | 1,972 | 0,03 | High_in_IC25 |
| GO_NEGATIVE_REGULATION_OF_PEPTIDASE_ACTIVITY | 164 | 1,972 | 0,03 | High_in_IC25 |
| GO_POSITIVE_REGULATION_OF_AXONOGENESIS | 57 | 1,97 | 0,03 | High_in_IC25 |
| GO_CYTOKINE_PRODUCTION | 73 | 1,969 | 0,03 | High_in_IC25 |
| GO_REGULATION_OF_LEUKOCYTE_DIFFERENTIATION | 163 | 1,965 | 0,03 | High_in_IC25 |
| GO_POSITIVE_REGULATION_OF_NF_KAPPAB_TRANSCRIPTION_FACTOR_ACTIVITY | 107 | 1,964 | 0,03 | High_in_IC25 |
| GO_OLIGOSACCHARIDE_METABOLIC_PROCESS | 42 | 1,963 | 0,03 | High_in_IC25 |
| GO_MORPHOGENESIS_OF_AN_EPITHELIAL_SHEET | 37 | 1,962 | 0,03 | High_in_IC25 |
| GO_SENSORY_PERCEPTION_OF_TEMPERATURE_STIMULUS | 16 | 1,962 | 0,03 | High_in_IC25 |
| GO_APOPTOTIC_SIGNALING_PATHWAY | 238 | 1,961 | 0,03 | High_in_IC25 |
| GO_POSITIVE_REGULATION_OF_HORMONE_SECRETION | 75 | 1,961 | 0,03 | High_in_IC25 |
| GO_MAINTENANCE_OF_PROTEIN_LOCALIZATION_IN_ORGANELLE | 22 | 1,96 | 0,03 | High_in_IC25 |
| GO_GOLGI_TO_PLASMA_MEMBRANE_TRANSPORT | 38 | 1,959 | 0,03 | High_in_IC25 |
| GO_REGULATION_OF_INSULIN_RECEPTOR_SIGNALING_PATHWAY | 36 | 1,958 | 0,03 | High_in_IC25 |
| GO_REGULATION_OF_TRANSMEMBRANE_RECEPTOR_PROTEIN_SERINE_THREONINE_KINASE_SIGNALING_PATHWAY | 145 | 1,956 | 0,03 | High_in_IC25 |
| GO_POSITIVE_REGULATION_OF_EXOCYTOSIS | 61 | 1,954 | 0,03 | High_in_IC25 |
| GO_REGULATION_OF_BLOOD_CIRCULATION | 176 | 1,954 | 0,03 | High_in_IC25 |
| GO_CYTOSOLIC_CALCIUM_ION_TRANSPORT | 34 | 1,954 | 0,03 | High_in_IC25 |
| GO_REGULATION_OF_CELL_SIZE | 127 | 1,953 | 0,03 | High_in_IC25 |
| GO_REGULATION_OF_PROTEIN_AUTOPHOSPHORYLATION | 29 | 1,951 | 0,03 | High_in_IC25 |
| GO_CELLULAR_GLUCOSE_HOMEOSTASIS | 56 | 1,949 | 0,03 | High_in_IC25 |
| GO_REGULATION_OF_CAMP_METABOLIC_PROCESS | 77 | 1,948 | 0,03 | High_in_IC25 |
| GO_KERATINIZATION | 27 | 1,945 | 0,03 | High_in_IC25 |
| GO_ANION_TRANSMEMBRANE_TRANSPORT | 152 | 1,944 | 0,03 | High_in_IC25 |
| GO_REGULATION_OF_POSITIVE_CHEMOTAXIS | 15 | 1,943 | 0,03 | High_in_IC25 |
| GO_REGULATION_OF_TRANSFORMING_GROWTH_FACTOR_BETA_PRODUCTION | 21 | 1,943 | 0,03 | High_in_IC25 |
| GO_RESPONSE_TO_DRUG | 311 | 1,94 | 0,03 | High_in_IC25 |
| GO_REGULATION_OF_IMMUNOGLOBULIN_PRODUCTION | 31 | 1,938 | 0,03 | High_in_IC25 |
| GO_POSITIVE_REGULATION_OF_NERVOUS_SYSTEM_DEVELOPMENT | 303 | 1,937 | 0,03 | High_in_IC25 |
| GO_POSITIVE_REGULATION_OF_ENDOTHELIAL_CELL_MIGRATION | 49 | 1,937 | 0,03 | High_in_IC25 |
| GO_CELLULAR_PIGMENTATION | 43 | 1,936 | 0,03 | High_in_IC25 |
| GO_REGULATION_OF_SUBSTRATE_ADHESION_DEPENDENT_CELL_SPREADING | 35 | 1,935 | 0,03 | High_in_IC25 |
| GO_AUTOPHAGOSOME_ORGANIZATION | 36 | 1,931 | 0,03 | High_in_IC25 |
| GO_POSITIVE_REGULATION_OF_IMMUNOGLOBULIN_PRODUCTION | 17 | 1,93 | 0,03 | High_in_IC25 |
| GO_NEGATIVE_REGULATION_OF_CELL_PROJECTION_ORGANIZATION | 113 | 1,93 | 0,03 | High_in_IC25 |
| GO_KERATINOCYTE_DIFFERENTIATION | 70 | 1,93 | 0,03 | High_in_IC25 |
| GO_POSITIVE_REGULATION_OF_PROTEIN_SECRETION | 142 | 1,926 | 0,03 | High_in_IC25 |
| GO_REGULATION_OF_EPITHELIAL_CELL_PROLIFERATION | 209 | 1,925 | 0,03 | High_in_IC25 |
| GO_PROTEIN_LOCALIZATION_TO_ENDOPLASMIC_RETICULUM | 117 | 1,92 | 0,03 | High_in_IC25 |
| GO_POSITIVE_REGULATION_OF_TRANSMEMBRANE_TRANSPORT | 83 | 1,92 | 0,03 | High_in_IC25 |
| GO_REGULATION_OF_ENDOTHELIAL_CELL_MIGRATION | 81 | 1,917 | 0,03 | High_in_IC25 |
| GO_HEART_DEVELOPMENT | 324 | 1,917 | 0,03 | High_in_IC25 |
| GO_REGULATION_OF_CALCIUM_ION_TRANSPORT_INTO_CYTOSOL | 56 | 1,914 | 0,03 | High_in_IC25 |
| GO_REGULATION_OF_BINDING | 237 | 1,913 | 0,03 | High_in_IC25 |
| GO_TUBE_DEVELOPMENT | 396 | 1,912 | 0,03 | High_in_IC25 |
| GO_NEGATIVE_REGULATION_OF_ION_TRANSPORT | 81 | 1,908 | 0,03 | High_in_IC25 |
| GO_NEGATIVE_REGULATION_OF_HOMOTYPIC_CELL_CELL_ADHESION | 73 | 1,907 | 0,03 | High_in_IC25 |
| GO_ER_ASSOCIATED_UBIQUITIN_DEPENDENT_PROTEIN_CATABOLIC_PROCESS | 56 | 1,906 | 0,04 | High_in_IC25 |
| GO_ACTIVATION_OF_JUN_KINASE_ACTIVITY | 31 | 1,906 | 0,04 | High_in_IC25 |
| GO_PLACENTA_DEVELOPMENT | 117 | 1,905 | 0,04 | High_in_IC25 |
| GO_RESPONSE_TO_MECHANICAL_STIMULUS | 151 | 1,904 | 0,04 | High_in_IC25 |
| GO_NEGATIVE_REGULATION_OF_IMMUNE_RESPONSE | 80 | 1,904 | 0,04 | High_in_IC25 |
| GO_FEMALE_GAMETE_GENERATION | 68 | 1,901 | 0,04 | High_in_IC25 |
| GO_POSITIVE_REGULATION_OF_PEPTIDYL_TYROSINE_PHOSPHORYLATION | 106 | 1,898 | 0,04 | High_in_IC25 |
| GO_REGULATION_OF_PROTEIN_BINDING | 143 | 1,898 | 0,04 | High_in_IC25 |
| GO_REGULATION_OF_PEPTIDYL_THREONINE_PHOSPHORYLATION | 31 | 1,898 | 0,04 | High_in_IC25 |
| GO_NEGATIVE_REGULATION_OF_CELL_DIFFERENTIATION | 423 | 1,896 | 0,04 | High_in_IC25 |
| GO_REGULATION_OF_PROTEIN_COMPLEX_ASSEMBLY | 314 | 1,896 | 0,04 | High_in_IC25 |
| GO_REGULATION_OF_DEVELOPMENTAL_GROWTH | 195 | 1,895 | 0,04 | High_in_IC25 |
| GO_PROTEIN_DEGLYCOSYLATION | 16 | 1,892 | 0,04 | High_in_IC25 |
| GO_REGULATION_OF_ESTABLISHMENT_OF_PROTEIN_LOCALIZATION_TO_PLASMA_MEMBRANE | 41 | 1,892 | 0,04 | High_in_IC25 |
| GO_PHOSPHOLIPID_METABOLIC_PROCESS | 285 | 1,891 | 0,04 | High_in_IC25 |
| GO_FAT_CELL_DIFFERENTIATION | 83 | 1,89 | 0,04 | High_in_IC25 |
| GO_NEGATIVE_REGULATION_OF_EXTRINSIC_APOPTOTIC_SIGNALING_PATHWAY_VIA_DEATH_DOMAIN_RECEPTORS | 26 | 1,89 | 0,04 | High_in_IC25 |
| GO_REGULATION_OF_CELLULAR_RESPONSE_TO_GROWTH_FACTOR_STIMULUS | 164 | 1,888 | 0,04 | High_in_IC25 |
| GO_NEGATIVE_REGULATION_OF_RESPONSE_TO_EXTRACELLULAR_STIMULUS | 29 | 1,887 | 0,04 | High_in_IC25 |
| GO_REGULATION_OF_SMOOTH_MUSCLE_CELL_MIGRATION | 39 | 1,887 | 0,04 | High_in_IC25 |
| GO_REGULATION_OF_ANTIGEN_RECEPTOR_MEDIATED_SIGNALING_PATHWAY | 30 | 1,887 | 0,04 | High_in_IC25 |
| GO_CELL_CYCLE_ARREST | 130 | 1,887 | 0,04 | High_in_IC25 |
| GO_REGULATION_OF_NUCLEOTIDE_CATABOLIC_PROCESS | 27 | 1,886 | 0,04 | High_in_IC25 |
| GO_POSITIVE_REGULATION_OF_CYTOSKELETON_ORGANIZATION | 139 | 1,884 | 0,04 | High_in_IC25 |
| GO_REGULATION_OF_MEMBRANE_PROTEIN_ECTODOMAIN_PROTEOLYSIS | 15 | 1,884 | 0,04 | High_in_IC25 |
| GO_REGULATION_OF_LEUKOCYTE_APOPTOTIC_PROCESS | 55 | 1,882 | 0,04 | High_in_IC25 |
| GO_NEGATIVE_REGULATION_OF_RESPONSE_TO_ENDOPLASMIC_RETICULUM_STRESS | 31 | 1,88 | 0,04 | High_in_IC25 |
| GO_REGULATION_OF_HEART_CONTRACTION | 129 | 1,88 | 0,04 | High_in_IC25 |
| GO_RESPONSE_TO_INTERLEUKIN_1 | 84 | 1,879 | 0,04 | High_in_IC25 |
| GO_TISSUE_HOMEOSTASIS | 112 | 1,879 | 0,04 | High_in_IC25 |
| GO_GLUCOSAMINE_CONTAINING_COMPOUND_METABOLIC_PROCESS | 19 | 1,879 | 0,04 | High_in_IC25 |
| GO_PROTEIN_UBIQUITINATION_INVOLVED_IN_UBIQUITIN_DEPENDENT_PROTEIN_CATABOLIC_PROCESS | 119 | 1,877 | 0,04 | High_in_IC25 |
| GO_INTERACTION_WITH_HOST | 106 | 1,875 | 0,04 | High_in_IC25 |
| GO_REGULATION_OF_CELL_MIGRATION_INVOLVED_IN_SPROUTING_ANGIOGENESIS | 17 | 1,875 | 0,04 | High_in_IC25 |
| GO_REGULATION_OF_FATTY_ACID_BIOSYNTHETIC_PROCESS | 24 | 1,874 | 0,04 | High_in_IC25 |
| GO_GLYCEROLIPID_METABOLIC_PROCESS | 263 | 1,873 | 0,04 | High_in_IC25 |
| GO_INTESTINAL_ABSORPTION | 18 | 1,873 | 0,04 | High_in_IC25 |
| GO_STEROID_BIOSYNTHETIC_PROCESS | 83 | 1,873 | 0,04 | High_in_IC25 |
| GO_TOLL_LIKE_RECEPTOR_SIGNALING_PATHWAY | 64 | 1,872 | 0,04 | High_in_IC25 |
| GO_POSITIVE_REGULATION_OF_EXTRINSIC_APOPTOTIC_SIGNALING_PATHWAY | 46 | 1,871 | 0,04 | High_in_IC25 |
| GO_REGULATION_OF_PHOSPHATASE_ACTIVITY | 89 | 1,87 | 0,04 | High_in_IC25 |
| GO_GANGLIOSIDE_METABOLIC_PROCESS | 15 | 1,87 | 0,04 | High_in_IC25 |
| GO_PHOSPHOLIPID_BIOSYNTHETIC_PROCESS | 190 | 1,869 | 0,04 | High_in_IC25 |
| GO_CELLULAR_RESPONSE_TO_OSMOTIC_STRESS | 18 | 1,869 | 0,04 | High_in_IC25 |
| GO_CELLULAR_RESPONSE_TO_OXIDATIVE_STRESS | 161 | 1,868 | 0,04 | High_in_IC25 |
| GO_ENDODERM_DEVELOPMENT | 55 | 1,865 | 0,04 | High_in_IC25 |
| GO_NEGATIVE_REGULATION_OF_PROTEIN_TYROSINE_KINASE_ACTIVITY | 17 | 1,865 | 0,04 | High_in_IC25 |
| GO_POSITIVE_REGULATION_OF_REGULATED_SECRETORY_PATHWAY | 32 | 1,864 | 0,04 | High_in_IC25 |
| GO_VISUAL_BEHAVIOR | 33 | 1,862 | 0,04 | High_in_IC25 |
| GO_PEPTIDYL_ASPARAGINE_MODIFICATION | 33 | 1,861 | 0,04 | High_in_IC25 |
| GO_NEGATIVE_REGULATION_OF_JNK_CASCADE | 26 | 1,861 | 0,04 | High_in_IC25 |
| GO_POSITIVE_REGULATION_OF_NF_KAPPAB_IMPORT_INTO_NUCLEUS | 17 | 1,861 | 0,04 | High_in_IC25 |
| GO_CELLULAR_RESPONSE_TO_AMINO_ACID_STIMULUS | 40 | 1,86 | 0,04 | High_in_IC25 |
| GO_POSITIVE_REGULATION_OF_ION_TRANSPORT | 144 | 1,858 | 0,04 | High_in_IC25 |
| GO_REGULATION_OF_AXONOGENESIS | 127 | 1,858 | 0,04 | High_in_IC25 |
| GO_ADHERENS_JUNCTION_ORGANIZATION | 56 | 1,857 | 0,04 | High_in_IC25 |
| GO_RESPONSE_TO_OSMOTIC_STRESS | 51 | 1,853 | 0,04 | High_in_IC25 |
| GO_POSITIVE_REGULATION_OF_TUMOR_NECROSIS_FACTOR_SUPERFAMILY_CYTOKINE_PRODUCTION | 36 | 1,853 | 0,04 | High_in_IC25 |
| GO_POSITIVE_REGULATION_OF_CYTOPLASMIC_TRANSPORT | 218 | 1,853 | 0,04 | High_in_IC25 |
| GO_FC_RECEPTOR_SIGNALING_PATHWAY | 161 | 1,852 | 0,04 | High_in_IC25 |
| GO_REGULATION_OF_EXTENT_OF_CELL_GROWTH | 76 | 1,852 | 0,04 | High_in_IC25 |
| GO_INSULIN_SECRETION | 24 | 1,851 | 0,04 | High_in_IC25 |
| GO_CELLULAR_RESPONSE_TO_GLUCAGON_STIMULUS | 27 | 1,85 | 0,04 | High_in_IC25 |
| GO_LOCOMOTORY_BEHAVIOR | 109 | 1,848 | 0,04 | High_in_IC25 |
| GO_SYNAPTIC_SIGNALING | 240 | 1,845 | 0,04 | High_in_IC25 |
| GO_PEPTIDE_SECRETION | 36 | 1,843 | 0,04 | High_in_IC25 |
| GO_REGULATION_OF_ANION_TRANSPORT | 93 | 1,842 | 0,04 | High_in_IC25 |
| GO_T_CELL_ACTIVATION_INVOLVED_IN_IMMUNE_RESPONSE | 36 | 1,841 | 0,04 | High_in_IC25 |
| GO_AMINOGLYCAN_CATABOLIC_PROCESS | 51 | 1,839 | 0,04 | High_in_IC25 |
| GO_RESPONSE_TO_ACID_CHEMICAL | 235 | 1,838 | 0,04 | High_in_IC25 |
| GO_REGULATION_OF_PEPTIDE_SECRETION | 132 | 1,835 | 0,05 | High_in_IC25 |
| GO_ENDOTHELIAL_CELL_PROLIFERATION | 18 | 1,834 | 0,05 | High_in_IC25 |
| GO_CELLULAR_RESPONSE_TO_ABIOTIC_STIMULUS | 210 | 1,832 | 0,05 | High_in_IC25 |
| GO_NEGATIVE_REGULATION_OF_STRESS_ACTIVATED_PROTEIN_KINASE_SIGNALING_CASCADE | 34 | 1,832 | 0,05 | High_in_IC25 |
| GO_PEPTIDYL_TYROSINE_MODIFICATION | 133 | 1,83 | 0,05 | High_in_IC25 |
| GO_POSITIVE_REGULATION_OF_PEPTIDE_SECRETION | 57 | 1,83 | 0,05 | High_in_IC25 |
| GO_POSITIVE_REGULATION_OF_REACTIVE_OXYGEN_SPECIES_METABOLIC_PROCESS | 62 | 1,829 | 0,05 | High_in_IC25 |
| GO_VIRION_ASSEMBLY | 34 | 1,827 | 0,05 | High_in_IC25 |
| GO_SINGLE_ORGANISM_MEMBRANE_FUSION | 103 | 1,826 | 0,05 | High_in_IC25 |
| GO_REGULATION_OF_DEPHOSPHORYLATION | 115 | 1,824 | 0,05 | High_in_IC25 |
| GO_ENDOPLASMIC_RETICULUM_TO_CYTOSOL_TRANSPORT | 19 | 1,823 | 0,05 | High_in_IC25 |
| GO_POSITIVE_REGULATION_OF_DEPHOSPHORYLATION | 35 | 1,822 | 0,05 | High_in_IC25 |
| GO_POSITIVE_REGULATION_OF_ACTIN_FILAMENT_POLYMERIZATION | 55 | 1,821 | 0,05 | High_in_IC25 |
| GO_AXON_EXTENSION | 29 | 1,82 | 0,05 | High_in_IC25 |
| GO_REGULATION_OF_RECEPTOR_RECYCLING | 17 | 1,82 | 0,05 | High_in_IC25 |
| GO_NEGATIVE_REGULATION_OF_IMMUNE_EFFECTOR_PROCESS | 71 | 1,819 | 0,05 | High_in_IC25 |
| GO_NEGATIVE_REGULATION_OF_LEUKOCYTE_PROLIFERATION | 48 | 1,818 | 0,05 | High_in_IC25 |
| GO_AMIDE_TRANSPORT | 61 | 1,817 | 0,05 | High_in_IC25 |
| GO_REGULATION_OF_REACTIVE_OXYGEN_SPECIES_METABOLIC_PROCESS | 115 | 1,815 | 0,05 | High_in_IC25 |
| GO_CELLULAR_RESPONSE_TO_ACID_CHEMICAL | 132 | 1,815 | 0,05 | High_in_IC25 |
| GO_SUBSTRATE_DEPENDENT_CELL_MIGRATION | 24 | 1,814 | 0,05 | High_in_IC25 |
| GO_ERBB2_SIGNALING_PATHWAY | 36 | 1,813 | 0,05 | High_in_IC25 |
| GO_NEURON_PROJECTION_EXTENSION | 41 | 1,812 | 0,05 | High_in_IC25 |
| GO_VESICLE_MEDIATED_TRANSPORT_BETWEEN_ENDOSOMAL_COMPARTMENTS | 19 | 1,811 | 0,05 | High_in_IC25 |
| GO_SKIN_DEVELOPMENT | 150 | 1,811 | 0,05 | High_in_IC25 |
| GO_RESPONSE_TO_STEROL | 16 | 1,811 | 0,05 | High_in_IC25 |
| GO_RESPONSE_TO_NUTRIENT | 140 | 1,811 | 0,05 | High_in_IC25 |
| GO_PROTEIN_TARGETING_TO_PLASMA_MEMBRANE | 18 | 1,809 | 0,05 | High_in_IC25 |
| GO_POSITIVE_REGULATION_OF_NUCLEOCYTOPLASMIC_TRANSPORT | 94 | 1,809 | 0,05 | High_in_IC25 |
| GO_RNA_SPLICING | 303 | -7,345 | 0 | Low_in_IC25 |
| GO_RNA_SPLICING_VIA_TRANSESTERIFICATION_REACTIONS | 228 | -7,103 | 0 | Low_in_IC25 |
| GO_MRNA_PROCESSING | 353 | -7,016 | 0 | Low_in_IC25 |
| GO_CELLULAR_RESPIRATION | 132 | -5,97 | 0 | Low_in_IC25 |
| GO_DNA_REPLICATION | 180 | -5,842 | 0 | Low_in_IC25 |
| GO_NUCLEOSIDE_MONOPHOSPHATE_METABOLIC_PROCESS | 205 | -5,738 | 0 | Low_in_IC25 |
| GO_OXIDATIVE_PHOSPHORYLATION | 77 | -5,611 | 0 | Low_in_IC25 |
| GO_RIBONUCLEOPROTEIN_COMPLEX_BIOGENESIS | 395 | -5,399 | 0 | Low_in_IC25 |
| GO_REGULATION_OF_CELL_CYCLE_PROCESS | 480 | -5,372 | 0 | Low_in_IC25 |
| GO_NUCLEOSIDE_TRIPHOSPHATE_METABOLIC_PROCESS | 189 | -5,299 | 0 | Low_in_IC25 |
| GO_DNA_REPAIR | 423 | -5,295 | 0 | Low_in_IC25 |
| GO_NUCLEOBASE_CONTAINING_SMALL_MOLECULE_METABOLIC_PROCESS | 422 | -5,276 | 0 | Low_in_IC25 |
| GO_GLYCOSYL_COMPOUND_METABOLIC_PROCESS | 300 | -5,26 | 0 | Low_in_IC25 |
| GO_ELECTRON_TRANSPORT_CHAIN | 85 | -5,256 | 0 | Low_in_IC25 |
| GO_REGULATION_OF_MITOTIC_CELL_CYCLE | 419 | -5,217 | 0 | Low_in_IC25 |
| GO_RIBONUCLEOPROTEIN_COMPLEX_LOCALIZATION | 99 | -5,038 | 0 | Low_in_IC25 |
| GO_MITOCHONDRIAL_TRANSLATION | 105 | -5,017 | 0 | Low_in_IC25 |
| GO_NUCLEAR_EXPORT | 125 | -4,968 | 0 | Low_in_IC25 |
| GO_DNA_DEPENDENT_DNA_REPLICATION | 85 | -4,965 | 0 | Low_in_IC25 |
| GO_TRANSLATIONAL_TERMINATION | 92 | -4,917 | 0 | Low_in_IC25 |
| GO_NUCLEAR_CHROMOSOME_SEGREGATION | 184 | -4,889 | 0 | Low_in_IC25 |
| GO_NCRNA_PROCESSING | 359 | -4,888 | 0 | Low_in_IC25 |
| GO_MITOTIC_NUCLEAR_DIVISION | 315 | -4,854 | 0 | Low_in_IC25 |
| GO_SISTER_CHROMATID_SEGREGATION | 158 | -4,841 | 0 | Low_in_IC25 |
| GO_ORGANELLE_FISSION | 403 | -4,832 | 0 | Low_in_IC25 |
| GO_NCRNA_METABOLIC_PROCESS | 493 | -4,827 | 0 | Low_in_IC25 |
| GO_CHROMOSOME_SEGREGATION | 220 | -4,771 | 0 | Low_in_IC25 |
| GO_RNA_LOCALIZATION | 154 | -4,739 | 0 | Low_in_IC25 |
| GO_REGULATION_OF_CELL_CYCLE_PHASE_TRANSITION | 293 | -4,73 | 0 | Low_in_IC25 |
| GO_TRANSLATIONAL_ELONGATION | 110 | -4,717 | 0 | Low_in_IC25 |
| GO_PURINE_CONTAINING_COMPOUND_METABOLIC_PROCESS | 309 | -4,673 | 0 | Low_in_IC25 |
| GO_CELL_DIVISION | 400 | -4,586 | 0 | Low_in_IC25 |
| GO_POSTTRANSCRIPTIONAL_REGULATION_OF_GENE_EXPRESSION | 396 | -4,585 | 0 | Low_in_IC25 |
| GO_MITOCHONDRIAL_RESPIRATORY_CHAIN_COMPLEX_I_BIOGENESIS | 52 | -4,576 | 0 | Low_in_IC25 |
| GO_MACROMOLECULAR_COMPLEX_DISASSEMBLY | 166 | -4,558 | 0 | Low_in_IC25 |
| GO_SISTER_CHROMATID_COHESION | 101 | -4,551 | 0 | Low_in_IC25 |
| GO_CELLULAR_PROTEIN_COMPLEX_DISASSEMBLY | 115 | -4,549 | 0 | Low_in_IC25 |
| GO_ANAPHASE_PROMOTING_COMPLEX_DEPENDENT_CATABOLIC_PROCESS | 73 | -4,526 | 0 | Low_in_IC25 |
| GO_ENERGY_DERIVATION_BY_OXIDATION_OF_ORGANIC_COMPOUNDS | 187 | -4,492 | 0 | Low_in_IC25 |
| GO_DNA_RECOMBINATION | 176 | -4,469 | 0 | Low_in_IC25 |
| GO_DNA_TEMPLATED_TRANSCRIPTION_TERMINATION | 84 | -4,437 | 0 | Low_in_IC25 |
| GO_GENERATION_OF_PRECURSOR_METABOLITES_AND_ENERGY | 243 | -4,432 | 0 | Low_in_IC25 |
| GO_NEGATIVE_REGULATION_OF_PROTEIN_MODIFICATION_BY_SMALL_PROTEIN_CONJUGATION_OR_REMOVAL | 127 | -4,386 | 0 | Low_in_IC25 |
| GO_RNA_3_END_PROCESSING | 78 | -4,364 | 0 | Low_in_IC25 |
| GO_MITOCHONDRIAL_RESPIRATORY_CHAIN_COMPLEX_ASSEMBLY | 69 | -4,329 | 0 | Low_in_IC25 |
| GO_DNA_CONFORMATION_CHANGE | 222 | -4,254 | 0 | Low_in_IC25 |
| GO_NUCLEAR_TRANSPORT | 300 | -4,239 | 0 | Low_in_IC25 |
| GO_CELLULAR_COMPONENT_DISASSEMBLY | 422 | -4,224 | 0 | Low_in_IC25 |
| GO_REGULATION_OF_RNA_STABILITY | 126 | -4,216 | 0 | Low_in_IC25 |
| GO_NUCLEOBASE_CONTAINING_COMPOUND_TRANSPORT | 165 | -4,169 | 0 | Low_in_IC25 |
| GO_TERMINATION_OF_RNA_POLYMERASE_II_TRANSCRIPTION | 46 | -4,123 | 0 | Low_in_IC25 |
| GO_CELL_CYCLE_PHASE_TRANSITION | 220 | -4,12 | 0 | Low_in_IC25 |
| GO_RIBOSOME_BIOGENESIS | 285 | -4,06 | 0 | Low_in_IC25 |
| GO_TRNA_TRANSPORT | 32 | -4,053 | 0 | Low_in_IC25 |
| GO_AMIDE_BIOSYNTHETIC_PROCESS | 451 | -4,038 | 0 | Low_in_IC25 |
| GO_GENE_SILENCING_BY_RNA | 117 | -4,015 | 0 | Low_in_IC25 |
| GO_GENE_SILENCING | 170 | -3,993 | 0 | Low_in_IC25 |
| GO_CELL_CYCLE_CHECKPOINT | 178 | -3,955 | 0 | Low_in_IC25 |
| GO_MITOCHONDRIAL_TRANSMEMBRANE_TRANSPORT | 49 | -3,939 | 0 | Low_in_IC25 |
| GO_PROTEIN_SUMOYLATION | 110 | -3,927 | 0 | Low_in_IC25 |
| GO_DNA_BIOSYNTHETIC_PROCESS | 100 | -3,915 | 0 | Low_in_IC25 |
| GO_REGULATION_OF_PROTEIN_UBIQUITINATION_INVOLVED_IN_UBIQUITIN_DEPENDENT_PROTEIN_CATABOLIC_PROCESS | 94 | -3,851 | 0 | Low_in_IC25 |
| GO_CHROMATIN_MODIFICATION | 455 | -3,84 | 0 | Low_in_IC25 |
| GO_MEMBRANE_DISASSEMBLY | 43 | -3,826 | 0 | Low_in_IC25 |
| GO_DNA_SYNTHESIS_INVOLVED_IN_DNA_REPAIR | 62 | -3,803 | 0 | Low_in_IC25 |
| GO_TRNA_METABOLIC_PROCESS | 166 | -3,792 | 0 | Low_in_IC25 |
| GO_PEPTIDE_METABOLIC_PROCESS | 496 | -3,791 | 0 | Low_in_IC25 |
| GO_REGULATION_OF_CHROMOSOME_ORGANIZATION | 237 | -3,769 | 0 | Low_in_IC25 |
| GO_REGULATION_OF_CELLULAR_RESPONSE_TO_HEAT | 74 | -3,761 | 0 | Low_in_IC25 |
| GO_NEGATIVE_REGULATION_OF_CELL_CYCLE_PROCESS | 192 | -3,743 | 0 | Low_in_IC25 |
| GO_MULTI_ORGANISM_LOCALIZATION | 64 | -3,731 | 0 | Low_in_IC25 |
| GO_DNA_PACKAGING | 146 | -3,728 | 0 | Low_in_IC25 |
| GO_POSITIVE_REGULATION_OF_LIGASE_ACTIVITY | 103 | -3,678 | 0 | Low_in_IC25 |
| GO_NEGATIVE_REGULATION_OF_MITOTIC_CELL_CYCLE | 183 | -3,677 | 0 | Low_in_IC25 |
| GO_DNA_STRAND_ELONGATION_INVOLVED_IN_DNA_REPLICATION | 25 | -3,658 | 0 | Low_in_IC25 |
| GO_DNA_REPLICATION_INITIATION | 23 | -3,632 | 0 | Low_in_IC25 |
| GO_MRNA_3_END_PROCESSING | 55 | -3,618 | 0 | Low_in_IC25 |
| GO_NUCLEOTIDE_EXCISION_REPAIR | 105 | -3,618 | 0 | Low_in_IC25 |
| GO_COFACTOR_METABOLIC_PROCESS | 273 | -3,608 | 0 | Low_in_IC25 |
| GO_PURINE_NUCLEOSIDE_MONOPHOSPHATE_BIOSYNTHETIC_PROCESS | 54 | -3,59 | 0 | Low_in_IC25 |
| GO_RIBONUCLEOPROTEIN_COMPLEX_SUBUNIT_ORGANIZATION | 171 | -3,589 | 0 | Low_in_IC25 |
| GO_REGULATION_OF_TELOMERASE_RNA_LOCALIZATION_TO_CAJAL_BODY | 15 | -3,566 | 0 | Low_in_IC25 |
| GO_TELOMERE_MAINTENANCE_VIA_RECOMBINATION | 31 | -3,538 | 0 | Low_in_IC25 |
| GO_TRANSCRIPTION_COUPLED_NUCLEOTIDE_EXCISION_REPAIR | 72 | -3,532 | 0 | Low_in_IC25 |
| GO_TRANSLESION_SYNTHESIS | 37 | -3,512 | 0 | Low_in_IC25 |
| GO_CHROMATIN_ASSEMBLY_OR_DISASSEMBLY | 139 | -3,506 | 0 | Low_in_IC25 |
| GO_REGULATION_OF_LIGASE_ACTIVITY | 122 | -3,499 | 0 | Low_in_IC25 |
| GO_TRNA_PROCESSING | 106 | -3,492 | 0 | Low_in_IC25 |
| GO_PROTEIN_IMPORT | 129 | -3,483 | 0 | Low_in_IC25 |
| GO_DNA_DAMAGE_RESPONSE_DETECTION_OF_DNA_DAMAGE | 35 | -3,48 | 0 | Low_in_IC25 |
| GO_PEPTIDYL_LYSINE_MODIFICATION | 273 | -3,479 | 0 | Low_in_IC25 |
| GO_REGULATION_OF_ORGAN_MORPHOGENESIS | 188 | -3,474 | 0 | Low_in_IC25 |
| GO_MITOTIC_RECOMBINATION | 38 | -3,47 | 0 | Low_in_IC25 |
| GO_REGULATION_OF_MICROTUBULE_BASED_PROCESS | 205 | -3,47 | 0 | Low_in_IC25 |
| GO_MITOTIC_CELL_CYCLE_CHECKPOINT | 129 | -3,466 | 0 | Low_in_IC25 |
| GO_RRNA_METABOLIC_PROCESS | 237 | -3,455 | 0 | Low_in_IC25 |
| GO_REGULATION_OF_PROTEIN_MODIFICATION_BY_SMALL_PROTEIN_CONJUGATION_OR_REMOVAL | 256 | -3,453 | 0 | Low_in_IC25 |
| GO_NEGATIVE_REGULATION_OF_CELL_CYCLE | 379 | -3,421 | 0 | Low_in_IC25 |
| GO_CELL_CYCLE_G1_S_PHASE_TRANSITION | 94 | -3,409 | 0 | Low_in_IC25 |
| GO_DNA_INTEGRITY_CHECKPOINT | 135 | -3,407 | 0 | Low_in_IC25 |
| GO_PROTEIN_DNA_COMPLEX_SUBUNIT_ORGANIZATION | 180 | -3,404 | 0 | Low_in_IC25 |
| GO_MITOCHONDRIAL_ATP_SYNTHESIS_COUPLED_PROTON_TRANSPORT | 17 | -3,403 | 0 | Low_in_IC25 |
| GO_CELLULAR_PROTEIN_COMPLEX_ASSEMBLY | 288 | -3,378 | 0 | Low_in_IC25 |
| GO_RNA_CATABOLIC_PROCESS | 210 | -3,363 | 0 | Low_in_IC25 |
| GO_REGULATION_OF_MRNA_METABOLIC_PROCESS | 89 | -3,363 | 0 | Low_in_IC25 |
| GO_POSITIVE_REGULATION_OF_CELLULAR_PROTEIN_CATABOLIC_PROCESS | 176 | -3,345 | 0 | Low_in_IC25 |
| GO_MITOCHONDRIAL_TRANSPORT | 159 | -3,341 | 0 | Low_in_IC25 |
| GO_REGULATION_OF_DNA_METABOLIC_PROCESS | 279 | -3,332 | 0 | Low_in_IC25 |
| GO_POSITIVE_REGULATION_OF_PROTEIN_MODIFICATION_BY_SMALL_PROTEIN_CONJUGATION_OR_REMOVAL | 181 | -3,328 | 0 | Low_in_IC25 |
| GO_DNA_STRAND_ELONGATION | 29 | -3,314 | 0 | Low_in_IC25 |
| GO_GLYCOSYL_COMPOUND_BIOSYNTHETIC_PROCESS | 103 | -3,297 | 0 | Low_in_IC25 |
| GO_POSITIVE_REGULATION_OF_CHROMOSOME_ORGANIZATION | 130 | -3,289 | 0 | Low_in_IC25 |
| GO_REGULATION_OF_ESTABLISHMENT_OF_PLANAR_POLARITY | 99 | -3,283 | 0 | Low_in_IC25 |
| GO_MEIOTIC_CELL_CYCLE | 132 | -3,278 | 0,000007 | Low_in_IC25 |
| GO_POSTREPLICATION_REPAIR | 49 | -3,275 | 0,000007 | Low_in_IC25 |
| GO_POSITIVE_REGULATION_OF_PROTEOLYSIS | 312 | -3,247 | 0,000007 | Low_in_IC25 |
| GO_ORGANIC_CYCLIC_COMPOUND_CATABOLIC_PROCESS | 345 | -3,246 | 0,000007 | Low_in_IC25 |
| GO_MITOTIC_SISTER_CHROMATID_SEGREGATION | 80 | -3,244 | 0,000007 | Low_in_IC25 |
| GO_POSITIVE_REGULATION_OF_CELL_CYCLE_PROCESS | 212 | -3,237 | 0,000007 | Low_in_IC25 |
| GO_ESTABLISHMENT_OF_PROTEIN_LOCALIZATION_TO_ORGANELLE | 323 | -3,232 | 0,000007 | Low_in_IC25 |
| GO_NUCLEOSIDE_MONOPHOSPHATE_BIOSYNTHETIC_PROCESS | 73 | -3,232 | 0,000007 | Low_in_IC25 |
| GO_REGULATION_OF_CELLULAR_AMINO_ACID_METABOLIC_PROCESS | 54 | -3,207 | 0,000007 | Low_in_IC25 |
| GO_COENZYME_METABOLIC_PROCESS | 215 | -3,188 | 0,000006 | Low_in_IC25 |
| GO_NUCLEAR_IMPORT | 111 | -3,184 | 0,000006 | Low_in_IC25 |
| GO_ATP_DEPENDENT_CHROMATIN_REMODELING | 63 | -3,152 | 0,000006 | Low_in_IC25 |
| GO_PROTEIN_TARGETING_TO_MITOCHONDRION | 46 | -3,15 | 0,000006 | Low_in_IC25 |
| GO_NUCLEOSIDE_PHOSPHATE_BIOSYNTHETIC_PROCESS | 148 | -3,148 | 0,000006 | Low_in_IC25 |
| GO_REGULATION_OF_NUCLEAR_DIVISION | 131 | -3,146 | 0,000006 | Low_in_IC25 |
| GO_AEROBIC_RESPIRATION | 50 | -3,145 | 0,000006 | Low_in_IC25 |
| GO_REGULATION_OF_CELLULAR_PROTEIN_CATABOLIC_PROCESS | 246 | -3,143 | 0,000006 | Low_in_IC25 |
| GO_DNA_REPLICATION_INDEPENDENT_NUCLEOSOME_ORGANIZATION | 46 | -3,139 | 0,000006 | Low_in_IC25 |
| GO_PURINE_NUCLEOSIDE_BIOSYNTHETIC_PROCESS | 77 | -3,135 | 0,000006 | Low_in_IC25 |
| GO_COVALENT_CHROMATIN_MODIFICATION | 287 | -3,125 | 0,000006 | Low_in_IC25 |
| GO_RNA_MODIFICATION | 105 | -3,118 | 0,000006 | Low_in_IC25 |
| GO_NUCLEOTIDE_EXCISION_REPAIR_DNA_GAP_FILLING | 23 | -3,096 | 0,000006 | Low_in_IC25 |
| GO_REGULATION_OF_CHROMOSOME_SEGREGATION | 77 | -3,095 | 0,000006 | Low_in_IC25 |
| GO_SPLICEOSOMAL_SNRNP_ASSEMBLY | 33 | -3,093 | 0,000006 | Low_in_IC25 |
| GO_CELL_CYCLE_G2_M_PHASE_TRANSITION | 120 | -3,09 | 0,000006 | Low_in_IC25 |
| GO_NEGATIVE_REGULATION_OF_CELL_CYCLE_PHASE_TRANSITION | 133 | -3,086 | 0,000006 | Low_in_IC25 |
| GO_REGULATION_OF_SIGNAL_TRANSDUCTION_BY_P53_CLASS_MEDIATOR | 144 | -3,083 | 0,000006 | Low_in_IC25 |
| GO_NUCLEIC_ACID_PHOSPHODIESTER_BOND_HYDROLYSIS | 217 | -3,062 | 0,000006 | Low_in_IC25 |
| GO_REGULATION_OF_CENTROSOME_CYCLE | 34 | -3,058 | 0,00001 | Low_in_IC25 |
| GO_ANTIGEN_PROCESSING_AND_PRESENTATION_OF_EXOGENOUS_PEPTIDE_ANTIGEN_VIA_MHC_CLASS_I | 59 | -3,056 | 0,00001 | Low_in_IC25 |
| GO_DOUBLE_STRAND_BREAK_REPAIR | 134 | -3,046 | 0,00001 | Low_in_IC25 |
| GO_ENERGY_COUPLED_PROTON_TRANSPORT_DOWN_ELECTROCHEMICAL_GRADIENT | 22 | -3,039 | 0,00001 | Low_in_IC25 |
| GO_PROTEIN_LOCALIZATION_TO_ORGANELLE | 484 | -3,033 | 0,00001 | Low_in_IC25 |
| GO_REGULATION_OF_CELL_CYCLE_G1_S_PHASE_TRANSITION | 136 | -3,005 | 0,00001 | Low_in_IC25 |
| GO_MEIOTIC_CELL_CYCLE_PROCESS | 105 | -3,002 | 0,00001 | Low_in_IC25 |
| GO_PURINE_CONTAINING_COMPOUND_BIOSYNTHETIC_PROCESS | 110 | -2,999 | 0,00001 | Low_in_IC25 |
| GO_ATP_BIOSYNTHETIC_PROCESS | 31 | -2,991 | 0,00001 | Low_in_IC25 |
| GO_TELOMERE_ORGANIZATION | 90 | -2,985 | 0,00001 | Low_in_IC25 |
| GO_REGULATION_OF_CELL_DIVISION | 209 | -2,974 | 0,00001 | Low_in_IC25 |
| GO_NUCLEAR_ENVELOPE_ORGANIZATION | 72 | -2,971 | 0,00001 | Low_in_IC25 |
| GO_NEGATIVE_REGULATION_OF_ORGANELLE_ORGANIZATION | 331 | -2,935 | 0,00002 | Low_in_IC25 |
| GO_TRNA_MODIFICATION | 54 | -2,93 | 0,00002 | Low_in_IC25 |
| GO_MULTI_ORGANISM_METABOLIC_PROCESS | 136 | -2,923 | 0,00002 | Low_in_IC25 |
| GO_SPLICEOSOMAL_COMPLEX_ASSEMBLY | 37 | -2,905 | 0,00002 | Low_in_IC25 |
| GO_POSITIVE_REGULATION_OF_CELL_CYCLE | 289 | -2,903 | 0,00002 | Low_in_IC25 |
| GO_REGULATION_OF_CELLULAR_AMIDE_METABOLIC_PROCESS | 307 | -2,872 | 0,00004 | Low_in_IC25 |
| GO_COFACTOR_BIOSYNTHETIC_PROCESS | 140 | -2,867 | 0,00004 | Low_in_IC25 |
| GO_POSITIVE_REGULATION_OF_PROTEIN_CATABOLIC_PROCESS | 233 | -2,85 | 0,00005 | Low_in_IC25 |
| GO_DNA_TEMPLATED_TRANSCRIPTION_INITIATION | 169 | -2,845 | 0,00005 | Low_in_IC25 |
| GO_NIK_NF_KAPPAB_SIGNALING | 79 | -2,832 | 0,00006 | Low_in_IC25 |
| GO_DNA_TEMPLATED_TRANSCRIPTION_ELONGATION | 93 | -2,831 | 0,00006 | Low_in_IC25 |
| GO_MEIOSIS_I | 53 | -2,827 | 0,00006 | Low_in_IC25 |
| GO_REGULATION_OF_CELLULAR_AMINE_METABOLIC_PROCESS | 70 | -2,825 | 0,00006 | Low_in_IC25 |
| GO_PROTEIN_TARGETING | 360 | -2,815 | 0,00007 | Low_in_IC25 |
| GO_REGULATION_OF_MICROTUBULE_POLYMERIZATION_OR_DEPOLYMERIZATION | 153 | -2,814 | 0,00007 | Low_in_IC25 |
| GO_NON_CANONICAL_WNT_SIGNALING_PATHWAY | 125 | -2,813 | 0,00007 | Low_in_IC25 |
| GO_NEGATIVE_REGULATION_OF_CHROMOSOME_ORGANIZATION | 85 | -2,793 | 0,0001 | Low_in_IC25 |
| GO_ANTIGEN_PROCESSING_AND_PRESENTATION_OF_PEPTIDE_ANTIGEN_VIA_MHC_CLASS_I | 78 | -2,779 | 0,0001 | Low_in_IC25 |
| GO_MITOTIC_DNA_INTEGRITY_CHECKPOINT | 94 | -2,77 | 0,0001 | Low_in_IC25 |
| GO_REGULATION_OF_DNA_REPLICATION | 134 | -2,769 | 0,0001 | Low_in_IC25 |
| GO_REGULATION_OF_SISTER_CHROMATID_SEGREGATION | 61 | -2,761 | 0,0002 | Low_in_IC25 |
| GO_PROTEIN_LOCALIZATION_TO_MITOCHONDRION | 62 | -2,757 | 0,0002 | Low_in_IC25 |
| GO_RNA_PHOSPHODIESTER_BOND_HYDROLYSIS | 104 | -2,753 | 0,0002 | Low_in_IC25 |
| GO_POSITIVE_REGULATION_OF_VIRAL_TRANSCRIPTION | 37 | -2,748 | 0,0002 | Low_in_IC25 |
| GO_POSITIVE_REGULATION_OF_DNA_METABOLIC_PROCESS | 148 | -2,739 | 0,0002 | Low_in_IC25 |
| GO_RNA_PHOSPHODIESTER_BOND_HYDROLYSIS_ENDONUCLEOLYTIC | 50 | -2,729 | 0,0002 | Low_in_IC25 |
| GO_NEGATIVE_REGULATION_OF_CANONICAL_WNT_SIGNALING_PATHWAY | 134 | -2,709 | 0,0002 | Low_in_IC25 |
| GO_DNA_GEOMETRIC_CHANGE | 78 | -2,695 | 0,0002 | Low_in_IC25 |
| GO_PROTEIN_TRANSMEMBRANE_TRANSPORT | 42 | -2,694 | 0,0002 | Low_in_IC25 |
| GO_TRANSCRIPTION_ELONGATION_FROM_RNA_POLYMERASE_II_PROMOTER | 78 | -2,684 | 0,0003 | Low_in_IC25 |
| GO_NUCLEOTIDE_EXCISION_REPAIR_DNA_INCISION | 37 | -2,681 | 0,0003 | Low_in_IC25 |
| GO_REGULATION_OF_GENE_EXPRESSION_EPIGENETIC | 191 | -2,676 | 0,0003 | Low_in_IC25 |
| GO_REGULATION_OF_PROTEIN_CATABOLIC_PROCESS | 346 | -2,672 | 0,0003 | Low_in_IC25 |
| GO_RNA_CAPPING | 37 | -2,669 | 0,0003 | Low_in_IC25 |
| GO_COENZYME_BIOSYNTHETIC_PROCESS | 106 | -2,665 | 0,0003 | Low_in_IC25 |
| GO_POSITIVE_REGULATION_OF_MRNA_PROCESSING | 28 | -2,653 | 0,0003 | Low_in_IC25 |
| GO_REGULATION_OF_MRNA_SPLICING_VIA_SPLICEOSOME | 38 | -2,649 | 0,0003 | Low_in_IC25 |
| GO_ERROR_FREE_TRANSLESION_SYNTHESIS | 19 | -2,642 | 0,0004 | Low_in_IC25 |
| GO_HYDROGEN_ION_TRANSMEMBRANE_TRANSPORT | 79 | -2,638 | 0,0004 | Low_in_IC25 |
| GO_POSITIVE_REGULATION_OF_CHROMATIN_MODIFICATION | 74 | -2,633 | 0,0004 | Low_in_IC25 |
| GO_NUCLEOSIDE_TRIPHOSPHATE_BIOSYNTHETIC_PROCESS | 52 | -2,63 | 0,0004 | Low_in_IC25 |
| GO_OXIDOREDUCTION_COENZYME_METABOLIC_PROCESS | 89 | -2,626 | 0,0004 | Low_in_IC25 |
| GO_POSITIVE_REGULATION_OF_MITOTIC_CELL_CYCLE | 105 | -2,621 | 0,0004 | Low_in_IC25 |
| GO_ANTIGEN_PROCESSING_AND_PRESENTATION_OF_PEPTIDE_ANTIGEN | 143 | -2,611 | 0,0004 | Low_in_IC25 |
| GO_RIBONUCLEOSIDE_TRIPHOSPHATE_BIOSYNTHETIC_PROCESS | 44 | -2,61 | 0,0004 | Low_in_IC25 |
| GO_REGULATION_OF_CELLULAR_KETONE_METABOLIC_PROCESS | 128 | -2,602 | 0,0005 | Low_in_IC25 |
| GO_REGULATION_OF_RNA_SPLICING | 65 | -2,6 | 0,0005 | Low_in_IC25 |
| GO_CENTROMERE_COMPLEX_ASSEMBLY | 41 | -2,599 | 0,0005 | Low_in_IC25 |
| GO_CELL_PROLIFERATION | 497 | -2,548 | 0,0007 | Low_in_IC25 |
| GO_DNA_REPLICATION_DEPENDENT_NUCLEOSOME_ORGANIZATION | 28 | -2,533 | 0,0008 | Low_in_IC25 |
| GO_VIRAL_LIFE_CYCLE | 258 | -2,526 | 0,0008 | Low_in_IC25 |
| GO_CELLULAR_ALDEHYDE_METABOLIC_PROCESS | 66 | -2,523 | 0,0008 | Low_in_IC25 |
| GO_HYDROGEN_TRANSPORT | 102 | -2,507 | 0,0009 | Low_in_IC25 |
| GO_REGULATION_OF_CELL_CYCLE_CHECKPOINT | 26 | -2,506 | 0,0009 | Low_in_IC25 |
| GO_NEGATIVE_REGULATION_OF_TRANSFERASE_ACTIVITY | 288 | -2,505 | 0,0009 | Low_in_IC25 |
| GO_MEIOTIC_CHROMOSOME_SEGREGATION | 36 | -2,497 | 0,001 | Low_in_IC25 |
| GO_NON_RECOMBINATIONAL_REPAIR | 60 | -2,492 | 0,001 | Low_in_IC25 |
| GO_CHROMATIN_REMODELING | 130 | -2,491 | 0,001 | Low_in_IC25 |
| GO_POSITIVE_REGULATION_OF_MRNA_METABOLIC_PROCESS | 39 | -2,483 | 0,001 | Low_in_IC25 |
| GO_POSITIVE_REGULATION_OF_DNA_BIOSYNTHETIC_PROCESS | 50 | -2,48 | 0,001 | Low_in_IC25 |
| GO_PROTEIN_EXPORT_FROM_NUCLEUS | 28 | -2,476 | 0,001 | Low_in_IC25 |
| GO_METHYLATION | 218 | -2,464 | 0,001 | Low_in_IC25 |
| GO_SIGNAL_TRANSDUCTION_IN_RESPONSE_TO_DNA_DAMAGE | 88 | -2,452 | 0,001 | Low_in_IC25 |
| GO_POSITIVE_REGULATION_OF_ORGANELLE_ORGANIZATION | 476 | -2,447 | 0,001 | Low_in_IC25 |
| GO_NEGATIVE_REGULATION_OF_CELL_CYCLE_G1_S_PHASE_TRANSITION | 91 | -2,436 | 0,001 | Low_in_IC25 |
| GO_ERROR_PRONE_TRANSLESION_SYNTHESIS | 17 | -2,43 | 0,001 | Low_in_IC25 |
| GO_MICROTUBULE_BASED_PROCESS | 406 | -2,429 | 0,001 | Low_in_IC25 |
| GO_STRAND_DISPLACEMENT | 21 | -2,414 | 0,002 | Low_in_IC25 |
| GO_RECOMBINATIONAL_REPAIR | 62 | -2,413 | 0,002 | Low_in_IC25 |
| GO_RIBOSOMAL_SMALL_SUBUNIT_BIOGENESIS | 55 | -2,413 | 0,002 | Low_in_IC25 |
| GO_NUCLEAR_TRANSCRIBED_MRNA_CATABOLIC_PROCESS_DEADENYLATION_DEPENDENT_DECAY | 54 | -2,41 | 0,002 | Low_in_IC25 |
| GO_REGULATION_OF_SULFUR_METABOLIC_PROCESS | 17 | -2,41 | 0,002 | Low_in_IC25 |
| GO_NEGATIVE_REGULATION_OF_WNT_SIGNALING_PATHWAY | 161 | -2,407 | 0,002 | Low_in_IC25 |
| GO_PROTEASOMAL_PROTEIN_CATABOLIC_PROCESS | 243 | -2,407 | 0,002 | Low_in_IC25 |
| GO_REGULATION_OF_TYPE_I_INTERFERON_PRODUCTION | 100 | -2,403 | 0,002 | Low_in_IC25 |
| GO_NITROGEN_COMPOUND_TRANSPORT | 362 | -2,4 | 0,002 | Low_in_IC25 |
| GO_CHROMATIN_SILENCING_AT_RDNA | 33 | -2,4 | 0,002 | Low_in_IC25 |
| GO_REGULATION_OF_DNA_BIOSYNTHETIC_PROCESS | 76 | -2,398 | 0,002 | Low_in_IC25 |
| GO_RESPONSE_TO_STEROID_HORMONE | 348 | -2,396 | 0,002 | Low_in_IC25 |
| GO_INNATE_IMMUNE_RESPONSE_ACTIVATING_CELL_SURFACE_RECEPTOR_SIGNALING_PATHWAY | 92 | -2,393 | 0,002 | Low_in_IC25 |
| GO_CHROMOSOME_CONDENSATION | 24 | -2,393 | 0,002 | Low_in_IC25 |
| GO_REGULATION_OF_DNA_TEMPLATED_TRANSCRIPTION_ELONGATION | 38 | -2,391 | 0,002 | Low_in_IC25 |
| GO_REGULATION_OF_TRANSLATIONAL_INITIATION | 73 | -2,383 | 0,002 | Low_in_IC25 |
| GO_NCRNA_TRANSCRIPTION | 86 | -2,383 | 0,002 | Low_in_IC25 |
| GO_RESPONSE_TO_INTERFERON_BETA | 19 | -2,382 | 0,002 | Low_in_IC25 |
| GO_REGULATION_OF_ALTERNATIVE_MRNA_SPLICING_VIA_SPLICEOSOME | 18 | -2,377 | 0,002 | Low_in_IC25 |
| GO_TRANSLATIONAL_INITIATION | 142 | -2,371 | 0,002 | Low_in_IC25 |
| GO_NUCLEOBASE_BIOSYNTHETIC_PROCESS | 17 | -2,368 | 0,002 | Low_in_IC25 |
| GO_NUCLEUS_ORGANIZATION | 114 | -2,363 | 0,002 | Low_in_IC25 |
| GO_ORGANOPHOSPHATE_BIOSYNTHETIC_PROCESS | 362 | -2,356 | 0,002 | Low_in_IC25 |
| GO_POSITIVE_REGULATION_OF_DNA_TEMPLATED_TRANSCRIPTION_ELONGATION | 22 | -2,352 | 0,002 | Low_in_IC25 |
| GO_SPINDLE_CHECKPOINT | 23 | -2,349 | 0,002 | Low_in_IC25 |
| GO_CHROMOSOME_SEPARATION | 16 | -2,344 | 0,003 | Low_in_IC25 |
| GO_CHROMATIN_SILENCING | 72 | -2,343 | 0,003 | Low_in_IC25 |
| GO_INTERSTRAND_CROSS_LINK_REPAIR | 36 | -2,327 | 0,003 | Low_in_IC25 |
| GO_REGULATION_OF_MRNA_3_END_PROCESSING | 28 | -2,316 | 0,003 | Low_in_IC25 |
| GO_DNA_DEPENDENT_DNA_REPLICATION_MAINTENANCE_OF_FIDELITY | 19 | -2,315 | 0,003 | Low_in_IC25 |
| GO_REGULATION_OF_DNA_DEPENDENT_DNA_REPLICATION | 38 | -2,297 | 0,003 | Low_in_IC25 |
| GO_BASE_EXCISION_REPAIR | 38 | -2,297 | 0,003 | Low_in_IC25 |
| GO_POSITIVE_REGULATION_OF_TELOMERE_MAINTENANCE_VIA_TELOMERE_LENGTHENING | 30 | -2,296 | 0,003 | Low_in_IC25 |
| GO_POSITIVE_REGULATION_OF_DNA_REPAIR | 33 | -2,293 | 0,004 | Low_in_IC25 |
| GO_HISTONE_EXCHANGE | 43 | -2,291 | 0,004 | Low_in_IC25 |
| GO_MITOCHONDRIAL_MEMBRANE_ORGANIZATION | 87 | -2,289 | 0,004 | Low_in_IC25 |
| GO_NUCLEAR_TRANSCRIBED_MRNA_CATABOLIC_PROCESS_NONSENSE_MEDIATED_DECAY | 113 | -2,286 | 0,004 | Low_in_IC25 |
| GO_FC_EPSILON_RECEPTOR_SIGNALING_PATHWAY | 113 | -2,277 | 0,004 | Low_in_IC25 |
| GO_NEGATIVE_REGULATION_OF_CELLULAR_AMIDE_METABOLIC_PROCESS | 110 | -2,277 | 0,004 | Low_in_IC25 |
| GO_POSITIVE_REGULATION_OF_CELL_DIVISION | 94 | -2,276 | 0,004 | Low_in_IC25 |
| GO_WNT_SIGNALING_PATHWAY | 292 | -2,273 | 0,004 | Low_in_IC25 |
| GO_POSITIVE_REGULATION_OF_CANONICAL_WNT_SIGNALING_PATHWAY | 104 | -2,271 | 0,004 | Low_in_IC25 |
| GO_REGULATION_OF_CENTROSOME_DUPLICATION | 28 | -2,269 | 0,004 | Low_in_IC25 |
| GO_NEGATIVE_REGULATION_OF_NUCLEAR_DIVISION | 39 | -2,266 | 0,004 | Low_in_IC25 |
| GO_NEGATIVE_REGULATION_OF_MITOTIC_NUCLEAR_DIVISION | 31 | -2,265 | 0,004 | Low_in_IC25 |
| GO_POSITIVE_REGULATION_OF_GENE_EXPRESSION_EPIGENETIC | 72 | -2,264 | 0,004 | Low_in_IC25 |
| GO_T_CELL_RECEPTOR_SIGNALING_PATHWAY | 112 | -2,261 | 0,004 | Low_in_IC25 |
| GO_ANTIGEN_RECEPTOR_MEDIATED_SIGNALING_PATHWAY | 130 | -2,26 | 0,004 | Low_in_IC25 |
| GO_TRANSCRIPTION_FROM_RNA_POLYMERASE_I_PROMOTER | 35 | -2,259 | 0,004 | Low_in_IC25 |
| GO_REGULATION_OF_MEGAKARYOCYTE_DIFFERENTIATION | 17 | -2,256 | 0,004 | Low_in_IC25 |
| GO_NEGATIVE_REGULATION_OF_TYPE_I_INTERFERON_PRODUCTION | 37 | -2,256 | 0,004 | Low_in_IC25 |
| GO_NEGATIVE_REGULATION_OF_CYTOSKELETON_ORGANIZATION | 185 | -2,254 | 0,004 | Low_in_IC25 |
| GO_MICROTUBULE_ORGANIZING_CENTER_ORGANIZATION | 73 | -2,244 | 0,005 | Low_in_IC25 |
| GO_NEGATIVE_REGULATION_OF_CELL_DIVISION | 51 | -2,243 | 0,005 | Low_in_IC25 |
| GO_G1_DNA_DAMAGE_CHECKPOINT | 68 | -2,242 | 0,005 | Low_in_IC25 |
| GO_SPINDLE_ASSEMBLY | 59 | -2,241 | 0,005 | Low_in_IC25 |
| GO_POSITIVE_REGULATION_OF_TYPE_I_INTERFERON_PRODUCTION | 63 | -2,241 | 0,005 | Low_in_IC25 |
| GO_MITOCHONDRIAL_FUSION | 19 | -2,241 | 0,005 | Low_in_IC25 |
| GO_BETA_CATENIN_TCF_COMPLEX_ASSEMBLY | 35 | -2,239 | 0,005 | Low_in_IC25 |
| GO_REGULATION_OF_CHROMATIN_ORGANIZATION | 126 | -2,234 | 0,005 | Low_in_IC25 |
| GO_RESPONSE_TO_INTERFERON_ALPHA | 17 | -2,222 | 0,005 | Low_in_IC25 |
| GO_REGULATION_OF_MULTI_ORGANISM_PROCESS | 370 | -2,222 | 0,005 | Low_in_IC25 |
| GO_PURINE_NUCLEOBASE_METABOLIC_PROCESS | 18 | -2,217 | 0,005 | Low_in_IC25 |
| GO_PROTEIN_LOCALIZATION_TO_NUCLEUS | 130 | -2,216 | 0,005 | Low_in_IC25 |
| GO_POSITIVE_REGULATION_OF_WNT_SIGNALING_PATHWAY | 127 | -2,215 | 0,005 | Low_in_IC25 |
| GO_REGULATION_OF_DNA_REPAIR | 65 | -2,21 | 0,006 | Low_in_IC25 |
| GO_REGULATION_OF_TELOMERE_MAINTENANCE_VIA_TELOMERE_LENGTHENING | 45 | -2,209 | 0,006 | Low_in_IC25 |
| GO_REGULATION_OF_TRANSCRIPTION_FROM_RNA_POLYMERASE_I_PROMOTER | 21 | -2,2 | 0,006 | Low_in_IC25 |
| GO_REGULATION_OF_GLUCOSE_TRANSPORT | 80 | -2,199 | 0,006 | Low_in_IC25 |
| GO_REGULATION_OF_PROTEASOMAL_PROTEIN_CATABOLIC_PROCESS | 164 | -2,198 | 0,006 | Low_in_IC25 |
| GO_NUCLEAR_TRANSCRIBED_MRNA_CATABOLIC_PROCESS_EXONUCLEOLYTIC | 30 | -2,198 | 0,006 | Low_in_IC25 |
| GO_ANTIGEN_PROCESSING_AND_PRESENTATION | 171 | -2,193 | 0,006 | Low_in_IC25 |
| GO_TUMOR_NECROSIS_FACTOR_MEDIATED_SIGNALING_PATHWAY | 99 | -2,19 | 0,006 | Low_in_IC25 |
| GO_REGULATION_OF_HEMATOPOIETIC_PROGENITOR_CELL_DIFFERENTIATION | 27 | -2,188 | 0,006 | Low_in_IC25 |
| GO_DNA_LIGATION | 16 | -2,183 | 0,006 | Low_in_IC25 |
| GO_SNRNA_METABOLIC_PROCESS | 80 | -2,181 | 0,007 | Low_in_IC25 |
| GO_RESPONSE_TO_IONIZING_RADIATION | 128 | -2,18 | 0,007 | Low_in_IC25 |
| GO_REGULATION_OF_TELOMERE_MAINTENANCE | 59 | -2,178 | 0,007 | Low_in_IC25 |
| GO_NEGATIVE_REGULATION_OF_GENE_EXPRESSION_EPIGENETIC | 89 | -2,169 | 0,007 | Low_in_IC25 |
| GO_BLASTOCYST_DEVELOPMENT | 49 | -2,166 | 0,007 | Low_in_IC25 |
| GO_STEM_CELL_PROLIFERATION | 40 | -2,163 | 0,007 | Low_in_IC25 |
| GO_NCRNA_3_END_PROCESSING | 20 | -2,163 | 0,007 | Low_in_IC25 |
| GO_MACROMOLECULE_METHYLATION | 163 | -2,162 | 0,007 | Low_in_IC25 |
| GO_PROTEIN_FOLDING | 186 | -2,16 | 0,007 | Low_in_IC25 |
| GO_MISMATCH_REPAIR | 28 | -2,153 | 0,008 | Low_in_IC25 |
| GO_POSITIVE_REGULATION_OF_CHROMOSOME_SEGREGATION | 24 | -2,153 | 0,008 | Low_in_IC25 |
| GO_REGULATION_OF_INNATE_IMMUNE_RESPONSE | 275 | -2,15 | 0,008 | Low_in_IC25 |
| GO_MICROTUBULE_CYTOSKELETON_ORGANIZATION | 273 | -2,147 | 0,008 | Low_in_IC25 |
| GO_MATURATION_OF_SSU_RRNA | 39 | -2,145 | 0,008 | Low_in_IC25 |
| GO_RESPONSE_TO_RADIATION | 326 | -2,144 | 0,008 | Low_in_IC25 |
| GO_PROTEIN_TETRAMERIZATION | 105 | -2,142 | 0,008 | Low_in_IC25 |
| GO_POSITIVE_REGULATION_OF_CELL_CYCLE_ARREST | 78 | -2,139 | 0,008 | Low_in_IC25 |
| GO_POSITIVE_REGULATION_OF_NUCLEAR_DIVISION | 47 | -2,137 | 0,008 | Low_in_IC25 |
| GO_TRICARBOXYLIC_ACID_METABOLIC_PROCESS | 34 | -2,136 | 0,008 | Low_in_IC25 |
| GO_POSITIVE_REGULATION_OF_CELL_CYCLE_PHASE_TRANSITION | 62 | -2,134 | 0,008 | Low_in_IC25 |
| GO_PROTEIN_LOCALIZATION_TO_CHROMOSOME | 37 | -2,134 | 0,008 | Low_in_IC25 |
| GO_NEGATIVE_REGULATION_OF_PROTEIN_COMPLEX_DISASSEMBLY | 142 | -2,12 | 0,009 | Low_in_IC25 |
| GO_PROTEIN_TRANSPORT_ALONG_MICROTUBULE | 23 | -2,118 | 0,009 | Low_in_IC25 |
| GO_POSITIVE_REGULATION_OF_CATABOLIC_PROCESS | 344 | -2,099 | 0,01 | Low_in_IC25 |
| GO_PROTEIN_HETEROTETRAMERIZATION | 31 | -2,095 | 0,01 | Low_in_IC25 |
| GO_NEGATIVE_REGULATION_OF_CHROMOSOME_SEGREGATION | 25 | -2,095 | 0,01 | Low_in_IC25 |
| GO_SIGNAL_TRANSDUCTION_BY_P53_CLASS_MEDIATOR | 114 | -2,094 | 0,01 | Low_in_IC25 |
| GO_QUINONE_METABOLIC_PROCESS | 26 | -2,084 | 0,01 | Low_in_IC25 |
| GO_REGULATION_OF_PROTEASOMAL_UBIQUITIN_DEPENDENT_PROTEIN_CATABOLIC_PROCESS | 134 | -2,084 | 0,01 | Low_in_IC25 |
| GO_FEMALE_MEIOTIC_DIVISION | 20 | -2,073 | 0,01 | Low_in_IC25 |
| GO_POSITIVE_REGULATION_OF_TELOMERE_MAINTENANCE | 41 | -2,071 | 0,01 | Low_in_IC25 |
| GO_TONGUE_DEVELOPMENT | 16 | -2,069 | 0,01 | Low_in_IC25 |
| GO_METAPHASE_PLATE_CONGRESSION | 38 | -2,065 | 0,01 | Low_in_IC25 |
| GO_FIBROBLAST_GROWTH_FACTOR_RECEPTOR_SIGNALING_PATHWAY | 61 | -2,055 | 0,01 | Low_in_IC25 |
| GO_REGULATION_OF_CANONICAL_WNT_SIGNALING_PATHWAY | 194 | -2,053 | 0,01 | Low_in_IC25 |
| GO_REGULATION_OF_CELL_CYCLE_ARREST | 97 | -2,052 | 0,01 | Low_in_IC25 |
| GO_INNER_MITOCHONDRIAL_MEMBRANE_ORGANIZATION | 17 | -2,052 | 0,01 | Low_in_IC25 |
| GO_MATURATION_OF_5_8S_RRNA_FROM_TRICISTRONIC_RRNA_TRANSCRIPT_SSU_RRNA_5_8S_RRNA_LSU_RRNA_ | 19 | -2,051 | 0,01 | Low_in_IC25 |
| GO_CHROMOSOME_LOCALIZATION | 51 | -2,047 | 0,01 | Low_in_IC25 |
| GO_REGULATION_OF_MITOCHONDRION_ORGANIZATION | 190 | -2,037 | 0,01 | Low_in_IC25 |
| GO_POSITIVE_REGULATION_OF_MITOCHONDRION_ORGANIZATION | 144 | -2,036 | 0,01 | Low_in_IC25 |
| GO_BINDING_OF_SPERM_TO_ZONA_PELLUCIDA | 20 | -2,035 | 0,01 | Low_in_IC25 |
| GO_REGULATION_OF_SYMBIOSIS_ENCOMPASSING_MUTUALISM_THROUGH_PARASITISM | 175 | -2,031 | 0,01 | Low_in_IC25 |
| GO_REGULATION_OF_TYPE_I_INTERFERON_MEDIATED_SIGNALING_PATHWAY | 26 | -2,031 | 0,01 | Low_in_IC25 |
| GO_AGING | 194 | -2,022 | 0,02 | Low_in_IC25 |
| GO_POSITIVE_REGULATION_OF_CELLULAR_AMIDE_METABOLIC_PROCESS | 96 | -2,021 | 0,02 | Low_in_IC25 |
| GO_REGULATION_OF_PROTEIN_STABILITY | 191 | -2,021 | 0,02 | Low_in_IC25 |
| GO_RNA_POLYADENYLATION | 27 | -2,019 | 0,02 | Low_in_IC25 |
| GO_NEGATIVE_REGULATION_OF_MRNA_METABOLIC_PROCESS | 27 | -2,017 | 0,02 | Low_in_IC25 |
| GO_LIPID_OXIDATION | 53 | -2,013 | 0,02 | Low_in_IC25 |
| GO_PROTEIN_POLYUBIQUITINATION | 229 | -2,01 | 0,02 | Low_in_IC25 |
| GO_NUCLEAR_PORE_ORGANIZATION | 15 | -2,009 | 0,02 | Low_in_IC25 |
| GO_RIBOSOMAL_LARGE_SUBUNIT_BIOGENESIS | 48 | -2,008 | 0,02 | Low_in_IC25 |
| GO_DEFENSE_RESPONSE_TO_VIRUS | 120 | -2,008 | 0,02 | Low_in_IC25 |
| GO_NEGATIVE_REGULATION_OF_DNA_METABOLIC_PROCESS | 93 | -2,006 | 0,02 | Low_in_IC25 |
| GO_NEGATIVE_REGULATION_OF_CELLULAR_PROTEIN_CATABOLIC_PROCESS | 53 | -2,005 | 0,02 | Low_in_IC25 |
| GO_ENTRAINMENT_OF_CIRCADIAN_CLOCK | 23 | -1,998 | 0,02 | Low_in_IC25 |
| GO_OSTEOBLAST_DIFFERENTIATION | 107 | -1,998 | 0,02 | Low_in_IC25 |
| GO_POSITIVE_REGULATION_OF_DNA_REPLICATION | 69 | -1,993 | 0,02 | Low_in_IC25 |
| GO_CHROMATIN_DISASSEMBLY | 15 | -1,991 | 0,02 | Low_in_IC25 |
| GO_KETONE_BIOSYNTHETIC_PROCESS | 20 | -1,988 | 0,02 | Low_in_IC25 |
| GO_CELLULAR_KETONE_METABOLIC_PROCESS | 57 | -1,985 | 0,02 | Low_in_IC25 |
| GO_REGULATION_OF_PRI_MIRNA_TRANSCRIPTION_FROM_RNA_POLYMERASE_II_PROMOTER | 15 | -1,983 | 0,02 | Low_in_IC25 |
| GO_PHOTOPERIODISM | 20 | -1,981 | 0,02 | Low_in_IC25 |
| GO_TRANSCRIPTION_INITIATION_FROM_RNA_POLYMERASE_II_PROMOTER | 124 | -1,979 | 0,02 | Low_in_IC25 |
| GO_POSITIVE_REGULATION_OF_TRANSCRIPTION_INITIATION_FROM_RNA_POLYMERASE_II_PROMOTER | 16 | -1,976 | 0,02 | Low_in_IC25 |
| GO_MITOCHONDRIAL_RNA_METABOLIC_PROCESS | 26 | -1,974 | 0,02 | Low_in_IC25 |
| GO_POSITIVE_REGULATION_OF_MITOTIC_NUCLEAR_DIVISION | 39 | -1,968 | 0,02 | Low_in_IC25 |
| GO_XENOPHAGY | 82 | -1,965 | 0,02 | Low_in_IC25 |
| GO_BLASTOCYST_FORMATION | 23 | -1,96 | 0,02 | Low_in_IC25 |
| GO_MITOTIC_SPINDLE_ORGANIZATION | 62 | -1,948 | 0,02 | Low_in_IC25 |
| GO_REGULATION_OF_CIRCADIAN_RHYTHM | 78 | -1,947 | 0,02 | Low_in_IC25 |
| GO_SOMATIC_DIVERSIFICATION_OF_IMMUNE_RECEPTORS | 35 | -1,947 | 0,02 | Low_in_IC25 |
| GO_RNA_METHYLATION | 45 | -1,942 | 0,02 | Low_in_IC25 |
| GO_REGULATION_OF_VIRAL_TRANSCRIPTION | 58 | -1,936 | 0,02 | Low_in_IC25 |
| GO_HISTONE_UBIQUITINATION | 32 | -1,934 | 0,02 | Low_in_IC25 |
| GO_NUCLEAR_TRANSCRIBED_MRNA_POLY_A_TAIL_SHORTENING | 25 | -1,916 | 0,03 | Low_in_IC25 |
| GO_NEGATIVE_REGULATION_OF_HEMATOPOIETIC_PROGENITOR_CELL_DIFFERENTIATION | 18 | -1,913 | 0,03 | Low_in_IC25 |
| GO_ANATOMICAL_STRUCTURE_HOMEOSTASIS | 208 | -1,91 | 0,03 | Low_in_IC25 |
| GO_CLEAVAGE_INVOLVED_IN_RRNA_PROCESSING | 18 | -1,907 | 0,03 | Low_in_IC25 |
| GO_REGULATION_OF_DNA_BINDING | 76 | -1,905 | 0,03 | Low_in_IC25 |
| GO_PIGMENT_BIOSYNTHETIC_PROCESS | 38 | -1,904 | 0,03 | Low_in_IC25 |
| GO_MAINTENANCE_OF_CELL_NUMBER | 106 | -1,904 | 0,03 | Low_in_IC25 |
| GO_RESPONSE_TO_VIRUS | 187 | -1,903 | 0,03 | Low_in_IC25 |
| GO_NEGATIVE_REGULATION_OF_CELL_AGING | 15 | -1,9 | 0,03 | Low_in_IC25 |
| GO_REGULATION_OF_CHOLESTEROL_METABOLIC_PROCESS | 16 | -1,898 | 0,03 | Low_in_IC25 |
| GO_TOXIN_TRANSPORT | 32 | -1,896 | 0,03 | Low_in_IC25 |
| GO_AXIS_ELONGATION | 20 | -1,893 | 0,03 | Low_in_IC25 |
| GO_POSITIVE_REGULATION_OF_TRANSLATIONAL_INITIATION | 19 | -1,892 | 0,03 | Low_in_IC25 |
| GO_REGULATION_OF_DEFENSE_RESPONSE_TO_VIRUS_BY_HOST | 104 | -1,888 | 0,03 | Low_in_IC25 |
| GO_NEGATIVE_REGULATION_OF_RNA_SPLICING | 17 | -1,885 | 0,03 | Low_in_IC25 |
| GO_PROTEIN_REFOLDING | 20 | -1,883 | 0,03 | Low_in_IC25 |
| GO_REGULATION_OF_PROTEIN_COMPLEX_DISASSEMBLY | 185 | -1,878 | 0,03 | Low_in_IC25 |
| GO_POSITIVE_REGULATION_OF_DNA_TEMPLATED_TRANSCRIPTION_INITIATION | 23 | -1,873 | 0,03 | Low_in_IC25 |
| GO_POSITIVE_REGULATION_OF_MRNA_3_END_PROCESSING | 17 | -1,872 | 0,03 | Low_in_IC25 |
| GO_REGULATION_OF_DOUBLE_STRAND_BREAK_REPAIR | 35 | -1,871 | 0,03 | Low_in_IC25 |
| GO_DEFENSE_RESPONSE_TO_OTHER_ORGANISM | 308 | -1,869 | 0,03 | Low_in_IC25 |
| GO_RRNA_TRANSCRIPTION | 18 | -1,869 | 0,03 | Low_in_IC25 |
| GO_BETA_CATENIN_DESTRUCTION_COMPLEX_DISASSEMBLY | 17 | -1,868 | 0,03 | Low_in_IC25 |
| GO_ENTRAINMENT_OF_CIRCADIAN_CLOCK_BY_PHOTOPERIOD | 17 | -1,867 | 0,03 | Low_in_IC25 |
| GO_RESPONSE_TO_UV | 117 | -1,867 | 0,03 | Low_in_IC25 |
| GO_REGULATION_OF_PROTEIN_LOCALIZATION_TO_NUCLEUS | 173 | -1,864 | 0,03 | Low_in_IC25 |
| GO_REGULATION_OF_WNT_SIGNALING_PATHWAY | 247 | -1,864 | 0,03 | Low_in_IC25 |
| GO_RESPONSE_TO_THYROID_HORMONE | 15 | -1,856 | 0,03 | Low_in_IC25 |
| GO_REGULATION_OF_TRANSCRIPTION_INITIATION_FROM_RNA_POLYMERASE_II_PROMOTER | 21 | -1,854 | 0,03 | Low_in_IC25 |
| GO_PROTEIN_STABILIZATION | 113 | -1,847 | 0,04 | Low_in_IC25 |
| GO_PIGMENT_METABOLIC_PROCESS | 44 | -1,841 | 0,04 | Low_in_IC25 |
| GO_CELLULAR_MODIFIED_AMINO_ACID_BIOSYNTHETIC_PROCESS | 41 | -1,837 | 0,04 | Low_in_IC25 |
| GO_RRNA_MODIFICATION | 20 | -1,834 | 0,04 | Low_in_IC25 |
| GO_POSITIVE_REGULATION_OF_RNA_SPLICING | 20 | -1,834 | 0,04 | Low_in_IC25 |
| GO_CHROMOSOME_ORGANIZATION_INVOLVED_IN_MEIOTIC_CELL_CYCLE | 26 | -1,834 | 0,04 | Low_in_IC25 |
| GO_HISTONE_H4_ACETYLATION | 41 | -1,831 | 0,04 | Low_in_IC25 |
| GO_TRANSCRIPTION_FROM_RNA_POLYMERASE_III_PROMOTER | 40 | -1,827 | 0,04 | Low_in_IC25 |
| GO_REGULATION_OF_RESPONSE_TO_DNA_DAMAGE_STIMULUS | 127 | -1,825 | 0,04 | Low_in_IC25 |
| GO_NEGATIVE_REGULATION_OF_APOPTOTIC_SIGNALING_PATHWAY | 161 | -1,82 | 0,04 | Low_in_IC25 |
| GO_REGULATION_OF_HISTONE_METHYLATION | 42 | -1,817 | 0,04 | Low_in_IC25 |
| GO_NEGATIVE_REGULATION_OF_HISTONE_ACETYLATION | 15 | -1,804 | 0,04 | Low_in_IC25 |
| GO_NUCLEOBASE_METABOLIC_PROCESS | 33 | -1,803 | 0,04 | Low_in_IC25 |
| GO_POSITIVE_REGULATION_OF_INTERFERON_BETA_PRODUCTION | 24 | -1,803 | 0,04 | Low_in_IC25 |
| GO_REGULATION_OF_CHROMATIN_SILENCING | 18 | -1,802 | 0,04 | Low_in_IC25 |
| GO_PROTEIN_ACYLATION | 126 | -1,801 | 0,04 | Low_in_IC25 |
| GO_RESPONSE_TO_FIBROBLAST_GROWTH_FACTOR | 85 | -1,8 | 0,04 | Low_in_IC25 |
| GO_CYTOPLASMIC_TRANSLATION | 38 | -1,799 | 0,05 | Low_in_IC25 |
| GO_SOMATIC_DIVERSIFICATION_OF_IMMUNOGLOBULINS | 23 | -1,799 | 0,05 | Low_in_IC25 |
| GO_REGULATION_OF_TRANSLATIONAL_ELONGATION | 23 | -1,796 | 0,05 | Low_in_IC25 |
| GO_PROTEIN_ACETYLATION | 99 | -1,795 | 0,05 | Low_in_IC25 |
| GO_MRNA_SPLICE_SITE_SELECTION | 15 | -1,785 | 0,05 | Low_in_IC25 |
| GO_REGULATION_OF_GLYCOPROTEIN_METABOLIC_PROCESS | 35 | -1,782 | 0,05 | Low_in_IC25 |
| GO_MACROMOLECULE_DEACYLATION | 60 | -1,781 | 0,05 | Low_in_IC25 |
| GO_RECIPROCAL_DNA_RECOMBINATION | 30 | -1,777 | 0,05 | Low_in_IC25 |
