# Supplementary material for: Induction of ER and mitochondrial stress by the alkylphosphocholine erufosine in oral squamous cell carcinoma cells
Source: Cell Death Dis. 2018 Feb 20;9(3):296. doi: 10.1038/s41419-018-0342-2 (PMC5833417; doi:10.1038/s41419-018-0342-2)
Supplement: Supplementary file 12 — Supplementary Table 4c [file 41419_2018_342_MOESM12_ESM.docx]

Table S4c: Gene Ontology terms relating to positive enrichment of Biological processes at IC75 concentration of erufosine

| **Biological Processes** | **SIZE** | **Normalized Enrichment Score** | **FDR.q.val** | **TYPE** |
| --- | --- | --- | --- | --- |
| GO_REGULATION_OF_GTPASE_ACTIVITY | 494 | 4,031 | 0 | High_in_IC75 |
| GO_CELLULAR_RESPONSE_TO_TOPOLOGICALLY_INCORRECT_PROTEIN | 110 | 3,896 | 0 | High_in_IC75 |
| GO_GOLGI_VESICLE_TRANSPORT | 280 | 3,859 | 0 | High_in_IC75 |
| GO_RESPONSE_TO_ENDOPLASMIC_RETICULUM_STRESS | 209 | 3,834 | 0 | High_in_IC75 |
| GO_REGULATION_OF_CELL_ADHESION | 462 | 3,688 | 0 | High_in_IC75 |
| GO_ACTIN_FILAMENT_BASED_PROCESS | 337 | 3,659 | 0 | High_in_IC75 |
| GO_POSITIVE_REGULATION_OF_CELL_DEATH | 496 | 3,525 | 0 | High_in_IC75 |
| GO_ENDOMEMBRANE_SYSTEM_ORGANIZATION | 393 | 3,501 | 0 | High_in_IC75 |
| GO_ER_TO_GOLGI_VESICLE_MEDIATED_TRANSPORT | 142 | 3,469 | 0 | High_in_IC75 |
| GO_G_PROTEIN_COUPLED_RECEPTOR_SIGNALING_PATHWAY | 488 | 3,463 | 0 | High_in_IC75 |
| GO_RESPONSE_TO_BACTERIUM | 316 | 3,443 | 0 | High_in_IC75 |
| GO_REGULATION_OF_CELL_CELL_ADHESION | 265 | 3,414 | 0 | High_in_IC75 |
| GO_REGULATION_OF_CELL_ACTIVATION | 321 | 3,413 | 0 | High_in_IC75 |
| GO_IRE1_MEDIATED_UNFOLDED_PROTEIN_RESPONSE | 56 | 3,411 | 0 | High_in_IC75 |
| GO_RESPONSE_TO_TOPOLOGICALLY_INCORRECT_PROTEIN | 147 | 3,402 | 0 | High_in_IC75 |
| GO_NEGATIVE_REGULATION_OF_INTRACELLULAR_SIGNAL_TRANSDUCTION | 350 | 3,363 | 0 | High_in_IC75 |
| GO_CELL_JUNCTION_ORGANIZATION | 144 | 3,36 | 0 | High_in_IC75 |
| GO_POSITIVE_REGULATION_OF_LOCOMOTION | 312 | 3,355 | 0 | High_in_IC75 |
| GO_EPITHELIAL_CELL_DIFFERENTIATION | 351 | 3,327 | 0 | High_in_IC75 |
| GO_REGULATION_OF_RESPONSE_TO_WOUNDING | 276 | 3,292 | 0 | High_in_IC75 |
| GO_REGULATION_OF_MAPK_CASCADE | 481 | 3,274 | 0 | High_in_IC75 |
| GO_NEGATIVE_REGULATION_OF_CELL_CELL_ADHESION | 100 | 3,267 | 0 | High_in_IC75 |
| GO_NEGATIVE_REGULATION_OF_CELL_PROLIFERATION | 490 | 3,261 | 0,00005 | High_in_IC75 |
| GO_CELL_ACTIVATION | 396 | 3,194 | 0,00005 | High_in_IC75 |
| GO_RESPONSE_TO_MOLECULE_OF_BACTERIAL_ORIGIN | 226 | 3,18 | 0,00005 | High_in_IC75 |
| GO_MEMBRANE_BUDDING | 98 | 3,172 | 0,00004 | High_in_IC75 |
| GO_REGULATION_OF_CYTOKINE_PRODUCTION | 412 | 3,167 | 0,00004 | High_in_IC75 |
| GO_VASCULATURE_DEVELOPMENT | 351 | 3,153 | 0,00004 | High_in_IC75 |
| GO_SINGLE_ORGANISM_CELL_ADHESION | 327 | 3,152 | 0,00004 | High_in_IC75 |
| GO_CELLULAR_RESPONSE_TO_EXTERNAL_STIMULUS | 214 | 3,147 | 0,00004 | High_in_IC75 |
| GO_GOLGI_ORGANIZATION | 75 | 3,145 | 0,00004 | High_in_IC75 |
| GO_BLOOD_VESSEL_MORPHOGENESIS | 266 | 3,116 | 0,00007 | High_in_IC75 |
| GO_EPIDERMAL_GROWTH_FACTOR_RECEPTOR_SIGNALING_PATHWAY | 48 | 3,107 | 0,00007 | High_in_IC75 |
| GO_POSITIVE_REGULATION_OF_RESPONSE_TO_EXTERNAL_STIMULUS | 194 | 3,094 | 0,00007 | High_in_IC75 |
| GO_WOUND_HEALING | 345 | 3,077 | 0,00007 | High_in_IC75 |
| GO_SMALL_GTPASE_MEDIATED_SIGNAL_TRANSDUCTION | 297 | 3,068 | 0,0001 | High_in_IC75 |
| GO_NEGATIVE_REGULATION_OF_CELL_ACTIVATION | 111 | 3,044 | 0,0001 | High_in_IC75 |
| GO_REGULATION_OF_STRESS_ACTIVATED_PROTEIN_KINASE_SIGNALING_CASCADE | 164 | 3,013 | 0,0002 | High_in_IC75 |
| GO_SENSORY_PERCEPTION_OF_CHEMICAL_STIMULUS | 136 | 3,005 | 0,0002 | High_in_IC75 |
| GO_REGULATION_OF_T_CELL_PROLIFERATION | 95 | 2,997 | 0,0002 | High_in_IC75 |
| GO_NEGATIVE_REGULATION_OF_PHOSPHORUS_METABOLIC_PROCESS | 414 | 2,986 | 0,0002 | High_in_IC75 |
| GO_REGULATION_OF_CELL_MORPHOGENESIS | 418 | 2,976 | 0,0002 | High_in_IC75 |
| GO_POSITIVE_REGULATION_OF_KINASE_ACTIVITY | 373 | 2,967 | 0,0002 | High_in_IC75 |
| GO_REGULATION_OF_SMALL_GTPASE_MEDIATED_SIGNAL_TRANSDUCTION | 213 | 2,958 | 0,0002 | High_in_IC75 |
| GO_REGULATION_OF_CELL_PROJECTION_ORGANIZATION | 412 | 2,952 | 0,0002 | High_in_IC75 |
| GO_CELLULAR_RESPONSE_TO_CYTOKINE_STIMULUS | 435 | 2,923 | 0,0002 | High_in_IC75 |
| GO_POSITIVE_REGULATION_OF_PROTEIN_SERINE_THREONINE_KINASE_ACTIVITY | 227 | 2,922 | 0,0002 | High_in_IC75 |
| GO_VACUOLAR_TRANSPORT | 227 | 2,921 | 0,0002 | High_in_IC75 |
| GO_SKIN_DEVELOPMENT | 150 | 2,914 | 0,0002 | High_in_IC75 |
| GO_CELLULAR_RESPONSE_TO_EXTRACELLULAR_STIMULUS | 153 | 2,908 | 0,0002 | High_in_IC75 |
| GO_NEGATIVE_REGULATION_OF_PHOSPHORYLATION | 331 | 2,907 | 0,0002 | High_in_IC75 |
| GO_MORPHOGENESIS_OF_AN_EPITHELIUM | 304 | 2,898 | 0,0002 | High_in_IC75 |
| GO_LEUKOCYTE_ACTIVATION | 288 | 2,894 | 0,0002 | High_in_IC75 |
| GO_REGULATION_OF_FAT_CELL_DIFFERENTIATION | 80 | 2,884 | 0,0002 | High_in_IC75 |
| GO_REGULATION_OF_HOMOTYPIC_CELL_CELL_ADHESION | 209 | 2,874 | 0,0002 | High_in_IC75 |
| GO_RESPONSE_TO_PEPTIDE | 305 | 2,871 | 0,0002 | High_in_IC75 |
| GO_REGULATION_OF_ANATOMICAL_STRUCTURE_SIZE | 341 | 2,869 | 0,0002 | High_in_IC75 |
| GO_EPITHELIAL_CELL_DEVELOPMENT | 140 | 2,867 | 0,0002 | High_in_IC75 |
| GO_REGULATION_OF_LEUKOCYTE_PROLIFERATION | 133 | 2,861 | 0,0002 | High_in_IC75 |
| GO_KERATINOCYTE_DIFFERENTIATION | 70 | 2,855 | 0,0002 | High_in_IC75 |
| GO_REGULATION_OF_GROWTH | 462 | 2,848 | 0,0002 | High_in_IC75 |
| GO_POSITIVE_REGULATION_OF_STRESS_ACTIVATED_PROTEIN_KINASE_SIGNALING_CASCADE | 114 | 2,84 | 0,0002 | High_in_IC75 |
| GO_REGULATION_OF_INSULIN_RECEPTOR_SIGNALING_PATHWAY | 36 | 2,82 | 0,0003 | High_in_IC75 |
| GO_REGULATION_OF_CYTOKINE_BIOSYNTHETIC_PROCESS | 61 | 2,816 | 0,0004 | High_in_IC75 |
| GO_IN_UTERO_EMBRYONIC_DEVELOPMENT | 252 | 2,803 | 0,0004 | High_in_IC75 |
| GO_REGULATION_OF_AUTOPHAGY | 215 | 2,8 | 0,0004 | High_in_IC75 |
| GO_SENSORY_PERCEPTION | 377 | 2,799 | 0,0004 | High_in_IC75 |
| GO_REGULATION_OF_I_KAPPAB_KINASE_NF_KAPPAB_SIGNALING | 198 | 2,797 | 0,0004 | High_in_IC75 |
| GO_CELL_JUNCTION_ASSEMBLY | 102 | 2,792 | 0,0005 | High_in_IC75 |
| GO_REGULATION_OF_CELLULAR_COMPONENT_SIZE | 259 | 2,789 | 0,0004 | High_in_IC75 |
| GO_REGULATION_OF_APOPTOTIC_SIGNALING_PATHWAY | 301 | 2,789 | 0,0004 | High_in_IC75 |
| GO_REGULATION_OF_PROTEIN_SERINE_THREONINE_KINASE_ACTIVITY | 380 | 2,786 | 0,0004 | High_in_IC75 |
| GO_TAXIS | 307 | 2,783 | 0,0005 | High_in_IC75 |
| GO_REGULATION_OF_SECRETION | 463 | 2,765 | 0,0006 | High_in_IC75 |
| GO_NEGATIVE_REGULATION_OF_IMMUNE_SYSTEM_PROCESS | 269 | 2,765 | 0,0005 | High_in_IC75 |
| GO_NEGATIVE_REGULATION_OF_CELL_ADHESION | 170 | 2,755 | 0,0006 | High_in_IC75 |
| GO_REGULATION_OF_RAS_PROTEIN_SIGNAL_TRANSDUCTION | 142 | 2,748 | 0,0006 | High_in_IC75 |
| GO_POSITIVE_REGULATION_OF_CELL_ADHESION | 262 | 2,735 | 0,0006 | High_in_IC75 |
| GO_EMBRYONIC_PLACENTA_DEVELOPMENT | 70 | 2,732 | 0,0006 | High_in_IC75 |
| GO_REGULATION_OF_LIPASE_ACTIVITY | 54 | 2,73 | 0,0006 | High_in_IC75 |
| GO_ERBB_SIGNALING_PATHWAY | 70 | 2,702 | 0,0008 | High_in_IC75 |
| GO_PROTEIN_CATABOLIC_PROCESS | 484 | 2,699 | 0,0008 | High_in_IC75 |
| GO_REGULATION_OF_HEMOPOIESIS | 227 | 2,692 | 0,0008 | High_in_IC75 |
| GO_POSITIVE_REGULATION_OF_STAT_CASCADE | 49 | 2,69 | 0,0009 | High_in_IC75 |
| GO_ACTIN_FILAMENT_ORGANIZATION | 140 | 2,69 | 0,0008 | High_in_IC75 |
| GO_REGULATION_OF_CELL_GROWTH | 291 | 2,682 | 0,0009 | High_in_IC75 |
| GO_POSITIVE_REGULATION_OF_LIPASE_ACTIVITY | 40 | 2,682 | 0,0009 | High_in_IC75 |
| GO_NEGATIVE_REGULATION_OF_HOMOTYPIC_CELL_CELL_ADHESION | 73 | 2,67 | 0,001 | High_in_IC75 |
| GO_RESPONSE_TO_OXIDATIVE_STRESS | 287 | 2,665 | 0,001 | High_in_IC75 |
| GO_REGULATION_OF_PEPTIDYL_TYROSINE_PHOSPHORYLATION | 147 | 2,661 | 0,001 | High_in_IC75 |
| GO_REPRODUCTIVE_SYSTEM_DEVELOPMENT | 311 | 2,65 | 0,001 | High_in_IC75 |
| GO_SINGLE_ORGANISM_MEMBRANE_BUDDING | 61 | 2,648 | 0,001 | High_in_IC75 |
| GO_NEGATIVE_REGULATION_OF_KINASE_ACTIVITY | 200 | 2,648 | 0,001 | High_in_IC75 |
| GO_TISSUE_MORPHOGENESIS | 390 | 2,639 | 0,001 | High_in_IC75 |
| GO_RESPONSE_TO_WOUNDING | 408 | 2,634 | 0,001 | High_in_IC75 |
| GO_TUBE_DEVELOPMENT | 396 | 2,624 | 0,001 | High_in_IC75 |
| GO_REGULATION_OF_PROTEIN_KINASE_B_SIGNALING | 95 | 2,623 | 0,001 | High_in_IC75 |
| GO_GRANULOCYTE_ACTIVATION | 16 | 2,623 | 0,001 | High_in_IC75 |
| GO_REGULATION_OF_WOUND_HEALING | 89 | 2,619 | 0,001 | High_in_IC75 |
| GO_CELL_CELL_ADHESION | 400 | 2,616 | 0,001 | High_in_IC75 |
| GO_LEUKOCYTE_MIGRATION | 177 | 2,614 | 0,001 | High_in_IC75 |
| GO_CELLULAR_RESPONSE_TO_GLUCOSE_STARVATION | 27 | 2,593 | 0,002 | High_in_IC75 |
| GO_POSITIVE_REGULATION_OF_SECRETION | 253 | 2,59 | 0,002 | High_in_IC75 |
| GO_MYELOID_LEUKOCYTE_ACTIVATION | 65 | 2,586 | 0,002 | High_in_IC75 |
| GO_POSITIVE_REGULATION_OF_MAPK_CASCADE | 337 | 2,579 | 0,002 | High_in_IC75 |
| GO_POSITIVE_REGULATION_OF_I_KAPPAB_KINASE_NF_KAPPAB_SIGNALING | 148 | 2,567 | 0,002 | High_in_IC75 |
| GO_POSITIVE_REGULATION_OF_T_CELL_PROLIFERATION | 56 | 2,558 | 0,002 | High_in_IC75 |
| GO_TRANSMEMBRANE_RECEPTOR_PROTEIN_TYROSINE_KINASE_SIGNALING_PATHWAY | 391 | 2,557 | 0,002 | High_in_IC75 |
| GO_POSITIVE_REGULATION_OF_NF_KAPPAB_TRANSCRIPTION_FACTOR_ACTIVITY | 107 | 2,553 | 0,002 | High_in_IC75 |
| GO_CELL_MORPHOGENESIS_INVOLVED_IN_DIFFERENTIATION | 354 | 2,551 | 0,002 | High_in_IC75 |
| GO_REGULATION_OF_ACTIN_FILAMENT_BASED_PROCESS | 238 | 2,551 | 0,002 | High_in_IC75 |
| GO_IMMUNE_EFFECTOR_PROCESS | 326 | 2,551 | 0,002 | High_in_IC75 |
| GO_REGULATION_OF_INFLAMMATORY_RESPONSE | 191 | 2,545 | 0,002 | High_in_IC75 |
| GO_VASCULAR_ENDOTHELIAL_GROWTH_FACTOR_RECEPTOR_SIGNALING_PATHWAY | 64 | 2,543 | 0,002 | High_in_IC75 |
| GO_RESPONSE_TO_GROWTH_FACTOR | 353 | 2,538 | 0,002 | High_in_IC75 |
| GO_LENS_FIBER_CELL_DIFFERENTIATION | 18 | 2,535 | 0,002 | High_in_IC75 |
| GO_EPHRIN_RECEPTOR_SIGNALING_PATHWAY | 72 | 2,534 | 0,002 | High_in_IC75 |
| GO_CELLULAR_RESPONSE_TO_STARVATION | 97 | 2,532 | 0,002 | High_in_IC75 |
| GO_MUSCLE_STRUCTURE_DEVELOPMENT | 303 | 2,531 | 0,002 | High_in_IC75 |
| GO_REGULATION_OF_CELL_MATRIX_ADHESION | 72 | 2,527 | 0,002 | High_in_IC75 |
| GO_EMBRYO_DEVELOPMENT_ENDING_IN_BIRTH_OR_EGG_HATCHING | 423 | 2,52 | 0,002 | High_in_IC75 |
| GO_REGULATION_OF_MAP_KINASE_ACTIVITY | 246 | 2,519 | 0,002 | High_in_IC75 |
| GO_POSITIVE_REGULATION_OF_CELL_CELL_ADHESION | 164 | 2,519 | 0,002 | High_in_IC75 |
| GO_VESICLE_ORGANIZATION | 231 | 2,51 | 0,002 | High_in_IC75 |
| GO_CELL_SUBSTRATE_JUNCTION_ASSEMBLY | 34 | 2,508 | 0,002 | High_in_IC75 |
| GO_INFLAMMATORY_RESPONSE | 289 | 2,505 | 0,002 | High_in_IC75 |
| GO_POSITIVE_REGULATION_OF_CELLULAR_RESPONSE_TO_TRANSFORMING_GROWTH_FACTOR_BETA_STIMULUS | 21 | 2,505 | 0,002 | High_in_IC75 |
| GO_REGULATION_OF_INTERFERON_GAMMA_PRODUCTION | 56 | 2,504 | 0,002 | High_in_IC75 |
| GO_ANGIOGENESIS | 219 | 2,504 | 0,002 | High_in_IC75 |
| GO_REGULATION_OF_CELL_SHAPE | 106 | 2,499 | 0,002 | High_in_IC75 |
| GO_REGULATION_OF_VASCULATURE_DEVELOPMENT | 168 | 2,491 | 0,003 | High_in_IC75 |
| GO_REGULATION_OF_BODY_FLUID_LEVELS | 359 | 2,489 | 0,003 | High_in_IC75 |
| GO_POSITIVE_REGULATION_OF_CYTOKINE_BIOSYNTHETIC_PROCESS | 35 | 2,489 | 0,003 | High_in_IC75 |
| GO_REGULATION_OF_JNK_CASCADE | 131 | 2,488 | 0,003 | High_in_IC75 |
| GO_REGULATION_OF_ERAD_PATHWAY | 23 | 2,483 | 0,003 | High_in_IC75 |
| GO_ACTIN_FILAMENT_BUNDLE_ORGANIZATION | 44 | 2,478 | 0,003 | High_in_IC75 |
| GO_REGULATION_OF_VACUOLE_ORGANIZATION | 38 | 2,473 | 0,003 | High_in_IC75 |
| GO_REGULATION_OF_EXTRINSIC_APOPTOTIC_SIGNALING_PATHWAY | 122 | 2,468 | 0,003 | High_in_IC75 |
| GO_REGULATION_OF_RESPONSE_TO_EXTRACELLULAR_STIMULUS | 150 | 2,464 | 0,003 | High_in_IC75 |
| GO_DEVELOPMENTAL_PROCESS_INVOLVED_IN_REPRODUCTION | 431 | 2,463 | 0,003 | High_in_IC75 |
| GO_REGULATION_OF_TYROSINE_PHOSPHORYLATION_OF_STAT_PROTEIN | 45 | 2,462 | 0,003 | High_in_IC75 |
| GO_VESICLE_TARGETING | 64 | 2,461 | 0,003 | High_in_IC75 |
| GO_REGULATION_OF_ORGANELLE_ASSEMBLY | 126 | 2,46 | 0,003 | High_in_IC75 |
| GO_POSITIVE_REGULATION_OF_CELL_ACTIVATION | 194 | 2,458 | 0,003 | High_in_IC75 |
| GO_REGULATION_OF_LEUKOCYTE_MIGRATION | 98 | 2,453 | 0,003 | High_in_IC75 |
| GO_ACTIVATION_OF_PROTEIN_KINASE_ACTIVITY | 218 | 2,452 | 0,003 | High_in_IC75 |
| GO_POSITIVE_REGULATION_OF_APOPTOTIC_SIGNALING_PATHWAY | 151 | 2,452 | 0,003 | High_in_IC75 |
| GO_NEGATIVE_REGULATION_OF_TRANSPORT | 315 | 2,452 | 0,003 | High_in_IC75 |
| GO_RAS_PROTEIN_SIGNAL_TRANSDUCTION | 125 | 2,448 | 0,003 | High_in_IC75 |
| GO_ENDOTHELIUM_DEVELOPMENT | 71 | 2,446 | 0,003 | High_in_IC75 |
| GO_NEGATIVE_REGULATION_OF_SMALL_GTPASE_MEDIATED_SIGNAL_TRANSDUCTION | 33 | 2,446 | 0,003 | High_in_IC75 |
| GO_REGULATION_OF_PLASMA_MEMBRANE_ORGANIZATION | 63 | 2,444 | 0,003 | High_in_IC75 |
| GO_POSITIVE_REGULATION_OF_CYTOKINE_PRODUCTION | 264 | 2,441 | 0,003 | High_in_IC75 |
| GO_LEUKOCYTE_DIFFERENTIATION | 210 | 2,433 | 0,003 | High_in_IC75 |
| GO_REGULATION_OF_LEUKOCYTE_DEGRANULATION | 34 | 2,433 | 0,003 | High_in_IC75 |
| GO_POSITIVE_REGULATION_OF_CHEMOTAXIS | 77 | 2,433 | 0,003 | High_in_IC75 |
| GO_ADHERENS_JUNCTION_ORGANIZATION | 56 | 2,432 | 0,003 | High_in_IC75 |
| GO_REGULATION_OF_EPIDERMAL_GROWTH_FACTOR_ACTIVATED_RECEPTOR_ACTIVITY | 19 | 2,43 | 0,003 | High_in_IC75 |
| GO_POSITIVE_REGULATION_OF_VASCULATURE_DEVELOPMENT | 97 | 2,425 | 0,003 | High_in_IC75 |
| GO_ENDOCYTOSIS | 351 | 2,425 | 0,003 | High_in_IC75 |
| GO_PLACENTA_DEVELOPMENT | 117 | 2,424 | 0,003 | High_in_IC75 |
| GO_TUBE_MORPHOGENESIS | 243 | 2,423 | 0,003 | High_in_IC75 |
| GO_REGULATION_OF_LEUKOCYTE_MEDIATED_IMMUNITY | 103 | 2,422 | 0,003 | High_in_IC75 |
| GO_RESPONSE_TO_STARVATION | 121 | 2,421 | 0,003 | High_in_IC75 |
| GO_REGULATION_OF_ADAPTIVE_IMMUNE_RESPONSE | 77 | 2,415 | 0,003 | High_in_IC75 |
| GO_POSITIVE_REGULATION_OF_LEUKOCYTE_PROLIFERATION | 84 | 2,414 | 0,004 | High_in_IC75 |
| GO_MYELOID_CELL_DIFFERENTIATION | 148 | 2,413 | 0,003 | High_in_IC75 |
| GO_POSITIVE_REGULATION_OF_INTRINSIC_APOPTOTIC_SIGNALING_PATHWAY | 47 | 2,413 | 0,004 | High_in_IC75 |
| GO_CYTOKINE_MEDIATED_SIGNALING_PATHWAY | 316 | 2,412 | 0,003 | High_in_IC75 |
| GO_POSITIVE_REGULATION_OF_AUTOPHAGY | 66 | 2,409 | 0,004 | High_in_IC75 |
| GO_NEGATIVE_REGULATION_OF_HEMATOPOIETIC_PROGENITOR_CELL_DIFFERENTIATION | 18 | 2,401 | 0,004 | High_in_IC75 |
| GO_POSITIVE_REGULATION_OF_FAT_CELL_DIFFERENTIATION | 39 | 2,386 | 0,004 | High_in_IC75 |
| GO_RESPONSE_TO_FLUID_SHEAR_STRESS | 28 | 2,382 | 0,004 | High_in_IC75 |
| GO_RHYTHMIC_PROCESS | 221 | 2,38 | 0,004 | High_in_IC75 |
| GO_LEUKOCYTE_CELL_CELL_ADHESION | 181 | 2,38 | 0,004 | High_in_IC75 |
| GO_POSITIVE_REGULATION_OF_PEPTIDYL_TYROSINE_PHOSPHORYLATION | 106 | 2,377 | 0,004 | High_in_IC75 |
| GO_CELLULAR_RESPONSE_TO_BIOTIC_STIMULUS | 120 | 2,376 | 0,004 | High_in_IC75 |
| GO_REGULATION_OF_ANTIGEN_RECEPTOR_MEDIATED_SIGNALING_PATHWAY | 30 | 2,375 | 0,004 | High_in_IC75 |
| GO_REGULATION_OF_CELL_MORPHOGENESIS_INVOLVED_IN_DIFFERENTIATION | 251 | 2,366 | 0,004 | High_in_IC75 |
| GO_REGULATION_OF_T_CELL_RECEPTOR_SIGNALING_PATHWAY | 20 | 2,365 | 0,004 | High_in_IC75 |
| GO_CELLULAR_DEFENSE_RESPONSE | 35 | 2,365 | 0,004 | High_in_IC75 |
| GO_REGULATION_OF_VESICLE_MEDIATED_TRANSPORT | 344 | 2,362 | 0,005 | High_in_IC75 |
| GO_LOCALIZATION_WITHIN_MEMBRANE | 97 | 2,361 | 0,005 | High_in_IC75 |
| GO_REGULATION_OF_ENDOCRINE_PROCESS | 32 | 2,36 | 0,005 | High_in_IC75 |
| GO_REGULATION_OF_PROTEIN_TYROSINE_KINASE_ACTIVITY | 50 | 2,358 | 0,005 | High_in_IC75 |
| GO_CELLULAR_TRANSITION_METAL_ION_HOMEOSTASIS | 57 | 2,356 | 0,005 | High_in_IC75 |
| GO_NEGATIVE_REGULATION_OF_PROTEIN_KINASE_B_SIGNALING | 29 | 2,355 | 0,005 | High_in_IC75 |
| GO_NEGATIVE_REGULATION_OF_GROWTH | 177 | 2,347 | 0,005 | High_in_IC75 |
| GO_CELLULAR_RESPONSE_TO_LIPID | 338 | 2,342 | 0,005 | High_in_IC75 |
| GO_CELLULAR_RESPONSE_TO_INTERFERON_GAMMA | 87 | 2,34 | 0,005 | High_in_IC75 |
| GO_NEGATIVE_REGULATION_OF_IMMUNE_RESPONSE | 80 | 2,335 | 0,005 | High_in_IC75 |
| GO_REGULATION_OF_MAST_CELL_ACTIVATION | 29 | 2,332 | 0,005 | High_in_IC75 |
| GO_VISUAL_BEHAVIOR | 33 | 2,33 | 0,005 | High_in_IC75 |
| GO_MYELOID_LEUKOCYTE_MEDIATED_IMMUNITY | 30 | 2,321 | 0,006 | High_in_IC75 |
| GO_CELLULAR_RESPONSE_TO_NITROGEN_COMPOUND | 375 | 2,317 | 0,006 | High_in_IC75 |
| GO_CELLULAR_RESPONSE_TO_OXYGEN_LEVELS | 125 | 2,313 | 0,006 | High_in_IC75 |
| GO_RESPONSE_TO_INTERFERON_GAMMA | 101 | 2,313 | 0,006 | High_in_IC75 |
| GO_POSITIVE_REGULATION_OF_TRANSMEMBRANE_RECEPTOR_PROTEIN_SERINE_THREONINE_KINASE_SIGNALING_PATHWAY | 69 | 2,305 | 0,006 | High_in_IC75 |
| GO_CIRCULATORY_SYSTEM_PROCESS | 217 | 2,297 | 0,007 | High_in_IC75 |
| GO_EPIDERMIS_DEVELOPMENT | 180 | 2,297 | 0,007 | High_in_IC75 |
| GO_KERATINIZATION | 27 | 2,292 | 0,007 | High_in_IC75 |
| GO_DIVALENT_INORGANIC_CATION_HOMEOSTASIS | 217 | 2,29 | 0,007 | High_in_IC75 |
| GO_REGULATION_OF_ERBB_SIGNALING_PATHWAY | 73 | 2,29 | 0,007 | High_in_IC75 |
| GO_CELLULAR_RESPONSE_TO_OXIDATIVE_STRESS | 161 | 2,284 | 0,007 | High_in_IC75 |
| GO_REGULATION_OF_CELLULAR_RESPONSE_TO_TRANSFORMING_GROWTH_FACTOR_BETA_STIMULUS | 79 | 2,283 | 0,007 | High_in_IC75 |
| GO_HEART_DEVELOPMENT | 324 | 2,279 | 0,007 | High_in_IC75 |
| GO_REGULATION_OF_BLOOD_CIRCULATION | 176 | 2,274 | 0,007 | High_in_IC75 |
| GO_EPIDERMAL_CELL_DIFFERENTIATION | 97 | 2,272 | 0,007 | High_in_IC75 |
| GO_REGULATION_OF_NEURON_DEATH | 196 | 2,268 | 0,008 | High_in_IC75 |
| GO_NEGATIVE_REGULATION_OF_HEMOPOIESIS | 96 | 2,266 | 0,008 | High_in_IC75 |
| GO_NEGATIVE_REGULATION_OF_T_CELL_PROLIFERATION | 39 | 2,264 | 0,008 | High_in_IC75 |
| GO_AUTOPHAGY | 326 | 2,263 | 0,008 | High_in_IC75 |
| GO_MORPHOGENESIS_OF_A_BRANCHING_STRUCTURE | 125 | 2,263 | 0,008 | High_in_IC75 |
| GO_LIPOPOLYSACCHARIDE_MEDIATED_SIGNALING_PATHWAY | 22 | 2,262 | 0,008 | High_in_IC75 |
| GO_RESPONSE_TO_INSULIN | 165 | 2,262 | 0,008 | High_in_IC75 |
| GO_SIGNAL_TRANSDUCTION_BY_PROTEIN_PHOSPHORYLATION | 316 | 2,261 | 0,008 | High_in_IC75 |
| GO_I_KAPPAB_KINASE_NF_KAPPAB_SIGNALING | 55 | 2,257 | 0,008 | High_in_IC75 |
| GO_RESPONSE_TO_HYDROGEN_PEROXIDE | 98 | 2,249 | 0,008 | High_in_IC75 |
| GO_POSITIVE_REGULATION_OF_IMMUNE_EFFECTOR_PROCESS | 105 | 2,246 | 0,008 | High_in_IC75 |
| GO_ENDOSOME_ORGANIZATION | 57 | 2,246 | 0,008 | High_in_IC75 |
| GO_REGULATION_OF_TRANSMEMBRANE_RECEPTOR_PROTEIN_SERINE_THREONINE_KINASE_SIGNALING_PATHWAY | 145 | 2,245 | 0,008 | High_in_IC75 |
| GO_POSITIVE_REGULATION_OF_ESTABLISHMENT_OF_PROTEIN_LOCALIZATION | 391 | 2,245 | 0,008 | High_in_IC75 |
| GO_ESTABLISHMENT_OF_ENDOTHELIAL_BARRIER | 29 | 2,245 | 0,008 | High_in_IC75 |
| GO_REGULATION_OF_MAST_CELL_ACTIVATION_INVOLVED_IN_IMMUNE_RESPONSE | 25 | 2,244 | 0,008 | High_in_IC75 |
| GO_REGULATION_OF_NEURON_PROJECTION_DEVELOPMENT | 290 | 2,242 | 0,008 | High_in_IC75 |
| GO_REGULATION_OF_CELLULAR_RESPONSE_TO_INSULIN_STIMULUS | 47 | 2,239 | 0,009 | High_in_IC75 |
| GO_CELLULAR_RESPONSE_TO_HYDROGEN_PEROXIDE | 56 | 2,237 | 0,009 | High_in_IC75 |
| GO_REGULATION_OF_HAIR_CYCLE | 15 | 2,235 | 0,009 | High_in_IC75 |
| GO_CELLULAR_RESPONSE_TO_HORMONE_STIMULUS | 414 | 2,235 | 0,009 | High_in_IC75 |
| GO_VESICLE_COATING | 62 | 2,234 | 0,009 | High_in_IC75 |
| GO_VACUOLE_ORGANIZATION | 146 | 2,233 | 0,009 | High_in_IC75 |
| GO_NEGATIVE_REGULATION_OF_SECRETION | 130 | 2,231 | 0,009 | High_in_IC75 |
| GO_NEGATIVE_REGULATION_OF_MAPK_CASCADE | 120 | 2,228 | 0,009 | High_in_IC75 |
| GO_REGULATION_OF_NEURON_APOPTOTIC_PROCESS | 149 | 2,227 | 0,009 | High_in_IC75 |
| GO_NEGATIVE_REGULATION_OF_ERBB_SIGNALING_PATHWAY | 42 | 2,227 | 0,009 | High_in_IC75 |
| GO_ACTIN_FILAMENT_BASED_MOVEMENT | 53 | 2,226 | 0,009 | High_in_IC75 |
| GO_ORGANELLE_LOCALIZATION | 342 | 2,225 | 0,009 | High_in_IC75 |
| GO_NEGATIVE_REGULATION_OF_CELLULAR_RESPONSE_TO_INSULIN_STIMULUS | 27 | 2,225 | 0,009 | High_in_IC75 |
| GO_REGULATION_OF_CYTOSOLIC_CALCIUM_ION_CONCENTRATION | 117 | 2,22 | 0,009 | High_in_IC75 |
| GO_REGULATION_OF_EPITHELIAL_CELL_MIGRATION | 127 | 2,217 | 0,009 | High_in_IC75 |
| GO_REGULATION_OF_MYELOID_CELL_DIFFERENTIATION | 138 | 2,21 | 0,01 | High_in_IC75 |
| GO_INNATE_IMMUNE_RESPONSE | 389 | 2,21 | 0,01 | High_in_IC75 |
| GO_LEUKOCYTE_DEGRANULATION | 24 | 2,203 | 0,01 | High_in_IC75 |
| GO_POSITIVE_REGULATION_OF_EPITHELIAL_CELL_MIGRATION | 80 | 2,201 | 0,01 | High_in_IC75 |
| GO_ESTABLISHMENT_OR_MAINTENANCE_OF_CELL_POLARITY | 114 | 2,201 | 0,01 | High_in_IC75 |
| GO_REGULATION_OF_INTRINSIC_APOPTOTIC_SIGNALING_PATHWAY | 121 | 2,198 | 0,01 | High_in_IC75 |
| GO_CELLULAR_HOMEOSTASIS | 459 | 2,196 | 0,01 | High_in_IC75 |
| GO_RESPONSE_TO_EXTRACELLULAR_STIMULUS | 319 | 2,193 | 0,01 | High_in_IC75 |
| GO_PLASMA_MEMBRANE_ORGANIZATION | 164 | 2,193 | 0,01 | High_in_IC75 |
| GO_POSITIVE_REGULATION_OF_LEUKOCYTE_MIGRATION | 71 | 2,191 | 0,01 | High_in_IC75 |
| GO_CELLULAR_RESPONSE_TO_FLUID_SHEAR_STRESS | 18 | 2,189 | 0,01 | High_in_IC75 |
| GO_REGULATION_OF_ENDOPLASMIC_RETICULUM_STRESS_INDUCED_INTRINSIC_APOPTOTIC_SIGNALING_PATHWAY | 26 | 2,189 | 0,01 | High_in_IC75 |
| GO_INTERFERON_GAMMA_MEDIATED_SIGNALING_PATHWAY | 57 | 2,175 | 0,01 | High_in_IC75 |
| GO_CELL_CYCLE_ARREST | 130 | 2,171 | 0,01 | High_in_IC75 |
| GO_ER_ASSOCIATED_UBIQUITIN_DEPENDENT_PROTEIN_CATABOLIC_PROCESS | 56 | 2,17 | 0,01 | High_in_IC75 |
| GO_REGULATION_OF_HEMATOPOIETIC_PROGENITOR_CELL_DIFFERENTIATION | 27 | 2,169 | 0,01 | High_in_IC75 |
| GO_REGULATION_OF_SYSTEM_PROCESS | 310 | 2,167 | 0,01 | High_in_IC75 |
| GO_POSITIVE_REGULATION_OF_RESPONSE_TO_EXTRACELLULAR_STIMULUS | 42 | 2,163 | 0,01 | High_in_IC75 |
| GO_CELLULAR_RESPONSE_TO_AMINO_ACID_STARVATION | 22 | 2,155 | 0,01 | High_in_IC75 |
| GO_ENDOTHELIAL_CELL_DIFFERENTIATION | 56 | 2,151 | 0,01 | High_in_IC75 |
| GO_REGULATION_OF_CELLULAR_RESPONSE_TO_GROWTH_FACTOR_STIMULUS | 164 | 2,151 | 0,01 | High_in_IC75 |
| GO_ER_NUCLEUS_SIGNALING_PATHWAY | 33 | 2,151 | 0,01 | High_in_IC75 |
| GO_REGULATION_OF_PHOSPHOLIPASE_ACTIVITY | 44 | 2,15 | 0,01 | High_in_IC75 |
| GO_EMBRYONIC_MORPHOGENESIS | 391 | 2,148 | 0,01 | High_in_IC75 |
| GO_POSITIVE_REGULATION_OF_AXON_EXTENSION | 29 | 2,147 | 0,01 | High_in_IC75 |
| GO_REGULATION_OF_LEUKOCYTE_DIFFERENTIATION | 163 | 2,146 | 0,01 | High_in_IC75 |
| GO_CIRCADIAN_REGULATION_OF_GENE_EXPRESSION | 47 | 2,144 | 0,01 | High_in_IC75 |
| GO_REGULATION_OF_DEVELOPMENTAL_GROWTH | 195 | 2,142 | 0,01 | High_in_IC75 |
| GO_POSITIVE_REGULATION_OF_SEQUENCE_SPECIFIC_DNA_BINDING_TRANSCRIPTION_FACTOR_ACTIVITY | 179 | 2,14 | 0,01 | High_in_IC75 |
| GO_ARTERY_MORPHOGENESIS | 30 | 2,139 | 0,01 | High_in_IC75 |
| GO_REGULATION_OF_INTERLEUKIN_6_PRODUCTION | 65 | 2,138 | 0,01 | High_in_IC75 |
| GO_POSITIVE_REGULATION_OF_LIPID_CATABOLIC_PROCESS | 19 | 2,138 | 0,01 | High_in_IC75 |
| GO_VESICLE_MEDIATED_TRANSPORT_BETWEEN_ENDOSOMAL_COMPARTMENTS | 19 | 2,138 | 0,01 | High_in_IC75 |
| GO_TOLL_LIKE_RECEPTOR_SIGNALING_PATHWAY | 64 | 2,136 | 0,01 | High_in_IC75 |
| GO_PROTEIN_AUTOPHOSPHORYLATION | 150 | 2,133 | 0,01 | High_in_IC75 |
| GO_REGULATION_OF_SEQUENCE_SPECIFIC_DNA_BINDING_TRANSCRIPTION_FACTOR_ACTIVITY | 285 | 2,129 | 0,01 | High_in_IC75 |
| GO_PROTEIN_LOCALIZATION_TO_GOLGI_APPARATUS | 27 | 2,126 | 0,02 | High_in_IC75 |
| GO_MICROVILLUS_ORGANIZATION | 19 | 2,124 | 0,02 | High_in_IC75 |
| GO_REGULATION_OF_EXTRINSIC_APOPTOTIC_SIGNALING_PATHWAY_VIA_DEATH_DOMAIN_RECEPTORS | 45 | 2,124 | 0,02 | High_in_IC75 |
| GO_NEGATIVE_REGULATION_OF_HYDROLASE_ACTIVITY | 271 | 2,119 | 0,02 | High_in_IC75 |
| GO_POSITIVE_REGULATION_OF_CELL_PROJECTION_ORGANIZATION | 224 | 2,119 | 0,02 | High_in_IC75 |
| GO_APOPTOTIC_SIGNALING_PATHWAY | 238 | 2,118 | 0,02 | High_in_IC75 |
| GO_NEGATIVE_REGULATION_OF_APOPTOTIC_SIGNALING_PATHWAY | 161 | 2,118 | 0,02 | High_in_IC75 |
| GO_RESPONSE_TO_OXYGEN_LEVELS | 255 | 2,117 | 0,02 | High_in_IC75 |
| GO_RESPONSE_TO_INTERLEUKIN_1 | 84 | 2,116 | 0,02 | High_in_IC75 |
| GO_PROTEIN_EXIT_FROM_ENDOPLASMIC_RETICULUM | 18 | 2,114 | 0,02 | High_in_IC75 |
| GO_LYMPHOCYTE_ACTIVATION | 241 | 2,112 | 0,02 | High_in_IC75 |
| GO_EPITHELIAL_CELL_PROLIFERATION | 67 | 2,111 | 0,02 | High_in_IC75 |
| GO_NEGATIVE_REGULATION_OF_INTERFERON_GAMMA_PRODUCTION | 21 | 2,111 | 0,02 | High_in_IC75 |
| GO_REGULATION_OF_AUTOPHAGOSOME_ASSEMBLY | 31 | 2,111 | 0,02 | High_in_IC75 |
| GO_POSITIVE_REGULATION_OF_CELL_MORPHOGENESIS_INVOLVED_IN_DIFFERENTIATION | 129 | 2,11 | 0,02 | High_in_IC75 |
| GO_REGULATION_OF_LYMPHOCYTE_MIGRATION | 26 | 2,109 | 0,02 | High_in_IC75 |
| GO_NEGATIVE_REGULATION_OF_LEUKOCYTE_PROLIFERATION | 48 | 2,109 | 0,02 | High_in_IC75 |
| GO_REGULATION_OF_RECEPTOR_ACTIVITY | 85 | 2,106 | 0,02 | High_in_IC75 |
| GO_CELL_CHEMOTAXIS | 101 | 2,106 | 0,02 | High_in_IC75 |
| GO_ERAD_PATHWAY | 65 | 2,103 | 0,02 | High_in_IC75 |
| GO_AUTOPHAGOSOME_ORGANIZATION | 36 | 2,102 | 0,02 | High_in_IC75 |
| GO_INTRINSIC_APOPTOTIC_SIGNALING_PATHWAY_IN_RESPONSE_TO_ENDOPLASMIC_RETICULUM_STRESS | 29 | 2,102 | 0,02 | High_in_IC75 |
| GO_HEMOSTASIS | 222 | 2,097 | 0,02 | High_in_IC75 |
| GO_REGULATION_OF_EXTENT_OF_CELL_GROWTH | 76 | 2,093 | 0,02 | High_in_IC75 |
| GO_NEURON_PROJECTION_DEVELOPMENT | 384 | 2,092 | 0,02 | High_in_IC75 |
| GO_REGULATION_OF_PHOSPHOLIPASE_C_ACTIVITY | 24 | 2,092 | 0,02 | High_in_IC75 |
| GO_REGULATION_OF_CELL_SIZE | 127 | 2,087 | 0,02 | High_in_IC75 |
| GO_REGULATION_OF_AXONOGENESIS | 127 | 2,087 | 0,02 | High_in_IC75 |
| GO_POSITIVE_REGULATION_OF_CELL_DEVELOPMENT | 336 | 2,086 | 0,02 | High_in_IC75 |
| GO_NEGATIVE_REGULATION_OF_RESPONSE_TO_ENDOPLASMIC_RETICULUM_STRESS | 31 | 2,085 | 0,02 | High_in_IC75 |
| GO_NATURAL_KILLER_CELL_ACTIVATION | 33 | 2,085 | 0,02 | High_in_IC75 |
| GO_NEGATIVE_REGULATION_OF_EXTRINSIC_APOPTOTIC_SIGNALING_PATHWAY | 77 | 2,082 | 0,02 | High_in_IC75 |
| GO_ANTIMICROBIAL_HUMORAL_RESPONSE | 25 | 2,081 | 0,02 | High_in_IC75 |
| GO_REGULATION_OF_COAGULATION | 59 | 2,078 | 0,02 | High_in_IC75 |
| GO_HOMOTYPIC_CELL_CELL_ADHESION | 38 | 2,078 | 0,02 | High_in_IC75 |
| GO_REGULATION_OF_INTRACELLULAR_TRANSPORT | 494 | 2,078 | 0,02 | High_in_IC75 |
| GO_RETROGRADE_TRANSPORT_VESICLE_RECYCLING_WITHIN_GOLGI | 21 | 2,077 | 0,02 | High_in_IC75 |
| GO_NEGATIVE_REGULATION_OF_LEUKOCYTE_MEDIATED_IMMUNITY | 32 | 2,077 | 0,02 | High_in_IC75 |
| GO_RESPONSE_TO_NERVE_GROWTH_FACTOR | 30 | 2,073 | 0,02 | High_in_IC75 |
| GO_REGULATION_OF_PROTEIN_SECRETION | 266 | 2,073 | 0,02 | High_in_IC75 |
| GO_IMMUNE_SYSTEM_DEVELOPMENT | 436 | 2,072 | 0,02 | High_in_IC75 |
| GO_REGULATION_OF_DNA_TEMPLATED_TRANSCRIPTION_IN_RESPONSE_TO_STRESS | 61 | 2,069 | 0,02 | High_in_IC75 |
| GO_POSITIVE_REGULATION_OF_REACTIVE_OXYGEN_SPECIES_METABOLIC_PROCESS | 62 | 2,067 | 0,02 | High_in_IC75 |
| GO_REGULATION_OF_CELL_SUBSTRATE_ADHESION | 132 | 2,066 | 0,02 | High_in_IC75 |
| GO_CELL_ACTIVATION_INVOLVED_IN_IMMUNE_RESPONSE | 93 | 2,066 | 0,02 | High_in_IC75 |
| GO_PEPTIDYL_SERINE_MODIFICATION | 123 | 2,065 | 0,02 | High_in_IC75 |
| GO_CYTOKINE_PRODUCTION | 73 | 2,064 | 0,02 | High_in_IC75 |
| GO_NEGATIVE_REGULATION_OF_ANOIKIS | 15 | 2,063 | 0,02 | High_in_IC75 |
| GO_POSITIVE_REGULATION_OF_PHOSPHOLIPASE_ACTIVITY | 34 | 2,063 | 0,02 | High_in_IC75 |
| GO_RESPONSE_TO_ALCOHOL | 259 | 2,059 | 0,02 | High_in_IC75 |
| GO_CELL_CELL_SIGNALING | 442 | 2,058 | 0,02 | High_in_IC75 |
| GO_POSITIVE_REGULATION_OF_TYROSINE_PHOSPHORYLATION_OF_STAT3_PROTEIN | 27 | 2,054 | 0,02 | High_in_IC75 |
| GO_REGULATION_OF_REACTIVE_OXYGEN_SPECIES_METABOLIC_PROCESS | 115 | 2,051 | 0,02 | High_in_IC75 |
| GO_POSITIVE_REGULATION_OF_IMMUNE_RESPONSE | 383 | 2,051 | 0,02 | High_in_IC75 |
| GO_MAINTENANCE_OF_PROTEIN_LOCALIZATION_IN_ORGANELLE | 22 | 2,048 | 0,02 | High_in_IC75 |
| GO_REGULATION_OF_ALPHA_BETA_T_CELL_PROLIFERATION | 15 | 2,044 | 0,02 | High_in_IC75 |
| GO_NEGATIVE_REGULATION_OF_MACROAUTOPHAGY | 19 | 2,042 | 0,02 | High_in_IC75 |
| GO_ENDOTHELIAL_CELL_DEVELOPMENT | 40 | 2,039 | 0,02 | High_in_IC75 |
| GO_POSITIVE_REGULATION_OF_RESPONSE_TO_WOUNDING | 98 | 2,037 | 0,02 | High_in_IC75 |
| GO_ARTERY_DEVELOPMENT | 47 | 2,035 | 0,02 | High_in_IC75 |
| GO_REGULATION_OF_ESTABLISHMENT_OF_PROTEIN_LOCALIZATION_TO_PLASMA_MEMBRANE | 41 | 2,034 | 0,02 | High_in_IC75 |
| GO_REGULATION_OF_TUMOR_NECROSIS_FACTOR_SUPERFAMILY_CYTOKINE_PRODUCTION | 71 | 2,029 | 0,02 | High_in_IC75 |
| GO_NEGATIVE_REGULATION_OF_RESPONSE_TO_EXTERNAL_STIMULUS | 198 | 2,026 | 0,02 | High_in_IC75 |
| GO_POSITIVE_REGULATION_OF_DEFENSE_RESPONSE | 263 | 2,026 | 0,02 | High_in_IC75 |
| GO_RESPONSE_TO_STEROID_HORMONE | 348 | 2,026 | 0,02 | High_in_IC75 |
| GO_POSITIVE_REGULATION_OF_LEUKOCYTE_CHEMOTAXIS | 53 | 2,023 | 0,02 | High_in_IC75 |
| GO_REGULATION_OF_TRANSFORMING_GROWTH_FACTOR_BETA_PRODUCTION | 21 | 2,023 | 0,02 | High_in_IC75 |
| GO_MULTIVESICULAR_BODY_ORGANIZATION | 29 | 2,021 | 0,02 | High_in_IC75 |
| GO_NEGATIVE_REGULATION_OF_EXOCYTOSIS | 22 | 2,02 | 0,02 | High_in_IC75 |
| GO_OVULATION_CYCLE | 82 | 2,018 | 0,02 | High_in_IC75 |
| GO_REGULATION_OF_HEART_CONTRACTION | 129 | 2,017 | 0,02 | High_in_IC75 |
| GO_POSITIVE_REGULATION_OF_TRANSCRIPTION_FROM_RNA_POLYMERASE_II_PROMOTER_IN_RESPONSE_TO_STRESS | 20 | 2,015 | 0,02 | High_in_IC75 |
| GO_DEVELOPMENTAL_MATURATION | 138 | 2,015 | 0,02 | High_in_IC75 |
| GO_OVARIAN_FOLLICLE_DEVELOPMENT | 48 | 2,012 | 0,02 | High_in_IC75 |
| GO_PEPTIDYL_TYROSINE_MODIFICATION | 133 | 2,004 | 0,03 | High_in_IC75 |
| GO_REGULATION_OF_INTERLEUKIN_2_PRODUCTION | 36 | 2,001 | 0,03 | High_in_IC75 |
| GO_REGULATION_OF_CHEMOTAXIS | 120 | 2,001 | 0,03 | High_in_IC75 |
| GO_ENDOSOME_TO_LYSOSOME_TRANSPORT | 38 | 1,997 | 0,03 | High_in_IC75 |
| GO_CELLULAR_CHEMICAL_HOMEOSTASIS | 372 | 1,997 | 0,03 | High_in_IC75 |
| GO_REGULATION_OF_ACTIN_FILAMENT_LENGTH | 125 | 1,997 | 0,03 | High_in_IC75 |
| GO_REGULATION_OF_CELL_MIGRATION_INVOLVED_IN_SPROUTING_ANGIOGENESIS | 17 | 1,997 | 0,03 | High_in_IC75 |
| GO_REGULATION_OF_MYELOID_LEUKOCYTE_DIFFERENTIATION | 78 | 1,986 | 0,03 | High_in_IC75 |
| GO_ZINC_ION_HOMEOSTASIS | 16 | 1,983 | 0,03 | High_in_IC75 |
| GO_REGULATION_OF_ENDOTHELIAL_CELL_MIGRATION | 81 | 1,983 | 0,03 | High_in_IC75 |
| GO_RESPONSE_TO_INORGANIC_SUBSTANCE | 365 | 1,983 | 0,03 | High_in_IC75 |
| GO_REGULATION_OF_NATURAL_KILLER_CELL_ACTIVATION | 19 | 1,982 | 0,03 | High_in_IC75 |
| GO_RESPONSE_TO_ARSENIC_CONTAINING_SUBSTANCE | 26 | 1,978 | 0,03 | High_in_IC75 |
| GO_REGULATION_OF_BINDING | 237 | 1,977 | 0,03 | High_in_IC75 |
| GO_POSITIVE_REGULATION_OF_NEURON_PROJECTION_DEVELOPMENT | 167 | 1,976 | 0,03 | High_in_IC75 |
| GO_CELL_MORPHOGENESIS_INVOLVED_IN_NEURON_DIFFERENTIATION | 254 | 1,976 | 0,03 | High_in_IC75 |
| GO_REGULATION_OF_ENDOCYTOSIS | 147 | 1,975 | 0,03 | High_in_IC75 |
| GO_VASCULOGENESIS | 39 | 1,974 | 0,03 | High_in_IC75 |
| GO_EMBRYONIC_ORGAN_DEVELOPMENT | 287 | 1,973 | 0,03 | High_in_IC75 |
| GO_REGULATION_OF_ADHERENS_JUNCTION_ORGANIZATION | 41 | 1,973 | 0,03 | High_in_IC75 |
| GO_NEGATIVE_REGULATION_OF_RESPONSE_TO_WOUNDING | 105 | 1,972 | 0,03 | High_in_IC75 |
| GO_REGULATION_OF_ACTIN_FILAMENT_DEPOLYMERIZATION | 39 | 1,969 | 0,03 | High_in_IC75 |
| GO_NEGATIVE_REGULATION_OF_BINDING | 106 | 1,965 | 0,03 | High_in_IC75 |
| GO_REGULATION_OF_BLOOD_PRESSURE | 102 | 1,965 | 0,03 | High_in_IC75 |
| GO_CELLULAR_RESPONSE_TO_REACTIVE_OXYGEN_SPECIES | 89 | 1,964 | 0,03 | High_in_IC75 |
| GO_NEGATIVE_REGULATION_OF_CELL_GROWTH | 132 | 1,961 | 0,03 | High_in_IC75 |
| GO_NEURON_PROJECTION_MORPHOGENESIS | 281 | 1,959 | 0,03 | High_in_IC75 |
| GO_SIGNAL_RELEASE | 116 | 1,957 | 0,03 | High_in_IC75 |
| GO_NEGATIVE_REGULATION_OF_IMMUNE_EFFECTOR_PROCESS | 71 | 1,955 | 0,03 | High_in_IC75 |
| GO_REGULATION_OF_STAT_CASCADE | 89 | 1,954 | 0,03 | High_in_IC75 |
| GO_NEGATIVE_REGULATION_OF_NEURAL_PRECURSOR_CELL_PROLIFERATION | 15 | 1,953 | 0,03 | High_in_IC75 |
| GO_CHROMATIN_SILENCING_AT_RDNA | 33 | 1,952 | 0,03 | High_in_IC75 |
| GO_REGULATION_OF_ENDOTHELIAL_CELL_APOPTOTIC_PROCESS | 31 | 1,952 | 0,03 | High_in_IC75 |
| GO_REGULATION_OF_INNATE_IMMUNE_RESPONSE | 275 | 1,952 | 0,03 | High_in_IC75 |
| GO_BEHAVIOR | 311 | 1,95 | 0,03 | High_in_IC75 |
| GO_CHEMOKINE_MEDIATED_SIGNALING_PATHWAY | 33 | 1,949 | 0,03 | High_in_IC75 |
| GO_OVULATION_CYCLE_PROCESS | 64 | 1,949 | 0,03 | High_in_IC75 |
| GO_CELLULAR_RESPONSE_TO_TOXIC_SUBSTANCE | 18 | 1,949 | 0,03 | High_in_IC75 |
| GO_PROTEIN_HETEROOLIGOMERIZATION | 90 | 1,949 | 0,03 | High_in_IC75 |
| GO_CYCLIC_NUCLEOTIDE_METABOLIC_PROCESS | 30 | 1,946 | 0,03 | High_in_IC75 |
| GO_MAINTENANCE_OF_LOCATION_IN_CELL | 71 | 1,946 | 0,03 | High_in_IC75 |
| GO_TRANSITION_METAL_ION_HOMEOSTASIS | 81 | 1,945 | 0,03 | High_in_IC75 |
| GO_METAL_ION_TRANSPORT | 348 | 1,943 | 0,03 | High_in_IC75 |
| GO_REGULATION_OF_HORMONE_SECRETION | 167 | 1,943 | 0,03 | High_in_IC75 |
| GO_BRANCHING_MORPHOGENESIS_OF_AN_EPITHELIAL_TUBE | 98 | 1,942 | 0,03 | High_in_IC75 |
| GO_REGULATION_OF_CARBOHYDRATE_METABOLIC_PROCESS | 122 | 1,942 | 0,03 | High_in_IC75 |
| GO_COGNITION | 161 | 1,94 | 0,03 | High_in_IC75 |
| GO_HEART_MORPHOGENESIS | 139 | 1,936 | 0,03 | High_in_IC75 |
| GO_ORGAN_OR_TISSUE_SPECIFIC_IMMUNE_RESPONSE | 16 | 1,934 | 0,03 | High_in_IC75 |
| GO_CARDIAC_EPITHELIAL_TO_MESENCHYMAL_TRANSITION | 18 | 1,933 | 0,03 | High_in_IC75 |
| GO_RESPONSE_TO_CAMP | 70 | 1,933 | 0,03 | High_in_IC75 |
| GO_POSITIVE_REGULATION_OF_SMOOTH_MUSCLE_CELL_PROLIFERATION | 45 | 1,932 | 0,03 | High_in_IC75 |
| GO_POSITIVE_REGULATION_OF_ENDOTHELIAL_CELL_MIGRATION | 49 | 1,932 | 0,03 | High_in_IC75 |
| GO_REGULATION_OF_RESPONSE_TO_CYTOKINE_STIMULUS | 113 | 1,93 | 0,03 | High_in_IC75 |
| GO_PROTEIN_KINASE_B_SIGNALING | 28 | 1,927 | 0,03 | High_in_IC75 |
| GO_MAINTENANCE_OF_LOCATION | 105 | 1,927 | 0,03 | High_in_IC75 |
| GO_ESTABLISHMENT_OF_PROTEIN_LOCALIZATION_TO_GOLGI | 17 | 1,924 | 0,04 | High_in_IC75 |
| GO_CELLULAR_RESPONSE_TO_ORGANIC_CYCLIC_COMPOUND | 346 | 1,923 | 0,04 | High_in_IC75 |
| GO_POSITIVE_REGULATION_OF_MAP_KINASE_ACTIVITY | 153 | 1,922 | 0,04 | High_in_IC75 |
| GO_MYELOID_CELL_ACTIVATION_INVOLVED_IN_IMMUNE_RESPONSE | 31 | 1,919 | 0,04 | High_in_IC75 |
| GO_MITOCHONDRION_LOCALIZATION | 30 | 1,917 | 0,04 | High_in_IC75 |
| GO_RETROGRADE_TRANSPORT_ENDOSOME_TO_GOLGI | 66 | 1,917 | 0,04 | High_in_IC75 |
| GO_SYNCYTIUM_FORMATION | 15 | 1,917 | 0,04 | High_in_IC75 |
| GO_ENDODERM_DEVELOPMENT | 55 | 1,914 | 0,04 | High_in_IC75 |
| GO_INTRA_GOLGI_VESICLE_MEDIATED_TRANSPORT | 43 | 1,911 | 0,04 | High_in_IC75 |
| GO_POSITIVE_REGULATION_OF_PROTEIN_SECRETION | 142 | 1,909 | 0,04 | High_in_IC75 |
| GO_REGULATION_OF_TYROSINE_PHOSPHORYLATION_OF_STAT3_PROTEIN | 33 | 1,904 | 0,04 | High_in_IC75 |
| GO_MYELOID_LEUKOCYTE_DIFFERENTIATION | 70 | 1,9 | 0,04 | High_in_IC75 |
| GO_CARDIAC_SEPTUM_DEVELOPMENT | 60 | 1,898 | 0,04 | High_in_IC75 |
| GO_STRESS_ACTIVATED_PROTEIN_KINASE_SIGNALING_CASCADE | 85 | 1,898 | 0,04 | High_in_IC75 |
| GO_RETROGRADE_VESICLE_MEDIATED_TRANSPORT_GOLGI_TO_ER | 74 | 1,894 | 0,04 | High_in_IC75 |
| GO_REGULATION_OF_PEPTIDYL_THREONINE_PHOSPHORYLATION | 31 | 1,893 | 0,04 | High_in_IC75 |
| GO_REGULATION_OF_RHO_PROTEIN_SIGNAL_TRANSDUCTION | 81 | 1,891 | 0,04 | High_in_IC75 |
| GO_NEUTROPHIL_MEDIATED_IMMUNITY | 15 | 1,883 | 0,04 | High_in_IC75 |
| GO_PHOSPHATIDYLINOSITOL_DEPHOSPHORYLATION | 18 | 1,883 | 0,04 | High_in_IC75 |
| GO_POSITIVE_REGULATION_OF_GROWTH | 166 | 1,883 | 0,04 | High_in_IC75 |
| GO_LEUKOCYTE_CHEMOTAXIS | 74 | 1,881 | 0,04 | High_in_IC75 |
| GO_CIRCADIAN_RHYTHM | 100 | 1,88 | 0,04 | High_in_IC75 |
| GO_FEMALE_GAMETE_GENERATION | 68 | 1,879 | 0,04 | High_in_IC75 |
| GO_PHOSPHATIDIC_ACID_METABOLIC_PROCESS | 23 | 1,879 | 0,04 | High_in_IC75 |
| GO_DENDRITE_DEVELOPMENT | 55 | 1,878 | 0,04 | High_in_IC75 |
| GO_NEGATIVE_REGULATION_OF_MUSCLE_CELL_DIFFERENTIATION | 38 | 1,878 | 0,04 | High_in_IC75 |
| GO_REGULATION_OF_JUN_KINASE_ACTIVITY | 67 | 1,875 | 0,04 | High_in_IC75 |
| GO_JNK_CASCADE | 66 | 1,872 | 0,04 | High_in_IC75 |
| GO_REGULATION_OF_SMOOTH_MUSCLE_CELL_MIGRATION | 39 | 1,871 | 0,04 | High_in_IC75 |
| GO_NEGATIVE_REGULATION_OF_NEURON_APOPTOTIC_PROCESS | 99 | 1,871 | 0,04 | High_in_IC75 |
| GO_DIGESTION | 67 | 1,871 | 0,04 | High_in_IC75 |
| GO_NEGATIVE_REGULATION_OF_CELL_DIFFERENTIATION | 423 | 1,87 | 0,04 | High_in_IC75 |
| GO_REGULATION_OF_PEPTIDE_TRANSPORT | 169 | 1,87 | 0,04 | High_in_IC75 |
| GO_CELLULAR_RESPONSE_TO_ALCOHOL | 88 | 1,869 | 0,04 | High_in_IC75 |
| GO_REGULATION_OF_MUSCLE_CELL_DIFFERENTIATION | 110 | 1,868 | 0,04 | High_in_IC75 |
| GO_MRNA_TRANSCRIPTION | 17 | 1,867 | 0,05 | High_in_IC75 |
| GO_GASTRULATION | 116 | 1,867 | 0,04 | High_in_IC75 |
| GO_ESTABLISHMENT_OF_PROTEIN_LOCALIZATION_TO_PLASMA_MEMBRANE | 78 | 1,866 | 0,05 | High_in_IC75 |
| GO_POSITIVE_REGULATION_OF_INFLAMMATORY_RESPONSE | 64 | 1,865 | 0,05 | High_in_IC75 |
| GO_FORMATION_OF_PRIMARY_GERM_LAYER | 81 | 1,864 | 0,05 | High_in_IC75 |
| GO_HETEROTYPIC_CELL_CELL_ADHESION | 21 | 1,864 | 0,05 | High_in_IC75 |
| GO_NUCLEOTIDE_SUGAR_BIOSYNTHETIC_PROCESS | 18 | 1,863 | 0,05 | High_in_IC75 |
| GO_LEUKOCYTE_MEDIATED_IMMUNITY | 104 | 1,862 | 0,05 | High_in_IC75 |
| GO_INTERACTION_WITH_HOST | 106 | 1,86 | 0,05 | High_in_IC75 |
| GO_EXTRACELLULAR_STRUCTURE_ORGANIZATION | 214 | 1,858 | 0,05 | High_in_IC75 |
| GO_POSITIVE_REGULATION_OF_CELL_MATRIX_ADHESION | 31 | 1,854 | 0,05 | High_in_IC75 |
| GO_POSITIVE_REGULATION_OF_NF_KAPPAB_IMPORT_INTO_NUCLEUS | 17 | 1,854 | 0,05 | High_in_IC75 |
| GO_POSITIVE_REGULATION_OF_MITOCHONDRIAL_MEMBRANE_PERMEABILITY | 18 | 1,849 | 0,05 | High_in_IC75 |
| GO_PROTEIN_DEPHOSPHORYLATION | 161 | 1,849 | 0,05 | High_in_IC75 |
| GO_NEGATIVE_REGULATION_OF_MAP_KINASE_ACTIVITY | 63 | 1,846 | 0,05 | High_in_IC75 |
| GO_REGULATION_OF_T_CELL_MEDIATED_IMMUNITY | 35 | 1,845 | 0,05 | High_in_IC75 |
| GO_FEMALE_SEX_DIFFERENTIATION | 82 | 1,843 | 0,05 | High_in_IC75 |
| GO_NCRNA_METABOLIC_PROCESS | 493 | -8,043 | 0 | Low_in_IC75 |
| GO_NCRNA_PROCESSING | 359 | -7,841 | 0 | Low_in_IC75 |
| GO_MITOCHONDRIAL_TRANSLATION | 105 | -7,377 | 0 | Low_in_IC75 |
| GO_GLYCOSYL_COMPOUND_METABOLIC_PROCESS | 300 | -7,35 | 0 | Low_in_IC75 |
| GO_NUCLEOBASE_CONTAINING_SMALL_MOLECULE_METABOLIC_PROCESS | 422 | -7,232 | 0 | Low_in_IC75 |
| GO_NUCLEOSIDE_MONOPHOSPHATE_METABOLIC_PROCESS | 205 | -6,991 | 0 | Low_in_IC75 |
| GO_MITOTIC_NUCLEAR_DIVISION | 315 | -6,946 | 0 | Low_in_IC75 |
| GO_CELLULAR_RESPIRATION | 132 | -6,93 | 0 | Low_in_IC75 |
| GO_TRANSLATIONAL_TERMINATION | 92 | -6,923 | 0 | Low_in_IC75 |
| GO_ORGANELLE_FISSION | 403 | -6,88 | 0 | Low_in_IC75 |
| GO_RIBONUCLEOPROTEIN_COMPLEX_BIOGENESIS | 395 | -6,786 | 0 | Low_in_IC75 |
| GO_TRNA_METABOLIC_PROCESS | 166 | -6,741 | 0 | Low_in_IC75 |
| GO_CELL_DIVISION | 400 | -6,653 | 0 | Low_in_IC75 |
| GO_DNA_REPAIR | 423 | -6,563 | 0 | Low_in_IC75 |
| GO_RNA_SPLICING_VIA_TRANSESTERIFICATION_REACTIONS | 228 | -6,539 | 0 | Low_in_IC75 |
| GO_NUCLEOSIDE_TRIPHOSPHATE_METABOLIC_PROCESS | 189 | -6,511 | 0 | Low_in_IC75 |
| GO_TRANSLATIONAL_ELONGATION | 110 | -6,496 | 0 | Low_in_IC75 |
| GO_RNA_SPLICING | 303 | -6,485 | 0 | Low_in_IC75 |
| GO_NUCLEAR_CHROMOSOME_SEGREGATION | 184 | -6,475 | 0 | Low_in_IC75 |
| GO_MACROMOLECULAR_COMPLEX_DISASSEMBLY | 166 | -6,458 | 0 | Low_in_IC75 |
| GO_CELLULAR_PROTEIN_COMPLEX_DISASSEMBLY | 115 | -6,436 | 0 | Low_in_IC75 |
| GO_CHROMOSOME_SEGREGATION | 220 | -6,415 | 0 | Low_in_IC75 |
| GO_SISTER_CHROMATID_SEGREGATION | 158 | -6,41 | 0 | Low_in_IC75 |
| GO_AMIDE_BIOSYNTHETIC_PROCESS | 451 | -6,306 | 0 | Low_in_IC75 |
| GO_OXIDATIVE_PHOSPHORYLATION | 77 | -6,174 | 0 | Low_in_IC75 |
| GO_REGULATION_OF_CELL_CYCLE_PROCESS | 480 | -6,147 | 0 | Low_in_IC75 |
| GO_MRNA_PROCESSING | 353 | -6,147 | 0 | Low_in_IC75 |
| GO_PURINE_CONTAINING_COMPOUND_METABOLIC_PROCESS | 309 | -6,079 | 0 | Low_in_IC75 |
| GO_RIBOSOME_BIOGENESIS | 285 | -6,07 | 0 | Low_in_IC75 |
| GO_GENERATION_OF_PRECURSOR_METABOLITES_AND_ENERGY | 243 | -6,046 | 0 | Low_in_IC75 |
| GO_REGULATION_OF_MITOTIC_CELL_CYCLE | 419 | -6,045 | 0 | Low_in_IC75 |
| GO_PEPTIDE_METABOLIC_PROCESS | 496 | -6,027 | 0 | Low_in_IC75 |
| GO_CELLULAR_COMPONENT_DISASSEMBLY | 422 | -5,973 | 0 | Low_in_IC75 |
| GO_SISTER_CHROMATID_COHESION | 101 | -5,91 | 0 | Low_in_IC75 |
| GO_DNA_REPLICATION | 180 | -5,895 | 0 | Low_in_IC75 |
| GO_ELECTRON_TRANSPORT_CHAIN | 85 | -5,833 | 0 | Low_in_IC75 |
| GO_COFACTOR_METABOLIC_PROCESS | 273 | -5,767 | 0 | Low_in_IC75 |
| GO_TRNA_PROCESSING | 106 | -5,735 | 0 | Low_in_IC75 |
| GO_ENERGY_DERIVATION_BY_OXIDATION_OF_ORGANIC_COMPOUNDS | 187 | -5,696 | 0 | Low_in_IC75 |
| GO_REGULATION_OF_CELL_CYCLE_PHASE_TRANSITION | 293 | -5,664 | 0 | Low_in_IC75 |
| GO_ANAPHASE_PROMOTING_COMPLEX_DEPENDENT_CATABOLIC_PROCESS | 73 | -5,66 | 0 | Low_in_IC75 |
| GO_MITOCHONDRIAL_RESPIRATORY_CHAIN_COMPLEX_ASSEMBLY | 69 | -5,639 | 0 | Low_in_IC75 |
| GO_POSTTRANSCRIPTIONAL_REGULATION_OF_GENE_EXPRESSION | 396 | -5,529 | 0 | Low_in_IC75 |
| GO_MITOCHONDRIAL_RESPIRATORY_CHAIN_COMPLEX_I_BIOGENESIS | 52 | -5,528 | 0 | Low_in_IC75 |
| GO_GLYCOSYL_COMPOUND_BIOSYNTHETIC_PROCESS | 103 | -5,339 | 0 | Low_in_IC75 |
| GO_REGULATION_OF_RNA_STABILITY | 126 | -5,236 | 0 | Low_in_IC75 |
| GO_COENZYME_METABOLIC_PROCESS | 215 | -5,23 | 0 | Low_in_IC75 |
| GO_RRNA_METABOLIC_PROCESS | 237 | -5,22 | 0 | Low_in_IC75 |
| GO_DNA_RECOMBINATION | 176 | -5,072 | 0 | Low_in_IC75 |
| GO_RNA_LOCALIZATION | 154 | -5,028 | 0 | Low_in_IC75 |
| GO_RNA_MODIFICATION | 105 | -5,018 | 0 | Low_in_IC75 |
| GO_CELL_CYCLE_PHASE_TRANSITION | 220 | -4,994 | 0 | Low_in_IC75 |
| GO_NUCLEOSIDE_MONOPHOSPHATE_BIOSYNTHETIC_PROCESS | 73 | -4,979 | 0 | Low_in_IC75 |
| GO_DNA_DEPENDENT_DNA_REPLICATION | 85 | -4,884 | 0 | Low_in_IC75 |
| GO_POSITIVE_REGULATION_OF_LIGASE_ACTIVITY | 103 | -4,877 | 0 | Low_in_IC75 |
| GO_REGULATION_OF_LIGASE_ACTIVITY | 122 | -4,816 | 0 | Low_in_IC75 |
| GO_CELLULAR_AMINO_ACID_METABOLIC_PROCESS | 258 | -4,796 | 0 | Low_in_IC75 |
| GO_REGULATION_OF_PROTEIN_UBIQUITINATION_INVOLVED_IN_UBIQUITIN_DEPENDENT_PROTEIN_CATABOLIC_PROCESS | 94 | -4,788 | 0 | Low_in_IC75 |
| GO_NEGATIVE_REGULATION_OF_PROTEIN_MODIFICATION_BY_SMALL_PROTEIN_CONJUGATION_OR_REMOVAL | 127 | -4,771 | 0 | Low_in_IC75 |
| GO_NUCLEOSIDE_PHOSPHATE_BIOSYNTHETIC_PROCESS | 148 | -4,754 | 0 | Low_in_IC75 |
| GO_MITOTIC_SISTER_CHROMATID_SEGREGATION | 80 | -4,691 | 0 | Low_in_IC75 |
| GO_CELLULAR_PROTEIN_COMPLEX_ASSEMBLY | 288 | -4,681 | 0 | Low_in_IC75 |
| GO_COFACTOR_BIOSYNTHETIC_PROCESS | 140 | -4,678 | 0 | Low_in_IC75 |
| GO_PURINE_NUCLEOSIDE_MONOPHOSPHATE_BIOSYNTHETIC_PROCESS | 54 | -4,666 | 0 | Low_in_IC75 |
| GO_NUCLEOBASE_CONTAINING_COMPOUND_TRANSPORT | 165 | -4,641 | 0 | Low_in_IC75 |
| GO_NUCLEIC_ACID_PHOSPHODIESTER_BOND_HYDROLYSIS | 217 | -4,622 | 0 | Low_in_IC75 |
| GO_REGULATION_OF_CHROMOSOME_SEGREGATION | 77 | -4,598 | 0 | Low_in_IC75 |
| GO_PURINE_NUCLEOSIDE_BIOSYNTHETIC_PROCESS | 77 | -4,578 | 0 | Low_in_IC75 |
| GO_RIBONUCLEOPROTEIN_COMPLEX_LOCALIZATION | 99 | -4,558 | 0 | Low_in_IC75 |
| GO_POSITIVE_REGULATION_OF_PROTEIN_MODIFICATION_BY_SMALL_PROTEIN_CONJUGATION_OR_REMOVAL | 181 | -4,557 | 0 | Low_in_IC75 |
| GO_NUCLEAR_TRANSPORT | 300 | -4,555 | 0 | Low_in_IC75 |
| GO_MITOCHONDRIAL_TRANSMEMBRANE_TRANSPORT | 49 | -4,552 | 0 | Low_in_IC75 |
| GO_REGULATION_OF_PROTEIN_MODIFICATION_BY_SMALL_PROTEIN_CONJUGATION_OR_REMOVAL | 256 | -4,547 | 0 | Low_in_IC75 |
| GO_NUCLEAR_EXPORT | 125 | -4,542 | 0 | Low_in_IC75 |
| GO_REGULATION_OF_MICROTUBULE_BASED_PROCESS | 205 | -4,54 | 0 | Low_in_IC75 |
| GO_DNA_TEMPLATED_TRANSCRIPTION_TERMINATION | 84 | -4,528 | 0 | Low_in_IC75 |
| GO_DNA_BIOSYNTHETIC_PROCESS | 100 | -4,478 | 0 | Low_in_IC75 |
| GO_NUCLEOTIDE_EXCISION_REPAIR | 105 | -4,454 | 0 | Low_in_IC75 |
| GO_REGULATION_OF_CELLULAR_AMINO_ACID_METABOLIC_PROCESS | 54 | -4,445 | 0 | Low_in_IC75 |
| GO_REGULATION_OF_CELL_DIVISION | 209 | -4,36 | 0 | Low_in_IC75 |
| GO_REGULATION_OF_CHROMOSOME_ORGANIZATION | 237 | -4,339 | 0 | Low_in_IC75 |
| GO_CELL_CYCLE_CHECKPOINT | 178 | -4,282 | 0 | Low_in_IC75 |
| GO_REGULATION_OF_CELLULAR_PROTEIN_CATABOLIC_PROCESS | 246 | -4,222 | 0 | Low_in_IC75 |
| GO_REGULATION_OF_CELLULAR_AMINE_METABOLIC_PROCESS | 70 | -4,217 | 0 | Low_in_IC75 |
| GO_AEROBIC_RESPIRATION | 50 | -4,187 | 0 | Low_in_IC75 |
| GO_TRNA_MODIFICATION | 54 | -4,171 | 0 | Low_in_IC75 |
| GO_PURINE_CONTAINING_COMPOUND_BIOSYNTHETIC_PROCESS | 110 | -4,168 | 0 | Low_in_IC75 |
| GO_NEGATIVE_REGULATION_OF_ORGANELLE_ORGANIZATION | 331 | -4,161 | 0 | Low_in_IC75 |
| GO_ORGANOPHOSPHATE_BIOSYNTHETIC_PROCESS | 362 | -4,157 | 0 | Low_in_IC75 |
| GO_MITOCHONDRIAL_TRANSPORT | 159 | -4,137 | 0 | Low_in_IC75 |
| GO_ANTIGEN_PROCESSING_AND_PRESENTATION_OF_EXOGENOUS_PEPTIDE_ANTIGEN_VIA_MHC_CLASS_I | 59 | -4,117 | 0 | Low_in_IC75 |
| GO_NEGATIVE_REGULATION_OF_MITOTIC_CELL_CYCLE | 183 | -4,112 | 0 | Low_in_IC75 |
| GO_REGULATION_OF_SISTER_CHROMATID_SEGREGATION | 61 | -4,104 | 0 | Low_in_IC75 |
| GO_RNA_3_END_PROCESSING | 78 | -4,071 | 0 | Low_in_IC75 |
| GO_REGULATION_OF_CELLULAR_RESPONSE_TO_HEAT | 74 | -4,062 | 0 | Low_in_IC75 |
| GO_COENZYME_BIOSYNTHETIC_PROCESS | 106 | -4,044 | 0 | Low_in_IC75 |
| GO_POSITIVE_REGULATION_OF_CELLULAR_PROTEIN_CATABOLIC_PROCESS | 176 | -4,028 | 0 | Low_in_IC75 |
| GO_SMALL_MOLECULE_CATABOLIC_PROCESS | 254 | -4,012 | 0 | Low_in_IC75 |
| GO_CARBOHYDRATE_DERIVATIVE_BIOSYNTHETIC_PROCESS | 459 | -4,005 | 0 | Low_in_IC75 |
| GO_REGULATION_OF_ESTABLISHMENT_OF_PLANAR_POLARITY | 99 | -3,987 | 0 | Low_in_IC75 |
| GO_REGULATION_OF_NUCLEAR_DIVISION | 131 | -3,962 | 0 | Low_in_IC75 |
| GO_PROTEIN_SUMOYLATION | 110 | -3,959 | 0 | Low_in_IC75 |
| GO_MEIOTIC_CELL_CYCLE | 132 | -3,958 | 0 | Low_in_IC75 |
| GO_RIBONUCLEOSIDE_TRIPHOSPHATE_BIOSYNTHETIC_PROCESS | 44 | -3,947 | 0 | Low_in_IC75 |
| GO_RNA_PHOSPHODIESTER_BOND_HYDROLYSIS | 104 | -3,942 | 0 | Low_in_IC75 |
| GO_REGULATION_OF_CELLULAR_KETONE_METABOLIC_PROCESS | 128 | -3,938 | 0 | Low_in_IC75 |
| GO_NIK_NF_KAPPAB_SIGNALING | 79 | -3,896 | 0 | Low_in_IC75 |
| GO_TERMINATION_OF_RNA_POLYMERASE_II_TRANSCRIPTION | 46 | -3,881 | 0 | Low_in_IC75 |
| GO_POSITIVE_REGULATION_OF_PROTEOLYSIS | 312 | -3,877 | 0 | Low_in_IC75 |
| GO_SMALL_MOLECULE_BIOSYNTHETIC_PROCESS | 347 | -3,873 | 0 | Low_in_IC75 |
| GO_PROTEIN_IMPORT | 129 | -3,872 | 0 | Low_in_IC75 |
| GO_TRANSCRIPTION_COUPLED_NUCLEOTIDE_EXCISION_REPAIR | 72 | -3,849 | 0 | Low_in_IC75 |
| GO_CELL_CYCLE_G2_M_PHASE_TRANSITION | 120 | -3,842 | 0 | Low_in_IC75 |
| GO_ATP_DEPENDENT_CHROMATIN_REMODELING | 63 | -3,832 | 0 | Low_in_IC75 |
| GO_RIBONUCLEOPROTEIN_COMPLEX_SUBUNIT_ORGANIZATION | 171 | -3,828 | 0 | Low_in_IC75 |
| GO_TRNA_TRANSPORT | 32 | -3,824 | 0 | Low_in_IC75 |
| GO_PROTEIN_DNA_COMPLEX_SUBUNIT_ORGANIZATION | 180 | -3,816 | 0 | Low_in_IC75 |
| GO_DNA_SYNTHESIS_INVOLVED_IN_DNA_REPAIR | 62 | -3,815 | 0 | Low_in_IC75 |
| GO_MEMBRANE_DISASSEMBLY | 43 | -3,809 | 0 | Low_in_IC75 |
| GO_DNA_CONFORMATION_CHANGE | 222 | -3,805 | 0 | Low_in_IC75 |
| GO_MITOCHONDRIAL_ATP_SYNTHESIS_COUPLED_PROTON_TRANSPORT | 17 | -3,802 | 0 | Low_in_IC75 |
| GO_NEGATIVE_REGULATION_OF_CELL_CYCLE_PROCESS | 192 | -3,799 | 0 | Low_in_IC75 |
| GO_REGULATION_OF_ORGAN_MORPHOGENESIS | 188 | -3,785 | 0 | Low_in_IC75 |
| GO_PROTEIN_LOCALIZATION_TO_ORGANELLE | 484 | -3,779 | 0 | Low_in_IC75 |
| GO_TELOMERE_MAINTENANCE_VIA_RECOMBINATION | 31 | -3,776 | 0 | Low_in_IC75 |
| GO_DOUBLE_STRAND_BREAK_REPAIR | 134 | -3,775 | 0 | Low_in_IC75 |
| GO_MEIOTIC_CELL_CYCLE_PROCESS | 105 | -3,753 | 0 | Low_in_IC75 |
| GO_MITOTIC_CELL_CYCLE_CHECKPOINT | 129 | -3,741 | 0 | Low_in_IC75 |
| GO_ANTIGEN_PROCESSING_AND_PRESENTATION_OF_PEPTIDE_ANTIGEN_VIA_MHC_CLASS_I | 78 | -3,739 | 0 | Low_in_IC75 |
| GO_NEGATIVE_REGULATION_OF_CELL_CYCLE | 379 | -3,716 | 0 | Low_in_IC75 |
| GO_ORGANIC_CYCLIC_COMPOUND_CATABOLIC_PROCESS | 345 | -3,702 | 0 | Low_in_IC75 |
| GO_REGULATION_OF_DNA_METABOLIC_PROCESS | 279 | -3,7 | 0 | Low_in_IC75 |
| GO_DNA_STRAND_ELONGATION_INVOLVED_IN_DNA_REPLICATION | 25 | -3,682 | 0 | Low_in_IC75 |
| GO_MITOTIC_RECOMBINATION | 38 | -3,679 | 0 | Low_in_IC75 |
| GO_ESTABLISHMENT_OF_PROTEIN_LOCALIZATION_TO_ORGANELLE | 323 | -3,668 | 0 | Low_in_IC75 |
| GO_REGULATION_OF_TELOMERASE_RNA_LOCALIZATION_TO_CAJAL_BODY | 15 | -3,661 | 0 | Low_in_IC75 |
| GO_REGULATION_OF_MICROTUBULE_POLYMERIZATION_OR_DEPOLYMERIZATION | 153 | -3,661 | 0 | Low_in_IC75 |
| GO_ATP_BIOSYNTHETIC_PROCESS | 31 | -3,66 | 0 | Low_in_IC75 |
| GO_NUCLEOSIDE_TRIPHOSPHATE_BIOSYNTHETIC_PROCESS | 52 | -3,66 | 0 | Low_in_IC75 |
| GO_MICROTUBULE_BASED_PROCESS | 406 | -3,654 | 0 | Low_in_IC75 |
| GO_RIBOSOMAL_SMALL_SUBUNIT_BIOGENESIS | 55 | -3,654 | 0 | Low_in_IC75 |
| GO_REGULATION_OF_PROTEIN_CATABOLIC_PROCESS | 346 | -3,649 | 0 | Low_in_IC75 |
| GO_NON_CANONICAL_WNT_SIGNALING_PATHWAY | 125 | -3,645 | 0 | Low_in_IC75 |
| GO_MULTI_ORGANISM_LOCALIZATION | 64 | -3,623 | 0 | Low_in_IC75 |
| GO_DNA_STRAND_ELONGATION | 29 | -3,612 | 0 | Low_in_IC75 |
| GO_DNA_REPLICATION_INITIATION | 23 | -3,607 | 0 | Low_in_IC75 |
| GO_NUCLEAR_IMPORT | 111 | -3,567 | 0 | Low_in_IC75 |
| GO_MITOCHONDRIAL_RNA_METABOLIC_PROCESS | 26 | -3,544 | 0 | Low_in_IC75 |
| GO_NUCLEAR_ENVELOPE_ORGANIZATION | 72 | -3,543 | 0 | Low_in_IC75 |
| GO_PROTEIN_TARGETING_TO_MITOCHONDRION | 46 | -3,538 | 0 | Low_in_IC75 |
| GO_DNA_GEOMETRIC_CHANGE | 78 | -3,501 | 0 | Low_in_IC75 |
| GO_ANTIGEN_PROCESSING_AND_PRESENTATION_OF_PEPTIDE_ANTIGEN | 143 | -3,494 | 0 | Low_in_IC75 |
| GO_CELL_CYCLE_G1_S_PHASE_TRANSITION | 94 | -3,491 | 0 | Low_in_IC75 |
| GO_ORGANIC_ACID_BIOSYNTHETIC_PROCESS | 204 | -3,473 | 0 | Low_in_IC75 |
| GO_CELLULAR_ALDEHYDE_METABOLIC_PROCESS | 66 | -3,464 | 0 | Low_in_IC75 |
| GO_METHYLATION | 218 | -3,431 | 0 | Low_in_IC75 |
| GO_POSITIVE_REGULATION_OF_PROTEIN_CATABOLIC_PROCESS | 233 | -3,424 | 0 | Low_in_IC75 |
| GO_PROTEIN_TARGETING | 360 | -3,418 | 0 | Low_in_IC75 |
| GO_RNA_PHOSPHODIESTER_BOND_HYDROLYSIS_ENDONUCLEOLYTIC | 50 | -3,416 | 0 | Low_in_IC75 |
| GO_NEGATIVE_REGULATION_OF_CYTOSKELETON_ORGANIZATION | 185 | -3,408 | 0 | Low_in_IC75 |
| GO_REGULATION_OF_CELLULAR_AMIDE_METABOLIC_PROCESS | 307 | -3,359 | 0 | Low_in_IC75 |
| GO_ENERGY_COUPLED_PROTON_TRANSPORT_DOWN_ELECTROCHEMICAL_GRADIENT | 22 | -3,345 | 0 | Low_in_IC75 |
| GO_METAPHASE_PLATE_CONGRESSION | 38 | -3,344 | 0 | Low_in_IC75 |
| GO_MITOTIC_SPINDLE_ORGANIZATION | 62 | -3,339 | 0 | Low_in_IC75 |
| GO_DNA_INTEGRITY_CHECKPOINT | 135 | -3,334 | 0 | Low_in_IC75 |
| GO_OXIDOREDUCTION_COENZYME_METABOLIC_PROCESS | 89 | -3,324 | 0 | Low_in_IC75 |
| GO_ORGANELLE_ASSEMBLY | 384 | -3,32 | 0 | Low_in_IC75 |
| GO_CHROMOSOME_LOCALIZATION | 51 | -3,297 | 0 | Low_in_IC75 |
| GO_NEGATIVE_REGULATION_OF_CELL_CYCLE_PHASE_TRANSITION | 133 | -3,296 | 0 | Low_in_IC75 |
| GO_TRICARBOXYLIC_ACID_METABOLIC_PROCESS | 34 | -3,293 | 0 | Low_in_IC75 |
| GO_RNA_CATABOLIC_PROCESS | 210 | -3,288 | 0 | Low_in_IC75 |
| GO_DNA_REPLICATION_INDEPENDENT_NUCLEOSOME_ORGANIZATION | 46 | -3,273 | 0,000005 | Low_in_IC75 |
| GO_DNA_PACKAGING | 146 | -3,268 | 0,000005 | Low_in_IC75 |
| GO_CHROMATIN_ASSEMBLY_OR_DISASSEMBLY | 139 | -3,265 | 0,000005 | Low_in_IC75 |
| GO_ORGANIC_ACID_CATABOLIC_PROCESS | 156 | -3,252 | 0,000005 | Low_in_IC75 |
| GO_PEPTIDYL_LYSINE_MODIFICATION | 273 | -3,25 | 0,000005 | Low_in_IC75 |
| GO_REGULATION_OF_DNA_REPLICATION | 134 | -3,244 | 0,000005 | Low_in_IC75 |
| GO_RECOMBINATIONAL_REPAIR | 62 | -3,242 | 0,000005 | Low_in_IC75 |
| GO_TUMOR_NECROSIS_FACTOR_MEDIATED_SIGNALING_PATHWAY | 99 | -3,23 | 0,000005 | Low_in_IC75 |
| GO_MRNA_3_END_PROCESSING | 55 | -3,215 | 0,000005 | Low_in_IC75 |
| GO_POSITIVE_REGULATION_OF_DNA_METABOLIC_PROCESS | 148 | -3,212 | 0,000005 | Low_in_IC75 |
| GO_SULFUR_COMPOUND_METABOLIC_PROCESS | 272 | -3,211 | 0,000004 | Low_in_IC75 |
| GO_PROTEIN_LOCALIZATION_TO_MITOCHONDRION | 62 | -3,204 | 0,000004 | Low_in_IC75 |
| GO_RNA_METHYLATION | 45 | -3,174 | 0,000004 | Low_in_IC75 |
| GO_SPINDLE_ASSEMBLY | 59 | -3,164 | 0,000004 | Low_in_IC75 |
| GO_PROTEASOMAL_PROTEIN_CATABOLIC_PROCESS | 243 | -3,163 | 0,000004 | Low_in_IC75 |
| GO_MONOCARBOXYLIC_ACID_METABOLIC_PROCESS | 364 | -3,152 | 0,000004 | Low_in_IC75 |
| GO_NEGATIVE_REGULATION_OF_MITOTIC_NUCLEAR_DIVISION | 31 | -3,149 | 0,000004 | Low_in_IC75 |
| GO_NEGATIVE_REGULATION_OF_PROTEIN_COMPLEX_DISASSEMBLY | 142 | -3,146 | 0,000004 | Low_in_IC75 |
| GO_ALPHA_AMINO_ACID_METABOLIC_PROCESS | 173 | -3,131 | 0,000004 | Low_in_IC75 |
| GO_SPINDLE_CHECKPOINT | 23 | -3,124 | 0,000004 | Low_in_IC75 |
| GO_POSITIVE_REGULATION_OF_CHROMOSOME_ORGANIZATION | 130 | -3,122 | 0,000004 | Low_in_IC75 |
| GO_DNA_DAMAGE_RESPONSE_DETECTION_OF_DNA_DAMAGE | 35 | -3,114 | 0,000004 | Low_in_IC75 |
| GO_MATURATION_OF_SSU_RRNA | 39 | -3,112 | 0,000004 | Low_in_IC75 |
| GO_REGULATION_OF_SIGNAL_TRANSDUCTION_BY_P53_CLASS_MEDIATOR | 144 | -3,109 | 0,000004 | Low_in_IC75 |
| GO_NEGATIVE_REGULATION_OF_CELL_DIVISION | 51 | -3,108 | 0,000004 | Low_in_IC75 |
| GO_POSITIVE_REGULATION_OF_CANONICAL_WNT_SIGNALING_PATHWAY | 104 | -3,098 | 0,000004 | Low_in_IC75 |
| GO_MEIOTIC_CHROMOSOME_SEGREGATION | 36 | -3,094 | 0,000004 | Low_in_IC75 |
| GO_MEIOSIS_I | 53 | -3,09 | 0,000004 | Low_in_IC75 |
| GO_PROTEIN_TRANSMEMBRANE_TRANSPORT | 42 | -3,086 | 0,000004 | Low_in_IC75 |
| GO_NCRNA_3_END_PROCESSING | 20 | -3,078 | 0,000004 | Low_in_IC75 |
| GO_INNATE_IMMUNE_RESPONSE_ACTIVATING_CELL_SURFACE_RECEPTOR_SIGNALING_PATHWAY | 92 | -3,065 | 0,000004 | Low_in_IC75 |
| GO_ANTIGEN_PROCESSING_AND_PRESENTATION | 171 | -3,057 | 0,000008 | Low_in_IC75 |
| GO_POSTREPLICATION_REPAIR | 49 | -3,057 | 0,000008 | Low_in_IC75 |
| GO_NCRNA_TRANSCRIPTION | 86 | -3,028 | 0,000008 | Low_in_IC75 |
| GO_CHROMATIN_MODIFICATION | 455 | -3,026 | 0,000008 | Low_in_IC75 |
| GO_SPLICEOSOMAL_SNRNP_ASSEMBLY | 33 | -3,016 | 0,000008 | Low_in_IC75 |
| GO_TRNA_METHYLATION | 21 | -3,009 | 0,000008 | Low_in_IC75 |
| GO_NEGATIVE_REGULATION_OF_NUCLEAR_DIVISION | 39 | -3,007 | 0,000008 | Low_in_IC75 |
| GO_TELOMERE_ORGANIZATION | 90 | -3,004 | 0,000008 | Low_in_IC75 |
| GO_REGULATION_OF_CENTROSOME_CYCLE | 34 | -2,996 | 0,000008 | Low_in_IC75 |
| GO_CELLULAR_AMINO_ACID_BIOSYNTHETIC_PROCESS | 73 | -2,992 | 0,000008 | Low_in_IC75 |
| GO_REGULATION_OF_PROTEASOMAL_PROTEIN_CATABOLIC_PROCESS | 164 | -2,984 | 0,000008 | Low_in_IC75 |
| GO_SNRNA_PROCESSING | 22 | -2,965 | 0,000007 | Low_in_IC75 |
| GO_TRANSLESION_SYNTHESIS | 37 | -2,965 | 0,000007 | Low_in_IC75 |
| GO_DNA_DOUBLE_STRAND_BREAK_PROCESSING | 16 | -2,963 | 0,000007 | Low_in_IC75 |
| GO_THIOESTER_METABOLIC_PROCESS | 67 | -2,951 | 0,00001 | Low_in_IC75 |
| GO_FC_EPSILON_RECEPTOR_SIGNALING_PATHWAY | 113 | -2,951 | 0,00001 | Low_in_IC75 |
| GO_CENTROMERE_COMPLEX_ASSEMBLY | 41 | -2,951 | 0,00001 | Low_in_IC75 |
| GO_NUCLEOBASE_BIOSYNTHETIC_PROCESS | 17 | -2,95 | 0,00001 | Low_in_IC75 |
| GO_CHROMATIN_REMODELING | 130 | -2,947 | 0,00001 | Low_in_IC75 |
| GO_NEGATIVE_REGULATION_OF_CANONICAL_WNT_SIGNALING_PATHWAY | 134 | -2,94 | 0,00001 | Low_in_IC75 |
| GO_SNRNA_METABOLIC_PROCESS | 80 | -2,939 | 0,00001 | Low_in_IC75 |
| GO_GENE_SILENCING_BY_RNA | 117 | -2,927 | 0,00001 | Low_in_IC75 |
| GO_REGULATION_OF_TELOMERE_MAINTENANCE | 59 | -2,925 | 0,00001 | Low_in_IC75 |
| GO_NITROGEN_COMPOUND_TRANSPORT | 362 | -2,923 | 0,00001 | Low_in_IC75 |
| GO_INNER_MITOCHONDRIAL_MEMBRANE_ORGANIZATION | 17 | -2,914 | 0,00001 | Low_in_IC75 |
| GO_REGULATION_OF_TELOMERE_MAINTENANCE_VIA_TELOMERE_LENGTHENING | 45 | -2,911 | 0,00001 | Low_in_IC75 |
| GO_HYDROGEN_ION_TRANSMEMBRANE_TRANSPORT | 79 | -2,909 | 0,00001 | Low_in_IC75 |
| GO_T_CELL_RECEPTOR_SIGNALING_PATHWAY | 112 | -2,908 | 0,00001 | Low_in_IC75 |
| GO_MICROTUBULE_CYTOSKELETON_ORGANIZATION | 273 | -2,901 | 0,00002 | Low_in_IC75 |
| GO_AMINO_ACID_ACTIVATION | 50 | -2,897 | 0,00002 | Low_in_IC75 |
| GO_REGULATION_OF_PROTEIN_COMPLEX_DISASSEMBLY | 185 | -2,894 | 0,00003 | Low_in_IC75 |
| GO_RIBOSOMAL_LARGE_SUBUNIT_BIOGENESIS | 48 | -2,885 | 0,00003 | Low_in_IC75 |
| GO_POSITIVE_REGULATION_OF_CELL_CYCLE_PROCESS | 212 | -2,882 | 0,00003 | Low_in_IC75 |
| GO_NEGATIVE_REGULATION_OF_TRANSFERASE_ACTIVITY | 288 | -2,882 | 0,00003 | Low_in_IC75 |
| GO_CARBOHYDRATE_METABOLIC_PROCESS | 482 | -2,864 | 0,00003 | Low_in_IC75 |
| GO_NEGATIVE_REGULATION_OF_CHROMOSOME_ORGANIZATION | 85 | -2,863 | 0,00003 | Low_in_IC75 |
| GO_RRNA_MODIFICATION | 20 | -2,861 | 0,00003 | Low_in_IC75 |
| GO_ALPHA_AMINO_ACID_BIOSYNTHETIC_PROCESS | 62 | -2,85 | 0,00004 | Low_in_IC75 |
| GO_REGULATION_OF_DNA_BIOSYNTHETIC_PROCESS | 76 | -2,843 | 0,00004 | Low_in_IC75 |
| GO_NUCLEOBASE_METABOLIC_PROCESS | 33 | -2,831 | 0,00004 | Low_in_IC75 |
| GO_NEGATIVE_REGULATION_OF_CHROMOSOME_SEGREGATION | 25 | -2,827 | 0,00004 | Low_in_IC75 |
| GO_MULTI_ORGANISM_METABOLIC_PROCESS | 136 | -2,825 | 0,00004 | Low_in_IC75 |
| GO_GENE_SILENCING | 170 | -2,817 | 0,00004 | Low_in_IC75 |
| GO_HYDROGEN_TRANSPORT | 102 | -2,815 | 0,00004 | Low_in_IC75 |
| GO_POSITIVE_REGULATION_OF_TRANSFERASE_ACTIVITY | 497 | -2,813 | 0,00005 | Low_in_IC75 |
| GO_DICARBOXYLIC_ACID_METABOLIC_PROCESS | 81 | -2,801 | 0,00006 | Low_in_IC75 |
| GO_POSITIVE_REGULATION_OF_CELL_CYCLE | 289 | -2,796 | 0,00007 | Low_in_IC75 |
| GO_WNT_SIGNALING_PATHWAY | 292 | -2,793 | 0,00008 | Low_in_IC75 |
| GO_NUCLEOTIDE_EXCISION_REPAIR_DNA_INCISION | 37 | -2,789 | 0,00008 | Low_in_IC75 |
| GO_LIPOSACCHARIDE_METABOLIC_PROCESS | 85 | -2,787 | 0,00008 | Low_in_IC75 |
| GO_PROTEIN_POLYUBIQUITINATION | 229 | -2,784 | 0,00008 | Low_in_IC75 |
| GO_CYTOCHROME_COMPLEX_ASSEMBLY | 22 | -2,77 | 0,00009 | Low_in_IC75 |
| GO_CYTOKINESIS | 75 | -2,763 | 0,0001 | Low_in_IC75 |
| GO_REGULATION_OF_PROTEASOMAL_UBIQUITIN_DEPENDENT_PROTEIN_CATABOLIC_PROCESS | 134 | -2,757 | 0,0001 | Low_in_IC75 |
| GO_TRANSCRIPTION_FROM_RNA_POLYMERASE_I_PROMOTER | 35 | -2,751 | 0,0001 | Low_in_IC75 |
| GO_MATURATION_OF_SSU_RRNA_FROM_TRICISTRONIC_RRNA_TRANSCRIPT_SSU_RRNA_5_8S_RRNA_LSU_RRNA_ | 30 | -2,751 | 0,0001 | Low_in_IC75 |
| GO_SULFUR_COMPOUND_BIOSYNTHETIC_PROCESS | 157 | -2,75 | 0,0001 | Low_in_IC75 |
| GO_POSITIVE_REGULATION_OF_CELL_DIVISION | 94 | -2,738 | 0,0001 | Low_in_IC75 |
| GO_CELLULAR_MODIFIED_AMINO_ACID_METABOLIC_PROCESS | 159 | -2,729 | 0,0001 | Low_in_IC75 |
| GO_PSEUDOURIDINE_SYNTHESIS | 17 | -2,728 | 0,0001 | Low_in_IC75 |
| GO_CELL_PROLIFERATION | 497 | -2,728 | 0,0001 | Low_in_IC75 |
| GO_THIOESTER_BIOSYNTHETIC_PROCESS | 44 | -2,725 | 0,0001 | Low_in_IC75 |
| GO_ANTIGEN_RECEPTOR_MEDIATED_SIGNALING_PATHWAY | 130 | -2,722 | 0,0001 | Low_in_IC75 |
| GO_NEGATIVE_REGULATION_OF_WNT_SIGNALING_PATHWAY | 161 | -2,713 | 0,0002 | Low_in_IC75 |
| GO_NUCLEAR_TRANSCRIBED_MRNA_CATABOLIC_PROCESS_DEADENYLATION_DEPENDENT_DECAY | 54 | -2,709 | 0,0002 | Low_in_IC75 |
| GO_LIPID_OXIDATION | 53 | -2,706 | 0,0002 | Low_in_IC75 |
| GO_GPI_ANCHOR_METABOLIC_PROCESS | 30 | -2,702 | 0,0002 | Low_in_IC75 |
| GO_RNA_CAPPING | 37 | -2,7 | 0,0002 | Low_in_IC75 |
| GO_POSITIVE_REGULATION_OF_DNA_REPAIR | 33 | -2,7 | 0,0002 | Low_in_IC75 |
| GO_STRAND_DISPLACEMENT | 21 | -2,69 | 0,0002 | Low_in_IC75 |
| GO_GLYCOLIPID_BIOSYNTHETIC_PROCESS | 47 | -2,687 | 0,0002 | Low_in_IC75 |
| GO_POSITIVE_REGULATION_OF_CHROMOSOME_SEGREGATION | 24 | -2,677 | 0,0002 | Low_in_IC75 |
| GO_POSITIVE_REGULATION_OF_CATABOLIC_PROCESS | 344 | -2,674 | 0,0002 | Low_in_IC75 |
| GO_MICROTUBULE_BASED_MOVEMENT | 154 | -2,668 | 0,0002 | Low_in_IC75 |
| GO_MITOCHONDRIAL_MEMBRANE_ORGANIZATION | 87 | -2,668 | 0,0002 | Low_in_IC75 |
| GO_POSITIVE_REGULATION_OF_DNA_BIOSYNTHETIC_PROCESS | 50 | -2,657 | 0,0002 | Low_in_IC75 |
| GO_REGULATION_OF_DNA_REPAIR | 65 | -2,656 | 0,0002 | Low_in_IC75 |
| GO_MICROTUBULE_ORGANIZING_CENTER_ORGANIZATION | 73 | -2,656 | 0,0002 | Low_in_IC75 |
| GO_NUCLEAR_TRANSCRIBED_MRNA_CATABOLIC_PROCESS_EXONUCLEOLYTIC | 30 | -2,646 | 0,0002 | Low_in_IC75 |
| GO_NUCLEOTIDE_EXCISION_REPAIR_DNA_GAP_FILLING | 23 | -2,644 | 0,0003 | Low_in_IC75 |
| GO_CILIUM_MORPHOGENESIS | 152 | -2,639 | 0,0003 | Low_in_IC75 |
| GO_MITOTIC_DNA_INTEGRITY_CHECKPOINT | 94 | -2,634 | 0,0003 | Low_in_IC75 |
| GO_CELLULAR_MODIFIED_AMINO_ACID_BIOSYNTHETIC_PROCESS | 41 | -2,633 | 0,0003 | Low_in_IC75 |
| GO_FATTY_ACID_BETA_OXIDATION | 39 | -2,631 | 0,0003 | Low_in_IC75 |
| GO_CILIUM_ORGANIZATION | 134 | -2,631 | 0,0003 | Low_in_IC75 |
| GO_POSITIVE_REGULATION_OF_WNT_SIGNALING_PATHWAY | 127 | -2,629 | 0,0003 | Low_in_IC75 |
| GO_DNA_TEMPLATED_TRANSCRIPTION_ELONGATION | 93 | -2,628 | 0,0003 | Low_in_IC75 |
| GO_PROTEIN_LOCALIZATION_TO_NUCLEUS | 130 | -2,615 | 0,0003 | Low_in_IC75 |
| GO_PROTEIN_FOLDING | 186 | -2,614 | 0,0003 | Low_in_IC75 |
| GO_PROTEIN_LOCALIZATION_TO_CHROMOSOME | 37 | -2,614 | 0,0003 | Low_in_IC75 |
| GO_RIBOSOME_ASSEMBLY | 50 | -2,607 | 0,0003 | Low_in_IC75 |
| GO_PYRIMIDINE_NUCLEOSIDE_BIOSYNTHETIC_PROCESS | 27 | -2,595 | 0,0003 | Low_in_IC75 |
| GO_PURINE_NUCLEOBASE_METABOLIC_PROCESS | 18 | -2,588 | 0,0004 | Low_in_IC75 |
| GO_ASPARTATE_FAMILY_AMINO_ACID_BIOSYNTHETIC_PROCESS | 20 | -2,582 | 0,0004 | Low_in_IC75 |
| GO_HISTONE_EXCHANGE | 43 | -2,582 | 0,0004 | Low_in_IC75 |
| GO_BASE_EXCISION_REPAIR | 38 | -2,573 | 0,0004 | Low_in_IC75 |
| GO_CHROMOSOME_SEPARATION | 16 | -2,571 | 0,0004 | Low_in_IC75 |
| GO_QUINONE_METABOLIC_PROCESS | 26 | -2,568 | 0,0004 | Low_in_IC75 |
| GO_MISMATCH_REPAIR | 28 | -2,563 | 0,0004 | Low_in_IC75 |
| GO_POSITIVE_REGULATION_OF_TELOMERE_MAINTENANCE_VIA_TELOMERE_LENGTHENING | 30 | -2,557 | 0,0004 | Low_in_IC75 |
| GO_MATURATION_OF_5_8S_RRNA_FROM_TRICISTRONIC_RRNA_TRANSCRIPT_SSU_RRNA_5_8S_RRNA_LSU_RRNA_ | 19 | -2,554 | 0,0005 | Low_in_IC75 |
| GO_CYTOSKELETON_DEPENDENT_CYTOKINESIS | 36 | -2,553 | 0,0005 | Low_in_IC75 |
| GO_ERROR_FREE_TRANSLESION_SYNTHESIS | 19 | -2,549 | 0,0005 | Low_in_IC75 |
| GO_NEGATIVE_REGULATION_OF_PROTEIN_MODIFICATION_PROCESS | 497 | -2,546 | 0,0005 | Low_in_IC75 |
| GO_PIGMENT_BIOSYNTHETIC_PROCESS | 38 | -2,541 | 0,0005 | Low_in_IC75 |
| GO_CARBOHYDRATE_BIOSYNTHETIC_PROCESS | 100 | -2,538 | 0,0005 | Low_in_IC75 |
| GO_NUCLEAR_PORE_ORGANIZATION | 15 | -2,536 | 0,0005 | Low_in_IC75 |
| GO_NUCLEUS_ORGANIZATION | 114 | -2,535 | 0,0005 | Low_in_IC75 |
| GO_REGULATION_OF_CYTOSKELETON_ORGANIZATION | 408 | -2,532 | 0,0005 | Low_in_IC75 |
| GO_ASPARTATE_FAMILY_AMINO_ACID_METABOLIC_PROCESS | 43 | -2,529 | 0,0005 | Low_in_IC75 |
| GO_REGULATION_OF_UBIQUITIN_PROTEIN_LIGASE_ACTIVITY | 17 | -2,517 | 0,0006 | Low_in_IC75 |
| GO_SOMATIC_DIVERSIFICATION_OF_IMMUNE_RECEPTORS | 35 | -2,515 | 0,0006 | Low_in_IC75 |
| GO_DEOXYRIBONUCLEOTIDE_METABOLIC_PROCESS | 29 | -2,512 | 0,0006 | Low_in_IC75 |
| GO_FATTY_ACID_METABOLIC_PROCESS | 210 | -2,509 | 0,0006 | Low_in_IC75 |
| GO_GLOBAL_GENOME_NUCLEOTIDE_EXCISION_REPAIR | 32 | -2,488 | 0,0007 | Low_in_IC75 |
| GO_REGULATION_OF_CANONICAL_WNT_SIGNALING_PATHWAY | 194 | -2,488 | 0,0007 | Low_in_IC75 |
| GO_REGULATION_OF_CELL_CYCLE_G1_S_PHASE_TRANSITION | 136 | -2,482 | 0,0007 | Low_in_IC75 |
| GO_NON_RECOMBINATIONAL_REPAIR | 60 | -2,482 | 0,0007 | Low_in_IC75 |
| GO_PTERIDINE_CONTAINING_COMPOUND_METABOLIC_PROCESS | 28 | -2,474 | 0,0007 | Low_in_IC75 |
| GO_CELLULAR_KETONE_METABOLIC_PROCESS | 57 | -2,473 | 0,0007 | Low_in_IC75 |
| GO_MACROMOLECULE_METHYLATION | 163 | -2,472 | 0,0007 | Low_in_IC75 |
| GO_REGULATION_OF_CELL_CYCLE_CHECKPOINT | 26 | -2,464 | 0,0008 | Low_in_IC75 |
| GO_REGULATION_OF_TELOMERASE_ACTIVITY | 36 | -2,456 | 0,0009 | Low_in_IC75 |
| GO_MONOSACCHARIDE_CATABOLIC_PROCESS | 49 | -2,456 | 0,0009 | Low_in_IC75 |
| GO_REGULATION_OF_CELLULAR_PROTEIN_LOCALIZATION | 458 | -2,434 | 0,001 | Low_in_IC75 |
| GO_RESPONSE_TO_IONIZING_RADIATION | 128 | -2,433 | 0,001 | Low_in_IC75 |
| GO_TRANSCRIPTION_FROM_RNA_POLYMERASE_III_PROMOTER | 40 | -2,431 | 0,001 | Low_in_IC75 |
| GO_REGULATION_OF_CELL_CYCLE_G2_M_PHASE_TRANSITION | 53 | -2,408 | 0,001 | Low_in_IC75 |
| GO_POSITIVE_REGULATION_OF_MITOTIC_CELL_CYCLE | 105 | -2,407 | 0,001 | Low_in_IC75 |
| GO_CELLULAR_AMINO_ACID_CATABOLIC_PROCESS | 84 | -2,405 | 0,001 | Low_in_IC75 |
| GO_NEGATIVE_REGULATION_OF_DNA_METABOLIC_PROCESS | 93 | -2,404 | 0,001 | Low_in_IC75 |
| GO_PYRUVATE_METABOLIC_PROCESS | 50 | -2,403 | 0,001 | Low_in_IC75 |
| GO_TRANSCRIPTION_ELONGATION_FROM_RNA_POLYMERASE_II_PROMOTER | 78 | -2,402 | 0,001 | Low_in_IC75 |
| GO_PROTEIN_EXPORT_FROM_NUCLEUS | 28 | -2,401 | 0,001 | Low_in_IC75 |
| GO_REGULATION_OF_DNA_DEPENDENT_DNA_REPLICATION | 38 | -2,401 | 0,001 | Low_in_IC75 |
| GO_TRANSLATIONAL_INITIATION | 142 | -2,395 | 0,001 | Low_in_IC75 |
| GO_RESPIRATORY_CHAIN_COMPLEX_IV_ASSEMBLY | 17 | -2,389 | 0,001 | Low_in_IC75 |
| GO_POSITIVE_REGULATION_OF_TELOMERE_MAINTENANCE | 41 | -2,389 | 0,001 | Low_in_IC75 |
| GO_POSITIVE_REGULATION_OF_MITOTIC_NUCLEAR_DIVISION | 39 | -2,387 | 0,001 | Low_in_IC75 |
| GO_PYRIMIDINE_CONTAINING_COMPOUND_BIOSYNTHETIC_PROCESS | 35 | -2,386 | 0,001 | Low_in_IC75 |
| GO_NUCLEAR_TRANSCRIBED_MRNA_CATABOLIC_PROCESS_NONSENSE_MEDIATED_DECAY | 113 | -2,38 | 0,001 | Low_in_IC75 |
| GO_DETECTION_OF_STIMULUS | 228 | -2,377 | 0,001 | Low_in_IC75 |
| GO_CHROMOSOME_CONDENSATION | 24 | -2,373 | 0,001 | Low_in_IC75 |
| GO_POSITIVE_REGULATION_OF_CELL_CYCLE_PHASE_TRANSITION | 62 | -2,368 | 0,002 | Low_in_IC75 |
| GO_PIGMENT_METABOLIC_PROCESS | 44 | -2,359 | 0,002 | Low_in_IC75 |
| GO_REGULATION_OF_WNT_SIGNALING_PATHWAY | 247 | -2,358 | 0,002 | Low_in_IC75 |
| GO_ERROR_PRONE_TRANSLESION_SYNTHESIS | 17 | -2,353 | 0,002 | Low_in_IC75 |
| GO_MATURATION_OF_5_8S_RRNA | 27 | -2,351 | 0,002 | Low_in_IC75 |
| GO_DEOXYRIBOSE_PHOSPHATE_CATABOLIC_PROCESS | 18 | -2,351 | 0,002 | Low_in_IC75 |
| GO_CYTOPLASMIC_TRANSLATION | 38 | -2,336 | 0,002 | Low_in_IC75 |
| GO_VIRAL_LIFE_CYCLE | 258 | -2,335 | 0,002 | Low_in_IC75 |
| GO_FATTY_ACID_CATABOLIC_PROCESS | 57 | -2,331 | 0,002 | Low_in_IC75 |
| GO_INTERSTRAND_CROSS_LINK_REPAIR | 36 | -2,33 | 0,002 | Low_in_IC75 |
| GO_REGULATION_OF_CENTROSOME_DUPLICATION | 28 | -2,328 | 0,002 | Low_in_IC75 |
| GO_NEGATIVE_REGULATION_OF_CELLULAR_PROTEIN_CATABOLIC_PROCESS | 53 | -2,324 | 0,002 | Low_in_IC75 |
| GO_DNA_TEMPLATED_TRANSCRIPTION_INITIATION | 169 | -2,323 | 0,002 | Low_in_IC75 |
| GO_POSITIVE_REGULATION_OF_ORGANELLE_ORGANIZATION | 476 | -2,314 | 0,002 | Low_in_IC75 |
| GO_CENTROSOME_CYCLE | 39 | -2,301 | 0,002 | Low_in_IC75 |
| GO_BRANCHED_CHAIN_AMINO_ACID_METABOLIC_PROCESS | 21 | -2,297 | 0,002 | Low_in_IC75 |
| GO_CLEAVAGE_INVOLVED_IN_RRNA_PROCESSING | 18 | -2,294 | 0,002 | Low_in_IC75 |
| GO_GLYCERALDEHYDE_3_PHOSPHATE_METABOLIC_PROCESS | 18 | -2,291 | 0,003 | Low_in_IC75 |
| GO_NUCLEOTIDE_EXCISION_REPAIR_PREINCISION_COMPLEX_ASSEMBLY | 29 | -2,29 | 0,003 | Low_in_IC75 |
| GO_TELOMERE_MAINTENANCE_VIA_TELOMERE_LENGTHENING | 22 | -2,289 | 0,003 | Low_in_IC75 |
| GO_PYRIMIDINE_NUCLEOTIDE_METABOLIC_PROCESS | 39 | -2,289 | 0,003 | Low_in_IC75 |
| GO_SOMATIC_CELL_DNA_RECOMBINATION | 29 | -2,282 | 0,003 | Low_in_IC75 |
| GO_PYRIMIDINE_NUCLEOSIDE_METABOLIC_PROCESS | 39 | -2,278 | 0,003 | Low_in_IC75 |
| GO_NUCLEOTIDE_EXCISION_REPAIR_DNA_DAMAGE_RECOGNITION | 23 | -2,278 | 0,003 | Low_in_IC75 |
| GO_FOLIC_ACID_CONTAINING_COMPOUND_METABOLIC_PROCESS | 22 | -2,266 | 0,003 | Low_in_IC75 |
| GO_REGULATION_OF_MRNA_METABOLIC_PROCESS | 89 | -2,263 | 0,003 | Low_in_IC75 |
| GO_COVALENT_CHROMATIN_MODIFICATION | 287 | -2,262 | 0,003 | Low_in_IC75 |
| GO_PORE_COMPLEX_ASSEMBLY | 15 | -2,261 | 0,003 | Low_in_IC75 |
| GO_SOMATIC_DIVERSIFICATION_OF_IMMUNOGLOBULINS | 23 | -2,26 | 0,003 | Low_in_IC75 |
| GO_PYRIMIDINE_NUCLEOTIDE_BIOSYNTHETIC_PROCESS | 26 | -2,254 | 0,003 | Low_in_IC75 |
| GO_POSITIVE_REGULATION_OF_CELLULAR_AMIDE_METABOLIC_PROCESS | 96 | -2,246 | 0,003 | Low_in_IC75 |
| GO_MITOTIC_CYTOKINESIS | 28 | -2,244 | 0,003 | Low_in_IC75 |
| GO_FATTY_ACYL_COA_METABOLIC_PROCESS | 40 | -2,242 | 0,003 | Low_in_IC75 |
| GO_TELOMERE_MAINTENANCE_VIA_TELOMERASE | 15 | -2,241 | 0,004 | Low_in_IC75 |
| GO_RNA_DEPENDENT_DNA_BIOSYNTHETIC_PROCESS | 20 | -2,239 | 0,004 | Low_in_IC75 |
| GO_TETRAPYRROLE_BIOSYNTHETIC_PROCESS | 24 | -2,236 | 0,004 | Low_in_IC75 |
| GO_MONOCARBOXYLIC_ACID_BIOSYNTHETIC_PROCESS | 126 | -2,232 | 0,004 | Low_in_IC75 |
| GO_MONOCARBOXYLIC_ACID_CATABOLIC_PROCESS | 74 | -2,226 | 0,004 | Low_in_IC75 |
| GO_DNA_LIGATION | 16 | -2,223 | 0,004 | Low_in_IC75 |
| GO_POSITIVE_REGULATION_OF_CELLULAR_PROTEIN_LOCALIZATION | 295 | -2,215 | 0,004 | Low_in_IC75 |
| GO_CARBOHYDRATE_CATABOLIC_PROCESS | 93 | -2,214 | 0,004 | Low_in_IC75 |
| GO_CELLULAR_COMPONENT_ASSEMBLY_INVOLVED_IN_MORPHOGENESIS | 171 | -2,206 | 0,004 | Low_in_IC75 |
| GO_REGULATION_OF_TRANSCRIPTION_FROM_RNA_POLYMERASE_I_PROMOTER | 21 | -2,197 | 0,005 | Low_in_IC75 |
| GO_MONOSACCHARIDE_METABOLIC_PROCESS | 144 | -2,193 | 0,005 | Low_in_IC75 |
| GO_PYRIMIDINE_CONTAINING_COMPOUND_METABOLIC_PROCESS | 59 | -2,185 | 0,005 | Low_in_IC75 |
| GO_FC_RECEPTOR_SIGNALING_PATHWAY | 161 | -2,18 | 0,005 | Low_in_IC75 |
| GO_REGULATION_OF_TRANSLATIONAL_INITIATION | 73 | -2,179 | 0,005 | Low_in_IC75 |
| GO_POSITIVE_REGULATION_OF_DNA_REPLICATION | 69 | -2,179 | 0,005 | Low_in_IC75 |
| GO_REGULATION_OF_DOUBLE_STRAND_BREAK_REPAIR | 35 | -2,178 | 0,005 | Low_in_IC75 |
| GO_RNA_SECONDARY_STRUCTURE_UNWINDING | 36 | -2,174 | 0,005 | Low_in_IC75 |
| GO_MITOTIC_SPINDLE_ASSEMBLY | 36 | -2,172 | 0,005 | Low_in_IC75 |
| GO_SOMATIC_RECOMBINATION_OF_IMMUNOGLOBULIN_GENE_SEGMENTS | 18 | -2,168 | 0,005 | Low_in_IC75 |
| GO_FEMALE_MEIOTIC_DIVISION | 20 | -2,164 | 0,006 | Low_in_IC75 |
| GO_RESPONSE_TO_UV | 117 | -2,159 | 0,006 | Low_in_IC75 |
| GO_PROTEIN_TRANSPORT_ALONG_MICROTUBULE | 23 | -2,158 | 0,006 | Low_in_IC75 |
| GO_LIPID_BIOSYNTHETIC_PROCESS | 403 | -2,151 | 0,006 | Low_in_IC75 |
| GO_GLUCOSE_METABOLIC_PROCESS | 87 | -2,15 | 0,006 | Low_in_IC75 |
| GO_LIPOPROTEIN_BIOSYNTHETIC_PROCESS | 73 | -2,149 | 0,006 | Low_in_IC75 |
| GO_ANATOMICAL_STRUCTURE_HOMEOSTASIS | 208 | -2,147 | 0,006 | Low_in_IC75 |
| GO_CHROMATIN_DISASSEMBLY | 15 | -2,145 | 0,006 | Low_in_IC75 |
| GO_TOXIN_TRANSPORT | 32 | -2,141 | 0,006 | Low_in_IC75 |
| GO_NUCLEOTIDE_EXCISION_REPAIR_PREINCISION_COMPLEX_STABILIZATION | 21 | -2,129 | 0,007 | Low_in_IC75 |
| GO_TONGUE_DEVELOPMENT | 16 | -2,126 | 0,007 | Low_in_IC75 |
| GO_REGULATION_OF_CHOLESTEROL_METABOLIC_PROCESS | 16 | -2,125 | 0,007 | Low_in_IC75 |
| GO_ANTEROGRADE_AXONAL_TRANSPORT | 22 | -2,121 | 0,007 | Low_in_IC75 |
| GO_LIPOPROTEIN_METABOLIC_PROCESS | 99 | -2,118 | 0,007 | Low_in_IC75 |
| GO_PTERIDINE_CONTAINING_COMPOUND_BIOSYNTHETIC_PROCESS | 17 | -2,115 | 0,007 | Low_in_IC75 |
| GO_NEGATIVE_REGULATION_OF_DNA_REPLICATION | 46 | -2,115 | 0,007 | Low_in_IC75 |
| GO_OSTEOBLAST_DIFFERENTIATION | 107 | -2,106 | 0,008 | Low_in_IC75 |
| GO_SPLICEOSOMAL_COMPLEX_ASSEMBLY | 37 | -2,104 | 0,008 | Low_in_IC75 |
| GO_POSITIVE_REGULATION_OF_TYPE_I_INTERFERON_PRODUCTION | 63 | -2,096 | 0,008 | Low_in_IC75 |
| GO_TETRAHYDROFOLATE_METABOLIC_PROCESS | 16 | -2,093 | 0,008 | Low_in_IC75 |
| GO_NEGATIVE_REGULATION_OF_CELL_CYCLE_G1_S_PHASE_TRANSITION | 91 | -2,09 | 0,008 | Low_in_IC75 |
| GO_PREASSEMBLY_OF_GPI_ANCHOR_IN_ER_MEMBRANE | 15 | -2,088 | 0,009 | Low_in_IC75 |
| GO_POSITIVE_REGULATION_OF_RESPONSE_TO_DNA_DAMAGE_STIMULUS | 58 | -2,084 | 0,009 | Low_in_IC75 |
| GO_HEME_BIOSYNTHETIC_PROCESS | 18 | -2,078 | 0,009 | Low_in_IC75 |
| GO_NUCLEOSIDE_DIPHOSPHATE_METABOLIC_PROCESS | 66 | -2,077 | 0,009 | Low_in_IC75 |
| GO_CHROMOSOME_ORGANIZATION_INVOLVED_IN_MEIOTIC_CELL_CYCLE | 26 | -2,076 | 0,009 | Low_in_IC75 |
| GO_ACETYL_COA_METABOLIC_PROCESS | 23 | -2,066 | 0,01 | Low_in_IC75 |
| GO_REGULATION_OF_CYCLIN_DEPENDENT_PROTEIN_KINASE_ACTIVITY | 86 | -2,061 | 0,01 | Low_in_IC75 |
| GO_RNA_PHOSPHODIESTER_BOND_HYDROLYSIS_EXONUCLEOLYTIC | 35 | -2,057 | 0,01 | Low_in_IC75 |
| GO_RECIPROCAL_DNA_RECOMBINATION | 30 | -2,057 | 0,01 | Low_in_IC75 |
| GO_RESPONSE_TO_TUMOR_NECROSIS_FACTOR | 178 | -2,054 | 0,01 | Low_in_IC75 |
| GO_REGULATION_OF_MESENCHYMAL_CELL_PROLIFERATION | 24 | -2,053 | 0,01 | Low_in_IC75 |
| GO_MEMBRANE_LIPID_METABOLIC_PROCESS | 137 | -2,053 | 0,01 | Low_in_IC75 |
| GO_DNA_DEPENDENT_DNA_REPLICATION_MAINTENANCE_OF_FIDELITY | 19 | -2,05 | 0,01 | Low_in_IC75 |
| GO_POSITIVE_REGULATION_OF_TELOMERASE_ACTIVITY | 24 | -2,048 | 0,01 | Low_in_IC75 |
| GO_PORPHYRIN_CONTAINING_COMPOUND_METABOLIC_PROCESS | 28 | -2,041 | 0,01 | Low_in_IC75 |
| GO_POSITIVE_REGULATION_OF_NUCLEAR_DIVISION | 47 | -2,04 | 0,01 | Low_in_IC75 |
| GO_PROTEIN_HOMOTETRAMERIZATION | 44 | -2,033 | 0,01 | Low_in_IC75 |
| GO_MACROMITOPHAGY | 94 | -2,033 | 0,01 | Low_in_IC75 |
| GO_NEGATIVE_REGULATION_OF_DNA_BIOSYNTHETIC_PROCESS | 23 | -2,028 | 0,01 | Low_in_IC75 |
| GO_REGULATION_OF_RESPONSE_TO_DNA_DAMAGE_STIMULUS | 127 | -2,023 | 0,01 | Low_in_IC75 |
| GO_RIBOSOMAL_SMALL_SUBUNIT_ASSEMBLY | 17 | -2,019 | 0,01 | Low_in_IC75 |
| GO_REGULATION_OF_GENE_EXPRESSION_EPIGENETIC | 191 | -2,012 | 0,01 | Low_in_IC75 |
| GO_ESTABLISHMENT_OF_LOCALIZATION_BY_MOVEMENT_ALONG_MICROTUBULE | 86 | -2,012 | 0,01 | Low_in_IC75 |
| GO_PEROXISOME_ORGANIZATION | 27 | -2,01 | 0,01 | Low_in_IC75 |
| GO_BINDING_OF_SPERM_TO_ZONA_PELLUCIDA | 20 | -2,009 | 0,01 | Low_in_IC75 |
| GO_MITOCHONDRIAL_GENOME_MAINTENANCE | 23 | -2,006 | 0,01 | Low_in_IC75 |
| GO_POSITIVE_REGULATION_OF_PROTEIN_LOCALIZATION_TO_NUCLEUS | 100 | -2,006 | 0,01 | Low_in_IC75 |
| GO_ALPHA_AMINO_ACID_CATABOLIC_PROCESS | 68 | -2,005 | 0,01 | Low_in_IC75 |
| GO_REGULATION_OF_MITOCHONDRION_ORGANIZATION | 190 | -2,001 | 0,01 | Low_in_IC75 |
| GO_ATP_GENERATION_FROM_ADP | 31 | -1,997 | 0,01 | Low_in_IC75 |
| GO_G2_DNA_DAMAGE_CHECKPOINT | 29 | -1,994 | 0,01 | Low_in_IC75 |
| GO_RESPONSE_TO_RADIATION | 326 | -1,993 | 0,01 | Low_in_IC75 |
| GO_GLYOXYLATE_METABOLIC_PROCESS | 20 | -1,99 | 0,01 | Low_in_IC75 |
| GO_TRANSCRIPTION_INITIATION_FROM_RNA_POLYMERASE_II_PROMOTER | 124 | -1,984 | 0,02 | Low_in_IC75 |
| GO_HEXOSE_METABOLIC_PROCESS | 118 | -1,983 | 0,02 | Low_in_IC75 |
| GO_PROTEIN_O_LINKED_MANNOSYLATION | 15 | -1,98 | 0,02 | Low_in_IC75 |
| GO_ESTABLISHMENT_OF_MITOTIC_SPINDLE_LOCALIZATION | 21 | -1,977 | 0,02 | Low_in_IC75 |
| GO_REGULATION_OF_TRANSLATIONAL_FIDELITY | 15 | -1,975 | 0,02 | Low_in_IC75 |
| GO_NUCLEOTIDE_EXCISION_REPAIR_DNA_DUPLEX_UNWINDING | 22 | -1,973 | 0,02 | Low_in_IC75 |
| GO_CYTOSKELETON_DEPENDENT_INTRACELLULAR_TRANSPORT | 101 | -1,965 | 0,02 | Low_in_IC75 |
| GO_REGULATION_OF_HISTONE_METHYLATION | 42 | -1,961 | 0,02 | Low_in_IC75 |
| GO_REGULATION_OF_MRNA_SPLICING_VIA_SPLICEOSOME | 38 | -1,961 | 0,02 | Low_in_IC75 |
| GO_REGULATION_OF_GLUCOSE_TRANSPORT | 80 | -1,96 | 0,02 | Low_in_IC75 |
| GO_KETONE_BIOSYNTHETIC_PROCESS | 20 | -1,959 | 0,02 | Low_in_IC75 |
| GO_POSITIVE_REGULATION_OF_MITOCHONDRION_ORGANIZATION | 144 | -1,958 | 0,02 | Low_in_IC75 |
| GO_CELLULAR_METABOLIC_COMPOUND_SALVAGE | 31 | -1,957 | 0,02 | Low_in_IC75 |
| GO_HOMOLOGOUS_CHROMOSOME_SEGREGATION | 22 | -1,953 | 0,02 | Low_in_IC75 |
| GO_MONOSACCHARIDE_BIOSYNTHETIC_PROCESS | 46 | -1,947 | 0,02 | Low_in_IC75 |
| GO_METHIONINE_METABOLIC_PROCESS | 15 | -1,946 | 0,02 | Low_in_IC75 |
| GO_POSITIVE_REGULATION_OF_VIRAL_TRANSCRIPTION | 37 | -1,943 | 0,02 | Low_in_IC75 |
| GO_PROTEIN_COMPLEX_LOCALIZATION | 44 | -1,941 | 0,02 | Low_in_IC75 |
| GO_NUCLEOTIDE_PHOSPHORYLATION | 46 | -1,939 | 0,02 | Low_in_IC75 |
| GO_MANNOSYLATION | 31 | -1,934 | 0,02 | Low_in_IC75 |
| GO_PROTEIN_TETRAMERIZATION | 105 | -1,933 | 0,02 | Low_in_IC75 |
| GO_POSITIVE_REGULATION_OF_DNA_BINDING | 36 | -1,929 | 0,02 | Low_in_IC75 |
| GO_RESPONSE_TO_INTERFERON_BETA | 19 | -1,926 | 0,02 | Low_in_IC75 |
| GO_GUANOSINE_CONTAINING_COMPOUND_METABOLIC_PROCESS | 37 | -1,923 | 0,02 | Low_in_IC75 |
| GO_REGULATION_OF_SISTER_CHROMATID_COHESION | 16 | -1,921 | 0,02 | Low_in_IC75 |
| GO_REGULATION_OF_HISTONE_H3_K4_METHYLATION | 22 | -1,918 | 0,02 | Low_in_IC75 |
| GO_AXIS_ELONGATION | 20 | -1,918 | 0,02 | Low_in_IC75 |
| GO_ACTIVATION_OF_INNATE_IMMUNE_RESPONSE | 165 | -1,916 | 0,02 | Low_in_IC75 |
| GO_RRNA_TRANSCRIPTION | 18 | -1,911 | 0,02 | Low_in_IC75 |
| GO__DE_NOVO_PROTEIN_FOLDING | 17 | -1,907 | 0,02 | Low_in_IC75 |
| GO_NEGATIVE_REGULATION_OF_EPITHELIAL_CELL_PROLIFERATION | 83 | -1,905 | 0,02 | Low_in_IC75 |
| GO_REGULATION_OF_TRANSLATIONAL_ELONGATION | 23 | -1,903 | 0,02 | Low_in_IC75 |
| GO_PROTEIN_MATURATION | 205 | -1,903 | 0,02 | Low_in_IC75 |
| GO_REGULATION_OF_DNA_BINDING | 76 | -1,897 | 0,02 | Low_in_IC75 |
| GO_RIBONUCLEOSIDE_DIPHOSPHATE_METABOLIC_PROCESS | 53 | -1,893 | 0,02 | Low_in_IC75 |
| GO_PROTEIN_REFOLDING | 20 | -1,891 | 0,02 | Low_in_IC75 |
| GO_POSITIVE_REGULATION_OF_CHROMATIN_MODIFICATION | 74 | -1,888 | 0,02 | Low_in_IC75 |
| GO_GLUCOSE_6_PHOSPHATE_METABOLIC_PROCESS | 18 | -1,887 | 0,02 | Low_in_IC75 |
| GO_SIGNAL_TRANSDUCTION_BY_P53_CLASS_MEDIATOR | 114 | -1,885 | 0,02 | Low_in_IC75 |
| GO_REGULATION_OF_PROTEIN_STABILITY | 191 | -1,885 | 0,02 | Low_in_IC75 |
| GO_POSITIVE_REGULATION_OF_PEPTIDASE_ACTIVITY | 130 | -1,884 | 0,02 | Low_in_IC75 |
| GO_MULTICELLULAR_ORGANISM_AGING | 20 | -1,882 | 0,02 | Low_in_IC75 |
| GO_METALLO_SULFUR_CLUSTER_ASSEMBLY | 16 | -1,874 | 0,03 | Low_in_IC75 |
| GO_GLUTAMINE_FAMILY_AMINO_ACID_METABOLIC_PROCESS | 53 | -1,871 | 0,03 | Low_in_IC75 |
| GO_NADP_METABOLIC_PROCESS | 23 | -1,867 | 0,03 | Low_in_IC75 |
| GO_ADP_METABOLIC_PROCESS | 38 | -1,864 | 0,03 | Low_in_IC75 |
| GO_EPIDERMIS_MORPHOGENESIS | 20 | -1,861 | 0,03 | Low_in_IC75 |
| GO_NEGATIVE_REGULATION_OF_CELLULAR_AMIDE_METABOLIC_PROCESS | 110 | -1,861 | 0,03 | Low_in_IC75 |
| GO_G1_DNA_DAMAGE_CHECKPOINT | 68 | -1,858 | 0,03 | Low_in_IC75 |
| GO_RESPONSE_TO_INTERFERON_ALPHA | 17 | -1,852 | 0,03 | Low_in_IC75 |
| GO_SPINDLE_LOCALIZATION | 35 | -1,85 | 0,03 | Low_in_IC75 |
| GO_NADH_METABOLIC_PROCESS | 29 | -1,85 | 0,03 | Low_in_IC75 |
| GO_SMOOTHENED_SIGNALING_PATHWAY | 47 | -1,846 | 0,03 | Low_in_IC75 |
| GO_ESTABLISHMENT_OF_MITOTIC_SPINDLE_ORIENTATION | 17 | -1,846 | 0,03 | Low_in_IC75 |
| GO_HISTONE_PHOSPHORYLATION | 22 | -1,845 | 0,03 | Low_in_IC75 |
| GO_NEGATIVE_REGULATION_OF_PROTEIN_BINDING | 65 | -1,837 | 0,03 | Low_in_IC75 |
| GO_PALLIUM_DEVELOPMENT | 112 | -1,829 | 0,03 | Low_in_IC75 |
| GO_SIGNAL_TRANSDUCTION_IN_RESPONSE_TO_DNA_DAMAGE | 88 | -1,828 | 0,03 | Low_in_IC75 |
| GO_REGULATION_OF_TYPE_I_INTERFERON_PRODUCTION | 100 | -1,824 | 0,03 | Low_in_IC75 |
| GO_POSITIVE_REGULATION_OF_TRANSCRIPTION_INITIATION_FROM_RNA_POLYMERASE_II_PROMOTER | 16 | -1,82 | 0,03 | Low_in_IC75 |
| GO_ORGANELLE_DISASSEMBLY | 137 | -1,819 | 0,03 | Low_in_IC75 |
| GO_MEMBRANE_LIPID_BIOSYNTHETIC_PROCESS | 85 | -1,816 | 0,03 | Low_in_IC75 |
| GO_POSITIVE_REGULATION_OF_INNATE_IMMUNE_RESPONSE | 191 | -1,815 | 0,03 | Low_in_IC75 |
| GO_POSITIVE_REGULATION_OF_DNA_TEMPLATED_TRANSCRIPTION_INITIATION | 23 | -1,811 | 0,04 | Low_in_IC75 |
| GO_NCRNA_CATABOLIC_PROCESS | 18 | -1,808 | 0,04 | Low_in_IC75 |
| GO_SULFUR_AMINO_ACID_BIOSYNTHETIC_PROCESS | 16 | -1,804 | 0,04 | Low_in_IC75 |
| GO_PROTEIN_OLIGOMERIZATION | 329 | -1,801 | 0,04 | Low_in_IC75 |
| GO_WATER_SOLUBLE_VITAMIN_METABOLIC_PROCESS | 66 | -1,801 | 0,04 | Low_in_IC75 |
| GO_NUCLEOSIDE_PHOSPHATE_CATABOLIC_PROCESS | 48 | -1,798 | 0,04 | Low_in_IC75 |
| GO_POSITIVE_REGULATION_OF_VIRAL_PROCESS | 86 | -1,797 | 0,04 | Low_in_IC75 |
| GO_HEPARAN_SULFATE_PROTEOGLYCAN_METABOLIC_PROCESS | 19 | -1,797 | 0,04 | Low_in_IC75 |
| GO_RIBOSOMAL_LARGE_SUBUNIT_ASSEMBLY | 22 | -1,796 | 0,04 | Low_in_IC75 |
| GO_PURINE_CONTAINING_COMPOUND_CATABOLIC_PROCESS | 35 | -1,796 | 0,04 | Low_in_IC75 |
| GO_AROMATIC_AMINO_ACID_FAMILY_METABOLIC_PROCESS | 16 | -1,789 | 0,04 | Low_in_IC75 |
| GO_TETRAPYRROLE_METABOLIC_PROCESS | 42 | -1,785 | 0,04 | Low_in_IC75 |
| GO_XENOPHAGY | 82 | -1,783 | 0,04 | Low_in_IC75 |
| GO_REGULATION_OF_DNA_TEMPLATED_TRANSCRIPTION_INITIATION | 29 | -1,781 | 0,04 | Low_in_IC75 |
| GO_HEPARAN_SULFATE_PROTEOGLYCAN_BIOSYNTHETIC_PROCESS | 16 | -1,781 | 0,04 | Low_in_IC75 |
| GO_RESPONSE_TO_TOXIC_SUBSTANCE | 178 | -1,776 | 0,04 | Low_in_IC75 |
| GO_PROTEIN_K11_LINKED_UBIQUITINATION | 25 | -1,773 | 0,04 | Low_in_IC75 |
| GO_NEGATIVE_REGULATION_OF_TELOMERE_MAINTENANCE_VIA_TELOMERE_LENGTHENING | 17 | -1,769 | 0,04 | Low_in_IC75 |
| GO_ESTABLISHMENT_OF_SPINDLE_ORIENTATION | 23 | -1,769 | 0,04 | Low_in_IC75 |
| GO_COENZYME_A_METABOLIC_PROCESS | 15 | -1,766 | 0,04 | Low_in_IC75 |
| GO_VITAMIN_METABOLIC_PROCESS | 86 | -1,761 | 0,04 | Low_in_IC75 |
| GO_IMMUNOGLOBULIN_PRODUCTION | 30 | -1,761 | 0,04 | Low_in_IC75 |
| GO_REGULATION_OF_TRANSCRIPTION_INITIATION_FROM_RNA_POLYMERASE_II_PROMOTER | 21 | -1,76 | 0,04 | Low_in_IC75 |
| GO_HISTONE_MRNA_METABOLIC_PROCESS | 26 | -1,759 | 0,04 | Low_in_IC75 |
| GO_STEROL_METABOLIC_PROCESS | 92 | -1,754 | 0,05 | Low_in_IC75 |
| GO_2_OXOGLUTARATE_METABOLIC_PROCESS | 18 | -1,753 | 0,05 | Low_in_IC75 |
| GO_HEXOSE_CATABOLIC_PROCESS | 39 | -1,753 | 0,05 | Low_in_IC75 |
| GO_REGULATION_OF_PROTEIN_LOCALIZATION_TO_NUCLEUS | 173 | -1,749 | 0,05 | Low_in_IC75 |
| GO_NEGATIVE_REGULATION_OF_TELOMERE_MAINTENANCE | 26 | -1,749 | 0,05 | Low_in_IC75 |
| GO_NAD_METABOLIC_PROCESS | 42 | -1,749 | 0,05 | Low_in_IC75 |
| GO_RRNA_CATABOLIC_PROCESS | 15 | -1,748 | 0,05 | Low_in_IC75 |
| GO_CEREBRAL_CORTEX_DEVELOPMENT | 82 | -1,748 | 0,05 | Low_in_IC75 |
| GO_MITOCHONDRIAL_FUSION | 19 | -1,742 | 0,05 | Low_in_IC75 |
| GO_STEROID_METABOLIC_PROCESS | 172 | -1,739 | 0,05 | Low_in_IC75 |
| GO_POSITIVE_REGULATION_OF_MRNA_PROCESSING | 28 | -1,736 | 0,05 | Low_in_IC75 |
| GO_FATTY_ACID_BIOSYNTHETIC_PROCESS | 82 | -1,736 | 0,05 | Low_in_IC75 |
| GO_ALCOHOL_METABOLIC_PROCESS | 258 | -1,736 | 0,05 | Low_in_IC75 |
| GO_CENTROSOME_DUPLICATION | 28 | -1,735 | 0,05 | Low_in_IC75 |
| GO_ALDITOL_METABOLIC_PROCESS | 16 | -1,733 | 0,05 | Low_in_IC75 |
| GO_REGULATION_OF_CHROMATIN_ORGANIZATION | 126 | -1,731 | 0,05 | Low_in_IC75 |
| GO_REGULATION_OF_FIBROBLAST_GROWTH_FACTOR_RECEPTOR_SIGNALING_PATHWAY | 19 | -1,731 | 0,05 | Low_in_IC75 |
| GO_PROTEIN_MANNOSYLATION | 20 | -1,731 | 0,05 | Low_in_IC75 |
