# Supplementary material for: Induction of ER and mitochondrial stress by the alkylphosphocholine erufosine in oral squamous cell carcinoma cells
Source: Cell Death Dis. 2018 Feb 20;9(3):296. doi: 10.1038/s41419-018-0342-2 (PMC5833417; doi:10.1038/s41419-018-0342-2)
Supplement: Supplementary file 13 — Supplementary Table 5a [file 41419_2018_342_MOESM13_ESM.docx]

Table S5a: Differential regulation of autophagic genes upon IC50 exposure of erufosine in HN-5 cells

| **Symbol** | **Definition** | **Log Fold Change** | **Average Expression** | **t-statistics** | **P.Value** | **adj.P.Val** |
| --- | --- | --- | --- | --- | --- | --- |
| ULK1 | Homo sapiens unc-51-like kinase 1 (C. elegans) (ULK1), mRNA. | 1,87767 | 10,44580 | 5,18343 | 0,000476 | 0,012601 |
| ULK1 | Homo sapiens unc-51-like kinase 1 (C. elegans) (ULK1), mRNA. | 1,79931 | 10,15535 | 4,89848 | 0,000714 | 0,015627 |
| MTSS1 | Homo sapiens metastasis suppressor 1 (MTSS1), mRNA. | 1,63410 | 9,94104 | 7,42024 | 0,000029 | 0,003704 |
| WIPI1 | Homo sapiens WD repeat domain, phosphoinositide interacting 1 (WIPI1), mRNA. | 1,53398 | 8,74354 | 8,90456 | 0,000006 | 0,001981 |
| ITGB4 | Homo sapiens integrin, beta 4 (ITGB4), transcript variant 2, mRNA. | 1,34705 | 11,22811 | 8,39012 | 0,000010 | 0,002458 |
| NPC1 | Homo sapiens Niemann-Pick disease, type C1 (NPC1), mRNA. | 1,33397 | 9,25546 | 7,27672 | 0,000034 | 0,004015 |
| ITGB4 | Homo sapiens integrin, beta 4 (ITGB4), transcript variant 3, mRNA. | 1,23483 | 10,26505 | 7,92601 | 0,000017 | 0,003155 |
| MTSS1 | Homo sapiens metastasis suppressor 1 (MTSS1), mRNA. | 1,13553 | 8,39421 | 6,57087 | 0,000078 | 0,005518 |
| XBP1 | Homo sapiens X-box binding protein 1 (XBP1), transcript variant 1, mRNA. | 1,05119 | 10,85507 | 6,71209 | 0,000066 | 0,004935 |
| MAP1LC3B | Homo sapiens microtubule-associated protein 1 light chain 3 beta (MAP1LC3B), mRNA. | 1,03284 | 9,02125 | 7,43500 | 0,000029 | 0,003681 |
| XBP1 | Homo sapiens X-box binding protein 1 (XBP1), transcript variant 2, mRNA. | 1,02050 | 10,79362 | 5,35889 | 0,000373 | 0,011228 |
| LARP1B | Homo sapiens La ribonucleoprotein domain family, member 1B (LARP1B), transcript variant 3, mRNA. | 0,98654 | 9,12820 | 7,70640 | 0,000021 | 0,003320 |
| RRAGC | Homo sapiens Ras-related GTP binding C (RRAGC), mRNA. | 0,91023 | 8,97200 | 4,97056 | 0,000643 | 0,014727 |
| VPS28 | Homo sapiens vacuolar protein sorting 28 homolog (S. cerevisiae) (VPS28), transcript variant 1, mRNA. | 0,90940 | 9,76918 | 4,07809 | 0,002442 | 0,029975 |
| SH3GLB1 | Homo sapiens SH3-domain GRB2-like endophilin B1 (SH3GLB1), mRNA. | 0,90254 | 10,25645 | 8,17201 | 0,000013 | 0,002718 |
| HK2 | Homo sapiens hexokinase 2 (HK2), mRNA. | 0,88618 | 8,71941 | 4,22716 | 0,001939 | 0,026308 |
| DAP | Homo sapiens death-associated protein (DAP), mRNA. | 0,83836 | 9,19919 | 4,63599 | 0,001047 | 0,018813 |
| VPS37B | Homo sapiens vacuolar protein sorting 37 homolog B (S. cerevisiae) (VPS37B), mRNA. | 0,77615 | 9,62231 | 3,79227 | 0,003831 | 0,038970 |
| SQSTM1 | Homo sapiens sequestosome 1 (SQSTM1), mRNA. | 0,69621 | 12,84273 | 5,01068 | 0,000608 | 0,014236 |
| WDR45 | Homo sapiens WD repeat domain 45 (WDR45), transcript variant 1, mRNA. | 0,68048 | 8,09291 | 5,30754 | 0,000401 | 0,011532 |
| FNBP1L | Homo sapiens formin binding protein 1-like (FNBP1L), transcript variant 1, mRNA. | 0,64832 | 8,46408 | 4,61514 | 0,001080 | 0,019212 |
| PRKAG2 | Homo sapiens protein kinase, AMP-activated, gamma 2 non-catalytic subunit (PRKAG2), transcript variant c, mRNA. | 0,62974 | 8,51105 | 3,58494 | 0,005346 | 0,047748 |
| SLC35C1 | Homo sapiens solute carrier family 35, member C1 (SLC35C1), mRNA. | 0,55321 | 8,58541 | 6,47749 | 0,000087 | 0,005878 |
| STAM | Homo sapiens signal transducing adaptor molecule (SH3 domain and ITAM motif) 1 (STAM), mRNA. | 0,53918 | 8,40637 | 4,65973 | 0,001011 | 0,018360 |
| NRBF2 | Homo sapiens nuclear receptor binding factor 2 (NRBF2), mRNA. | 0,53105 | 8,17743 | 4,38682 | 0,001520 | 0,023078 |
| USP13 | Homo sapiens ubiquitin specific peptidase 13 (isopeptidase T-3) (USP13), mRNA. | -0,53380 | 7,96105 | -4,63255 | 0,001052 | 0,018869 |
| CHAF1B | Homo sapiens chromatin assembly factor 1, subunit B (p60) (CHAF1B), mRNA. | -0,54700 | 7,82196 | -6,21213 | 0,000121 | 0,006828 |
| TMEM39B | Homo sapiens transmembrane protein 39B (TMEM39B), mRNA. | -0,58494 | 8,55520 | -5,84403 | 0,000195 | 0,008123 |
| BLOC1S1 | Homo sapiens biogenesis of lysosome-related organelles complex-1, subunit 1 (BLOC1S1), mRNA. | -0,66958 | 8,33632 | -4,14718 | 0,002193 | 0,028036 |
| TOMM5 | Homo sapiens translocase of outer mitochondrial membrane 5 homolog (yeast) (TOMM5), nuclear gene encoding mitochondrial protein, transcript variant 1, mRNA. | -0,67519 | 11,04395 | -4,45021 | 0,001381 | 0,021836 |
| FANCL | Homo sapiens Fanconi anemia, complementation group L (FANCL), mRNA. | -0,70689 | 7,91916 | -5,33233 | 0,000387 | 0,011322 |
| C7orf59 | Homo sapiens chromosome 7 open reading frame 59 (C7orf59), mRNA. | -0,71218 | 10,38013 | -6,32662 | 0,000105 | 0,006325 |
| TOMM22 | Homo sapiens translocase of outer mitochondrial membrane 22 homolog (yeast) (TOMM22), nuclear gene encoding mitochondrial protein, mRNA. | -0,73670 | 9,32872 | -6,20691 | 0,000122 | 0,006845 |
| RFWD3 | Homo sapiens ring finger and WD repeat domain 3 (RFWD3), mRNA. | -0,81898 | 8,79470 | -6,01411 | 0,000156 | 0,007378 |
| ATP1B1 | Homo sapiens ATPase, Na+/K+ transporting, beta 1 polypeptide (ATP1B1), transcript variant 1, mRNA. | -0,85388 | 9,50575 | -4,98139 | 0,000634 | 0,014573 |
| SNRPF | Homo sapiens small nuclear ribonucleoprotein polypeptide F (SNRPF), mRNA. | -0,89040 | 11,45882 | -3,70242 | 0,004423 | 0,042522 |
| LSM4 | Homo sapiens LSM4 homolog, U6 small nuclear RNA associated (S. cerevisiae) (LSM4), mRNA. | -0,93970 | 10,59295 | -3,71244 | 0,004353 | 0,042098 |
| ATP1B1 | Homo sapiens ATPase, Na+/K+ transporting, beta 1 polypeptide (ATP1B1), transcript variant 2, mRNA. | -0,96706 | 10,42939 | -3,94376 | 0,003013 | 0,033766 |
| PNPO | Homo sapiens pyridoxamine 5'-phosphate oxidase (PNPO), mRNA. | -1,03201 | 9,26012 | -3,72642 | 0,004256 | 0,041641 |
| ATP1B1 | Homo sapiens ATPase, Na+/K+ transporting, beta 1 polypeptide (ATP1B1), transcript variant 1, mRNA. | -1,06454 | 9,53913 | -4,68890 | 0,000968 | 0,017934 |
