# Supplementary material for: Induction of ER and mitochondrial stress by the alkylphosphocholine erufosine in oral squamous cell carcinoma cells
Source: Cell Death Dis. 2018 Feb 20;9(3):296. doi: 10.1038/s41419-018-0342-2 (PMC5833417; doi:10.1038/s41419-018-0342-2)
Supplement: Supplementary file 14 — Supplementary Table 5b [file 41419_2018_342_MOESM14_ESM.docx]

Table S5b: Differential regulation of autophagic genes upon IC75 exposure of erufosine in HN-5 cells

| **Symbol** | **Definition** | **Log Fold Change** | **Average Expression** | **t-statistics** | **P.Value** | **adj.P.Val** |
| --- | --- | --- | --- | --- | --- | --- |
| ULK1 | Homo sapiens unc-51-like kinase 1 (C. elegans) (ULK1), mRNA. | 2,27277 | 10,15535 | 6,18744 | 0,00013 | 0,00250 |
| ULK1 | Homo sapiens unc-51-like kinase 1 (C. elegans) (ULK1), mRNA. | 2,24849 | 10,44580 | 6,20711 | 0,00012 | 0,00247 |
| GABARAPL1 | Homo sapiens GABA(A) receptor-associated protein like 1 (GABARAPL1), mRNA. | 1,77877 | 9,36359 | 6,43441 | 0,00009 | 0,00215 |
| WIPI1 | Homo sapiens WD repeat domain, phosphoinositide interacting 1 (WIPI1), mRNA. | 1,73053 | 8,74354 | 10,04553 | 0,00000 | 0,00044 |
| NPC1 | Homo sapiens Niemann-Pick disease, type C1 (NPC1), mRNA. | 1,72884 | 9,25546 | 9,43076 | 0,00000 | 0,00052 |
| MAP1LC3B | Homo sapiens microtubule-associated protein 1 light chain 3 beta (MAP1LC3B), mRNA. | 1,32937 | 9,02125 | 9,56961 | 0,00000 | 0,00051 |
| LARP1B | Homo sapiens La ribonucleoprotein domain family, member 1B (LARP1B), transcript variant 3, mRNA. | 1,31981 | 9,12820 | 10,30978 | 0,00000 | 0,00041 |
| XBP1 | Homo sapiens X-box binding protein 1 (XBP1), transcript variant 1, mRNA. | 1,23546 | 10,85507 | 7,88873 | 0,00002 | 0,00101 |
| ITGB4 | Homo sapiens integrin, beta 4 (ITGB4), transcript variant 2, mRNA. | 1,21715 | 11,22811 | 7,58107 | 0,00002 | 0,00117 |
| ITGB4 | Homo sapiens integrin, beta 4 (ITGB4), transcript variant 3, mRNA. | 1,21155 | 10,26505 | 7,77655 | 0,00002 | 0,00107 |
| SQSTM1 | Homo sapiens sequestosome 1 (SQSTM1), mRNA. | 1,20357 | 12,84273 | 8,66219 | 0,00001 | 0,00073 |
| MTSS1 | Homo sapiens metastasis suppressor 1 (MTSS1), mRNA. | 1,19627 | 9,94104 | 5,43211 | 0,00034 | 0,00433 |
| WDR45 | Homo sapiens WD repeat domain 45 (WDR45), transcript variant 1, mRNA. | 1,19423 | 8,09291 | 9,31457 | 0,00000 | 0,00054 |
| HGS | Homo sapiens hepatocyte growth factor-regulated tyrosine kinase substrate (HGS), mRNA. | 1,18447 | 10,57936 | 5,44090 | 0,00033 | 0,00431 |
| XBP1 | Homo sapiens X-box binding protein 1 (XBP1), transcript variant 2, mRNA. | 1,12677 | 10,79362 | 5,91694 | 0,00018 | 0,00306 |
| VPS37B | Homo sapiens vacuolar protein sorting 37 homolog B (S. cerevisiae) (VPS37B), mRNA. | 1,04073 | 9,62231 | 5,08506 | 0,00055 | 0,00569 |
| PINK1 | Homo sapiens PTEN induced putative kinase 1 (PINK1), nuclear gene encoding mitochondrial protein, mRNA. | 1,03584 | 8,96969 | 3,69216 | 0,00450 | 0,02241 |
| RRAGC | Homo sapiens Ras-related GTP binding C (RRAGC), mRNA. | 1,02858 | 8,97200 | 5,61684 | 0,00026 | 0,00381 |
| PRKAG2 | Homo sapiens protein kinase, AMP-activated, gamma 2 non-catalytic subunit (PRKAG2), transcript variant c, mRNA. | 0,94216 | 8,51105 | 5,36345 | 0,00037 | 0,00453 |
| SH3GLB1 | Homo sapiens SH3-domain GRB2-like endophilin B1 (SH3GLB1), mRNA. | 0,93079 | 10,25645 | 8,42777 | 0,00001 | 0,00080 |
| PLEKHM1 | PREDICTED: Homo sapiens pleckstrin homology domain containing, family M (with RUN domain) member 1 (PLEKHM1), mRNA. | 0,92407 | 8,05844 | 3,46600 | 0,00649 | 0,02924 |
| HK2 | Homo sapiens hexokinase 2 (HK2), mRNA. | 0,89373 | 8,71941 | 4,26318 | 0,00183 | 0,01211 |
| MTMR3 | Homo sapiens myotubularin related protein 3 (MTMR3), transcript variant 3, mRNA. | 0,88228 | 8,35178 | 6,26453 | 0,00011 | 0,00239 |
| VPS28 | Homo sapiens vacuolar protein sorting 28 homolog (S. cerevisiae) (VPS28), transcript variant 1, mRNA. | 0,84308 | 9,76918 | 3,78067 | 0,00390 | 0,02022 |
| ATG2A | Homo sapiens ATG2 autophagy related 2 homolog A (S. cerevisiae) (ATG2A), mRNA. | 0,82563 | 7,85753 | 5,27255 | 0,00042 | 0,00492 |
| NRBF2 | Homo sapiens nuclear receptor binding factor 2 (NRBF2), mRNA. | 0,81733 | 8,17743 | 6,75174 | 0,00006 | 0,00180 |
| MTSS1 | Homo sapiens metastasis suppressor 1 (MTSS1), mRNA. | 0,79618 | 8,39421 | 4,60721 | 0,00109 | 0,00870 |
| RB1CC1 | Homo sapiens RB1-inducible coiled-coil 1 (RB1CC1), transcript variant 2, mRNA. | 0,78240 | 8,84843 | 4,89775 | 0,00071 | 0,00663 |
| STAT2 | Homo sapiens signal transducer and activator of transcription 2, 113kDa (STAT2), mRNA. | 0,75141 | 8,91837 | 4,10783 | 0,00233 | 0,01427 |
| SMURF1 | Homo sapiens SMAD specific E3 ubiquitin protein ligase 1 (SMURF1), transcript variant 2, mRNA. | 0,74715 | 7,90975 | 3,51146 | 0,00602 | 0,02772 |
| C12orf44 | Homo sapiens chromosome 12 open reading frame 44 (C12orf44), transcript variant 2, mRNA. | 0,73872 | 9,42565 | 4,79470 | 0,00083 | 0,00732 |
| FNBP1L | Homo sapiens formin binding protein 1-like (FNBP1L), transcript variant 1, mRNA. | 0,72454 | 8,46408 | 5,15778 | 0,00049 | 0,00538 |
| DAP | Homo sapiens death-associated protein (DAP), mRNA. | 0,71630 | 9,19919 | 3,96102 | 0,00293 | 0,01667 |
| MEX3C | Homo sapiens mex-3 homolog C (C. elegans) (MEX3C), mRNA. | 0,71626 | 8,34376 | 7,51167 | 0,00003 | 0,00121 |
| MTMR14 | Homo sapiens myotubularin related protein 14 (MTMR14), transcript variant 2, mRNA. | 0,67860 | 8,50924 | 5,47601 | 0,00032 | 0,00421 |
| UBQLN1 | Homo sapiens ubiquilin 1 (UBQLN1), transcript variant 2, mRNA. | 0,66896 | 9,55650 | 4,17415 | 0,00210 | 0,01331 |
| VPS28 | Homo sapiens vacuolar protein sorting 28 homolog (S. cerevisiae) (VPS28), transcript variant 1, mRNA. | 0,63824 | 9,18699 | 3,02909 | 0,01335 | 0,04942 |
| SLC35C1 | Homo sapiens solute carrier family 35, member C1 (SLC35C1), mRNA. | 0,61770 | 8,58541 | 7,23262 | 0,00004 | 0,00137 |
| CLDN7 | Homo sapiens claudin 7 (CLDN7), mRNA. | 0,59457 | 7,81207 | 4,46468 | 0,00135 | 0,00994 |
| STAM | Homo sapiens signal transducing adaptor molecule (SH3 domain and ITAM motif) 1 (STAM), mRNA. | 0,58274 | 8,40637 | 5,03618 | 0,00059 | 0,00593 |
| ATG9A | Homo sapiens ATG9 autophagy related 9 homolog A (S. cerevisiae) (ATG9A), transcript variant 1, mRNA. | 0,56332 | 8,45357 | 5,79990 | 0,00021 | 0,00332 |
| UBQLN1 | Homo sapiens ubiquilin 1 (UBQLN1), transcript variant 2, mRNA. | 0,54208 | 8,59062 | 4,21232 | 0,00198 | 0,01281 |
| VPS37C | Homo sapiens vacuolar protein sorting 37 homolog C (S. cerevisiae) (VPS37C), mRNA. | 0,53137 | 9,74474 | 4,01344 | 0,00270 | 0,01581 |
| NR2C2 | Homo sapiens nuclear receptor subfamily 2, group C, member 2 (NR2C2), mRNA. | 0,53003 | 7,74751 | 4,55915 | 0,00117 | 0,00909 |
| ATG9A | Homo sapiens ATG9 autophagy related 9 homolog A (S. cerevisiae) (ATG9A), transcript variant 1, mRNA. | 0,51482 | 8,71257 | 3,05316 | 0,01282 | 0,04800 |
| TP53INP1 | Homo sapiens tumor protein p53 inducible nuclear protein 1 (TP53INP1), mRNA. | 0,50233 | 7,76125 | 3,23963 | 0,00941 | 0,03803 |
| TMEM39B | Homo sapiens transmembrane protein 39B (TMEM39B), mRNA. | -0,54388 | 8,55520 | -5,43378 | 0,00034 | 0,00433 |
| USP13 | Homo sapiens ubiquitin specific peptidase 13 (isopeptidase T-3) (USP13), mRNA. | -0,55208 | 7,67014 | -6,71020 | 0,00007 | 0,00183 |
| TXLNA | Homo sapiens taxilin alpha (TXLNA), mRNA. | -0,55306 | 9,29169 | -6,72119 | 0,00007 | 0,00182 |
| COX8A | Homo sapiens cytochrome c oxidase subunit 8A (ubiquitous) (COX8A), mRNA. | -0,58192 | 12,95134 | -3,61156 | 0,00512 | 0,02454 |
| ATG4C | Homo sapiens ATG4 autophagy related 4 homolog C (S. cerevisiae) (ATG4C), transcript variant 7, mRNA. | -0,58194 | 8,03976 | -7,87160 | 0,00002 | 0,00101 |
| SNRPD1 | Homo sapiens small nuclear ribonucleoprotein D1 polypeptide 16kDa (SNRPD1), mRNA. | -0,59873 | 7,79476 | -6,56848 | 0,00008 | 0,00198 |
| PARK7 | Homo sapiens Parkinson disease (autosomal recessive, early onset) 7 (PARK7), mRNA. | -0,60413 | 11,71677 | -3,72560 | 0,00426 | 0,02155 |
| TOMM6 | Homo sapiens translocase of outer mitochondrial membrane 6 homolog (yeast) (TOMM6), nuclear gene encoding mitochondrial protein, mRNA. | -0,63538 | 10,56757 | -3,61288 | 0,00511 | 0,02451 |
| MYLK | Homo sapiens myosin light chain kinase (MYLK), transcript variant 8, mRNA. | -0,65551 | 7,68946 | -5,24793 | 0,00044 | 0,00499 |
| TOMM20 | Homo sapiens translocase of outer mitochondrial membrane 20 homolog (yeast) (TOMM20), nuclear gene encoding mitochondrial protein, mRNA. | -0,65786 | 10,99362 | -3,23026 | 0,00955 | 0,03843 |
| USP13 | Homo sapiens ubiquitin specific peptidase 13 (isopeptidase T-3) (USP13), mRNA. | -0,66831 | 7,96105 | -5,79986 | 0,00021 | 0,00332 |
| CHAF1B | Homo sapiens chromatin assembly factor 1, subunit B (p60) (CHAF1B), mRNA. | -0,67031 | 7,82196 | -7,61251 | 0,00002 | 0,00115 |
| MFN2 | Homo sapiens mitofusin 2 (MFN2), nuclear gene encoding mitochondrial protein, mRNA. | -0,69969 | 9,02156 | -6,41131 | 0,00009 | 0,00216 |
| HSPA8 | Homo sapiens heat shock 70kDa protein 8 (HSPA8), transcript variant 2, mRNA. | -0,70982 | 12,37837 | -3,40184 | 0,00720 | 0,03158 |
| TOMM20 | Homo sapiens translocase of outer mitochondrial membrane 20 homolog (yeast) (TOMM20), nuclear gene encoding mitochondrial protein, mRNA. | -0,71038 | 10,27750 | -3,85171 | 0,00349 | 0,01871 |
| BLOC1S1 | Homo sapiens biogenesis of lysosome-related organelles complex-1, subunit 1 (BLOC1S1), mRNA. | -0,72387 | 8,33632 | -4,48344 | 0,00131 | 0,00976 |
| NUP93 | Homo sapiens nucleoporin 93kDa (NUP93), mRNA. | -0,75570 | 10,00502 | -3,87474 | 0,00336 | 0,01826 |
| SNRPB | Homo sapiens small nuclear ribonucleoprotein polypeptides B and B1 (SNRPB), transcript variant 2, mRNA. | -0,75681 | 11,66754 | -4,34011 | 0,00163 | 0,01121 |
| TMEM203 | Homo sapiens transmembrane protein 203 (TMEM203), mRNA. | -0,76372 | 9,39552 | -6,32091 | 0,00011 | 0,00229 |
| EI24 | Homo sapiens etoposide induced 2.4 mRNA (EI24), transcript variant 2, mRNA. | -0,76841 | 10,26727 | -8,05439 | 0,00001 | 0,00095 |
| TOMM22 | Homo sapiens translocase of outer mitochondrial membrane 22 homolog (yeast) (TOMM22), nuclear gene encoding mitochondrial protein, mRNA. | -0,81450 | 9,32872 | -6,86243 | 0,00006 | 0,00169 |
| FANCL | Homo sapiens Fanconi anemia, complementation group L (FANCL), mRNA. | -0,88078 | 7,91916 | -6,64407 | 0,00007 | 0,00190 |
| PLOD2 | Homo sapiens procollagen-lysine, 2-oxoglutarate 5-dioxygenase 2 (PLOD2), transcript variant 2, mRNA. | -0,90805 | 9,70168 | -3,74694 | 0,00412 | 0,02105 |
| ATP1B1 | Homo sapiens ATPase, Na+/K+ transporting, beta 1 polypeptide (ATP1B1), transcript variant 1, mRNA. | -0,90876 | 9,50575 | -5,30155 | 0,00040 | 0,00479 |
| RFWD3 | Homo sapiens ring finger and WD repeat domain 3 (RFWD3), mRNA. | -0,91329 | 8,79470 | -6,70667 | 0,00007 | 0,00183 |
| TOMM5 | Homo sapiens translocase of outer mitochondrial membrane 5 homolog (yeast) (TOMM5), nuclear gene encoding mitochondrial protein, transcript variant 1, mRNA. | -0,95653 | 11,04395 | -6,30453 | 0,00011 | 0,00231 |
| SNRPF | Homo sapiens small nuclear ribonucleoprotein polypeptide F (SNRPF), mRNA. | -0,96599 | 11,83932 | -4,02694 | 0,00264 | 0,01558 |
| SLC37A4 | Homo sapiens solute carrier family 37 (glucose-6-phosphate transporter), member 4 (SLC37A4), mRNA. | -1,00736 | 9,24989 | -4,30092 | 0,00173 | 0,01170 |
| TMEM203 | Homo sapiens transmembrane protein 203 (TMEM203), mRNA. | -1,01584 | 9,54477 | -7,65635 | 0,00002 | 0,00113 |
| SNRPF | Homo sapiens small nuclear ribonucleoprotein polypeptide F (SNRPF), mRNA. | -1,03628 | 11,45882 | -4,30901 | 0,00171 | 0,01159 |
| TOMM40 | Homo sapiens translocase of outer mitochondrial membrane 40 homolog (yeast) (TOMM40), nuclear gene encoding mitochondrial protein, mRNA. | -1,05119 | 10,81225 | -4,06950 | 0,00247 | 0,01491 |
| DYNLL1 | Homo sapiens dynein, light chain, LC8-type 1 (DYNLL1), transcript variant 1, mRNA. | -1,06426 | 11,23961 | -6,10948 | 0,00014 | 0,00267 |
| HSP90AA1 | Homo sapiens heat shock protein 90kDa alpha (cytosolic), class A member 1 (HSP90AA1), transcript variant 1, mRNA. | -1,07338 | 12,18221 | -3,20891 | 0,00990 | 0,03947 |
| ATP1B1 | Homo sapiens ATPase, Na+/K+ transporting, beta 1 polypeptide (ATP1B1), transcript variant 2, mRNA. | -1,24781 | 10,42939 | -5,08871 | 0,00054 | 0,00568 |
| ATP1B1 | Homo sapiens ATPase, Na+/K+ transporting, beta 1 polypeptide (ATP1B1), transcript variant 1, mRNA. | -1,29035 | 9,53913 | -5,68347 | 0,00024 | 0,00361 |
| LSM4 | Homo sapiens LSM4 homolog, U6 small nuclear RNA associated (S. cerevisiae) (LSM4), mRNA. | -1,29678 | 10,59295 | -5,12313 | 0,00052 | 0,00551 |
| MDH1 | Homo sapiens malate dehydrogenase 1, NAD (soluble) (MDH1), mRNA. | -1,51850 | 11,61712 | -4,80328 | 0,00082 | 0,00725 |
| PNPO | Homo sapiens pyridoxamine 5'-phosphate oxidase (PNPO), mRNA. | -1,63793 | 9,26012 | -5,91429 | 0,00018 | 0,00306 |
