# Supplementary material for: Induction of ER and mitochondrial stress by the alkylphosphocholine erufosine in oral squamous cell carcinoma cells
Source: Cell Death Dis. 2018 Feb 20;9(3):296. doi: 10.1038/s41419-018-0342-2 (PMC5833417; doi:10.1038/s41419-018-0342-2)
Supplement: Supplementary file 15 — Supplementary Table 6a [file 41419_2018_342_MOESM15_ESM.docx]

Table S6a: Differential regulation of apoptotic genes upon IC25 exposure of erufosine in HN-5 cells

| **Symbol** | **Definition** | **Log Fold Change** | **Average Expression** | **t-statistics** | **P.Value** | **adj.P.Val** |
| --- | --- | --- | --- | --- | --- | --- |
| CDKN1A | Homo sapiens cyclin-dependent kinase inhibitor 1A (p21, Cip1) (CDKN1A), transcript variant 1, mRNA. | 2,30193 | 11,50962 | 6,20365 | 0,0001228 | 0,0401145 |
| RHOB | Homo sapiens ras homolog gene family, member B (RHOB), mRNA. | 2,16758 | 10,15124 | 7,77621 | 0,0000197 | 0,0192045 |
| EMP1 | Homo sapiens epithelial membrane protein 1 (EMP1), mRNA. | 1,56303 | 10,78806 | 7,55856 | 0,0000249 | 0,0197879 |
| SLC20A1 | Homo sapiens solute carrier family 20 (phosphate transporter), member 1 (SLC20A1), mRNA. | 1,19226 | 11,40864 | 8,17157 | 0,0000130 | 0,0166227 |
| BCL2L1 | Homo sapiens BCL2-like 1 (BCL2L1), nuclear gene encoding mitochondrial protein, transcript variant 1, mRNA. | 1,00295 | 11,43766 | 6,14957 | 0,0001315 | 0,0401626 |
