# Supplementary material for: Induction of ER and mitochondrial stress by the alkylphosphocholine erufosine in oral squamous cell carcinoma cells
Source: Cell Death Dis. 2018 Feb 20;9(3):296. doi: 10.1038/s41419-018-0342-2 (PMC5833417; doi:10.1038/s41419-018-0342-2)
Supplement: Supplementary file 16 — Supplementary Table 6b [file 41419_2018_342_MOESM16_ESM.docx]

Table S6b: Differential regulation of apoptotic genes upon IC50 exposure of erufosine in HN-5 cells

| **Symbol** | **Definition** | **Log Fold Change** | **Average Expression** | **t-statistics** | **P.Value** | **adj.P.Val** |
| --- | --- | --- | --- | --- | --- | --- |
| CDKN1A | Homo sapiens cyclin-dependent kinase inhibitor 1A (p21, Cip1) (CDKN1A), transcript variant 1, mRNA. | 3,55917 | 11,50962 | 9,59188 | 3,27E-06 | 1,67E-03 |
| RHOB | Homo sapiens ras homolog gene family, member B (RHOB), mRNA. | 3,20507 | 10,15124 | 11,49822 | 6,57E-07 | 6,49E-04 |
| IL1B | Homo sapiens interleukin 1, beta (IL1B), mRNA. | 3,11814 | 11,03957 | 4,13765 | 2,23E-03 | 2,83E-02 |
| ISG20 | Homo sapiens interferon stimulated exonuclease gene 20kDa (ISG20), mRNA. | 2,10418 | 9,91594 | 5,19799 | 4,67E-04 | 1,24E-02 |
| IER3 | Homo sapiens immediate early response 3 (IER3), mRNA. | 1,93910 | 12,79992 | 6,14264 | 1,33E-04 | 7,04E-03 |
| IL1A | Homo sapiens interleukin 1, alpha (IL1A), mRNA. | 1,91772 | 11,28831 | 7,74395 | 2,04E-05 | 3,25E-03 |
| EMP1 | Homo sapiens epithelial membrane protein 1 (EMP1), mRNA. | 1,91284 | 10,78806 | 9,25021 | 4,48E-06 | 1,88E-03 |
| SAT1 | Homo sapiens spermidine/spermine N1-acetyltransferase 1 (SAT1), mRNA. | 1,80526 | 11,07082 | 6,78625 | 6,02E-05 | 4,79E-03 |
| JUN | Homo sapiens jun oncogene (JUN), mRNA. | 1,45151 | 10,79761 | 7,10243 | 4,16E-05 | 4,22E-03 |
| BCL2L1 | Homo sapiens BCL2-like 1 (BCL2L1), nuclear gene encoding mitochondrial protein, transcript variant 1, mRNA. | 1,38610 | 11,43766 | 8,49890 | 9,29E-06 | 2,36E-03 |
| GNA15 | Homo sapiens guanine nucleotide binding protein (G protein), alpha 15 (Gq class) (GNA15), mRNA. | 1,34588 | 9,64492 | 6,97193 | 4,84E-05 | 4,48E-03 |
| SLC20A1 | Homo sapiens solute carrier family 20 (phosphate transporter), member 1 (SLC20A1), mRNA. | 1,29372 | 11,40864 | 8,86694 | 6,46E-06 | 1,98E-03 |
| TIMP1 | Homo sapiens TIMP metallopeptidase inhibitor 1 (TIMP1), mRNA. | 1,25737 | 10,41281 | 5,58723 | 2,74E-04 | 9,60E-03 |
| MCL1 | Homo sapiens myeloid cell leukemia sequence 1 (BCL2-related) (MCL1), transcript variant 1, mRNA. | 1,10309 | 9,72149 | 5,97636 | 1,64E-04 | 7,56E-03 |
| GADD45A | Homo sapiens growth arrest and DNA-damage-inducible, alpha (GADD45A), mRNA. | 1,08282 | 9,89642 | 5,13282 | 5,11E-04 | 1,30E-02 |
| GADD45A | Homo sapiens growth arrest and DNA-damage-inducible, alpha (GADD45A), mRNA. | 1,08245 | 9,61294 | 4,66166 | 1,01E-03 | 1,83E-02 |
| ANXA1 | Homo sapiens annexin A1 (ANXA1), mRNA. | 0,84074 | 12,82326 | 6,55385 | 7,96E-05 | 5,55E-03 |
| DAP | Homo sapiens death-associated protein (DAP), mRNA. | 0,83836 | 9,19919 | 4,63599 | 1,05E-03 | 1,88E-02 |
| ATF3 | Homo sapiens activating transcription factor 3 (ATF3), transcript variant 4, mRNA. | 0,79794 | 8,58906 | 5,38991 | 3,58E-04 | 1,10E-02 |
| TNFRSF12A | Homo sapiens tumor necrosis factor receptor superfamily, member 12A (TNFRSF12A), mRNA. | 0,79445 | 11,50558 | 8,55686 | 8,77E-06 | 2,36E-03 |
| BCL2L1 | Homo sapiens BCL2-like 1 (BCL2L1), nuclear gene encoding mitochondrial protein, transcript variant 1, mRNA. | 0,78411 | 8,31739 | 6,41167 | 9,47E-05 | 6,09E-03 |
| DDIT3 | Homo sapiens DNA-damage-inducible transcript 3 (DDIT3), mRNA. | 0,76275 | 8,13181 | 5,45107 | 3,29E-04 | 1,05E-02 |
| BNIP3L | Homo sapiens BCL2/adenovirus E1B 19kDa interacting protein 3-like (BNIP3L), mRNA. | 0,72694 | 8,99688 | 3,76953 | 3,97E-03 | 3,99E-02 |
| SQSTM1 | Homo sapiens sequestosome 1 (SQSTM1), mRNA. | 0,69621 | 12,84273 | 5,01068 | 6,08E-04 | 1,42E-02 |
| BCL2L2 | Homo sapiens BCL2-like 2 (BCL2L2), mRNA. | 0,63521 | 9,90133 | 3,85767 | 3,45E-03 | 3,68E-02 |
| PEA15 | Homo sapiens phosphoprotein enriched in astrocytes 15 (PEA15), mRNA. | 0,63450 | 10,13164 | 4,78385 | 8,43E-04 | 1,69E-02 |
| TSPO | Homo sapiens translocator protein (18kDa) (TSPO), transcript variant PBR, mRNA. | 0,57287 | 11,31828 | 4,28923 | 1,76E-03 | 2,50E-02 |
| RELA | Homo sapiens v-rel reticuloendotheliosis viral oncogene homolog A (avian) (RELA), mRNA. | 0,57246 | 8,52701 | 3,78326 | 3,89E-03 | 3,93E-02 |
| BRCA1 | Homo sapiens breast cancer 1, early onset (BRCA1), transcript variant BRCA1-delta14-17, mRNA. | -0,55159 | 7,78842 | -5,33213 | 3,87E-04 | 1,13E-02 |
| BID | Homo sapiens BH3 interacting domain death agonist (BID), transcript variant 1, mRNA. | -0,55263 | 8,16570 | -5,14296 | 5,04E-04 | 1,29E-02 |
| BRCA1 | Homo sapiens breast cancer 1, early onset (BRCA1), transcript variant BRCA1-delta11b, mRNA. | -0,58930 | 7,63589 | -5,38922 | 3,58E-04 | 1,10E-02 |
| DNAJA1 | Homo sapiens DnaJ (Hsp40) homolog, subfamily A, member 1 (DNAJA1), mRNA. | -0,60625 | 11,63228 | -3,85680 | 3,46E-03 | 3,68E-02 |
| CDK2 | Homo sapiens cyclin-dependent kinase 2 (CDK2), transcript variant 1, mRNA. | -0,68233 | 9,11065 | -5,20990 | 4,59E-04 | 1,24E-02 |
| CASP2 | Homo sapiens caspase 2, apoptosis-related cysteine peptidase (CASP2), transcript variant 1, mRNA. | -0,71169 | 9,39808 | -4,36274 | 1,58E-03 | 2,36E-02 |
| RARA | Homo sapiens retinoic acid receptor, alpha (RARA), transcript variant 1, mRNA. | -0,87519 | 8,52297 | -3,89361 | 3,26E-03 | 3,55E-02 |
| HMGB2 | Homo sapiens high-mobility group box 2 (HMGB2), mRNA. | -1,20989 | 8,72458 | -3,88535 | 3,30E-03 | 3,57E-02 |
