# Supplementary material for: Induction of ER and mitochondrial stress by the alkylphosphocholine erufosine in oral squamous cell carcinoma cells
Source: Cell Death Dis. 2018 Feb 20;9(3):296. doi: 10.1038/s41419-018-0342-2 (PMC5833417; doi:10.1038/s41419-018-0342-2)
Supplement: Supplementary file 17 — Supplementary Table 6c [file 41419_2018_342_MOESM17_ESM.docx]

Table S6c: Differential regulation of apoptotic genes upon IC75 exposure of erufosine in HN-5 cells

| **Symbol** | **Definition** | **Log Fold Change** | **Average Expression** | **t-statistics** | **P.Value** | **adj.P.Val** |
| --- | --- | --- | --- | --- | --- | --- |
| CDKN1A | Homo sapiens cyclin-dependent kinase inhibitor 1A (p21, Cip1) (CDKN1A), transcript variant 1, mRNA. | 3,85223 | 11,50962 | 10,38168 | 1,631E-06 | 4,055E-04 |
| RHOB | Homo sapiens ras homolog gene family, member B (RHOB), mRNA. | 3,26956 | 10,15124 | 11,72959 | 5,492E-07 | 2,815E-04 |
| IL1B | Homo sapiens interleukin 1, beta (IL1B), mRNA. | 2,78832 | 11,03957 | 3,69999 | 4,441E-03 | 2,220E-02 |
| ATF3 | Homo sapiens activating transcription factor 3 (ATF3), transcript variant 4, mRNA. | 2,74294 | 8,58906 | 18,52805 | 8,281E-09 | 7,330E-05 |
| ISG20 | Homo sapiens interferon stimulated exonuclease gene 20kDa (ISG20), mRNA. | 2,32677 | 9,91594 | 5,74786 | 2,210E-04 | 3,454E-03 |
| SAT1 | Homo sapiens spermidine/spermine N1-acetyltransferase 1 (SAT1), mRNA. | 2,30291 | 11,07082 | 8,65698 | 7,939E-06 | 7,267E-04 |
| JUN | Homo sapiens jun oncogene (JUN), mRNA. | 2,24307 | 10,79761 | 10,97564 | 9,953E-07 | 3,085E-04 |
| EMP1 | Homo sapiens epithelial membrane protein 1 (EMP1), mRNA. | 2,12700 | 10,78806 | 10,28583 | 1,770E-06 | 4,060E-04 |
| IER3 | Homo sapiens immediate early response 3 (IER3), mRNA. | 1,98122 | 12,79992 | 6,27608 | 1,121E-04 | 2,371E-03 |
| PMAIP1 | Homo sapiens phorbol-12-myristate-13-acetate-induced protein 1 (PMAIP1), mRNA. | 1,90872 | 8,58437 | 5,55270 | 2,867E-04 | 3,980E-03 |
| IL1A | Homo sapiens interleukin 1, alpha (IL1A), mRNA. | 1,66567 | 11,28831 | 6,72616 | 6,469E-05 | 1,819E-03 |
| GNA15 | Homo sapiens guanine nucleotide binding protein (G protein), alpha 15 (Gq class) (GNA15), mRNA. | 1,50961 | 9,64492 | 7,82011 | 1,878E-05 | 1,043E-03 |
| HMOX1 | Homo sapiens heme oxygenase (decycling) 1 (HMOX1), mRNA. | 1,50581 | 7,85828 | 4,71810 | 9,276E-04 | 7,902E-03 |
| SLC20A1 | Homo sapiens solute carrier family 20 (phosphate transporter), member 1 (SLC20A1), mRNA. | 1,46793 | 11,40864 | 10,06102 | 2,151E-06 | 4,329E-04 |
| BIRC3 | Homo sapiens baculoviral IAP repeat-containing 3 (BIRC3), transcript variant 2, mRNA. | 1,36084 | 8,14328 | 5,42448 | 3,412E-04 | 4,341E-03 |
| MCL1 | Homo sapiens myeloid cell leukemia sequence 1 (BCL2-related) (MCL1), transcript variant 1, mRNA. | 1,32303 | 9,72149 | 7,16795 | 3,863E-05 | 1,414E-03 |
| GADD45A | Homo sapiens growth arrest and DNA-damage-inducible, alpha (GADD45A), mRNA. | 1,31296 | 9,61294 | 5,65436 | 2,502E-04 | 3,689E-03 |
| GADD45A | Homo sapiens growth arrest and DNA-damage-inducible, alpha (GADD45A), mRNA. | 1,31151 | 9,89642 | 6,21683 | 1,208E-04 | 2,453E-03 |
| SQSTM1 | Homo sapiens sequestosome 1 (SQSTM1), mRNA. | 1,20357 | 12,84273 | 8,66219 | 7,898E-06 | 7,267E-04 |
| TIMP1 | Homo sapiens TIMP metallopeptidase inhibitor 1 (TIMP1), mRNA. | 1,16950 | 10,41281 | 5,19676 | 4,673E-04 | 5,220E-03 |
| DDIT3 | Homo sapiens DNA-damage-inducible transcript 3 (DDIT3), mRNA. | 1,16799 | 8,13181 | 8,34717 | 1,083E-05 | 8,344E-04 |
| H1F0 | Homo sapiens H1 histone family, member 0 (H1F0), mRNA. | 1,14449 | 10,61746 | 3,78162 | 3,897E-03 | 2,021E-02 |
| BCL2L1 | Homo sapiens BCL2-like 1 (BCL2L1), nuclear gene encoding mitochondrial protein, transcript variant 1, mRNA. | 1,03053 | 11,43766 | 6,31872 | 1,063E-04 | 2,292E-03 |
| IL18 | Homo sapiens interleukin 18 (interferon-gamma-inducing factor) (IL18), mRNA. | 0,99429 | 11,78929 | 8,96363 | 5,886E-06 | 6,389E-04 |
| IGFBP6 | Homo sapiens insulin-like growth factor binding protein 6 (IGFBP6), mRNA. | 0,98343 | 10,20949 | 3,70638 | 4,395E-03 | 2,201E-02 |
| ANXA1 | Homo sapiens annexin A1 (ANXA1), mRNA. | 0,93363 | 12,82326 | 7,27791 | 3,409E-05 | 1,340E-03 |
| NEDD9 | Homo sapiens neural precursor cell expressed, developmentally down-regulated 9 (NEDD9), transcript variant 2, mRNA. | 0,90154 | 7,54138 | 12,53619 | 3,014E-07 | 2,163E-04 |
| SPTAN1 | Homo sapiens spectrin, alpha, non-erythrocytic 1 (alpha-fodrin) (SPTAN1), mRNA. | 0,84021 | 8,61658 | 5,16973 | 4,853E-04 | 5,333E-03 |
| RELA | Homo sapiens v-rel reticuloendotheliosis viral oncogene homolog A (avian) (RELA), mRNA. | 0,81408 | 8,52701 | 5,38007 | 3,626E-04 | 4,471E-03 |
| MCL1 | Homo sapiens myeloid cell leukemia sequence 1 (BCL2-related) (MCL1), transcript variant 1, mRNA. | 0,81094 | 8,55268 | 3,31810 | 8,265E-03 | 3,478E-02 |
| BNIP3L | Homo sapiens BCL2/adenovirus E1B 19kDa interacting protein 3-like (BNIP3L), mRNA. | 0,80420 | 8,99688 | 4,17015 | 2,117E-03 | 1,337E-02 |
| BCL2L2 | Homo sapiens BCL2-like 2 (BCL2L2), mRNA. | 0,79824 | 9,90133 | 4,84779 | 7,678E-04 | 6,961E-03 |
| PMAIP1 | Homo sapiens phorbol-12-myristate-13-acetate-induced protein 1 (PMAIP1), mRNA. | 0,76897 | 7,68132 | 3,83366 | 3,587E-03 | 1,910E-02 |
| BMP2 | Homo sapiens bone morphogenetic protein 2 (BMP2), mRNA. | 0,76378 | 8,14185 | 5,59850 | 2,696E-04 | 3,856E-03 |
| TNFRSF12A | Homo sapiens tumor necrosis factor receptor superfamily, member 12A (TNFRSF12A), mRNA. | 0,73576 | 11,50558 | 7,92478 | 1,680E-05 | 9,818E-04 |
| DAP | Homo sapiens death-associated protein (DAP), mRNA. | 0,71630 | 9,19919 | 3,96102 | 2,933E-03 | 1,667E-02 |
| PEA15 | Homo sapiens phosphoprotein enriched in astrocytes 15 (PEA15), mRNA. | 0,71484 | 10,13164 | 5,38957 | 3,579E-04 | 4,434E-03 |
| PDCD4 | Homo sapiens programmed cell death 4 (neoplastic transformation inhibitor) (PDCD4), transcript variant 2, mRNA. | 0,70394 | 8,67024 | 4,18825 | 2,059E-03 | 1,311E-02 |
| SOD2 | Homo sapiens superoxide dismutase 2, mitochondrial (SOD2), nuclear gene encoding mitochondrial protein, transcript variant 2, mRNA. | 0,69276 | 9,48525 | 4,11619 | 2,301E-03 | 1,412E-02 |
| BCL10 | Homo sapiens B-cell CLL/lymphoma 10 (BCL10), mRNA. | 0,69185 | 8,17444 | 5,47484 | 3,186E-04 | 4,216E-03 |
| BCL2L1 | Homo sapiens BCL2-like 1 (BCL2L1), nuclear gene encoding mitochondrial protein, transcript variant 1, mRNA. | 0,69025 | 8,31739 | 5,64418 | 2,536E-04 | 3,722E-03 |
| PPP2R5B | Homo sapiens protein phosphatase 2, regulatory subunit B', beta isoform (PPP2R5B), mRNA. | 0,66852 | 7,70041 | 9,69957 | 2,965E-06 | 4,915E-04 |
| IFNGR1 | Homo sapiens interferon gamma receptor 1 (IFNGR1), mRNA. | 0,58700 | 8,96240 | 5,74619 | 2,215E-04 | 3,454E-03 |
| BTG3 | Homo sapiens BTG family, member 3 (BTG3), mRNA. | 0,58265 | 9,36936 | 4,51216 | 1,259E-03 | 9,467E-03 |
| GADD45B | Homo sapiens growth arrest and DNA-damage-inducible, beta (GADD45B), mRNA. | 0,56810 | 8,07063 | 4,39379 | 1,504E-03 | 1,066E-02 |
| DNAJC3 | Homo sapiens DnaJ (Hsp40) homolog, subfamily C, member 3 (DNAJC3), mRNA. | 0,51436 | 7,74047 | 4,01436 | 2,697E-03 | 1,580E-02 |
| BID | Homo sapiens BH3 interacting domain death agonist (BID), transcript variant 1, mRNA. | -0,50201 | 8,16570 | -4,67196 | 9,927E-04 | 8,215E-03 |
| LEF1 | Homo sapiens lymphoid enhancer-binding factor 1 (LEF1), mRNA. | -0,51546 | 7,78822 | -7,66047 | 2,231E-05 | 1,131E-03 |
| DFFA | Homo sapiens DNA fragmentation factor, 45kDa, alpha polypeptide (DFFA), transcript variant 2, mRNA. | -0,55321 | 8,70579 | -3,34574 | 7,898E-03 | 3,372E-02 |
| DNAJA1 | Homo sapiens DnaJ (Hsp40) homolog, subfamily A, member 1 (DNAJA1), mRNA. | -0,56250 | 11,63228 | -3,57846 | 5,402E-03 | 2,551E-02 |
| LEF1 | Homo sapiens lymphoid enhancer-binding factor 1 (LEF1), mRNA. | -0,59973 | 7,81828 | -7,36095 | 3,105E-05 | 1,285E-03 |
| BRCA1 | Homo sapiens breast cancer 1, early onset (BRCA1), transcript variant BRCA1-delta11b, mRNA. | -0,65567 | 7,63589 | -5,99617 | 1,599E-04 | 2,892E-03 |
| CDK2 | Homo sapiens cyclin-dependent kinase 2 (CDK2), transcript variant 1, mRNA. | -0,71141 | 9,11065 | -5,43192 | 3,377E-04 | 4,330E-03 |
| RARA | Homo sapiens retinoic acid receptor, alpha (RARA), transcript variant 1, mRNA. | -0,73870 | 8,52297 | -3,28640 | 8,708E-03 | 3,605E-02 |
| BRCA1 | Homo sapiens breast cancer 1, early onset (BRCA1), transcript variant BRCA1-delta14-17, mRNA. | -0,76029 | 7,78842 | -7,34967 | 3,145E-05 | 1,291E-03 |
| CASP1 | Homo sapiens caspase 1, apoptosis-related cysteine peptidase (interleukin 1, beta, convertase) (CASP1), transcript variant delta, mRNA. | -0,79391 | 8,07587 | -3,93077 | 3,076E-03 | 1,715E-02 |
| CASP1 | Homo sapiens caspase 1, apoptosis-related cysteine peptidase (interleukin 1, beta, convertase) (CASP1), transcript variant delta, mRNA. | -0,82478 | 8,04560 | -3,69490 | 4,477E-03 | 2,234E-02 |
| TNFSF10 | Homo sapiens tumor necrosis factor (ligand) superfamily, member 10 (TNFSF10), mRNA. | -0,84267 | 8,05185 | -3,85827 | 3,449E-03 | 1,857E-02 |
| CASP2 | Homo sapiens caspase 2, apoptosis-related cysteine peptidase (CASP2), transcript variant 1, mRNA. | -1,07444 | 9,39808 | -6,58640 | 7,653E-05 | 1,958E-03 |
| PPT1 | Homo sapiens palmitoyl-protein thioesterase 1 (ceroid-lipofuscinosis, neuronal 1, infantile) (PPT1), mRNA. | -1,24202 | 10,61318 | -3,43992 | 6,768E-03 | 3,020E-02 |
| HMGB2 | Homo sapiens high-mobility group box 2 (HMGB2), mRNA. | -1,28343 | 8,72458 | -4,12150 | 2,282E-03 | 1,404E-02 |
| HMGB2 | Homo sapiens high-mobility group box 2 (HMGB2), mRNA. | -1,43599 | 9,03780 | -3,62124 | 5,041E-03 | 2,427E-02 |
| CAV1 | Homo sapiens caveolin 1, caveolae protein, 22kDa (CAV1), mRNA. | -1,61747 | 12,82809 | -3,54925 | 5,664E-03 | 2,643E-02 |
| CAV1 | Homo sapiens caveolin 1, caveolae protein, 22kDa (CAV1), mRNA. | -1,65950 | 9,84065 | -3,99507 | 2,780E-03 | 1,607E-02 |
| TOP2A | Homo sapiens topoisomerase (DNA) II alpha 170kDa (TOP2A), mRNA. | -2,42609 | 9,89283 | -3,14042 | 1,109E-02 | 4,289E-02 |
