# Supplementary material for: Induction of ER and mitochondrial stress by the alkylphosphocholine erufosine in oral squamous cell carcinoma cells
Source: Cell Death Dis. 2018 Feb 20;9(3):296. doi: 10.1038/s41419-018-0342-2 (PMC5833417; doi:10.1038/s41419-018-0342-2)
Supplement: Supplementary file 18 — Supplementary Table 7 [file 41419_2018_342_MOESM18_ESM.docx]

**Table S7. Primer sequences for qRT-PCR**

| **Gene** | **Forward Primer** | **Reverse Primer** | **Probe Number^a)^** |
| --- | --- | --- | --- |
| ATF3 | tttgccatccagaacaagc | catcttcttcaggggctacct | 53 |
| PERK | ccagccttagcaaaccagag | tcttggtcccactggaagag | 58 |
| DDIT3 | cagagctggaacctgaggag | tgtttatggctgctttggtg | 9 |
| XBP1 | ggagttaagacagcgcttgg | cactggcctcacttcattcc | 37 |
| ATF4 | tggtcagtccctccaacaac | ctatacccaacagggcatcc | 88 |
| EDEM1 | gccgaaacctcatgagttaaa | tcatcaggtacacgattgcag | 78 |
| DNAJB9 | ccaccctgacaaaaataagagc | tcttttcgtctattagcatctgagagt | 89 |
| HSPA5 | agctgtagcgtatggtgctg | aaggggacatacatcaagcagt | 64 |
| ATG5 | ccgcaggacagtgtgtca | tttcaaccaaagccaaacct | 12 |
| GAPDH | agccacatcgctcagacac | gcccaatacgaccaaatcc | 60 |

**^a)^** Probe number from probes of the Universal Probe Library (Roche)
