# Supplementary material for: Induction of ER and mitochondrial stress by the alkylphosphocholine erufosine in oral squamous cell carcinoma cells
Source: Cell Death Dis. 2018 Feb 20;9(3):296. doi: 10.1038/s41419-018-0342-2 (PMC5833417; doi:10.1038/s41419-018-0342-2)
Supplement: Supplementary file 19 — Supplementary Table 8 [file 41419_2018_342_MOESM19_ESM.docx]

**Table S8. shRNA oligo sequences for PERK and XBP1**

| **Gene** | **Forward Sequence (5’-3’)** | **Reverse Sequence (5’-3’)** |
| --- | --- | --- |
| PERK  shRNA TRCN0000262382 | CCGGGGCAACCATTGTGCTAATAAACTCGAGTTTATTAGCACAATGGTTGCCTTTTTG | AATTCAAAAAGGCAACCATTGTGCTAATAAACTCGAGTTTATTAGCACAATGGTTGCC |
| XBP1  shRNA  (in CDS) | CCGGGAACAGCAAGTGGTAGATTTACTCGAGTAAATCTACCACTTGCTGTTCTTTTTG | AATTCAAAAAGAACAGCAAGTGGTAGATTTACTCGAGTAAATCTACCACTTGCTGTTC |
