# Supplementary figures and images for: Induction of ER and mitochondrial stress by the alkylphosphocholine erufosine in oral squamous cell carcinoma cells
Source: Cell Death Dis. 2018 Feb 20;9(3):296. doi: 10.1038/s41419-018-0342-2 (PMC5833417; doi:10.1038/s41419-018-0342-2)

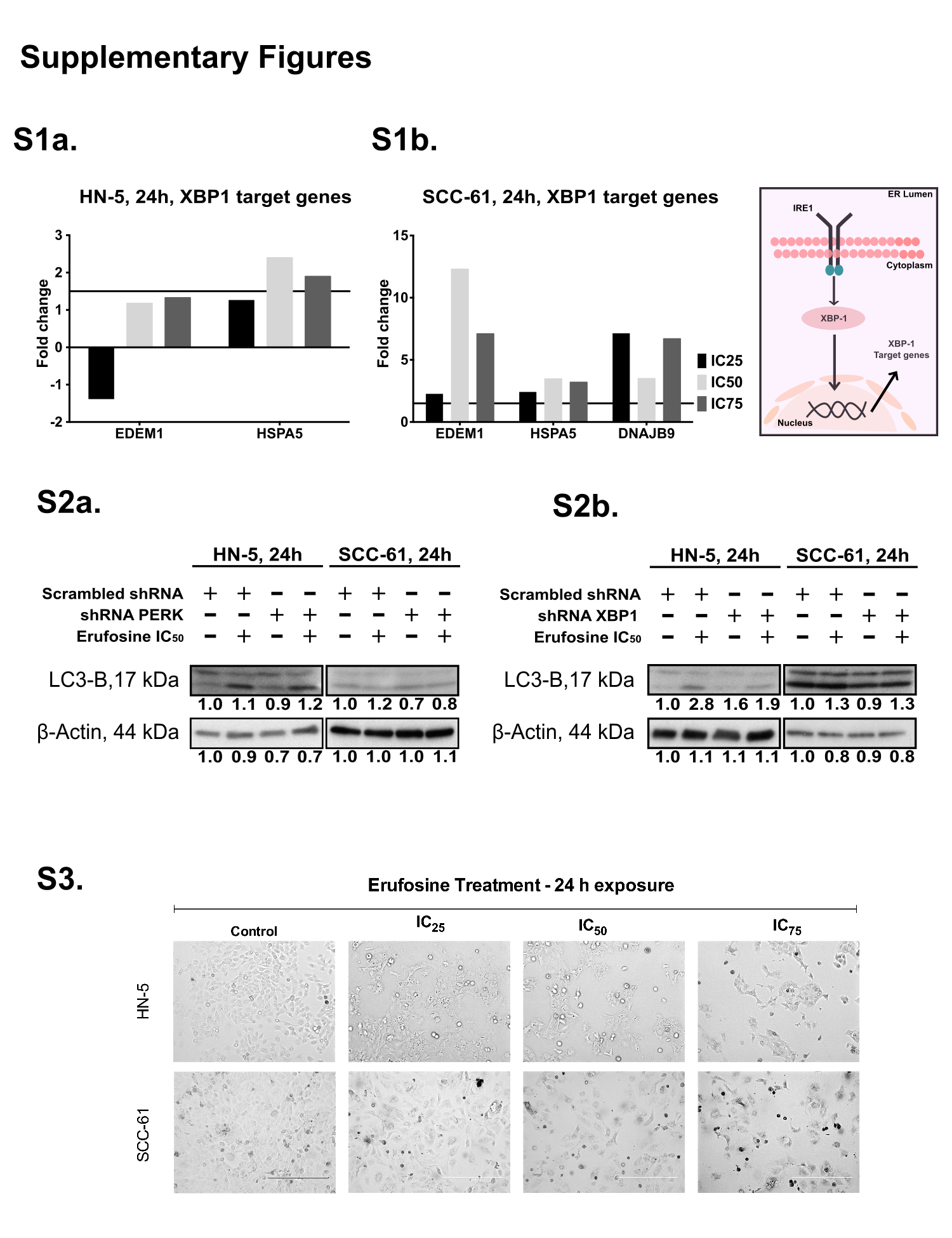

Supplement: Supplementary file 20 — Suppl. Figures S1-S3 [file 41419_2018_342_MOESM20_ESM.png]

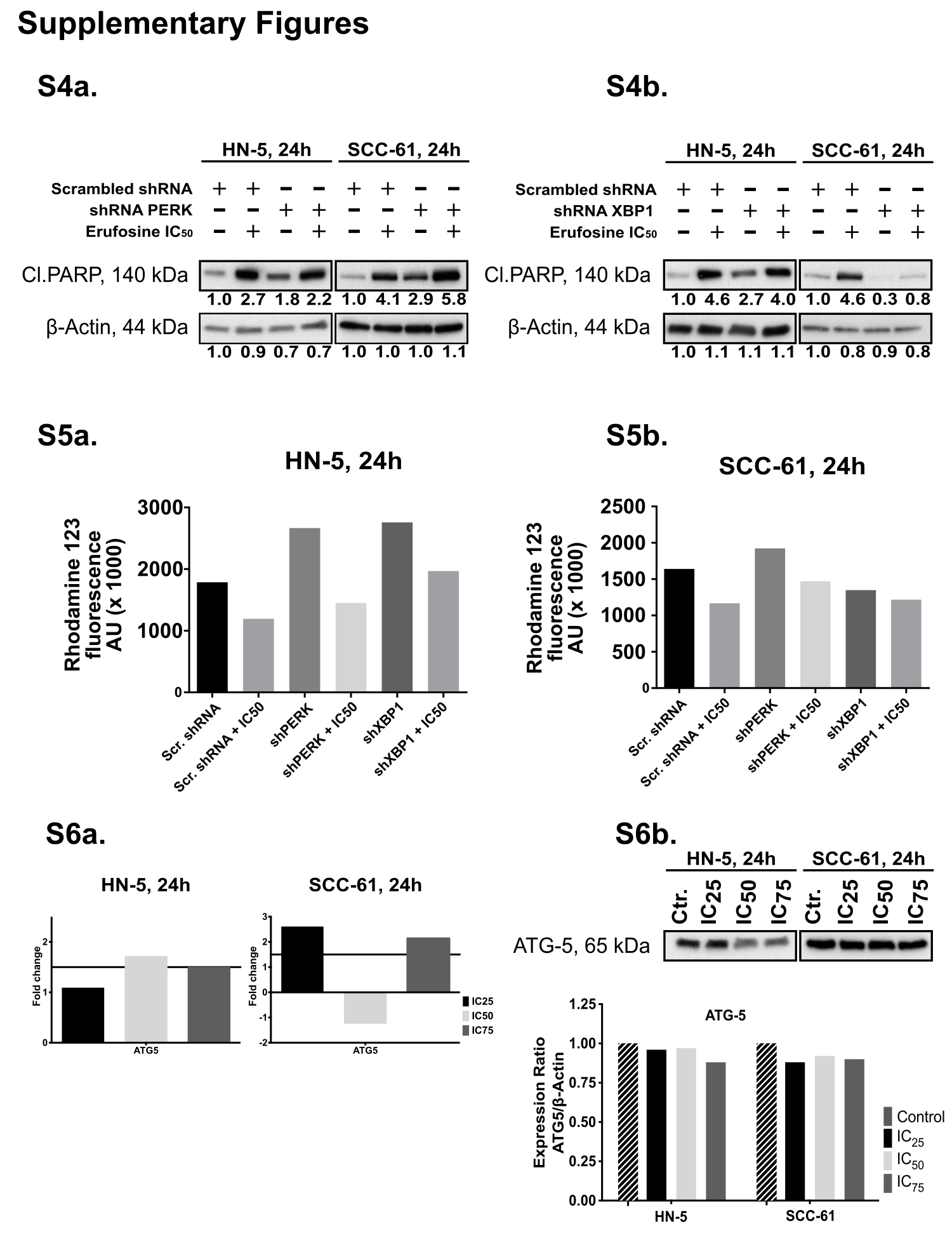

Supplement: Supplementary file 21 — Suppl. Figures S4-S6 [file 41419_2018_342_MOESM21_ESM.png]
