# Supplementary material for: Induction of ER and mitochondrial stress by the alkylphosphocholine erufosine in oral squamous cell carcinoma cells
Source: Cell Death Dis. 2018 Feb 20;9(3):296. doi: 10.1038/s41419-018-0342-2 (PMC5833417; doi:10.1038/s41419-018-0342-2)
Supplement: Supplementary file 22 — Supplementary Figure Legends [file 41419_2018_342_MOESM22_ESM.docx]

**Supplementary Figure Legends**

**Fig. S1a, b:** mRNA expression of XBP1 target genes in two OSCC cell lines post 24h of erufosine exposure. HN-5 cells show only upregulation of HSPA5 whereas SCC-61 cells show high upregulation of all the XBP1 targets. A cut-off of 1.5 fold increase is indicated by a dotted line. The fold change is calculated according to 2^-∆∆CT^ method.

**Fig. S2:** Effect of shPERK knockdown (a) on autophagy in the two OSCC cell lines alone or in combination with erufosine. Increased LC3B level is seen in scrambled cells when exposed to erufosine. The levels are either unchanged in HN5 cells or slightly decreased in SCC-61 cells. The shPERK knockdown cells in combination with erufosine showed no significant changes when compared to untreated knockdown cells. (b) Effect of shXBP1 knockdown (b) on autophagy in two OSCC cell lines alone or in combination with erufosine when compared to scrambled control cells. HN-5 shXBP1 cells showed increased LC3B levels whereas in SCC-61 shXBP1 cells, LC3B levels remained unchanged. Erufosine exposure to knockdown cells increased LC3-B levels in comparison to untreated shXBP1 knockdown cells. Protein level changes were deduced by dividing the densitometry output for each band by that for the corresponding β- actin band.

**Fig. S3:** Morphological changes observed in the OSCC cell lines post erufosine treatment in a dose dependent manner. Cells were incubated in 6 well plates, exposed to the respective IC concentrations and viewed under a light microscope. Signs of cells undergoing apoptosis with distinct morphological changes can be seen in both cell lines.

**Fig. S4:** Changes in cleaved PARP levels were examined to decipher changes in the apoptotic states of scrambled and knockdown cells. (a) Effect on shPERK knockdown cells in the two OSCC cell lines alone or in combination with erufosine. An increased basal level of cleaved PARP was seen in knockdown HN5 and SCC-61 cells when compared with scrambled controls. The cleaved PARP levels were increased upon erufosine exposure in both knockdown cells. (b) Changes in the cleaved PARP levels in shXBP1 knockdown cells in two OSCC cell lines alone or in combination with erufosine when compared to scrambled control cells. HN-5 shXBP1 cells showed increased basal level of apoptosis whereas in SCC-61 shXBP1 cells, the cleaved PARP level was reduced when compared to the scrambled control. Protein level changes were deduced by dividing the densitometry output for each band by that for the corresponding β- actin band.

**Fig. S5:** Dysregulation of mitochondria upon erufosine exposure in shPERK and shXBP1 knockdown cells in (a) HN5 and (b) SCC-61 cells. Measurement of mitochondrial membrane potential (Δψm) with Rhodamine was carried out by flow cytometry. The IC50 concentration of erufosine was used for both cell lines and fluorescence intensity was measured post 24h.

**Fig. S6**: Modulation of ATG-5 at (a) mRNA and (b) protein levels in the two OSCC cell line post 24h of erufosine exposure.
